# Associations between gut microbiota and incident fractures in the FINRISK cohort

**DOI:** 10.1038/s41522-024-00530-8

**Published:** 2024-08-14

**Authors:** Louise Grahnemo, Oleg Kambur, Leo Lahti, Pekka Jousilahti, Teemu Niiranen, Rob Knight, Veikko Salomaa, Aki S. Havulinna, Claes Ohlsson

**Affiliations:** 1 Department of Internal Medicine and Clinical Nutrition, Institute of Medicine, Sahlgrenska Osteoporosis Centre, Centre for Bone and Arthritis Research at the Sahlgrenska Academy, University of Gothenburg, Gothenburg, Sweden; 2 Department of Public Health and Welfare, Finnish Institute for Health and Welfare, Helsinki, Finland; 3 Department of Computing, University of Turku, Turku, Finland; 4 Department of Internal Medicine, University of Turku, Turku, Finland; 5 Division of Medicine, Turku University Hospital, Turku, Finland; 6 Department of Pediatrics, University of California San Diego, La Jolla, CA USA; 7 Center for Microbiome Innovation, Joan and Irwin Jacobs School of Engineering, University of California San Diego, La Jolla, CA USA; 8 Department of Bioengineering, University of California San Diego, La Jolla, CA USA; 9 Department of Computer Science and Engineering, University of California San Diego, La Jolla, CA USA; 10 Institute for Molecular Medicine Finland (FIMM), HiLIFE, University of Helsinki, Helsinki, Finland; 11 Region Västra Götaland, Sahlgrenska University Hospital, Department of Drug Treatment, Gothenburg, Sweden

**Keywords:** Microbiota, Metagenomics

## Abstract

The gut microbiota (GM) can regulate bone mass, but its association with incident fractures is unknown. We used Cox regression models to determine whether the GM composition is associated with incident fractures in the large FINRISK 2002 cohort (*n* = 7043, 1092 incident fracture cases, median follow-up time 18 years) with information on GM composition and functionality from shotgun metagenome sequencing. Higher alpha diversity was associated with decreased fracture risk (hazard ratio [HR] 0.92 per standard deviation increase in Shannon index, 95% confidence interval 0.87–0.96). For beta diversity, the first principal component was associated with fracture risk (Aitchison distance, HR 0.90, 0.85–0.96). In predefined phyla analyses, we observed that the relative abundance of Proteobacteria was associated with increased fracture risk (HR 1.14, 1.07–1.20), while the relative abundance of Tenericutes was associated with decreased fracture risk (HR 0.90, 0.85–0.96). Explorative sub-analyses within the Proteobacteria phylum showed that higher relative abundance of Gammaproteobacteria was associated with increased fracture risk. Functionality analyses showed that pathways related to amino acid metabolism and lipopolysaccharide biosynthesis associated with fracture risk. The relative abundance of Proteobacteria correlated with pathways for amino acid metabolism, while the relative abundance of Tenericutes correlated with pathways for butyrate synthesis. In conclusion, the overall GM composition was associated with incident fractures. The relative abundance of Proteobacteria, especially Gammaproteobacteria, was associated with increased fracture risk, while the relative abundance of Tenericutes was associated with decreased fracture risk. Functionality analyses demonstrated that pathways known to regulate bone health may underlie these associations.

## Introduction

Fragility fractures are commonly caused by osteoporosis, a disease characterized by low bone mass and altered bone microarchitecture^1^. One in two women and one in four men will at some point suffer an osteoporotic fracture^2^. Low bone mineral density (BMD) is the major causal risk factor for fractures^3,4^. In addition, fracture risk is influenced by bone quality parameters and non-skeletal factors such as neuromuscular control and cognition, which influence the risk of falling^5^.

The gut microbiota (GM) can regulate bone mass in rodents and humans^6–9^. Previous human studies on the association between GM composition and bone mass parameters have yielded inconsistent results^10–16^. The inconsistent results may be explained by the small sample sizes and the cross-sectional settings of the previous studies. Orwoll et al.^15^ estimated that large sample sizes (*n* > 6000) are required to account for multiple testing when evaluating associations between the many taxa present at genus/species levels and bone mass parameters. However, a recent targeted study including both a large discovery cohort and a replication cohort identified three bacterial species reproducibly associated with appendicular lean mass^17^. It is unknown if the GM composition is associated with risk of falls or bone quality parameters that also may influence fracture risk in humans. Furthermore, to our knowledge, the associations between GM composition and incident fractures have not been evaluated in a prospective setting.

Thus, previous studies evaluating the associations between GM composition and bone health have been small and often not adjusted for relevant covariates. The present study is substantially larger than previous studies, enabling adjustment for several important covariates. The overall aim of the present study was to determine whether the GM composition is associated with incident fractures, adjusted for multiple relevant covariates, in the large prospective FINRISK 2002 cohort (*n* = 7043, 1092 fracture cases). In this cohort, GM composition and functionality were quantified using metagenome sequencing, and the median follow-up time was 18 years.

## Results

### Alpha and beta diversity measures were associated with incident fracture risk

**Fig. 1:**
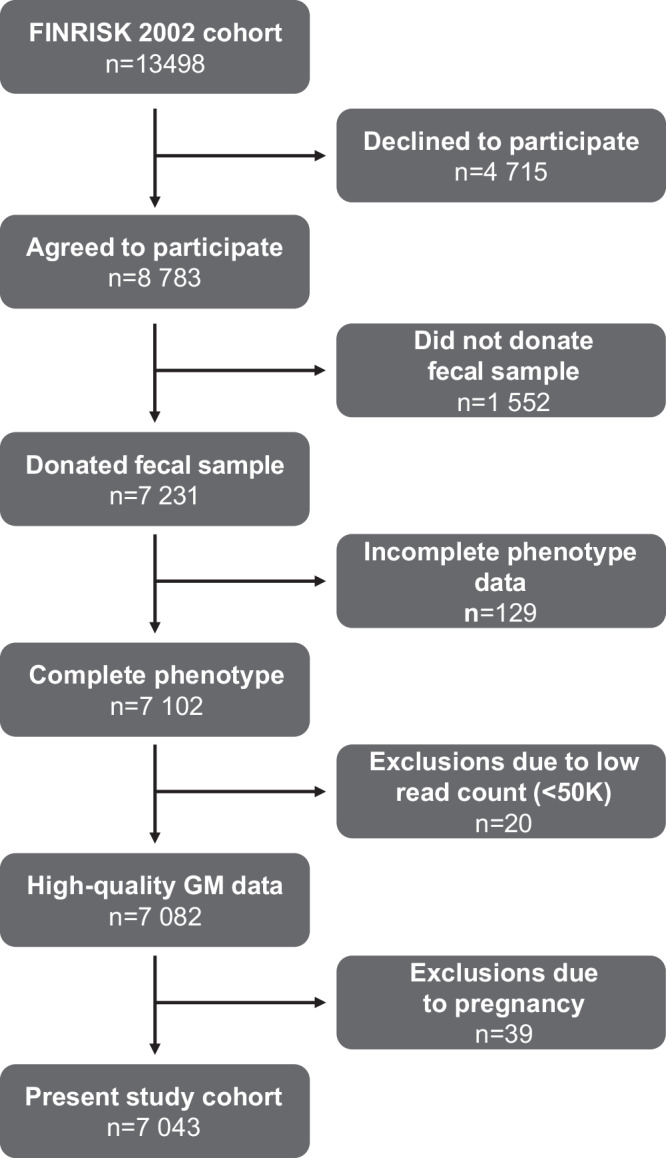
Flow chart. Numbers of excluded and included participants.

**Fig. 2:**
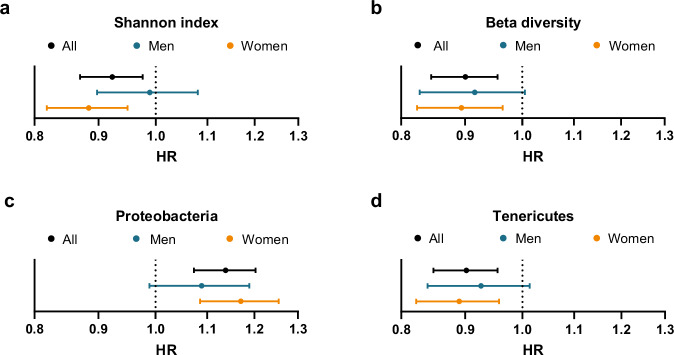
Gut microbiota composition is associated with the risk of incident fractures. Associations for **a** alpha diversity (Shannon index), **b** the first principal component of Aitchison distance (beta diversity), **c** the relative abundance of Proteobacteria, and **d** the relative abundance of Tenericutes in the entire cohort (*n* = 7043 [1092 fractures], men (*n* = 3188 [442 fractures]), or women (*n* = 3,855 [650 fractures]). Cox regressions were adjusted using the main model. Data are hazard ratios (HRs) and 95% confidence intervals (CIs).

We first used the FINRISK 2002 cohort to determine whether measures of the overall GM composition were associated with risk of incident fractures (main outcome, *n* = 1092, Fig. 1, Table 1). Using the main model (adjusted for age, gender, medications, antibiotics, and previous fractures), we observed that higher alpha diversity was associated with decreased risk of fractures (HR 0.92 per standard deviation increase in Shannon index, 95% confidence interval 0.87–0.96, *P* = 0.006, Fig. 2a). For beta diversity, principal component (PC) 1 of the Aitchison distance was associated with fracture risk (HR 0.90, 95% CI 0.85–0.96, *P* = 0.0007; Fig. 2b, Supplementary Table 1). These findings demonstrate that the overall GM composition, as determined by alpha and beta diversity measures, was associated with incident fracture risk.

**Table 1:** Study characteristics

Variable	All (n = 7043)	Cases (n = 1092)	Controls (n = 5951)
Age (years), mean (SD)	49.6 (12.9)	53.2 (12.8)	48.9 (12.8)
Women, *n* (%)	3855 (54.7)	650 (59.5)	3205 (53.9)
Medication use, *n* (%)	2925 (41.5)	559 (51.2)	2366 (39.8)
Antibiotic treatment—6 months, *n* (%)	980 (13.9)	157 (14.4)	823 (13.8)
Antibiotic treatment—1 month, *n* (%)	275 (3.9)	46 (4.2)	229 (3.8)
Prevalent fractures, *n* (%)	610 (8.7)	174 (15.9)	436 (7.3)
Current smoker, *n* (%)	1641 (23.4)^a^	253 (23.4)^b^	1388 (23.4)^a^
Hormone replacement therapy, *n* (%)	959 (13.6)^b^	194 (17.8)	765 (12.9)^b^
Alcohol intake (g/week), median (IQR)	36.0 (9.0–103.5)^c^	30.6 (2.7–105.6)^d^	36.0 (9.0–102.9)^c^
High alcohol intake, *n* (%)	585 (8.7)^c^	102 (9.9)^d^	483 (8.4)^c^
Physical activity level, *n* (%)	^c^	^a^	^d^
basically sedentary	1464 (21.1)	240 (22.5)	1224 (20.9)
light excercise level	3910 (56.4)	609 (57.1)	3301 (56.2)
moderate to high excercise level	1562 (22.5)	217 (20.4)	1345 (22.9)
Healthy food choice score, mean (SD)	200.2 (88.3)^e^	205.7 (86)^c^	199.3 (88.7)^e^
CRP (ng/ml), median (IQR)	1.1 (0.5–2.6)^a^	1.3 (0.6–3)^b^	1.1 (0.5–2.5)^a^
Shannon diversity index, mean (SD)	3.40 (0.43)	3.37 (0.43)	3.40 (0.43)
Follow-up time (years), median (IQR)	17.8 (14.0–17.9)	9.0 (2.5–13.5)	17.8 (17.7–17.9)

Missing individuals:

^a^21–50,

^b^Less than 20,

^c^146–284,

^d^66–90,

^e^1124–1362.

SD standard deviation, IQR, interquartile range

### Proteobacteria and Tenericutes were associated with incident fracture risk

To avoid the challenge with multiple testing described by Orwoll et al.^15^ when evaluating the associations for multiple taxa with bone health parameters, we only performed predefined association analyses for the 10 most abundant phyla with incident fracture risk (Supplementary Table 2–3). Following conservative Bonferroni correction (*p* < 0.05/10 = 0.005 required), the relative abundance of Proteobacteria was associated with increased fracture risk (HR 1.14, 1.07–1.20, *P* = 1.0 × 10^−5^), while the relative abundance of Tenericutes was associated with decreased fracture risk (HR 0.90, 0.85–0.96, *P* = 5.4 × 10^−4^) (Fig. 2c, d, Supplementary Table 3). Following adjustment for multiple testing, all proportional hazard assumptions were met for the four main findings of the study (Supplementary Table 4). The effect sizes of the associations of Proteobacteria and Tenericutes with fractures (main model) were similar after further adjustments for smoking, hormone replacement therapy, alcohol use, physical activity (extended model), and diet (diet model; Supplementary Table 5). In sensitivity analyses, we excluded individuals with antibiotic treatment, previous fractures, inflammatory bowel disease, other major diseases (cardiovascular disease, cancer, diabetes), or fractures within two years after the baseline, with similar results (Supplementary Table 6).

There was a low degree of correlation between Proteobacteria and Tenericutes (Kendall’s tau-b coefficient = −0.17), and the effect sizes where similar when including both phyla in the same Cox regression model (Proteobacteria—individually: HR 1.14, 1.07–1.20; combined: HR 1.12, 1.07–1.20, Tenericutes—individually: HR 0.90, 0.85–0.96; combined: HR 0.92, 0.87–0.98).

Proteobacteria consists mainly of Gammaproteobacteria, Betaproteobacteria, and Deltaproteobacteria; together these three classes had a combined mean relative abundance of 3.17% out of the 3.23% for Proteobacteria. Gammaproteobacteria and Deltaproteobacteria were statistically significantly associated with increased risk of fractures, and for Betaproteobacteria, a similar trend was observed (Supplementary Table 7). The combined relative abundance of Gammaproteobacteria, Betaproteobacteria, and Deltaproteobacteria was robustly associated with increased risk of fractures (HR 1.12; 1.06–1.19, *P* = 6.5 × 10^−5^, Supplementary Table 7).

**Fig. 3:**
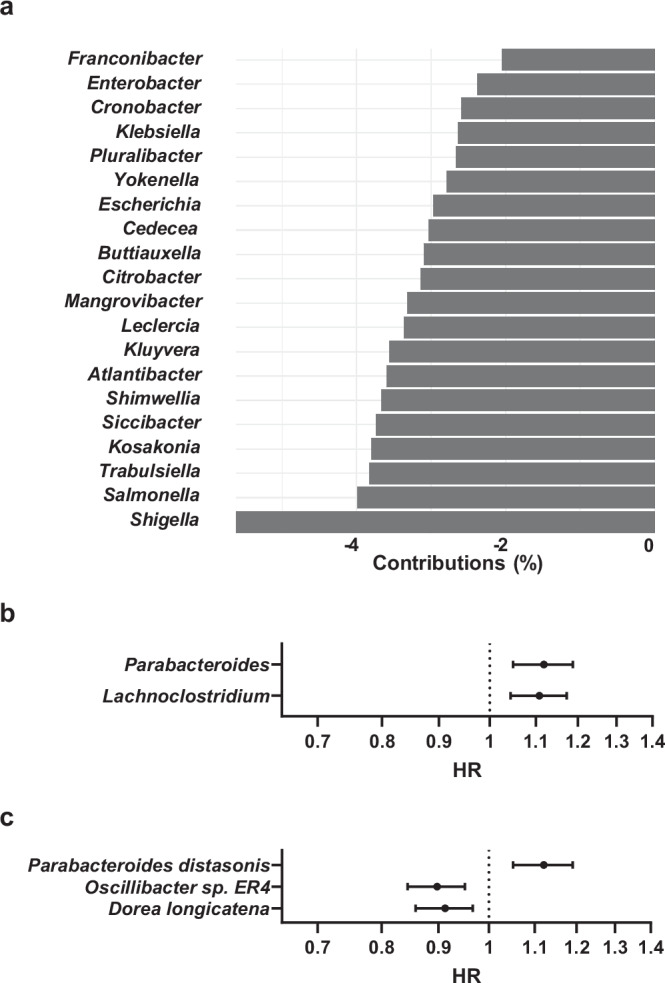
Principal component of genera as well as individual common taxa associated with fracture risk. **a** The 20 most contributing genera of the second principal component. **b**, **c** Among the 25 most common genera and species, **b** two genera and **c** three species were associated with any fracture using Cox regressions adjusted using the main model with FDR correction (Supplementary Tables 9 and 10). **b**, **c** Data are hazard ratios (HRs) and 95% confidence intervals (CIs). *n* = 7043 (1092 fractures).

Furthermore, we used principal component analysis to unconditionally explore the genera that contributed the most to the associations between the overall GM compositional parameters and fractures. These genera analyses showed that both PC1 and PC2 were associated with the risk of fractures (Supplementary Table 8, Supplementary Fig. 1). Interestingly, the top 20 most contributing genera of PC2 were all Proteobacteria that belonged to the Gammaproteobacteria class and the Enterobacteriaceae family (Fig. 3). Among these, the most common genera were the pathogenic genera Escherichia (1.6% mean relative abundance), Shigella (0.13% mean relative abundance), and Klebsiella (0.13% mean relative abundance (Fig. 3a)).

The Tenericutes phylum (0.010% mean relative abundance) consists entirely of the class Mollicutes (0.009% mean relative abundance, HR 0.89, 0.84–0.94), with the orders Mycoplasmatales (0.004% mean relative abundance, HR 0.93, 0.88–0.99), Acholeplasmatales (0.003% mean relative abundance, HR 0.91, 0.86–0.96), and Entomoplasmatales (0.002% mean relative abundance, HR 0.90, 0.85–0.95) as the major contributors to the association with decreased fracture risk.

Explorative analyses of the 25 most abundant genera and the 25 most abundant species suggested that two genera, *Parabacteroides* and *Lachnoclostridium*, and three species, *Oscillibacter sp. ER4*, *Parabacteroides distasonis*, and *Dorea longicatena*, were associated with the risk of fractures passing the FDR threshold (Fig. 3b, c, Supplementary Tables 9–10). In sensitivity analyses, we excluded individuals with antibiotic treatment, previous fractures, inflammatory bowel disease, or fractures within two years after the baseline, with similar results (Supplementary Table 11). The proportional hazard assumption was met for *Lachnoclostridium*, *Parabacteroides*, *Dorea longicatena*, and *Parabacteroides distasonis* but not for *Oscillibacter sp. ER4* (Supplementary Table 12). Since the proportional hazard assumption was not met for *Oscillibacter sp. ER4*, one should be cautious in the interpretation of the observed association between this taxa and fracture risk.

### Gender-stratified analyses

Explorative gender-stratified analyses showed that the Shannon index associated with fractures in women (*n* = 3855, cases *n* = 650, HR 0.88; 0.82–0.95) but not in men (*n* = 3188, cases *n* = 442, HR 0.99; 0.90–1.08) (Fig. 2a). However, we did not observe any statistically significant interaction between Shannon index and gender (*P* = 0.098 for the interaction). The associations for Proteobacteria, Tenericutes, and beta diversity with fracture risk were statistically significant in women but not in men; however, the trends for these associations in the less powered male sub-cohort were similar as observed in women (Fig. 2b–d). For analysis of women alone, no major differences for the associations with fractures were observed after further adjustment for menopause state (Supplementary Table 13).

### Associations between phyla and hip fractures

Further explorative sub-analyses demonstrated that Proteobacteria was associated with increased (HR 1.13, 1.04–1.23) and Tenericutes with decreased (HR 0.90, 0.82–0.99) risk of MOF (hip, humerus, forearm, and vertebral fractures, *n* = 458) with similar effect sizes as observed for fractures at any bone site (Supplementary Fig. 2a, b). The effect sizes were also similar or stronger for hip fractures (*n* = 136) but only statistically significant for Tenericutes (Supplementary Fig. 2a, b).

### GM composition is associated with the inflammatory marker CRP

As GM has been shown to affect inflammation^6,18,19^, a well-known inducer of bone loss, we tested whether the identified GM composition parameters, associated with fracture risk in the present study, also associated with the inflammatory marker CRP. Relative abundance of Proteobacteria was associated with increased levels of CRP (*P* = 6.7 × 10^−7^), while Shannon index (*P* = 9.4 × 10^−11^) and relative abundance of Tenericutes (*P* = 3.2 × 10^−9^) were associated with decreased levels of CRP (Supplementary Table 14). Furthermore, Aitchison PC1 was strongly associated with CRP levels (*P* = 1.2 × 10^−17^, Supplementary Table 14). For all these GM composition parameters, their associations with incident fractures and CRP were in the same direction. Next, we explored whether the strengths of the associations for these GM composition parameters with incident fractures were attenuated by adjustment for CRP; however, the strengths were largely unchanged (Supplementary Table 15).

### GM functional groups are associated with fracture risk

**Fig. 4:**
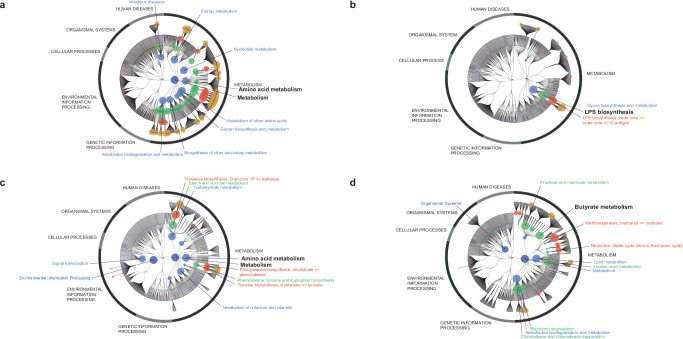
Functionality plots. Functionality plots using **a** the top 25 functional groups associated with decreased fracture risk (main model, Supplementary Table 17), **b** all 10 functional groups associated with increased fracture risk (main model, Supplementary Table 18), **c** the top 25 functional groups (all negatively) correlated with the relative abundance of Proteobacteria and also associated with fracture risk (Supplementary Table 20), and **d** the top 25 functional groups (all positively) correlated with the relative abundance of Tenericutes and also associated with fracture risk (Supplementary Table 22). The node size is determined by the average estimates of the functional groups (Kyoto Encyclopedia of Genes and Genomes orthology [KO] groups) assigned to that node. Estimates are 1/hazard ratio for negative associations (**a**), hazard ratio for positive associations (**b**), and absolute Pearson’s *r* for correlations (**c**, **d**). Labels are shown for nodes with a size ≥ 200. Different colors indicate the different functional layers: biological categories (dark blue), biological processes (light blue), pathways (green), module (red), and KO groups (yellow). Labels for pathways known to regulate bone health are highlighted in bold black. *n* = 7043 (1092 fractures). LPS lipopolysaccharide.

We identified 3017 functional groups, of which 785 (26.0%) were associated with risk of fractures (FDR < 0.05; Supplementary Table 16). Most of the functional groups associated with fractures (775/785) were associated with decreased risk of fractures (Supplementary Table 16). These functional groups were frequently within the biological categories of metabolism, in the process of amino acid metabolism. This finding was more evident among the top 25 associations (Fig. 4a, Supplementary Table 17). Among the functional groups associated with increased risk of fractures, the strongest association belonged to the biological category of glycan biosynthesis and metabolism, in the process of lipopolysaccharide (LPS) biosynthesis (Fig. 4b, Supplementary Table 18).

To identify mechanisms that may mediate the observed associations for Proteobacteria and Tenericutes with fracture risk, we determined the relationships between the relative abundances of Proteobacteria and Tenericutes, functional groups, and risk of fractures. Interestingly, we found that 119 out of the 150 (79.3%) functional groups most strongly correlated with the relative abundance of Proteobacteria and 147 out of the 150 (98.0%) functional groups most strongly correlated with the relative abundances of Tenericutes were also statistically significantly (*P* < 0.05) associated with fracture risk (Supplementary Tables 19, 21). Using binomial tests, we found a strong enrichment for functional groups associated with fractures among the 150 functional groups most strongly associated with Proteobacteria (*P* = 3.2 × 10^−42^) and Tenericutes (*P* = 2.5 × 10^−81^), suggesting that these functional groups may be involved in the associations for Proteobacteria and Tenericutes with fracture risk.

For the top 25 functional groups correlated with the relative abundance of Proteobacteria and associated with fractures, we found that these functional groups were negatively correlated with the relative abundance of Proteobacteria and frequently within the process of amino acid metabolism (Fig. 4c, Supplementary Table 20). The amino acid-related correlations for Proteobacteria included a negative correlation for a functional group involved in the synthesis of branched-chain amino acids (leucine dehydrogenase, Supplementary Table 20), a group of amino acids previously associated with bone health parameters^20,21^. For the top 25 functional groups correlated with the relative abundance of Tenericutes and associated with fractures, we found that these groups were positively correlated with relative abundance of Tenericutes, with the third most strongly correlated group belonging to the pathway for metabolism of the short-chain fatty acid butyrate (Fig. 4d, Supplementary Table 22). We also explored the correlation between downstream taxa of Tenericutes and butyrate metabolism (K00100) and found strong correlations with class (Mollicutes: *r* = 0.52, *P* < 5.9 × 10^−323^) and order (Mycoplasmatales: *r* = 0.35, *P* = 8.9 × 10^−204^; Acholeplasmatales: *r* = 0.36, *P* = 2.3 × 10^−214^; Entomoplasmatales: *r* = 0.38, *P* = 1.3 × 10^−242^) levels. For the relative abundance of Gammaproteobacteria, the strongest positive correlation was for a functional group belonging to the process of LPS biosynthesis (K03280, *r* = 0.37, FDR-adjusted *P* = 2.8 × 10^−226^).

## Discussion

Although the GM can regulate bone mass^6,8,9^, it was unknown if GM composition is associated with incident fractures. Here, we demonstrated that the overall GM composition was associated with incident fractures in the large prospective FINRISK 2002 cohort. We observed that measures of both alpha and beta diversity were associated with fracture risk. At the phyla level, the relative abundance of Proteobacteria was associated with increased fracture risk, while the relative abundance of Tenericutes was associated with decreased fracture risk. The association between the Proteobacteria and fracture risk was driven by genera of the Gammaproteobacteria class and the Enterobacteriaceae family, including the pathogenic genera Escherichia, Shigella, and Klebsiella. Furthermore, functionality analyses demonstrated that pathways known to regulate bone health may underly the identified associations between GM composition parameters and incident fractures.

Our finding that higher alpha diversity was associated with decreased fracture risk is in line with a previous study showing that osteoporosis was associated with decreased alpha diversity^12^, while another study showed the opposite^16^. However, as these two previous cross-sectional studies included a low number of participants (*n* < 100), the robustness of these prior findings are limited. Gender-stratified analyses showed that low Shannon index was associated with high fracture risk in women but not in men. However, formal interaction analyses did not show a statistically significant interaction between Shannon index and gender.

Proteobacteria—especially Gammaproteobacteria—has previously been associated with mortality^22^ and several diseases such as obesity, inflammatory bowel disease, and irritable bowel syndrome^23^, and now also with incident fractures. A negative effect of Proteobacteria on bone health is supported by a small study of 104 postmenopausal women, demonstrating that Proteobacteria and genera within this phylum were enriched in women with low BMD^12^.

Tenericutes are small bacteria devoid of cell walls that may cause pneumonia and urogenital tract disease^24^. However, the relative abundance of Tenericutes phylum was lower in individuals with some inflammation-related disorders compared with controls^25,26^. In the present study, the relative abundance of Tenericutes was associated with decreased risk of incident fractures, supporting a health-promoting effect of Tenericutes.

For the 25 most common genera and species, explorative analyses showed that two genera and three species were associated with the risk of fractures passing the FDR threshold. Our finding that *Parabacteroides* and *Parabacteroides distasonis* were associated with increased fracture risk is in line with some small studies suggesting that higher relative abundance of *Parabacteroides* is associated with osteoporosis (control subjects *n* = 31–64, osteoporotic subjects *n* = 42–44)^12,27^ and that higher relative abundance of *Parabacteroides distasonis* is associated with lower BMD (*n* = 499)^11^. In our study, *Lachnoclostridium* was also associated with increased fracture risk, similarly to a small study suggesting that osteoporotic women (*n* = 34) had higher relative abundance of *Lachnoclostridium* than control subjects (*n* = 51)^28^. Furthermore, *Dorea longicatena* has previously been associated with increased lean mass in the large HUNT cohort (*n* = 5196)^17^, a finding that may help to explain the association between high relative abundance of *Dorea longicatena* and decreased fracture risk in the current study. Apart from determining associations between the most common genera and species, we also determined the genera that contributed most to the first and second principal components. The principal component analysis was not restricted to common genera. Except for *Escherichia*, the most contributing genera were not common, and thus the 2 genera and the 3 species associated with fracture risk in our explorative analyses did not belong to the most contributing genera of the first or second principal component.

Inflammation is known to cause bone loss mainly by increasing bone resorption^29^. Inflammation-induced bone loss is driven by pro-inflammatory cytokines that can induce the formation of CRP, a clinical marker of inflammation^29,30^. It has also been reported that high CRP is associated with increased risk of incident fractures^31^. As inflammation has been shown to be affected by the GM^6,18,19^, we tested whether the GM composition was associated with CRP. The relative abundance of Proteobacteria—associated with increased fracture risk—was associated with increased CRP levels, while the relative abundance of Tenericutes and the Shannon index—associated with decreased fracture risk—were associated with decreased CRP levels. In line with our results, low GM diversity has previously been associated with increased CRP levels^32^. Together, these findings imply that our observed associations between GM composition parameters and fracture risk may be mediated via CRP. However, when we included CRP as a covariate in the fracture models, the effect sizes were largely unaffected, indicating that other factors are more important for the observed associations.

Our functionality analyses demonstrated that pathways related to amino acid metabolism were associated with fracture risk. The relative abundance of Proteobacteria was negatively correlated with the synthesis of branched-chain amino acids, known to associate with bone health parameters^20,21^; thus, this finding may contribute to the observed association between higher relative abundance of Proteobacteria and increased fracture risk. In line with these data, circulating levels of several amino acids have been reported to associate with decreased fracture risk^20^, and a recent Mendelian randomization study demonstrated that high levels of genetically determined valine and isoleucine, two branched-chain amino acids, increase BMD^21^.

In the present study, functional analyses also showed that LPS biosynthesis was related to increased fracture risk and increased relative abundance of Gammaproteobacteria. LPS is a strong inducer of inflammation, resulting in elevated levels of proinflammatory cytokines and, thereby, inflammation-induced bone loss^33^. Gram negative bacteria such as Gammaproteobacteria are the source of LPS which enters the circulation in increased amounts when the gut permeability is increased^34^. Based on these findings, we propose that high levels of Gammaproteobacteria may increase circulating LPS and, thereby, result in inflammation-induced bone loss, contributing to the increased fracture risk observed in the present study.

Some GM species ferment fibers into short-chain fatty acids like butyrate. These bacterial metabolites have been shown to provide benefits for the host. In mice, short-chain fatty acids have been shown to increase both bone and muscle mass^35,36^, and in humans, high circulating butyrate levels have been causally associated with increased lean mass^37^. Both low bone and muscle mass are associated with increased fracture risk^38,39^. In our study, high relative abundance of Tenericutes was associated with decreased fracture risk. This finding might be explained by the fact that Tenericutes, including the downstream class Mollicutes and orders Mycoplasmatales, Acholeplasmatales, and Entomoplasmatales, correlated with enhanced butyrate metabolism and, thereby, promote musculoskeletal health. This notion is supported by the present finding that the relative abundance of Tenericutes was positively associated with butyrate biosynthesis.

Our study has several strengths including the large size of the cohort (*n* = 7043) with a high number of incident fracture cases (*n* = 1092), the long follow-up time (18 years), and the shotgun metagenome sequencing. However, our study also has limitations. Our study mainly included participants of north European descent, limiting the generalizability across populations. For FINRISK, in general, there are differences between participants and non-participants; lower socioeconomic class and existing health issues are associated with non-participation^40,41^. Also, younger people have been less likely to participate. Non-participation related to not giving stool samples has not been studied. Our stool samples were stored in −20 °C for 15 years, and small changes in relative abundances of some rare taxa cannot be excluded. However, the main features of microbiome are known to be fairly stable in different conditions^42–45^, and our analyses were focused on common taxa. Furthermore, all samples were processed similarly, and it is unlikely that prolonged storage could distort the associations with future fractures during the follow-up. Although our shallow shotgun metagenome sequencing provides enhanced taxonomic resolution over 16 S sequencing, the taxonomic resolution is still lower than for deep metagenome sequencing. However, shallow metagenome sequencing has been shown to highly correlate with deep metagenome sequencing data^46^. As Orwoll et al. suggested that large sample sizes are needed to determine associations between GM and bone health parameters^15^, we restricted the predefined analysis to the overall GM composition (alpha and beta diversities) and the ten most abundant phyla to avoid challenges with multiple testing. Subsequently, we may have missed associations for rare phyla. In addition, as we only had information on the GM at baseline, we could not consider changes of the GM during follow-up. Also, baseline BMD data are missing in FINRISK; therefore, we could not determine if the identified taxa are associated with fracture risk independently of BMD. Although the present study is the largest thus far within the bone field, even larger studies or meta-analyses of several cohorts are needed to study associations at the species level, especially for dichotomous outcomes such as incident fractures. Another limitation is that we do not provide direct causal evidence for the role of Proteobacteria or Tenericutes on fracture risk.

In conclusion, the overall GM composition was associated with incident fractures in the FINRISK 2002 cohort. The relative abundance of Proteobacteria, especially Gammaproteobacteria, was associated with increased fracture risk, while the relative abundance of Tenericutes was associated with decreased fracture risk. Functional analyses demonstrated that pathways known to regulate bone health may underly these associations. When additional large-scale cohort studies with metagenome sequence data and a substantial number of incident fractures are available, meta-analyses of available studies should be performed to validate the present findings across populations. These future large-scale meta-analyses will also give the opportunity to perform well-powered studies to identify specific species associated with incident fracture risk.

## Methods

### Study participants

In Finland, the FINRISK cohort study have collected data on risk factors for cardiovascular disease every 5 years since 1972^47^. In the current study, we used the FINRISK 2002 cohort for which permanent Finnish residents (at least 1 year of residency and a personal identification code) were eligible to participate if they were 25–74 years of age and living in one of six different regions in Finland: North Karelia, Northern Savo, Oulu, Lapland, Turku and Loimaa, or Helsinki and Vantaa. Eligible participants were randomly selected (stratified by gender, determined by the social security number, and 10 year age groups) through the National Population Information System (http://www.vrk.fi/en). In 2002, 13498 individuals were invited and 8783 participated, of which 7231 participants donated fecal samples, and among these, 7102 participants had sufficient phenotype data for the main model used in the present study. Next, we excluded 20 participants that had low-read counts (<50 K) and, finally, additionally 39 participants that were pregnant at baseline. The resulting 7043 eligible participants were all included in the current study to maximize power (Fig. 1). Baseline visits took place during a 3 month period in the beginning of 2002 (2002 January 13–2002 April 19). All relevant ethical regulations were followed when performing the study. The Coordinating Ethics Committee of the Helsinki University Hospital District (Helsinki, Finland) approved the study protocol for FINRISK 2002 (ref. no. 558/E3/2001), and all participants provided written informed consent.

### Questionnaires

Participants responded to questionnaires regarding their physical activity, alcohol and dietary intake, smoking status, and use of hormone replacement therapy. The physical activity was assessed as the general level of leisure time physical activity, coded as: (1) basically sedentary; (2) light exercise; (3) moderate to high level conditioning activity, like running, skiing etc., or competitive sports. Alcohol consumption was quantified as the average weekly pure alcohol use in grams during the past 12 months. To indicate very high intake as a risk factor, the alcohol consumption variable was dichotomized, with alcohol consumption ≥231 g/week coded as 1 and <231 as 0 (as implemented in the Finnish version of the fracture risk calculator FRAX, https://frax.shef.ac.uk/FRAX/tool.aspx?lang=fi). Current smokers were defined as participants that smoked daily at baseline. Healthy dietary intake was calculated as a healthy food choice score by adding the food propensity questionnaire responses for intake of food items recommended in the Nordic Nutrition Recommendations^48^: fish; poultry, fruits; berries; fresh, non-sweetened berry and fruit juices; vegetables (including beans and lentils); nuts and seeds; low-fat cheeses; salad dressings and oils; and fiber-rich breads^49^.

### Medication

Several medications are known to affect the gut microbiota^50^. Therefore, we defined the participants as users or non-users of medications starting with ATC codes^51^ A (alimentary tract and metabolism), B (blood and blood forming organs), C (cardiovascular system), G (genito urinary system and sex hormones), H (systemic hormonal preparations, excl. sex hormones and insulins), L (antineoplastic and immunomodulating agents), N (nervous system), or P (antiparasitic products, insecticides and repellents) based on their prescribed drug purchases within 3 months before baseline. Similarly, antibiotic treatment was defined as purchases of antibiotics (ATC codes starting with J01) within 6 months before baseline for adjustments in models. In addition, we performed sensitivity analyses in which participants that purchased antibiotics within 1 month before baseline were excluded from the analyses. Data were obtained from the National Prescribed Drug Purchase Registry.

### Biomarker analysis

High sensitive-CRP was quantified in serum samples using the Architect ci8200 Chemistry Analyzer (Abbott Laboratories, USA).

### Fracture assessment

We recorded both previous fractures that occurred before the baseline collection of the samples for GM analyses and incident fractures that occurred after the collection of the samples for GM analyses. In the analyses of fracture risk, we only evaluated incident fractures, but we adjusted for previous fractures. Fractures were assessed from the start of the register in 1969 or birth date of each participant, whichever occurred later, to the diagnosis of fracture, death, or the end of follow-up (31 December 2019) using International Classification of Diseases (ICD) codes in registries (Supplementary Table 23). The fracture data in these registries are derived from medical records. Each permanent resident in Finland is given a personal identity number, which can be linked to public health records and registers. This enables the linking of study data with broader health records. The records ensure practically complete coverage of all significant health events for an individual’s lifetime in Finland. Only those few participants who moved permanently abroad before health events or 31st of December 2019 were lost to follow-up. The reliability of Finnish health records has been verified^52,53^. Incidence of any fracture was the primary endpoint, while hip and major osteoporotic fractures (MOF) were secondary outcomes. MOF were defined as a fracture of the hip, spine, wrist, or humerus.

### Assessment of major diseases

To identify participants as having prevalent inflammatory bowel disease, cancer, cardiovascular disease, or diabetes, we used (1) ICD codes from the Care Register for Health Care (hospital discharges and specialized outpatient care), (2) Anatomical Therapeutic Chemical (ATC) codes from the Drug Reimbursement and Purchase Registers, (3) Nordic Medico Statistical Committee (NOMESCO) codes, (4) Finnish hospital league codes, (5) national heart patient codes, or (6) reimbursements from Kela (The Social Insurance Institution of Finland). Unless otherwise specified, codes matching the start of the specified code are used to identify individuals with disease. Individuals with inflammatory bowel disease were defined by having Crohn’s disease (ICD-10 K50, ICD-9 555, or ICD-8 5630), ulcerative colitis (ICD-10: K51, ICD-9: 556 but not 5564 A, or ICD-8: 5631 or 569), or by receiving a special reimbursement for Crohn’s disease or ulcerative colitis from Kela (codes 208 or 209). Individuals with cancer were defined by ICD-10 codes (C0–C3, C40–C43, C45–C49, or C5–C9) or ICD-8/9 codes (14–16, 170–172, 174–179, 18–19, or 200–208). Individuals with cardiovascular disease were defined by having experienced stroke (ICD-10 I161 or I163–164 but not I636; ICD-9 431, 4330 A, 4331 A, 4339 A, 4340 A, 4341 A, 4349 A, or 436; or ICD-8 code 431, 433, 434, 436 but not 43101 or 43191), coronary artery disease (CAD; ICD-10: I200 or I21–121, ICD-8/9: 410 or 4110), or procedures related to CAD (NOMESCO codes FNF, FNG, TFN40, FN1AT, FN1BT, FN1YT, FNA, FNB, FNC, FND or FNE; Finnish hospital league codes 5311–5315; national heart patient codes exactly matching 82–84, 11, 25, 111–113, or 119 or starting with AN2–AN4, ANA, ANB, AA, AA2, AA3, or AAX). Individuals with diabetes were defined by ICD-10 codes E10–E14, ICD-8/9 code 250, Kela drug reimbursement code 103 or 215, purchases of diabetes medication (ATC A10), minimum three purchases if no other register code was found.

### Fecal sample collection, DNA extraction, and library collection

During the baseline survey, stool samples were collected by willing participants at home using an ad hoc kit constructed in-house in the Finnish Institute for Health and Welfare (THL) with detailed instructions and a scoop method. No preservative was used in the sampling tubes (50 ml Falcon tubes). The participants mailed their samples overnight between Monday and Thursday under Finnish winter conditions (from January through March 2002) to the laboratory of THL where the samples were immediately frozen at −20 °C. Special care was taken to avoid delayed transit at the post office over the weekend. The stool samples were stored unthawed at −20 °C until they were transferred in 2017 to the University of California, San Diego for microbiome sequencing, as previously described^54^. In brief, fecal DNA was extracted using the MagAttract PowerSoil DNA Kit (Qiagen) and Earth Microbiome Project protocols^55^. The library was generated by a miniaturized version of the Kapa HyperPlus Illumina-compatible library prep kit (Kapa Biosystems)^56^. DNA extracts were normalized to 5 ng per sample using an Echo 550 robot (Labcyte, Inc.). Enzymatic fragmentation, end-repair, and adapter-ligation were performed using a Mosquito HV robot (TTP Labtech, Ltd). Following barcoding and amplification, the libraries were sequenced on an Illumina HiSeq 4000 instrument, producing on average 900,000 reads per sample.

### Taxonomic profiling

We analyzed shotgun metagenomic sequences using a pipeline built with the Snakemake bioinformatics workflow library (https://github.com/biocore/oecophylla)^57^. We trimmed the sequences for quality and adapter sequences using Atropos^58^ and removed host reads by genome mapping against the human genome assembly GRCh38 with Bowtie2^59^. We assigned sequences to taxonomy using SHOGUN v1.0.5^60^ against a database containing all complete bacterial, archaeal, and viral genomes available from NCBI RefSeq as of version 82 (May 8, 2017). SHOGUN calls Bowtie2 to align sequencing data against reference genomes. For each query sequence, up to 16 hits were returned to maximize the inclusion of closely related organisms to which the query sequence matches equally or similarly well (i.e., they all have a chance of being the true positive). As a trade-off, this behavior could potentially result in a larger number of organisms than Bowtie2’s default behavior, which returns one hit per query. We then processed the results to estimate the relative abundance of taxa.

### Functional profiling

Functional profiles were calculated from a combination of observed and predicted Kyoto Encyclopedia of Genes and Genomes orthology (KO) group annotations from the RefSeq genomes following the default parameters of the SHOGUN tool, as previously described^60^. Briefly, the final KO table represents a weighted average of directly observed functional genes and those estimated to be present but unsampled, based on their predicted presence within an observed genome.

### Statistics

#### Software

Data was statistically analyzed using R (version 3.6.3, https://www.R-project.org/), while data was visualized using R, GraphPad Prism (version 9.5.1), or FuncTree (Yamada Lab, Tokyo Institute of Technology, Tokyo, Japan^61^).

#### Variable calculations and transformations

Prior to analyses, the microbiome data was filtered to exclude taxa representing viruses, archaea, or plasmids, along with taxa present in <3 subjects. The abundance data was then compositionally transformed for the analyses, except for alpha diversity. Shannon index was used as the measure of alpha diversity, calculated directly from the filtered taxa count data at the species level using the R packages *microbiome* (version 1.8.0) and *vegan* (version 2.5.7). Aitchison distance was used as a measure of beta diversity, calculated using the R packages *microbiome* and *vegan* after centered log-ratio (CLR) transformation to reduce the distribution skewness. Principal component analysis was performed on Aitchison distance and CLR-transformed taxa abundances using the R-package *stats* (version 3.6.2). Individual taxon abundances (phylum, class, order, family, genus, and species) were CLR-transformed. For the pathway analysis, the predicted KO groups were used. For each sample, the relative KO group abundances were gathered from the strain-level data and log10-transformed to reduce the skewness. CRP was divided into quartiles. For relevant comparisons between effect sizes, we standardized the exposure and continuous outcome variables.

#### Statistical analyses

We assessed associations between GM metrics (composition and function) and risk of fractures using Cox regression. In all models, age was used as the time scale^62^ and gender as stratum. Several covariate adjustments were used. In the main model, we adjusted for established factors strongly associated with GM composition and/or fracture risk (medication, antibiotics, and previous fractures). In the extended model, we also adjusted for several other covariates that also may confound the association between GM composition and fracture risk (main model plus smoking, hormone replacement therapy, alcohol use, and physical activity). Diet is a major determinant of the GM composition, but information on diet was only available in a subsample of the present cohort. Therefore, we added a third model also adjusted for diet (the diet model – extended model plus diet), including lower number of subjects. For associations between GM metrics and CRP quartiles, we used linear regressions adjusted using the following covariates: age, gender, medication, antibiotics, and previous fractures. We assessed Pearson’s correlation between functional groups and gut microbial composition. Our predefined main exposures were the 10 most abundant phyla. To correct for multiple testing, we used conservative Bonferroni correction for associations between the 10 most abundant phyla evaluated and fracture risk (*P* ≤ 0.005 [0.05/10] was considered statistically significant). For the large number of explorative analyses of functionality and at the genus and species levels, we used Benjamini-Hochberg multiple testing false discovery rate correction (FDR; corrected *P* < 0.05 was considered statistically significant). All tests were 2-sided.

To visualize relative importance of functional pathways related to fracture risk or taxa, we used FuncTree^61^ to create separate plots for positive (plot based on hazard ratio [HR]) and negative associations (plot based on 1/HR) and positive and negative correlations (plots based on absolute values for Pearson’s r).

#### Handling of missing data

We did not impute missing values. In the main model (adjusting for age, gender, medication, antibiotics, and previous fractures), we included 7043 individuals of which 1092 had sustained a fracture, while the extended model (further adjusting for smoking, hormone replacement therapy, alcohol use, and physical activity) included 6641 participants of which 998 had sustained a fracture, and the diet model (further adjusted for diet) included 5460 participants of which 809 had sustained a fracture. The extended and diet models included fewer participants as they were missing information on one or more of the covariates used in these models: 35 for smoking, 3 for hormone replacement therapy, 284 for alcohol use, 107 for physical activity, and 1362 for diet.

### Supplementary information

Supplementary Figures and Tables

## Data Availability

The FINRISK 2002 data described in the manuscript are available from the Finnish Institute for Health and Welfare Biobank based on a written application as instructed on the website of the Biobank (https://thl.fi/en/web/thl-biobank/for-researchers/application-process). The phenotype data are not publicly available because they contain information that could compromise research participant privacy/consent. The metagenomic data are available from the European Genome-Phenome Archive (accession number EGAD00001007035).
