# Supplementary material for: Associations between gut microbiota and incident fractures in the FINRISK cohort
Source: NPJ Biofilms Microbiomes. 2024 Aug 14;10:69. doi: 10.1038/s41522-024-00530-8 (PMC11324742; doi:10.1038/s41522-024-00530-8)
Supplement: Supplementary file 1 — Supplementary Figures and Tables [file 41522_2024_530_MOESM1_ESM.pdf]

SUPPLEMENTARY INFORMATION
Associations between gut microbiota and incident fractures in the FINRISK cohort

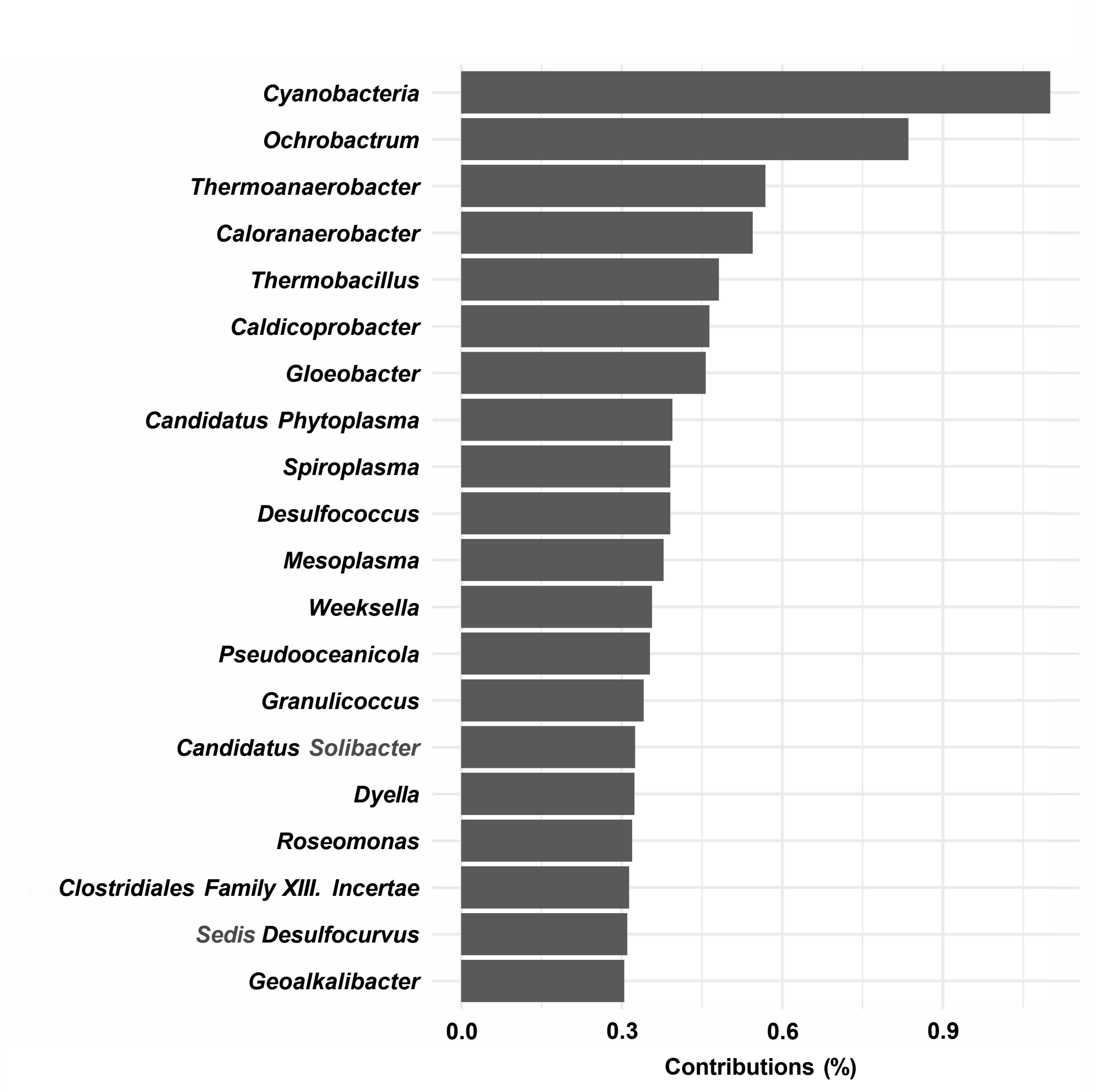

Supplementary Figure 1. The 20 most contributing genera of the first principal component belong to a mixture of phyla and classes. Principal component analysis was performed on the abundance of genera.

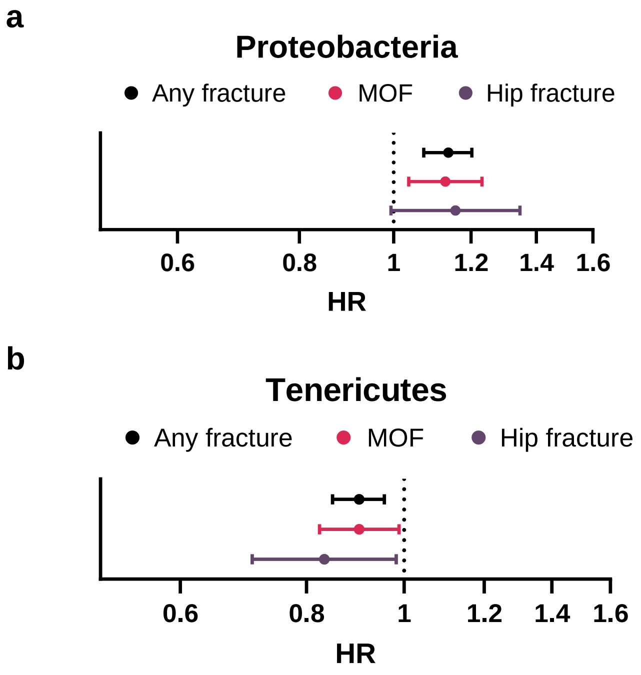

Supplementary Figure 2. Associations between phyla and different fracture types. Associations for **a** the relative abundance of Proteobacteria and **b** the relative abundance of Tenericutes with fracture at any bone site (1,092 fractures), major osteoporotic fracture (MOF, 458 fractures), and hip fractures (136 fractures). Cox regressions were adjusted using the main model. Data are hazard ratios (HR) and 95% confidence intervals (CI).

Supplementary Table 1. Associations between beta diversity and risk of fractures

Predictor	HR	2.5% CI	97.5% CI	P	Variance explained (%)
Aitchison distance					
PC 1	0.90	0.85	0.96	6.8E-04	86.1
PC 2	0.96	0.91	1.02	2.3E-01	4.3
PC 3	1.02	0.96	1.08	5.9E-01	1.3

Cox regressions for the main model (adjusted for age, gender, medication, antibiotics, and previous fractures) with principal components (PCs) as exposure and risk of fractures as outcome. n = 7043 (1092 fractures). HR, hazard ratio; CI, confidence interval

Supplementary Table 2. The relative abundance of the 10 most abundant phyla

Phyla	All (n=7043)	Cases (n=1092)	Controls (n=5951)
Bacteroidetes	51.4 (38.3–63.3)	52.3 (38.8–64.5)	51.3 (38.2–63.1)
Firmicutes	40.3 (30.0–52.0)	39.6 (28.2–50.8)	40.5 (30.4–52.2)
Actinobacteria	2.22 (1.11–4.48)	2.06 (1.02–4.22)	2.25 (1.13–4.52)
Proteobacteria	1.66 (0.97–2.86)	1.71 (1–3.12)	1.65 (0.96–2.83)
Verrucomicrobia	0.09 (0.01–0.80)	0.09 (0.01–0.87)	0.09 (0.01–0.80)
Spirochaetes	0.009 (0.006–0.014)	0.009 (0.005–0.013)	0.009 (0.006–0.014)
Cyanobacteria	0.008 (0.005–0.012)	0.007 (0.004–0.012)	0.008 (0.005–0.012)
Tenericutes	0.004 (0.001–0.011)	0.003 (0.001–0.011)	0.004 (0.001–0.011)
Synergistetes	0.003 (0.002–0.006)	0.003 (0.002–0.006)	0.003 (0.002–0.006)
Fusobacteria	0.003 (0.001–0.006)	0.003 (0.001–0.005)	0.003 (0.001–0.006)

Data are median (interquartile range). These phyla were dectectable in ≥ 98.9 of the participants

Supplementary Table 3. Associations between the relative abundances of the 10 most abundant phyla and risk of incident fractures

Phyla	HR	2.5% CI	97.5% CI	P
Bacteroidetes	1.08	1.02	1.15	0.011
Firmicutes	1.03	0.97	1.10	0.292
Actinobacteria	1.04	0.98	1.11	0.205
**Proteobacteria**	**1.14**	**1.07**	**1.20**	**1.0E-05**
Verrucomicrobia	1.01	0.95	1.07	0.829
Spirochaetes	0.98	0.92	1.04	0.422
Cyanobacteria	0.99	0.93	1.05	0.771
**Tenericutes**	**0.90**	**0.85**	**0.96**	**5.4E-04**
Synergistetes	0.97	0.91	1.03	0.331
Fusobacteria	1.00	0.94	1.06	0.962

Cox regressions with fractures as outcome, relative abundance of phyla as exposure, and adjusted using the main model (including age, gender, medication, antibiotics, and previous fractures as covariates). Phyla that passed the Bonferonni-corrected significance level (a = 0.05/10 = 0.005) are shown in bold. n = 7043 (1092 cases). HR, hazard ratio; CI, confidence interval

Supplementary Table 4. Associations between gut microbial compositional parameters with risk of incident fractures and corresponding tests for proportional hazards assumption.

					Proportional hazards assumption	
**Predictor**	**HR**	**2.5% CI**	**97.5% CI**	**P cox**	**P exposure variable**	**P whole model**
Shannon index	0.92	0.87	0.98	5.9E-03	0.45	0.87
Beta diversity (Aitchison PC1)	0.90	0.85	0.96	6.8E-04	0.02	0.20
Proteobacteria	1.14	1.07	1.20	1.0E-05	0.30	0.75
Tenericutes	0.90	0.85	0.96	5.4E-04	0.32	0.79

Cox regressions with fractures as outcome, compositional parameters as exposure, and adjusted using the main model (including age, gender, medication, antibiotics, and previous fractures). For tests of proportinal hazards assumption, 0.0125 (0.05/4) was considered statistically significant. n = 7043 (1092 cases). HR, hazard ratio; CI, confidence interval; PC, principal component

Supplementary Table 5. Associations between the relative abundances of Proteobacteria and Tenericutes with risk of incident fractures

Phyla	Model	HR	2.5% CI	97.5% CI	P	n total	n fractures
Proteobacteria	main	1.14	1.07	1.20	1.0E-05	7043	1092
	extended	1.12	1.05	1.19	2.2E-04	6641	998
	diet	1.11	1.04	1.19	2.2E-03	5460	809
Tenericutes	main	0.90	0.85	0.96	5.4E-04	7043	1092
	extended	0.92	0.87	0.98	1.5E-02	6641	998
	diet	0.91	0.85	0.97	6.4E-03	5460	809

Cox regressions with fractures as outcome, relative abundance of phyla as exposure, and adjusted using the main model – age, gender, medication, antibiotics, and previous fractures; the extended model – main model plus smoking, hormone replacement therapy, alcohol use, and physical activity; and the diet model – extended model plus diet. HR, hazard ratio; CI, confidence interval

Supplementary Table 6. Sensitivity analyses excluding participants with previous fractures, antibiotic treatment, fractures within 2 years after baseline, inflammatory bowel disease, or other major diseases

	HR	2.5% CI	97.5% CI	P	n total	n fractures
**Shannon index**						
All	0.92	0.87	0.98	5.9E-03	7043	1092
Excluding previous fractures	0.91	0.86	0.97	4.2E-03	6433	918
Excluding antibiotic treated	0.93	0.88	0.99	1.6E-02	6768	1046
Excluding fractures within 2 years after baseline	0.92	0.86	0.98	8.6E-03	6793	842
Excluding prevalent inflammatory bowel disease	0.92	0.87	0.97	4.4E-03	6985	1083
Excluding prevalent cancer, CVD, and diabetes	0.92	0.87	0.98	9.4E-03	6343	953
**Beta diversity (Aitchison PC1)**						
All	0.90	0.85	0.96	6.8E-04	7043	1092
Excluding previous fractures	0.91	0.85	0.97	5.8E-03	6433	918
Excluding antibiotic treated	0.89	0.84	0.95	4.1E-04	6768	1046
Excluding fractures within 2 years after baseline	0.91	0.85	0.97	6.6E-03	6793	842
Excluding prevalent inflammatory bowel disease	0.90	0.84	0.95	5.6E-04	6985	1083
Excluding prevalent cancer, CVD, and diabetes	0.89	0.83	0.95	3.0E-04	6343	953
**Proteobacteria**						
All	1.14	1.07	1.20	1.0E-05	7043	1092
Excluding previous fractures	1.13	1.06	1.20	1.5E-04	6433	918
Excluding antibiotic treated	1.12	1.06	1.19	1.2E-04	6768	1046
Excluding fractures within 2 years after baseline	1.13	1.06	1.21	2.4E-04	6793	842
Excluding prevalent inflammatory bowel disease	1.14	1.07	1.20	1.1E-05	6985	1083
Excluding prevalent cancer, CVD, and diabetes	1.13	1.06	1.20	1.1E-04	6343	953
**Tenericutes**						
All	0.90	0.85	0.96	5.4E-04	7043	1092
Excluding previous fractures	0.91	0.86	0.97	5.0E-03	6433	918
Excluding antibiotic treated	0.89	0.84	0.95	2.7E-04	6768	1046
Excluding fractures within 2 years after baseline	0.91	0.86	0.98	9.5E-03	6793	842
Excluding prevalent inflammatory bowel disease	0.90	0.85	0.95	4.2E-04	6985	1083
Excluding prevalent cancer, CVD, and diabetes	0.89	0.83	0.95	3.4E-04	6343	953

Cox regressions with any fractures as outcome, relative abundance of phyla as exposure, and adjusted using the main model (including age, gender, medication, antibiotics, and previous fractures). Diabetes included both type 1 and type 2. CVD, cardiovascular disease; HR, hazard ratio; CI, confidence interval; PC, principal component

Supplementary Table 7. Associations between the relative abundances of different genera belonging to the Proteobacteria phylum and the risk of incident fractures

Predictor	HR	2.5% CI	97.5% CI	P
Gammaproteobacteria	1.06	1.00	1.13	4.1E-02
Betaproteobacteria	1.03	0.97	1.10	2.7E-01
Deltaproteobacteria	1.06	1.00	1.13	4.7E-02
Combined	1.12	1.06	1.19	6.5E-05

Cox regressions with incident fractures as outcome, relative abundance of phyla as exposure, and adjusted using the main model (including age, gender, medication, antibiotics, and previous fractures as covariates). n = 7043 (1092 cases). HR, hazard ratio; CI, confidence interval. Combined = Gammaproteobacteria + Betaproteobacteria + Deltaproteobacteria

Supplementary Table 8. Associations between genus PC's and risk of incident fractures

Predictor	HR	2.5% CI	97.5% CI	P	Variance explained (%)
PC 1	1.11	1.05	1.18	5.2E-04	4.2
PC 2	0.93	0.88	0.98	1.2E-02	2.0
PC 3	0.95	0.89	1.01	7.9E-02	1.5

Cox regressions with incident fractures as outcome, taxa principle componen (PC) as exposure, and adjusted using the main model (including age, gender, medication, antibiotics, and previous fractures as covariates). n = 7043 (1092 cases). HR, hazard ratio; CI, confidence interval

Supplementary Table 9. Associations between the relative abundances of the 25 most abundant genera with risk of incident fractures

Predictor	Mean (SD)	Median (IQR)	HR	2.5% CI	97.5% CI	P	FDR-adjusted P
Parabacteroides	2.1 (2.6)	1.5 (0.6-2.7)	1.12	1.05	1.19	4.6E-04	7.5E-03
Lachnoclostridium	0.7 (1.1)	0.5 (0.4-0.7)	1.11	1.04	1.17	6.0E-04	7.5E-03
Ruminococcus	2.2 (2.0)	1.6 (0.9-2.9)	0.93	0.87	0.98	0.013	0.111
Dorea	2.8 (2.6)	2.1 (1.2-3.6)	0.94	0.88	1.00	0.038	0.235
Bacteroides	32.8 (17.7)	31.5 (19.2-45.1)	1.06	1.00	1.13	0.057	0.235
Escherichia	1.6 (5.6)	0.1 (0.0-0.6)	1.06	1.00	1.12	0.061	0.235
Tyzzerella	0.7 (1.1)	0.5 (0.3-0.8)	1.06	1.00	1.12	0.066	0.235
Barnesiella	1.1 (1.5)	0.4 (0.1-1.6)	1.05	0.99	1.12	0.097	0.302
Blautia	5.1 (4.3)	3.9 (2.5-6.3)	1.04	0.98	1.11	0.192	0.533
Oscillibacter	2.2 (1.6)	1.8 (1.1-2.8)	0.97	0.91	1.03	0.263	0.654
Dialister	0.9 (2.2)	0.1 (0.0-0.9)	1.03	0.97	1.09	0.310	0.654
Ruminiclostridium	1.1 (1.9)	0.4 (0.2-1.0)	0.97	0.91	1.03	0.333	0.654
Collinsella	0.6 (0.9)	0.3 (0.1-0.7)	0.97	0.91	1.03	0.340	0.654
Coprococcus	2.1 (2.1)	1.5 (0.8-2.6)	0.98	0.92	1.04	0.433	0.774
Roseburia	9.4 (7.2)	7.6 (4.3-12.5)	0.98	0.93	1.04	0.552	0.852
Clostridium	1.4 (1.3)	1.0 (0.7-1.6)	1.02	0.96	1.08	0.608	0.852
Prevotella	5.7 (12.7)	0.4 (0.2-1.9)	1.01	0.95	1.08	0.658	0.852
Odoribacter	0.7 (0.6)	0.5 (0.2-0.9)	1.01	0.95	1.08	0.660	0.852
Anaerostipes	1.1 (1.5)	0.6 (0.3-1.3)	0.99	0.93	1.05	0.691	0.852
Butyrivibrio	1.3 (4.1)	0.1 (0.1-0.2)	0.99	0.93	1.05	0.724	0.852
Alistipes	6.6 (5.7)	5.4 (2.7-9.0)	1.01	0.95	1.07	0.729	0.852
Bifidobacterium	2.7 (4.0)	1.3 (0.5-3.2)	1.01	0.95	1.07	0.778	0.852
Eubacterium	2.3 (1.7)	1.9 (1.2-2.9)	0.99	0.93	1.05	0.783	0.852
Akkermansia	1.0 (2.6)	0.1 (0.0-0.8)	1.00	0.95	1.07	0.908	0.946
Faecalibacterium	4.7 (3.1)	4.1 (2.6-6.1)	1.00	0.94	1.06	0.959	0.959

Cox regressions with incident fractures as outcome, relative abundance of the top 25 species as exposure, and adjusted using the main model (including age, gender, medication, antibiotics, and previous fractures). n = 7043 (1092 cases). HR, hazard ratio; CI, confidence interval; FDR, false discovery rate.

Supplementary Table 10. Associations between the relative abundances of the 25 most abundant species with risk of incident fractures

Predictor	Mean (SD)	Median (IQR)	HR	2.5% CI	97.5% CI	P	FDR-adjusted P
Oscillibacter sp. ER4	1.6 (1.5)	1.2 (0.4-2.1)	0.90	0.84	0.95	3.30E-04	4.36E-03
Parabacteroides distasonis	1.4 (2.3)	0.8 (0.2-1.7)	1.12	1.05	1.19	3.49E-04	4.36E-03
Dorea longicatena	1.6 (1.7)	1.1 (0.6-2.1)	0.91	0.86	0.97	2.28E-03	0.019
Bacteroides fragilis	1.4 (3.3)	0.3 (0.1-1.2)	1.08	1.02	1.14	0.013	0.083
Bacteroides cellulosilyticus	1.6 (3.8)	0.2 (0.1-1.3)	1.07	1.01	1.13	0.028	0.141
Roseburia faecis	2.7 (3.5)	1.5 (0.4-3.7)	0.94	0.89	1.00	0.050	0.190
Bacteroides thetaiotaomicron	2.2 (3.3)	1.4 (0.7-2.6)	1.06	1.00	1.13	0.053	0.190
Blautia sp. KLE 1732	1.2 (1.4)	0.7 (0.4-1.4)	0.94	0.89	1.00	0.071	0.223
Escherichia coli	1.6 (5.6)	0.1 (0.0-0.6)	1.05	0.99	1.12	0.105	0.279
Bacteroides uniformis	7.8 (7.8)	5.5 (2.1-11.2)	1.05	0.99	1.12	0.111	0.279
Coprococcus comes	1.5 (1.4)	1.1 (0.6-1.9)	0.96	0.91	1.02	0.228	0.493
Roseburia inulinivorans	3.3 (2.9)	2.5 (1.4-4.3)	0.97	0.91	1.03	0.260	0.493
Anaerostipes hadrus	1.0 (1.4)	0.6 (0.3-1.2)	0.97	0.91	1.03	0.265	0.493
Alistipes finegoldii	1.3 (2.5)	0.5 (0.2-1.4)	1.03	0.97	1.10	0.276	0.493
Bacteroides ovatus	3.1 (4.6)	1.5 (0.7-3.4)	1.03	0.97	1.09	0.400	0.667
Bacteroides plebeius	1.3 (3.4)	0.4 (0.2-0.9)	1.02	0.97	1.09	0.432	0.675
Roseburia intestinalis	2.4 (3.2)	1.2 (0.6-2.8)	0.98	0.92	1.04	0.496	0.711
Bifidobacterium adolescentis	1.5 (2.7)	0.5 (0.1-1.8)	0.98	0.92	1.04	0.561	0.711
Butyrivibrio crossotus	1.5 (4.9)	0.1 (0.1-0.2)	0.98	0.93	1.04	0.576	0.711
Prevotella copri	3.9 (11.3)	0.0 (0.0-0.1)	1.02	0.96	1.08	0.580	0.711
Faecalibacterium prausnitzii	4.5 (3.0)	3.9 (2.5-5.9)	0.98	0.93	1.05	0.597	0.711
Alistipes putredinis	2.8 (2.7)	2.2 (0.4-4.1)	1.01	0.95	1.07	0.709	0.797
Blautia obeum	3.2 (2.8)	2.4 (1.5-4.0)	1.01	0.95	1.07	0.733	0.797
Bacteroides vulgatus	11.1 (9.2)	9.2 (4.1-15.9)	1.01	0.95	1.07	0.786	0.819
Bacteroides stercoris	1.5 (3.3)	0.4 (0.2-1.0)	1.00	0.94	1.06	0.902	0.902

Cox regressions with incident fractures as outcome, relative abundance of the top 25 species as exposure, and adjusted using the main model (including age, gender, medication, antibiotics, and previous fractures). n = 7043 (1092 cases). HR, hazard ratio; CI, confidence interval; FDR, false discovery rate.

Supplementary Table 11. Sensitivity analyses excluding participants with previous fractures, antibiotic treatment, fractures within 2 years after baseline, or inflammatory bowel disease for the top genera and species associations

	HR	2.5% CI	97.5% CI	P	n total	n fractures
Dorea longicatena						
All	0.91	0.86	0.97	2.3E-03	7043	1092
Excluding previous fractures	0.91	0.86	0.97	5.8E-03	6433	918
Excluding antibiotic treated	0.91	0.86	0.97	4.2E-03	6768	1046
Excluding fractures within 2 years after baseline	0.91	0.85	0.98	7.8E-03	6793	842
Excluding prevalent inflammatory bowel disease	0.91	0.85	0.96	1.1E-03	6985	1083
Oscillibacter sp. ER4						
All	0.90	0.84	0.95	3.3E-04	7043	1092
Excluding previous fractures	0.89	0.83	0.95	3.7E-04	6433	918
Excluding antibiotic treated	0.90	0.85	0.96	8.5E-04	6768	1046
Excluding fractures within 2 years after baseline	0.91	0.85	0.97	4.8E-03	6793	842
Excluding prevalent inflammatory bowel disease	0.90	0.85	0.95	4.1E-04	6985	1083
Parabacteroides distasonis						
All	1.12	1.05	1.19	3.5E-04	7043	1092
Excluding previous fractures	1.13	1.05	1.21	4.7E-04	6433	918
Excluding antibiotic treated	1.12	1.05	1.19	5.4E-04	6768	1046
Excluding fractures within 2 years after baseline	1.15	1.07	1.23	1.6E-04	6793	842
Excluding prevalent inflammatory bowel disease	1.12	1.05	1.19	3.4E-04	6985	1083
Lachnoclostridium						
All	1.11	1.04	1.17	6.0E-04	7043	1092
Excluding previous fractures	1.07	1.00	1.14	3.8E-02	6433	918
Excluding antibiotic treated	1.12	1.06	1.19	1.6E-04	6768	1046
Excluding fractures within 2 years after baseline	1.07	1.00	1.14	5.6E-02	6793	842
Excluding prevalent inflammatory bowel disease	1.11	1.04	1.17	6.2E-04	6985	1083
Parabacteroides						
All	1.12	1.05	1.19	4.6E-04	7043	1092
Excluding previous fractures	1.12	1.05	1.20	1.2E-03	6433	918
Excluding antibiotic treated	1.12	1.05	1.19	8.7E-04	6768	1046
Excluding fractures within 2 years after baseline	1.14	1.07	1.23	1.7E-04	6793	842
Excluding prevalent inflammatory bowel disease	1.12	1.05	1.19	4.7E-04	6985	1083

Cox regressions with any fractures as outcome, relative abundance of phyla as exposure, and adjusted using the main model (including age, gender, medication, antibiotics, and previous fractures). HR, hazard ratio; CI, confidence interval; PC, principal component

Supplementary Table 12. Associations between key species and genera with risk of incident fractures and corresponding tests for proportional hazards assumption.

					Proportional hazards assumption	
	**HR**	**2.5% CI**	**97.5% CI**	**P cox**	**P exposure variable**	**P whole model**
Dorea longicatena	0.91	0.86	0.97	2.3E-03	0.098	0.47
Oscillibacter sp. ER4	0.90	0.84	0.95	3.3E-04	0.00014	0.0041
Parabacteroides distasonis	1.12	1.05	1.19	3.5E-04	0.30	0.78
Lachnoclostridium	1.11	1.04	1.17	6.0E-04	0.026	0.20
Parabacteroides	1.12	1.05	1.19	4.6E-04	0.32	0.80

Cox regressions with fractures as outcome, compositional parameters as exposure, and adjusted using the main model (including age, gender, medication, antibiotics, and previous fractures). For tests of proportinal hazards assumption, 0.010 (0.05/5) was considered statistically significant. n = 7043 (1092 cases). HR, hazard ratio; CI, confidence interval

Supplementary Table 13. Associations between gut microbial compositional parameters with risk of incident fractures in women with or without adjustment for menopause status

	HR	2.5% CI	97.5% CI	P	n total	n fractures
**Shannon diversity**						
Without menopause adjustment	0.88	0.82	0.95	9.2E-04	3855	650
With menopause adjustment	0.88	0.82	0.95	1.0E-03	3852	650
**Beta diversity**						
Without menopause adjustment	0.89	0.82	0.97	4.6E-03	3855	650
With menopause adjustment	0.89	0.82	0.97	4.7E-03	3852	650
**Proteobacteria**						
Without menopause adjustment	1.17	1.09	1.26	2.6E-05	3855	650
With menopause adjustment	1.17	1.09	1.26	2.7E-05	3852	650
**Tenericutes**						
Without menopause adjustment	0.89	0.82	0.96	2.3E-03	3855	650
With menopause adjustment	0.89	0.82	0.96	2.2E-03	3852	650

Cox regressions with any fractures as outcome; alpha diversity (Shannon index), the first principal component of Aitchison distance (beta diversity), and the relative abundances of Proteobacteria and Tenericutes as exposure. Only women were included in the analyses that were adjusted using the main model (including age, gender, medication, antibiotics, and previous fractures) with or without additional adjustement for menopause status. HR, hazard ratio; CI, confidence interval

Supplementary Table 14. Associations between gut microbiota compositional parameters and quartiles of serum levels of CRP

Predictor	β	2.5% CI	97.5% CI	P
Shannon diversity	-0.12	-0.15	-0.08	9.4E-11
Aitchison PC1	-0.15	-0.19	-0.12	1.2E-17
Proteobacteria	0.09	0.05	0.12	6.7E-07
Tenericutes	-0.11	-0.14	-0.07	3.2E-09

Linear regressions with serum CRP as outcome, compositional meterices as exposure, and adjusted using the main model (including age, gender, medication, antibiotics, and previous fractures as covariates). n = 6993 (1083 cases). β values are given quartile change of CRP per standard deviation increase in compositional metrices. CI, confidence interval; PC, principal component

Supplementary Table 15. Association between relative abundances of phyla and risk of incident fractures, with or without the adjustment for CRP

Predictor	Model	HR	2.5% CI	97.5% CI	P
Shannon diversity	main model	0.92	0.87	0.98	5.9E-03
	main model + CRP	0.93	0.88	0.99	2.0E-02
Aitchison PC 1	main model	0.90	0.85	0.96	6.8E-04
	main model + CRP	0.91	0.85	0.96	1.9E-03
Proteobacteria	main model	1.14	1.07	1.20	1.0E-05
	main model + CRP	1.13	1.07	1.20	3.5E-05
Tenericutes	main model	0.90	0.85	0.96	5.4E-04
	main model + CRP	0.90	0.85	0.96	9.5E-04

Cox regressions with incident fractures as outcome, relative abundance of phyla as exposure, and adjusted using the main model including age, gender, medication, antibiotics, and previous fractures as covariates (n = 7043 [1092 cases]) or main model plus C-reactive protein (CRP) (n = 6993 [1083 cases]). HR, hazard ratio; CI, confidence interval; PC, principal component

Supplementary Table 16. Associations between functional groups and risk of incident fractures

Cox regressions with any fractures as outcome, functional groups as exposure, and adjusted using the main model (including age, gender, medication, antibiotics, and previous fractures as covariates). n = 7043 (1092 cases). HR, hazard ratio; CI, confidence interval, FDR, false discovery rate

Predictor	Description	HR	2.5% CI	97.5% CI	P	FDR-adjusted P
K05587	bidirectional [NiFe] hydrogenase diaphorase subunit [EC:7.1.1.2]	0.88	0.83	0.93	5.5E-06	8.6E-03
K15518	deoxyguanosine kinase [EC:2.7.1.113]	0.88	0.84	0.93	8.9E-06	8.6E-03
K13853	3-deoxy-7-phosphoheptulonate synthase / chorismate mutase [EC:2.5.1.54 5.4.99.5]	0.88	0.83	0.93	1.2E-05	8.6E-03
K16242	phenol/toluene 2-monooxygenase (NADH) P3/A3 [EC:1.14.13.244 1.14.13.243]	0.89	0.84	0.94	1.9E-05	8.6E-03
K02829	cytochrome aa3-600 menaquinol oxidase subunit IV [EC:7.1.1.5]	0.89	0.84	0.94	2.5E-05	8.6E-03
K01003	oxaloacetate decarboxylase [EC:4.1.1.112]	0.89	0.84	0.94	2.5E-05	8.6E-03
K15629	fatty-acid peroxygenase [EC:1.11.2.4]	0.88	0.83	0.94	3.1E-05	8.6E-03
K00365	urate oxidase [EC:1.7.3.3]	0.89	0.84	0.94	3.2E-05	8.6E-03
K01431	beta-ureidopropionase [EC:3.5.1.6]	0.89	0.84	0.94	3.7E-05	8.6E-03
K05521	ADP-ribosyl-[dinitrogen reductase] hydrolase [EC:3.2.2.24]	0.88	0.83	0.94	3.7E-05	8.6E-03
K11693	peptidoglycan pentaglycine glycine transferase (the first glycine) [EC:2.3.2.16]	0.89	0.84	0.94	3.9E-05	8.6E-03
K07130	arylformamidase [EC:3.5.1.9]	0.88	0.83	0.94	4.1E-05	8.6E-03
K00643	5-aminolevulinate synthase [EC:2.3.1.37]	0.89	0.84	0.94	4.1E-05	8.6E-03
K01166	ribonuclease T2 [EC:4.6.1.19]	0.89	0.84	0.94	4.5E-05	8.6E-03
K13832	3-dehydroquinate dehydratase / shikimate dehydrogenase [EC:4.2.1.10 1.1.1.25]	0.88	0.83	0.94	4.7E-05	8.6E-03
K15761	toluene monooxygenase system protein B [EC:1.14.13.236 1.14.13.-]	0.89	0.84	0.94	5.3E-05	8.6E-03
K05586	bidirectional [NiFe] hydrogenase diaphorase subunit [EC:7.1.1.2]	0.89	0.84	0.94	5.5E-05	8.6E-03
K05588	bidirectional [NiFe] hydrogenase diaphorase subunit [EC:7.1.1.2]	0.89	0.84	0.94	5.5E-05	8.6E-03
K00831	phosphoserine aminotransferase [EC:2.6.1.52]	0.90	0.86	0.95	5.5E-05	8.6E-03
K14048	urease subunit gamma/beta [EC:3.5.1.5]	0.89	0.84	0.94	5.8E-05	8.6E-03
K00588	caffeoyl-CoA O-methyltransferase [EC:2.1.1.104]	0.89	0.84	0.94	6.0E-05	8.6E-03
K15632	23S rRNA (adenine-C8)-methyltransferase [EC:2.1.1.224]	0.89	0.84	0.94	7.3E-05	9.3E-03
K00491	nitric-oxide synthase, bacterial [EC:1.14.14.47]	0.89	0.84	0.94	8.0E-05	9.3E-03
K15534	beta-D-galactosyl-(1->4)-L-rhamnose phosphorylase [EC:2.4.1.247]	0.89	0.84	0.94	8.0E-05	9.3E-03
K01442	choloylglycine hydrolase [EC:3.5.1.24]	0.91	0.87	0.95	8.3E-05	9.3E-03
K03438	16S rRNA (cytosine1402-N4)-methyltransferase [EC:2.1.1.199]	0.90	0.86	0.95	8.5E-05	9.3E-03
K00570	phosphatidylethanolamine/phosphatidyl-N-methylethanolamine N-methyltransferase [EC:2.1.1.17 2.1.1.71]	0.89	0.84	0.94	8.9E-05	9.3E-03
K09699	2-oxoisovalerate dehydrogenase E2 component (dihydrolipoyl transacylase) [EC:2.3.1.168]	0.89	0.84	0.94	9.1E-05	9.3E-03
K04766	acetoin utilization protein AcuA [EC:2.3.1.-]	0.89	0.84	0.94	9.7E-05	9.3E-03
K11211	3-deoxy-D-manno-octulosonic acid kinase [EC:2.7.1.166]	0.89	0.84	0.94	9.9E-05	9.3E-03
K00577	tetrahydromethanopterin S-methyltransferase subunit A [EC:2.1.1.86]	0.89	0.83	0.94	1.0E-04	9.3E-03
K00066	GDP-mannose 6-dehydrogenase [EC:1.1.1.132]	0.89	0.84	0.94	1.0E-04	9.3E-03
K03280	UDP-N-acetylglucosamine:(glucosyl)LPS alpha-1,2-N-acetylglucosaminyltransferase [EC:2.4.1.56]	1.12	1.06	1.19	1.1E-04	9.3E-03
K01114	phospholipase C [EC:3.1.4.3]	0.89	0.84	0.94	1.1E-04	9.3E-03
K09883	cobaltochelatase CobT [EC:6.6.1.2]	0.89	0.84	0.95	1.1E-04	9.3E-03
K00100	butanol dehydrogenase [EC:1.1.1.-]	0.89	0.84	0.95	1.2E-04	9.3E-03
K01303	acylaminoacyl-peptidase [EC:3.4.19.1]	0.89	0.84	0.94	1.2E-04	9.3E-03
K01469	5-oxoprolinase (ATP-hydrolysing) [EC:3.5.2.9]	0.89	0.84	0.95	1.2E-04	9.3E-03
K06178	23S rRNA pseudouridine2605 synthase [EC:5.4.99.22]	0.91	0.87	0.95	1.2E-04	9.3E-03
K00015	glyoxylate reductase [EC:1.1.1.26]	0.89	0.84	0.95	1.2E-04	9.3E-03

Predictor	Description	HR	2.5% CI	97.5% CI	P	FDR-adjusted P
K14980	two-component system, OmpR family, sensor histidine kinase ChvG [EC:2.7.13.3]	0.89	0.84	0.95	1.3E-04	9.3E-03
K11532	fructose-1,6-bisphosphatase II / sedoheptulose-1,7-bisphosphatase [EC:3.1.3.11 3.1.3.37]	0.90	0.85	0.95	1.3E-04	9.4E-03
K16146	maltokinase [EC:2.7.1.175]	0.90	0.85	0.95	1.4E-04	9.7E-03
K14660	nodulation protein E [EC:2.3.1.-]	0.90	0.85	0.95	1.4E-04	9.7E-03
K00531	nitrogenase delta subunit [EC:1.18.6.1]	0.89	0.84	0.95	1.5E-04	9.7E-03
K01283	peptidyl-dipeptidase A [EC:3.4.15.1]	0.89	0.84	0.95	1.5E-04	9.7E-03
K13607	cinnamoyl-CoA:phenyllactate CoA-transferase [EC:2.8.3.17]	0.90	0.85	0.95	1.6E-04	1.0E-02
K13004	galacturonosyltransferase WbtD [EC:2.4.1.-]	0.89	0.84	0.95	1.6E-04	1.0E-02
K08081	tropinone reductase I [EC:1.1.1.206]	0.90	0.85	0.95	1.7E-04	1.0E-02
K00514	zeta-carotene desaturase [EC:1.3.5.6]	0.90	0.85	0.95	1.7E-04	1.0E-02
K01586	diaminopimelate decarboxylase [EC:4.1.1.20]	0.90	0.86	0.95	1.7E-04	1.0E-02
K16173	glutaryl-CoA dehydrogenase (non-decarboxylating) [EC:1.3.99.32]	0.90	0.85	0.95	1.9E-04	1.0E-02
K03786	3-dehydroquinate dehydratase II [EC:4.2.1.10]	0.92	0.87	0.96	1.9E-04	1.0E-02
K05951	NAD+---dinitrogen-reductase ADP-D-ribosyltransferase [EC:2.4.2.37]	0.90	0.85	0.95	1.9E-04	1.0E-02
K15894	UDP-N-acetylglucosamine 4,6-dehydratase/5-epimerase [EC:4.2.1.115 5.1.3.-]	0.90	0.85	0.95	1.9E-04	1.0E-02
K00469	inositol oxygenase [EC:1.13.99.1]	0.90	0.85	0.95	1.9E-04	1.0E-02
K02277	cytochrome c oxidase subunit IV [EC:7.1.1.9]	0.89	0.84	0.95	2.0E-04	1.0E-02
K15764	toluene monooxygenase system protein E [EC:1.14.13.236 1.14.13.-]	0.90	0.85	0.95	2.0E-04	1.0E-02
K00600	glycine hydroxymethyltransferase [EC:2.1.2.1]	0.91	0.87	0.96	2.0E-04	1.0E-02
K12526	bifunctional diaminopimelate decarboxylase / aspartate kinase [EC:4.1.1.20 2.7.2.4]	0.90	0.85	0.95	2.0E-04	1.0E-02
K00166	2-oxoisovalerate dehydrogenase E1 component alpha subunit [EC:1.2.4.4]	0.89	0.84	0.95	2.0E-04	1.0E-02
K10960	geranylgeranyl diphosphate/geranylgeranyl-bacteriochlorophyllide a reductase [EC:1.3.1.83 1.3.1.111]	0.90	0.85	0.95	2.1E-04	1.0E-02
K06012	spore protease [EC:3.4.24.78]	0.89	0.84	0.95	2.2E-04	1.0E-02
K13015	UDP-N-acetyl-D-glucosamine dehydrogenase [EC:1.1.1.136]	0.89	0.84	0.95	2.2E-04	1.0E-02
K00319	methylenetetrahydromethanopterin dehydrogenase [EC:1.5.98.1]	0.89	0.84	0.95	2.3E-04	1.0E-02
K01569	oxalate decarboxylase [EC:4.1.1.2]	0.90	0.85	0.95	2.4E-04	1.1E-02
K09065	N-acetylornithine carbamoyltransferase [EC:2.1.3.9]	0.90	0.85	0.95	2.4E-04	1.1E-02
K00167	2-oxoisovalerate dehydrogenase E1 component beta subunit [EC:1.2.4.4]	0.90	0.84	0.95	2.5E-04	1.1E-02
K11614	two-component system, CitB family, sensor histidine kinase MalK [EC:2.7.13.3]	0.89	0.84	0.95	2.6E-04	1.1E-02
K01617	2-oxo-3-hexenedioate decarboxylase [EC:4.1.1.77]	0.90	0.85	0.95	2.6E-04	1.1E-02
K00803	alkyldihydroxyacetonephosphate synthase [EC:2.5.1.26]	0.89	0.84	0.95	2.6E-04	1.1E-02
K00274	monoamine oxidase [EC:1.4.3.4]	0.89	0.84	0.95	2.6E-04	1.1E-02
K13598	two-component system, NtrC family, nitrogen regulation sensor histidine kinase NtrY [EC:2.7.13.3]	0.90	0.84	0.95	2.8E-04	1.1E-02
K14748	ethylbenzene dioxygenase subunit alpha [EC:1.14.12.-]	0.90	0.85	0.95	2.8E-04	1.1E-02
K14749	ethylbenzene dioxygenase subunit beta [EC:1.14.12.-]	0.90	0.85	0.95	2.8E-04	1.1E-02
K00253	isovaleryl-CoA dehydrogenase [EC:1.3.8.4]	0.90	0.84	0.95	2.8E-04	1.1E-02
K15733	dye decolorizing peroxidase [EC:1.11.1.19]	0.90	0.85	0.95	2.9E-04	1.1E-02
K01046	triacylglycerol lipase [EC:3.1.1.3]	0.90	0.84	0.95	3.0E-04	1.1E-02
K07717	two-component system, sensor histidine kinase GlnK [EC:2.7.13.3]	0.90	0.84	0.95	3.0E-04	1.1E-02
K01078	acid phosphatase [EC:3.1.3.2]	0.90	0.85	0.95	3.0E-04	1.1E-02
K16648	arabinofuranan 3-O-arabinosyltransferase [EC:2.4.2.-]	0.90	0.85	0.95	3.1E-04	1.1E-02
K00102	D-lactate dehydrogenase (cytochrome) [EC:1.1.2.4]	0.90	0.84	0.95	3.1E-04	1.1E-02
K03405	magnesium chelatase subunit I [EC:6.6.1.1]	0.89	0.84	0.95	3.2E-04	1.1E-02
K01800	maleylacetoacetate isomerase [EC:5.2.1.2]	0.90	0.85	0.95	3.3E-04	1.2E-02

Predictor	Description	HR	2.5% CI	97.5% CI	P	FDR-adjusted P
K15067	2-aminomuconate deaminase [EC:3.5.99.5]	0.90	0.85	0.95	3.4E-04	1.2E-02
K06208	chorismate mutase [EC:5.4.99.5]	0.90	0.84	0.95	3.4E-04	1.2E-02
K05922	quinone-reactive Ni/Fe-hydrogenase large subunit [EC:1.12.5.1]	0.90	0.85	0.95	3.4E-04	1.2E-02
K00544	betaine-homocysteine S-methyltransferase [EC:2.1.1.5]	0.90	0.84	0.95	3.5E-04	1.2E-02
K14654	2,5-diamino-6-(ribosylamino)-4(3H)-pyrimidinone 5'-phosphate reductase [EC:1.1.1.302]	0.90	0.84	0.95	3.6E-04	1.2E-02
K02827	cytochrome aa3-600 menaquinol oxidase subunit I [EC:7.1.1.5]	0.90	0.85	0.96	3.6E-04	1.2E-02
K00197	acetyl-CoA decarbonylase/synthase, CODH/ACS complex subunit gamma [EC:2.1.1.245]	0.90	0.84	0.95	3.7E-04	1.2E-02
K00263	leucine dehydrogenase [EC:1.4.1.9]	0.90	0.85	0.95	3.7E-04	1.2E-02
K00619	amino-acid N-acetyltransferase [EC:2.3.1.1]	0.90	0.85	0.95	3.7E-04	1.2E-02
K00148	glutathione-independent formaldehyde dehydrogenase [EC:1.2.1.46]	0.90	0.85	0.95	3.8E-04	1.2E-02
K11357	two-component system, cell cycle sensor histidine kinase DivJ [EC:2.7.13.3]	0.90	0.85	0.95	3.8E-04	1.2E-02
K00533	ferredoxin hydrogenase large subunit [EC:1.12.7.2]	0.90	0.85	0.95	3.9E-04	1.2E-02
K10977	methanogen homocitrate synthase [EC:2.3.3.14 2.3.3.-]	0.90	0.84	0.95	4.0E-04	1.2E-02
K01518	bis(5'-nucleosidyl)-tetraphosphatase [EC:3.6.1.17]	1.10	1.04	1.15	4.0E-04	1.2E-02
K00500	phenylalanine-4-hydroxylase [EC:1.14.16.1]	0.90	0.85	0.95	4.0E-04	1.2E-02
K07716	two-component system, cell cycle sensor histidine kinase PleC [EC:2.7.13.3]	0.90	0.85	0.96	4.0E-04	1.2E-02
K00220	cyclohexadieny/prephenate dehydrogenase [EC:1.3.1.43 1.3.1.12]	0.90	0.85	0.96	4.0E-04	1.2E-02
K02591	nitrogenase molybdenum-iron protein beta chain [EC:1.18.6.1]	0.90	0.85	0.95	4.1E-04	1.2E-02
K04101	protocatechuate 4,5-dioxygenase, beta chain [EC:1.13.11.8]	0.90	0.85	0.95	4.1E-04	1.2E-02
K00686	protein-glutamine gamma-glutamyltransferase [EC:2.3.2.13]	0.90	0.85	0.95	4.1E-04	1.2E-02
K07094	heptaprenylglyceryl phosphate synthase [EC:2.5.1.-]	0.90	0.85	0.95	4.2E-04	1.2E-02
K10978	methanogen homoisocitrate dehydrogenase [EC:1.1.1.87 1.1.1.-]	0.90	0.84	0.95	4.2E-04	1.2E-02
K01820	L-rhamnose isomerase / sugar isomerase [EC:5.3.1.14 5.3.1.-]	0.90	0.85	0.95	4.2E-04	1.2E-02
K10843	DNA excision repair protein ERCC-3 [EC:3.6.4.12]	0.90	0.85	0.95	4.2E-04	1.2E-02
K13282	cyanophycinase [EC:3.4.15.6]	0.90	0.85	0.95	4.3E-04	1.2E-02
K11337	bacteriochlorophyllide a dehydrogenase [EC:1.1.1.396]	0.90	0.85	0.96	4.3E-04	1.2E-02
K00456	cysteine dioxygenase [EC:1.13.11.20]	0.90	0.86	0.96	4.5E-04	1.2E-02
K01689	enolase [EC:4.2.1.11]	0.91	0.87	0.96	4.5E-04	1.2E-02
K00326	cytochrome-b5 reductase [EC:1.6.2.2]	1.10	1.04	1.15	4.5E-04	1.2E-02
K05929	phosphoethanolamine N-methyltransferase [EC:2.1.1.103]	1.10	1.04	1.15	4.5E-04	1.2E-02
K00579	tetrahydromethanopterin S-methyltransferase subunit C [EC:2.1.1.86]	0.90	0.84	0.95	4.6E-04	1.2E-02
K00451	homogentisate 1,2-dioxygenase [EC:1.13.11.5]	0.90	0.85	0.95	4.6E-04	1.2E-02
K04127	isopenicillin-N epimerase [EC:5.1.1.17]	0.90	0.85	0.96	4.6E-04	1.2E-02
K00452	3-hydroxyanthranilate 3,4-dioxygenase [EC:1.13.11.6]	0.90	0.85	0.95	4.7E-04	1.2E-02
K00980	glycerol-3-phosphate cytidylyltransferase [EC:2.7.7.39]	0.90	0.85	0.95	4.8E-04	1.2E-02
K00194	acetyl-CoA decarbonylase/synthase, CODH/ACS complex subunit delta [EC:2.1.1.245]	0.90	0.84	0.95	4.8E-04	1.2E-02
K07653	two-component system, OmpR family, sensor histidine kinase MprB [EC:2.7.13.3]	0.90	0.85	0.96	4.9E-04	1.2E-02
K03045	DNA-directed RNA polymerase subunit B\ [EC:2.7.7.6]	0.90	0.85	0.95	4.9E-04	1.2E-02
K01457	allophanate hydrolase [EC:3.5.1.54]	0.90	0.85	0.95	4.9E-04	1.2E-02
K00578	tetrahydromethanopterin S-methyltransferase subunit B [EC:2.1.1.86]	0.90	0.85	0.95	5.0E-04	1.2E-02
K01127	glycosylphosphatidylinositol phospholipase D [EC:3.1.4.50]	1.09	1.04	1.15	5.0E-04	1.2E-02
K12508	feruloyl-CoA synthase [EC:6.2.1.34]	0.90	0.85	0.96	5.1E-04	1.2E-02
K14136	decaprenyl-phosphate phosphoribosyltransferase [EC:2.4.2.45]	0.90	0.86	0.96	5.1E-04	1.2E-02
K01965	propionyl-CoA carboxylase alpha chain [EC:6.4.1.3]	0.90	0.85	0.96	5.2E-04	1.2E-02

Predictor	Description	HR	2.5% CI	97.5% CI	P	FDR-adjusted P
K09844	carotenoid 1,2-hydratase [EC:4.2.1.131]	0.90	0.85	0.96	5.2E-04	1.2E-02
K00203	formylmethanofuran dehydrogenase subunit D [EC:1.2.7.12]	0.90	0.85	0.96	5.3E-04	1.2E-02
K01561	haloacetate dehalogenase [EC:3.8.1.3]	0.90	0.85	0.96	5.3E-04	1.2E-02
K01623	fructose-bisphosphate aldolase, class I [EC:4.1.2.13]	0.90	0.85	0.96	5.4E-04	1.2E-02
K14986	two-component system, LuxR family, sensor kinase FixL [EC:2.7.13.3]	0.91	0.86	0.96	5.4E-04	1.2E-02
K02586	nitrogenase molybdenum-iron protein alpha chain [EC:1.18.6.1]	0.90	0.85	0.96	5.4E-04	1.2E-02
K00399	methyl-coenzyme M reductase alpha subunit [EC:2.8.4.1]	0.90	0.85	0.95	5.5E-04	1.2E-02
K12454	CDP-paratose 2-epimerase [EC:5.1.3.10]	0.90	0.85	0.96	5.5E-04	1.2E-02
K14127	F420-non-reducing hydrogenase iron-sulfur subunit [EC:1.12.99.- 1.8.98.5 1.8.98.6]	0.90	0.85	0.96	5.7E-04	1.2E-02
K04040	chlorophyll/bacteriochlorophyll a synthase [EC:2.5.1.62 2.5.1.133]	0.91	0.86	0.96	5.7E-04	1.2E-02
K00401	methyl-coenzyme M reductase beta subunit [EC:2.8.4.1]	0.90	0.85	0.96	5.7E-04	1.2E-02
K05299	formate dehydrogenase (NADP+) alpha subunit [EC:1.17.1.10]	0.90	0.85	0.96	5.7E-04	1.2E-02
K01834	2,3-bisphosphoglycerate-dependent phosphoglycerate mutase [EC:5.4.2.11]	0.91	0.86	0.96	5.8E-04	1.2E-02
K02803	N-acetylglucosamine PTS system EIIB component [EC:2.7.1.193]	0.91	0.86	0.96	5.9E-04	1.2E-02
K01968	3-methylcrotonyl-CoA carboxylase alpha subunit [EC:6.4.1.4]	0.90	0.85	0.96	5.9E-04	1.2E-02
K01476	arginase [EC:3.5.3.1]	0.90	0.85	0.96	6.0E-04	1.2E-02
K00947	molybdenum storage protein	0.90	0.85	0.96	6.0E-04	1.2E-02
K07651	two-component system, OmpR family, sensor histidine kinase ResE [EC:2.7.13.3]	0.90	0.85	0.96	6.0E-04	1.2E-02
K11691	two-component system, CitB family, sensor histidine kinase DctS [EC:2.7.13.3]	0.91	0.86	0.96	6.1E-04	1.2E-02
K13503	anthranilate synthase [EC:4.1.3.27]	0.90	0.85	0.96	6.1E-04	1.2E-02
K02316	DNA primase [EC:2.7.7.101]	0.92	0.87	0.96	6.1E-04	1.2E-02
K00532	ferredoxin hydrogenase [EC:1.12.7.2]	0.90	0.85	0.96	6.2E-04	1.2E-02
K00356	NA	0.90	0.85	0.96	6.2E-04	1.2E-02
K09835	prolycopene isomerase [EC:5.2.1.13]	0.91	0.85	0.96	6.2E-04	1.2E-02
K02274	cytochrome c oxidase subunit I [EC:7.1.1.9]	0.90	0.85	0.96	6.2E-04	1.2E-02
K14259	2-dehydro-3-deoxy-D-arabinonate dehydratase [EC:4.2.1.141]	0.90	0.85	0.96	6.2E-04	1.2E-02
K04343	streptomycin 6-kinase [EC:2.7.1.72]	0.90	0.85	0.96	6.3E-04	1.2E-02
K13541	cobalt-precorrin 5A hydrolase / cobalt-factor III methyltransferase / precorrin-3B C17-methyltransferase [EC:3.7.1.12 2.1.1.272 2.1.1.131]	0.91	0.85	0.96	6.3E-04	1.2E-02
K16149	1,4-alpha-glucan branching enzyme [EC:2.4.1.18]	0.90	0.85	0.96	6.4E-04	1.2E-02
K00354	NADPH2 dehydrogenase [EC:1.6.99.1]	0.90	0.85	0.96	6.5E-04	1.2E-02
K00091	dihydroflavonol-4-reductase [EC:1.1.1.219]	0.90	0.85	0.96	6.5E-04	1.2E-02
K02828	cytochrome aa3-600 menaquinol oxidase subunit III [EC:7.1.1.5]	0.91	0.86	0.96	6.5E-04	1.2E-02
K03044	DNA-directed RNA polymerase subunit B' [EC:2.7.7.6]	0.90	0.85	0.96	6.5E-04	1.2E-02
K00582	tetrahydromethanopterin S-methyltransferase subunit F [EC:2.1.1.86]	0.90	0.85	0.96	6.6E-04	1.2E-02
K14338	cytochrome P450 / NADPH-cytochrome P450 reductase [EC:1.14.14.1 1.6.2.4]	0.91	0.86	0.96	6.6E-04	1.2E-02
K02482	two-component system, NtrC family, sensor kinase [EC:2.7.13.3]	0.90	0.85	0.96	6.8E-04	1.2E-02
K00008	L-iditol 2-dehydrogenase [EC:1.1.1.14]	0.90	0.85	0.96	6.8E-04	1.2E-02
K02228	precorrin-6A synthase [EC:2.1.1.152]	0.90	0.85	0.96	6.8E-04	1.2E-02
K01941	urea carboxylase [EC:6.3.4.6]	0.90	0.85	0.96	6.8E-04	1.2E-02
K01746	formiminotetrahydrofolate cyclodeaminase [EC:4.3.1.4]	0.90	0.85	0.96	6.9E-04	1.2E-02
K14138	acetyl-CoA synthase [EC:2.3.1.169]	0.90	0.85	0.96	6.9E-04	1.2E-02
K15781	putative phosphoserine phosphatase / 1-acylglycerol-3-phosphate O-acyltransferase [EC:3.1.3.3 2.3.1.51]	0.91	0.86	0.96	6.9E-04	1.2E-02
K00303	sarcosine oxidase, subunit beta [EC:1.5.3.1]	0.90	0.85	0.96	7.0E-04	1.2E-02
K04037	light-independent protochlorophyllide reductase subunit L [EC:1.3.7.7]	0.90	0.85	0.96	7.0E-04	1.2E-02

Predictor	Description	HR	2.5% CI	97.5% CI	P	FDR-adjusted P
K15921	arabinoxylan arabinofuranohydrolase [EC:3.2.1.55]	0.90	0.85	0.96	7.0E-04	1.2E-02
K00720	ceramide glucosyltransferase [EC:2.4.1.80]	0.90	0.85	0.96	7.1E-04	1.2E-02
K01400	bacillolysin [EC:3.4.24.28]	0.91	0.86	0.96	7.1E-04	1.2E-02
K11783	futalosine hydrolase [EC:3.2.2.26]	0.90	0.85	0.96	7.1E-04	1.2E-02
K15372	taurine---2-oxoglutarate transaminase [EC:2.6.1.55]	0.90	0.85	0.96	7.1E-04	1.2E-02
K00122	formate dehydrogenase [EC:1.17.1.9]	0.90	0.85	0.96	7.2E-04	1.2E-02
K00392	sulfite reductase (ferredoxin) [EC:1.8.7.1]	0.90	0.85	0.96	7.2E-04	1.2E-02
K00808	homospermidine synthase [EC:2.5.1.44]	0.91	0.86	0.96	7.2E-04	1.2E-02
K00038	3alpha(or 20beta)-hydroxysteroid dehydrogenase [EC:1.1.1.53]	0.91	0.86	0.96	7.3E-04	1.2E-02
K07446	tRNA (guanine10-N2)-dimethyltransferase [EC:2.1.1.213]	0.90	0.85	0.96	7.4E-04	1.2E-02
K00472	prolyl 4-hydroxylase [EC:1.14.11.2]	0.91	0.86	0.96	7.5E-04	1.2E-02
K15519	deoxyadenosine/deoxycytidine kinase [EC:2.7.1.76 2.7.1.74]	0.91	0.86	0.96	7.5E-04	1.2E-02
K01444	N4-(beta-N-acetylglucosaminyl)-L-asparaginase [EC:3.5.1.26]	0.91	0.85	0.96	7.5E-04	1.2E-02
K02683	DNA primase small subunit [EC:2.7.7.102]	0.90	0.85	0.96	7.6E-04	1.2E-02
K00958	sulfate adenylyltransferase [EC:2.7.7.4]	0.90	0.85	0.96	7.6E-04	1.2E-02
K10217	aminomuconate-semialdehyde/2-hydroxymuconate-6-semialdehyde dehydrogenase [EC:1.2.1.32 1.2.1.85]	0.91	0.85	0.96	7.7E-04	1.2E-02
K15924	glucuronoarabinoxylan endo-1,4-beta-xylanase [EC:3.2.1.136]	0.90	0.85	0.96	7.7E-04	1.2E-02
K04117	cyclohexanecarboxyl-CoA dehydrogenase [EC:1.3.99.-]	0.91	0.86	0.96	7.7E-04	1.2E-02
K00486	kynurenine 3-monooxygenase [EC:1.14.13.9]	0.90	0.85	0.96	7.8E-04	1.2E-02
K04719	5,6-dimethylbenzimidazole synthase [EC:1.13.11.79]	0.90	0.85	0.96	7.8E-04	1.2E-02
K03519	aerobic carbon-monoxide dehydrogenase medium subunit [EC:1.2.5.3]	0.90	0.85	0.96	7.8E-04	1.2E-02
K12256	putrescine---pyruvate transaminase [EC:2.6.1.113]	0.90	0.85	0.96	7.9E-04	1.2E-02
K00141	benzaldehyde dehydrogenase (NAD) [EC:1.2.1.28]	0.91	0.85	0.96	7.9E-04	1.2E-02
K11629	two-component system, OmpR family, bacitracin resistance sensor histidine kinase BceS [EC:2.7.13.3]	0.91	0.86	0.96	7.9E-04	1.2E-02
K07508	acetyl-CoA acyltransferase 2 [EC:2.3.1.16]	0.91	0.86	0.96	7.9E-04	1.2E-02
K15513	benzoyl-CoA-dihydrodiol lyase [EC:4.1.2.44]	0.91	0.86	0.96	7.9E-04	1.2E-02
K00232	acyl-CoA oxidase [EC:1.3.3.6]	0.91	0.85	0.96	8.0E-04	1.2E-02
K02594	homocitrate synthase NifV [EC:2.3.3.14]	0.90	0.85	0.96	8.0E-04	1.2E-02
K07539	6-oxocyclohex-1-ene-carbonyl-CoA hydrolase [EC:3.7.1.21]	0.91	0.86	0.96	8.0E-04	1.2E-02
K00580	tetrahydromethanopterin S-methyltransferase subunit D [EC:2.1.1.86]	0.90	0.85	0.96	8.0E-04	1.2E-02
K14162	error-prone DNA polymerase [EC:2.7.7.7]	0.90	0.85	0.96	8.1E-04	1.2E-02
K14268	5-aminovalerate/4-aminobutyrate aminotransferase [EC:2.6.1.48 2.6.1.19]	0.91	0.86	0.96	8.2E-04	1.2E-02
K00702	cellobiose phosphorylase [EC:2.4.1.20]	0.90	0.85	0.96	8.2E-04	1.2E-02
K11780	7,8-didemethyl-8-hydroxy-5-deazariboflavin synthase [EC:4.3.1.32]	0.91	0.86	0.96	8.2E-04	1.2E-02
K00954	pantetheine-phosphate adenylyltransferase [EC:2.7.7.3]	0.92	0.87	0.96	8.2E-04	1.2E-02
K15864	nitrite reductase (NO-forming) / hydroxylamine reductase [EC:1.7.2.1 1.7.99.1]	0.91	0.86	0.96	8.2E-04	1.2E-02
K06118	UDP-sulfoquinovose synthase [EC:3.13.1.1]	0.91	0.86	0.96	8.2E-04	1.2E-02
K01724	4a-hydroxytetrahydrobiopterin dehydratase [EC:4.2.1.96]	0.90	0.85	0.96	8.3E-04	1.2E-02
K15526	L-cysteine:1D-myo-inositol 2-amino-2-deoxy-alpha-D-glucopyranoside ligase [EC:6.3.1.13]	0.90	0.85	0.96	8.3E-04	1.2E-02
K11210	metallothiol transferase [EC:2.5.1.-]	0.91	0.86	0.96	8.4E-04	1.2E-02
K09809	CDP-glycerol glycerophosphotransferase [EC:2.7.8.12]	0.90	0.85	0.96	8.5E-04	1.2E-02
K00202	formylmethanofuran dehydrogenase subunit C [EC:1.2.7.12]	0.90	0.85	0.96	8.6E-04	1.2E-02
K00311	electron-transferring-flavoprotein dehydrogenase [EC:1.5.5.1]	0.90	0.85	0.96	8.7E-04	1.2E-02
K07558	tRNA nucleotidyltransferase (CCA-adding enzyme) [EC:2.7.7.72]	0.90	0.85	0.96	8.8E-04	1.2E-02

Predictor	Description	HR	2.5% CI	97.5% CI	P	FDR-adjusted P
K06134	3-demethoxyubiquinol 3-hydroxylase [EC:1.14.99.60]	0.90	0.85	0.96	8.8E-04	1.2E-02
K12503	short-chain Z-isoprenyl diphosphate synthase [EC:2.5.1.68]	0.91	0.85	0.96	8.8E-04	1.2E-02
K03051	DNA-directed RNA polymerase subunit F [EC:2.7.7.6]	0.90	0.85	0.96	8.9E-04	1.2E-02
K00402	methyl-coenzyme M reductase gamma subunit [EC:2.8.4.1]	0.90	0.85	0.96	8.9E-04	1.2E-02
K15371	glutamate dehydrogenase [EC:1.4.1.2]	0.90	0.85	0.96	9.0E-04	1.2E-02
K00019	3-hydroxybutyrate dehydrogenase [EC:1.1.1.30]	0.90	0.85	0.96	9.0E-04	1.2E-02
K11782	chorismate dehydratase [EC:4.2.1.151]	0.90	0.85	0.96	9.0E-04	1.2E-02
K05988	dextranase [EC:3.2.1.11]	0.91	0.86	0.96	9.1E-04	1.2E-02
K15408	cytochrome c oxidase subunit I+III [EC:7.1.1.9]	0.91	0.86	0.96	9.1E-04	1.2E-02
K10780	enoyl-[acyl-carrier protein] reductase III [EC:1.3.1.104]	0.91	0.86	0.96	9.1E-04	1.2E-02
K13075	N-acyl homoserine lactone hydrolase [EC:3.1.1.81]	0.91	0.86	0.96	9.1E-04	1.2E-02
K13485	2-oxo-4-hydroxy-4-carboxy-5-ureidoimidazoline decarboxylase [EC:4.1.1.97]	0.91	0.86	0.96	9.2E-04	1.2E-02
K06399	stage IV sporulation protein B [EC:3.4.21.116]	0.90	0.85	0.96	9.2E-04	1.2E-02
K00318	proline dehydrogenase [EC:1.5.5.2]	0.90	0.85	0.96	9.2E-04	1.2E-02
K07031	D-glycero-alpha-D-manno-heptose-7-phosphate kinase [EC:2.7.1.168]	0.90	0.85	0.96	9.3E-04	1.2E-02
K02275	cytochrome c oxidase subunit II [EC:7.1.1.9]	0.90	0.85	0.96	9.3E-04	1.2E-02
K02276	cytochrome c oxidase subunit III [EC:7.1.1.9]	0.90	0.85	0.96	9.3E-04	1.2E-02
K06382	stage II sporulation protein E [EC:3.1.3.16]	0.90	0.85	0.96	9.4E-04	1.2E-02
K05927	quinone-reactive Ni/Fe-hydrogenase small subunit [EC:1.12.5.1]	0.91	0.86	0.96	9.4E-04	1.2E-02
K00933	creatine kinase [EC:2.7.3.2]	0.91	0.86	0.96	9.4E-04	1.2E-02
K00273	D-amino-acid oxidase [EC:1.4.3.3]	0.91	0.86	0.96	9.4E-04	1.2E-02
K03821	poly[(R)-3-hydroxyalkanoate] polymerase subunit PhaC [EC:2.3.1.304]	0.91	0.85	0.96	9.5E-04	1.2E-02
K07469	aldehyde oxidoreductase [EC:1.2.99.7]	0.90	0.85	0.96	9.5E-04	1.2E-02
K06982	pantoate kinase [EC:2.7.1.169]	0.90	0.85	0.96	9.5E-04	1.2E-02
K03392	aminocarboxymuconate-semialdehyde decarboxylase [EC:4.1.1.45]	0.91	0.86	0.96	9.6E-04	1.2E-02
K16216	benzil reductase ((S)-benzoin forming) [EC:1.1.1.320]	0.91	0.85	0.96	9.6E-04	1.2E-02
K01969	3-methylcrotonyl-CoA carboxylase beta subunit [EC:6.4.1.4]	0.91	0.85	0.96	9.6E-04	1.2E-02
K03050	DNA-directed RNA polymerase subunit E\ [EC:2.7.7.6]	0.90	0.85	0.96	9.7E-04	1.2E-02
K00507	stearoyl-CoA desaturase (Delta-9 desaturase) [EC:1.14.19.1]	0.90	0.85	0.96	9.7E-04	1.2E-02
K00249	acyl-CoA dehydrogenase [EC:1.3.8.7]	0.91	0.85	0.96	9.7E-04	1.2E-02
K01924	UDP-N-acetylmuramate--alanine ligase [EC:6.3.2.8]	0.92	0.87	0.97	9.8E-04	1.2E-02
K01266	D-aminopeptidase [EC:3.4.11.19]	0.91	0.85	0.96	9.8E-04	1.2E-02
K10855	acetone carboxylase, beta subunit [EC:6.4.1.6]	0.91	0.86	0.96	9.8E-04	1.2E-02
K01555	fumarylacetoacetase [EC:3.7.1.2]	0.91	0.85	0.96	9.9E-04	1.2E-02
K06034	sulfopyruvate decarboxylase subunit alpha [EC:4.1.1.79]	0.90	0.85	0.96	9.9E-04	1.2E-02
K06379	stage II sporulation protein AB (anti-sigma F factor) [EC:2.7.11.1]	0.90	0.85	0.96	9.9E-04	1.2E-02
K14083	trimethylamine---corrinoid protein Co-methyltransferase [EC:2.1.1.250]	0.91	0.85	0.96	1.0E-03	1.2E-02
K01732	pectin lyase [EC:4.2.2.10]	0.90	0.85	0.96	1.0E-03	1.2E-02
K01178	glucoamylase [EC:3.2.1.3]	0.91	0.85	0.96	1.0E-03	1.2E-02
K00153	S-(hydroxymethyl)mycothiol dehydrogenase [EC:1.1.1.306]	0.91	0.85	0.96	1.0E-03	1.2E-02
K14128	F420-non-reducing hydrogenase small subunit [EC:1.12.99.- 1.8.98.5]	0.90	0.85	0.96	1.0E-03	1.2E-02
K16306	fructose-bisphosphate aldolase / 2-amino-3,7-dideoxy-D-threo-hept-6-ulosonate synthase [EC:4.1.2.13 2.2.1.10]	0.91	0.85	0.96	1.0E-03	1.2E-02
K02319	DNA polymerase, archaea type [EC:2.7.7.7]	0.90	0.85	0.96	1.0E-03	1.2E-02
K13533	two-component system, sporulation sensor kinase E [EC:2.7.13.3]	0.91	0.86	0.96	1.0E-03	1.2E-02

Predictor	Description	HR	2.5% CI	97.5% CI	P	FDR-adjusted P
K01301	N-acetylated-alpha-linked acidic dipeptidase [EC:3.4.17.21]	0.91	0.86	0.96	1.0E-03	1.2E-02
K13531	methylated-DNA-[protein]-cysteine S-methyltransferase [EC:2.1.1.63]	0.91	0.86	0.96	1.0E-03	1.2E-02
K07516	3-hydroxyacyl-CoA dehydrogenase [EC:1.1.1.35]	0.91	0.85	0.96	1.0E-03	1.2E-02
K00663	aminoglycoside 6'-N-acetyltransferase [EC:2.3.1.82]	0.91	0.86	0.96	1.0E-03	1.2E-02
K11781	5-amino-6-(D-ribitylamino)uracil---L-tyrosine 4-hydroxyphenyl transferase [EC:2.5.1.147]	0.90	0.85	0.96	1.0E-03	1.2E-02
K04480	methanol---5-hydroxybenzimidazolylcobamide Co-methyltransferase [EC:2.1.1.90]	0.90	0.85	0.96	1.1E-03	1.2E-02
K10856	acetone carboxylase, gamma subunit [EC:6.4.1.6]	0.91	0.86	0.96	1.1E-03	1.2E-02
K00404	cytochrome c oxidase cbb3-type subunit I [EC:7.1.1.9]	0.91	0.85	0.96	1.1E-03	1.2E-02
K00685	arginyl-tRNA---protein transferase [EC:2.3.2.8]	0.91	0.85	0.96	1.1E-03	1.2E-02
K02323	DNA polymerase II small subunit [EC:2.7.7.7]	0.90	0.85	0.96	1.1E-03	1.2E-02
K00171	pyruvate ferredoxin oxidoreductase delta subunit [EC:1.2.7.1]	0.91	0.85	0.96	1.1E-03	1.2E-02
K01602	ribulose-bisphosphate carboxylase small chain [EC:4.1.1.39]	0.91	0.86	0.96	1.1E-03	1.2E-02
K00569	thiopurine S-methyltransferase [EC:2.1.1.67]	0.91	0.85	0.96	1.1E-03	1.2E-02
K12583	phosphatidylinositol alpha 1,6-mannosyltransferase [EC:2.4.1.-]	0.91	0.86	0.96	1.1E-03	1.2E-02
K11180	dissimilatory sulfite reductase alpha subunit [EC:1.8.99.5]	0.91	0.85	0.96	1.1E-03	1.2E-02
K04039	light-independent protochlorophyllide reductase subunit B [EC:1.3.7.7]	0.91	0.86	0.96	1.1E-03	1.2E-02
K01567	peptidoglycan-N-acetylmuramic acid deacetylase [EC:3.5.1.-]	0.90	0.85	0.96	1.1E-03	1.2E-02
K11336	3-vinyl bacteriochlorophyllide hydratase [EC:4.2.1.165]	0.91	0.86	0.96	1.1E-03	1.2E-02
K00661	maltose O-acetyltransferase [EC:2.3.1.79]	0.92	0.87	0.97	1.1E-03	1.2E-02
K00205	4Fe-4S ferredoxin	0.91	0.85	0.96	1.1E-03	1.2E-02
K03428	magnesium-protoporphyrin O-methyltransferase [EC:2.1.1.11]	0.91	0.86	0.96	1.1E-03	1.2E-02
K14731	epsilon-lactone hydrolase [EC:3.1.1.83]	0.91	0.86	0.96	1.1E-03	1.2E-02
K10797	2-enoate reductase [EC:1.3.1.31]	0.90	0.85	0.96	1.1E-03	1.2E-02
K00317	dimethylamine/trimethylamine dehydrogenase [EC:1.5.8.1 1.5.8.2]	0.91	0.86	0.96	1.1E-03	1.2E-02
K13767	enoyl-CoA hydratase [EC:4.2.1.17]	0.90	0.85	0.96	1.1E-03	1.2E-02
K10255	acyl-lipid omega-6 desaturase (Delta-12 desaturase) [EC:1.14.19.23 1.14.19.45]	0.91	0.85	0.96	1.2E-03	1.2E-02
K05973	poly(3-hydroxybutyrate) depolymerase [EC:3.1.1.75]	0.91	0.86	0.96	1.2E-03	1.2E-02
K01113	alkaline phosphatase D [EC:3.1.3.1]	0.91	0.85	0.96	1.2E-03	1.2E-02
K01907	acetoacetyl-CoA synthetase [EC:6.2.1.16]	0.91	0.85	0.96	1.2E-03	1.2E-02
K02636	cytochrome b6-f complex iron-sulfur subunit [EC:7.1.1.6]	0.91	0.86	0.96	1.2E-03	1.2E-02
K08966	2-hydroxy-3-keto-5-methylthiopentenyl-1-phosphate phosphatase [EC:3.1.3.87]	0.91	0.86	0.96	1.2E-03	1.2E-02
K01473	N-methylhydantoinase A [EC:3.5.2.14]	0.91	0.86	0.96	1.2E-03	1.2E-02
K00822	beta-alanine--pyruvate transaminase [EC:2.6.1.18]	0.91	0.85	0.96	1.2E-03	1.2E-02
K01434	penicillin G amidase [EC:3.5.1.11]	0.91	0.85	0.96	1.2E-03	1.2E-02
K08355	arsenite oxidase small subunit [EC:1.20.2.1 1.20.9.1]	0.91	0.86	0.96	1.2E-03	1.2E-02
K00114	alcohol dehydrogenase (cytochrome c) [EC:1.1.2.8]	0.91	0.86	0.96	1.2E-03	1.2E-02
K00527	ribonucleoside-triphosphate reductase (thioredoxin) [EC:1.17.4.2]	0.92	0.87	0.97	1.2E-03	1.2E-02
K00252	glutaryl-CoA dehydrogenase [EC:1.3.8.6]	0.91	0.85	0.96	1.2E-03	1.2E-02
K03930	putative tributyrin esterase [EC:3.1.1.-]	0.91	0.85	0.96	1.2E-03	1.2E-02
K07823	3-oxoadipyl-CoA thiolase [EC:2.3.1.174]	0.91	0.86	0.96	1.2E-03	1.2E-02
K00584	tetrahydromethanopterin S-methyltransferase subunit H [EC:2.1.1.86]	0.90	0.85	0.96	1.2E-03	1.2E-02
K00443	coenzyme F420 hydrogenase subunit gamma [EC:1.12.98.1]	0.91	0.85	0.96	1.2E-03	1.2E-02
K00992	N-acetyl-alpha-D-muramate 1-phosphate uridylyltransferase [EC:2.7.7.99]	0.91	0.85	0.96	1.2E-03	1.2E-02
K01728	pectate lyase [EC:4.2.2.2]	0.91	0.85	0.96	1.2E-03	1.2E-02

Predictor	Description	HR	2.5% CI	97.5% CI	P	FDR-adjusted P
K03540	ribonuclease P protein subunit RPR2 [EC:3.1.26.5]	0.90	0.85	0.96	1.2E-03	1.2E-02
K03539	ribonuclease P/MRP protein subunit RPP1 [EC:3.1.26.5]	0.91	0.85	0.96	1.2E-03	1.2E-02
K01556	kynureninase [EC:3.7.1.3]	0.91	0.85	0.96	1.2E-03	1.2E-02
K00270	phenylalanine dehydrogenase [EC:1.4.1.20]	0.90	0.85	0.96	1.3E-03	1.2E-02
K01004	phosphatidylcholine synthase [EC:2.7.8.24]	0.91	0.86	0.96	1.3E-03	1.2E-02
K15916	glucose/mannose-6-phosphate isomerase [EC:5.3.1.9 5.3.1.8]	0.90	0.85	0.96	1.3E-03	1.2E-02
K06375	stage 0 sporulation protein B (sporulation initiation phosphotransferase) [EC:2.7.-.-]	0.91	0.86	0.96	1.3E-03	1.2E-02
K10747	DNA ligase 1 [EC:6.5.1.1 6.5.1.6 6.5.1.7]	0.90	0.85	0.96	1.3E-03	1.2E-02
K04116	cyclohexanecarboxylate-CoA ligase [EC:6.2.1.-]	0.91	0.86	0.96	1.3E-03	1.2E-02
K13688	cyclic beta-1,2-glucan synthetase [EC:2.4.1.-]	0.91	0.86	0.96	1.3E-03	1.2E-02
K00436	NAD-reducing hydrogenase large subunit [EC:1.12.1.2]	0.91	0.86	0.96	1.3E-03	1.2E-02
K01455	formamidase [EC:3.5.1.49]	0.91	0.86	0.96	1.3E-03	1.2E-02
K00972	UDP-N-acetylglucosamine/UDP-N-acetylgalactosamine diphosphorylase [EC:2.7.7.23 2.7.7.83]	0.91	0.85	0.96	1.3E-03	1.2E-02
K15856	GDP-4-dehydro-6-deoxy-D-mannose reductase [EC:1.1.1.281]	0.91	0.85	0.96	1.3E-03	1.2E-02
K00446	catechol 2,3-dioxygenase [EC:1.13.11.2]	0.91	0.86	0.96	1.3E-03	1.2E-02
K00505	tyrosinase [EC:1.14.18.1]	0.91	0.86	0.97	1.3E-03	1.2E-02
K00201	formylmethanofuran dehydrogenase subunit B [EC:1.2.7.12]	0.90	0.85	0.96	1.3E-03	1.2E-02
K07557	archaeosine synthase alpha-subunit [EC:2.6.1.97 2.6.1.-]	0.91	0.85	0.96	1.3E-03	1.2E-02
K15011	two-component system, sensor histidine kinase RegB [EC:2.7.13.3]	0.91	0.86	0.96	1.3E-03	1.2E-02
K00554	tRNA (guanine37-N1)-methyltransferase [EC:2.1.1.228]	0.91	0.86	0.96	1.3E-03	1.2E-02
K04794	peptidyl-tRNA hydrolase, PTH2 family [EC:3.1.1.29]	0.90	0.85	0.96	1.3E-03	1.2E-02
K03793	pteridine reductase [EC:1.5.1.33]	0.91	0.86	0.96	1.3E-03	1.2E-02
K00304	sarcosine oxidase, subunit delta [EC:1.5.3.1]	0.91	0.86	0.96	1.4E-03	1.2E-02
K00761	uracil phosphoribosyltransferase [EC:2.4.2.9]	0.92	0.87	0.97	1.4E-03	1.2E-02
K04799	flap endonuclease-1 [EC:3.-.-.-]	0.91	0.85	0.96	1.4E-03	1.2E-02
K14339	alpha-1,6-mannosyltransferase [EC:2.4.1.-]	0.91	0.86	0.96	1.4E-03	1.2E-02
K16421	4-hydroxymandelate synthase [EC:1.13.11.46]	0.91	0.86	0.97	1.4E-03	1.2E-02
K03537	ribonuclease P/MRP protein subunit POP5 [EC:3.1.26.5]	0.91	0.85	0.96	1.4E-03	1.2E-02
K02476	two-component system, CitB family, sensor kinase [EC:2.7.13.3]	0.91	0.86	0.96	1.4E-03	1.2E-02
K09722	4-phosphopantoate---beta-alanine ligase [EC:6.3.2.36]	0.91	0.85	0.96	1.4E-03	1.2E-02
K00301	sarcosine oxidase [EC:1.5.3.1]	0.91	0.86	0.96	1.4E-03	1.2E-02
K14446	crotonyl-CoA carboxylase/reductase [EC:1.3.1.85]	0.91	0.85	0.96	1.4E-03	1.2E-02
K15429	tRNA (guanine37-N1)-methyltransferase [EC:2.1.1.228]	0.91	0.85	0.96	1.4E-03	1.2E-02
K00282	glycine dehydrogenase subunit 1 [EC:1.4.4.2]	0.91	0.85	0.96	1.4E-03	1.2E-02
K07654	two-component system, OmpR family, sensor histidine kinase MtrB [EC:2.7.13.3]	0.91	0.86	0.96	1.4E-03	1.2E-02
K01578	malonyl-CoA decarboxylase [EC:4.1.1.9]	0.91	0.86	0.96	1.4E-03	1.2E-02
K00459	nitronate monooxygenase [EC:1.13.12.16]	0.91	0.86	0.96	1.4E-03	1.2E-02
K12996	rhamnosyltransferase [EC:2.4.1.-]	0.91	0.85	0.96	1.4E-03	1.2E-02
K00925	acetate kinase [EC:2.7.2.1]	0.92	0.87	0.97	1.4E-03	1.2E-02
K01213	galacturan 1,4-alpha-galacturonidase [EC:3.2.1.67]	0.90	0.85	0.96	1.4E-03	1.2E-02
K10125	two-component system, NtrC family, C4-dicarboxylate transport sensor histidine kinase DctB [EC:2.7.13.3]	0.91	0.86	0.96	1.4E-03	1.3E-02
K06446	acyl-CoA dehydrogenase [EC:1.3.99.-]	0.91	0.86	0.96	1.4E-03	1.3E-02
K03660	N-glycosylase/DNA lyase [EC:3.2.2.- 4.2.99.18]	0.91	0.85	0.96	1.4E-03	1.3E-02
K15520	mycothiol synthase [EC:2.3.1.189]	0.91	0.86	0.96	1.5E-03	1.3E-02

Predictor	Description	HR	2.5% CI	97.5% CI	P	FDR-adjusted P
K00453	tryptophan 2,3-dioxygenase [EC:1.13.11.11]	0.91	0.86	0.96	1.5E-03	1.3E-02
K13280	signal peptidase I [EC:3.4.21.89]	0.91	0.85	0.96	1.5E-03	1.3E-02
K03536	ribonuclease P protein component [EC:3.1.26.5]	0.92	0.87	0.97	1.5E-03	1.3E-02
K00283	glycine dehydrogenase subunit 2 [EC:1.4.4.2]	0.91	0.85	0.96	1.5E-03	1.3E-02
K07680	two-component system, NarL family, sensor histidine kinase ComP [EC:2.7.13.3]	0.91	0.86	0.97	1.5E-03	1.3E-02
K11389	glyceraldehyde-3-phosphate dehydrogenase (ferredoxin) [EC:1.2.7.6]	0.91	0.86	0.97	1.5E-03	1.3E-02
K01265	methionyl aminopeptidase [EC:3.4.11.18]	0.92	0.88	0.97	1.5E-03	1.3E-02
K00411	ubiquinol-cytochrome c reductase iron-sulfur subunit [EC:7.1.1.8]	0.91	0.86	0.96	1.5E-03	1.3E-02
K00783	23S rRNA (pseudouridine1915-N3)-methyltransferase [EC:2.1.1.177]	0.91	0.85	0.96	1.5E-03	1.3E-02
K16514	4-oxalomesaconate tautomerase [EC:5.3.2.8]	0.91	0.86	0.97	1.6E-03	1.3E-02
K12995	O-antigen biosynthesis alpha-1,3-mannosyltransferase [EC:2.4.1.348 2.4.1.-]	0.91	0.85	0.96	1.6E-03	1.4E-02
K05518	phosphoserine phosphatase RsbX [EC:3.1.3.3]	0.91	0.86	0.97	1.6E-03	1.4E-02
K08965	2,3-diketo-5-methylthiopentyl-1-phosphate enolase [EC:5.3.2.5]	0.91	0.86	0.97	1.6E-03	1.4E-02
K10854	acetone carboxylase, alpha subunit [EC:6.4.1.6]	0.91	0.86	0.97	1.6E-03	1.4E-02
K00150	glyceraldehyde-3-phosphate dehydrogenase (NAD(P)) [EC:1.2.1.59]	0.91	0.85	0.96	1.6E-03	1.4E-02
K15358	enamidase [EC:3.5.2.18]	0.91	0.86	0.97	1.6E-03	1.4E-02
K08082	two-component system, LytTR family, sensor histidine kinase AlgZ [EC:2.7.13.3]	0.91	0.86	0.97	1.7E-03	1.4E-02
K11260	4Fe-4S ferredoxin	0.91	0.86	0.96	1.7E-03	1.4E-02
K00457	4-hydroxyphenylpyruvate dioxygenase [EC:1.13.11.27]	0.91	0.86	0.96	1.7E-03	1.4E-02
K11333	3,8-divinyl chlorophyllide a/chlorophyllide a reductase subunit X [EC:1.3.7.14 1.3.7.15]	0.91	0.86	0.97	1.7E-03	1.4E-02
K01535	H+-transporting ATPase [EC:7.1.2.1]	0.91	0.86	0.97	1.7E-03	1.4E-02
K11176	IMP cyclohydrolase [EC:3.5.4.10]	0.91	0.86	0.96	1.7E-03	1.4E-02
K01474	N-methylhydantoinase B [EC:3.5.2.14]	0.91	0.86	0.97	1.7E-03	1.4E-02
K14080	[methyl-Co(III) methanol/glycine betaine-specific corrinoid protein]:coenzyme M methyltransferase [EC:2.1.1.246 2.1.1.377]	0.91	0.85	0.96	1.7E-03	1.4E-02
K00118	glucose-fructose oxidoreductase [EC:1.1.99.28]	0.91	0.86	0.97	1.7E-03	1.4E-02
K00302	sarcosine oxidase, subunit alpha [EC:1.5.3.1]	0.91	0.86	0.96	1.7E-03	1.4E-02
K02826	cytochrome aa3-600 menaquinol oxidase subunit II [EC:7.1.1.5]	0.91	0.86	0.97	1.7E-03	1.4E-02
K01458	N-formylglutamate deformylase [EC:3.5.1.68]	0.91	0.86	0.97	1.7E-03	1.4E-02
K05602	histidinol-phosphatase [EC:3.1.3.15]	0.91	0.86	0.97	1.7E-03	1.4E-02
K01083	3-phytase [EC:3.1.3.8]	0.91	0.86	0.97	1.7E-03	1.4E-02
K01090	protein phosphatase [EC:3.1.3.16]	0.91	0.86	0.97	1.7E-03	1.4E-02
K13006	UDP-perosamine 4-acetyltransferase [EC:2.3.1.-]	0.91	0.86	0.97	1.7E-03	1.4E-02
K01563	haloalkane dehalogenase [EC:3.8.1.5]	0.91	0.86	0.97	1.7E-03	1.4E-02
K00583	tetrahydromethanopterin S-methyltransferase subunit G [EC:2.1.1.86]	0.91	0.86	0.96	1.8E-03	1.4E-02
K01915	glutamine synthetase [EC:6.3.1.2]	0.92	0.87	0.97	1.8E-03	1.4E-02
K03403	magnesium chelatase subunit H [EC:6.6.1.1]	0.91	0.86	0.97	1.8E-03	1.4E-02
K05281	2'-hydroxyisoflavone reductase [EC:1.3.1.45]	0.92	0.87	0.97	1.8E-03	1.4E-02
K03330	glutamyl-tRNA(Gln) amidotransferase subunit E [EC:6.3.5.7]	0.91	0.85	0.96	1.8E-03	1.4E-02
K08691	malyl-CoA/(S)-citramalyl-CoA lyase [EC:4.1.3.24 4.1.3.25]	0.91	0.86	0.97	1.8E-03	1.4E-02
K15511	benzoyl-CoA 2,3-epoxidase subunit A [EC:1.14.13.208]	0.91	0.86	0.97	1.8E-03	1.4E-02
K02668	two-component system, NtrC family, sensor histidine kinase PilS [EC:2.7.13.3]	0.91	0.86	0.97	1.8E-03	1.4E-02
K00594	alditol oxidase [EC:1.1.3.41]	0.91	0.86	0.97	1.9E-03	1.4E-02
K03047	DNA-directed RNA polymerase subunit D [EC:2.7.7.6]	0.91	0.86	0.97	1.9E-03	1.4E-02
K00187	2-oxoisovalerate ferredoxin oxidoreductase beta subunit [EC:1.2.7.7]	0.91	0.86	0.97	1.9E-03	1.4E-02

Predictor	Description	HR	2.5% CI	97.5% CI	P	FDR-adjusted P
K08587	clostripain [EC:3.4.22.8]	0.91	0.86	0.97	1.9E-03	1.4E-02
K16619	phospholipase C / alpha-toxin [EC:3.1.4.3]	0.91	0.86	0.97	1.9E-03	1.4E-02
K16011	mannose-1-phosphate guanylyltransferase / mannose-6-phosphate isomerase [EC:2.7.7.13 5.3.1.8]	0.91	0.86	0.97	1.9E-03	1.4E-02
K00608	aspartate carbamoyltransferase [EC:2.1.3.2]	0.91	0.86	0.97	1.9E-03	1.4E-02
K01770	2-C-methyl-D-erythritol 2,4-cyclodiphosphate synthase [EC:4.6.1.12]	0.92	0.88	0.97	1.9E-03	1.4E-02
K16171	fumarylacetoacetate (FAA) hydrolase [EC:3.7.1.2]	0.91	0.86	0.97	1.9E-03	1.4E-02
K04566	lysyl-tRNA synthetase, class I [EC:6.1.1.6]	0.91	0.85	0.96	1.9E-03	1.5E-02
K01027	3-oxoacid CoA-transferase [EC:2.8.3.5]	0.91	0.86	0.97	1.9E-03	1.5E-02
K05827	[lysine-biosynthesis-protein LysW]---L-2-aminoadipate ligase [EC:6.3.2.43]	0.91	0.86	0.97	1.9E-03	1.5E-02
K10674	ectoine hydroxylase [EC:1.14.11.55]	0.91	0.86	0.97	1.9E-03	1.5E-02
K11131	H/ACA ribonucleoprotein complex subunit 4 [EC:5.4.99.-]	0.91	0.86	0.97	2.0E-03	1.5E-02
K10220	4-oxalmesaconate hydratase [EC:4.2.1.83]	0.91	0.86	0.97	2.0E-03	1.5E-02
K07254	tRNA (cytidine56-2'-O)-methyltransferase [EC:2.1.1.206]	0.91	0.86	0.97	2.0E-03	1.5E-02
K01477	allantoicase [EC:3.5.3.4]	0.91	0.86	0.97	2.0E-03	1.5E-02
K00126	formate dehydrogenase subunit delta [EC:1.17.1.9]	0.91	0.86	0.97	2.0E-03	1.5E-02
K00672	formylmethanofuran--tetrahydromethanopterin N-formyltransferase [EC:2.3.1.101]	0.91	0.85	0.97	2.0E-03	1.5E-02
K07155	quercetin 2,3-dioxygenase [EC:1.13.11.24]	0.91	0.86	0.97	2.0E-03	1.5E-02
K07536	2-ketocyclohexanecarboxyl-CoA hydrolase [EC:3.1.2.-]	0.91	0.86	0.97	2.0E-03	1.5E-02
K13924	two-component system, chemotaxis family, CheB/CheR fusion protein [EC:2.1.1.80 3.1.1.61]	0.91	0.86	0.97	2.1E-03	1.5E-02
K10672	glycine reductase complex component B subunit gamma [EC:1.21.4.2]	0.91	0.86	0.97	2.1E-03	1.5E-02
K00372	assimilatory nitrate reductase catalytic subunit [EC:1.7.99.-]	0.91	0.86	0.97	2.1E-03	1.5E-02
K03520	aerobic carbon-monoxide dehydrogenase large subunit [EC:1.2.5.3]	0.91	0.86	0.97	2.1E-03	1.5E-02
K01236	maltooligosyltrehalose trehalohydrolase [EC:3.2.1.141]	0.91	0.86	0.97	2.1E-03	1.5E-02
K01795	mannuronan 5-epimerase [EC:5.1.3.37]	0.91	0.86	0.97	2.1E-03	1.5E-02
K03550	holliday junction DNA helicase RuvA [EC:3.6.4.12]	0.92	0.88	0.97	2.1E-03	1.5E-02
K10531	L-ornithine N5-monooxygenase [EC:1.14.13.195 1.14.13.196]	0.91	0.86	0.97	2.1E-03	1.5E-02
K06121	glycerol dehydratase medium subunit [EC:4.2.1.30]	0.91	0.86	0.97	2.1E-03	1.5E-02
K13668	phosphatidyl-myo-inositol dimannoside synthase [EC:2.4.1.346]	0.91	0.86	0.97	2.1E-03	1.5E-02
K00132	acetaldehyde dehydrogenase (acetylating) [EC:1.2.1.10]	0.91	0.86	0.97	2.1E-03	1.5E-02
K01576	benzoylformate decarboxylase [EC:4.1.1.7]	0.91	0.86	0.97	2.1E-03	1.5E-02
K01170	tRNA-intron endonuclease, archaea type [EC:4.6.1.16]	0.91	0.86	0.97	2.1E-03	1.5E-02
K03411	chemotaxis protein CheD [EC:3.5.1.44]	0.91	0.86	0.97	2.1E-03	1.5E-02
K02493	release factor glutamine methyltransferase [EC:2.1.1.297]	0.92	0.88	0.97	2.2E-03	1.5E-02
K09845	1-hydroxycarotenoid 3,4-desaturase [EC:1.3.99.27]	0.91	0.86	0.97	2.2E-03	1.5E-02
K08097	phosphosulfolactate synthase [EC:4.4.1.19]	0.91	0.86	0.97	2.2E-03	1.5E-02
K11261	formylmethanofuran dehydrogenase subunit E [EC:1.2.7.12]	0.91	0.86	0.97	2.2E-03	1.5E-02
K08646	peptidyl-Lys metalloendopeptidase [EC:3.4.24.20]	0.92	0.87	0.97	2.2E-03	1.5E-02
K10670	glycine/sarcosine/betaine reductase complex component A [EC:1.21.4.2 1.21.4.3 1.21.4.4]	0.91	0.86	0.97	2.2E-03	1.5E-02
K01039	glutaconate CoA-transferase, subunit A [EC:2.8.3.12]	0.91	0.86	0.97	2.2E-03	1.5E-02
K11915	serine/threonine protein phosphatase Stp1 [EC:3.1.3.16]	0.92	0.87	0.97	2.2E-03	1.5E-02
K01707	5-dehydro-4-deoxyglucarate dehydratase [EC:4.2.1.41]	0.91	0.86	0.97	2.2E-03	1.5E-02
K14164	glycyl-tRNA synthetase [EC:6.1.1.14]	0.92	0.86	0.97	2.2E-03	1.5E-02
K00440	coenzyme F420 hydrogenase subunit alpha [EC:1.12.98.1]	0.91	0.86	0.97	2.3E-03	1.5E-02
K01133	choline-sulfatase [EC:3.1.6.6]	0.91	0.86	0.97	2.3E-03	1.5E-02

Predictor	Description	HR	2.5% CI	97.5% CI	P	FDR-adjusted P
K01727	hyaluronate lyase [EC:4.2.2.1]	0.91	0.86	0.97	2.3E-03	1.5E-02
K13766	methylglutaconyl-CoA hydratase [EC:4.2.1.18]	0.91	0.86	0.97	2.3E-03	1.5E-02
K01449	N-acetylmuramoyl-L-alanine amidase [EC:3.5.1.28]	0.91	0.86	0.97	2.3E-03	1.5E-02
K00856	adenosine kinase [EC:2.7.1.20]	0.91	0.86	0.97	2.3E-03	1.5E-02
K01067	acetyl-CoA hydrolase [EC:3.1.2.1]	0.92	0.86	0.97	2.3E-03	1.5E-02
K15778	phosphomannomutase / phosphoglucomutase [EC:5.4.2.8 5.4.2.2]	0.91	0.86	0.97	2.3E-03	1.5E-02
K14520	4-hydroxyacetophenone monooxygenase [EC:1.14.13.84]	0.92	0.87	0.97	2.3E-03	1.5E-02
K05577	NAD(P)H-quinone oxidoreductase subunit 5 [EC:7.1.1.2]	0.91	0.86	0.97	2.3E-03	1.5E-02
K06044	(1->4)-alpha-D-glucan 1-alpha-D-glucosylmutase [EC:5.4.99.15]	0.91	0.86	0.97	2.3E-03	1.5E-02
K07537	cyclohexa-1,5-dienecarbonyl-CoA hydratase [EC:4.2.1.100]	0.91	0.86	0.97	2.3E-03	1.5E-02
K15512	benzoyl-CoA 2,3-epoxidase subunit B [EC:1.14.13.208]	0.91	0.86	0.97	2.3E-03	1.5E-02
K03396	S-(hydroxymethyl)glutathione synthase [EC:4.4.1.22]	0.91	0.86	0.97	2.3E-03	1.5E-02
K03166	DNA topoisomerase VI subunit A [EC:5.6.2.2]	0.91	0.86	0.97	2.3E-03	1.6E-02
K06443	lycopene beta-cyclase [EC:5.5.1.19]	0.91	0.86	0.97	2.3E-03	1.6E-02
K05305	fucokinase [EC:2.7.1.52]	0.92	0.87	0.97	2.3E-03	1.6E-02
K16649	rhamnopyranosyl-N-acetylglucosaminyl-diphospho-decaprenol beta-1,3/1,4-galactofuranosyltransferase [EC:2.4.1.287]	0.92	0.87	0.97	2.4E-03	1.6E-02
K10562	rhamnose transport system ATP-binding protein [EC:7.5.2.-]	0.91	0.86	0.97	2.4E-03	1.6E-02
K05580	NAD(P)H-quinone oxidoreductase subunit I [EC:7.1.1.2]	0.91	0.86	0.97	2.4E-03	1.6E-02
K00510	heme oxygenase 1 [EC:1.14.14.18]	0.91	0.86	0.97	2.4E-03	1.6E-02
K15652	3-dehydroshikimate dehydratase [EC:4.2.1.118]	0.92	0.87	0.97	2.4E-03	1.6E-02
K15888	tritrans,polycis-undecaprenyl-diphosphate synthase [geranylgeranyl-diphosphate specific] [EC:2.5.1.89]	0.91	0.86	0.97	2.5E-03	1.6E-02
K13017	UDP-2-acetamido-2-deoxy-ribo-hexuluronate aminotransferase [EC:2.6.1.98]	0.91	0.86	0.97	2.5E-03	1.6E-02
K03333	cholesterol oxidase [EC:1.1.3.6]	0.91	0.86	0.97	2.5E-03	1.6E-02
K13812	bifunctional enzyme Fae/Hps [EC:4.2.1.147 4.1.2.43]	0.91	0.86	0.97	2.5E-03	1.6E-02
K00448	protocatechuate 3,4-dioxygenase, alpha subunit [EC:1.13.11.3]	0.91	0.86	0.97	2.5E-03	1.6E-02
K01209	alpha-L-arabinofuranosidase [EC:3.2.1.55]	0.92	0.88	0.97	2.5E-03	1.6E-02
K00496	alkane 1-monooxygenase [EC:1.14.15.3]	0.91	0.86	0.97	2.5E-03	1.6E-02
K08687	N-carbamoylsarcosine amidase [EC:3.5.1.59]	0.91	0.86	0.97	2.5E-03	1.6E-02
K14126	F420-non-reducing hydrogenase large subunit [EC:1.12.99.- 1.8.98.5]	0.91	0.86	0.97	2.5E-03	1.6E-02
K02480	two-component system, NarL family, sensor kinase [EC:2.7.13.3]	0.91	0.86	0.97	2.5E-03	1.6E-02
K03041	DNA-directed RNA polymerase subunit A' [EC:2.7.7.6]	0.91	0.86	0.97	2.5E-03	1.6E-02
K00624	carnitine O-acetyltransferase [EC:2.3.1.7]	0.92	0.87	0.97	2.6E-03	1.7E-02
K04108	4-hydroxybenzoyl-CoA reductase subunit alpha [EC:1.1.7.1]	0.91	0.86	0.97	2.6E-03	1.7E-02
K00952	nicotinamide-nucleotide adenylyltransferase [EC:2.7.7.1]	0.91	0.86	0.97	2.6E-03	1.7E-02
K01184	polygalacturonase [EC:3.2.1.15]	0.92	0.87	0.97	2.7E-03	1.7E-02
K04036	divinyl protochlorophyllide a 8-vinyl-reductase [EC:1.-.-.-]	0.92	0.87	0.97	2.7E-03	1.7E-02
K00320	5,10-methylenetetrahydromethanopterin reductase [EC:1.5.98.2]	0.91	0.86	0.97	2.7E-03	1.7E-02
K15898	pseudaminic acid synthase [EC:2.5.1.97]	0.91	0.86	0.97	2.7E-03	1.7E-02
K01415	endothelin-converting enzyme [EC:3.4.24.71]	0.92	0.87	0.97	2.7E-03	1.7E-02
K04038	light-independent protochlorophyllide reductase subunit N [EC:1.3.7.7]	0.91	0.86	0.97	2.7E-03	1.7E-02
K15521	D-inositol-3-phosphate glycosyltransferase [EC:2.4.1.250]	0.91	0.86	0.97	2.7E-03	1.7E-02
K01857	3-carboxy-cis,cis-muconate cycloisomerase [EC:5.5.1.2]	0.91	0.86	0.97	2.8E-03	1.7E-02
K07055	tRNA wybutosine-synthesizing protein 2 [EC:2.5.1.114]	0.91	0.86	0.97	2.8E-03	1.7E-02
K14949	serine/threonine-protein kinase PknG [EC:2.7.11.1]	0.92	0.86	0.97	2.8E-03	1.7E-02

Predictor	Description	HR	2.5% CI	97.5% CI	P	FDR-adjusted P
K10215	monooxygenase [EC:1.14.13.-]	0.92	0.87	0.97	2.8E-03	1.7E-02
K00481	p-hydroxybenzoate 3-monooxygenase [EC:1.14.13.2]	0.91	0.86	0.97	2.8E-03	1.7E-02
K10764	O-succinylhomoserine sulfhydrylase [EC:2.5.1.-]	0.91	0.86	0.97	2.8E-03	1.7E-02
K07535	2-hydroxycyclohexanecarboxyl-CoA dehydrogenase [EC:1.1.1.-]	0.92	0.87	0.97	2.8E-03	1.8E-02
K01432	arylformamidase [EC:3.5.1.9]	0.92	0.87	0.97	2.8E-03	1.8E-02
K04100	protocatechuate 4,5-dioxygenase, alpha chain [EC:1.13.11.8]	0.92	0.86	0.97	2.8E-03	1.8E-02
K01286	D-alanyl-D-alanine carboxypeptidase [EC:3.4.16.4]	0.91	0.86	0.97	2.9E-03	1.8E-02
K01387	microbial collagenase [EC:3.4.24.3]	0.92	0.86	0.97	2.9E-03	1.8E-02
K05994	bacterial leucyl aminopeptidase [EC:3.4.11.10]	0.92	0.87	0.97	2.9E-03	1.8E-02
K11942	isobutyryl-CoA mutase [EC:5.4.99.13]	0.91	0.86	0.97	2.9E-03	1.8E-02
K15739	D-alanine---(R)-lactate ligase [EC:6.1.2.1]	0.92	0.87	0.97	3.0E-03	1.8E-02
K13942	5,10-methenyltetrahydromethanopterin hydrogenase [EC:1.12.98.2]	0.91	0.86	0.97	3.0E-03	1.8E-02
K06928	nucleoside-triphosphatase [EC:3.6.1.15]	0.91	0.86	0.97	3.0E-03	1.8E-02
K01796	alpha-methylacyl-CoA racemase [EC:5.1.99.4]	0.91	0.86	0.97	3.0E-03	1.8E-02
K03716	spore photoproduct lyase [EC:4.1.99.14]	0.91	0.86	0.97	3.0E-03	1.8E-02
K16047	3-hydroxy-9,10-secoandrosta-1,3,5(10)-triene-9,17-dione monooxygenase [EC:1.14.14.12]	0.92	0.87	0.97	3.0E-03	1.8E-02
K13057	trehalose synthase [EC:2.4.1.245]	0.92	0.86	0.97	3.0E-03	1.8E-02
K01499	methenyltetrahydromethanopterin cyclohydrolase [EC:3.5.4.27]	0.91	0.86	0.97	3.0E-03	1.8E-02
K01055	3-oxoadipate enol-lactonase [EC:3.1.1.24]	0.92	0.86	0.97	3.1E-03	1.9E-02
K07718	two-component system, sensor histidine kinase YesM [EC:2.7.13.3]	0.91	0.86	0.97	3.1E-03	1.9E-02
K03921	acyl-[acyl-carrier-protein] desaturase [EC:1.14.19.2 1.14.19.11 1.14.19.26]	0.92	0.86	0.97	3.1E-03	1.9E-02
K10844	DNA excision repair protein ERCC-2 [EC:3.6.4.12]	0.91	0.86	0.97	3.1E-03	1.9E-02
K13481	xanthine dehydrogenase small subunit [EC:1.17.1.4]	0.92	0.86	0.97	3.2E-03	1.9E-02
K00612	carbamoyltransferase [EC:2.1.3.-]	0.91	0.86	0.97	3.2E-03	1.9E-02
K05886	serine 3-dehydrogenase (NADP+) [EC:1.1.1.276]	0.92	0.86	0.97	3.2E-03	1.9E-02
K05889	polyvinyl alcohol dehydrogenase (cytochrome) [EC:1.1.2.6]	0.92	0.87	0.97	3.2E-03	1.9E-02
K13587	two-component system, cell cycle sensor histidine kinase and response regulator CckA [EC:2.7.13.3]	0.92	0.87	0.97	3.2E-03	1.9E-02
K13063	2-amino-4-deoxychorismate synthase [EC:2.6.1.86]	0.92	0.87	0.97	3.2E-03	1.9E-02
K03538	ribonuclease P protein subunit POP4 [EC:3.1.26.5]	0.91	0.86	0.97	3.2E-03	1.9E-02
K11731	citronellyl-CoA dehydrogenase [EC:1.3.99.-]	0.92	0.86	0.97	3.2E-03	1.9E-02
K01028	3-oxoacid CoA-transferase subunit A [EC:2.8.3.5]	0.91	0.86	0.97	3.2E-03	1.9E-02
K06981	isopentenyl phosphate kinase [EC:2.7.4.26]	0.91	0.86	0.97	3.3E-03	1.9E-02
K00830	alanine-glyoxylate transaminase / serine-glyoxylate transaminase / serine-pyruvate transaminase [EC:2.6.1.44 2.6.1.45 2.6.1.51]	0.92	0.86	0.97	3.3E-03	1.9E-02
K03042	DNA-directed RNA polymerase subunit A\ [EC:2.7.7.6]	0.91	0.86	0.97	3.3E-03	1.9E-02
K00518	nickel superoxide dismutase [EC:1.15.1.1]	0.92	0.87	0.97	3.3E-03	1.9E-02
K10715	two-component system, sensor histidine kinase RpfC [EC:2.7.13.3]	0.92	0.87	0.97	3.3E-03	1.9E-02
K13663	acyltransferase [EC:2.3.1.-]	0.92	0.86	0.97	3.3E-03	1.9E-02
K15230	ATP-citrate lyase alpha-subunit [EC:2.3.3.8]	0.92	0.87	0.97	3.3E-03	1.9E-02
K15231	ATP-citrate lyase beta-subunit [EC:2.3.3.8]	0.92	0.87	0.97	3.3E-03	1.9E-02
K00555	tRNA (guanine26-N2/guanine27-N2)-dimethyltransferase [EC:2.1.1.215 2.1.1.216]	0.91	0.86	0.97	3.3E-03	1.9E-02
K16188	tetraprenyl-beta-curcumene synthase [EC:4.2.3.130]	0.92	0.86	0.97	3.3E-03	1.9E-02
K11754	dihydrofolate synthase / folylpolyglutamate synthase [EC:6.3.2.12 6.3.2.17]	0.93	0.88	0.97	3.3E-03	1.9E-02
K01768	adenylate cyclase [EC:4.6.1.1]	0.91	0.86	0.97	3.3E-03	1.9E-02
K11528	UDP-N-acetylglucosamine pyrophosphorylase [EC:2.7.7.23]	0.92	0.87	0.97	3.3E-03	1.9E-02

Predictor	Description	HR	2.5% CI	97.5% CI	P	FDR-adjusted P
K08688	creatinase [EC:3.5.3.3]	0.92	0.87	0.97	3.4E-03	1.9E-02
K08295	2-aminobenzoate-CoA ligase [EC:6.2.1.32]	0.92	0.86	0.97	3.4E-03	1.9E-02
K16650	galactofuranosylgalactofuranosylrhamnosyl-N-acetylglucosaminyl-diphospho-decaprenol beta-1,5/1,6-galactofuranosyltransferase [EC:2.4.1.288]	0.91	0.86	0.97	3.4E-03	1.9E-02
K00154	coniferyl-aldehyde dehydrogenase [EC:1.2.1.68]	0.91	0.86	0.97	3.4E-03	1.9E-02
K07739	elongator complex protein 3 [EC:2.3.1.48]	0.92	0.86	0.97	3.4E-03	1.9E-02
K03053	DNA-directed RNA polymerase subunit H [EC:2.7.7.6]	0.91	0.86	0.97	3.4E-03	1.9E-02
K01342	subtilisin [EC:3.4.21.62]	0.92	0.87	0.97	3.4E-03	1.9E-02
K07732	riboflavin kinase, archaea type [EC:2.7.1.161]	0.91	0.86	0.97	3.4E-03	1.9E-02
K14658	nodulation protein A [EC:2.3.1.-]	0.92	0.87	0.97	3.4E-03	1.9E-02
K00441	coenzyme F420 hydrogenase subunit beta [EC:1.12.98.1]	0.91	0.86	0.97	3.4E-03	1.9E-02
K15022	formate dehydrogenase (NADP+) beta subunit [EC:1.17.1.10]	0.92	0.87	0.97	3.5E-03	1.9E-02
K06015	N-acyl-D-amino-acid deacylase [EC:3.5.1.81]	0.91	0.86	0.97	3.5E-03	2.0E-02
K02201	pantetheine-phosphate adenylyltransferase [EC:2.7.7.3]	0.91	0.86	0.97	3.5E-03	2.0E-02
K10187	germacradienol/geosmin synthase [EC:4.2.3.22 4.2.3.75 4.1.99.16]	0.92	0.87	0.97	3.5E-03	2.0E-02
K01208	cyclomaltodextrinase / maltogenic alpha-amylase / neopullulanase [EC:3.2.1.54 3.2.1.133 3.2.1.135]	0.91	0.86	0.97	3.5E-03	2.0E-02
K01392	thimet oligopeptidase [EC:3.4.24.15]	0.92	0.87	0.97	3.5E-03	2.0E-02
K03433	proteasome beta subunit [EC:3.4.25.1]	0.91	0.86	0.97	3.6E-03	2.0E-02
K01212	levanase [EC:3.2.1.65]	0.92	0.87	0.97	3.6E-03	2.0E-02
K10942	two-component system, sensor histidine kinase FlrB [EC:2.7.13.3]	0.92	0.87	0.97	3.6E-03	2.0E-02
K13671	alpha-1,2-mannosyltransferase [EC:2.4.1.-]	0.92	0.86	0.97	3.6E-03	2.0E-02
K11212	LPPG:FO 2-phospho-L-lactate transferase [EC:2.7.8.28]	0.91	0.86	0.97	3.6E-03	2.0E-02
K14941	2-phospho-L-lactate/phosphoenolpyruvate guanylyltransferase [EC:2.7.7.68 2.7.7.105]	0.91	0.86	0.97	3.7E-03	2.0E-02
K03153	glycine oxidase [EC:1.4.3.19]	0.92	0.86	0.97	3.7E-03	2.0E-02
K00186	2-oxoisovalerate ferredoxin oxidoreductase alpha subunit [EC:1.2.7.7]	0.92	0.87	0.97	3.7E-03	2.0E-02
K00360	assimilatory nitrate reductase electron transfer subunit [EC:1.7.99.-]	0.92	0.87	0.97	3.8E-03	2.1E-02
K03167	DNA topoisomerase VI subunit B [EC:5.6.2.2]	0.91	0.86	0.97	3.9E-03	2.1E-02
K16653	decaprenylphospho-beta-D-ribofuranose 2-oxidase [EC:1.1.98.3]	0.92	0.87	0.97	3.9E-03	2.1E-02
K09461	anthraniloyl-CoA monooxygenase [EC:1.14.13.40]	0.92	0.87	0.97	3.9E-03	2.1E-02
K15755	2'-aminobiphenyl-2,3-diol 1,2-dioxygenase, large subunit [EC:1.13.11.-]	0.92	0.87	0.97	4.0E-03	2.2E-02
K16652	decaprenylphospho-beta-D-erythro-pentofuranosid-2-ulose 2-reductase [EC:1.1.1.333]	0.92	0.87	0.97	4.0E-03	2.2E-02
K13039	sulfopyruvate decarboxylase subunit beta [EC:4.1.1.79]	0.92	0.86	0.97	4.0E-03	2.2E-02
K00305	sarcosine oxidase, subunit gamma [EC:1.5.3.1]	0.92	0.86	0.97	4.0E-03	2.2E-02
K00368	nitrite reductase (NO-forming) [EC:1.7.2.1]	0.92	0.86	0.97	4.0E-03	2.2E-02
K02303	uroporphyrin-III C-methyltransferase [EC:2.1.1.107]	0.91	0.86	0.97	4.0E-03	2.2E-02
K15509	sulfopropanediol 3-dehydrogenase [EC:1.1.1.308]	0.92	0.87	0.97	4.0E-03	2.2E-02
K16320	anthranilate 1,2-dioxygenase small subunit [EC:1.14.12.1]	0.92	0.87	0.97	4.0E-03	2.2E-02
K00011	aldehyde reductase [EC:1.1.1.21]	0.92	0.87	0.97	4.1E-03	2.2E-02
K00256	NA	0.92	0.87	0.97	4.1E-03	2.2E-02
K00043	4-hydroxybutyrate dehydrogenase [EC:1.1.1.61]	0.92	0.86	0.97	4.1E-03	2.2E-02
K07178	RIO kinase 1 [EC:2.7.11.1]	0.91	0.86	0.97	4.1E-03	2.2E-02
K03795	sirohydrochlorin cobaltochelatase [EC:4.99.1.3]	0.91	0.86	0.97	4.1E-03	2.2E-02
K00172	pyruvate ferredoxin oxidoreductase gamma subunit [EC:1.2.7.1]	0.92	0.86	0.97	4.1E-03	2.2E-02
K09759	nondiscriminating aspartyl-tRNA synthetase [EC:6.1.1.23]	0.92	0.86	0.97	4.2E-03	2.2E-02
K02626	arginine decarboxylase [EC:4.1.1.19]	0.92	0.86	0.97	4.2E-03	2.2E-02

Predictor	Description	HR	2.5% CI	97.5% CI	P	FDR-adjusted P
K05297	rubredoxin---NAD+ reductase [EC:1.18.1.1]	0.92	0.87	0.97	4.2E-03	2.2E-02
K08256	phosphatidyl-myo-inositol alpha-mannosyltransferase [EC:2.4.1.345]	0.92	0.86	0.97	4.2E-03	2.2E-02
K07303	isoquinoline 1-oxidoreductase subunit beta [EC:1.3.99.16]	0.92	0.86	0.97	4.2E-03	2.2E-02
K15763	toluene monooxygenase system protein D [EC:1.14.13.236 1.14.13.-]	0.92	0.87	0.97	4.2E-03	2.2E-02
K13713	fusion protein PurCD [EC:6.3.2.6 6.3.4.13]	0.92	0.86	0.97	4.2E-03	2.2E-02
K12989	mannosyltransferase [EC:2.4.1.-]	0.92	0.87	0.97	4.3E-03	2.2E-02
K01029	3-oxoacid CoA-transferase subunit B [EC:2.8.3.5]	0.92	0.86	0.97	4.3E-03	2.2E-02
K13693	glucosyl-3-phosphoglycerate synthase [EC:2.4.1.266]	0.92	0.86	0.97	4.3E-03	2.2E-02
K07302	isoquinoline 1-oxidoreductase subunit alpha [EC:1.3.99.16]	0.92	0.86	0.97	4.3E-03	2.2E-02
K04090	indolepyruvate ferredoxin oxidoreductase [EC:1.2.7.8]	0.92	0.86	0.97	4.3E-03	2.2E-02
K12979	beta-hydroxylase [EC:1.14.11.-]	0.92	0.87	0.97	4.3E-03	2.2E-02
K00809	deoxyhypusine synthase [EC:2.5.1.46]	0.92	0.86	0.97	4.3E-03	2.2E-02
K02278	prepilin peptidase CpaA [EC:3.4.23.43]	0.92	0.86	0.97	4.3E-03	2.2E-02
K13950	para-aminobenzoate synthetase [EC:2.6.1.85]	0.92	0.86	0.97	4.3E-03	2.2E-02
K07697	two-component system, sporulation sensor kinase B [EC:2.7.13.3]	0.92	0.87	0.97	4.3E-03	2.2E-02
K11779	FO synthase [EC:2.5.1.147 4.3.1.32]	0.92	0.87	0.97	4.3E-03	2.2E-02
K04711	dihydroceramidase [EC:3.5.1.-]	0.92	0.87	0.97	4.3E-03	2.2E-02
K00200	formylmethanofuran dehydrogenase subunit A [EC:1.2.7.12]	0.92	0.87	0.97	4.4E-03	2.2E-02
K13530	AraC family transcriptional regulator, regulatory protein of adaptative response / methylphosphotriester-DNA alkyltransferase methyltransferase [EC:2.1.1.-]	0.92	0.86	0.97	4.4E-03	2.2E-02
K03862	vanillate monooxygenase [EC:1.14.13.82]	0.92	0.86	0.97	4.4E-03	2.2E-02
K06137	pyrroloquinoline-quinone synthase [EC:1.3.3.11]	0.92	0.87	0.97	4.4E-03	2.2E-02
K00059	3-oxoacyl-[acyl-carrier protein] reductase [EC:1.1.1.100]	0.93	0.88	0.98	4.4E-03	2.3E-02
K10795	D-proline reductase (dithiol)-stabilizing protein PrdD	0.92	0.87	0.98	4.4E-03	2.3E-02
K01308	g-D-glutamyl-meso-diaminopimelate peptidase [EC:3.4.19.11]	0.92	0.86	0.97	4.5E-03	2.3E-02
K10218	4-hydroxy-4-methyl-2-oxoglutarate aldolase [EC:4.1.3.17]	0.92	0.86	0.97	4.5E-03	2.3E-02
K05829	LysW-gamma-L-alpha-aminoadipyl-6-phosphate/LysW-L-glutamyl-5-phosphate reductase [EC:1.2.1.103 1.2.1.106]	0.92	0.87	0.98	4.5E-03	2.3E-02
K01668	tyrosine phenol-lyase [EC:4.1.99.2]	0.92	0.86	0.97	4.5E-03	2.3E-02
K15746	beta-carotene 3-hydroxylase [EC:1.14.15.24]	0.92	0.87	0.97	4.5E-03	2.3E-02
K07777	two-component system, NarL family, sensor histidine kinase DegS [EC:2.7.13.3]	0.92	0.86	0.97	4.5E-03	2.3E-02
K05352	ribitol-5-phosphate 2-dehydrogenase (NADP+) [EC:1.1.1.405]	0.92	0.86	0.97	4.6E-03	2.3E-02
K04114	benzoyl-CoA reductase subunit A [EC:1.3.7.8]	0.92	0.87	0.97	4.6E-03	2.3E-02
K04115	benzoyl-CoA reductase subunit D [EC:1.3.7.8]	0.92	0.87	0.97	4.6E-03	2.3E-02
K03059	DNA-directed RNA polymerase subunit P [EC:2.7.7.6]	0.92	0.86	0.97	4.6E-03	2.3E-02
K03852	sulfoacetaldehyde acetyltransferase [EC:2.3.3.15]	0.92	0.87	0.97	4.6E-03	2.3E-02
K06020	energy-dependent translational throttle protein EttA	0.92	0.87	0.97	4.6E-03	2.3E-02
K07583	tRNA pseudouridine synthase 10 [EC:5.4.99.25]	0.92	0.86	0.97	4.7E-03	2.3E-02
K16515	4-oxalomesaconate hydratase [EC:4.2.1.83]	0.92	0.87	0.97	4.7E-03	2.3E-02
K05350	beta-glucosidase [EC:3.2.1.21]	0.92	0.86	0.97	4.7E-03	2.3E-02
K05342	alpha,alpha-trehalose phosphorylase [EC:2.4.1.64]	0.92	0.86	0.97	4.7E-03	2.3E-02
K03418	N,N-dimethylformamidase large subunit [EC:3.5.1.56]	0.92	0.87	0.97	4.7E-03	2.3E-02
K12255	guanidinobutyrase [EC:3.5.3.7]	0.92	0.87	0.98	4.7E-03	2.3E-02
K09482	glutamyl-tRNA(Gln) amidotransferase subunit D [EC:6.3.5.7]	0.92	0.86	0.97	4.7E-03	2.3E-02
K16038	N-methyltransferase [EC:2.1.1.-]	0.91	0.86	0.97	4.8E-03	2.3E-02
K06970	23S rRNA (adenine1618-N6)-methyltransferase [EC:2.1.1.181]	1.09	1.03	1.16	4.8E-03	2.4E-02

Predictor	Description	HR	2.5% CI	97.5% CI	P	FDR-adjusted P
K03462	nicotinamide phosphoribosyltransferase [EC:2.4.2.12]	0.92	0.86	0.97	4.8E-03	2.4E-02
K02229	precorrin-3B synthase [EC:1.14.13.83]	0.92	0.87	0.97	4.8E-03	2.4E-02
K04109	4-hydroxybenzoyl-CoA reductase subunit beta [EC:1.1.7.1]	0.92	0.87	0.97	4.9E-03	2.4E-02
K04107	4-hydroxybenzoyl-CoA reductase subunit gamma [EC:1.1.7.1]	0.92	0.87	0.97	4.9E-03	2.4E-02
K01880	glycyl-tRNA synthetase [EC:6.1.1.14]	0.94	0.89	0.98	4.9E-03	2.4E-02
K13778	geranyl-CoA carboxylase beta subunit [EC:6.4.1.5]	0.92	0.87	0.97	4.9E-03	2.4E-02
K16150	glycogen synthase [EC:2.4.1.11]	0.92	0.87	0.98	5.0E-03	2.5E-02
K03648	uracil-DNA glycosylase [EC:3.2.2.27]	0.93	0.88	0.98	5.0E-03	2.5E-02
K00125	formate dehydrogenase (coenzyme F420) beta subunit [EC:1.17.98.3 1.8.98.6]	0.92	0.86	0.97	5.1E-03	2.5E-02
K04034	anaerobic magnesium-protoporphyrin IX monomethyl ester cyclase [EC:1.21.98.3]	0.92	0.87	0.97	5.1E-03	2.5E-02
K14267	N-succinyldiaminopimelate aminotransferase [EC:2.6.1.17]	0.92	0.87	0.97	5.1E-03	2.5E-02
K05715	2-phosphoglycerate kinase [EC:2.7.2.16]	0.92	0.86	0.97	5.2E-03	2.5E-02
K08077	UDP-sugar diphosphatase [EC:3.6.1.45]	0.92	0.87	0.98	5.2E-03	2.5E-02
K16647	arabinofuranan 3-O-arabinosyltransferase [EC:2.4.2.47]	0.92	0.87	0.98	5.2E-03	2.5E-02
K01865	(hydroxyamino)benzene mutase [EC:5.4.4.1]	0.92	0.87	0.98	5.3E-03	2.5E-02
K01829	disulfide reductase [EC:1.8.-.-]	0.92	0.87	0.98	5.3E-03	2.5E-02
K14727	3-oxoadipate enol-lactonase / 4-carboxymuconolactone decarboxylase [EC:3.1.1.24 4.1.1.44]	0.92	0.87	0.98	5.3E-03	2.5E-02
K13605	bacteriochlorophyll c synthase [EC:2.5.1.-]	0.92	0.87	0.98	5.3E-03	2.5E-02
K03725	archaea-specific helicase [EC:3.6.4.-]	0.92	0.87	0.98	5.3E-03	2.5E-02
K08252	receptor protein-tyrosine kinase [EC:2.7.10.1]	0.92	0.87	0.98	5.3E-03	2.5E-02
K03432	proteasome alpha subunit [EC:3.4.25.1]	0.92	0.86	0.97	5.3E-03	2.5E-02
K11646	3-dehydroquinate synthase II [EC:1.4.1.24]	0.92	0.86	0.97	5.4E-03	2.5E-02
K12234	coenzyme F420-0:L-glutamate ligase / coenzyme F420-1:gamma-L-glutamate ligase [EC:6.3.2.31 6.3.2.34]	0.92	0.86	0.97	5.4E-03	2.5E-02
K10221	2-pyrone-4,6-dicarboxylate lactonase [EC:3.1.1.57]	0.92	0.87	0.98	5.4E-03	2.5E-02
K11623	two-component system, NarL family, sensor histidine kinase YdfH [EC:2.7.13.3]	0.92	0.87	0.98	5.4E-03	2.5E-02
K13482	xanthine dehydrogenase large subunit [EC:1.17.1.4]	0.92	0.87	0.98	5.4E-03	2.5E-02
K01897	long-chain acyl-CoA synthetase [EC:6.2.1.3]	0.93	0.89	0.98	5.4E-03	2.6E-02
K13581	modification methylase [EC:2.1.1.72]	0.92	0.86	0.97	5.5E-03	2.6E-02
K10219	2-hydroxy-4-carboxymuconate semialdehyde hemiacetal dehydrogenase [EC:1.1.1.312]	0.92	0.87	0.98	5.5E-03	2.6E-02
K13540	precorrin-2 C20-methyltransferase / precorrin-3B C17-methyltransferase [EC:2.1.1.130 2.1.1.131]	0.92	0.87	0.98	5.5E-03	2.6E-02
K12349	neutral ceramidase [EC:3.5.1.23]	0.92	0.87	0.98	5.5E-03	2.6E-02
K00863	triose/dihydroxyacetone kinase / FAD-AMP lyase (cyclizing) [EC:2.7.1.28 2.7.1.29 4.6.1.15]	0.92	0.87	0.98	5.5E-03	2.6E-02
K00523	CDP-4-dehydro-6-deoxyglucose reductase, E3 [EC:1.17.1.1]	0.92	0.87	0.98	5.5E-03	2.6E-02
K00023	acetoacetyl-CoA reductase [EC:1.1.1.36]	0.92	0.87	0.98	5.5E-03	2.6E-02
K00508	linoleoyl-CoA desaturase [EC:1.14.19.3]	0.92	0.87	0.98	5.5E-03	2.6E-02
K15871	bile acid CoA-transferase [EC:2.8.3.25]	0.92	0.87	0.98	5.6E-03	2.6E-02
K15872	bile-acid 7alpha-dehydratase [EC:4.2.1.106]	0.92	0.87	0.98	5.6E-03	2.6E-02
K00993	ethanolaminephosphotransferase [EC:2.7.8.1]	0.92	0.86	0.97	5.6E-03	2.6E-02
K00477	phytanoyl-CoA hydroxylase [EC:1.14.11.18]	0.92	0.87	0.98	5.7E-03	2.6E-02
K01224	arabinogalactan endo-1,4-beta-galactosidase [EC:3.2.1.89]	0.92	0.86	0.98	5.7E-03	2.6E-02
K01622	fructose 1,6-bisphosphate aldolase/phosphatase [EC:4.1.2.13 3.1.3.11]	0.92	0.86	0.98	5.7E-03	2.6E-02
K00021	hydroxymethylglutaryl-CoA reductase (NADPH) [EC:1.1.1.34]	0.92	0.86	0.98	5.7E-03	2.6E-02
K02340	DNA polymerase III subunit delta [EC:2.7.7.7]	0.92	0.86	0.98	5.7E-03	2.6E-02
K16035	7-O-carbamoyltransferase [EC:2.1.3.-]	0.92	0.86	0.97	5.7E-03	2.6E-02

Predictor	Description	HR	2.5% CI	97.5% CI	P	FDR-adjusted P
K02322	DNA polymerase II large subunit [EC:2.7.7.7]	0.92	0.87	0.98	5.8E-03	2.7E-02
K16319	anthranilate 1,2-dioxygenase large subunit [EC:1.14.12.1]	0.92	0.87	0.98	5.8E-03	2.7E-02
K08258	staphopain A [EC:3.4.22.48]	0.92	0.86	0.98	5.8E-03	2.7E-02
K07540	benzylsuccinate synthase [EC:4.1.99.11]	0.92	0.87	0.98	5.8E-03	2.7E-02
K05898	3-oxosteroid 1-dehydrogenase [EC:1.3.99.4]	0.92	0.87	0.98	5.9E-03	2.7E-02
K05349	beta-glucosidase [EC:3.2.1.21]	0.93	0.88	0.98	6.0E-03	2.7E-02
K01299	carboxypeptidase Taq [EC:3.4.17.19]	0.92	0.86	0.98	6.0E-03	2.7E-02
K07151	dolichyl-diphosphooligosaccharide---protein glycosyltransferase [EC:2.4.99.18]	0.92	0.86	0.98	6.0E-03	2.7E-02
K10231	kojibiose phosphorylase [EC:2.4.1.230]	0.92	0.87	0.98	6.0E-03	2.7E-02
K00869	mevalonate kinase [EC:2.7.1.36]	0.92	0.87	0.98	6.0E-03	2.7E-02
K03056	DNA-directed RNA polymerase subunit L [EC:2.7.7.6]	0.92	0.87	0.98	6.1E-03	2.7E-02
K13603	3-vinyl bacteriochlorophyllide hydratase [EC:4.2.1.169]	0.92	0.87	0.98	6.1E-03	2.7E-02
K03170	reverse gyrase [EC:5.6.2.2 3.6.4.12]	0.92	0.87	0.98	6.1E-03	2.7E-02
K11444	two-component system, chemotaxis family, response regulator WspR [EC:2.7.7.65]	0.92	0.87	0.98	6.1E-03	2.8E-02
K03863	vanillate monooxygenase ferredoxin subunit	0.92	0.87	0.98	6.3E-03	2.8E-02
K00499	choline monooxygenase [EC:1.14.15.7]	0.92	0.87	0.98	6.3E-03	2.8E-02
K00662	aminoglycoside 3-N-acetyltransferase [EC:2.3.1.81]	0.92	0.87	0.98	6.4E-03	2.8E-02
K13601	bacteriochlorophyllide d C-8(2)-methyltransferase [EC:2.1.1.332]	0.92	0.87	0.98	6.4E-03	2.8E-02
K05343	maltose alpha-D-glucosyltransferase / alpha-amylase [EC:5.4.99.16 3.2.1.1]	0.92	0.87	0.98	6.4E-03	2.8E-02
K01075	4-hydroxybenzoyl-CoA thioesterase [EC:3.1.2.23]	0.92	0.87	0.98	6.4E-03	2.8E-02
K01179	endoglucanase [EC:3.2.1.4]	0.92	0.87	0.98	6.4E-03	2.9E-02
K05549	benzoate/toluate 1,2-dioxygenase subunit alpha [EC:1.14.12.10 1.14.12.-]	0.92	0.87	0.98	6.4E-03	2.9E-02
K08068	UDP-N-acetylglucosamine 2-epimerase (hydrolysing) [EC:3.2.1.183]	0.92	0.87	0.98	6.4E-03	2.9E-02
K00449	protocatechuate 3,4-dioxygenase, beta subunit [EC:1.13.11.3]	0.92	0.87	0.98	6.5E-03	2.9E-02
K07518	hydroxybutyrate-dimer hydrolase [EC:3.1.1.22]	0.92	0.87	0.98	6.5E-03	2.9E-02
K15514	3,4-dehydroadipyl-CoA semialdehyde dehydrogenase [EC:1.2.1.77]	0.92	0.87	0.98	6.5E-03	2.9E-02
K10796	D-proline reductase (dithiol)-stabilizing protein PrdE	0.93	0.87	0.98	6.6E-03	2.9E-02
K12254	4-guanidinobutyraldehyde dehydrogenase / NAD-dependent aldehyde dehydrogenase [EC:1.2.1.54 1.2.1.-]	0.92	0.87	0.98	6.6E-03	2.9E-02
K11387	arabinosyltransferase C [EC:2.4.2.-]	0.92	0.87	0.98	6.7E-03	2.9E-02
K13831	3-hexulose-6-phosphate synthase / 6-phospho-3-hexuloisomerase [EC:4.1.2.43 5.3.1.27]	0.92	0.87	0.98	6.7E-03	2.9E-02
K05884	L-2-hydroxycarboxylate dehydrogenase (NAD+) [EC:1.1.1.337]	0.92	0.87	0.98	6.7E-03	3.0E-02
K01777	proline racemase [EC:5.1.1.4]	0.92	0.87	0.98	6.8E-03	3.0E-02
K01233	chitosanase [EC:3.2.1.132]	0.92	0.87	0.98	6.8E-03	3.0E-02
K15525	N-acetyl-1-D-myo-inositol-2-amino-2-deoxy-alpha-D-glucopyranoside deacetylase [EC:3.5.1.103]	0.92	0.87	0.98	6.8E-03	3.0E-02
K00425	cytochrome bd ubiquinol oxidase subunit I [EC:7.1.1.7]	1.10	1.03	1.18	6.8E-03	3.0E-02
K07538	6-hydroxycyclohex-1-ene-1-carbonyl-CoA dehydrogenase [EC:1.1.1.368]	0.92	0.87	0.98	6.8E-03	3.0E-02
K00045	mannitol 2-dehydrogenase [EC:1.1.1.67]	0.92	0.87	0.98	6.8E-03	3.0E-02
K01705	homoaconitate hydratase [EC:4.2.1.36]	0.92	0.87	0.98	6.9E-03	3.0E-02
K10710	fructoselysine 6-kinase [EC:2.7.1.218]	0.92	0.87	0.98	7.0E-03	3.0E-02
K15669	D-glycero-alpha-D-manno-heptose 1-phosphate guanylyltransferase [EC:2.7.7.71]	0.92	0.87	0.98	7.0E-03	3.0E-02
K01273	membrane dipeptidase [EC:3.4.13.19]	0.92	0.87	0.98	7.1E-03	3.1E-02
K01318	glutamyl endopeptidase [EC:3.4.21.19]	0.92	0.87	0.98	7.2E-03	3.1E-02
K14653	2-amino-5-formylamino-6-ribosylaminopyrimidin-4(3H)-one 5'-monophosphate deformylase [EC:3.5.1.102]	0.92	0.87	0.98	7.2E-03	3.1E-02
K00437	[NiFe] hydrogenase large subunit [EC:1.12.2.1]	0.92	0.87	0.98	7.3E-03	3.1E-02

Predictor	Description	HR	2.5% CI	97.5% CI	P	FDR-adjusted P
K05873	adenylate cyclase, class 2 [EC:4.6.1.1]	0.92	0.87	0.98	7.3E-03	3.2E-02
K00534	ferredoxin hydrogenase small subunit [EC:1.12.7.2]	0.92	0.87	0.98	7.3E-03	3.2E-02
K13491	two-component system, chemotaxis family, response regulator WspF [EC:3.1.1.61]	0.93	0.87	0.98	7.4E-03	3.2E-02
K01060	cephalosporin-C deacetylase [EC:3.1.1.41]	0.92	0.87	0.98	7.4E-03	3.2E-02
K00082	5-amino-6-(5-phosphoribosylamino)uracil reductase [EC:1.1.1.193]	0.92	0.87	0.98	7.4E-03	3.2E-02
K00633	galactoside O-acetyltransferase [EC:2.3.1.18]	1.08	1.02	1.15	7.6E-03	3.2E-02
K01655	homocitrate synthase [EC:2.3.3.14]	0.93	0.88	0.98	7.8E-03	3.3E-02
K16317	tRNA (pseudouridine54-N1)-methyltransferase [EC:2.1.1.257]	0.92	0.87	0.98	7.9E-03	3.4E-02
K00839	(S)-ureidoglycine---glyoxylate transaminase [EC:2.6.1.112]	0.92	0.87	0.98	8.0E-03	3.4E-02
K03653	N-glycosylase/DNA lyase [EC:3.2.2.- 4.2.99.18]	0.92	0.87	0.98	8.1E-03	3.4E-02
K03049	DNA-directed RNA polymerase subunit E' [EC:2.7.7.6]	0.92	0.87	0.98	8.1E-03	3.4E-02
K00002	alcohol dehydrogenase (NADP+) [EC:1.1.1.2]	0.92	0.87	0.98	8.1E-03	3.4E-02
K01502	aliphatic nitrilase [EC:3.5.5.7]	0.92	0.87	0.98	8.1E-03	3.4E-02
K04093	chorismate mutase [EC:5.4.99.5]	0.92	0.87	0.98	8.1E-03	3.4E-02
K13777	geranyl-CoA carboxylase alpha subunit [EC:6.4.1.5]	0.92	0.87	0.98	8.1E-03	3.4E-02
K11711	two-component system, LuxR family, sensor histidine kinase DctS [EC:2.7.13.3]	0.93	0.87	0.98	8.2E-03	3.4E-02
K14519	NADP-dependent aldehyde dehydrogenase [EC:1.2.1.4]	0.92	0.87	0.98	8.2E-03	3.4E-02
K09846	demethylspheroidene O-methyltransferase [EC:2.1.1.210]	0.93	0.87	0.98	8.3E-03	3.5E-02
K16218	2-methylisoborneol synthase [EC:4.2.3.118]	0.93	0.88	0.98	8.4E-03	3.5E-02
K08651	thermitase [EC:3.4.21.66]	0.92	0.87	0.98	8.5E-03	3.5E-02
K13522	bifunctional NMN adenylyltransferase/nudix hydrolase [EC:2.7.7.1 3.6.1.-]	0.93	0.87	0.98	8.5E-03	3.6E-02
K15531	oligosaccharide reducing-end xylanase [EC:3.2.1.156]	0.92	0.87	0.98	8.5E-03	3.6E-02
K00140	malonate-semialdehyde dehydrogenase (acetylating) / methylmalonate-semialdehyde dehydrogenase [EC:1.2.1.18 1.2.1.27]	0.92	0.87	0.98	8.8E-03	3.7E-02
K08961	chondroitin-sulfate-ABC endolyase/exolyase [EC:4.2.2.20 4.2.2.21]	0.93	0.87	0.98	8.8E-03	3.7E-02
K12453	CDP-paratose synthetase [EC:1.1.1.342]	0.93	0.87	0.98	8.9E-03	3.7E-02
K01533	P-type Cu2+ transporter [EC:7.2.2.9]	0.93	0.89	0.98	9.0E-03	3.7E-02
K00376	nitrous-oxide reductase [EC:1.7.2.4]	0.92	0.87	0.98	9.0E-03	3.7E-02
K01959	pyruvate carboxylase subunit A [EC:6.4.1.1]	0.92	0.87	0.98	9.0E-03	3.7E-02
K00752	hyaluronan synthase [EC:2.4.1.212]	0.93	0.88	0.98	9.2E-03	3.8E-02
K00428	cytochrome c peroxidase [EC:1.11.1.5]	1.08	1.02	1.15	9.2E-03	3.8E-02
K00700	1,4-alpha-glucan branching enzyme [EC:2.4.1.18]	0.93	0.88	0.98	9.2E-03	3.8E-02
K16049	3,4-dihydroxy-9,10-secoandrosta-1,3,5(10)-triene-9,17-dione 4,5-dioxygenase [EC:1.13.11.25]	0.93	0.88	0.98	9.2E-03	3.8E-02
K11383	two-component system, NtrC family, sensor histidine kinase KinB [EC:2.7.13.3]	0.93	0.88	0.98	9.3E-03	3.8E-02
K00596	2,2-dialkylglycine decarboxylase (pyruvate) [EC:4.1.1.64]	0.93	0.88	0.98	9.3E-03	3.8E-02
K12992	O-antigen biosynthesis alpha-1,3-rhamnosyltransferase [EC:2.4.1.377]	0.92	0.87	0.98	9.3E-03	3.8E-02
K03794	sirohydrochlorin ferrochelatase [EC:4.99.1.4]	0.93	0.87	0.98	9.3E-03	3.8E-02
K01001	UDP-N-acetylglucosamine--dolichyl-phosphate N-acetylglucosaminephosphotransferase [EC:2.7.8.15]	0.92	0.87	0.98	9.4E-03	3.9E-02
K01896	medium-chain acyl-CoA synthetase [EC:6.2.1.2]	0.93	0.88	0.98	9.5E-03	3.9E-02
K00696	sucrose-phosphate synthase [EC:2.4.1.14]	0.93	0.87	0.98	9.5E-03	3.9E-02
K06720	L-ectoine synthase [EC:4.2.1.108]	0.93	0.87	0.98	9.5E-03	3.9E-02
K03851	taurine-pyruvate aminotransferase [EC:2.6.1.77]	0.93	0.88	0.98	9.7E-03	3.9E-02
K15510	coenzyme F420-dependent glucose-6-phosphate dehydrogenase [EC:1.1.98.2]	0.93	0.88	0.98	9.9E-03	4.0E-02
K07116	acyl-homoserine-lactone acylase [EC:3.5.1.97]	0.93	0.87	0.98	1.0E-02	4.0E-02
K00426	cytochrome bd ubiquinol oxidase subunit II [EC:7.1.1.7]	1.10	1.02	1.18	1.0E-02	4.1E-02

Predictor	Description	HR	2.5% CI	97.5% CI	P	FDR-adjusted P
K01505	1-aminocyclopropane-1-carboxylate deaminase [EC:3.5.99.7]	0.93	0.87	0.98	1.0E-02	4.1E-02
K03058	DNA-directed RNA polymerase subunit N [EC:2.7.7.6]	0.92	0.87	0.98	1.0E-02	4.1E-02
K08080	CMP-N-acetylneuraminate monooxygenase [EC:1.14.18.2]	0.90	0.82	0.97	1.0E-02	4.1E-02
K14733	limonene 1,2-monooxygenase [EC:1.14.13.107]	0.93	0.88	0.98	1.0E-02	4.1E-02
K00764	amidophosphoribosyltransferase [EC:2.4.2.14]	0.93	0.89	0.98	1.0E-02	4.1E-02
K06607	myo-inositol catabolism protein IolS [EC:1.1.1.-]	0.92	0.87	0.98	1.0E-02	4.1E-02
K15754	2'-aminobiphenyl-2,3-diol 1,2-dioxygenase, small subunit [EC:1.13.11.-]	0.93	0.87	0.98	1.0E-02	4.1E-02
K00198	anaerobic carbon-monoxide dehydrogenase catalytic subunit [EC:1.2.7.4]	0.92	0.87	0.98	1.0E-02	4.1E-02
K01219	beta-agarase [EC:3.2.1.81]	0.93	0.87	0.98	1.0E-02	4.2E-02
K16165	fumarylpyruvate hydrolase [EC:3.7.1.20]	0.93	0.87	0.98	1.0E-02	4.2E-02
K01641	hydroxymethylglutaryl-CoA synthase [EC:2.3.3.10]	0.92	0.87	0.98	1.1E-02	4.3E-02
K10253	DOPA 4,5-dioxygenase [EC:1.14.99.-]	0.93	0.88	0.98	1.1E-02	4.3E-02
K12420	ketoreductase [EC:1.1.1.-]	0.93	0.88	0.98	1.1E-02	4.3E-02
K05824	homoisocitrate dehydrogenase [EC:1.1.1.87]	0.93	0.88	0.98	1.1E-02	4.3E-02
K03652	DNA-3-methyladenine glycosylase [EC:3.2.2.21]	0.92	0.87	0.98	1.1E-02	4.3E-02
K05996	carboxypeptidase T [EC:3.4.17.18]	0.93	0.88	0.98	1.1E-02	4.3E-02
K04112	benzoyl-CoA reductase subunit C [EC:1.3.7.8]	0.93	0.87	0.98	1.1E-02	4.3E-02
K02473	UDP-N-acetylglucosamine/UDP-N-acetylgalactosamine 4-epimerase [EC:5.1.3.7 5.1.3.-]	0.93	0.88	0.98	1.1E-02	4.3E-02
K16164	acylpyruvate hydrolase [EC:3.7.1.5]	0.93	0.88	0.98	1.1E-02	4.3E-02
K05828	[amino group carrier protein]-L-2-aminoadipate 6-kinase [EC:2.7.2.17]	0.93	0.88	0.98	1.1E-02	4.3E-02
K04113	benzoyl-CoA reductase subunit B [EC:1.3.7.8]	0.93	0.87	0.98	1.1E-02	4.3E-02
K01904	4-coumarate--CoA ligase [EC:6.2.1.12]	0.92	0.87	0.98	1.1E-02	4.4E-02
K10774	tyrosine ammonia-lyase [EC:4.3.1.23]	0.92	0.87	0.98	1.1E-02	4.4E-02
K14337	alpha-1,6-mannosyltransferase [EC:2.4.1.-]	0.93	0.88	0.98	1.2E-02	4.6E-02
K05341	amylosucrase [EC:2.4.1.4]	0.93	0.87	0.98	1.2E-02	4.6E-02
K03382	hydroxydechloroatrazine ethylaminohydrolase [EC:3.5.4.43]	0.93	0.87	0.98	1.2E-02	4.6E-02
K01053	gluconolactonase [EC:3.1.1.17]	0.93	0.87	0.98	1.2E-02	4.6E-02
K00266	glutamate synthase (NADPH) small chain [EC:1.4.1.13]	0.93	0.89	0.99	1.2E-02	4.7E-02
K13500	chondroitin synthase [EC:2.4.1.175 2.4.1.226]	0.93	0.87	0.98	1.2E-02	4.7E-02
K15982	3-ketosteroid 9alpha-monooxygenase subunit A [EC:1.14.15.30]	0.93	0.88	0.98	1.2E-02	4.7E-02
K00836	diaminobutyrate-2-oxoglutarate transaminase [EC:2.6.1.76]	0.93	0.87	0.98	1.2E-02	4.7E-02
K00466	tryptophan 2-monooxygenase [EC:1.13.12.3]	0.93	0.88	0.98	1.2E-02	4.7E-02
K00433	non-heme chloroperoxidase [EC:1.11.1.10]	0.92	0.87	0.98	1.2E-02	4.7E-02
K01593	aromatic-L-amino-acid/L-tryptophan decarboxylase [EC:4.1.1.28 4.1.1.105]	0.93	0.88	0.98	1.2E-02	4.8E-02
K15792	MurE/MurF fusion protein [EC:6.3.2.13 6.3.2.10]	0.93	0.88	0.98	1.2E-02	4.8E-02
K06718	L-2,4-diaminobutyric acid acetyltransferase [EC:2.3.1.178]	0.93	0.88	0.98	1.3E-02	4.8E-02
K07546	E-phenylitaconyl-CoA hydratase [EC:4.2.1.-]	0.93	0.88	0.98	1.3E-02	4.8E-02
K16039	N-glycosyltransferase [EC:2.4.1.-]	0.93	0.87	0.98	1.3E-02	4.9E-02
K07284	sortase A [EC:3.4.22.70]	0.93	0.87	0.98	1.3E-02	4.9E-02
K00820	glutamine---fructose-6-phosphate transaminase (isomerizing) [EC:2.6.1.16]	0.93	0.89	0.99	1.3E-02	4.9E-02
K01014	aryl sulfotransferase [EC:2.8.2.1]	0.93	0.88	0.98	1.3E-02	5.0E-02
K07544	benzylsuccinate CoA-transferase BbsF subunit [EC:2.8.3.15]	0.93	0.88	0.98	1.3E-02	5.0E-02
K00315	dimethylglycine dehydrogenase [EC:1.5.8.4]	0.93	0.87	0.98	1.3E-02	5.0E-02
K01227	mannosyl-glycoprotein endo-beta-N-acetylglucosaminidase [EC:3.2.1.96]	0.93	0.87	0.98	1.3E-02	5.0E-02

Predictor	Description	HR	2.5% CI	97.5% CI	P	FDR-adjusted P
K01735	3-dehydroquinate synthase [EC:4.2.3.4]	1.08	1.02	1.15	1.3E-02	5.1E-02
K12252	arginine:pyruvate transaminase [EC:2.6.1.84]	0.93	0.88	0.99	1.3E-02	5.1E-02
K13659	2-beta-glucuronyltransferase [EC:2.4.1.264]	0.93	0.88	0.99	1.4E-02	5.2E-02
K01591	orotidine-5'-phosphate decarboxylase [EC:4.1.1.23]	0.93	0.87	0.98	1.4E-02	5.2E-02
K00849	galactokinase [EC:2.7.1.6]	0.93	0.88	0.99	1.4E-02	5.2E-02
K13020	UDP-N-acetyl-2-amino-2-deoxyglucuronate dehydrogenase [EC:1.1.1.335]	0.93	0.88	0.99	1.4E-02	5.2E-02
K06949	ribosome biogenesis GTPase / thiamine phosphate phosphatase [EC:3.6.1.- 3.1.3.100]	1.09	1.02	1.16	1.4E-02	5.2E-02
K10622	HCOMODA/2-hydroxy-3-carboxy-muconic semialdehyde decarboxylase [EC:4.1.1.-]	0.93	0.88	0.99	1.4E-02	5.2E-02
K16178	dimethylamine---corrinoid protein Co-methyltransferase [EC:2.1.1.249]	0.93	0.87	0.98	1.4E-02	5.3E-02
K03737	pyruvate-ferredoxin/flavodoxin oxidoreductase [EC:1.2.7.1 1.2.7.-]	1.09	1.02	1.17	1.4E-02	5.3E-02
K01032	3-oxoadipate CoA-transferase, beta subunit [EC:2.8.3.6]	0.93	0.88	0.99	1.4E-02	5.4E-02
K00004	(R,R)-butanediol dehydrogenase / meso-butanediol dehydrogenase / diacetyl reductase [EC:1.1.1.4 1.1.1.- 1.1.1.303]	0.93	0.87	0.99	1.4E-02	5.4E-02
K00854	xylulokinase [EC:2.7.1.17]	0.94	0.89	0.99	1.5E-02	5.5E-02
K08096	GTP cyclohydrolase IIa [EC:3.5.4.29]	0.93	0.87	0.99	1.5E-02	5.5E-02
K14627	dehydratase [EC:4.2.1.-]	0.93	0.88	0.99	1.5E-02	5.5E-02
K15862	cytochrome c oxidase cbb3-type subunit I/II [EC:7.1.1.9]	0.93	0.88	0.99	1.5E-02	5.6E-02
K05979	2-phosphosulfolactate phosphatase [EC:3.1.3.71]	0.93	0.87	0.99	1.5E-02	5.6E-02
K01868	threonyl-tRNA synthetase [EC:6.1.1.3]	1.09	1.02	1.16	1.5E-02	5.6E-02
K01601	ribulose-bisphosphate carboxylase large chain [EC:4.1.1.39]	0.93	0.87	0.99	1.5E-02	5.7E-02
K01733	threonine synthase [EC:4.2.3.1]	0.94	0.89	0.99	1.5E-02	5.7E-02
K02548	1,4-dihydroxy-2-naphthoate polyprenyltransferase [EC:2.5.1.74]	1.09	1.02	1.17	1.5E-02	5.8E-02
K07533	foldase protein PrsA [EC:5.2.1.8]	0.93	0.87	0.99	1.6E-02	5.8E-02
K03416	methylmalonyl-CoA carboxyltransferase 5S subunit [EC:2.1.3.1]	0.93	0.88	0.99	1.6E-02	5.8E-02
K01928	UDP-N-acetylmuramoyl-L-alanyl-D-glutamate--2,6-diaminopimelate ligase [EC:6.3.2.13]	0.93	0.87	0.99	1.6E-02	5.8E-02
K09693	teichoic acid transport system ATP-binding protein [EC:7.5.2.4]	0.93	0.87	0.99	1.6E-02	5.9E-02
K00131	glyceraldehyde-3-phosphate dehydrogenase (NADP+) [EC:1.2.1.9]	0.93	0.87	0.99	1.6E-02	5.9E-02
K00875	D-ribulokinase [EC:2.7.1.47]	0.93	0.88	0.99	1.6E-02	5.9E-02
K02293	15-cis-phytoene desaturase [EC:1.3.5.5]	0.93	0.88	0.99	1.6E-02	5.9E-02
K05991	endoglycosylceramidase [EC:3.2.1.123]	0.93	0.88	0.99	1.6E-02	5.9E-02
K01822	steroid Delta-isomerase [EC:5.3.3.1]	0.93	0.88	0.99	1.6E-02	5.9E-02
K04105	4-hydroxybenzoate-CoA ligase [EC:6.2.1.27 6.2.1.25]	0.93	0.88	0.99	1.6E-02	5.9E-02
K14681	NA	0.93	0.88	0.99	1.6E-02	6.0E-02
K07650	two-component system, OmpR family, sensor histidine kinase CssS [EC:2.7.13.3]	0.93	0.87	0.99	1.7E-02	6.1E-02
K01574	acetoacetate decarboxylase [EC:4.1.1.4]	0.93	0.88	0.99	1.7E-02	6.2E-02
K14940	gamma-F420-2:alpha-L-glutamate ligase [EC:6.3.2.32]	0.93	0.88	0.99	1.7E-02	6.2E-02
K06863	5-formaminoimidazole-4-carboxamide-1-(beta)-D-ribofuranosyl 5'-monophosphate synthetase [EC:6.3.4.23]	0.93	0.88	0.99	1.7E-02	6.2E-02
K11434	type I protein arginine methyltransferase [EC:2.1.1.319]	0.93	0.88	0.99	1.7E-02	6.2E-02
K03404	magnesium chelatase subunit D [EC:6.6.1.1]	0.93	0.87	0.99	1.7E-02	6.2E-02
K05716	cyclic 2,3-diphosphoglycerate synthase [EC:6.5.1.9]	0.93	0.88	0.99	1.7E-02	6.2E-02
K01799	maleate isomerase [EC:5.2.1.1]	0.93	0.88	0.99	1.7E-02	6.2E-02
K07549	benzoylsuccinyl-CoA thiolase BbsA subunit [EC:2.3.1.-]	0.93	0.88	0.99	1.7E-02	6.3E-02
K07550	benzoylsuccinyl-CoA thiolase BbsB subunit [EC:2.3.1.-]	0.93	0.88	0.99	1.7E-02	6.3E-02
K01736	chorismate synthase [EC:4.2.3.5]	0.93	0.87	0.99	1.7E-02	6.3E-02
K13686	galactan 5-O-arabinofuranosyltransferase [EC:2.4.2.46]	0.93	0.88	0.99	1.7E-02	6.3E-02

Predictor	Description	HR	2.5% CI	97.5% CI	P	FDR-adjusted P
K07548	2-[hydroxy(phenyl)methyl]-succinyl-CoA dehydrogenase BbsD subunit [EC:1.1.1.35]	0.93	0.88	0.99	1.7E-02	6.3E-02
K10811	thiamine pyridinylase [EC:2.5.1.2]	0.93	0.88	0.99	1.7E-02	6.3E-02
K00986	RNA-directed DNA polymerase [EC:2.7.7.49]	0.94	0.89	0.99	1.7E-02	6.3E-02
K03380	phenol 2-monooxygenase (NADPH) [EC:1.14.13.7]	0.93	0.88	0.99	1.7E-02	6.3E-02
K13380	NADH-quinone oxidoreductase subunit B/C/D [EC:7.1.1.2]	0.93	0.88	0.99	1.7E-02	6.3E-02
K15532	unsaturated rhamnogalacturonyl hydrolase [EC:3.2.1.172]	0.94	0.89	0.99	1.8E-02	6.3E-02
K01909	long-chain-fatty-acid--[acyl-carrier-protein] ligase [EC:6.2.1.20]	0.93	0.88	0.99	1.8E-02	6.4E-02
K13745	L-2,4-diaminobutyrate decarboxylase [EC:4.1.1.86]	0.93	0.88	0.99	1.8E-02	6.4E-02
K08644	tentoxilysin [EC:3.4.24.68]	0.93	0.88	0.99	1.8E-02	6.6E-02
K00271	valine dehydrogenase (NAD+) [EC:1.4.1.23]	0.93	0.88	0.99	1.8E-02	6.6E-02
K01851	salicylate biosynthesis isochorismate synthase [EC:5.4.4.2]	0.93	0.88	0.99	1.8E-02	6.6E-02
K14667	minimal PKS ketosynthase (KS/KS alpha) [EC:2.3.1.-]	0.93	0.88	0.99	1.8E-02	6.6E-02
K15228	methylamine dehydrogenase light chain [EC:1.4.9.1]	0.93	0.88	0.99	1.9E-02	6.6E-02
K01488	adenosine deaminase [EC:3.5.4.4]	1.07	1.01	1.14	1.9E-02	6.6E-02
K06941	23S rRNA (adenine2503-C2)-methyltransferase [EC:2.1.1.192]	0.94	0.88	0.99	1.9E-02	6.7E-02
K03385	nitrite reductase (cytochrome c-552) [EC:1.7.2.2]	1.07	1.01	1.14	1.9E-02	6.7E-02
K01565	N-sulfoglucosamine sulfohydrolase [EC:3.10.1.1]	0.93	0.88	0.99	1.9E-02	6.7E-02
K07587	O-phosphoseryl-tRNA synthetase [EC:6.1.1.27]	0.93	0.88	0.99	1.9E-02	6.7E-02
K01031	3-oxoadipate CoA-transferase, alpha subunit [EC:2.8.3.6]	0.93	0.88	0.99	1.9E-02	6.7E-02
K05303	O-methyltransferase [EC:2.1.1.-]	0.93	0.88	0.99	1.9E-02	6.7E-02
K13779	isohexenylglutaconyl-CoA hydratase [EC:4.2.1.57]	0.93	0.88	0.99	1.9E-02	6.8E-02
K03602	exodeoxyribonuclease VII small subunit [EC:3.1.11.6]	0.93	0.88	0.99	1.9E-02	6.8E-02
K13930	triphosphoribosyl-dephospho-CoA synthase [EC:2.4.2.52]	0.93	0.88	0.99	1.9E-02	6.8E-02
K03581	exodeoxyribonuclease V alpha subunit [EC:3.1.11.5]	0.94	0.89	0.99	2.0E-02	6.9E-02
K02122	V/A-type H+/Na+-transporting ATPase subunit F	0.93	0.88	0.99	2.0E-02	7.0E-02
K03055	DNA-directed RNA polymerase subunit K [EC:2.7.7.6]	0.93	0.88	0.99	2.0E-02	7.0E-02
K01182	oligo-1,6-glucosidase [EC:3.2.1.10]	0.93	0.88	0.99	2.0E-02	7.0E-02
K05552	minimal PKS chain-length factor (CLF/KS beta) [EC:2.3.1.- 2.3.1.260 2.3.1.235]	0.93	0.88	0.99	2.0E-02	7.0E-02
K14680	RNA ligase [EC:6.5.1.3]	0.93	0.88	0.99	2.0E-02	7.1E-02
K14333	2,3-dihydroxybenzoate decarboxylase [EC:4.1.1.46]	0.93	0.88	0.99	2.0E-02	7.2E-02
K15538	glycoprotein endo-alpha-1,2-mannosidase [EC:3.2.1.130]	0.93	0.88	0.99	2.1E-02	7.2E-02
K13065	shikimate O-hydroxycinnamoyltransferase [EC:2.3.1.133]	0.93	0.88	0.99	2.1E-02	7.2E-02
K01406	serralysin [EC:3.4.24.40]	0.93	0.88	0.99	2.1E-02	7.3E-02
K01801	maleylpyruvate isomerase [EC:5.2.1.4]	0.93	0.88	0.99	2.1E-02	7.3E-02
K01838	beta-phosphoglucomutase [EC:5.4.2.6]	0.93	0.88	0.99	2.1E-02	7.3E-02
K01785	aldose 1-epimerase [EC:5.1.3.3]	0.94	0.89	0.99	2.1E-02	7.3E-02
K00297	methylenetetrahydrofolate reductase (NADH) [EC:1.5.1.54]	0.94	0.89	0.99	2.1E-02	7.3E-02
K03342	para-aminobenzoate synthetase / 4-amino-4-deoxychorismate lyase [EC:2.6.1.85 4.1.3.38]	0.93	0.88	0.99	2.1E-02	7.3E-02
K07545	(R)-benzylsuccinyl-CoA dehydrogenase [EC:1.3.8.3]	0.93	0.88	0.99	2.1E-02	7.4E-02
K00169	pyruvate ferredoxin oxidoreductase alpha subunit [EC:1.2.7.1]	0.93	0.88	0.99	2.1E-02	7.4E-02
K13028	aldoxime dehydratase [EC:4.99.1.5]	0.93	0.88	0.99	2.2E-02	7.5E-02
K00480	salicylate hydroxylase [EC:1.14.13.1]	0.93	0.88	0.99	2.2E-02	7.5E-02
K14215	trans,polycis-decaprenyl diphosphate synthase [EC:2.5.1.86]	0.93	0.88	0.99	2.2E-02	7.5E-02
K01721	nitrile hydratase subunit alpha [EC:4.2.1.84]	0.94	0.88	0.99	2.2E-02	7.6E-02

Predictor	Description	HR	2.5% CI	97.5% CI	P	FDR-adjusted P
K14335	alpha-1,6-mannosyltransferase [EC:2.4.1.-]	0.94	0.88	0.99	2.2E-02	7.6E-02
K13677	1,2-diacylglycerol-3-alpha-glucose alpha-1,2-glucosyltransferase [EC:2.4.1.208]	0.93	0.88	0.99	2.2E-02	7.7E-02
K07652	two-component system, OmpR family, sensor histidine kinase VicK [EC:2.7.13.3]	0.93	0.88	0.99	2.2E-02	7.7E-02
K06868	Sep-tRNA:Cys-tRNA synthetase [EC:2.5.1.73]	0.94	0.88	0.99	2.2E-02	7.7E-02
K01214	isoamylase [EC:3.2.1.68]	0.93	0.88	0.99	2.2E-02	7.7E-02
K15751	carbazole 1,9a-dioxygenase [EC:1.14.12.22]	0.93	0.88	0.99	2.2E-02	7.7E-02
K07543	benzylsuccinate CoA-transferase BbsE subunit [EC:2.8.3.15]	0.93	0.88	0.99	2.3E-02	7.7E-02
K13657	alpha-1,3-mannosyltransferase [EC:2.4.1.252]	0.93	0.88	0.99	2.3E-02	7.7E-02
K15229	methylamine dehydrogenase heavy chain [EC:1.4.9.1]	0.94	0.88	0.99	2.3E-02	7.7E-02
K00635	diacylglycerol O-acyltransferase / wax synthase [EC:2.3.1.20 2.3.1.75]	0.93	0.88	0.99	2.3E-02	7.8E-02
K04099	gallate dioxygenase [EC:1.13.11.57]	0.93	0.88	0.99	2.3E-02	7.8E-02
K05999	xanthomonalisin [EC:3.4.21.101]	0.94	0.88	0.99	2.3E-02	7.8E-02
K00471	gamma-butyrobetaine dioxygenase [EC:1.14.11.1]	0.94	0.88	0.99	2.3E-02	7.9E-02
K15913	UDP-N-acetylbacillosamine N-acetyltransferase [EC:2.3.1.203]	0.93	0.88	0.99	2.3E-02	7.9E-02
K16163	maleylpyruvate isomerase [EC:5.2.1.4]	0.94	0.88	0.99	2.4E-02	8.0E-02
K04712	sphingolipid 4-desaturase/C4-monooxygenase [EC:1.14.19.17 1.14.18.5]	0.94	0.89	0.99	2.4E-02	8.0E-02
K07649	two-component system, OmpR family, sensor histidine kinase TctE [EC:2.7.13.3]	0.93	0.88	0.99	2.4E-02	8.0E-02
K02189	cobalt-precorrin 5A hydrolase [EC:3.7.1.12]	0.93	0.88	0.99	2.4E-02	8.1E-02
K00188	2-oxoisovalerate ferredoxin oxidoreductase delta subunit [EC:1.2.7.7]	0.93	0.88	0.99	2.4E-02	8.1E-02
K03469	ribonuclease HI [EC:3.1.26.4]	0.94	0.89	0.99	2.4E-02	8.1E-02
K02123	V/A-type H+/Na+-transporting ATPase subunit I	0.94	0.89	0.99	2.4E-02	8.1E-02
K00316	spermidine dehydrogenase [EC:1.5.99.6]	0.94	0.89	0.99	2.4E-02	8.1E-02
K10713	5,6,7,8-tetrahydromethanopterin hydro-lyase [EC:4.2.1.147]	0.94	0.88	0.99	2.4E-02	8.1E-02
K05822	tetrahydrodipicolinate N-acetyltransferase [EC:2.3.1.89]	0.93	0.88	0.99	2.4E-02	8.2E-02
K11637	two-component system, CitB family, sensor histidine kinase CitS [EC:2.7.13.3]	0.94	0.88	0.99	2.4E-02	8.2E-02
K01835	phosphoglucomutase [EC:5.4.2.2]	0.94	0.89	0.99	2.5E-02	8.2E-02
K03343	putrescine oxidase [EC:1.4.3.10]	0.94	0.89	0.99	2.5E-02	8.2E-02
K12977	lipid A 1-phosphatase [EC:3.1.3.-]	0.93	0.87	0.99	2.5E-02	8.2E-02
K08070	2-alkenal reductase [EC:1.3.1.74]	0.93	0.88	0.99	2.5E-02	8.2E-02
K09516	all-trans-retinol 13,14-reductase [EC:1.3.99.23]	0.94	0.88	0.99	2.5E-02	8.3E-02
K00798	cob(I)alamin adenosyltransferase [EC:2.5.1.17]	0.94	0.89	0.99	2.5E-02	8.5E-02
K03101	signal peptidase II [EC:3.4.23.36]	0.93	0.88	0.99	2.5E-02	8.5E-02
K05934	precorrin-3B C17-methyltransferase / cobalt-factor III methyltransferase [EC:2.1.1.131 2.1.1.272]	0.93	0.88	0.99	2.6E-02	8.5E-02
K07260	zinc D-Ala-D-Ala carboxypeptidase [EC:3.4.17.14]	0.93	0.88	0.99	2.6E-02	8.5E-02
K00841	aminotransferase [EC:2.6.1.-]	0.93	0.88	0.99	2.6E-02	8.5E-02
K15983	3-ketosteroid 9alpha-monooxygenase subunit B [EC:1.14.15.30]	0.94	0.88	0.99	2.6E-02	8.5E-02
K01176	alpha-amylase [EC:3.2.1.1]	1.07	1.01	1.14	2.6E-02	8.5E-02
K16043	scyllo-inositol 2-dehydrogenase (NAD+) [EC:1.1.1.370]	0.94	0.89	0.99	2.6E-02	8.5E-02
K11023	pertussis toxin subunit 1 [EC:2.4.2.-]	1.07	1.01	1.14	2.6E-02	8.5E-02
K01539	sodium/potassium-transporting ATPase subunit alpha [EC:7.2.2.13]	0.94	0.88	0.99	2.6E-02	8.6E-02
K05841	sterol 3beta-glucosyltransferase [EC:2.4.1.173]	0.93	0.88	0.99	2.6E-02	8.6E-02
K00193	acetyl-CoA decarbonylase/synthase, CODH/ACS complex subunit beta [EC:2.3.1.169]	0.94	0.88	0.99	2.6E-02	8.6E-02
K12553	penicillin-binding protein 3 [EC:3.4.-.-]	0.93	0.88	0.99	2.6E-02	8.6E-02
K00035	D-galactose 1-dehydrogenase [EC:1.1.1.48]	0.94	0.88	0.99	2.6E-02	8.7E-02

Predictor	Description	HR	2.5% CI	97.5% CI	P	FDR-adjusted P
K01892	histidyl-tRNA synthetase [EC:6.1.1.21]	0.93	0.88	0.99	2.7E-02	8.7E-02
K02802	NA	0.94	0.89	0.99	2.7E-02	8.8E-02
K02501	imidazole glycerol-phosphate synthase subunit HisH [EC:4.3.2.10]	0.93	0.88	0.99	2.7E-02	8.9E-02
K00248	butyryl-CoA dehydrogenase [EC:1.3.8.1]	0.94	0.88	0.99	2.7E-02	8.9E-02
K01884	cysteinyl-tRNA synthetase, unknown class [EC:6.1.1.16]	0.94	0.88	0.99	2.7E-02	8.9E-02
K03816	xanthine phosphoribosyltransferase [EC:2.4.2.22]	0.94	0.89	0.99	2.7E-02	8.9E-02
K05593	aminoglycoside 6-adenylyltransferase [EC:2.7.7.-]	0.93	0.88	0.99	2.8E-02	9.0E-02
K14668	minimal PKS chain-length factor (CLF/KS beta) [EC:2.3.1.-]	0.94	0.89	0.99	2.8E-02	9.0E-02
K15855	exo-1,4-beta-D-glucosaminidase [EC:3.2.1.165]	0.93	0.88	0.99	2.8E-02	9.0E-02
K08600	sortase B [EC:3.4.22.71]	0.93	0.88	0.99	2.8E-02	9.0E-02
K14266	tryptophan 7-halogenase [EC:1.14.19.9]	0.94	0.88	0.99	2.8E-02	9.1E-02
K00695	sucrose synthase [EC:2.4.1.13]	0.94	0.88	0.99	2.8E-02	9.1E-02
K01167	ribonuclease T1 [EC:4.6.1.24]	0.94	0.89	0.99	2.8E-02	9.1E-02
K03383	cyanuric acid amidohydrolase [EC:3.5.2.15]	0.94	0.88	0.99	2.9E-02	9.3E-02
K14165	atypical dual specificity phosphatase [EC:3.1.3.16 3.1.3.48]	0.94	0.88	0.99	2.9E-02	9.3E-02
K01616	multifunctional 2-oxoglutarate metabolism enzyme [EC:2.2.1.5 4.1.1.71 1.2.4.2 2.3.1.61]	0.94	0.89	0.99	2.9E-02	9.3E-02
K05830	LysW-gamma-L-lysine/LysW-L-ornithine aminotransferase [EC:2.6.1.118 2.6.1.-]	0.94	0.89	0.99	2.9E-02	9.4E-02
K03526	(E)-4-hydroxy-3-methylbut-2-enyl-diphosphate synthase [EC:1.17.7.1 1.17.7.3]	0.94	0.88	0.99	2.9E-02	9.4E-02
K01761	methionine-gamma-lyase [EC:4.4.1.11]	0.93	0.88	0.99	2.9E-02	9.4E-02
K03885	NADH:ubiquinone reductase (H+-translocating) [EC:7.1.1.2]	1.07	1.01	1.13	2.9E-02	9.5E-02
K01639	N-acetylneuraminate lyase [EC:4.1.3.3]	1.08	1.01	1.15	3.0E-02	9.5E-02
K01848	methylmalonyl-CoA mutase, N-terminal domain [EC:5.4.99.2]	0.93	0.88	0.99	3.0E-02	9.5E-02
K01849	methylmalonyl-CoA mutase, C-terminal domain [EC:5.4.99.2]	0.93	0.88	0.99	3.0E-02	9.6E-02
K01000	phospho-N-acetylmuramoyl-pentapeptide-transferase [EC:2.7.8.13]	0.93	0.88	0.99	3.0E-02	9.6E-02
K01452	chitin deacetylase [EC:3.5.1.41]	0.94	0.88	0.99	3.0E-02	9.6E-02
K05555	cyclase [EC:4.-.-.-]	0.94	0.89	0.99	3.0E-02	9.7E-02
K01056	peptidyl-tRNA hydrolase, PTH1 family [EC:3.1.1.29]	0.93	0.88	0.99	3.0E-02	9.7E-02
K15780	bifunctional protein TilS/HprT [EC:6.3.4.19 2.4.2.8]	0.94	0.88	0.99	3.0E-02	9.7E-02
K01200	pullulanase [EC:3.2.1.41]	0.94	0.90	0.99	3.0E-02	9.7E-02
K01531	P-type Mg2+ transporter [EC:7.2.2.14]	1.07	1.01	1.13	3.0E-02	9.7E-02
K08100	bilirubin oxidase [EC:1.3.3.5]	0.94	0.88	0.99	3.1E-02	9.8E-02
K00257	acyl-ACP dehydrogenase [EC:1.3.99.-]	0.94	0.89	0.99	3.1E-02	9.8E-02
K01663	imidazole glycerol-phosphate synthase [EC:4.3.2.10]	0.94	0.89	0.99	3.1E-02	9.8E-02
K01734	methylglyoxal synthase [EC:4.2.3.3]	0.94	0.89	0.99	3.1E-02	9.9E-02
K01597	diphosphomevalonate decarboxylase [EC:4.1.1.33]	0.94	0.88	0.99	3.2E-02	1.0E-01
K01955	carbamoyl-phosphate synthase large subunit [EC:6.3.5.5]	0.94	0.89	0.99	3.2E-02	1.0E-01
K13058	mannosylfructose-phosphate synthase [EC:2.4.1.246]	0.94	0.88	0.99	3.2E-02	1.0E-01
K00833	adenosylmethionine---8-amino-7-oxononanoate aminotransferase [EC:2.6.1.62]	1.07	1.01	1.13	3.2E-02	1.0E-01
K15895	UDP-4-amino-4,6-dideoxy-L-N-acetyl-beta-L-altrosamine transaminase [EC:2.6.1.92]	0.94	0.88	0.99	3.2E-02	1.0E-01
K01883	cysteinyl-tRNA synthetase [EC:6.1.1.16]	0.94	0.88	0.99	3.3E-02	1.0E-01
K05551	minimal PKS ketosynthase (KS/KS alpha) [EC:2.3.1.- 2.3.1.260 2.3.1.235]	0.94	0.89	0.99	3.3E-02	1.0E-01
K10212	glycosyl-4,4'-diaponeurosporenoate acyltransferase [EC:2.3.1.-]	0.94	0.88	0.99	3.3E-02	1.0E-01
K00561	23S rRNA (adenine-N6)-dimethyltransferase [EC:2.1.1.184]	0.94	0.88	0.99	3.3E-02	1.0E-01
K01686	mannonate dehydratase [EC:4.2.1.8]	0.94	0.89	1.00	3.3E-02	1.0E-01

Predictor	Description	HR	2.5% CI	97.5% CI	P	FDR-adjusted P
K05550	benzoate/toluate 1,2-dioxygenase subunit beta [EC:1.14.12.10 1.14.12.-]	0.94	0.89	0.99	3.3E-02	1.0E-01
K01215	glucan 1,6-alpha-glucosidase [EC:3.2.1.70]	0.94	0.88	0.99	3.3E-02	1.0E-01
K00581	tetrahydromethanopterin S-methyltransferase subunit E [EC:2.1.1.86]	0.94	0.88	1.00	3.4E-02	1.0E-01
K00824	D-alanine transaminase [EC:2.6.1.21]	0.94	0.88	0.99	3.4E-02	1.0E-01
K15054	(S)-mandelate dehydrogenase [EC:1.1.99.31]	0.94	0.89	1.00	3.4E-02	1.0E-01
K01804	L-arabinose isomerase [EC:5.3.1.4]	0.94	0.89	1.00	3.4E-02	1.0E-01
K13810	transaldolase / glucose-6-phosphate isomerase [EC:2.2.1.2 5.3.1.9]	0.94	0.89	1.00	3.4E-02	1.0E-01
K01869	leucyl-tRNA synthetase [EC:6.1.1.4]	0.94	0.89	1.00	3.4E-02	1.1E-01
K00762	orotate phosphoribosyltransferase [EC:2.4.2.10]	0.94	0.88	1.00	3.4E-02	1.1E-01
K00796	dihydropteroate synthase [EC:2.5.1.15]	1.07	1.01	1.15	3.5E-02	1.1E-01
K05928	tocopherol O-methyltransferase [EC:2.1.1.95]	0.94	0.88	1.00	3.5E-02	1.1E-01
K13687	arabinofuranosyltransferase [EC:2.4.2.-]	0.94	0.89	1.00	3.5E-02	1.1E-01
K03335	inosose dehydratase [EC:4.2.1.44]	0.94	0.88	1.00	3.5E-02	1.1E-01
K03655	ATP-dependent DNA helicase RecG [EC:3.6.4.12]	0.94	0.89	1.00	3.5E-02	1.1E-01
K05296	3(or 17)beta-hydroxysteroid dehydrogenase [EC:1.1.1.51]	0.94	0.89	1.00	3.5E-02	1.1E-01
K01263	NA	0.94	0.89	1.00	3.5E-02	1.1E-01
K16217	geranyl diphosphate 2-C-methyltransferase [EC:2.1.1.255]	0.94	0.89	1.00	3.5E-02	1.1E-01
K14633	ketoreductase RED2 [EC:1.1.1.-]	0.94	0.89	1.00	3.6E-02	1.1E-01
K05574	NAD(P)H-quinone oxidoreductase subunit 3 [EC:7.1.1.2]	0.94	0.88	1.00	3.6E-02	1.1E-01
K13660	beta-1,4-glucosyltransferase [EC:2.4.1.-]	0.94	0.89	1.00	3.6E-02	1.1E-01
K00054	hydroxymethylglutaryl-CoA reductase [EC:1.1.1.88]	0.94	0.88	1.00	3.6E-02	1.1E-01
K00859	dephospho-CoA kinase [EC:2.7.1.24]	1.07	1.00	1.15	3.7E-02	1.1E-01
K14415	tRNA-splicing ligase RtcB (3'-phosphate/5'-hydroxy nucleic acid ligase) [EC:6.5.1.8]	1.07	1.00	1.13	3.7E-02	1.1E-01
K01905	acetate---CoA ligase (ADP-forming) subunit alpha [EC:6.2.1.13]	0.94	0.89	1.00	3.7E-02	1.1E-01
K02224	cobyrinic acid a,c-diamide synthase [EC:6.3.5.9 6.3.5.11]	0.94	0.89	1.00	3.7E-02	1.1E-01
K06152	gluconate 2-dehydrogenase gamma chain [EC:1.1.99.3]	0.94	0.89	1.00	3.7E-02	1.1E-01
K01588	5-(carboxyamino)imidazole ribonucleotide mutase [EC:5.4.99.18]	0.94	0.88	1.00	3.7E-02	1.1E-01
K01889	phenylalanyl-tRNA synthetase alpha chain [EC:6.1.1.20]	0.94	0.88	1.00	3.7E-02	1.1E-01
K03464	muconolactone D-isomerase [EC:5.3.3.4]	0.94	0.89	1.00	3.7E-02	1.1E-01
K13995	maleamate amidohydrolase [EC:3.5.1.107]	0.94	0.89	1.00	3.7E-02	1.1E-01
K03388	heterodisulfide reductase subunit A2 [EC:1.8.7.3 1.8.98.4 1.8.98.5 1.8.98.6]	0.94	0.89	1.00	3.7E-02	1.1E-01
K01372	bleomycin hydrolase [EC:3.4.22.40]	0.94	0.90	1.00	3.8E-02	1.1E-01
K03465	thymidylate synthase (FAD) [EC:2.1.1.148]	0.94	0.88	1.00	3.8E-02	1.1E-01
K08690	cis-2,3-dihydrobiphenyl-2,3-diol dehydrogenase [EC:1.3.1.56]	0.94	0.89	1.00	3.8E-02	1.1E-01
K00790	UDP-N-acetylglucosamine 1-carboxyvinyltransferase [EC:2.5.1.7]	0.94	0.89	1.00	3.8E-02	1.1E-01
K00217	maleylacetate reductase [EC:1.3.1.32]	0.94	0.89	1.00	3.8E-02	1.1E-01
K10907	aminotransferase [EC:2.6.1.-]	0.94	0.88	1.00	3.8E-02	1.1E-01
K07778	two-component system, NarL family, sensor histidine kinase DesK [EC:2.7.13.3]	0.94	0.88	1.00	3.9E-02	1.2E-01
K01619	deoxyribose-phosphate aldolase [EC:4.1.2.4]	0.94	0.89	1.00	3.9E-02	1.2E-01
K02500	imidazole glycerol-phosphate synthase subunit HisF [EC:4.3.2.10]	0.94	0.88	1.00	3.9E-02	1.2E-01
K00461	arachidonate 5-lipoxygenase [EC:1.13.11.34]	0.94	0.89	1.00	3.9E-02	1.2E-01
K00969	nicotinate-nucleotide adenylyltransferase [EC:2.7.7.18]	0.94	0.89	1.00	4.0E-02	1.2E-01
K10673	streptomycin 3\-kinase [EC:2.7.1.87]	0.94	0.89	1.00	4.0E-02	1.2E-01
K03337	5-deoxy-glucuronate isomerase [EC:5.3.1.30]	0.94	0.88	1.00	4.0E-02	1.2E-01

Predictor	Description	HR	2.5% CI	97.5% CI	P	FDR-adjusted P
K11617	two-component system, NarL family, sensor histidine kinase LiaS [EC:2.7.13.3]	0.94	0.88	1.00	4.0E-02	1.2E-01
K00887	undecaprenol kinase [EC:2.7.1.66]	0.94	0.88	1.00	4.0E-02	1.2E-01
K01729	poly(beta-D-mannuronate) lyase [EC:4.2.2.3]	0.94	0.89	1.00	4.0E-02	1.2E-01
K02588	nitrogenase iron protein NifH	0.94	0.88	1.00	4.0E-02	1.2E-01
K00615	transketolase [EC:2.2.1.1]	0.94	0.89	1.00	4.0E-02	1.2E-01
K13941	2-amino-4-hydroxy-6-hydroxymethyldihydropteridine diphosphokinase / dihydropteroate synthase [EC:2.7.6.3 2.5.1.15]	0.94	0.88	1.00	4.0E-02	1.2E-01
K05797	4-cresol dehydrogenase (hydroxylating) flavoprotein subunit [EC:1.17.9.1]	0.94	0.89	1.00	4.0E-02	1.2E-01
K02470	DNA gyrase subunit B [EC:5.6.2.2]	0.94	0.88	1.00	4.0E-02	1.2E-01
K03046	DNA-directed RNA polymerase subunit beta' [EC:2.7.7.6]	0.94	0.88	1.00	4.1E-02	1.2E-01
K03390	heterodisulfide reductase subunit C2 [EC:1.8.7.3 1.8.98.4 1.8.98.5 1.8.98.6]	0.94	0.89	1.00	4.1E-02	1.2E-01
K05535	alpha 1,2-mannosyltransferase [EC:2.4.1.-]	0.94	0.88	1.00	4.1E-02	1.2E-01
K10681	two-component system, OmpR family, sensor histidine kinase SaeS [EC:2.7.13.3]	0.94	0.89	1.00	4.1E-02	1.2E-01
K02343	DNA polymerase III subunit gamma/tau [EC:2.7.7.7]	0.95	0.90	1.00	4.1E-02	1.2E-01
K02110	F-type H+-transporting ATPase subunit c	0.95	0.90	1.00	4.1E-02	1.2E-01
K00543	acetylserotonin O-methyltransferase [EC:2.1.1.4]	0.94	0.89	1.00	4.1E-02	1.2E-01
K01220	6-phospho-beta-galactosidase [EC:3.2.1.85]	0.94	0.89	1.00	4.1E-02	1.2E-01
K10830	manganese/zinc transport system ATP-binding protein [EC:7.2.2.5]	0.94	0.89	1.00	4.1E-02	1.2E-01
K05554	aromatase [EC:4.2.1.-]	0.94	0.89	1.00	4.2E-02	1.2E-01
K00262	glutamate dehydrogenase (NADP+) [EC:1.4.1.4]	0.95	0.90	1.00	4.2E-02	1.2E-01
K14029	methanol dehydrogenase (cytochrome c) subunit 2 [EC:1.1.2.7]	0.94	0.89	1.00	4.2E-02	1.2E-01
K13874	L-arabinonolactonase [EC:3.1.1.15]	0.94	0.89	1.00	4.2E-02	1.2E-01
K04782	isochorismate pyruvate lyase [EC:4.2.99.21]	0.94	0.88	1.00	4.3E-02	1.3E-01
K01583	arginine decarboxylase [EC:4.1.1.19]	0.94	0.89	1.00	4.3E-02	1.3E-01
K01358	ATP-dependent Clp protease, protease subunit [EC:3.4.21.92]	0.94	0.88	1.00	4.3E-02	1.3E-01
K05936	precorrin-4/cobalt-precorrin-4 C11-methyltransferase [EC:2.1.1.133 2.1.1.271]	0.94	0.88	1.00	4.4E-02	1.3E-01
K02491	two-component system, sporulation sensor kinase A [EC:2.7.13.3]	0.94	0.89	1.00	4.4E-02	1.3E-01
K13714	bifunctional autolysin [EC:3.5.1.28 3.2.1.96]	0.94	0.88	1.00	4.4E-02	1.3E-01
K04720	threonine-phosphate decarboxylase [EC:4.1.1.81]	0.95	0.90	1.00	4.4E-02	1.3E-01
K08298	L-carnitine CoA-transferase [EC:2.8.3.21]	1.06	1.00	1.13	4.5E-02	1.3E-01
K00261	glutamate dehydrogenase (NAD(P)+) [EC:1.4.1.3]	0.94	0.88	1.00	4.5E-02	1.3E-01
K01692	enoyl-CoA hydratase [EC:4.2.1.17]	0.94	0.89	1.00	4.5E-02	1.3E-01
K00721	dolichol-phosphate mannosyltransferase [EC:2.4.1.83]	0.95	0.90	1.00	4.5E-02	1.3E-01
K12658	4-hydroxyproline epimerase [EC:5.1.1.8]	0.94	0.89	1.00	4.5E-02	1.3E-01
K12073	1,4-dihydroxy-2-naphthoyl-CoA hydrolase [EC:3.1.2.28]	0.94	0.89	1.00	4.5E-02	1.3E-01
K01856	muconate cycloisomerase [EC:5.5.1.1]	0.94	0.89	1.00	4.5E-02	1.3E-01
K13061	acyl homoserine lactone synthase [EC:2.3.1.184]	0.94	0.89	1.00	4.5E-02	1.3E-01
K11440	choline dehydrogenase [EC:1.1.1.1]	0.94	0.89	1.00	4.5E-02	1.3E-01
K00874	2-dehydro-3-deoxygluconokinase [EC:2.7.1.45]	0.94	0.89	1.00	4.5E-02	1.3E-01
K04068	anaerobic ribonucleoside-triphosphate reductase activating protein [EC:1.97.1.4]	0.94	0.89	1.00	4.6E-02	1.3E-01
K00938	phosphomevalonate kinase [EC:2.7.4.2]	0.94	0.89	1.00	4.6E-02	1.3E-01
K00041	tagaturonate reductase [EC:1.1.1.58]	0.94	0.89	1.00	4.6E-02	1.3E-01
K14153	hydroxymethylpyrimidine kinase / phosphomethylpyrimidine kinase / thiamine-phosphate diphosphorylase [EC:2.7.1.49 2.7.4.7 2.5.1.3]	0.94	0.89	1.00	4.6E-02	1.3E-01
K00927	phosphoglycerate kinase [EC:2.7.2.3]	0.94	0.88	1.00	4.6E-02	1.3E-01
K04128	hydroxymethyl cephem carbamoyltransferase [EC:2.1.3.7]	0.94	0.89	1.00	4.6E-02	1.3E-01

Predictor	Description	HR	2.5% CI	97.5% CI	P	FDR-adjusted P
K01361	lactocepin [EC:3.4.21.96]	0.94	0.89	1.00	4.6E-02	1.3E-01
K00213	7-dehydrocholesterol reductase [EC:1.3.1.21]	0.94	0.88	1.00	4.7E-02	1.3E-01
K04567	lysyl-tRNA synthetase, class II [EC:6.1.1.6]	1.07	1.00	1.14	4.7E-02	1.3E-01
K03822	putative long chain acyl-CoA synthase [EC:6.2.1.-]	0.94	0.89	1.00	4.7E-02	1.3E-01
K01281	X-Pro dipeptidyl-peptidase [EC:3.4.14.11]	0.94	0.89	1.00	4.7E-02	1.3E-01
K00919	4-diphosphocytidyl-2-C-methyl-D-erythritol kinase [EC:2.7.1.148]	0.94	0.88	1.00	4.7E-02	1.4E-01
K03336	3D-(3,5/4)-trihydroxycyclohexane-1,2-dione acylhydrolase (decyclizing) [EC:3.7.1.22]	0.94	0.89	1.00	4.8E-02	1.4E-01
K16050	4,5:9,10-diseco-3-hydroxy-5,9,17-trioxoandrosta-1(10),2-diene-4-oate hydrolase [EC:3.7.1.17]	0.94	0.89	1.00	4.8E-02	1.4E-01
K15981	cholest-4-en-3-one 26-monooxygenase [EC:1.14.15.29]	0.94	0.89	1.00	4.8E-02	1.4E-01
K01253	microsomal epoxide hydrolase [EC:3.3.2.9]	0.94	0.89	1.00	4.8E-02	1.4E-01
K01416	snapalysin [EC:3.4.24.77]	0.94	0.89	1.00	4.8E-02	1.4E-01
K01674	carbonic anhydrase [EC:4.2.1.1]	0.94	0.88	1.00	4.8E-02	1.4E-01
K01437	aspartoacylase [EC:3.5.1.15]	0.94	0.89	1.00	4.8E-02	1.4E-01
K13007	glycosyltransferase WbpL [EC:2.4.1.-]	0.94	0.89	1.00	4.8E-02	1.4E-01
K00170	pyruvate ferredoxin oxidoreductase beta subunit [EC:1.2.7.1]	0.94	0.89	1.00	4.8E-02	1.4E-01
K01657	anthranilate synthase component I [EC:4.1.3.27]	0.95	0.90	1.00	4.9E-02	1.4E-01
K00075	UDP-N-acetylmuramate dehydrogenase [EC:1.3.1.98]	0.95	0.90	1.00	4.9E-02	1.4E-01
K00086	1,3-propanediol dehydrogenase [EC:1.1.1.202]	0.94	0.89	1.00	4.9E-02	1.4E-01
K03169	DNA topoisomerase III [EC:5.6.2.1]	0.94	0.89	1.00	4.9E-02	1.4E-01
K02190	sirohydrochlorin cobaltochelatase [EC:4.99.1.3]	0.95	0.90	1.00	4.9E-02	1.4E-01
K01610	phosphoenolpyruvate carboxykinase (ATP) [EC:4.1.1.49]	0.95	0.89	1.00	5.0E-02	1.4E-01
K13875	L-arabonate dehydrase [EC:4.2.1.25]	0.94	0.89	1.00	5.0E-02	1.4E-01
K00007	D-arabinitol 4-dehydrogenase [EC:1.1.1.11]	0.94	0.89	1.00	5.0E-02	1.4E-01
K00175	2-oxoglutarate/2-oxoacid ferredoxin oxidoreductase subunit beta [EC:1.2.7.3 1.2.7.11]	0.95	0.90	1.00	5.0E-02	1.4E-01
K01756	adenylosuccinate lyase [EC:4.3.2.2]	0.94	0.89	1.00	5.0E-02	1.4E-01
K02120	V/A-type H+/Na+-transporting ATPase subunit D	0.95	0.90	1.00	5.0E-02	1.4E-01
K00917	tagatose 6-phosphate kinase [EC:2.7.1.144]	0.94	0.89	1.00	5.0E-02	1.4E-01
K13798	DNA-directed RNA polymerase subunit B [EC:2.7.7.6]	0.94	0.89	1.00	5.1E-02	1.4E-01
K03366	meso-butanediol dehydrogenase / (S,S)-butanediol dehydrogenase / diacetyl reductase [EC:1.1.1.- 1.1.1.76 1.1.1.304]	0.94	0.89	1.00	5.1E-02	1.4E-01
K10714	methylene-tetrahydromethanopterin dehydrogenase [EC:1.5.1.-]	0.94	0.89	1.00	5.1E-02	1.4E-01
K02204	homoserine kinase type II [EC:2.7.1.39]	0.94	0.89	1.00	5.1E-02	1.4E-01
K15533	1,3-beta-galactosyl-N-acetylhexosamine phosphorylase [EC:2.4.1.211]	0.94	0.89	1.00	5.1E-02	1.4E-01
K01933	phosphoribosylformylglycinamidine cyclo-ligase [EC:6.3.3.1]	0.94	0.89	1.00	5.1E-02	1.4E-01
K01071	medium-chain acyl-[acyl-carrier-protein] hydrolase [EC:3.1.2.21]	0.94	0.89	1.00	5.1E-02	1.4E-01
K01758	cystathionine gamma-lyase [EC:4.4.1.1]	0.94	0.89	1.00	5.1E-02	1.4E-01
K01713	cyclohexadienyl dehydratase [EC:4.2.1.51 4.2.1.91]	0.94	0.89	1.00	5.2E-02	1.4E-01
K03813	molybdenum transport protein [EC:2.4.2.-]	1.06	1.00	1.13	5.2E-02	1.4E-01
K01051	pectinesterase [EC:3.1.1.11]	0.95	0.90	1.00	5.2E-02	1.4E-01
K01881	prolyl-tRNA synthetase [EC:6.1.1.15]	0.94	0.89	1.00	5.2E-02	1.4E-01
K00756	pyrimidine-nucleoside phosphorylase [EC:2.4.2.2]	0.94	0.89	1.00	5.2E-02	1.4E-01
K03918	L-lysine 6-transaminase [EC:2.6.1.36]	0.95	0.89	1.00	5.2E-02	1.4E-01
K13990	glutamate formiminotransferase / formiminotetrahydrofolate cyclodeaminase [EC:2.1.2.5 4.3.1.4]	0.94	0.89	1.00	5.2E-02	1.4E-01
K01448	N-acetylmuramoyl-L-alanine amidase [EC:3.5.1.28]	1.07	1.00	1.15	5.3E-02	1.5E-01
K01790	dTDP-4-dehydrorhamnose 3,5-epimerase [EC:5.1.3.13]	0.95	0.90	1.00	5.3E-02	1.5E-01

Predictor	Description	HR	2.5% CI	97.5% CI	P	FDR-adjusted P
K10775	phenylalanine ammonia-lyase [EC:4.3.1.24]	0.94	0.89	1.00	5.3E-02	1.5E-01
K06113	arabinan endo-1,5-alpha-L-arabinosidase [EC:3.2.1.99]	0.95	0.90	1.00	5.3E-02	1.5E-01
K10216	2-hydroxymuconate-semialdehyde hydrolase [EC:3.7.1.9]	0.94	0.89	1.00	5.3E-02	1.5E-01
K01677	fumarate hydratase subunit alpha [EC:4.2.1.2]	0.94	0.89	1.00	5.3E-02	1.5E-01
K00648	3-oxoacyl-[acyl-carrier-protein] synthase III [EC:2.3.1.180]	1.06	1.00	1.12	5.4E-02	1.5E-01
K08744	cardiolipin synthase (CMP-forming) [EC:2.7.8.41]	0.94	0.89	1.00	5.4E-02	1.5E-01
K05995	dipeptidase E [EC:3.4.13.21]	0.95	0.90	1.00	5.4E-02	1.5E-01
K00218	protochlorophyllide reductase [EC:1.3.1.33]	0.94	0.89	1.00	5.4E-02	1.5E-01
K04110	benzoate-CoA ligase [EC:6.2.1.25]	0.94	0.89	1.00	5.4E-02	1.5E-01
K00703	starch synthase [EC:2.4.1.21]	0.95	0.90	1.00	5.4E-02	1.5E-01
K05823	N-acetyldiaminopimelate deacetylase [EC:3.5.1.47]	0.94	0.89	1.00	5.4E-02	1.5E-01
K03379	cyclohexanone monooxygenase [EC:1.14.13.22]	0.95	0.89	1.00	5.4E-02	1.5E-01
K03783	purine-nucleoside phosphorylase [EC:2.4.2.1]	0.95	0.90	1.00	5.4E-02	1.5E-01
K16190	glucuronokinase [EC:2.7.1.43]	0.94	0.89	1.00	5.4E-02	1.5E-01
K05363	serine/alanine adding enzyme [EC:2.3.2.10]	0.94	0.89	1.00	5.5E-02	1.5E-01
K14028	methanol dehydrogenase (cytochrome c) subunit 1 [EC:1.1.2.7]	0.95	0.89	1.00	5.5E-02	1.5E-01
K05275	pyridoxine 4-dehydrogenase [EC:1.1.1.65]	0.94	0.89	1.00	5.5E-02	1.5E-01
K00567	methylated-DNA-[protein]-cysteine S-methyltransferase [EC:2.1.1.63]	1.07	1.00	1.14	5.5E-02	1.5E-01
K03429	processive 1,2-diacylglycerol beta-glucosyltransferase [EC:2.4.1.315]	0.94	0.89	1.00	5.6E-02	1.5E-01
K00096	glycerol-1-phosphate dehydrogenase [NAD(P)+] [EC:1.1.1.261]	0.94	0.89	1.00	5.6E-02	1.5E-01
K12555	penicillin-binding protein 2A [EC:2.4.1.129 3.4.16.4]	0.94	0.89	1.00	5.6E-02	1.5E-01
K13934	phosphoribosyl-dephospho-CoA transferase [EC:2.7.7.66]	0.94	0.89	1.00	5.6E-02	1.5E-01
K13776	citronellyl-CoA synthetase [EC:6.2.1.-]	0.95	0.89	1.00	5.6E-02	1.5E-01
K03399	cobalt-precorrin-7 (C5)-methyltransferase [EC:2.1.1.289]	0.94	0.89	1.00	5.6E-02	1.5E-01
K11927	ATP-dependent RNA helicase RhlE [EC:3.6.4.13]	1.07	1.00	1.14	5.6E-02	1.5E-01
K08967	1,2-dihydroxy-3-keto-5-methylthiopentene dioxygenase [EC:1.13.11.53 1.13.11.54]	0.94	0.89	1.00	5.6E-02	1.5E-01
K00819	ornithine--oxo-acid transaminase [EC:2.6.1.13]	0.95	0.90	1.00	5.7E-02	1.5E-01
K04561	nitric oxide reductase subunit B [EC:1.7.2.5]	0.94	0.89	1.00	5.7E-02	1.5E-01
K12448	UDP-arabinose 4-epimerase [EC:5.1.3.5]	0.94	0.89	1.00	5.7E-02	1.5E-01
K00133	aspartate-semialdehyde dehydrogenase [EC:1.2.1.11]	0.94	0.89	1.00	5.7E-02	1.5E-01
K13940	dihydroneopterin aldolase / 2-amino-4-hydroxy-6-hydroxymethyldihydropteridine diphosphokinase [EC:4.1.2.25 2.7.6.3]	0.94	0.89	1.00	5.7E-02	1.5E-01
K05783	dihydroxycyclohexadiene carboxylate dehydrogenase [EC:1.3.1.25 1.3.1.-]	0.94	0.89	1.00	5.7E-02	1.5E-01
K13929	malonate decarboxylase alpha subunit [EC:2.3.1.187]	0.94	0.89	1.00	5.7E-02	1.5E-01
K05947	mannosyl-3-phosphoglycerate synthase [EC:2.4.1.217]	0.94	0.89	1.00	5.7E-02	1.5E-01
K00865	glycerate 2-kinase [EC:2.7.1.165]	0.95	0.90	1.00	5.8E-02	1.5E-01
K16044	scyllo-inositol 2-dehydrogenase (NADP+) [EC:1.1.1.371]	0.95	0.89	1.00	5.8E-02	1.5E-01
K01678	fumarate hydratase subunit beta [EC:4.2.1.2]	0.94	0.89	1.00	5.8E-02	1.6E-01
K03100	signal peptidase I [EC:3.4.21.89]	0.95	0.90	1.00	5.8E-02	1.6E-01
K01322	prolyl oligopeptidase [EC:3.4.21.26]	0.95	0.89	1.00	5.9E-02	1.6E-01
K01858	myo-inositol-1-phosphate synthase [EC:5.5.1.4]	0.95	0.90	1.00	5.9E-02	1.6E-01
K03338	5-dehydro-2-deoxygluconokinase [EC:2.7.1.92]	0.94	0.89	1.00	5.9E-02	1.6E-01
K03715	1,2-diacylglycerol 3-beta-galactosyltransferase [EC:2.4.1.46]	0.94	0.89	1.00	5.9E-02	1.6E-01
K02191	cobalt-precorrin-6B (C15)-methyltransferase [EC:2.1.1.196]	0.94	0.89	1.00	5.9E-02	1.6E-01
K07547	2-[hydroxy(phenyl)methyl]-succinyl-CoA dehydrogenase BbsC subunit [EC:1.1.1.35]	0.95	0.89	1.00	6.0E-02	1.6E-01

Predictor	Description	HR	2.5% CI	97.5% CI	P	FDR-adjusted P
K01261	glutamyl aminopeptidase [EC:3.4.11.7]	0.94	0.89	1.00	6.0E-02	1.6E-01
K00876	uridine kinase [EC:2.7.1.48]	0.95	0.90	1.00	6.0E-02	1.6E-01
K01843	lysine 2,3-aminomutase [EC:5.4.3.2]	0.95	0.90	1.00	6.1E-02	1.6E-01
K02232	adenosylcobyric acid synthase [EC:6.3.5.10]	0.95	0.90	1.00	6.1E-02	1.6E-01
K12554	alanine adding enzyme [EC:2.3.2.-]	0.94	0.89	1.00	6.1E-02	1.6E-01
K01870	isoleucyl-tRNA synthetase [EC:6.1.1.5]	0.94	0.89	1.00	6.1E-02	1.6E-01
K01243	adenosylhomocysteine nucleosidase [EC:3.2.2.9]	0.95	0.90	1.00	6.1E-02	1.6E-01
K01953	asparagine synthase (glutamine-hydrolysing) [EC:6.3.5.4]	0.95	0.90	1.00	6.2E-02	1.6E-01
K13524	4-aminobutyrate aminotransferase / (S)-3-amino-2-methylpropionate transaminase [EC:2.6.1.19 2.6.1.22]	0.94	0.89	1.00	6.2E-02	1.6E-01
K06989	aspartate dehydrogenase [EC:1.4.1.21]	0.95	0.90	1.00	6.2E-02	1.6E-01
K08964	methylthioribulose-1-phosphate dehydratase [EC:4.2.1.109]	0.94	0.89	1.00	6.2E-02	1.6E-01
K08260	adenosylcobinamide hydrolase [EC:3.5.1.90]	0.95	0.89	1.00	6.3E-02	1.6E-01
K05597	glutamin-(asparagin-)ase [EC:3.5.1.38]	0.95	0.89	1.00	6.3E-02	1.7E-01
K07816	GTP pyrophosphokinase [EC:2.7.6.5]	0.94	0.89	1.00	6.4E-02	1.7E-01
K03564	thioredoxin-dependent peroxiredoxin [EC:1.11.1.24]	0.95	0.90	1.00	6.4E-02	1.7E-01
K08851	TP53 regulating kinase and related kinases [EC:2.7.11.1]	0.95	0.89	1.00	6.4E-02	1.7E-01
K01187	alpha-glucosidase [EC:3.2.1.20]	0.95	0.90	1.00	6.5E-02	1.7E-01
K01459	N-carbamoyl-D-amino-acid hydrolase [EC:3.5.1.77]	0.95	0.89	1.00	6.5E-02	1.7E-01
K10206	LL-diaminopimelate aminotransferase [EC:2.6.1.83]	0.95	0.90	1.00	6.5E-02	1.7E-01
K01174	micrococcal nuclease [EC:3.1.31.1]	0.95	0.89	1.00	6.6E-02	1.7E-01
K01205	alpha-N-acetylglucosaminidase [EC:3.2.1.50]	0.95	0.90	1.00	6.6E-02	1.7E-01
K02236	leader peptidase (prepilin peptidase) / N-methyltransferase [EC:3.4.23.43 2.1.1.-]	0.95	0.89	1.00	6.6E-02	1.7E-01
K01401	aureolysin [EC:3.4.24.29]	0.94	0.89	1.00	6.6E-02	1.7E-01
K04478	monofunctional glycosyltransferase [EC:2.4.1.129]	0.94	0.89	1.00	6.6E-02	1.7E-01
K11695	peptidoglycan pentaglycine glycine transferase (the fourth and fifth glycine) [EC:2.3.2.18]	0.94	0.89	1.00	6.6E-02	1.7E-01
K11694	peptidoglycan pentaglycine glycine transferase (the second and third glycine) [EC:2.3.2.17]	0.94	0.89	1.00	6.6E-02	1.7E-01
K11442	putative uridylyltransferase [EC:2.7.7.-]	0.94	0.89	1.00	6.6E-02	1.7E-01
K07681	two-component system, NarL family, vancomycin resistance sensor histidine kinase VraS [EC:2.7.13.3]	0.94	0.89	1.00	6.6E-02	1.7E-01
K06151	gluconate 2-dehydrogenase alpha chain [EC:1.1.99.3]	0.95	0.89	1.00	6.6E-02	1.7E-01
K01685	altronate hydrolase [EC:4.2.1.7]	0.95	0.90	1.00	6.7E-02	1.7E-01
K01710	dTDP-glucose 4,6-dehydratase [EC:4.2.1.46]	0.95	0.90	1.00	6.7E-02	1.7E-01
K10676	2,4-dichlorophenol 6-monooxygenase [EC:1.14.13.20]	0.95	0.89	1.00	6.7E-02	1.7E-01
K01890	phenylalanyl-tRNA synthetase beta chain [EC:6.1.1.20]	0.95	0.89	1.00	6.8E-02	1.7E-01
K00975	glucose-1-phosphate adenylyltransferase [EC:2.7.7.27]	0.95	0.89	1.00	6.8E-02	1.7E-01
K01781	mandelate racemase [EC:5.1.2.2]	0.95	0.89	1.00	6.8E-02	1.7E-01
K01956	carbamoyl-phosphate synthase small subunit [EC:6.3.5.5]	0.94	0.89	1.00	6.8E-02	1.7E-01
K00476	aspartate beta-hydroxylase [EC:1.14.11.16]	0.95	0.89	1.00	6.8E-02	1.7E-01
K05998	pseudomonalisin [EC:3.4.21.100]	0.95	0.89	1.00	6.8E-02	1.7E-01
K01278	dipeptidyl-peptidase 4 [EC:3.4.14.5]	0.95	0.90	1.00	6.9E-02	1.7E-01
K00349	Na+-transporting NADH:ubiquinone oxidoreductase subunit D [EC:7.2.1.1]	0.95	0.90	1.00	6.9E-02	1.7E-01
K12250	pentalenene synthase [EC:4.2.3.7]	0.94	0.89	1.00	6.9E-02	1.7E-01
K04340	scyllo-inosamine-4-phosphate amidinotransferase 1 [EC:2.1.4.2]	0.94	0.89	1.00	6.9E-02	1.7E-01
K02346	DNA polymerase IV [EC:2.7.7.7]	1.06	1.00	1.13	6.9E-02	1.7E-01
K03692	glucosylglycerol-phosphate synthase [EC:2.4.1.213]	0.95	0.89	1.00	6.9E-02	1.8E-01

Predictor	Description	HR	2.5% CI	97.5% CI	P	FDR-adjusted P
K15234	citryl-CoA lyase [EC:4.1.3.34]	0.95	0.89	1.00	6.9E-02	1.8E-01
K00347	Na+-transporting NADH:ubiquinone oxidoreductase subunit B [EC:7.2.1.1]	0.95	0.90	1.00	7.0E-02	1.8E-01
K01560	2-haloacid dehalogenase [EC:3.8.1.2]	0.95	0.89	1.00	7.0E-02	1.8E-01
K01399	pseudolysin [EC:3.4.24.26]	0.95	0.89	1.00	7.0E-02	1.8E-01
K10908	DNA-directed RNA polymerase, mitochondrial [EC:2.7.7.6]	0.95	0.89	1.00	7.0E-02	1.8E-01
K10773	endonuclease III [EC:4.2.99.18]	0.95	0.89	1.00	7.0E-02	1.8E-01
K02188	cobalt-precorrin-5B (C1)-methyltransferase [EC:2.1.1.195]	0.95	0.90	1.00	7.0E-02	1.8E-01
K01738	cysteine synthase [EC:2.5.1.47]	1.06	1.00	1.13	7.0E-02	1.8E-01
K00348	Na+-transporting NADH:ubiquinone oxidoreductase subunit C [EC:7.2.1.1]	0.95	0.90	1.00	7.1E-02	1.8E-01
K00941	hydroxymethylpyrimidine/phosphomethylpyrimidine kinase [EC:2.7.1.49 2.7.4.7]	0.95	0.90	1.00	7.1E-02	1.8E-01
K14659	chitooligosaccharide deacetylase [EC:3.5.1.-]	0.95	0.89	1.00	7.1E-02	1.8E-01
K14666	N-acetylglucosaminyltransferase [EC:2.4.1.-]	0.95	0.89	1.00	7.1E-02	1.8E-01
K16303	p-cumate 2,3-dioxygenase subunit beta [EC:1.14.12.25]	0.95	0.89	1.00	7.1E-02	1.8E-01
K05362	UDP-N-acetylmuramoyl-L-alanyl-D-glutamate-L-lysine ligase [EC:6.3.2.7]	0.95	0.89	1.00	7.1E-02	1.8E-01
K07255	taurine dehydrogenase small subunit [EC:1.4.2.-]	0.95	0.89	1.00	7.1E-02	1.8E-01
K09882	cobaltochelatase CobS [EC:6.6.1.2]	0.95	0.89	1.00	7.1E-02	1.8E-01
K03386	peroxiredoxin 2/4 [EC:1.11.1.24]	1.06	0.99	1.13	7.1E-02	1.8E-01
K01270	dipeptidase D [EC:3.4.13.-]	0.95	0.90	1.00	7.1E-02	1.8E-01
K01181	endo-1,4-beta-xylanase [EC:3.2.1.8]	0.95	0.90	1.00	7.1E-02	1.8E-01
K01972	DNA ligase (NAD+) [EC:6.5.1.2]	1.06	0.99	1.13	7.2E-02	1.8E-01
K15929	glycosyltransferase [EC:2.4.1.-]	0.95	0.89	1.01	7.2E-02	1.8E-01
K02338	DNA polymerase III subunit beta [EC:2.7.7.7]	0.95	0.89	1.01	7.2E-02	1.8E-01
K02536	UDP-3-O-[3-hydroxymyristoyl] glucosamine N-acyltransferase [EC:2.3.1.191]	1.06	0.99	1.14	7.3E-02	1.8E-01
K00346	Na+-transporting NADH:ubiquinone oxidoreductase subunit A [EC:7.2.1.1]	0.95	0.90	1.00	7.3E-02	1.8E-01
K00103	L-gulonolactone oxidase [EC:1.1.3.8]	0.95	0.90	1.00	7.3E-02	1.8E-01
K12997	rhamnosyltransferase [EC:2.4.1.-]	0.95	0.89	1.01	7.3E-02	1.8E-01
K09880	enolase-phosphatase E1 [EC:3.1.3.77]	0.95	0.89	1.01	7.3E-02	1.8E-01
K02793	mannose PTS system EIIA component [EC:2.7.1.191]	0.95	0.89	1.01	7.3E-02	1.8E-01
K15987	K(+)-stimulated pyrophosphate-energized sodium pump [EC:7.1.3.2]	0.95	0.90	1.00	7.3E-02	1.8E-01
K11181	dissimilatory sulfite reductase beta subunit [EC:1.8.99.5]	0.95	0.89	1.01	7.3E-02	1.8E-01
K03427	type I restriction enzyme M protein [EC:2.1.1.72]	0.95	0.90	1.00	7.4E-02	1.8E-01
K14205	phosphatidylglycerol lysyltransferase [EC:2.3.2.3]	0.95	0.89	1.01	7.4E-02	1.8E-01
K02335	DNA polymerase I [EC:2.7.7.7]	0.95	0.89	1.01	7.4E-02	1.8E-01
K02112	F-type H+/Na+-transporting ATPase subunit beta [EC:7.1.2.2 7.2.2.1]	0.95	0.90	1.00	7.4E-02	1.8E-01
K02756	NA	0.95	0.89	1.01	7.5E-02	1.8E-01
K01130	arylsulfatase [EC:3.1.6.1]	1.06	0.99	1.12	7.5E-02	1.9E-01
K03275	UDP-glucose:(glucosyl)LPS alpha-1,3-glucosyltransferase [EC:2.4.1.-]	1.06	0.99	1.12	7.5E-02	1.9E-01
K07173	S-ribosylhomocysteine lyase [EC:4.4.1.21]	0.95	0.90	1.01	7.5E-02	1.9E-01
K12994	O-antigen biosynthesis alpha-1,3-mannosyltransferase [EC:2.4.1.349 2.4.1.-]	0.95	0.90	1.01	7.6E-02	1.9E-01
K01092	myo-inositol-1(or 4)-monophosphatase [EC:3.1.3.25]	0.95	0.90	1.01	7.6E-02	1.9E-01
K03043	DNA-directed RNA polymerase subunit beta [EC:2.7.7.6]	0.95	0.90	1.01	7.6E-02	1.9E-01
K01246	DNA-3-methyladenine glycosylase I [EC:3.2.2.20]	0.95	0.90	1.01	7.6E-02	1.9E-01
K05946	N-acetylglucosaminyldiphosphoundecaprenol N-acetyl-beta-D-mannosaminyltransferase [EC:2.4.1.187]	0.95	0.90	1.01	7.7E-02	1.9E-01
K03741	arsenate reductase (thioredoxin) [EC:1.20.4.4]	0.95	0.90	1.01	7.7E-02	1.9E-01

Predictor	Description	HR	2.5% CI	97.5% CI	P	FDR-adjusted P
K10815	hydrogen cyanide synthase HcnB [EC:1.4.99.5]	0.95	0.90	1.01	7.8E-02	1.9E-01
K10816	hydrogen cyanide synthase HcnC [EC:1.4.99.5]	0.95	0.90	1.01	7.8E-02	1.9E-01
K01938	formate--tetrahydrofolate ligase [EC:6.3.4.3]	0.95	0.90	1.01	7.8E-02	1.9E-01
K00018	glycerate dehydrogenase [EC:1.1.1.29]	0.95	0.90	1.01	7.8E-02	1.9E-01
K00048	lactaldehyde reductase [EC:1.1.1.77]	0.95	0.90	1.01	7.8E-02	1.9E-01
K00930	acetylglutamate kinase [EC:2.7.2.8]	0.95	0.89	1.01	7.8E-02	1.9E-01
K07442	tRNA (adenine57-N1/adenine58-N1)-methyltransferase catalytic subunit [EC:2.1.1.219 2.1.1.220]	0.95	0.89	1.01	7.9E-02	1.9E-01
K01695	tryptophan synthase alpha chain [EC:4.2.1.20]	0.95	0.90	1.01	7.9E-02	1.9E-01
K13669	alpha-1,2-mannosyltransferase [EC:2.4.1.-]	0.95	0.90	1.01	8.0E-02	1.9E-01
K12556	penicillin-binding protein 2X	0.95	0.89	1.01	8.0E-02	2.0E-01
K07179	RIO kinase 2 [EC:2.7.11.1]	0.95	0.90	1.01	8.1E-02	2.0E-01
K00918	ADP-dependent phosphofructokinase/glucokinase [EC:2.7.1.146 2.7.1.147]	0.95	0.90	1.01	8.2E-02	2.0E-01
K03929	para-nitrobenzyl esterase [EC:3.1.1.-]	0.95	0.89	1.01	8.3E-02	2.0E-01
K12253	5-guanidino-2-oxopentanoate decarboxylase [EC:4.1.1.75]	0.95	0.89	1.01	8.3E-02	2.0E-01
K00520	mercuric reductase [EC:1.16.1.1]	0.95	0.89	1.01	8.4E-02	2.0E-01
K01297	muramoyltetrapeptide carboxypeptidase [EC:3.4.17.13]	1.05	0.99	1.12	8.4E-02	2.0E-01
K00616	transaldolase [EC:2.2.1.2]	0.95	0.91	1.01	8.4E-02	2.0E-01
K00760	hypoxanthine phosphoribosyltransferase [EC:2.4.2.8]	0.95	0.89	1.01	8.4E-02	2.0E-01
K00395	adenylylsulfate reductase, subunit B [EC:1.8.99.2]	0.95	0.89	1.01	8.4E-02	2.0E-01
K14583	1,2-dihydroxynaphthalene dioxygenase [EC:1.13.11.56]	0.95	0.89	1.01	8.5E-02	2.0E-01
K14584	2-hydroxychromene-2-carboxylate isomerase [EC:5.99.1.4]	0.95	0.89	1.01	8.5E-02	2.0E-01
K14582	cis-1,2-dihydro-1,2-dihydroxynaphthalene/dibenzothiophene dihydrodiol dehydrogenase [EC:1.3.1.29 1.3.1.60]	0.95	0.89	1.01	8.5E-02	2.0E-01
K14580	naphthalene 1,2-dioxygenase subunit beta [EC:1.14.12.12 1.14.12.23 1.14.12.24]	0.95	0.89	1.01	8.5E-02	2.0E-01
K06046	long-chain-fatty-acid---luciferin-component ligase [EC:6.2.1.19]	0.95	0.89	1.01	8.5E-02	2.1E-01
K03778	D-lactate dehydrogenase [EC:1.1.1.28]	0.95	0.90	1.01	8.6E-02	2.1E-01
K00844	hexokinase [EC:2.7.1.1]	1.05	0.99	1.12	8.7E-02	2.1E-01
K14189	uncharacterized oxidoreductase [EC:1.-.-.-]	0.95	0.90	1.01	8.7E-02	2.1E-01
K03218	23S rRNA (guanosine2251-2'-O)-methyltransferase [EC:2.1.1.185]	1.06	0.99	1.13	8.7E-02	2.1E-01
K05575	NAD(P)H-quinone oxidoreductase subunit 4 [EC:7.1.1.2]	0.95	0.89	1.01	8.7E-02	2.1E-01
K12132	eukaryotic-like serine/threonine-protein kinase [EC:2.7.11.1]	0.95	0.89	1.01	8.8E-02	2.1E-01
K16215	2-ketoarginine methyltransferase [EC:2.1.1.243]	0.95	0.89	1.01	8.8E-02	2.1E-01
K07154	serine/threonine-protein kinase HipA [EC:2.7.11.1]	0.95	0.90	1.01	8.9E-02	2.1E-01
K07272	rhamnosyltransferase [EC:2.4.1.-]	0.95	0.89	1.01	8.9E-02	2.1E-01
K00275	pyridoxamine 5'-phosphate oxidase [EC:1.4.3.5]	1.05	0.99	1.12	8.9E-02	2.1E-01
K01878	glycyl-tRNA synthetase alpha chain [EC:6.1.1.14]	1.05	0.99	1.12	8.9E-02	2.1E-01
K09680	type II pantothenate kinase [EC:2.7.1.33]	0.95	0.90	1.01	8.9E-02	2.1E-01
K07991	archaeal preflagellin peptidase FlaK [EC:3.4.23.52]	0.95	0.90	1.01	8.9E-02	2.1E-01
K14630	two-component flavin-dependent monooxygenase [EC:1.14.14.-]	0.95	0.89	1.01	8.9E-02	2.1E-01
K06120	glycerol dehydratase large subunit [EC:4.2.1.30]	0.95	0.89	1.01	8.9E-02	2.1E-01
K10530	L-lactate oxidase [EC:1.1.3.2]	0.95	0.90	1.01	8.9E-02	2.1E-01
K00560	thymidylate synthase [EC:2.1.1.45]	1.06	0.99	1.13	8.9E-02	2.1E-01
K01259	proline iminopeptidase [EC:3.4.11.5]	0.95	0.89	1.01	9.0E-02	2.1E-01
K15059	2-aminophenol/2-amino-5-chlorophenol 1,6-dioxygenase subunit beta [EC:1.13.11.74 1.13.11.76]	0.95	0.89	1.01	9.0E-02	2.1E-01
K15253	chlorocatechol 1,2-dioxygenase [EC:1.13.11.-]	0.95	0.89	1.01	9.0E-02	2.1E-01

Predictor	Description	HR	2.5% CI	97.5% CI	P	FDR-adjusted P
K01860	chloromuconate cycloisomerase [EC:5.5.1.7]	0.95	0.89	1.01	9.0E-02	2.1E-01
K01183	chitinase [EC:3.2.1.14]	0.95	0.90	1.01	9.0E-02	2.1E-01
K08685	quinohemoprotein amine dehydrogenase [EC:1.4.9.1]	0.95	0.90	1.01	9.0E-02	2.1E-01
K01235	alpha-glucuronidase [EC:3.2.1.139]	0.95	0.90	1.01	9.1E-02	2.1E-01
K00254	dihydroorotate dehydrogenase [EC:1.3.5.2]	1.05	0.99	1.12	9.1E-02	2.2E-01
K00687	penicillin-binding protein 2B	0.95	0.89	1.01	9.2E-02	2.2E-01
K06967	tRNA (adenine22-N1)-methyltransferase [EC:2.1.1.217]	0.95	0.89	1.01	9.3E-02	2.2E-01
K02121	V/A-type H+/Na+-transporting ATPase subunit E	0.96	0.91	1.01	9.3E-02	2.2E-01
K01887	arginyl-tRNA synthetase [EC:6.1.1.19]	0.95	0.89	1.01	9.3E-02	2.2E-01
K15242	2,6-dichloro-p-hydroquinone 1,2-dioxygenase [EC:1.13.11.-]	0.95	0.90	1.01	9.5E-02	2.2E-01
K00602	phosphoribosylaminoimidazolecarboxamide formyltransferase / IMP cyclohydrolase [EC:2.1.2.3 3.5.4.10]	1.06	0.99	1.13	9.5E-02	2.2E-01
K03518	aerobic carbon-monoxide dehydrogenase small subunit [EC:1.2.5.3]	0.95	0.89	1.01	9.5E-02	2.2E-01
K05917	sterol 14alpha-demethylase [EC:1.14.14.154 1.14.15.36]	0.95	0.90	1.01	9.5E-02	2.2E-01
K01198	xylan 1,4-beta-xylosidase [EC:3.2.1.37]	0.95	0.89	1.01	9.5E-02	2.2E-01
K01939	adenylosuccinate synthase [EC:6.3.4.4]	0.95	0.89	1.01	9.5E-02	2.2E-01
K12990	rhamnosyltransferase [EC:2.4.1.-]	0.95	0.89	1.01	9.6E-02	2.2E-01
K01635	tagatose 1,6-diphosphate aldolase [EC:4.1.2.40]	0.95	0.90	1.01	9.6E-02	2.2E-01
K04342	streptomycin-6-phosphatase [EC:3.1.3.39]	0.95	0.90	1.01	9.6E-02	2.3E-01
K01879	glycyl-tRNA synthetase beta chain [EC:6.1.1.14]	1.05	0.99	1.12	9.7E-02	2.3E-01
K01590	histidine decarboxylase [EC:4.1.1.22]	1.05	0.99	1.12	9.7E-02	2.3E-01
K00571	site-specific DNA-methyltransferase (adenine-specific) [EC:2.1.1.72]	1.05	0.99	1.12	9.8E-02	2.3E-01
K01057	6-phosphogluconolactonase [EC:3.1.1.31]	0.96	0.90	1.01	9.8E-02	2.3E-01
K01776	glutamate racemase [EC:5.1.1.3]	0.96	0.90	1.01	9.8E-02	2.3E-01
K15634	2,3-bisphosphoglycerate-dependent phosphoglycerate mutase [EC:5.4.2.11]	0.95	0.90	1.01	9.8E-02	2.3E-01
K01201	glucosylceramidase [EC:3.2.1.45]	0.95	0.90	1.01	9.8E-02	2.3E-01
K00658	2-oxoglutarate dehydrogenase E2 component (dihydrolipoamide succinyltransferase) [EC:2.3.1.61]	1.05	0.99	1.12	9.8E-02	2.3E-01
K00240	succinate dehydrogenase / fumarate reductase, iron-sulfur subunit [EC:1.3.5.1 1.3.5.4]	0.96	0.91	1.01	9.9E-02	2.3E-01
K01876	aspartyl-tRNA synthetase [EC:6.1.1.12]	0.95	0.89	1.01	1.0E-01	2.3E-01
K03381	catechol 1,2-dioxygenase [EC:1.13.11.1]	0.95	0.90	1.01	1.0E-01	2.3E-01
K13060	acyl homoserine lactone synthase [EC:2.3.1.184]	0.95	0.90	1.01	1.0E-01	2.3E-01
K11645	fructose-bisphosphate aldolase, class I [EC:4.1.2.13]	0.95	0.90	1.01	1.0E-01	2.3E-01
K11991	tRNA(adenine34) deaminase [EC:3.5.4.33]	0.96	0.91	1.01	1.0E-01	2.3E-01
K13678	1,2-diacylglycerol-3-alpha-glucose alpha-1,2-galactosyltransferase [EC:2.4.1.-]	0.95	0.89	1.01	1.0E-01	2.3E-01
K00177	2-oxoglutarate ferredoxin oxidoreductase subunit gamma [EC:1.2.7.3]	0.96	0.91	1.01	1.0E-01	2.3E-01
K01621	xylulose-5-phosphate/fructose-6-phosphate phosphoketolase [EC:4.1.2.9 4.1.2.22]	0.95	0.89	1.01	1.0E-01	2.3E-01
K01274	beta-Ala-Xaa dipeptidase [EC:3.4.13.-]	0.95	0.90	1.01	1.0E-01	2.3E-01
K14340	mannosyltransferase [EC:2.4.1.-]	0.95	0.90	1.01	1.0E-01	2.3E-01
K06606	2-keto-myo-inositol isomerase [EC:5.3.99.11]	0.95	0.90	1.01	1.0E-01	2.4E-01
K08475	two-component system, NtrC family, phosphoglycerate transport system sensor histidine kinase PgtB [EC:2.7.13.3]	0.95	0.90	1.01	1.0E-01	2.4E-01
K00929	butyrate kinase [EC:2.7.2.7]	0.96	0.91	1.01	1.0E-01	2.4E-01
K01803	triosephosphate isomerase (TIM) [EC:5.3.1.1]	0.95	0.89	1.01	1.0E-01	2.4E-01
K11611	meromycolic acid enoyl-[acyl-carrier-protein] reductase [EC:1.3.1.118]	0.95	0.90	1.01	1.0E-01	2.4E-01
K06133	4'-phosphopantetheinyl transferase [EC:2.7.8.-]	0.95	0.90	1.01	1.0E-01	2.4E-01
K00259	alanine dehydrogenase [EC:1.4.1.1]	0.96	0.91	1.01	1.0E-01	2.4E-01

Predictor	Description	HR	2.5% CI	97.5% CI	P	FDR-adjusted P
K03763	DNA polymerase III subunit alpha, Gram-positive type [EC:2.7.7.7]	0.95	0.90	1.01	1.0E-01	2.4E-01
K07407	alpha-galactosidase [EC:3.2.1.22]	0.95	0.90	1.01	1.0E-01	2.4E-01
K15904	bifunctional N6-L-threonylcarbamoyladenine synthase / protein kinase Bud32 [EC:2.3.1.234 2.7.11.1]	0.95	0.90	1.01	1.0E-01	2.4E-01
K00622	arylamine N-acetyltransferase [EC:2.3.1.5]	0.95	0.90	1.01	1.0E-01	2.4E-01
K08604	vibriolysin [EC:3.4.24.25]	0.95	0.90	1.01	1.0E-01	2.4E-01
K00995	CDP-diacylglycerol---glycerol-3-phosphate 3-phosphatidyltransferase [EC:2.7.8.5]	0.95	0.90	1.01	1.1E-01	2.4E-01
K02341	DNA polymerase III subunit delta' [EC:2.7.7.7]	0.95	0.89	1.01	1.1E-01	2.4E-01
K15918	D-glycerate 3-kinase [EC:2.7.1.31]	0.95	0.90	1.01	1.1E-01	2.4E-01
K00951	GTP pyrophosphokinase [EC:2.7.6.5]	0.96	0.90	1.01	1.1E-01	2.4E-01
K16176	methylamine---corrinoid protein Co-methyltransferase [EC:2.1.1.248]	0.95	0.89	1.01	1.1E-01	2.4E-01
K10533	limonene-1,2-epoxide hydrolase [EC:3.3.2.8]	0.95	0.90	1.01	1.1E-01	2.4E-01
K00970	poly(A) polymerase [EC:2.7.7.19]	1.05	0.99	1.12	1.1E-01	2.4E-01
K03707	thiaminase (transcriptional activator TenA) [EC:3.5.99.2]	0.95	0.90	1.01	1.1E-01	2.4E-01
K10916	two-component system, CAI-1 autoinducer sensor kinase/phosphatase CqsS [EC:2.7.13.3 3.1.3.-]	0.95	0.90	1.01	1.1E-01	2.4E-01
K14188	D-alanine--poly(phosphoribitol) ligase subunit 2 [EC:6.1.1.13]	0.95	0.90	1.01	1.1E-01	2.4E-01
K15760	toluene monooxygenase system protein A [EC:1.14.13.236 1.14.13.-]	0.95	0.90	1.01	1.1E-01	2.4E-01
K00180	indolepyruvate ferredoxin oxidoreductase, beta subunit [EC:1.2.7.8]	0.96	0.91	1.01	1.1E-01	2.4E-01
K00948	ribose-phosphate pyrophosphokinase [EC:2.7.6.1]	0.95	0.89	1.01	1.1E-01	2.4E-01
K13002	glycosyltransferase [EC:2.4.1.-]	0.96	0.90	1.01	1.1E-01	2.5E-01
K00743	N-acetyllactosaminide 3-alpha-galactosyltransferase [EC:2.4.1.87]	0.95	0.89	1.01	1.1E-01	2.5E-01
K14585	trans-o-hydroxybenzylidenepyruvate hydratase-aldolase [EC:4.1.2.45]	0.95	0.90	1.01	1.1E-01	2.5E-01
K10814	hydrogen cyanide synthase HcnA [EC:1.4.99.5]	0.95	0.90	1.01	1.1E-01	2.5E-01
K15866	2-(1,2-epoxy-1,2-dihydrophenyl)acetyl-CoA isomerase [EC:5.3.3.18]	0.95	0.90	1.01	1.1E-01	2.5E-01
K01945	phosphoribosylamine---glycine ligase [EC:6.3.4.13]	0.95	0.90	1.01	1.1E-01	2.5E-01
K11263	acetyl-CoA/propionyl-CoA carboxylase, biotin carboxylase, biotin carboxyl carrier protein [EC:6.4.1.2 6.4.1.3 6.3.4.14]	0.95	0.90	1.01	1.1E-01	2.5E-01
K01937	CTP synthase [EC:6.3.4.2]	0.95	0.90	1.01	1.1E-01	2.5E-01
K01750	ornithine cyclodeaminase [EC:4.3.1.12]	0.95	0.90	1.01	1.1E-01	2.5E-01
K02786	lactose PTS system EIIA component [EC:2.7.1.207]	0.95	0.90	1.01	1.1E-01	2.5E-01
K01436	amidohydrolase [EC:3.5.1.-]	0.95	0.90	1.01	1.1E-01	2.5E-01
K12988	alpha-1,3-rhamnosyltransferase [EC:2.4.1.-]	0.95	0.90	1.01	1.1E-01	2.5E-01
K04564	superoxide dismutase, Fe-Mn family [EC:1.15.1.1]	1.06	0.99	1.13	1.1E-01	2.5E-01
K02119	V/A-type H+/Na+-transporting ATPase subunit C	0.95	0.90	1.01	1.1E-01	2.5E-01
K01195	beta-glucuronidase [EC:3.2.1.31]	0.95	0.90	1.01	1.1E-01	2.5E-01
K15357	N-formylmaleamate deformylase [EC:3.5.1.106]	0.95	0.90	1.01	1.1E-01	2.5E-01
K00055	aryl-alcohol dehydrogenase [EC:1.1.1.90]	0.95	0.90	1.01	1.1E-01	2.5E-01
K01137	N-acetylglucosamine-6-sulfatase [EC:3.1.6.14]	0.95	0.90	1.01	1.1E-01	2.5E-01
K03339	6-phospho-5-dehydro-2-deoxy-D-gluconate aldolase [EC:4.1.2.29]	0.95	0.90	1.01	1.1E-01	2.5E-01
K02117	V/A-type H+/Na+-transporting ATPase subunit A [EC:7.1.2.2 7.2.2.1]	0.96	0.91	1.01	1.1E-01	2.5E-01
K05601	hydroxylamine reductase [EC:1.7.99.1]	1.05	0.99	1.11	1.1E-01	2.5E-01
K13419	serine/threonine-protein kinase PknK [EC:2.7.11.1]	0.95	0.90	1.01	1.1E-01	2.6E-01
K00620	glutamate N-acetyltransferase / amino-acid N-acetyltransferase [EC:2.3.1.35 2.3.1.1]	0.95	0.90	1.01	1.2E-01	2.6E-01
K10834	heme transport system ATP-binding protein [EC:7.6.2.5]	0.95	0.90	1.01	1.2E-01	2.6E-01
K00604	methionyl-tRNA formyltransferase [EC:2.1.2.9]	0.95	0.90	1.01	1.2E-01	2.6E-01
K14379	tartrate-resistant acid phosphatase type 5 [EC:3.1.3.2]	0.96	0.90	1.01	1.2E-01	2.6E-01

Predictor	Description	HR	2.5% CI	97.5% CI	P	FDR-adjusted P
K00046	gluconate 5-dehydrogenase [EC:1.1.1.69]	0.96	0.90	1.01	1.2E-01	2.6E-01
K00340	NADH-quinone oxidoreductase subunit K [EC:7.1.1.2]	1.05	0.99	1.12	1.2E-01	2.6E-01
K09903	uridylate kinase [EC:2.7.4.22]	0.95	0.90	1.01	1.2E-01	2.6E-01
K00245	fumarate reductase iron-sulfur subunit [EC:1.3.5.4]	1.05	0.99	1.11	1.2E-01	2.6E-01
K16147	starch synthase (maltosyl-transferring) [EC:2.4.99.16]	0.95	0.90	1.01	1.2E-01	2.6E-01
K00294	1-pyrroline-5-carboxylate dehydrogenase [EC:1.2.1.88]	0.95	0.90	1.01	1.2E-01	2.6E-01
K15896	UDP-4-amino-4,6-dideoxy-N-acetyl-beta-L-altrosamine N-acetyltransferase [EC:2.3.1.202]	0.95	0.90	1.01	1.2E-01	2.6E-01
K01819	galactose-6-phosphate isomerase [EC:5.3.1.26]	0.95	0.90	1.01	1.2E-01	2.6E-01
K01730	oligogalacturonide lyase [EC:4.2.2.6]	0.96	0.90	1.01	1.2E-01	2.6E-01
K05603	formimidoylglutamate deiminase [EC:3.5.3.13]	0.95	0.90	1.01	1.2E-01	2.6E-01
K14471	succinyl-CoA:(S)-malate CoA-transferase subunit A [EC:2.8.3.22]	0.95	0.90	1.01	1.2E-01	2.6E-01
K13043	N-succinyl-L-ornithine transcarbamylase [EC:2.1.3.11]	0.96	0.91	1.01	1.2E-01	2.6E-01
K01697	cystathionine beta-synthase [EC:4.2.1.22]	0.95	0.90	1.01	1.2E-01	2.6E-01
K00857	thymidine kinase [EC:2.7.1.21]	0.96	0.91	1.01	1.2E-01	2.6E-01
K00939	adenylate kinase [EC:2.7.4.3]	0.95	0.90	1.01	1.2E-01	2.6E-01
K12339	S-sulfo-L-cysteine synthase (O-acetyl-L-serine-dependent) [EC:2.5.1.144]	0.95	0.90	1.01	1.2E-01	2.6E-01
K02527	3-deoxy-D-manno-octulosonic-acid transferase [EC:2.4.99.12 2.4.99.13 2.4.99.14 2.4.99.15]	1.05	0.99	1.13	1.2E-01	2.6E-01
K00077	2-dehydropantoate 2-reductase [EC:1.1.1.169]	1.05	0.99	1.13	1.2E-01	2.6E-01
K12343	3-oxo-5-alpha-steroid 4-dehydrogenase 1 [EC:1.3.1.22]	0.96	0.91	1.01	1.2E-01	2.6E-01
K02523	octaprenyl-diphosphate synthase [EC:2.5.1.90]	0.96	0.90	1.01	1.2E-01	2.6E-01
K12999	glucosyltransferase [EC:2.4.1.-]	0.95	0.90	1.01	1.2E-01	2.6E-01
K00158	pyruvate oxidase [EC:1.2.3.3]	0.95	0.90	1.01	1.2E-01	2.6E-01
K01190	beta-galactosidase [EC:3.2.1.23]	0.96	0.91	1.01	1.2E-01	2.7E-01
K01580	glutamate decarboxylase [EC:4.1.1.15]	1.05	0.99	1.12	1.2E-01	2.7E-01
K01925	UDP-N-acetylmuramoylalanine--D-glutamate ligase [EC:6.3.2.9]	1.05	0.99	1.12	1.2E-01	2.7E-01
K03768	peptidyl-prolyl cis-trans isomerase B (cyclophilin B) [EC:5.2.1.8]	0.96	0.90	1.01	1.2E-01	2.7E-01
K00219	2,4-dienoyl-CoA reductase (NADPH2) [EC:1.3.1.34]	1.05	0.99	1.11	1.2E-01	2.7E-01
K08095	cutinase [EC:3.1.1.74]	0.96	0.90	1.01	1.2E-01	2.7E-01
K00590	site-specific DNA-methyltransferase (cytosine-N4-specific) [EC:2.1.1.113]	1.05	0.99	1.11	1.2E-01	2.7E-01
K01821	4-oxalocrotonate tautomerase [EC:5.3.2.6]	1.05	0.99	1.11	1.2E-01	2.7E-01
K05913	2,4'-dihydroxyacetophenone dioxygenase [EC:1.13.11.41]	0.95	0.90	1.01	1.2E-01	2.7E-01
K01654	N-acetylneuraminate synthase [EC:2.5.1.56]	0.96	0.90	1.01	1.2E-01	2.7E-01
K01874	methionyl-tRNA synthetase [EC:6.1.1.10]	0.95	0.90	1.01	1.2E-01	2.7E-01
K10211	4,4'-diaponeurosporenoate glycosyltransferase [EC:2.4.1.-]	0.95	0.90	1.01	1.3E-01	2.7E-01
K06027	vesicle-fusing ATPase [EC:3.6.4.6]	0.95	0.90	1.01	1.3E-01	2.7E-01
K04098	hydroxyquinol 1,2-dioxygenase [EC:1.13.11.37]	0.96	0.90	1.01	1.3E-01	2.7E-01
K00174	2-oxoglutarate/2-oxoacid ferredoxin oxidoreductase subunit alpha [EC:1.2.7.3 1.2.7.11]	0.96	0.91	1.01	1.3E-01	2.7E-01
K07056	16S rRNA (cytidine1402-2'-O)-methyltransferase [EC:2.1.1.198]	1.05	0.99	1.12	1.3E-01	2.7E-01
K08255	CoA-disulfide reductase [EC:1.8.1.14]	0.95	0.89	1.01	1.3E-01	2.7E-01
K12551	monofunctional glycosyltransferase [EC:2.4.1.129]	0.95	0.89	1.01	1.3E-01	2.7E-01
K01866	tyrosyl-tRNA synthetase [EC:6.1.1.1]	0.95	0.90	1.01	1.3E-01	2.7E-01
K00606	3-methyl-2-oxobutanoate hydroxymethyltransferase [EC:2.1.2.11]	1.05	0.99	1.12	1.3E-01	2.7E-01
K05956	geranylgeranyl transferase type-2 subunit beta [EC:2.5.1.60]	0.95	0.90	1.01	1.3E-01	2.7E-01
K13602	bacteriochlorophyllide d C-12(1)-methyltransferase [EC:2.1.1.331]	0.96	0.90	1.01	1.3E-01	2.7E-01

Predictor	Description	HR	2.5% CI	97.5% CI	P	FDR-adjusted P
K00464	all-trans-8'-apo-beta-carotenal 15,15'-oxygenase [EC:1.13.11.75]	0.96	0.90	1.01	1.3E-01	2.7E-01
K02641	ferredoxin--NADP+ reductase [EC:1.18.1.2]	0.96	0.90	1.01	1.3E-01	2.7E-01
K01210	glucan 1,3-beta-glucosidase [EC:3.2.1.58]	0.95	0.90	1.01	1.3E-01	2.7E-01
K06209	chorismate mutase [EC:5.4.99.5]	0.95	0.90	1.01	1.3E-01	2.7E-01
K03735	ethanolamine ammonia-lyase large subunit [EC:4.3.1.7]	0.95	0.90	1.01	1.3E-01	2.7E-01
K00847	fructokinase [EC:2.7.1.4]	0.96	0.91	1.01	1.3E-01	2.8E-01
K00765	ATP phosphoribosyltransferase [EC:2.4.2.17]	0.96	0.90	1.01	1.3E-01	2.8E-01
K06016	beta-ureidopropionase / N-carbamoyl-L-amino-acid hydrolase [EC:3.5.1.6 3.5.1.87]	0.95	0.90	1.01	1.3E-01	2.8E-01
K00537	arsenate reductase (glutaredoxin) [EC:1.20.4.1]	1.05	0.99	1.11	1.3E-01	2.8E-01
K00805	heptaprenyl diphosphate synthase component 1 [EC:2.5.1.30]	0.95	0.90	1.01	1.3E-01	2.8E-01
K13797	DNA-directed RNA polymerase subunit beta-beta' [EC:2.7.7.6]	0.96	0.90	1.01	1.3E-01	2.8E-01
K01154	type I restriction enzyme, S subunit [EC:3.1.21.3]	0.96	0.91	1.01	1.3E-01	2.8E-01
K01812	glucuronate isomerase [EC:5.3.1.12]	0.96	0.91	1.01	1.3E-01	2.8E-01
K00394	adenylylsulfate reductase, subunit A [EC:1.8.99.2]	0.95	0.90	1.01	1.3E-01	2.8E-01
K00766	anthranilate phosphoribosyltransferase [EC:2.4.2.18]	0.96	0.91	1.01	1.3E-01	2.8E-01
K12297	23S rRNA (guanine2069-N7)-methyltransferase / 23S rRNA (guanine2445-N2)-methyltransferase [EC:2.1.1.264 2.1.1.173]	1.05	0.99	1.11	1.3E-01	2.8E-01
K16305	fructose-bisphosphate aldolase / 6-deoxy-5-ketofructose 1-phosphate synthase [EC:4.1.2.13 2.2.1.11]	0.96	0.90	1.01	1.3E-01	2.8E-01
K16045	3beta-hydroxy-Delta5-steroid dehydrogenase / steroid Delta-isomerase [EC:1.1.1.145 5.3.3.1]	0.96	0.90	1.01	1.3E-01	2.8E-01
K13005	O-antigen biosynthesis alpha-1,3-abequosyltransferase [EC:2.4.1.60]	0.96	0.90	1.01	1.3E-01	2.8E-01
K06023	HPr kinase/phosphorylase [EC:2.7.11.- 2.7.4.-]	0.96	0.90	1.01	1.3E-01	2.8E-01
K01737	6-pyruvoyltetrahydropterin/6-carboxytetrahydropterin synthase [EC:4.2.3.12 4.1.2.50]	1.05	0.98	1.12	1.3E-01	2.8E-01
K03525	type III pantothenate kinase [EC:2.7.1.33]	0.96	0.91	1.01	1.3E-01	2.8E-01
K01712	urocanate hydratase [EC:4.2.1.49]	0.96	0.91	1.01	1.3E-01	2.8E-01
K05578	NAD(P)H-quinone oxidoreductase subunit 6 [EC:7.1.1.2]	0.96	0.90	1.01	1.3E-01	2.8E-01
K00020	3-hydroxyisobutyrate dehydrogenase [EC:1.1.1.31]	0.96	0.90	1.01	1.3E-01	2.8E-01
K07656	two-component system, OmpR family, sensor histidine kinase TrcS [EC:2.7.13.3]	0.96	0.90	1.01	1.4E-01	2.9E-01
K03394	precorrin-2/cobalt-factor-2 C20-methyltransferase [EC:2.1.1.130 2.1.1.151]	0.96	0.91	1.01	1.4E-01	2.9E-01
K13571	proteasome accessory factor A [EC:6.3.1.19]	0.95	0.90	1.01	1.4E-01	2.9E-01
K06179	23S rRNA pseudouridine955/2504/2580 synthase [EC:5.4.99.24]	0.96	0.90	1.01	1.4E-01	2.9E-01
K01596	phosphoenolpyruvate carboxykinase (GTP) [EC:4.1.1.32]	0.96	0.90	1.01	1.4E-01	2.9E-01
K00983	N-acylneuraminate cytidylyltransferase [EC:2.7.7.43]	0.96	0.91	1.01	1.4E-01	2.9E-01
K12308	beta-galactosidase [EC:3.2.1.23]	0.96	0.91	1.01	1.4E-01	2.9E-01
K16055	trehalose 6-phosphate synthase/phosphatase [EC:2.4.1.15 3.1.3.12]	0.96	0.91	1.01	1.4E-01	2.9E-01
K05367	penicillin-binding protein 1C [EC:2.4.1.129]	1.05	0.99	1.11	1.4E-01	2.9E-01
K00343	NADH-quinone oxidoreductase subunit N [EC:7.1.1.2]	1.05	0.99	1.11	1.4E-01	2.9E-01
K15897	UDP-2,4-diacetamido-2,4,6-trideoxy-beta-L-altropyranose hydrolase [EC:3.6.1.57]	0.96	0.90	1.01	1.4E-01	2.9E-01
K04757	serine/threonine-protein kinase RsbW [EC:2.7.11.1]	0.96	0.90	1.01	1.4E-01	2.9E-01
K00864	glycerol kinase [EC:2.7.1.30]	0.96	0.90	1.01	1.4E-01	2.9E-01
K00713	UDP-glucose:(glucosyl)LPS alpha-1,2-glucosyltransferase [EC:2.4.1.-]	1.05	0.99	1.11	1.4E-01	2.9E-01
K10708	fructoselysine 6-phosphate deglycase [EC:3.5.-.-]	0.96	0.90	1.01	1.4E-01	2.9E-01
K00039	ribitol 2-dehydrogenase [EC:1.1.1.56]	0.96	0.90	1.01	1.4E-01	2.9E-01
K07768	two-component system, OmpR family, sensor histidine kinase SenX3 [EC:2.7.13.3]	0.96	0.90	1.02	1.4E-01	3.0E-01
K02567	nitrate reductase (cytochrome) [EC:1.9.6.1]	1.05	0.99	1.11	1.4E-01	3.0E-01
K01704	3-isopropylmalate/(R)-2-methylmalate dehydratase small subunit [EC:4.2.1.33 4.2.1.35]	0.96	0.90	1.02	1.4E-01	3.0E-01

Predictor	Description	HR	2.5% CI	97.5% CI	P	FDR-adjusted P
K01779	aspartate racemase [EC:5.1.1.13]	0.96	0.90	1.02	1.4E-01	3.0E-01
K07271	lipopolysaccharide cholinephosphotransferase [EC:2.7.8.-]	0.96	0.91	1.01	1.4E-01	3.0E-01
K00832	aromatic-amino-acid transaminase [EC:2.6.1.57]	1.05	0.98	1.11	1.4E-01	3.0E-01
K07655	two-component system, OmpR family, sensor histidine kinase PrrB [EC:2.7.13.3]	0.96	0.90	1.01	1.5E-01	3.0E-01
K14257	tetracycline 7-halogenase / FADH2 O2-dependent halogenase [EC:1.14.19.49 1.14.19.-]	0.96	0.90	1.02	1.5E-01	3.0E-01
K06013	STE24 endopeptidase [EC:3.4.24.84]	0.96	0.90	1.02	1.5E-01	3.0E-01
K02217	ferritin [EC:1.16.3.2]	0.96	0.91	1.01	1.5E-01	3.0E-01
K00097	4-hydroxythreonine-4-phosphate dehydrogenase [EC:1.1.1.262]	1.05	0.98	1.12	1.5E-01	3.0E-01
K15785	L-2,4-diaminobutyrate transaminase [EC:2.6.1.76]	0.96	0.90	1.02	1.5E-01	3.0E-01
K04071	NA	0.96	0.90	1.02	1.5E-01	3.0E-01
K00846	ketohexokinase [EC:2.7.1.3]	0.96	0.90	1.02	1.5E-01	3.1E-01
K00634	phosphate butyryltransferase [EC:2.3.1.19]	0.96	0.91	1.01	1.5E-01	3.1E-01
K01875	seryl-tRNA synthetase [EC:6.1.1.11]	0.96	0.90	1.02	1.5E-01	3.1E-01
K03279	UDP-glucose:(galactosyl)LPS alpha-1,2-glucosyltransferase [EC:2.4.1.58]	1.05	0.98	1.11	1.5E-01	3.1E-01
K15765	toluene monooxygenase electron transfer component [EC:1.18.1.3]	0.96	0.90	1.02	1.5E-01	3.1E-01
K00511	squalene monooxygenase [EC:1.14.14.17]	0.96	0.90	1.02	1.5E-01	3.1E-01
K07444	putative N6-adenine-specific DNA methylase [EC:2.1.1.-]	0.96	0.91	1.01	1.5E-01	3.1E-01
K01786	NA	0.96	0.90	1.02	1.5E-01	3.1E-01
K03780	L(+)-tartrate dehydratase beta subunit [EC:4.2.1.32]	1.04	0.98	1.11	1.5E-01	3.1E-01
K01481	protein-arginine deiminase [EC:3.5.3.15]	0.96	0.90	1.02	1.5E-01	3.1E-01
K14469	acrylyl-CoA reductase (NADPH) / 3-hydroxypropionyl-CoA dehydratase / 3-hydroxypropionyl-CoA synthetase [EC:1.3.1.84 4.2.1.116 6.2.1.36]	0.96	0.90	1.02	1.5E-01	3.1E-01
K08094	6-phospho-3-hexuloisomerase [EC:5.3.1.27]	0.96	0.90	1.02	1.5E-01	3.1E-01
K00450	gentisate 1,2-dioxygenase [EC:1.13.11.4]	0.96	0.90	1.02	1.5E-01	3.1E-01
K05573	NAD(P)H-quinone oxidoreductase subunit 2 [EC:7.1.1.2]	0.96	0.90	1.02	1.5E-01	3.1E-01
K00962	polyribonucleotide nucleotidyltransferase [EC:2.7.7.8]	0.96	0.91	1.02	1.5E-01	3.2E-01
K03738	aldehyde:ferredoxin oxidoreductase [EC:1.2.7.5]	0.96	0.90	1.02	1.5E-01	3.2E-01
K01867	tryptophanyl-tRNA synthetase [EC:6.1.1.2]	0.96	0.90	1.02	1.5E-01	3.2E-01
K11173	hydroxyacid-oxoacid transhydrogenase [EC:1.1.99.24]	0.96	0.90	1.02	1.5E-01	3.2E-01
K01854	UDP-galactopyranose mutase [EC:5.4.99.9]	0.96	0.91	1.02	1.6E-01	3.2E-01
K07408	cytochrome P450 family 1 subfamily A1 [EC:1.14.14.1]	1.04	0.98	1.11	1.6E-01	3.2E-01
K00485	dimethylaniline monooxygenase (N-oxide forming) [EC:1.14.13.8]	1.04	0.98	1.11	1.6E-01	3.2E-01
K02563	UDP-N-acetylglucosamine--N-acetylmuramyl-(pentapeptide) pyrophosphoryl-undecaprenol N-acetylglucosamine transferase [EC:2.4.1.227]	0.96	0.90	1.02	1.6E-01	3.2E-01
K01153	type I restriction enzyme, R subunit [EC:3.1.21.3]	0.96	0.91	1.02	1.6E-01	3.2E-01
K01805	xylose isomerase [EC:5.3.1.5]	0.96	0.91	1.01	1.6E-01	3.2E-01
K11753	riboflavin kinase / FMN adenylyltransferase [EC:2.7.1.26 2.7.7.2]	0.96	0.90	1.02	1.6E-01	3.2E-01
K00978	glucose-1-phosphate cytidylyltransferase [EC:2.7.7.33]	0.96	0.91	1.02	1.6E-01	3.2E-01
K08692	malate-CoA ligase subunit alpha [EC:6.2.1.9]	0.96	0.90	1.02	1.6E-01	3.2E-01
K00806	undecaprenyl diphosphate synthase [EC:2.5.1.31]	0.96	0.90	1.02	1.6E-01	3.2E-01
K00128	aldehyde dehydrogenase (NAD+) [EC:1.2.1.3]	0.96	0.90	1.02	1.6E-01	3.2E-01
K04516	chorismate mutase [EC:5.4.99.5]	0.96	0.91	1.02	1.6E-01	3.2E-01
K01709	CDP-glucose 4,6-dehydratase [EC:4.2.1.45]	0.96	0.91	1.02	1.6E-01	3.2E-01
K00367	ferredoxin-nitrate reductase [EC:1.7.7.2]	0.96	0.91	1.02	1.6E-01	3.2E-01
K16212	4-O-beta-D-mannosyl-D-glucose phosphorylase [EC:2.4.1.281]	0.96	0.91	1.02	1.6E-01	3.2E-01
K15232	citryl-CoA synthetase large subunit [EC:6.2.1.18]	0.96	0.90	1.02	1.6E-01	3.2E-01

Predictor	Description	HR	2.5% CI	97.5% CI	P	FDR-adjusted P
K02782	glucitol/sorbitol PTS system EIIB component [EC:2.7.1.198]	0.96	0.90	1.02	1.6E-01	3.2E-01
K01940	argininosuccinate synthase [EC:6.3.4.5]	0.96	0.90	1.02	1.6E-01	3.3E-01
K15907	pentalenene oxygenase [EC:1.14.15.32]	0.96	0.90	1.02	1.6E-01	3.3E-01
K01744	aspartate ammonia-lyase [EC:4.3.1.1]	0.96	0.91	1.02	1.6E-01	3.3E-01
K00525	ribonucleoside-diphosphate reductase alpha chain [EC:1.17.4.1]	1.05	0.98	1.12	1.6E-01	3.3E-01
K11752	diaminohydroxyphosphoribosylaminopyrimidine deaminase / 5-amino-6-(5-phosphoribosylamino)uracil reductase [EC:3.5.4.26 1.1.1.193]	0.96	0.91	1.02	1.6E-01	3.3E-01
K01216	licheninase [EC:3.2.1.73]	1.04	0.98	1.11	1.6E-01	3.3E-01
K13018	UDP-2-acetamido-3-amino-2,3-dideoxy-glucuronate N-acetyltransferase [EC:2.3.1.201]	0.96	0.90	1.02	1.6E-01	3.3E-01
K00886	polyphosphate glucokinase [EC:2.7.1.63]	0.96	0.90	1.02	1.6E-01	3.3E-01
K01409	N6-L-threonylcarbamoyladenine synthase [EC:2.3.1.234]	0.96	0.90	1.02	1.7E-01	3.3E-01
K13786	cob(II)yrinic acid a,c-diamide reductase [EC:1.16.8.-]	0.96	0.90	1.02	1.7E-01	3.3E-01
K02028	polar amino acid transport system ATP-binding protein [EC:7.4.2.1]	0.96	0.90	1.02	1.7E-01	3.3E-01
K15784	N2-acetyl-L-2,4-diaminobutanoate deacetylase [EC:3.5.1.125]	0.96	0.91	1.02	1.7E-01	3.3E-01
K01693	imidazoleglycerol-phosphate dehydratase [EC:4.2.1.19]	0.96	0.90	1.02	1.7E-01	3.3E-01
K11533	fatty acid synthase, bacteria type [EC:2.3.1.-]	0.96	0.90	1.02	1.7E-01	3.3E-01
K00052	3-isopropylmalate dehydrogenase [EC:1.1.1.85]	0.96	0.90	1.02	1.7E-01	3.3E-01
K05301	sulfite dehydrogenase (cytochrome) subunit A [EC:1.8.2.1]	0.96	0.91	1.02	1.7E-01	3.3E-01
K00033	6-phosphogluconate dehydrogenase [EC:1.1.1.44 1.1.1.343]	0.96	0.91	1.02	1.7E-01	3.3E-01
K13787	geranylgeranyl diphosphate synthase, type I [EC:2.5.1.1 2.5.1.10 2.5.1.29]	0.96	0.90	1.02	1.7E-01	3.4E-01
K00074	3-hydroxybutyryl-CoA dehydrogenase [EC:1.1.1.157]	0.96	0.90	1.02	1.7E-01	3.4E-01
K04103	indolepyruvate decarboxylase [EC:4.1.1.74]	0.96	0.90	1.02	1.7E-01	3.4E-01
K12987	alpha-1,6-rhamnosyltransferase [EC:2.4.1.-]	0.96	0.91	1.02	1.7E-01	3.4E-01
K08289	phosphoribosylglycinamide formyltransferase 2 [EC:6.3.1.21]	0.96	0.91	1.02	1.7E-01	3.4E-01
K00802	spermine synthase [EC:2.5.1.22]	0.96	0.90	1.02	1.7E-01	3.4E-01
K01462	peptide deformylase [EC:3.5.1.88]	0.96	0.90	1.02	1.7E-01	3.4E-01
K00226	dihydroorotate dehydrogenase (fumarate) [EC:1.3.98.1]	0.96	0.91	1.02	1.7E-01	3.4E-01
K08265	heterodisulfide reductase subunit E [EC:1.8.98.1]	0.96	0.90	1.02	1.7E-01	3.4E-01
K01906	6-carboxyhexanoate--CoA ligase [EC:6.2.1.14]	0.96	0.90	1.02	1.7E-01	3.4E-01
K01280	tripeptidyl-peptidase II [EC:3.4.14.10]	0.96	0.90	1.02	1.7E-01	3.4E-01
K01921	D-alanine-D-alanine ligase [EC:6.3.2.4]	1.04	0.98	1.10	1.7E-01	3.4E-01
K00032	phosphogluconate 2-dehydrogenase [EC:1.1.1.43]	0.96	0.91	1.02	1.7E-01	3.4E-01
K15731	carboxy-terminal domain RNA polymerase II polypeptide A small phosphatase [EC:3.1.3.16]	0.96	0.91	1.02	1.7E-01	3.4E-01
K01468	imidazolonepropionase [EC:3.5.2.7]	0.96	0.91	1.02	1.7E-01	3.4E-01
K01048	lysophospholipase [EC:3.1.1.5]	0.96	0.90	1.02	1.7E-01	3.4E-01
K04518	prephenate dehydratase [EC:4.2.1.51]	0.96	0.91	1.02	1.7E-01	3.4E-01
K13001	mannosyltransferase [EC:2.4.1.-]	0.96	0.90	1.02	1.7E-01	3.4E-01
K02535	UDP-3-O-[3-hydroxymyristoyl] N-acetylglucosamine deacetylase [EC:3.5.1.108]	1.04	0.98	1.11	1.7E-01	3.4E-01
K01811	alpha-D-xyloside xylohydrolase [EC:3.2.1.177]	0.96	0.91	1.02	1.7E-01	3.4E-01
K09650	rhomboid-like protein [EC:3.4.21.105]	0.96	0.90	1.02	1.7E-01	3.4E-01
K04092	chorismate mutase [EC:5.4.99.5]	0.96	0.90	1.02	1.7E-01	3.4E-01
K00210	NA	1.04	0.98	1.11	1.7E-01	3.4E-01
K01054	acylglycerol lipase [EC:3.1.1.23]	0.96	0.90	1.02	1.7E-01	3.4E-01
K00338	NADH-quinone oxidoreductase subunit I [EC:7.1.1.2]	1.05	0.98	1.12	1.7E-01	3.4E-01
K11323	histone arginine demethylase JMJD6 [EC:1.14.11.-]	0.96	0.90	1.02	1.8E-01	3.4E-01

Predictor	Description	HR	2.5% CI	97.5% CI	P	FDR-adjusted P
K13019	UDP-GlcNAc3NAcA epimerase [EC:5.1.3.23]	0.96	0.91	1.02	1.8E-01	3.4E-01
K14974	6-hydroxynicotinate 3-monooxygenase [EC:1.14.13.114]	0.96	0.91	1.02	1.8E-01	3.5E-01
K00284	glutamate synthase (ferredoxin) [EC:1.4.7.1]	0.96	0.90	1.02	1.8E-01	3.5E-01
K00332	NADH-quinone oxidoreductase subunit C [EC:7.1.1.2]	0.96	0.90	1.02	1.8E-01	3.5E-01
K12292	ATP-binding cassette, subfamily C, bacterial, competence factor transporting protein [EC:3.4.22.-]	0.96	0.91	1.02	1.8E-01	3.5E-01
K01551	arsenite/tail-anchored protein-transporting ATPase [EC:7.3.2.7 7.3.-.-]	0.96	0.91	1.02	1.8E-01	3.5E-01
K06169	tRNA 2-(methylsulfanyl)-N6-isopentenyladenosine37 hydroxylase [EC:1.14.99.69]	0.96	0.90	1.02	1.8E-01	3.5E-01
K00342	NADH-quinone oxidoreductase subunit M [EC:7.1.1.2]	1.05	0.98	1.12	1.8E-01	3.5E-01
K08325	NADP-dependent alcohol dehydrogenase [EC:1.1.-.-]	0.96	0.91	1.02	1.8E-01	3.5E-01
K13040	two-component system, LuxR family, sensor histidine kinase TtrS [EC:2.7.13.3]	0.96	0.90	1.02	1.8E-01	3.5E-01
K01817	phosphoribosylanthranilate isomerase [EC:5.3.1.24]	0.96	0.91	1.02	1.8E-01	3.6E-01
K00652	8-amino-7-oxononanoate synthase [EC:2.3.1.47]	1.04	0.98	1.11	1.8E-01	3.6E-01
K11640	two-component system, LytTR family, sensor histidine kinase NatK [EC:2.7.13.3]	0.96	0.90	1.02	1.8E-01	3.6E-01
K13016	UDP-N-acetyl-2-amino-2-deoxyglucuronate dehydrogenase [EC:1.1.1.335]	0.96	0.91	1.02	1.8E-01	3.6E-01
K07704	two-component system, LytTR family, sensor histidine kinase LytS [EC:2.7.13.3]	0.96	0.90	1.02	1.8E-01	3.6E-01
K01284	peptidyl-dipeptidase Dcp [EC:3.4.15.5]	0.96	0.91	1.02	1.8E-01	3.6E-01
K08659	dipeptidase [EC:3.4.-.-]	0.96	0.90	1.02	1.8E-01	3.6E-01
K01808	ribose 5-phosphate isomerase B [EC:5.3.1.6]	0.96	0.91	1.02	1.8E-01	3.6E-01
K10619	p-cumate 2,3-dioxygenase subunit alpha [EC:1.14.12.25]	0.96	0.91	1.02	1.8E-01	3.6E-01
K14661	nodulation protein F [EC:2.3.1.-]	0.96	0.90	1.02	1.8E-01	3.6E-01
K01778	diaminopimelate epimerase [EC:5.1.1.7]	0.96	0.91	1.02	1.8E-01	3.6E-01
K00281	glycine dehydrogenase [EC:1.4.4.2]	1.04	0.98	1.11	1.8E-01	3.6E-01
K14067	malate-CoA ligase subunit beta [EC:6.2.1.9]	0.96	0.91	1.02	1.8E-01	3.6E-01
K06912	alpha-ketoglutarate-dependent 2,4-dichlorophenoxyacetate dioxygenase [EC:1.14.11.-]	0.96	0.91	1.02	1.8E-01	3.6E-01
K03389	heterodisulfide reductase subunit B2 [EC:1.8.7.3 1.8.98.4 1.8.98.5 1.8.98.6]	0.96	0.91	1.02	1.9E-01	3.6E-01
K06215	pyridoxal 5'-phosphate synthase pdxS subunit [EC:4.3.3.6]	0.96	0.91	1.02	1.9E-01	3.6E-01
K03168	DNA topoisomerase I [EC:5.6.2.1]	0.96	0.91	1.02	1.9E-01	3.6E-01
K09828	Delta24-sterol reductase [EC:1.3.1.72 1.3.1.-]	0.96	0.91	1.02	1.9E-01	3.6E-01
K14534	4-hydroxybutyryl-CoA dehydratase / vinylacetyl-CoA-Delta-isomerase [EC:4.2.1.120 5.3.3.3]	0.96	0.91	1.02	1.9E-01	3.6E-01
K01507	inorganic pyrophosphatase [EC:3.6.1.1]	0.96	0.90	1.02	1.9E-01	3.6E-01
K01338	ATP-dependent Lon protease [EC:3.4.21.53]	0.96	0.91	1.02	1.9E-01	3.6E-01
K01101	4-nitrophenyl phosphatase [EC:3.1.3.41]	0.96	0.91	1.02	1.9E-01	3.6E-01
K01771	1-phosphatidylinositol phosphodiesterase [EC:4.6.1.13]	1.04	0.98	1.10	1.9E-01	3.6E-01
K01772	protoporphyrin/coproporphyrin ferrochelatase [EC:4.99.1.1 4.99.1.9]	1.04	0.98	1.10	1.9E-01	3.6E-01
K12410	NAD-dependent deacetylase [EC:2.3.1.286]	0.96	0.91	1.02	1.9E-01	3.6E-01
K15976	putative NAD(P)H nitroreductase [EC:1.-.-.-]	0.96	0.91	1.02	1.9E-01	3.6E-01
K01893	asparaginyl-tRNA synthetase [EC:6.1.1.22]	0.96	0.91	1.02	1.9E-01	3.6E-01
K14631	flavin reductase ActVB [EC:1.5.1.-]	0.96	0.91	1.02	1.9E-01	3.6E-01
K15899	pseudaminic acid cytidylyltransferase [EC:2.7.7.81]	0.96	0.91	1.02	1.9E-01	3.6E-01
K05351	D-xylulose reductase [EC:1.1.1.9]	0.96	0.90	1.02	1.9E-01	3.6E-01
K04072	acetaldehyde dehydrogenase / alcohol dehydrogenase [EC:1.2.1.10 1.1.1.1]	0.96	0.90	1.02	1.9E-01	3.6E-01
K02495	oxygen-independent coproporphyrinogen III oxidase [EC:1.3.98.3]	1.04	0.98	1.10	1.9E-01	3.6E-01
K03470	ribonuclease HII [EC:3.1.26.4]	0.96	0.91	1.02	1.9E-01	3.7E-01
K13243	c-di-GMP-specific phosphodiesterase [EC:3.1.4.52]	1.04	0.98	1.11	1.9E-01	3.7E-01

Predictor	Description	HR	2.5% CI	97.5% CI	P	FDR-adjusted P
K14466	4-hydroxybutyrate---CoA ligase (AMP-forming) [EC:6.2.1.40]	0.96	0.90	1.02	1.9E-01	3.7E-01
K00208	enoyl-[acyl-carrier protein] reductase I [EC:1.3.1.9 1.3.1.10]	1.04	0.98	1.11	1.9E-01	3.7E-01
K08264	heterodisulfide reductase subunit D [EC:1.8.98.1]	0.96	0.91	1.02	1.9E-01	3.7E-01
K01385	thermopsin [EC:3.4.23.42]	0.96	0.90	1.02	1.9E-01	3.7E-01
K00333	NADH-quinone oxidoreductase subunit D [EC:7.1.1.2]	0.96	0.90	1.02	2.0E-01	3.7E-01
K04517	prephenate dehydrogenase [EC:1.3.1.12]	0.96	0.91	1.02	2.0E-01	3.7E-01
K01935	dethiobiotin synthetase [EC:6.3.3.3]	1.04	0.98	1.11	2.0E-01	3.7E-01
K00209	enoyl-[acyl-carrier protein] reductase / trans-2-enoyl-CoA reductase (NAD+) [EC:1.3.1.9 1.3.1.44]	0.96	0.90	1.02	2.0E-01	3.7E-01
K01844	beta-lysine 5,6-aminomutase alpha subunit [EC:5.4.3.3]	0.96	0.91	1.02	2.0E-01	3.7E-01
K02182	carnitine-CoA ligase [EC:6.2.1.48]	0.96	0.90	1.02	2.0E-01	3.7E-01
K00647	3-oxoacyl-[acyl-carrier-protein] synthase I [EC:2.3.1.41]	0.96	0.91	1.02	2.0E-01	3.7E-01
K00940	nucleoside-diphosphate kinase [EC:2.7.4.6]	1.04	0.98	1.11	2.0E-01	3.7E-01
K02687	ribosomal protein L11 methyltransferase [EC:2.1.1.-]	1.04	0.98	1.11	2.0E-01	3.7E-01
K13543	uroporphyrinogen III methyltransferase / synthase [EC:2.1.1.107 4.2.1.75]	0.96	0.91	1.02	2.0E-01	3.7E-01
K04479	DNA polymerase IV (archaeal DinB-like DNA polymerase) [EC:2.7.7.7]	0.96	0.91	1.02	2.0E-01	3.8E-01
K01347	IgA-specific serine endopeptidase [EC:3.4.21.72]	0.96	0.91	1.02	2.0E-01	3.8E-01
K07812	trimethylamine-N-oxide reductase (cytochrome c) [EC:1.7.2.3]	1.04	0.98	1.10	2.0E-01	3.8E-01
K11395	2-dehydro-3-deoxy-phosphogluconate/2-dehydro-3-deoxy-6-phosphogalactonate aldolase [EC:4.1.2.55]	0.96	0.91	1.02	2.0E-01	3.8E-01
K06122	glycerol dehydratase small subunit [EC:4.2.1.30]	0.96	0.91	1.02	2.0E-01	3.8E-01
K01523	phosphoribosyl-ATP pyrophosphohydrolase [EC:3.6.1.31]	0.96	0.91	1.02	2.0E-01	3.8E-01
K01813	L-rhamnose isomerase [EC:5.3.1.14]	0.96	0.91	1.02	2.0E-01	3.8E-01
K01809	mannose-6-phosphate isomerase [EC:5.3.1.8]	1.04	0.98	1.11	2.0E-01	3.8E-01
K00176	2-oxoglutarate ferredoxin oxidoreductase subunit delta [EC:1.2.7.3]	0.97	0.91	1.02	2.0E-01	3.8E-01
K09474	acid phosphatase (class A) [EC:3.1.3.2]	0.96	0.91	1.02	2.1E-01	3.9E-01
K08093	3-hexulose-6-phosphate synthase [EC:4.1.2.43]	0.96	0.91	1.02	2.1E-01	3.9E-01
K15923	alpha-L-fucosidase 2 [EC:3.2.1.51]	0.96	0.91	1.02	2.1E-01	3.9E-01
K01611	S-adenosylmethionine decarboxylase [EC:4.1.1.50]	1.04	0.98	1.10	2.1E-01	3.9E-01
K04787	mycobactin salicyl-AMP ligase [EC:6.3.2.-]	0.96	0.91	1.02	2.1E-01	3.9E-01
K01026	propionate CoA-transferase [EC:2.8.3.1]	0.96	0.90	1.02	2.1E-01	4.0E-01
K02115	F-type H+-transporting ATPase subunit gamma	0.96	0.91	1.02	2.1E-01	4.0E-01
K08728	nucleoside deoxyribosyltransferase [EC:2.4.2.6]	0.96	0.91	1.02	2.1E-01	4.0E-01
K01784	UDP-glucose 4-epimerase [EC:5.1.3.2]	1.04	0.98	1.10	2.1E-01	4.0E-01
K03896	acetyl CoA:N6-hydroxylysine acetyl transferase [EC:2.3.1.102]	1.04	0.98	1.10	2.1E-01	4.0E-01
K12552	penicillin-binding protein 1 [EC:3.4.-.-]	0.96	0.91	1.02	2.1E-01	4.0E-01
K15635	2,3-bisphosphoglycerate-independent phosphoglycerate mutase [EC:5.4.2.12]	0.97	0.91	1.02	2.1E-01	4.0E-01
K01815	4-deoxy-L-threo-5-hexosulose-uronate ketol-isomerase [EC:5.3.1.17]	0.96	0.91	1.02	2.1E-01	4.0E-01
K01515	ADP-ribose pyrophosphatase [EC:3.6.1.13]	1.04	0.98	1.10	2.2E-01	4.0E-01
K05825	2-aminoadipate transaminase [EC:2.6.1.-]	0.96	0.91	1.02	2.2E-01	4.0E-01
K05369	15,16-dihydrobiliverdin:ferredoxin oxidoreductase [EC:1.3.7.2]	0.96	0.91	1.02	2.2E-01	4.0E-01
K05356	all-trans-nonaprenyl-diphosphate synthase [EC:2.5.1.84 2.5.1.85]	0.96	0.91	1.02	2.2E-01	4.0E-01
K15226	arogenate dehydrogenase (NADP+) [EC:1.3.1.78]	0.96	0.91	1.02	2.2E-01	4.0E-01
K14331	fatty aldehyde decarbonylase [EC:4.1.99.5]	0.96	0.91	1.02	2.2E-01	4.0E-01
K14330	fatty aldehyde-generating acyl-ACP reductase [EC:1.2.1.80]	0.96	0.91	1.02	2.2E-01	4.0E-01
K05572	NAD(P)H-quinone oxidoreductase subunit 1 [EC:7.1.1.2]	0.96	0.91	1.02	2.2E-01	4.0E-01

Predictor	Description	HR	2.5% CI	97.5% CI	P	FDR-adjusted P
K05585	NAD(P)H-quinone oxidoreductase subunit N [EC:7.1.1.2]	0.96	0.91	1.02	2.2E-01	4.0E-01
K02288	phycocyanobilin lyase subunit alpha [EC:4.4.1.32]	0.96	0.91	1.02	2.2E-01	4.0E-01
K05370	phycoerythrobilin:ferredoxin oxidoreductase [EC:1.3.7.3]	0.96	0.91	1.02	2.2E-01	4.0E-01
K11520	two-component system, OmpR family, manganese sensing sensor histidine kinase [EC:2.7.13.3]	0.96	0.91	1.02	2.2E-01	4.0E-01
K03867	UDP-glucose:tetrahydrobiopterin glucosyltransferase [EC:2.4.1.-]	0.96	0.91	1.02	2.2E-01	4.0E-01
K05895	precorrin-6A/cobalt-precorrin-6A reductase [EC:1.3.1.54 1.3.1.106]	0.96	0.91	1.02	2.2E-01	4.0E-01
K03183	demethylmenaquinone methyltransferase / 2-methoxy-6-polyprenyl-1,4-benzoquinol methylase [EC:2.1.1.163 2.1.1.201]	1.04	0.98	1.11	2.2E-01	4.0E-01
K11912	serine/threonine-protein kinase PpkA [EC:2.7.11.1]	0.96	0.91	1.02	2.2E-01	4.0E-01
K00996	undecaprenyl-phosphate galactose phosphotransferase [EC:2.7.8.6]	0.96	0.91	1.02	2.2E-01	4.1E-01
K01676	fumarate hydratase, class I [EC:4.2.1.2]	1.04	0.98	1.11	2.2E-01	4.1E-01
K00966	mannose-1-phosphate guanylyltransferase [EC:2.7.7.13]	0.96	0.91	1.02	2.2E-01	4.1E-01
K03524	BirA family transcriptional regulator, biotin operon repressor / biotin---[acetyl-CoA-carboxylase] ligase [EC:6.3.4.15]	0.96	0.91	1.02	2.2E-01	4.1E-01
K01963	acetyl-CoA carboxylase carboxyl transferase subunit beta [EC:6.4.1.2 2.1.3.15]	0.96	0.91	1.02	2.2E-01	4.1E-01
K00221	alkylmercury lyase [EC:4.99.1.2]	0.96	0.91	1.02	2.2E-01	4.1E-01
K11527	two-component system, sensor histidine kinase and response regulator [EC:2.7.13.3]	0.97	0.92	1.02	2.2E-01	4.1E-01
K03332	fructan beta-fructosidase [EC:3.2.1.80]	1.04	0.98	1.10	2.2E-01	4.1E-01
K14744	prophage endopeptidase [EC:3.4.-.-]	1.04	0.98	1.10	2.3E-01	4.1E-01
K12447	UDP-sugar pyrophosphorylase [EC:2.7.7.64]	0.96	0.91	1.02	2.3E-01	4.1E-01
K05306	phosphonoacetaldehyde hydrolase [EC:3.11.1.1]	0.97	0.92	1.02	2.3E-01	4.1E-01
K07468	putative ATP-dependent DNA ligase [EC:6.5.1.1]	0.96	0.91	1.02	2.3E-01	4.1E-01
K03269	UDP-2,3-diacylglucosamine hydrolase [EC:3.6.1.54]	0.97	0.91	1.02	2.3E-01	4.1E-01
K00690	sucrose phosphorylase [EC:2.4.1.7]	0.96	0.91	1.02	2.3E-01	4.2E-01
K01023	arylsulfate sulfotransferase [EC:2.8.2.22]	0.97	0.91	1.02	2.3E-01	4.2E-01
K00003	homoserine dehydrogenase [EC:1.1.1.3]	0.96	0.91	1.02	2.3E-01	4.2E-01
K01628	L-fuculose-phosphate aldolase [EC:4.1.2.17]	0.96	0.91	1.02	2.3E-01	4.2E-01
K08307	membrane-bound lytic murein transglycosylase D [EC:4.2.2.-]	0.97	0.91	1.02	2.3E-01	4.2E-01
K01612	vanillate/4-hydroxybenzoate decarboxylase subunit C [EC:4.1.1.- 4.1.1.61]	0.96	0.91	1.02	2.3E-01	4.2E-01
K01446	peptidoglycan recognition protein	0.96	0.91	1.02	2.3E-01	4.2E-01
K12570	streptomycin 6-kinase [EC:2.7.1.72]	0.96	0.91	1.02	2.3E-01	4.2E-01
K02230	cobaltochelatase CobN [EC:6.6.1.2]	0.97	0.91	1.02	2.3E-01	4.2E-01
K00955	bifunctional enzyme CysN/CysC [EC:2.7.7.4 2.7.1.25]	0.96	0.91	1.02	2.3E-01	4.2E-01
K16558	succinoglycan biosynthesis protein ExoL [EC:2.-.-.-]	0.96	0.91	1.02	2.3E-01	4.2E-01
K00988	sulfate adenylyltransferase (ADP) / ATP adenylyltransferase [EC:2.7.7.5 2.7.7.53]	0.97	0.91	1.02	2.3E-01	4.2E-01
K04102	4,5-dihydroxyphthalate decarboxylase [EC:4.1.1.55]	0.96	0.91	1.02	2.3E-01	4.2E-01
K00134	glyceraldehyde 3-phosphate dehydrogenase (phosphorylating) [EC:1.2.1.12]	0.96	0.91	1.02	2.3E-01	4.2E-01
K05926	23S rRNA (adenosine1067-2'-O)-methyltransferase [EC:2.1.1.230]	0.96	0.91	1.02	2.3E-01	4.3E-01
K00028	malate dehydrogenase (decarboxylating) [EC:1.1.1.39]	1.04	0.98	1.10	2.3E-01	4.3E-01
K11717	cysteine desulfurase / selenocysteine lyase [EC:2.8.1.7 4.4.1.16]	0.96	0.91	1.02	2.3E-01	4.3E-01
K14157	alpha-aminoadipic semialdehyde synthase [EC:1.5.1.8 1.5.1.9]	1.04	0.98	1.10	2.4E-01	4.3E-01
K07304	peptide-methionine (S)-S-oxide reductase [EC:1.8.4.11]	0.97	0.91	1.02	2.4E-01	4.3E-01
K13074	biflaviolin synthase [EC:1.14.19.69]	0.97	0.91	1.02	2.4E-01	4.3E-01
K02169	malonyl-CoA O-methyltransferase [EC:2.1.1.197]	0.97	0.91	1.02	2.4E-01	4.3E-01
K01426	amidase [EC:3.5.1.4]	0.96	0.91	1.02	2.4E-01	4.3E-01
K00351	Na+-transporting NADH:ubiquinone oxidoreductase subunit F [EC:7.2.1.1]	0.97	0.92	1.02	2.4E-01	4.3E-01

Predictor	Description	HR	2.5% CI	97.5% CI	P	FDR-adjusted P
K14082	[methyl-Co(III) methylamine-specific corrinoid protein]:coenzyme M methyltransferase [EC:2.1.1.247]	0.96	0.91	1.02	2.4E-01	4.3E-01
K01818	L-fucose/D-arabinose isomerase [EC:5.3.1.25 5.3.1.3]	1.04	0.97	1.11	2.4E-01	4.3E-01
K01390	IgA-specific metalloendopeptidase [EC:3.4.24.13]	0.97	0.91	1.02	2.4E-01	4.3E-01
K00928	aspartate kinase [EC:2.7.2.4]	1.04	0.98	1.10	2.4E-01	4.3E-01
K05579	NAD(P)H-quinone oxidoreductase subunit H [EC:7.1.1.2]	0.96	0.91	1.02	2.4E-01	4.3E-01
K05581	NAD(P)H-quinone oxidoreductase subunit J [EC:7.1.1.2]	0.96	0.91	1.02	2.4E-01	4.3E-01
K05582	NAD(P)H-quinone oxidoreductase subunit K [EC:7.1.1.2]	0.96	0.91	1.02	2.4E-01	4.3E-01
K01089	imidazoleglycerol-phosphate dehydratase / histidinol-phosphatase [EC:4.2.1.19 3.1.3.15]	0.97	0.92	1.02	2.4E-01	4.3E-01
K00350	Na+-transporting NADH:ubiquinone oxidoreductase subunit E [EC:7.2.1.1]	0.97	0.92	1.02	2.4E-01	4.3E-01
K01356	repressor LexA [EC:3.4.21.88]	0.96	0.91	1.02	2.4E-01	4.3E-01
K02113	F-type H+-transporting ATPase subunit delta	0.97	0.91	1.02	2.4E-01	4.3E-01
K01491	methylenetetrahydrofolate dehydrogenase (NADP+) / methenyltetrahydrofolate cyclohydrolase [EC:1.5.1.5 3.5.4.9]	0.96	0.91	1.02	2.4E-01	4.3E-01
K13038	phosphopantothenoylcysteine decarboxylase / phosphopantothenate---cysteine ligase [EC:4.1.1.36 6.3.2.5]	1.04	0.98	1.10	2.4E-01	4.3E-01
K00675	N-hydroxyarylamine O-acetyltransferase [EC:2.3.1.118]	1.04	0.98	1.10	2.4E-01	4.3E-01
K13037	L-alanine-L-anticapsin ligase [EC:6.3.2.49]	0.96	0.91	1.02	2.4E-01	4.3E-01
K00651	homoserine O-succinyltransferase/O-acetyltransferase [EC:2.3.1.46 2.3.1.31]	0.97	0.91	1.02	2.4E-01	4.3E-01
K07309	Tat-targeted selenate reductase subunit YnfE [EC:1.97.1.9]	1.04	0.98	1.10	2.4E-01	4.3E-01
K01951	GMP synthase (glutamine-hydrolysing) [EC:6.3.5.2]	0.97	0.91	1.02	2.4E-01	4.4E-01
K00965	UDPglucose--hexose-1-phosphate uridylyltransferase [EC:2.7.7.12]	0.96	0.91	1.02	2.4E-01	4.4E-01
K11385	arabinosyltransferase A [EC:2.4.2.-]	0.97	0.91	1.02	2.4E-01	4.4E-01
K02506	leader peptidase HopD [EC:3.4.23.43]	1.04	0.98	1.10	2.4E-01	4.4E-01
K01120	3',5'-cyclic-nucleotide phosphodiesterase [EC:3.1.4.17]	0.97	0.91	1.02	2.5E-01	4.4E-01
K00298	N5-(carboxyethyl)ornithine synthase [EC:1.5.1.24]	0.97	0.91	1.02	2.5E-01	4.4E-01
K00013	histidinol dehydrogenase [EC:1.1.1.23]	1.04	0.97	1.11	2.5E-01	4.4E-01
K11381	2-oxoisovalerate dehydrogenase E1 component [EC:1.2.4.4]	1.04	0.98	1.10	2.5E-01	4.4E-01
K10915	CAI-1 autoinducer synthase [EC:2.3.-.-]	0.97	0.91	1.02	2.5E-01	4.4E-01
K14596	zeaxanthin glucosyltransferase [EC:2.4.1.276]	0.97	0.91	1.02	2.5E-01	4.4E-01
K06182	23S rRNA pseudouridine2604 synthase [EC:5.4.99.21]	0.97	0.91	1.03	2.5E-01	4.4E-01
K13051	L-asparaginase / beta-aspartyl-peptidase [EC:3.5.1.1 3.4.19.5]	0.97	0.91	1.02	2.5E-01	4.4E-01
K05364	penicillin-binding protein A	0.97	0.91	1.03	2.5E-01	4.5E-01
K08356	arsenite oxidase large subunit [EC:1.20.2.1 1.20.9.1]	0.97	0.91	1.02	2.5E-01	4.5E-01
K01971	bifunctional non-homologous end joining protein LigD [EC:6.5.1.1]	0.97	0.91	1.03	2.5E-01	4.5E-01
K03177	tRNA pseudouridine55 synthase [EC:5.4.99.25]	0.97	0.91	1.03	2.5E-01	4.5E-01
K04070	putative pyruvate formate lyase activating enzyme [EC:1.97.1.4]	0.97	0.91	1.03	2.5E-01	4.5E-01
K02111	F-type H+/Na+-transporting ATPase subunit alpha [EC:7.1.2.2 7.2.2.1]	0.97	0.91	1.02	2.5E-01	4.5E-01
K01271	Xaa-Pro dipeptidase [EC:3.4.13.9]	1.04	0.98	1.10	2.5E-01	4.5E-01
K11608	mycobacterial beta-ketoacyl-[acyl-carrier-protein] synthase III [EC:2.3.1.301]	0.97	0.91	1.02	2.6E-01	4.5E-01
K15373	sulfoacetaldehyde reductase [EC:1.1.1.313]	0.97	0.91	1.02	2.6E-01	4.5E-01
K08681	pyridoxal 5'-phosphate synthase pdxT subunit [EC:4.3.3.6]	0.97	0.91	1.02	2.6E-01	4.5E-01
K01091	phosphoglycolate phosphatase [EC:3.1.3.18]	1.03	0.98	1.10	2.6E-01	4.5E-01
K00129	aldehyde dehydrogenase (NAD(P)+) [EC:1.2.1.5]	1.03	0.98	1.09	2.6E-01	4.6E-01
K05525	linalool 8-monooxygenase [EC:1.14.14.84]	0.97	0.91	1.03	2.6E-01	4.6E-01
K07256	taurine dehydrogenase large subunit [EC:1.4.2.-]	0.97	0.91	1.03	2.6E-01	4.6E-01
K02114	F-type H+-transporting ATPase subunit epsilon	0.97	0.91	1.02	2.6E-01	4.6E-01

Predictor	Description	HR	2.5% CI	97.5% CI	P	FDR-adjusted P
K05606	methylmalonyl-CoA/ethylmalonyl-CoA epimerase [EC:5.1.99.1]	1.03	0.97	1.10	2.6E-01	4.6E-01
K00230	menaquinone-dependent protoporphyrinogen oxidase [EC:1.3.5.3]	0.97	0.91	1.03	2.6E-01	4.6E-01
K01629	rhamnulose-1-phosphate aldolase [EC:4.1.2.19]	0.97	0.91	1.03	2.6E-01	4.6E-01
K02314	replicative DNA helicase [EC:3.6.4.12]	0.97	0.91	1.03	2.6E-01	4.6E-01
K08261	D-sorbitol dehydrogenase (acceptor) [EC:1.1.99.21]	0.97	0.91	1.03	2.7E-01	4.7E-01
K00674	2,3,4,5-tetrahydropyridine-2,6-dicarboxylate N-succinyltransferase [EC:2.3.1.117]	0.97	0.91	1.03	2.7E-01	4.7E-01
K00152	salicylaldehyde dehydrogenase [EC:1.2.1.65]	0.97	0.91	1.03	2.7E-01	4.7E-01
K01207	beta-N-acetylhexosaminidase [EC:3.2.1.52]	0.97	0.91	1.03	2.7E-01	4.7E-01
K05985	ribonuclease M5 [EC:3.1.26.8]	0.97	0.91	1.03	2.7E-01	4.7E-01
K01745	histidine ammonia-lyase [EC:4.3.1.3]	0.97	0.92	1.02	2.7E-01	4.7E-01
K01159	crossover junction endodeoxyribonuclease RuvC [EC:3.1.21.10]	1.03	0.97	1.10	2.7E-01	4.7E-01
K15527	cysteate synthase [EC:2.5.1.76]	0.97	0.91	1.03	2.7E-01	4.7E-01
K00991	2-C-methyl-D-erythritol 4-phosphate cytidylyltransferase [EC:2.7.7.60]	0.97	0.91	1.03	2.7E-01	4.7E-01
K01640	hydroxymethylglutaryl-CoA lyase [EC:4.1.3.4]	0.97	0.91	1.03	2.7E-01	4.7E-01
K11755	phosphoribosyl-AMP cyclohydrolase / phosphoribosyl-ATP pyrophosphohydrolase [EC:3.5.4.19 3.6.1.31]	0.97	0.92	1.02	2.7E-01	4.7E-01
K11358	aspartate aminotransferase [EC:2.6.1.1]	0.97	0.91	1.03	2.7E-01	4.7E-01
K02427	23S rRNA (uridine2552-2'-O)-methyltransferase [EC:2.1.1.166]	0.97	0.91	1.03	2.7E-01	4.7E-01
K01267	aspartyl aminopeptidase [EC:3.4.11.21]	0.97	0.91	1.03	2.7E-01	4.8E-01
K02226	alpha-ribazole phosphatase [EC:3.1.3.73]	0.97	0.92	1.03	2.7E-01	4.8E-01
K01193	beta-fructofuranosidase [EC:3.2.1.26]	0.97	0.91	1.03	2.7E-01	4.8E-01
K03578	ATP-dependent helicase HrpA [EC:3.6.4.13]	0.97	0.91	1.03	2.7E-01	4.8E-01
K07646	two-component system, OmpR family, sensor histidine kinase KdpD [EC:2.7.13.3]	1.04	0.97	1.10	2.7E-01	4.8E-01
K00677	UDP-N-acetylglucosamine acyltransferase [EC:2.3.1.129]	1.04	0.97	1.10	2.7E-01	4.8E-01
K06045	squalene-hopene/tetraprenyl-beta-curcumene cyclase [EC:5.4.99.17 4.2.1.129]	0.97	0.91	1.03	2.7E-01	4.8E-01
K05576	NAD(P)H-quinone oxidoreductase subunit 4L [EC:7.1.1.2]	0.97	0.91	1.03	2.7E-01	4.8E-01
K07316	adenine-specific DNA-methyltransferase [EC:2.1.1.72]	1.03	0.97	1.10	2.8E-01	4.8E-01
K02852	UDP-N-acetyl-D-mannosaminouronate:lipid I N-acetyl-D-mannosaminouronosyltransferase [EC:2.4.1.180]	0.97	0.92	1.03	2.8E-01	4.8E-01
K07248	lactaldehyde dehydrogenase / glycolaldehyde dehydrogenase [EC:1.2.1.22 1.2.1.21]	0.97	0.91	1.03	2.8E-01	4.8E-01
K00691	maltose phosphorylase [EC:2.4.1.8]	0.97	0.91	1.03	2.8E-01	4.8E-01
K01666	4-hydroxy 2-oxovalerate aldolase [EC:4.1.3.39]	0.97	0.91	1.03	2.8E-01	4.8E-01
K01548	potassium-transporting ATPase KdpC subunit	1.03	0.97	1.10	2.8E-01	4.8E-01
K07674	two-component system, NarL family, nitrate/nitrite sensor histidine kinase NarQ [EC:2.7.13.3]	1.03	0.97	1.10	2.8E-01	4.8E-01
K01572	NA	0.97	0.92	1.03	2.8E-01	4.8E-01
K10944	methane/ammonia monooxygenase subunit A [EC:1.14.18.3 1.14.99.39]	0.97	0.91	1.03	2.8E-01	4.9E-01
K03426	NAD+ diphosphatase [EC:3.6.1.22]	0.97	0.92	1.03	2.8E-01	4.9E-01
K00603	glutamate formiminotransferase / 5-formyltetrahydrofolate cyclo-ligase [EC:2.1.2.5 6.3.3.2]	0.97	0.92	1.03	2.8E-01	4.9E-01
K03685	ribonuclease III [EC:3.1.26.3]	0.97	0.91	1.03	2.8E-01	4.9E-01
K15016	enoyl-CoA hydratase / 3-hydroxyacyl-CoA dehydrogenase [EC:4.2.1.17 1.1.1.35]	0.97	0.91	1.03	2.8E-01	4.9E-01
K15786	aspartate-semialdehyde dehydrogenase [EC:1.2.1.-]	0.97	0.91	1.03	2.8E-01	4.9E-01
K15036	acetyl-CoA/propionyl-CoA carboxylase [EC:6.4.1.2 6.4.1.3 2.1.3.15]	0.96	0.90	1.03	2.8E-01	4.9E-01
K08315	hydrogenase 3 maturation protease [EC:3.4.23.51]	0.97	0.91	1.03	2.8E-01	4.9E-01
K02435	aspartyl-tRNA(Asn)/glutamyl-tRNA(Gln) amidotransferase subunit C [EC:6.3.5.6 6.3.5.7]	0.97	0.91	1.03	2.8E-01	4.9E-01
K02118	V/A-type H+/Na+-transporting ATPase subunit B	0.97	0.92	1.03	2.9E-01	4.9E-01
K06176	tRNA pseudouridine13 synthase [EC:5.4.99.27]	0.97	0.91	1.03	2.9E-01	4.9E-01

Predictor	Description	HR	2.5% CI	97.5% CI	P	FDR-adjusted P
K02017	molybdate transport system ATP-binding protein [EC:7.3.2.5]	1.03	0.97	1.10	2.9E-01	4.9E-01
K02124	V/A-type H+/Na+-transporting ATPase subunit K	0.97	0.92	1.03	2.9E-01	4.9E-01
K13439	cysteine protease avirulence protein AvrRpt2 [EC:3.4.22.-]	0.97	0.91	1.03	2.9E-01	4.9E-01
K00757	uridine phosphorylase [EC:2.4.2.3]	0.97	0.92	1.03	2.9E-01	4.9E-01
K02858	3,4-dihydroxy 2-butanone 4-phosphate synthase [EC:4.1.99.12]	0.97	0.91	1.03	2.9E-01	4.9E-01
K01192	beta-mannosidase [EC:3.2.1.25]	0.97	0.92	1.03	2.9E-01	4.9E-01
K13927	holo-ACP synthase / triphosphoribosyl-dephospho-CoA synthase [EC:2.7.7.61 2.4.2.52]	0.97	0.91	1.03	2.9E-01	5.0E-01
K06042	precorrin-8X/cobalt-precorrin-8 methylmutase [EC:5.4.99.61 5.4.99.60]	0.97	0.91	1.03	2.9E-01	5.0E-01
K15017	malonyl-CoA/succinyl-CoA reductase (NADPH) [EC:1.2.1.75 1.2.1.76]	0.96	0.90	1.03	2.9E-01	5.0E-01
K15853	acyl transferase [EC:2.3.1.-]	0.97	0.91	1.03	2.9E-01	5.0E-01
K00231	protoporphyrinogen/coproporphyrinogen III oxidase [EC:1.3.3.4 1.3.3.15]	1.03	0.97	1.10	2.9E-01	5.0E-01
K13086	mannosylfructose-6-phosphate phosphatase [EC:3.1.3.79]	0.97	0.91	1.03	2.9E-01	5.0E-01
K01571	oxaloacetate decarboxylase (Na+ extruding) subunit alpha [EC:7.2.4.2]	0.97	0.91	1.03	2.9E-01	5.0E-01
K01895	acetyl-CoA synthetase [EC:6.2.1.1]	0.97	0.92	1.03	2.9E-01	5.0E-01
K10353	deoxyadenosine kinase [EC:2.7.1.76]	0.97	0.91	1.03	3.0E-01	5.0E-01
K00324	H+-translocating NAD(P) transhydrogenase subunit alpha [EC:1.6.1.2 7.1.1.1]	0.97	0.91	1.03	3.0E-01	5.0E-01
K01646	citrate lyase subunit gamma (acyl carrier protein)	1.03	0.97	1.10	3.0E-01	5.0E-01
K04487	cysteine desulfurase [EC:2.8.1.7]	0.97	0.91	1.03	3.0E-01	5.0E-01
K08884	serine/threonine protein kinase, bacterial [EC:2.7.11.1]	0.97	0.91	1.03	3.0E-01	5.1E-01
K01482	dimethylargininase [EC:3.5.3.18]	0.97	0.91	1.03	3.0E-01	5.1E-01
K11610	beta-ketoacyl ACP reductase [EC:1.1.1.100]	0.97	0.91	1.03	3.0E-01	5.1E-01
K08640	zinc D-Ala-D-Ala carboxypeptidase [EC:3.4.17.14]	0.97	0.91	1.03	3.0E-01	5.1E-01
K00185	dimethyl sulfoxide reductase membrane subunit	0.97	0.91	1.03	3.0E-01	5.1E-01
K00689	dextransucrase [EC:2.4.1.5]	0.97	0.92	1.03	3.0E-01	5.1E-01
K01673	carbonic anhydrase [EC:4.2.1.1]	1.03	0.97	1.10	3.0E-01	5.1E-01
K07404	6-phosphogluconolactonase [EC:3.1.1.31]	0.97	0.91	1.03	3.0E-01	5.1E-01
K01643	citrate lyase subunit alpha / citrate CoA-transferase [EC:2.8.3.10]	1.03	0.97	1.09	3.0E-01	5.1E-01
K01489	cytidine deaminase [EC:3.5.4.5]	0.97	0.92	1.03	3.0E-01	5.1E-01
K12466	(+)-trans-carveol dehydrogenase [EC:1.1.1.275]	0.97	0.91	1.03	3.1E-01	5.2E-01
K08282	non-specific serine/threonine protein kinase [EC:2.7.11.1]	0.97	0.91	1.03	3.1E-01	5.2E-01
K00797	spermidine synthase [EC:2.5.1.16]	0.97	0.91	1.03	3.1E-01	5.2E-01
K15915	undecaprenyl phosphate N,N'-diacetylbacillosamine 1-phosphate transferase [EC:2.7.8.36]	0.97	0.91	1.03	3.1E-01	5.2E-01
K15914	N,N'-diacetylbacillosaminyl-diphospho-undecaprenol alpha-1,3-N-acetylgalactosaminyltransferase [EC:2.4.1.290]	0.97	0.91	1.03	3.1E-01	5.2E-01
K00862	erythritol kinase (D-erythritol 1-phosphate-forming) [EC:2.7.1.215]	0.97	0.91	1.03	3.1E-01	5.2E-01
K01239	purine nucleosidase [EC:3.2.2.1]	0.97	0.91	1.03	3.1E-01	5.2E-01
K03534	L-rhamnose mutarotase [EC:5.1.3.32]	0.97	0.92	1.03	3.1E-01	5.2E-01
K12978	lipid A 4'-phosphatase [EC:3.1.3.-]	1.03	0.97	1.09	3.1E-01	5.2E-01
K02609	ring-1,2-phenylacetyl-CoA epoxidase subunit PaaA [EC:1.14.13.149]	0.97	0.91	1.03	3.1E-01	5.2E-01
K03587	cell division protein FtsI (penicillin-binding protein 3) [EC:3.4.16.4]	0.97	0.92	1.03	3.1E-01	5.2E-01
K06131	cardiolipin synthase A/B [EC:2.7.8.-]	0.97	0.92	1.03	3.1E-01	5.2E-01
K03206	azobenzene reductase [EC:1.7.1.6]	0.97	0.91	1.03	3.1E-01	5.2E-01
K03270	3-deoxy-D-manno-octulosonate 8-phosphate phosphatase (KDO 8-P phosphatase) [EC:3.1.3.45]	0.97	0.92	1.03	3.1E-01	5.2E-01
K04069	pyruvate formate lyase activating enzyme [EC:1.97.1.4]	0.97	0.92	1.03	3.1E-01	5.3E-01
K03081	NA	1.03	0.97	1.09	3.1E-01	5.3E-01

Predictor	Description	HR	2.5% CI	97.5% CI	P	FDR-adjusted P
K02412	flagellum-specific ATP synthase [EC:7.4.2.8]	0.97	0.91	1.03	3.1E-01	5.3E-01
K15064	syringate O-demethylase [EC:2.1.1.-]	0.97	0.91	1.03	3.2E-01	5.3E-01
K05878	phosphoenolpyruvate---glycerone phosphotransferase subunit DhaK [EC:2.7.1.121]	1.03	0.97	1.09	3.2E-01	5.3E-01
K05989	alpha-L-rhamnosidase [EC:3.2.1.40]	0.97	0.92	1.03	3.2E-01	5.3E-01
K01496	phosphoribosyl-AMP cyclohydrolase [EC:3.5.4.19]	0.97	0.91	1.03	3.2E-01	5.3E-01
K00737	beta-1,4-mannosyl-glycoprotein beta-1,4-N-acetylglucosaminyltransferase [EC:2.4.1.144]	0.97	0.91	1.03	3.2E-01	5.3E-01
K00850	6-phosphofructokinase 1 [EC:2.7.1.11]	0.97	0.92	1.03	3.2E-01	5.3E-01
K01315	plasminogen [EC:3.4.21.7]	0.97	0.91	1.03	3.2E-01	5.3E-01
K12500	thioesterase III [EC:3.1.2.-]	1.03	0.97	1.09	3.2E-01	5.4E-01
K00945	CMP/dCMP kinase [EC:2.7.4.25]	1.03	0.97	1.10	3.2E-01	5.4E-01
K00145	N-acetyl-gamma-glutamyl-phosphate reductase [EC:1.2.1.38]	1.03	0.97	1.10	3.2E-01	5.4E-01
K02759	cellobiose PTS system EIIA component [EC:2.7.1.196 2.7.1.205]	0.97	0.91	1.03	3.2E-01	5.4E-01
K01715	enoyl-CoA hydratase [EC:4.2.1.17]	0.97	0.91	1.03	3.2E-01	5.4E-01
K14742	tRNA threonylcarbamoyladenosine biosynthesis protein TsaB	0.97	0.92	1.03	3.2E-01	5.4E-01
K01185	lysozyme [EC:3.2.1.17]	1.03	0.97	1.09	3.2E-01	5.4E-01
K02822	ascorbate PTS system EIIB component [EC:2.7.1.194]	1.03	0.97	1.09	3.2E-01	5.4E-01
K06969	23S rRNA (cytosine1962-C5)-methyltransferase [EC:2.1.1.191]	1.03	0.97	1.09	3.2E-01	5.4E-01
K02203	phosphoserine / homoserine phosphotransferase [EC:3.1.3.3 2.7.1.39]	0.97	0.91	1.03	3.2E-01	5.4E-01
K00885	N-acylmannosamine kinase [EC:2.7.1.60]	1.03	0.97	1.09	3.2E-01	5.4E-01
K15450	tRNA wybutosine-synthesizing protein 3 [EC:2.1.1.282]	0.97	0.91	1.03	3.2E-01	5.4E-01
K01115	phospholipase D1/2 [EC:3.1.4.4]	0.97	0.92	1.03	3.3E-01	5.4E-01
K03789	[ribosomal protein S18]-alanine N-acetyltransferase [EC:2.3.1.266]	0.97	0.91	1.03	3.3E-01	5.4E-01
K07319	adenine-specific DNA-methyltransferase [EC:2.1.1.72]	1.03	0.97	1.09	3.3E-01	5.4E-01
K07306	anaerobic dimethyl sulfoxide reductase subunit A [EC:1.8.5.3]	1.03	0.97	1.09	3.3E-01	5.4E-01
K09457	7-cyano-7-deazaguanine reductase [EC:1.7.1.13]	0.97	0.92	1.03	3.3E-01	5.4E-01
K03215	23S rRNA (uracil1939-C5)-methyltransferase [EC:2.1.1.190]	0.97	0.92	1.03	3.3E-01	5.4E-01
K00366	ferredoxin-nitrite reductase [EC:1.7.7.1]	0.97	0.91	1.03	3.3E-01	5.4E-01
K03819	putative colanic acid biosynthesis acetyltransferase WcaB [EC:2.3.1.-]	1.03	0.97	1.09	3.3E-01	5.4E-01
K01573	oxaloacetate decarboxylase (Na+ extruding) subunit gamma	0.97	0.92	1.03	3.3E-01	5.4E-01
K14468	malonyl-CoA reductase / 3-hydroxypropionate dehydrogenase (NADP+) [EC:1.2.1.75 1.1.1.298]	0.97	0.91	1.03	3.3E-01	5.4E-01
K15052	propionyl-CoA carboxylase [EC:6.4.1.3 2.1.3.15]	0.97	0.91	1.03	3.3E-01	5.4E-01
K14472	succinyl-CoA:(S)-malate CoA-transferase subunit B [EC:2.8.3.22]	0.97	0.91	1.03	3.3E-01	5.4E-01
K01450	NA	0.97	0.91	1.03	3.3E-01	5.4E-01
K01841	phosphoenolpyruvate phosphomutase [EC:5.4.2.9]	1.03	0.97	1.09	3.3E-01	5.4E-01
K09459	phosphonopyruvate decarboxylase [EC:4.1.1.82]	1.03	0.97	1.09	3.3E-01	5.4E-01
K01582	lysine decarboxylase [EC:4.1.1.18]	1.03	0.97	1.09	3.3E-01	5.4E-01
K01840	phosphomannomutase [EC:5.4.2.8]	0.97	0.92	1.03	3.3E-01	5.4E-01
K12141	hydrogenase-4 component F [EC:1.-.-.-]	0.97	0.92	1.03	3.3E-01	5.4E-01
K00895	diphosphate-dependent phosphofructokinase [EC:2.7.1.90]	0.97	0.92	1.03	3.3E-01	5.4E-01
K03941	NADH dehydrogenase (ubiquinone) Fe-S protein 8 [EC:7.1.1.2]	0.97	0.91	1.03	3.3E-01	5.4E-01
K01592	tyrosine decarboxylase [EC:4.1.1.25]	1.03	0.97	1.09	3.3E-01	5.5E-01
K01433	formyltetrahydrofolate deformylase [EC:3.5.1.10]	0.97	0.92	1.03	3.4E-01	5.5E-01
K13727	phenolic acid decarboxylase [EC:4.1.1.-]	0.97	0.91	1.03	3.4E-01	5.5E-01
K00974	tRNA nucleotidyltransferase (CCA-adding enzyme) [EC:2.7.7.72 3.1.3.- 3.1.4.-]	0.97	0.91	1.03	3.4E-01	5.5E-01

Predictor	Description	HR	2.5% CI	97.5% CI	P	FDR-adjusted P
K01922	phosphopantothenate---cysteine ligase (ATP) [EC:6.3.2.51]	0.97	0.91	1.03	3.4E-01	5.5E-01
K01202	galactosylceramidase [EC:3.2.1.46]	0.97	0.91	1.03	3.4E-01	5.5E-01
K01142	exodeoxyribonuclease III [EC:3.1.11.2]	0.97	0.92	1.03	3.4E-01	5.5E-01
K08838	serine/threonine-protein kinase 24/25/MST4 [EC:2.7.11.1]	0.97	0.91	1.03	3.4E-01	5.5E-01
K00899	5-methylthioribose kinase [EC:2.7.1.100]	0.97	0.91	1.03	3.4E-01	5.5E-01
K08317	hydroxycarboxylate dehydrogenase A [EC:1.1.1.-]	1.03	0.97	1.09	3.4E-01	5.5E-01
K03767	peptidyl-prolyl cis-trans isomerase A (cyclophilin A) [EC:5.2.1.8]	0.97	0.91	1.03	3.4E-01	5.5E-01
K01085	glucose-1-phosphatase [EC:3.1.3.10]	1.03	0.97	1.09	3.4E-01	5.5E-01
K02821	ascorbate PTS system EIIA or EIIAB component [EC:2.7.1.194]	1.03	0.97	1.09	3.4E-01	5.6E-01
K08566	plasminogen activator [EC:3.4.23.48]	1.03	0.97	1.09	3.4E-01	5.6E-01
K01919	glutamate--cysteine ligase [EC:6.3.2.2]	1.03	0.97	1.09	3.4E-01	5.6E-01
K00693	glycogen synthase [EC:2.4.1.11]	0.97	0.92	1.03	3.4E-01	5.6E-01
K07698	two-component system, sporulation sensor kinase C [EC:2.7.13.3]	0.97	0.92	1.03	3.4E-01	5.6E-01
K13747	carboxynorspermidine decarboxylase [EC:4.1.1.96]	0.97	0.92	1.03	3.4E-01	5.6E-01
K00759	adenine phosphoribosyltransferase [EC:2.4.2.7]	0.97	0.92	1.03	3.4E-01	5.6E-01
K00290	saccharopine dehydrogenase (NAD+, L-lysine forming) [EC:1.5.1.7]	0.97	0.92	1.03	3.4E-01	5.6E-01
K12140	hydrogenase-4 component E [EC:1.-.-.-]	0.97	0.92	1.03	3.5E-01	5.6E-01
K04835	methylaspartate ammonia-lyase [EC:4.3.1.2]	1.03	0.97	1.09	3.5E-01	5.6E-01
K08969	L-glutamine---4-(methylsulfanyl)-2-oxobutanoate aminotransferase [EC:2.6.1.117]	0.97	0.91	1.03	3.5E-01	5.6E-01
K01487	guanine deaminase [EC:3.5.4.3]	1.03	0.97	1.09	3.5E-01	5.6E-01
K15038	succinyl-CoA reductase [EC:1.2.1.76]	0.97	0.91	1.03	3.5E-01	5.6E-01
K11066	N-acetylmuramoyl-L-alanine amidase [EC:3.5.1.28]	1.03	0.97	1.09	3.5E-01	5.6E-01
K08320	(d)CTP diphosphatase [EC:3.6.1.65]	1.03	0.97	1.09	3.5E-01	5.6E-01
K01952	phosphoribosylformylglycinamidine synthase [EC:6.3.5.3]	0.97	0.91	1.03	3.5E-01	5.6E-01
K12991	rhamnosyltransferase [EC:2.4.1.-]	0.97	0.92	1.03	3.5E-01	5.6E-01
K05711	2,3-dihydroxy-2,3-dihydrophenylpropionate dehydrogenase [EC:1.3.1.87]	1.03	0.97	1.09	3.5E-01	5.6E-01
K00288	methylenetetrahydrofolate dehydrogenase (NADP+) / methenyltetrahydrofolate cyclohydrolase / formyltetrahydrofolate synthetase [EC:1.5.1.5 3.5.4.9 6.3.4.3]	0.97	0.92	1.03	3.5E-01	5.6E-01
K01144	NA	0.97	0.92	1.03	3.5E-01	5.6E-01
K00286	pyrroline-5-carboxylate reductase [EC:1.5.1.2]	0.97	0.92	1.03	3.5E-01	5.6E-01
K01731	pectate disaccharide-lyase [EC:4.2.2.9]	0.97	0.92	1.03	3.5E-01	5.6E-01
K15912	UDP-N-acetylglucosamine 4,6-dehydratase [EC:4.2.1.135]	0.97	0.92	1.03	3.5E-01	5.6E-01
K07679	two-component system, NarL family, sensor histidine kinase EvgS [EC:2.7.13.3]	1.03	0.97	1.09	3.5E-01	5.6E-01
K04771	serine protease Do [EC:3.4.21.107]	0.97	0.92	1.03	3.5E-01	5.6E-01
K01752	L-serine dehydratase [EC:4.3.1.17]	1.03	0.97	1.09	3.5E-01	5.6E-01
K03278	UDP-D-galactose:(glucosyl)LPS alpha-1,3-D-galactosyltransferase [EC:2.4.1.44]	0.97	0.92	1.03	3.5E-01	5.6E-01
K00613	glycine amidinotransferase [EC:2.1.4.1]	0.97	0.91	1.03	3.5E-01	5.7E-01
K01079	phosphoserine phosphatase [EC:3.1.3.3]	0.97	0.92	1.03	3.5E-01	5.7E-01
K01492	phosphoribosylglycinamide/phosphoribosylaminoimidazolecarboxamide formyltransferase [EC:2.1.2.2 2.1.2.3]	0.97	0.92	1.03	3.5E-01	5.7E-01
K00692	levansucrase [EC:2.4.1.10]	0.97	0.91	1.03	3.5E-01	5.7E-01
K05358	quinate dehydrogenase (quinone) [EC:1.1.5.8]	0.97	0.92	1.03	3.5E-01	5.7E-01
K01093	4-phytase / acid phosphatase [EC:3.1.3.26 3.1.3.2]	1.03	0.97	1.09	3.6E-01	5.7E-01
K03079	L-ribulose-5-phosphate 3-epimerase [EC:5.1.3.22]	1.03	0.97	1.09	3.6E-01	5.7E-01
K01825	3-hydroxyacyl-CoA dehydrogenase / enoyl-CoA hydratase / 3-hydroxybutyryl-CoA epimerase / enoyl-CoA isomerase [EC:1.1.1.35 4.2.1.17 5.1.2.3 5.3.3.8]	1.03	0.97	1.09	3.6E-01	5.7E-01
K01042	L-seryl-tRNA(Ser) seleniumtransferase [EC:2.9.1.1]	1.03	0.97	1.09	3.6E-01	5.7E-01

Predictor	Description	HR	2.5% CI	97.5% CI	P	FDR-adjusted P
K00657	diamine N-acetyltransferase [EC:2.3.1.57]	0.97	0.92	1.03	3.6E-01	5.7E-01
K15910	UDP-N-acetylbacillosamine transaminase [EC:2.6.1.34]	0.97	0.92	1.03	3.6E-01	5.7E-01
K06269	serine/threonine-protein phosphatase PP1 catalytic subunit [EC:3.1.3.16]	1.03	0.97	1.08	3.6E-01	5.7E-01
K01598	phosphopantothenoylcysteine decarboxylase [EC:4.1.1.36]	0.97	0.92	1.03	3.6E-01	5.7E-01
K00325	H+-translocating NAD(P) transhydrogenase subunit beta [EC:1.6.1.2 7.1.1.1]	0.97	0.92	1.03	3.6E-01	5.7E-01
K05851	adenylate cyclase, class 1 [EC:4.6.1.1]	1.03	0.97	1.09	3.6E-01	5.8E-01
K11194	fructose PTS system EIIA component [EC:2.7.1.202]	0.97	0.92	1.03	3.6E-01	5.8E-01
K11195	fructose PTS system EIIB component [EC:2.7.1.202]	0.97	0.92	1.03	3.6E-01	5.8E-01
K06180	23S rRNA pseudouridine1911/1915/1917 synthase [EC:5.4.99.23]	0.97	0.92	1.03	3.6E-01	5.8E-01
K03895	aerobactin synthase [EC:6.3.2.39]	1.03	0.97	1.09	3.6E-01	5.8E-01
K01119	2',3'-cyclic-nucleotide 2'-phosphodiesterase / 3'-nucleotidase [EC:3.1.4.16 3.1.3.6]	0.97	0.92	1.03	3.6E-01	5.8E-01
K07811	trimethylamine-N-oxide reductase (cytochrome c) [EC:1.7.2.3]	1.03	0.97	1.09	3.6E-01	5.8E-01
K01096	phosphatidylglycerophosphatase B [EC:3.1.3.27 3.1.3.81 3.1.3.4 3.6.1.27]	1.03	0.97	1.09	3.6E-01	5.8E-01
K05966	triphosphoribosyl-dephospho-CoA synthase [EC:2.4.2.52]	0.97	0.92	1.03	3.6E-01	5.8E-01
K00189	2-oxoisovalerate/pyruvate ferredoxin oxidoreductase gamma subunit [EC:1.2.7.7 1.2.7.1]	1.03	0.97	1.09	3.6E-01	5.8E-01
K00990	[protein-PII] uridylyltransferase [EC:2.7.7.59]	0.97	0.92	1.03	3.7E-01	5.8E-01
K01914	aspartate--ammonia ligase [EC:6.3.1.1]	0.97	0.92	1.03	3.7E-01	5.8E-01
K02079	N-acetylgalactosamine-6-phosphate deacetylase [EC:3.5.1.25]	1.03	0.97	1.09	3.7E-01	5.8E-01
K12567	titin [EC:2.7.11.1]	1.03	0.97	1.09	3.7E-01	5.8E-01
K01929	UDP-N-acetylmuramoyl-tripeptide--D-alanyl-D-alanine ligase [EC:6.3.2.10]	0.97	0.92	1.03	3.7E-01	5.8E-01
K02434	aspartyl-tRNA(Asn)/glutamyl-tRNA(Gln) amidotransferase subunit B [EC:6.3.5.6 6.3.5.7]	0.97	0.92	1.03	3.7E-01	5.8E-01
K00892	inosine kinase [EC:2.7.1.73]	1.03	0.97	1.09	3.7E-01	5.8E-01
K03635	molybdopterin synthase catalytic subunit [EC:2.8.1.12]	0.97	0.92	1.03	3.7E-01	5.8E-01
K03476	L-ascorbate 6-phosphate lactonase [EC:3.1.1.-]	1.03	0.97	1.09	3.7E-01	5.8E-01
K08308	membrane-bound lytic murein transglycosylase E [EC:4.2.2.-]	1.03	0.97	1.09	3.7E-01	5.8E-01
K15777	4,5-DOPA dioxygenase extradiol [EC:1.13.11.-]	1.03	0.97	1.09	3.7E-01	5.8E-01
K13656	undecaprenyl-phosphate glucose phosphotransferase [EC:2.7.8.31]	0.97	0.92	1.03	3.7E-01	5.8E-01
K02611	ring-1,2-phenylacetyl-CoA epoxidase subunit PaaC [EC:1.14.13.149]	0.97	0.92	1.03	3.7E-01	5.8E-01
K01247	DNA-3-methyladenine glycosylase II [EC:3.2.2.21]	1.03	0.97	1.09	3.7E-01	5.8E-01
K03736	ethanolamine ammonia-lyase small subunit [EC:4.3.1.7]	0.97	0.92	1.03	3.7E-01	5.8E-01
K08303	U32 family peptidase [EC:3.4.-.-]	1.03	0.97	1.10	3.7E-01	5.8E-01
K06153	undecaprenyl-diphosphatase [EC:3.6.1.27]	1.03	0.97	1.09	3.7E-01	5.8E-01
K01910	[citrate (pro-3S)-lyase] ligase [EC:6.2.1.22]	1.03	0.97	1.09	3.7E-01	5.8E-01
K02363	2,3-dihydroxybenzoate---[aryl-carrier protein] ligase [EC:6.3.2.14 6.2.1.71]	0.97	0.92	1.03	3.7E-01	5.8E-01
K01960	pyruvate carboxylase subunit B [EC:6.4.1.1]	0.97	0.92	1.03	3.7E-01	5.8E-01
K01872	alanyl-tRNA synthetase [EC:6.1.1.7]	1.03	0.97	1.09	3.7E-01	5.8E-01
K04035	magnesium-protoporphyrin IX monomethyl ester (oxidative) cyclase [EC:1.14.13.81]	0.97	0.92	1.03	3.7E-01	5.8E-01
K11441	dehydrogluconokinase [EC:2.7.1.13]	0.97	0.92	1.03	3.7E-01	5.8E-01
K12944	nucleoside triphosphatase [EC:3.6.1.-]	1.03	0.97	1.09	3.7E-01	5.8E-01
K00111	glycerol-3-phosphate dehydrogenase [EC:1.1.5.3]	0.97	0.92	1.03	3.7E-01	5.8E-01
K15320	6-methylsalicylic acid synthase [EC:2.3.1.165]	0.97	0.92	1.03	3.7E-01	5.8E-01
K04565	superoxide dismutase, Cu-Zn family [EC:1.15.1.1]	0.97	0.92	1.03	3.7E-01	5.8E-01
K03894	N2-citryl-N6-acetyl-N6-hydroxylysine synthase [EC:6.3.2.38]	1.03	0.97	1.09	3.7E-01	5.8E-01
K02798	mannitol PTS system EIIA component [EC:2.7.1.197]	1.03	0.97	1.09	3.7E-01	5.8E-01

Predictor	Description	HR	2.5% CI	97.5% CI	P	FDR-adjusted P
K03473	erythronate-4-phosphate dehydrogenase [EC:1.1.1.290]	0.97	0.92	1.03	3.7E-01	5.8E-01
K08348	formate dehydrogenase-N, alpha subunit [EC:1.17.5.3]	1.03	0.97	1.09	3.8E-01	5.9E-01
K10805	acyl-CoA thioesterase II [EC:3.1.2.-]	0.97	0.92	1.03	3.8E-01	5.9E-01
K01150	deoxyribonuclease I [EC:3.1.21.1]	1.03	0.97	1.09	3.8E-01	5.9E-01
K00012	UDPglucose 6-dehydrogenase [EC:1.1.1.22]	1.03	0.97	1.09	3.8E-01	5.9E-01
K12984	(heptosyl)LPS beta-1,4-glucosyltransferase [EC:2.4.1.-]	0.97	0.92	1.03	3.8E-01	5.9E-01
K09758	aspartate 4-decarboxylase [EC:4.1.1.12]	0.97	0.92	1.03	3.8E-01	5.9E-01
K03752	molybdenum cofactor guanylyltransferase [EC:2.7.7.77]	0.97	0.92	1.03	3.8E-01	5.9E-01
K01312	trypsin [EC:3.4.21.4]	0.97	0.92	1.03	3.8E-01	5.9E-01
K02013	iron complex transport system ATP-binding protein [EC:7.2.2.-]	0.97	0.92	1.03	3.8E-01	5.9E-01
K12973	lipid IVA palmitoyltransferase [EC:2.3.1.251]	1.03	0.97	1.09	3.8E-01	5.9E-01
K00791	tRNA dimethylallyltransferase [EC:2.5.1.75]	0.98	0.92	1.03	3.8E-01	5.9E-01
K01337	lysyl endopeptidase [EC:3.4.21.50]	1.03	0.97	1.09	3.8E-01	5.9E-01
K08319	L-threonate 2-dehydrogenase [EC:1.1.1.411]	0.97	0.92	1.03	3.8E-01	5.9E-01
K03184	3-demethoxyubiquinol 3-hydroxylase [EC:1.14.99.60]	1.03	0.97	1.09	3.8E-01	5.9E-01
K03817	ribosomal-protein-serine acetyltransferase [EC:2.3.1.-]	0.97	0.92	1.03	3.8E-01	5.9E-01
K11529	glycerate 2-kinase [EC:2.7.1.165]	0.97	0.92	1.03	3.8E-01	5.9E-01
K09020	ureidoacrylate peracid hydrolase [EC:3.5.1.110]	1.03	0.97	1.09	3.8E-01	5.9E-01
K07639	two-component system, OmpR family, sensor histidine kinase RstB [EC:2.7.13.3]	1.03	0.97	1.09	3.8E-01	5.9E-01
K02372	3-hydroxyacyl-[acyl-carrier-protein] dehydratase [EC:4.2.1.59]	0.97	0.92	1.03	3.8E-01	5.9E-01
K01073	acyl-CoA hydrolase [EC:3.1.2.20]	0.97	0.92	1.03	3.8E-01	5.9E-01
K01077	alkaline phosphatase [EC:3.1.3.1]	1.03	0.97	1.09	3.8E-01	5.9E-01
K00801	farnesyl-diphosphate farnesyltransferase [EC:2.5.1.21]	0.97	0.92	1.03	3.8E-01	5.9E-01
K07637	two-component system, OmpR family, sensor histidine kinase PhoQ [EC:2.7.13.3]	1.03	0.97	1.09	3.8E-01	5.9E-01
K14051	c-di-GMP phosphodiesterase Gmr [EC:3.1.4.52]	1.03	0.97	1.09	3.8E-01	5.9E-01
K05526	succinylglutamate desuccinylase [EC:3.5.1.96]	1.03	0.97	1.09	3.8E-01	5.9E-01
K13788	phosphate acetyltransferase [EC:2.3.1.8]	0.97	0.92	1.03	3.8E-01	5.9E-01
K03621	phosphate acyltransferase [EC:2.3.1.274]	0.97	0.92	1.03	3.9E-01	5.9E-01
K16559	endo-1,3-1,4-beta-glycanase ExoK [EC:3.2.1.-]	0.97	0.92	1.03	3.9E-01	5.9E-01
K01443	N-acetylglucosamine-6-phosphate deacetylase [EC:3.5.1.25]	1.03	0.97	1.09	3.9E-01	5.9E-01
K07589	D-erythro-7,8-dihydroneopterin triphosphate epimerase [EC:5.1.99.7]	1.03	0.97	1.09	3.9E-01	6.0E-01
K05709	3-phenylpropionate/trans-cinnamate dioxygenase subunit beta [EC:1.14.12.19]	1.03	0.97	1.09	3.9E-01	6.0E-01
K05970	sialate O-acetylesterase [EC:3.1.1.53]	0.98	0.92	1.03	3.9E-01	6.0E-01
K06074	vitamin B12 transport system ATP-binding protein [EC:7.6.2.8]	1.03	0.97	1.09	3.9E-01	6.0E-01
K11392	16S rRNA (cytosine1407-C5)-methyltransferase [EC:2.1.1.178]	1.03	0.97	1.09	3.9E-01	6.0E-01
K02336	DNA polymerase II [EC:2.7.7.7]	1.03	0.97	1.09	3.9E-01	6.0E-01
K12661	L-rhamnonate dehydratase [EC:4.2.1.90]	0.97	0.92	1.03	3.9E-01	6.0E-01
K01250	pyrimidine-specific ribonucleoside hydrolase [EC:3.2.-.-]	1.03	0.97	1.09	3.9E-01	6.0E-01
K02760	cellobiose PTS system EIIB component [EC:2.7.1.196 2.7.1.205]	0.97	0.92	1.03	3.9E-01	6.0E-01
K00963	UTP--glucose-1-phosphate uridylyltransferase [EC:2.7.7.9]	1.03	0.97	1.09	3.9E-01	6.0E-01
K03897	lysine N6-hydroxylase [EC:1.14.13.59]	1.03	0.97	1.09	3.9E-01	6.0E-01
K05368	NAD(P)H-flavin reductase [EC:1.5.1.41]	1.03	0.97	1.09	3.9E-01	6.0E-01
K02778	glucose PTS system EIIB component [EC:2.7.1.199]	1.03	0.97	1.09	3.9E-01	6.0E-01
K01706	glucarate dehydratase [EC:4.2.1.40]	1.03	0.97	1.09	3.9E-01	6.0E-01

Predictor	Description	HR	2.5% CI	97.5% CI	P	FDR-adjusted P
K00040	fructuronate reductase [EC:1.1.1.57]	0.97	0.92	1.03	3.9E-01	6.0E-01
K15566	tRNA (adenine9-N1/guanine9-N1)-methyltransferase [EC:2.1.1.218 2.1.1.221]	0.97	0.92	1.03	4.0E-01	6.0E-01
K00009	mannitol-1-phosphate 5-dehydrogenase [EC:1.1.1.17]	1.03	0.97	1.09	4.0E-01	6.0E-01
K02484	two-component system, OmpR family, sensor kinase [EC:2.7.13.3]	0.98	0.92	1.03	4.0E-01	6.0E-01
K01470	creatinine amidohydrolase [EC:3.5.2.10]	0.98	0.92	1.03	4.0E-01	6.0E-01
K02304	precorrin-2 dehydrogenase / sirohydrochlorin ferrochelatase [EC:1.3.1.76 4.99.1.4]	0.97	0.92	1.03	4.0E-01	6.0E-01
K00184	dimethyl sulfoxide reductase iron-sulfur subunit	0.97	0.92	1.04	4.0E-01	6.0E-01
K07026	mannosyl-3-phosphoglycerate phosphatase [EC:3.1.3.70]	1.03	0.97	1.09	4.0E-01	6.0E-01
K10621	2,3-dihydroxy-p-cumate/2,3-dihydroxybenzoate 3,4-dioxygenase [EC:1.13.11.- 1.13.11.14]	0.98	0.92	1.03	4.0E-01	6.0E-01
K00016	L-lactate dehydrogenase [EC:1.1.1.27]	0.97	0.92	1.03	4.0E-01	6.0E-01
K03274	ADP-L-glycero-D-manno-heptose 6-epimerase [EC:5.1.3.20]	1.03	0.97	1.09	4.0E-01	6.0E-01
K01846	methylaspartate mutase sigma subunit [EC:5.4.99.1]	1.03	0.97	1.09	4.0E-01	6.0E-01
K14368	3-alpha-mycarosylerythronolide B desosaminyl transferase [EC:2.4.1.278]	0.97	0.92	1.03	4.0E-01	6.0E-01
K14366	6-deoxyerythronolide B hydroxylase [EC:1.14.15.35]	0.97	0.92	1.03	4.0E-01	6.0E-01
K13311	dTDP-3-amino-3,4,6-trideoxy-alpha-D-glucopyranose N,N-dimethyltransferase [EC:2.1.1.234]	0.97	0.92	1.03	4.0E-01	6.0E-01
K14370	erythromycin 12 hydroxylase [EC:1.14.13.154]	0.97	0.92	1.03	4.0E-01	6.0E-01
K14369	erythromycin 3''-O-methyltransferase [EC:2.1.1.254]	0.97	0.92	1.03	4.0E-01	6.0E-01
K02193	heme exporter protein A [EC:7.6.2.5]	1.03	0.97	1.09	4.0E-01	6.0E-01
K08309	soluble lytic murein transglycosylase [EC:4.2.2.-]	1.03	0.97	1.09	4.0E-01	6.1E-01
K00068	sorbitol-6-phosphate 2-dehydrogenase [EC:1.1.1.140]	1.03	0.97	1.09	4.0E-01	6.1E-01
K00192	anaerobic carbon-monoxide dehydrogenase, CODH/ACS complex subunit alpha [EC:1.2.7.4]	0.97	0.92	1.03	4.0E-01	6.1E-01
K02080	putative deaminase/isomerase [EC:3.5.99.-]	1.03	0.97	1.09	4.0E-01	6.1E-01
K03790	[ribosomal protein S5]-alanine N-acetyltransferase [EC:2.3.1.267]	0.97	0.92	1.03	4.0E-01	6.1E-01
K07751	PepB aminopeptidase [EC:3.4.11.23]	1.03	0.97	1.09	4.0E-01	6.1E-01
K00099	1-deoxy-D-xylulose-5-phosphate reductoisomerase [EC:1.1.1.267]	0.98	0.92	1.03	4.0E-01	6.1E-01
K01916	NAD+ synthase [EC:6.3.1.5]	0.97	0.92	1.03	4.0E-01	6.1E-01
K12525	bifunctional aspartokinase / homoserine dehydrogenase 2 [EC:2.7.2.4 1.1.1.3]	1.03	0.97	1.09	4.0E-01	6.1E-01
K08299	crotonobetainyl-CoA hydratase [EC:4.2.1.149]	0.97	0.92	1.04	4.0E-01	6.1E-01
K03669	membrane glycosyltransferase [EC:2.4.1.-]	1.03	0.97	1.09	4.1E-01	6.1E-01
K10542	methyl-galactoside transport system ATP-binding protein [EC:7.5.2.11]	0.97	0.92	1.04	4.1E-01	6.1E-01
K12507	acyl-CoA synthetase [EC:6.2.1.-]	1.03	0.97	1.09	4.1E-01	6.1E-01
K00036	glucose-6-phosphate 1-dehydrogenase [EC:1.1.1.49 1.1.1.363]	0.98	0.92	1.03	4.1E-01	6.1E-01
K01581	ornithine decarboxylase [EC:4.1.1.17]	1.03	0.97	1.09	4.1E-01	6.1E-01
K13829	shikimate kinase / 3-dehydroquinate synthase [EC:2.7.1.71 4.2.3.4]	0.97	0.92	1.04	4.1E-01	6.1E-01
K00050	NA	1.03	0.97	1.09	4.1E-01	6.1E-01
K01609	indole-3-glycerol phosphate synthase [EC:4.1.1.48]	1.03	0.97	1.09	4.1E-01	6.1E-01
K00688	glycogen phosphorylase [EC:2.4.1.1]	0.98	0.92	1.03	4.1E-01	6.1E-01
K01923	phosphoribosylaminoimidazole-succinocarboxamide synthase [EC:6.3.2.6]	0.98	0.92	1.03	4.1E-01	6.1E-01
K10678	nitroreductase [EC:1.-.-.-]	1.03	0.97	1.09	4.1E-01	6.1E-01
K01465	dihydroorotase [EC:3.5.2.3]	0.98	0.92	1.03	4.1E-01	6.1E-01
K11386	arabinosyltransferase B [EC:2.4.2.-]	0.98	0.92	1.03	4.1E-01	6.1E-01
K00784	ribonuclease Z [EC:3.1.26.11]	0.98	0.92	1.03	4.1E-01	6.1E-01
K01156	type III restriction enzyme [EC:3.1.21.5]	1.03	0.97	1.09	4.1E-01	6.2E-01
K00598	trans-aconitate 2-methyltransferase [EC:2.1.1.144]	1.03	0.97	1.09	4.1E-01	6.2E-01

Predictor	Description	HR	2.5% CI	97.5% CI	P	FDR-adjusted P
K11751	5'-nucleotidase / UDP-sugar diphosphatase [EC:3.1.3.5 3.6.1.45]	0.98	0.92	1.03	4.1E-01	6.2E-01
K03207	colanic acid biosynthesis protein WcaH [EC:3.6.1.-]	1.03	0.97	1.09	4.1E-01	6.2E-01
K00355	NAD(P)H dehydrogenase (quinone) [EC:1.6.5.2]	0.98	0.92	1.03	4.1E-01	6.2E-01
K12152	phosphatase NudJ [EC:3.6.1.-]	1.03	0.97	1.09	4.2E-01	6.2E-01
K01141	exodeoxyribonuclease I [EC:3.1.11.1]	1.03	0.97	1.09	4.2E-01	6.2E-01
K01912	phenylacetate-CoA ligase [EC:6.2.1.30]	0.98	0.92	1.03	4.2E-01	6.2E-01
K00873	pyruvate kinase [EC:2.7.1.40]	0.97	0.92	1.04	4.2E-01	6.2E-01
K09018	pyrimidine oxygenase [EC:1.14.99.46]	1.03	0.97	1.09	4.2E-01	6.2E-01
K11933	NADH oxidoreductase Hcr [EC:1.-.-.-]	1.03	0.97	1.09	4.2E-01	6.2E-01
K02345	DNA polymerase III subunit theta [EC:2.7.7.7]	1.03	0.97	1.09	4.2E-01	6.2E-01
K11938	HMP-PP phosphatase [EC:3.6.1.-]	1.03	0.97	1.09	4.2E-01	6.2E-01
K01649	2-isopropylmalate synthase [EC:2.3.3.13]	1.02	0.97	1.09	4.2E-01	6.2E-01
K13532	two-component system, sporulation sensor kinase D [EC:2.7.13.3]	0.98	0.92	1.04	4.2E-01	6.2E-01
K08313	fructose-6-phosphate aldolase 1 [EC:4.1.2.-]	1.02	0.97	1.09	4.2E-01	6.2E-01
K01575	acetolactate decarboxylase [EC:4.1.1.5]	0.98	0.92	1.04	4.2E-01	6.2E-01
K03656	ATP-dependent DNA helicase Rep [EC:3.6.4.12]	1.02	0.97	1.09	4.2E-01	6.2E-01
K16148	alpha-maltose-1-phosphate synthase [EC:2.4.1.342]	0.98	0.92	1.04	4.2E-01	6.2E-01
K07700	two-component system, CitB family, cit operon sensor histidine kinase CitA [EC:2.7.13.3]	1.02	0.97	1.09	4.2E-01	6.2E-01
K01791	UDP-N-acetylglucosamine 2-epimerase (non-hydrolysing) [EC:5.1.3.14]	0.98	0.92	1.03	4.2E-01	6.2E-01
K00276	primary-amine oxidase [EC:1.4.3.21]	0.98	0.92	1.04	4.2E-01	6.2E-01
K12904	phosphonoacetaldehyde reductase [EC:1.1.1.309]	1.02	0.97	1.09	4.2E-01	6.3E-01
K08723	5'-nucleotidase [EC:3.1.3.5]	1.02	0.97	1.09	4.2E-01	6.3E-01
K01918	pantoate--beta-alanine ligase [EC:6.3.2.1]	1.03	0.96	1.09	4.2E-01	6.3E-01
K08351	biotin/methionine sulfoxide reductase [EC:1.-.-.-]	1.02	0.97	1.09	4.2E-01	6.3E-01
K01040	glutaconate CoA-transferase, subunit B [EC:2.8.3.12]	0.98	0.92	1.04	4.2E-01	6.3E-01
K13938	dihydromonapterin reductase / dihydrofolate reductase [EC:1.5.1.50 1.5.1.3]	1.02	0.97	1.09	4.3E-01	6.3E-01
K07711	two-component system, NtrC family, sensor histidine kinase GlrK [EC:2.7.13.3]	1.02	0.97	1.09	4.3E-01	6.3E-01
K00494	alkanal monooxygenase alpha chain [EC:1.14.14.3]	0.98	0.92	1.04	4.3E-01	6.3E-01
K15854	alkanal monooxygenase beta chain [EC:1.14.14.3]	0.98	0.92	1.04	4.3E-01	6.3E-01
K03400	long-chain-fatty-acyl-CoA reductase [EC:1.2.1.50]	0.98	0.92	1.04	4.3E-01	6.3E-01
K02812	sorbose PTS system EIIA component [EC:2.7.1.206]	1.02	0.96	1.09	4.3E-01	6.3E-01
K02813	sorbose PTS system EIIB component [EC:2.7.1.206]	1.02	0.96	1.09	4.3E-01	6.3E-01
K01143	exodeoxyribonuclease (lambda-induced) [EC:3.1.11.3]	0.97	0.91	1.04	4.3E-01	6.3E-01
K01719	uroporphyrinogen-III synthase [EC:4.2.1.75]	0.98	0.92	1.03	4.3E-01	6.3E-01
K02428	XTP/dITP diphosphohydrolase [EC:3.6.1.66]	1.03	0.96	1.09	4.3E-01	6.3E-01
K03828	putative acetyltransferase [EC:2.3.1.-]	1.02	0.97	1.09	4.3E-01	6.3E-01
K12957	alcohol/geraniol dehydrogenase (NADP+) [EC:1.1.1.2 1.1.1.183]	1.02	0.97	1.09	4.3E-01	6.3E-01
K00179	indolepyruvate ferredoxin oxidoreductase, alpha subunit [EC:1.2.7.8]	0.98	0.93	1.03	4.3E-01	6.3E-01
K10817	6-deoxyerythronolide-B synthase EryAI [EC:2.3.1.94]	0.98	0.92	1.04	4.3E-01	6.3E-01
K03391	pentachlorophenol monooxygenase [EC:1.14.13.50]	0.98	0.92	1.04	4.3E-01	6.3E-01
K01439	succinyl-diaminopimelate desuccinylase [EC:3.5.1.18]	0.98	0.92	1.04	4.3E-01	6.3E-01
K03430	2-aminoethylphosphonate-pyruvate transaminase [EC:2.6.1.37]	0.98	0.92	1.03	4.3E-01	6.3E-01
K08682	acyl carrier protein phosphodiesterase [EC:3.1.4.14]	1.02	0.96	1.09	4.3E-01	6.3E-01
K09024	flavin reductase [EC:1.5.1.-]	1.02	0.96	1.09	4.3E-01	6.3E-01

Predictor	Description	HR	2.5% CI	97.5% CI	P	FDR-adjusted P
K12974	KDO2-lipid IV(A) palmitoleoyltransferase [EC:2.3.1.242]	1.02	0.96	1.09	4.3E-01	6.3E-01
K01633	7,8-dihydroneopterin aldolase/epimerase/oxygenase [EC:4.1.2.25 5.1.99.8 1.13.11.81]	0.98	0.92	1.04	4.3E-01	6.3E-01
K01501	nitrilase [EC:3.5.5.1]	0.98	0.92	1.04	4.3E-01	6.3E-01
K03472	D-erythrose 4-phosphate dehydrogenase [EC:1.2.1.72]	1.02	0.96	1.09	4.3E-01	6.3E-01
K12145	hydrogenase-4 component J [EC:1.-.-.-]	1.02	0.96	1.09	4.3E-01	6.3E-01
K01424	L-asparaginase [EC:3.5.1.1]	0.98	0.92	1.04	4.3E-01	6.3E-01
K14170	chorismate mutase / prephenate dehydratase [EC:5.4.99.5 4.2.1.51]	0.98	0.92	1.04	4.3E-01	6.3E-01
K00931	glutamate 5-kinase [EC:2.7.2.11]	0.98	0.92	1.04	4.3E-01	6.3E-01
K00374	nitrate reductase gamma subunit [EC:1.7.5.1 1.7.99.-]	1.02	0.96	1.09	4.3E-01	6.3E-01
K08314	fructose-6-phosphate aldolase 2 [EC:4.1.2.-]	1.02	0.96	1.09	4.3E-01	6.3E-01
K01484	succinylarginine dihydrolase [EC:3.5.3.23]	1.02	0.96	1.09	4.3E-01	6.3E-01
K00370	nitrate reductase / nitrite oxidoreductase, alpha subunit [EC:1.7.5.1 1.7.99.-]	1.02	0.96	1.09	4.3E-01	6.3E-01
K02551	2-succinyl-5-enolpyruvyl-6-hydroxy-3-cyclohexene-1-carboxylate synthase [EC:2.2.1.9]	1.03	0.96	1.09	4.4E-01	6.3E-01
K00957	sulfate adenylyltransferase subunit 2 [EC:2.7.7.4]	1.03	0.96	1.09	4.4E-01	6.3E-01
K04041	fructose-1,6-bisphosphatase III [EC:3.1.3.11]	0.98	0.92	1.04	4.4E-01	6.3E-01
K12111	evolved beta-galactosidase subunit alpha [EC:3.2.1.23]	1.02	0.96	1.09	4.4E-01	6.3E-01
K00883	2-dehydro-3-deoxygalactonokinase [EC:2.7.1.58]	1.02	0.96	1.09	4.4E-01	6.3E-01
K08322	L-gulonate 5-dehydrogenase [EC:1.1.1.380]	1.02	0.96	1.09	4.4E-01	6.3E-01
K03829	putative acetyltransferase [EC:2.3.1.-]	1.02	0.96	1.09	4.4E-01	6.3E-01
K06447	succinylglutamic semialdehyde dehydrogenase [EC:1.2.1.71]	1.02	0.96	1.09	4.4E-01	6.3E-01
K01630	2-dehydro-3-deoxyglucarate aldolase [EC:4.1.2.20]	1.02	0.96	1.09	4.4E-01	6.3E-01
K01186	sialidase-1 [EC:3.2.1.18]	1.02	0.96	1.09	4.4E-01	6.3E-01
K01760	cysteine-S-conjugate beta-lyase [EC:4.4.1.13]	0.98	0.92	1.04	4.4E-01	6.3E-01
K02439	thiosulfate sulfurtransferase [EC:2.8.1.1]	1.02	0.96	1.09	4.4E-01	6.3E-01
K07675	two-component system, NarL family, sensor histidine kinase UhpB [EC:2.7.13.3]	1.02	0.96	1.09	4.4E-01	6.3E-01
K03654	ATP-dependent DNA helicase RecQ [EC:3.6.4.12]	1.02	0.96	1.09	4.4E-01	6.3E-01
K15396	tRNA (cytidine32/uridine32-2'-O)-methyltransferase [EC:2.1.1.200]	1.02	0.96	1.09	4.4E-01	6.3E-01
K01627	2-dehydro-3-deoxyphosphooctonate aldolase (KDO 8-P synthase) [EC:2.5.1.55]	1.03	0.96	1.09	4.4E-01	6.3E-01
K01252	bifunctional isochorismate lyase / aryl carrier protein [EC:3.3.2.1 6.3.2.14]	1.02	0.96	1.09	4.4E-01	6.3E-01
K06957	tRNA(Met) cytidine acetyltransferase [EC:2.3.1.193]	1.02	0.96	1.09	4.4E-01	6.4E-01
K00840	succinylornithine aminotransferase [EC:2.6.1.81]	1.02	0.96	1.09	4.4E-01	6.4E-01
K01104	protein-tyrosine phosphatase [EC:3.1.3.48]	1.02	0.97	1.08	4.4E-01	6.4E-01
K11264	methylmalonyl-CoA decarboxylase [EC:4.1.1.-]	1.02	0.96	1.09	4.4E-01	6.4E-01
K00794	6,7-dimethyl-8-ribityllumazine synthase [EC:2.5.1.78]	1.02	0.96	1.09	4.4E-01	6.4E-01
K03214	RNA methyltransferase, TrmH family [EC:2.1.1.-]	1.02	0.96	1.09	4.4E-01	6.4E-01
K06222	2,5-diketo-D-gluconate reductase B [EC:1.1.1.346]	1.02	0.96	1.09	4.4E-01	6.4E-01
K07678	two-component system, NarL family, sensor histidine kinase BarA [EC:2.7.13.3]	1.02	0.96	1.09	4.4E-01	6.4E-01
K03551	holliday junction DNA helicase RuvB [EC:3.6.4.12]	1.02	0.96	1.09	4.4E-01	6.4E-01
K12136	hydrogenase-4 component A [EC:1.-.-.-]	1.02	0.96	1.09	4.4E-01	6.4E-01
K02564	glucosamine-6-phosphate deaminase [EC:3.5.99.6]	0.98	0.92	1.04	4.4E-01	6.4E-01
K00942	guanylate kinase [EC:2.7.4.8]	0.98	0.92	1.04	4.4E-01	6.4E-01
K05590	ATP-dependent RNA helicase SrmB [EC:3.6.4.13]	1.02	0.96	1.09	4.5E-01	6.4E-01
K01962	acetyl-CoA carboxylase carboxyl transferase subunit alpha [EC:6.4.1.2 2.1.3.15]	0.98	0.92	1.04	4.5E-01	6.4E-01
K07648	two-component system, OmpR family, aerobic respiration control sensor histidine kinase ArcB [EC:2.7.13.3]	1.02	0.96	1.09	4.5E-01	6.4E-01

Predictor	Description	HR	2.5% CI	97.5% CI	P	FDR-adjusted P
K03224	ATP synthase in type III secretion protein N [EC:7.4.2.8]	1.02	0.96	1.09	4.5E-01	6.4E-01
K13935	malonate decarboxylase epsilon subunit [EC:2.3.1.39]	0.98	0.92	1.04	4.5E-01	6.4E-01
K06021	NA	0.98	0.92	1.04	4.5E-01	6.4E-01
K00093	methanol dehydrogenase [EC:1.1.1.244]	0.98	0.92	1.04	4.5E-01	6.4E-01
K12142	hydrogenase-4 component G [EC:1.-.-.-]	1.02	0.96	1.09	4.5E-01	6.4E-01
K13574	hydroxycarboxylate dehydrogenase B [EC:1.1.1.237 1.1.1.-]	1.02	0.96	1.09	4.5E-01	6.4E-01
K12582	dTDP-N-acetylfucosamine:lipid II N-acetylfucosaminyltransferase [EC:2.4.1.325]	1.02	0.96	1.09	4.5E-01	6.4E-01
K00813	aspartate aminotransferase [EC:2.6.1.1]	1.02	0.96	1.09	4.5E-01	6.4E-01
K01480	agmatinase [EC:3.5.3.11]	0.98	0.92	1.04	4.5E-01	6.4E-01
K00146	phenylacetaldehyde dehydrogenase [EC:1.2.1.39]	0.98	0.92	1.04	4.5E-01	6.4E-01
K00880	L-xylulokinase [EC:2.7.1.53]	1.02	0.96	1.09	4.5E-01	6.4E-01
K08306	membrane-bound lytic murein transglycosylase C [EC:4.2.2.-]	1.02	0.96	1.09	4.5E-01	6.4E-01
K11633	two-component system, OmpR family, sensor histidine kinase YxdK [EC:2.7.13.3]	0.98	0.92	1.04	4.5E-01	6.4E-01
K01661	naphthoate synthase [EC:4.1.3.36]	1.02	0.96	1.09	4.5E-01	6.4E-01
K03772	FKBP-type peptidyl-prolyl cis-trans isomerase FkpA [EC:5.2.1.8]	0.98	0.92	1.04	4.5E-01	6.4E-01
K03078	3-dehydro-L-gulonate-6-phosphate decarboxylase [EC:4.1.1.85]	1.02	0.96	1.09	4.5E-01	6.4E-01
K01599	uroporphyrinogen decarboxylase [EC:4.1.1.37]	0.98	0.92	1.04	4.5E-01	6.4E-01
K05882	aryl-alcohol dehydrogenase (NADP+) [EC:1.1.1.91]	0.98	0.92	1.04	4.5E-01	6.4E-01
K10441	ribose transport system ATP-binding protein [EC:7.5.2.7]	0.98	0.92	1.04	4.5E-01	6.4E-01
K01802	peptidylprolyl isomerase [EC:5.2.1.8]	0.98	0.93	1.04	4.5E-01	6.4E-01
K02362	enterobactin synthetase component D [EC:6.3.2.14 2.7.8.-]	1.02	0.96	1.09	4.5E-01	6.4E-01
K00575	chemotaxis protein methyltransferase CheR [EC:2.1.1.80]	0.98	0.92	1.04	4.5E-01	6.4E-01
K07317	adenine-specific DNA-methyltransferase [EC:2.1.1.72]	0.98	0.92	1.04	4.6E-01	6.5E-01
K07640	two-component system, OmpR family, sensor histidine kinase CpxA [EC:2.7.13.3]	1.02	0.96	1.09	4.6E-01	6.5E-01
K01460	glutathionylspermidine amidase/synthetase [EC:3.5.1.78 6.3.1.8]	1.02	0.96	1.09	4.6E-01	6.5E-01
K01739	cystathionine gamma-synthase [EC:2.5.1.48]	1.02	0.96	1.09	4.6E-01	6.5E-01
K00812	aspartate aminotransferase [EC:2.6.1.1]	0.98	0.92	1.04	4.6E-01	6.5E-01
K08092	3-dehydro-L-gulonate 2-dehydrogenase [EC:1.1.1.130]	1.02	0.96	1.09	4.6E-01	6.5E-01
K02846	N-methyl-L-tryptophan oxidase [EC:1.5.3.-]	1.02	0.96	1.09	4.6E-01	6.5E-01
K00255	long-chain-acyl-CoA dehydrogenase [EC:1.3.8.8]	0.98	0.92	1.04	4.6E-01	6.5E-01
K02510	4-hydroxy-2-oxoheptanedioate aldolase [EC:4.1.2.52]	0.98	0.92	1.04	4.6E-01	6.5E-01
K10837	O-phosphoseryl-tRNA(Sec) kinase [EC:2.7.1.164]	1.02	0.96	1.08	4.6E-01	6.5E-01
K02291	15-cis-phytoene synthase [EC:2.5.1.32]	0.98	0.92	1.04	4.6E-01	6.5E-01
K00557	tRNA (uracil-5-)-methyltransferase [EC:2.1.1.35]	1.02	0.96	1.09	4.6E-01	6.5E-01
K00884	N-acetylglucosamine kinase [EC:2.7.1.59]	1.02	0.96	1.09	4.6E-01	6.5E-01
K08302	tagatose 1,6-diphosphate aldolase GatY/KbaY [EC:4.1.2.40]	1.02	0.96	1.09	4.6E-01	6.5E-01
K01494	dCTP deaminase [EC:3.5.4.13]	0.98	0.92	1.04	4.6E-01	6.5E-01
K04781	salicylate synthetase [EC:5.4.4.2 4.2.99.21]	1.02	0.96	1.09	4.6E-01	6.5E-01
K05304	sialic acid synthase [EC:2.5.1.56 2.5.1.57 2.5.1.132]	0.98	0.92	1.04	4.6E-01	6.5E-01
K12137	hydrogenase-4 component B [EC:1.-.-.-]	1.02	0.96	1.09	4.6E-01	6.5E-01
K03407	two-component system, chemotaxis family, sensor kinase CheA [EC:2.7.13.3]	0.98	0.92	1.04	4.6E-01	6.5E-01
K03412	two-component system, chemotaxis family, protein-glutamate methylesterase/glutaminase [EC:3.1.1.61 3.5.1.44]	0.98	0.92	1.04	4.6E-01	6.5E-01
K09473	gamma-glutamyl-gamma-aminobutyrate hydrolase [EC:3.5.1.94]	1.02	0.96	1.09	4.6E-01	6.5E-01
K02618	oxepin-CoA hydrolase / 3-oxo-5,6-dehydrosuberyl-CoA semialdehyde dehydrogenase [EC:3.3.2.12 1.2.1.91]	0.98	0.92	1.04	4.6E-01	6.5E-01

Predictor	Description	HR	2.5% CI	97.5% CI	P	FDR-adjusted P
K02472	UDP-N-acetyl-D-mannosaminuronic acid dehydrogenase [EC:1.1.1.336]	0.98	0.92	1.04	4.6E-01	6.5E-01
K09470	gamma-glutamylputrescine synthase [EC:6.3.1.11]	1.02	0.96	1.09	4.6E-01	6.5E-01
K11258	acetolactate synthase II small subunit [EC:2.2.1.6]	1.02	0.96	1.09	4.6E-01	6.5E-01
K02850	heptose II phosphotransferase [EC:2.7.1.-]	1.02	0.96	1.09	4.6E-01	6.5E-01
K11609	beta-ketoacyl ACP synthase [EC:2.3.1.293 2.3.1.294]	0.98	0.92	1.04	4.6E-01	6.5E-01
K00135	succinate-semialdehyde dehydrogenase / glutarate-semialdehyde dehydrogenase [EC:1.2.1.16 1.2.1.79 1.2.1.20]	0.98	0.92	1.04	4.6E-01	6.5E-01
K00211	prephenate dehydrogenase (NADP+) [EC:1.3.1.13]	0.98	0.92	1.04	4.6E-01	6.5E-01
K05708	3-phenylpropionate/trans-cinnamate dioxygenase subunit alpha [EC:1.14.12.19]	1.02	0.96	1.09	4.6E-01	6.5E-01
K05774	ribose 1,5-bisphosphokinase [EC:2.7.4.23]	1.02	0.96	1.09	4.6E-01	6.5E-01
K02794	mannose PTS system EIIAB component [EC:2.7.1.191]	0.98	0.92	1.04	4.6E-01	6.5E-01
K01698	porphobilinogen synthase [EC:4.2.1.24]	1.02	0.96	1.09	4.7E-01	6.5E-01
K13873	L-arabinose 1-dehydrogenase [EC:1.1.1.376]	0.98	0.92	1.04	4.7E-01	6.5E-01
K13421	uridine monophosphate synthetase [EC:2.4.2.10 4.1.1.23]	0.98	0.92	1.04	4.7E-01	6.5E-01
K07647	two-component system, OmpR family, sensor histidine kinase TorS [EC:2.7.13.3]	1.02	0.96	1.09	4.7E-01	6.5E-01
K04783	yersiniabactin salicyl-AMP ligase [EC:6.3.2.-]	1.02	0.96	1.09	4.7E-01	6.5E-01
K02560	lauroyl-Kdo2-lipid IVA myristoyltransferase [EC:2.3.1.243]	1.02	0.96	1.09	4.7E-01	6.5E-01
K06033	arylmalonate decarboxylase [EC:4.1.1.76]	0.98	0.92	1.04	4.7E-01	6.5E-01
K02231	adenosylcobinamide kinase / adenosylcobinamide-phosphate guanylyltransferase [EC:2.7.1.156 2.7.7.62]	1.02	0.96	1.09	4.7E-01	6.5E-01
K13014	undecaprenyl phosphate-alpha-L-ara4FN deformylase [EC:3.5.1.-]	1.02	0.96	1.09	4.7E-01	6.5E-01
K00058	D-3-phosphoglycerate dehydrogenase / 2-oxoglutarate reductase [EC:1.1.1.95 1.1.1.399]	1.02	0.96	1.08	4.7E-01	6.5E-01
K07229	ferric-chelate reductase (NADPH) [EC:1.16.1.9]	1.02	0.96	1.09	4.7E-01	6.5E-01
K16159	methane monooxygenase component A gamma chain [EC:1.14.13.25]	0.98	0.92	1.04	4.7E-01	6.5E-01
K16161	methane monooxygenase component C [EC:1.14.13.25]	0.98	0.92	1.04	4.7E-01	6.5E-01
K00860	adenylylsulfate kinase [EC:2.7.1.25]	0.98	0.93	1.04	4.7E-01	6.5E-01
K06445	acyl-CoA dehydrogenase [EC:1.3.99.-]	1.02	0.96	1.08	4.7E-01	6.5E-01
K08963	methylthioribose-1-phosphate isomerase [EC:5.3.1.23]	0.98	0.92	1.04	4.7E-01	6.5E-01
K00701	cyclomaltodextrin glucanotransferase [EC:2.4.1.19]	1.02	0.96	1.08	4.7E-01	6.5E-01
K03181	chorismate lyase [EC:4.1.3.40]	1.02	0.96	1.08	4.7E-01	6.5E-01
K01696	tryptophan synthase beta chain [EC:4.2.1.20]	0.98	0.93	1.04	4.7E-01	6.5E-01
K02509	2-oxo-hept-3-ene-1,7-dioate hydratase [EC:4.2.1.-]	1.02	0.96	1.09	4.7E-01	6.5E-01
K02844	UDP-glucose:(heptosyl)LPS alpha-1,3-glucosyltransferase [EC:2.4.1.-]	1.02	0.96	1.08	4.7E-01	6.5E-01
K00956	sulfate adenylyltransferase subunit 1 [EC:2.7.7.4]	0.98	0.93	1.04	4.7E-01	6.5E-01
K02799	NA	0.98	0.92	1.04	4.7E-01	6.5E-01
K00029	malate dehydrogenase (oxaloacetate-decarboxylating)(NADP+) [EC:1.1.1.40]	1.02	0.96	1.09	4.7E-01	6.5E-01
K10831	taurine transport system ATP-binding protein [EC:7.6.2.7]	1.02	0.96	1.09	4.7E-01	6.5E-01
K07645	two-component system, OmpR family, sensor histidine kinase QseC [EC:2.7.13.3]	1.02	0.96	1.08	4.8E-01	6.6E-01
K02745	N-acetylgalactosamine PTS system EIIB component [EC:2.7.1.-]	1.02	0.96	1.09	4.8E-01	6.6E-01
K12139	hydrogenase-4 component D [EC:1.-.-.-]	1.02	0.96	1.08	4.8E-01	6.6E-01
K01961	acetyl-CoA carboxylase, biotin carboxylase subunit [EC:6.4.1.2 6.3.4.14]	0.98	0.92	1.04	4.8E-01	6.6E-01
K12144	hydrogenase-4 component I [EC:1.-.-.-]	1.02	0.96	1.09	4.8E-01	6.6E-01
K03658	DNA helicase IV [EC:3.6.4.12]	0.98	0.92	1.04	4.8E-01	6.6E-01
K00076	7-alpha-hydroxysteroid dehydrogenase [EC:1.1.1.159]	1.02	0.96	1.09	4.8E-01	6.6E-01
K00260	glutamate dehydrogenase [EC:1.4.1.2]	0.98	0.92	1.04	4.8E-01	6.6E-01
K15922	sulfoquinovosidase [EC:3.2.1.199]	1.02	0.96	1.08	4.8E-01	6.6E-01

Predictor	Description	HR	2.5% CI	97.5% CI	P	FDR-adjusted P
K04042	bifunctional UDP-N-acetylglucosamine pyrophosphorylase / glucosamine-1-phosphate N-acetyltransferase [EC:2.7.7.23 2.3.1.157]	0.98	0.92	1.04	4.8E-01	6.6E-01
K03797	carboxyl-terminal processing protease [EC:3.4.21.102]	0.98	0.92	1.04	4.8E-01	6.6E-01
K05591	ATP-dependent RNA helicase DbpA [EC:3.6.4.13]	1.02	0.96	1.08	4.8E-01	6.6E-01
K11987	prostaglandin-endoperoxide synthase 2 [EC:1.14.99.1]	0.98	0.92	1.04	4.8E-01	6.6E-01
K00371	nitrate reductase / nitrite oxidoreductase, beta subunit [EC:1.7.5.1 1.7.99.-]	1.02	0.96	1.08	4.8E-01	6.6E-01
K02344	DNA polymerase III subunit psi [EC:2.7.7.7]	1.02	0.96	1.08	4.8E-01	6.6E-01
K04708	3-dehydrosphinganine reductase [EC:1.1.1.102]	0.98	0.92	1.04	4.8E-01	6.6E-01
K07264	4-amino-4-deoxy-L-arabinose transferase [EC:2.4.2.43]	1.02	0.96	1.08	4.8E-01	6.6E-01
K00673	arginine N-succinyltransferase [EC:2.3.1.109]	1.02	0.96	1.08	4.8E-01	6.6E-01
K11065	thioredoxin-dependent peroxiredoxin [EC:1.11.1.24]	0.98	0.92	1.04	4.8E-01	6.6E-01
K00162	pyruvate dehydrogenase E1 component beta subunit [EC:1.2.4.1]	0.98	0.92	1.04	4.8E-01	6.6E-01
K11202	fructose-like PTS system EIIB component [EC:2.7.1.-]	1.02	0.96	1.08	4.8E-01	6.6E-01
K13069	diguanylate cyclase [EC:2.7.7.65]	1.02	0.96	1.08	4.8E-01	6.6E-01
K02433	aspartyl-tRNA(Asn)/glutamyl-tRNA(Gln) amidotransferase subunit A [EC:6.3.5.6 6.3.5.7]	0.98	0.92	1.04	4.8E-01	6.6E-01
K03826	putative acetyltransferase [EC:2.3.1.-]	1.02	0.96	1.08	4.8E-01	6.6E-01
K03831	molybdopterin adenylyltransferase [EC:2.7.7.75]	1.02	0.96	1.08	4.8E-01	6.6E-01
K00998	CDP-diacylglycerol---serine O-phosphatidyltransferase [EC:2.7.8.8]	1.02	0.96	1.08	4.8E-01	6.6E-01
K08312	ADP-ribose diphosphatase [EC:3.6.1.-]	1.02	0.96	1.08	4.8E-01	6.6E-01
K00463	indoleamine 2,3-dioxygenase [EC:1.13.11.52]	1.02	0.96	1.08	4.8E-01	6.6E-01
K01478	arginine deiminase [EC:3.5.3.6]	0.98	0.92	1.04	4.8E-01	6.6E-01
K01681	aconitate hydratase [EC:4.2.1.3]	1.02	0.96	1.08	4.8E-01	6.6E-01
K07643	two-component system, OmpR family, sensor histidine kinase BasS [EC:2.7.13.3]	1.02	0.96	1.08	4.9E-01	6.6E-01
K01631	2-dehydro-3-deoxyphosphogalactonate aldolase [EC:4.1.2.21]	1.02	0.96	1.08	4.9E-01	6.6E-01
K01546	potassium-transporting ATPase potassium-binding subunit	1.02	0.96	1.09	4.9E-01	6.6E-01
K01447	N-acetylmuramoyl-L-alanine amidase [EC:3.5.1.28]	1.02	0.96	1.09	4.9E-01	6.6E-01
K03474	pyridoxine 5-phosphate synthase [EC:2.6.99.2]	1.02	0.96	1.09	4.9E-01	6.6E-01
K03082	NA	1.02	0.96	1.08	4.9E-01	6.6E-01
K01160	crossover junction endodeoxyribonuclease RusA [EC:3.1.21.10]	1.02	0.96	1.08	4.9E-01	6.7E-01
K12527	putative selenate reductase [EC:1.97.1.9]	1.02	0.96	1.08	4.9E-01	6.7E-01
K07676	two-component system, NarL family, sensor histidine kinase RcsD [EC:2.7.13.3]	1.02	0.96	1.08	4.9E-01	6.7E-01
K01708	galactarate dehydratase [EC:4.2.1.42]	1.02	0.96	1.08	4.9E-01	6.7E-01
K02853	enterobacterial common antigen polymerase [EC:2.4.1.-]	1.02	0.96	1.08	4.9E-01	6.7E-01
K05541	tRNA-dihydrouridine synthase C [EC:1.-.-.-]	1.02	0.96	1.08	4.9E-01	6.7E-01
K08599	YopT peptidase [EC:3.4.22.-]	0.98	0.92	1.04	4.9E-01	6.7E-01
K08591	acyl phosphate:glycerol-3-phosphate acyltransferase [EC:2.3.1.275]	0.98	0.92	1.04	4.9E-01	6.7E-01
K10798	poly [ADP-ribose] polymerase 2/3/4 [EC:2.4.2.30]	0.98	0.92	1.04	4.9E-01	6.7E-01
K02361	isochorismate synthase [EC:5.4.4.2]	0.98	0.93	1.04	4.9E-01	6.7E-01
K01521	CDP-diacylglycerol pyrophosphatase [EC:3.6.1.26]	1.02	0.96	1.08	4.9E-01	6.7E-01
K13501	anthranilate synthase / indole-3-glycerol phosphate synthase / phosphoribosylanthranilate isomerase [EC:4.1.3.27 4.1.1.48 5.3.1.24]	0.98	0.92	1.04	5.0E-01	6.7E-01
K00455	3,4-dihydroxyphenylacetate 2,3-dioxygenase [EC:1.13.11.15]	1.02	0.96	1.08	5.0E-01	6.7E-01
K10011	UDP-4-amino-4-deoxy-L-arabinose formyltransferase / UDP-glucuronic acid dehydrogenase (UDP-4-keto-hexauronic acid decarboxylating) [EC:2.1.2.13 1.1.1.305]	1.02	0.96	1.08	5.0E-01	6.7E-01
K00094	galactitol-1-phosphate 5-dehydrogenase [EC:1.1.1.251]	1.02	0.96	1.08	5.0E-01	6.7E-01
K03779	L(+)-tartrate dehydratase alpha subunit [EC:4.2.1.32]	1.02	0.96	1.08	5.0E-01	6.7E-01
K01407	protease III [EC:3.4.24.55]	1.02	0.96	1.08	5.0E-01	6.7E-01

Predictor	Description	HR	2.5% CI	97.5% CI	P	FDR-adjusted P
K00769	xanthine phosphoribosyltransferase [EC:2.4.2.22]	1.02	0.96	1.08	5.0E-01	6.7E-01
K05879	phosphoenolpyruvate---glycerone phosphotransferase subunit DhaL [EC:2.7.1.121]	1.02	0.96	1.08	5.0E-01	6.7E-01
K07673	two-component system, NarL family, nitrate/nitrite sensor histidine kinase NarX [EC:2.7.13.3]	1.02	0.96	1.08	5.0E-01	6.7E-01
K03341	O-phospho-L-seryl-tRNASec:L-selenocysteinyl-tRNA synthase [EC:2.9.1.2]	1.02	0.96	1.08	5.0E-01	6.7E-01
K08679	UDP-glucuronate 4-epimerase [EC:5.1.3.6]	1.02	0.96	1.08	5.0E-01	6.7E-01
K00073	ureidoglycolate dehydrogenase (NAD+) [EC:1.1.1.350]	1.02	0.96	1.08	5.0E-01	6.7E-01
K01607	4-carboxymuconolactone decarboxylase [EC:4.1.1.44]	1.02	0.96	1.08	5.0E-01	6.7E-01
K10536	agmatine deiminase [EC:3.5.3.12]	0.98	0.92	1.04	5.0E-01	6.7E-01
K02438	glycogen debranching enzyme [EC:3.2.1.196]	1.02	0.96	1.08	5.0E-01	6.7E-01
K03820	apolipoprotein N-acyltransferase [EC:2.3.1.269]	1.02	0.96	1.08	5.0E-01	6.7E-01
K02851	UDP-GlcNAc:undecaprenyl-phosphate/decaprenyl-phosphate GlcNAc-1-phosphate transferase [EC:2.7.8.33 2.7.8.35]	0.98	0.92	1.04	5.0E-01	6.7E-01
K07259	serine-type D-Ala-D-Ala carboxypeptidase/endopeptidase (penicillin-binding protein 4) [EC:3.4.16.4 3.4.21.-]	0.98	0.93	1.04	5.0E-01	6.7E-01
K15736	(S)-2-hydroxyglutarate dehydrogenase [EC:1.1.5.13]	1.02	0.96	1.08	5.0E-01	6.7E-01
K10111	multiple sugar transport system ATP-binding protein [EC:7.5.2.-]	1.02	0.96	1.08	5.0E-01	6.7E-01
K10909	two-component system, autoinducer 2 sensor kinase/phosphatase LuxQ [EC:2.7.13.3 3.1.3.-]	0.98	0.92	1.04	5.0E-01	6.7E-01
K13307	dTDP-3-amino-3,6-dideoxy-alpha-D-glucopyranose N,N-dimethyltransferase [EC:2.1.1.235]	0.98	0.92	1.04	5.0E-01	6.7E-01
K15944	nogalaviketone/aklaviketone reductase [EC:1.1.1.- 1.1.1.362]	0.98	0.92	1.04	5.0E-01	6.7E-01
K00851	gluconokinase [EC:2.7.1.12]	0.98	0.92	1.04	5.1E-01	6.8E-01
K10793	D-proline reductase (dithiol) PrdA [EC:1.21.4.1]	0.98	0.92	1.04	5.1E-01	6.8E-01
K00926	carbamate kinase [EC:2.7.2.2]	1.02	0.96	1.08	5.1E-01	6.8E-01
K15986	manganese-dependent inorganic pyrophosphatase [EC:3.6.1.1]	0.98	0.92	1.04	5.1E-01	6.8E-01
K08484	phosphotransferase system, enzyme I, PtsP [EC:2.7.3.9]	1.02	0.96	1.08	5.1E-01	6.8E-01
K13062	acyl homoserine lactone synthase [EC:2.3.1.184]	0.98	0.92	1.04	5.1E-01	6.8E-01
K01241	AMP nucleosidase [EC:3.2.2.4]	0.98	0.93	1.04	5.1E-01	6.8E-01
K09011	(R)-citramalate synthase [EC:2.3.1.182]	0.98	0.93	1.04	5.1E-01	6.8E-01
K14155	cysteine-S-conjugate beta-lyase [EC:4.4.1.13]	0.98	0.92	1.04	5.1E-01	6.8E-01
K03774	FKBP-type peptidyl-prolyl cis-trans isomerase SlpA [EC:5.2.1.8]	1.02	0.96	1.08	5.1E-01	6.8E-01
K00031	isocitrate dehydrogenase [EC:1.1.1.42]	0.98	0.93	1.04	5.1E-01	6.8E-01
K12972	glyoxylate/hydroxypyruvate reductase [EC:1.1.1.79 1.1.1.81]	1.02	0.96	1.08	5.1E-01	6.8E-01
K07251	thiamine kinase [EC:2.7.1.89]	1.02	0.96	1.08	5.1E-01	6.8E-01
K03777	D-lactate dehydrogenase (quinone) [EC:1.1.5.12]	1.02	0.96	1.08	5.1E-01	6.8E-01
K07710	two-component system, NtrC family, sensor histidine kinase AtoS [EC:2.7.13.3]	1.02	0.96	1.08	5.1E-01	6.8E-01
K01966	propionyl-CoA carboxylase beta chain [EC:6.4.1.3 2.1.3.15]	1.02	0.96	1.08	5.1E-01	6.8E-01
K08678	UDP-glucuronate decarboxylase [EC:4.1.1.35]	1.02	0.96	1.08	5.1E-01	6.8E-01
K02614	acyl-CoA thioesterase [EC:3.1.2.-]	0.98	0.93	1.04	5.1E-01	6.8E-01
K01653	acetolactate synthase I/III small subunit [EC:2.2.1.6]	0.98	0.92	1.04	5.1E-01	6.8E-01
K15461	tRNA 5-methylaminomethyl-2-thiouridine biosynthesis bifunctional protein [EC:2.1.1.61 1.5.-.-]	1.02	0.96	1.08	5.1E-01	6.8E-01
K06132	cardiolipin synthase C [EC:2.7.8.-]	1.02	0.96	1.08	5.2E-01	6.8E-01
K01354	oligopeptidase B [EC:3.4.21.83]	1.02	0.96	1.08	5.2E-01	6.8E-01
K14581	naphthalene 1,2-dioxygenase ferredoxin reductase component [EC:1.18.1.7]	1.02	0.96	1.08	5.2E-01	6.8E-01
K03182	4-hydroxy-3-polyprenylbenzoate decarboxylase [EC:4.1.1.98]	1.02	0.96	1.08	5.2E-01	6.8E-01
K06282	hydrogenase small subunit [EC:1.12.99.6]	1.02	0.96	1.08	5.2E-01	6.8E-01
K01714	4-hydroxy-tetrahydrodipicolinate synthase [EC:4.3.3.7]	0.98	0.92	1.04	5.2E-01	6.8E-01
K01070	S-formylglutathione hydrolase [EC:3.1.2.12]	1.02	0.96	1.08	5.2E-01	6.9E-01

Predictor	Description	HR	2.5% CI	97.5% CI	P	FDR-adjusted P
K01194	alpha,alpha-trehalase [EC:3.2.1.28]	1.02	0.96	1.08	5.2E-01	6.9E-01
K10027	phytoene desaturase [EC:1.3.99.26 1.3.99.28 1.3.99.29 1.3.99.31]	0.98	0.92	1.04	5.2E-01	6.9E-01
K02337	DNA polymerase III subunit alpha [EC:2.7.7.7]	0.98	0.92	1.04	5.2E-01	6.9E-01
K12945	GDP-mannose pyrophosphatase NudK [EC:3.6.1.-]	1.02	0.96	1.08	5.2E-01	6.9E-01
K01911	o-succinylbenzoate---CoA ligase [EC:6.2.1.26]	1.02	0.96	1.09	5.2E-01	6.9E-01
K07107	acyl-CoA thioester hydrolase [EC:3.1.2.-]	1.02	0.96	1.09	5.2E-01	6.9E-01
K03080	NA	1.02	0.96	1.08	5.2E-01	6.9E-01
K03471	ribonuclease HIII [EC:3.1.26.4]	0.98	0.92	1.04	5.3E-01	6.9E-01
K02773	galactitol PTS system EIIA component [EC:2.7.1.200]	1.02	0.96	1.08	5.3E-01	6.9E-01
K00484	flavin reductase (NADH) [EC:1.5.1.36]	1.02	0.96	1.08	5.3E-01	6.9E-01
K03856	3-deoxy-7-phosphoheptulonate synthase [EC:2.5.1.54]	0.98	0.92	1.04	5.3E-01	6.9E-01
K06193	protein PhnA	1.02	0.96	1.08	5.3E-01	6.9E-01
K06041	arabinose-5-phosphate isomerase [EC:5.3.1.13]	1.02	0.96	1.09	5.3E-01	6.9E-01
K01608	tartronate-semialdehyde synthase [EC:4.1.1.47]	1.02	0.96	1.08	5.3E-01	6.9E-01
K16066	3-hydroxy acid dehydrogenase / malonic semialdehyde reductase [EC:1.1.1.381 1.1.1.-]	1.02	0.96	1.08	5.3E-01	6.9E-01
K12248	beta-galactoside alpha-2,6-sialyltransferase (sialyltransferase 0160) [EC:2.4.99.1]	1.02	0.96	1.08	5.3E-01	6.9E-01
K01467	beta-lactamase class C [EC:3.5.2.6]	1.02	0.96	1.08	5.3E-01	6.9E-01
K11391	23S rRNA (guanine1835-N2)-methyltransferase [EC:2.1.1.174]	1.02	0.96	1.08	5.3E-01	6.9E-01
K01711	GDPmannose 4,6-dehydratase [EC:4.2.1.47]	1.02	0.96	1.09	5.3E-01	6.9E-01
K00053	ketol-acid reductoisomerase [EC:1.1.1.86]	0.98	0.92	1.04	5.3E-01	6.9E-01
K01486	adenine deaminase [EC:3.5.4.2]	0.98	0.92	1.04	5.3E-01	6.9E-01
K00526	ribonucleoside-diphosphate reductase beta chain [EC:1.17.4.1]	1.02	0.96	1.08	5.3E-01	6.9E-01
K00785	N-acetyllactosaminide alpha-2,3-sialyltransferase [EC:2.4.99.6]	0.98	0.92	1.04	5.3E-01	6.9E-01
K01341	kexin [EC:3.4.21.61]	0.98	0.93	1.04	5.3E-01	6.9E-01
K00090	glyoxylate/hydroxypyruvate/2-ketogluconate reductase [EC:1.1.1.79 1.1.1.81 1.1.1.215]	1.02	0.96	1.08	5.3E-01	6.9E-01
K02170	pimeloyl-[acyl-carrier protein] methyl ester esterase [EC:3.1.1.85]	1.02	0.96	1.08	5.3E-01	6.9E-01
K01684	galactonate dehydratase [EC:4.2.1.6]	1.02	0.96	1.08	5.3E-01	6.9E-01
K05939	acyl-[acyl-carrier-protein]-phospholipid O-acyltransferase / long-chain-fatty-acid--[acyl-carrier-protein] ligase [EC:2.3.1.40 6.2.1.20]	1.02	0.96	1.08	5.3E-01	6.9E-01
K07641	two-component system, OmpR family, sensor histidine kinase CreC [EC:2.7.13.3]	1.02	0.96	1.08	5.3E-01	6.9E-01
K14287	methionine transaminase [EC:2.6.1.88]	1.02	0.96	1.08	5.3E-01	6.9E-01
K14467	4-hydroxybutyrate---CoA ligase (AMP-forming) [EC:6.2.1.40]	1.02	0.96	1.08	5.3E-01	6.9E-01
K02056	simple sugar transport system ATP-binding protein [EC:7.5.2.-]	0.98	0.92	1.04	5.3E-01	6.9E-01
K01520	dUTP pyrophosphatase [EC:3.6.1.23]	0.98	0.93	1.04	5.3E-01	6.9E-01
K06968	23S rRNA (cytidine2498-2'-O)-methyltransferase [EC:2.1.1.186]	1.02	0.96	1.08	5.3E-01	7.0E-01
K01537	P-type Ca2+ transporter type 2C [EC:7.2.2.10]	0.98	0.93	1.04	5.4E-01	7.0E-01
K00906	isocitrate dehydrogenase kinase/phosphatase [EC:2.7.11.5 3.1.3.-]	1.02	0.96	1.08	5.4E-01	7.0E-01
K01754	threonine dehydratase [EC:4.3.1.19]	0.98	0.93	1.04	5.4E-01	7.0E-01
K08253	non-specific protein-tyrosine kinase [EC:2.7.10.2]	0.98	0.93	1.04	5.4E-01	7.0E-01
K13450	phosphothreonine lyase [EC:4.2.3.-]	1.02	0.96	1.08	5.4E-01	7.0E-01
K01766	cysteine sulfinate desulfinase [EC:4.4.1.-]	1.02	0.96	1.08	5.4E-01	7.0E-01
K13244	c-di-GMP-specific phosphodiesterase [EC:3.1.4.52]	1.02	0.96	1.08	5.4E-01	7.0E-01
K05887	quinate/shikimate dehydrogenase [EC:1.1.1.282]	1.02	0.96	1.08	5.4E-01	7.0E-01
K01169	ribonuclease I (enterobacter ribonuclease) [EC:4.6.1.21]	1.02	0.96	1.08	5.4E-01	7.0E-01
K00057	glycerol-3-phosphate dehydrogenase (NAD(P)+) [EC:1.1.1.94]	1.02	0.96	1.08	5.4E-01	7.0E-01

Predictor	Description	HR	2.5% CI	97.5% CI	P	FDR-adjusted P
K03395	aminoglycoside 3-N-acetyltransferase I [EC:2.3.1.60]	0.98	0.93	1.04	5.4E-01	7.0E-01
K12674	(carboxyethyl)arginine beta-lactam-synthase [EC:6.3.3.4]	0.98	0.93	1.04	5.4E-01	7.0E-01
K12675	clavaminate synthase [EC:1.14.11.21]	0.98	0.93	1.04	5.4E-01	7.0E-01
K12673	N2-(2-carboxyethyl)arginine synthase [EC:2.5.1.66]	0.98	0.93	1.04	5.4E-01	7.0E-01
K03439	tRNA (guanine-N7-)-methyltransferase [EC:2.1.1.33]	0.98	0.92	1.05	5.4E-01	7.0E-01
K02774	galactitol PTS system EIIB component [EC:2.7.1.200]	1.02	0.96	1.08	5.4E-01	7.0E-01
K02108	F-type H+-transporting ATPase subunit a	0.98	0.93	1.04	5.4E-01	7.0E-01
K05713	2,3-dihydroxyphenylpropionate 1,2-dioxygenase [EC:1.13.11.16]	1.02	0.96	1.08	5.4E-01	7.0E-01
K16370	6-phosphofructokinase 2 [EC:2.7.1.11]	1.02	0.96	1.08	5.4E-01	7.0E-01
K08324	succinate-semialdehyde dehydrogenase [EC:1.2.1.16 1.2.1.24]	1.02	0.96	1.08	5.4E-01	7.0E-01
K01665	para-aminobenzoate synthetase component I [EC:2.6.1.85]	1.02	0.96	1.08	5.5E-01	7.1E-01
K01525	bis(5'-nucleosyl)-tetraphosphatase (symmetrical) [EC:3.6.1.41]	1.02	0.96	1.08	5.5E-01	7.1E-01
K05910	NADH peroxidase [EC:1.11.1.1]	0.98	0.92	1.04	5.5E-01	7.1E-01
K00137	aminobutyraldehyde dehydrogenase [EC:1.2.1.19]	1.02	0.96	1.08	5.5E-01	7.1E-01
K14682	amino-acid N-acetyltransferase [EC:2.3.1.1]	1.02	0.96	1.08	5.5E-01	7.1E-01
K13498	indole-3-glycerol phosphate synthase / phosphoribosylanthranilate isomerase [EC:4.1.1.48 5.3.1.24]	1.02	0.96	1.08	5.5E-01	7.1E-01
K09761	16S rRNA (uracil1498-N3)-methyltransferase [EC:2.1.1.193]	0.98	0.93	1.04	5.5E-01	7.1E-01
K01277	dipeptidyl-peptidase III [EC:3.4.14.4]	0.98	0.93	1.04	5.5E-01	7.1E-01
K00574	cyclopropane-fatty-acyl-phospholipid synthase [EC:2.1.1.79]	1.02	0.96	1.08	5.5E-01	7.1E-01
K00984	streptomycin 3\-adenylyltransferase [EC:2.7.7.47]	1.02	0.96	1.08	5.5E-01	7.1E-01
K02045	sulfate/thiosulfate transport system ATP-binding protein [EC:7.3.2.3]	1.02	0.96	1.08	5.5E-01	7.1E-01
K06175	tRNA pseudouridine65 synthase [EC:5.4.99.26]	1.02	0.96	1.08	5.5E-01	7.1E-01
K00105	alpha-glycerophosphate oxidase [EC:1.1.3.21]	0.98	0.93	1.04	5.5E-01	7.1E-01
K00763	nicotinate phosphoribosyltransferase [EC:6.3.4.21]	1.02	0.96	1.08	5.5E-01	7.1E-01
K06281	hydrogenase large subunit [EC:1.12.99.6]	1.02	0.96	1.08	5.5E-01	7.1E-01
K12660	2-dehydro-3-deoxy-L-rhamnonate aldolase [EC:4.1.2.53]	1.02	0.96	1.08	5.6E-01	7.1E-01
K07588	GTPase [EC:3.6.5.-]	1.02	0.96	1.09	5.6E-01	7.1E-01
K04118	pimeloyl-CoA dehydrogenase [EC:1.3.1.62]	0.98	0.93	1.04	5.6E-01	7.1E-01
K03823	phosphinothricin acetyltransferase [EC:2.3.1.183]	0.98	0.93	1.04	5.6E-01	7.1E-01
K00848	rhamnulokinase [EC:2.7.1.5]	0.98	0.93	1.04	5.6E-01	7.1E-01
K01934	5-formyltetrahydrofolate cyclo-ligase [EC:6.3.3.2]	1.02	0.96	1.08	5.6E-01	7.1E-01
K00897	kanamycin kinase [EC:2.7.1.95]	0.98	0.92	1.04	5.6E-01	7.1E-01
K00558	DNA (cytosine-5)-methyltransferase 1 [EC:2.1.1.37]	0.98	0.93	1.04	5.6E-01	7.1E-01
K00322	NAD(P) transhydrogenase [EC:1.6.1.1]	1.02	0.96	1.08	5.6E-01	7.1E-01
K15740	tetrahydromethanopterin:alpha-L-glutamate ligase [EC:6.3.2.33]	1.02	0.96	1.08	5.6E-01	7.1E-01
K10563	formamidopyrimidine-DNA glycosylase [EC:3.2.2.23 4.2.99.18]	1.02	0.96	1.08	5.6E-01	7.1E-01
K15257	tRNA (mo5U34)-methyltransferase [EC:2.1.1.-]	1.02	0.96	1.08	5.6E-01	7.1E-01
K02298	cytochrome o ubiquinol oxidase subunit I [EC:7.1.1.3]	1.02	0.96	1.08	5.6E-01	7.1E-01
K09472	4-(gamma-glutamylamino)butanal dehydrogenase [EC:1.2.1.99]	1.02	0.96	1.08	5.6E-01	7.1E-01
K02446	fructose-1,6-bisphosphatase II [EC:3.1.3.11]	1.02	0.96	1.08	5.6E-01	7.1E-01
K13876	2-keto-3-deoxy-L-arabinonate dehydratase [EC:4.2.1.43]	0.98	0.93	1.04	5.6E-01	7.2E-01
K02083	allantoate deiminase [EC:3.5.3.9]	1.02	0.96	1.08	5.6E-01	7.2E-01
K09887	dCTP deaminase (dUMP-forming) [EC:3.5.4.30]	1.02	0.96	1.08	5.6E-01	7.2E-01
K00960	DNA-directed RNA polymerase subunit 13 [EC:2.7.7.6]	0.98	0.93	1.04	5.6E-01	7.2E-01

Predictor	Description	HR	2.5% CI	97.5% CI	P	FDR-adjusted P
K07709	two-component system, NtrC family, sensor histidine kinase HydH [EC:2.7.13.3]	1.02	0.96	1.08	5.6E-01	7.2E-01
K01512	acylphosphatase [EC:3.6.1.7]	0.98	0.93	1.04	5.6E-01	7.2E-01
K00631	glycerol-3-phosphate O-acyltransferase [EC:2.3.1.15]	1.02	0.96	1.08	5.6E-01	7.2E-01
K03212	23S rRNA (uracil747-C5)-methyltransferase [EC:2.1.1.189]	1.02	0.96	1.08	5.6E-01	7.2E-01
K13919	propanediol dehydratase medium subunit [EC:4.2.1.28]	1.02	0.96	1.08	5.6E-01	7.2E-01
K02297	cytochrome o ubiquinol oxidase subunit II [EC:7.1.1.3]	1.02	0.96	1.08	5.6E-01	7.2E-01
K02474	UDP-N-acetyl-D-glucosamine/UDP-N-acetyl-D-galactosamine dehydrogenase [EC:1.1.1.136 1.1.1.-]	0.98	0.93	1.04	5.7E-01	7.2E-01
K16157	methane monooxygenase component A alpha chain [EC:1.14.13.25]	0.98	0.93	1.04	5.7E-01	7.2E-01
K16158	methane monooxygenase component A beta chain [EC:1.14.13.25]	0.98	0.93	1.04	5.7E-01	7.2E-01
K14665	amidohydrolase [EC:3.5.1.-]	0.98	0.93	1.04	5.7E-01	7.2E-01
K02109	F-type H+-transporting ATPase subunit b	0.98	0.93	1.04	5.7E-01	7.2E-01
K01589	5-(carboxyamino)imidazole ribonucleotide synthase [EC:6.3.4.18]	0.98	0.93	1.04	5.7E-01	7.2E-01
K01638	malate synthase [EC:2.3.3.9]	1.02	0.96	1.08	5.7E-01	7.2E-01
K06442	23S rRNA (cytidine1920-2'-O)/16S rRNA (cytidine1409-2'-O)-methyltransferase [EC:2.1.1.226 2.1.1.227]	0.98	0.93	1.04	5.7E-01	7.2E-01
K11627	pyrrolysyl-tRNA synthetase [EC:6.1.1.26]	0.98	0.93	1.04	5.7E-01	7.2E-01
K03582	exodeoxyribonuclease V beta subunit [EC:3.1.11.5]	1.02	0.96	1.08	5.7E-01	7.2E-01
K08483	phosphoenolpyruvate-protein phosphotransferase (PTS system enzyme I) [EC:2.7.3.9]	1.02	0.96	1.08	5.7E-01	7.2E-01
K15984	16S rRNA (guanine1516-N2)-methyltransferase [EC:2.1.1.242]	1.02	0.96	1.08	5.8E-01	7.3E-01
K03583	exodeoxyribonuclease V gamma subunit [EC:3.1.11.5]	1.02	0.96	1.08	5.8E-01	7.3E-01
K00104	glycolate oxidase [EC:1.1.3.15]	1.02	0.96	1.08	5.8E-01	7.3E-01
K01006	pyruvate, orthophosphate dikinase [EC:2.7.9.1]	0.98	0.93	1.04	5.8E-01	7.3E-01
K02227	adenosylcobinamide-phosphate synthase [EC:6.3.1.10]	0.98	0.93	1.04	5.8E-01	7.3E-01
K00855	phosphoribulokinase [EC:2.7.1.19]	1.02	0.96	1.08	5.8E-01	7.3E-01
K00772	5'-methylthioadenosine phosphorylase [EC:2.4.2.28]	0.98	0.93	1.04	5.8E-01	7.3E-01
K00384	thioredoxin reductase (NADPH) [EC:1.8.1.9]	0.98	0.93	1.04	5.8E-01	7.3E-01
K01438	acetylornithine deacetylase [EC:3.5.1.16]	0.98	0.93	1.04	5.8E-01	7.3E-01
K01485	cytosine/creatinine deaminase [EC:3.5.4.1 3.5.4.21]	0.98	0.93	1.04	5.8E-01	7.3E-01
K02364	L-serine---[L-seryl-carrier protein] ligase [EC:6.3.2.14 6.2.1.72]	1.02	0.96	1.08	5.8E-01	7.3E-01
K00821	acetylornithine/N-succinyldiaminopimelate aminotransferase [EC:2.6.1.11 2.6.1.17]	0.98	0.93	1.04	5.8E-01	7.3E-01
K01147	exoribonuclease II [EC:3.1.13.1]	1.02	0.96	1.08	5.8E-01	7.3E-01
K00912	tetraacyldisaccharide 4'-kinase [EC:2.7.1.130]	1.02	0.96	1.08	5.8E-01	7.3E-01
K05964	holo-ACP synthase [EC:2.7.7.61]	1.02	0.96	1.08	5.8E-01	7.3E-01
K00852	ribokinase [EC:2.7.1.15]	0.98	0.93	1.04	5.8E-01	7.3E-01
K00014	shikimate dehydrogenase [EC:1.1.1.25]	0.98	0.93	1.04	5.8E-01	7.3E-01
K08318	4-hydroxybutyrate dehydrogenase / sulfolactaldehyde 3-reductase [EC:1.1.1.61 1.1.1.373]	1.02	0.96	1.08	5.8E-01	7.3E-01
K11201	fructose-like PTS system EIIA component [EC:2.7.1.-]	1.02	0.96	1.08	5.8E-01	7.3E-01
K07757	sugar-phosphatase [EC:3.1.3.23]	1.02	0.96	1.08	5.8E-01	7.3E-01
K03788	acid phosphatase (class B) [EC:3.1.3.2]	1.02	0.96	1.08	5.8E-01	7.3E-01
K02082	D-galactosamine 6-phosphate deaminase/isomerase [EC:3.5.99.-]	1.02	0.96	1.08	5.8E-01	7.3E-01
K02840	UDP-D-galactose:(glucosyl)LPS alpha-1,6-D-galactosyltransferase [EC:2.4.1.-]	1.02	0.96	1.08	5.9E-01	7.3E-01
K00943	dTMP kinase [EC:2.7.4.9]	0.98	0.93	1.04	5.9E-01	7.3E-01
K14441	ribosomal protein S12 methylthiotransferase [EC:2.8.4.4]	1.02	0.95	1.09	5.9E-01	7.3E-01
K13609	delta1-piperideine-2-carboxylate reductase [EC:1.5.1.21]	0.98	0.93	1.04	5.9E-01	7.3E-01
K01682	aconitate hydratase 2 / 2-methylisocitrate dehydratase [EC:4.2.1.3 4.2.1.99]	1.02	0.96	1.08	5.9E-01	7.3E-01

Predictor	Description	HR	2.5% CI	97.5% CI	P	FDR-adjusted P
K01716	3-hydroxyacyl-[acyl-carrier protein] dehydratase / trans-2-decenoyl-[acyl-carrier protein] isomerase [EC:4.2.1.59 5.3.3.14]	1.02	0.96	1.08	5.9E-01	7.3E-01
K00161	pyruvate dehydrogenase E1 component alpha subunit [EC:1.2.4.1]	0.98	0.93	1.04	5.9E-01	7.3E-01
K03824	putative acetyltransferase [EC:2.3.1.-]	1.02	0.96	1.08	5.9E-01	7.4E-01
K02552	menaquinone-specific isochorismate synthase [EC:5.4.4.2]	1.02	0.96	1.08	5.9E-01	7.4E-01
K07310	Tat-targeted selenate reductase subunit YnfF [EC:1.97.1.9]	1.02	0.96	1.08	5.9E-01	7.4E-01
K05919	superoxide reductase [EC:1.15.1.2]	0.98	0.93	1.04	5.9E-01	7.4E-01
K00528	ferredoxin/flavodoxin---NADP+ reductase [EC:1.18.1.2 1.19.1.1]	0.98	0.93	1.04	5.9E-01	7.4E-01
K03750	molybdopterin molybdotransferase [EC:2.10.1.1]	1.02	0.96	1.08	5.9E-01	7.4E-01
K01139	GTP diphosphokinase / guanosine-3',5'-bis(diphosphate) 3'-diphosphatase [EC:2.7.6.5 3.1.7.2]	1.02	0.96	1.08	6.0E-01	7.4E-01
K01129	dGTPase [EC:3.1.5.1]	0.98	0.93	1.04	6.0E-01	7.4E-01
K09471	gamma-glutamylputrescine oxidase [EC:1.4.3.-]	1.02	0.96	1.08	6.0E-01	7.4E-01
K12700	non-specific riboncleoside hydrolase [EC:3.2.-.-]	1.02	0.96	1.08	6.0E-01	7.4E-01
K02469	DNA gyrase subunit A [EC:5.6.2.2]	0.98	0.93	1.05	6.0E-01	7.4E-01
K15020	acryloyl-coenzyme A reductase [EC:1.3.1.84]	0.98	0.93	1.04	6.0E-01	7.4E-01
K01725	cyanate lyase [EC:4.2.1.104]	1.02	0.96	1.08	6.0E-01	7.4E-01
K16214	UDP-N-acetylglucosamine kinase [EC:2.7.1.176]	0.98	0.93	1.05	6.0E-01	7.4E-01
K10017	histidine transport system ATP-binding protein [EC:7.4.2.1]	1.02	0.96	1.08	6.0E-01	7.4E-01
K07638	two-component system, OmpR family, osmolarity sensor histidine kinase EnvZ [EC:2.7.13.3]	1.02	0.96	1.08	6.0E-01	7.4E-01
K07701	two-component system, CitB family, sensor histidine kinase DcuS [EC:2.7.13.3]	1.02	0.96	1.08	6.0E-01	7.5E-01
K03052	DNA-directed RNA polymerase subunit G [EC:2.7.7.6]	0.98	0.93	1.05	6.0E-01	7.5E-01
K00937	polyphosphate kinase [EC:2.7.4.1]	0.98	0.93	1.04	6.0E-01	7.5E-01
K01690	phosphogluconate dehydratase [EC:4.2.1.12]	1.02	0.96	1.08	6.0E-01	7.5E-01
K02549	o-succinylbenzoate synthase [EC:4.2.1.113]	1.02	0.96	1.08	6.1E-01	7.5E-01
K00548	5-methyltetrahydrofolate--homocysteine methyltransferase [EC:2.1.1.13]	1.02	0.96	1.08	6.1E-01	7.5E-01
K11216	autoinducer-2 kinase [EC:2.7.1.189]	1.02	0.96	1.08	6.1E-01	7.5E-01
K04032	ethanolamine utilization cobalamin adenosyltransferase [EC:2.5.1.17]	1.02	0.96	1.08	6.1E-01	7.5E-01
K01659	2-methylcitrate synthase [EC:2.3.3.5]	1.02	0.96	1.08	6.1E-01	7.5E-01
K08321	3-hydroxy-5-phosphonooxypentane-2,4-dione thiolase [EC:2.3.1.245]	1.02	0.96	1.08	6.1E-01	7.5E-01
K16651	L-threonine kinase [EC:2.7.1.177]	0.98	0.93	1.05	6.1E-01	7.5E-01
K08304	membrane-bound lytic murein transglycosylase A [EC:4.2.2.-]	1.02	0.96	1.08	6.1E-01	7.5E-01
K00712	poly(glycerol-phosphate) alpha-glucosyltransferase [EC:2.4.1.52]	0.98	0.93	1.05	6.1E-01	7.5E-01
K01740	O-acetylhomoserine (thiol)-lyase [EC:2.5.1.49]	0.99	0.93	1.04	6.1E-01	7.5E-01
K11628	mycocerosic acid synthase [EC:2.3.1.111]	0.98	0.93	1.05	6.1E-01	7.5E-01
K12445	trans enoyl reductase [EC:1.3.1.-]	0.98	0.93	1.05	6.1E-01	7.5E-01
K07682	two-component system, NarL family, sensor histidine kinase DevS [EC:2.7.13.3]	0.98	0.93	1.05	6.1E-01	7.5E-01
K00010	myo-inositol 2-dehydrogenase / D-chiro-inositol 1-dehydrogenase [EC:1.1.1.18 1.1.1.369]	0.98	0.93	1.05	6.1E-01	7.5E-01
K10984	galactosamine PTS system EIIB component [EC:2.7.1.-]	1.02	0.96	1.08	6.1E-01	7.5E-01
K01355	omptin [EC:3.4.23.49]	1.02	0.96	1.08	6.1E-01	7.5E-01
K15587	nickel transport system ATP-binding protein [EC:7.2.2.11]	1.02	0.96	1.08	6.1E-01	7.5E-01
K00529	3-phenylpropionate/trans-cinnamate dioxygenase ferredoxin reductase component [EC:1.18.1.3]	1.02	0.96	1.08	6.1E-01	7.5E-01
K00432	glutathione peroxidase [EC:1.11.1.9]	0.98	0.93	1.04	6.1E-01	7.5E-01
K00215	4-hydroxy-tetrahydrodipicolinate reductase [EC:1.17.1.8]	1.02	0.96	1.08	6.1E-01	7.5E-01
K02299	cytochrome o ubiquinol oxidase subunit III	1.02	0.96	1.08	6.1E-01	7.5E-01
K00868	pyridoxine kinase [EC:2.7.1.35]	0.99	0.93	1.04	6.1E-01	7.5E-01

Predictor	Description	HR	2.5% CI	97.5% CI	P	FDR-adjusted P
K01753	D-serine dehydratase [EC:4.3.1.18]	1.02	0.96	1.08	6.1E-01	7.5E-01
K08311	putative (di)nucleoside polyphosphate hydrolase [EC:3.6.1.-]	1.02	0.96	1.08	6.2E-01	7.5E-01
K15515	sulfoacetaldehyde dehydrogenase [EC:1.2.1.81]	1.02	0.96	1.08	6.2E-01	7.5E-01
K05520	protease I [EC:3.5.1.124]	0.99	0.93	1.04	6.2E-01	7.6E-01
K10545	D-xylose transport system ATP-binding protein [EC:7.5.2.10]	1.02	0.96	1.08	6.2E-01	7.6E-01
K04486	histidinol-phosphatase (PHP family) [EC:3.1.3.15]	1.02	0.96	1.08	6.2E-01	7.6E-01
K03825	L-phenylalanine/L-methionine N-acetyltransferase [EC:2.3.1.53 2.3.1.-]	1.02	0.96	1.08	6.2E-01	7.6E-01
K05365	penicillin-binding protein 1B [EC:2.4.1.129 3.4.16.4]	1.02	0.96	1.08	6.2E-01	7.6E-01
K00098	L-idonate 5-dehydrogenase [EC:1.1.1.264]	1.02	0.96	1.08	6.2E-01	7.6E-01
K00030	isocitrate dehydrogenase (NAD+) [EC:1.1.1.41]	1.02	0.96	1.08	6.2E-01	7.6E-01
K02781	glucitol/sorbitol PTS system EIIA component [EC:2.7.1.198]	1.02	0.96	1.08	6.2E-01	7.6E-01
K00545	catechol O-methyltransferase [EC:2.1.1.6]	0.99	0.93	1.05	6.2E-01	7.6E-01
K08693	2',3'-cyclic-nucleotide 2'-phosphodiesterase / 3'-nucleotidase / 5'-nucleotidase [EC:3.1.4.16 3.1.3.6 3.1.3.5]	0.99	0.93	1.05	6.2E-01	7.6E-01
K05539	tRNA-dihydrouridine synthase A [EC:1.-.-.-]	1.02	0.96	1.08	6.2E-01	7.6E-01
K00467	lactate 2-monooxygenase [EC:1.13.12.4]	0.99	0.93	1.05	6.2E-01	7.6E-01
K14261	alanine-synthesizing transaminase [EC:2.6.1.-]	1.02	0.96	1.08	6.2E-01	7.6E-01
K10824	nickel transport system ATP-binding protein [EC:7.2.2.11]	1.02	0.96	1.08	6.2E-01	7.6E-01
K07320	ribosomal protein L3 glutamine methyltransferase [EC:2.1.1.298]	1.02	0.96	1.08	6.2E-01	7.6E-01
K01667	tryptophanase [EC:4.1.99.1]	1.02	0.96	1.08	6.2E-01	7.6E-01
K06221	2,5-diketo-D-gluconate reductase A [EC:1.1.1.346]	1.02	0.96	1.08	6.2E-01	7.6E-01
K01428	urease subunit alpha [EC:3.5.1.5]	0.99	0.93	1.05	6.2E-01	7.6E-01
K03672	thioredoxin 2 [EC:1.8.1.8]	1.02	0.96	1.08	6.2E-01	7.6E-01
K10539	L-arabinose transport system ATP-binding protein [EC:7.5.2.12]	1.02	0.96	1.08	6.2E-01	7.6E-01
K00795	farnesyl diphosphate synthase [EC:2.5.1.1 2.5.1.10]	1.02	0.96	1.08	6.2E-01	7.6E-01
K00656	formate C-acetyltransferase [EC:2.3.1.54]	0.99	0.93	1.05	6.2E-01	7.6E-01
K12745	deacetoxycephalosporin-C hydroxylase [EC:1.14.11.26]	0.99	0.93	1.05	6.3E-01	7.6E-01
K12744	deacetoxycephalosporin-C synthase [EC:1.14.20.1]	0.99	0.93	1.05	6.3E-01	7.6E-01
K04126	isopenicillin-N synthase [EC:1.21.3.1]	0.99	0.93	1.05	6.3E-01	7.6E-01
K12743	N-(5-amino-5-carboxypentanoyl)-L-cysteinyl-D-valine synthase [EC:6.3.2.26]	0.99	0.93	1.05	6.3E-01	7.6E-01
K15734	all-trans-retinol dehydrogenase (NAD+) [EC:1.1.1.105]	0.99	0.93	1.05	6.3E-01	7.6E-01
K01850	chorismate mutase [EC:5.4.99.5]	0.99	0.93	1.05	6.3E-01	7.6E-01
K01775	alanine racemase [EC:5.1.1.1]	1.01	0.96	1.08	6.3E-01	7.6E-01
K00024	malate dehydrogenase [EC:1.1.1.37]	1.02	0.96	1.08	6.3E-01	7.6E-01
K01613	phosphatidylserine decarboxylase [EC:4.1.1.65]	1.02	0.95	1.08	6.3E-01	7.6E-01
K07642	two-component system, OmpR family, sensor histidine kinase BaeS [EC:2.7.13.3]	1.01	0.96	1.08	6.3E-01	7.6E-01
K01258	tripeptide aminopeptidase [EC:3.4.11.4]	1.01	0.96	1.08	6.3E-01	7.6E-01
K01788	N-acylglucosamine-6-phosphate 2-epimerase [EC:5.1.3.9]	1.01	0.96	1.08	6.3E-01	7.6E-01
K10778	AraC family transcriptional regulator, regulatory protein of adaptative response / methylated-DNA-[protein]-cysteine methyltransferase [EC:2.1.1.63]	1.01	0.96	1.08	6.3E-01	7.6E-01
K01430	urease subunit gamma [EC:3.5.1.5]	0.99	0.93	1.05	6.3E-01	7.6E-01
K01826	5-carboxymethyl-2-hydroxymuconate isomerase [EC:5.3.3.10]	1.01	0.96	1.08	6.3E-01	7.6E-01
K10794	D-proline reductase (dithiol) PrdB [EC:1.21.4.1]	0.99	0.93	1.05	6.3E-01	7.6E-01
K12138	hydrogenase-4 component C [EC:1.-.-.-]	1.01	0.96	1.08	6.3E-01	7.6E-01
K00901	diacylglycerol kinase (ATP) [EC:2.7.1.107]	0.99	0.93	1.04	6.3E-01	7.6E-01
K03922	acyl-[acyl-carrier-protein] desaturase [EC:1.14.19.2]	0.99	0.93	1.05	6.3E-01	7.6E-01

Predictor	Description	HR	2.5% CI	97.5% CI	P	FDR-adjusted P
K07106	N-acetylmuramic acid 6-phosphate etherase [EC:4.2.1.126]	0.99	0.93	1.05	6.4E-01	7.7E-01
K00605	aminomethyltransferase [EC:2.1.2.10]	1.02	0.95	1.08	6.4E-01	7.7E-01
K01625	2-dehydro-3-deoxyphosphogluconate aldolase / (4S)-4-hydroxy-2-oxoglutarate aldolase [EC:4.1.2.14 4.1.3.42]	0.99	0.93	1.05	6.4E-01	7.7E-01
K03732	ATP-dependent RNA helicase RhlB [EC:3.6.4.13]	1.01	0.96	1.08	6.4E-01	7.7E-01
K02848	heptose I phosphotransferase [EC:2.7.1.-]	1.01	0.96	1.08	6.4E-01	7.7E-01
K01787	N-acylglucosamine 2-epimerase [EC:5.1.3.8]	0.99	0.93	1.04	6.4E-01	7.7E-01
K03815	xanthosine phosphorylase [EC:2.4.2.-]	1.01	0.96	1.08	6.4E-01	7.7E-01
K03775	FKBP-type peptidyl-prolyl cis-trans isomerase SlyD [EC:5.2.1.8]	0.99	0.93	1.04	6.4E-01	7.7E-01
K01647	citrate synthase [EC:2.3.3.1]	0.99	0.93	1.05	6.4E-01	7.7E-01
K04568	elongation factor P--(R)-beta-lysine ligase [EC:6.3.1.-]	1.01	0.96	1.08	6.4E-01	7.7E-01
K01852	lanosterol synthase [EC:5.4.99.7]	0.99	0.93	1.05	6.4E-01	7.7E-01
K11410	short-chain 2-methylacyl-CoA dehydrogenase [EC:1.3.8.5]	1.01	0.96	1.08	6.5E-01	7.7E-01
K03928	carboxylesterase [EC:3.1.1.1]	0.99	0.93	1.05	6.5E-01	7.7E-01
K00645	[acyl-carrier-protein] S-malonyltransferase [EC:2.3.1.39]	1.01	0.95	1.08	6.5E-01	7.8E-01
K01741	DNA-(apurinic or apyrimidinic site) lyase [EC:4.2.99.18]	0.99	0.93	1.05	6.5E-01	7.8E-01
K03919	DNA oxidative demethylase [EC:1.14.11.33]	1.01	0.96	1.08	6.5E-01	7.8E-01
K08300	ribonuclease E [EC:3.1.26.12]	1.01	0.95	1.08	6.5E-01	7.8E-01
K01699	propanediol dehydratase large subunit [EC:4.2.1.28]	1.01	0.96	1.08	6.5E-01	7.8E-01
K00982	[glutamine synthetase] adenylyltransferase / [glutamine synthetase]-adenylyl-L-tyrosine phosphorylase [EC:2.7.7.42 2.7.7.89]	1.01	0.95	1.08	6.5E-01	7.8E-01
K00335	NADH-quinone oxidoreductase subunit F [EC:7.1.1.2]	0.99	0.93	1.05	6.5E-01	7.8E-01
K12985	(galactosyl)LPS 1,2-glucosyltransferase [EC:2.4.1.-]	1.01	0.95	1.08	6.5E-01	7.8E-01
K12983	UDP-glucose:(glucosyl)LPS beta-1,3-glucosyltransferase [EC:2.4.1.-]	1.01	0.95	1.08	6.5E-01	7.8E-01
K16328	pseudouridine kinase [EC:2.7.1.83]	1.01	0.95	1.08	6.5E-01	7.8E-01
K00788	thiamine-phosphate pyrophosphorylase [EC:2.5.1.3]	1.01	0.95	1.08	6.5E-01	7.8E-01
K01823	isopentenyl-diphosphate Delta-isomerase [EC:5.3.3.2]	0.99	0.93	1.05	6.5E-01	7.8E-01
K13979	alcohol dehydrogenase (NADP+) [EC:1.1.1.2]	0.99	0.93	1.05	6.5E-01	7.8E-01
K07708	two-component system, NtrC family, nitrogen regulation sensor histidine kinase GlnL [EC:2.7.13.3]	1.01	0.95	1.08	6.5E-01	7.8E-01
K06019	pyrophosphatase PpaX [EC:3.6.1.1]	1.01	0.96	1.08	6.5E-01	7.8E-01
K08641	zinc D-Ala-D-Ala dipeptidase [EC:3.4.13.22]	0.99	0.93	1.05	6.5E-01	7.8E-01
K05712	3-(3-hydroxy-phenyl)propionate hydroxylase [EC:1.14.13.127]	1.01	0.96	1.08	6.5E-01	7.8E-01
K03417	methylisocitrate lyase [EC:4.1.3.30]	1.01	0.95	1.08	6.5E-01	7.8E-01
K08305	membrane-bound lytic murein transglycosylase B [EC:4.2.2.-]	1.01	0.96	1.08	6.5E-01	7.8E-01
K13085	phosphatidylinositol-4,5-bisphosphate 4-phosphatase [EC:3.1.3.78]	1.01	0.95	1.08	6.6E-01	7.8E-01
K02478	two-component system, LytTR family, sensor kinase [EC:2.7.13.3]	1.01	0.95	1.08	6.6E-01	7.8E-01
K01950	NAD+ synthase (glutamine-hydrolysing) [EC:6.3.5.1]	0.99	0.93	1.05	6.6E-01	7.8E-01
K12981	KDO transferase III [EC:2.4.99.-]	1.01	0.95	1.08	6.6E-01	7.8E-01
K04765	nucleoside triphosphate diphosphatase [EC:3.6.1.9]	1.01	0.95	1.08	6.6E-01	7.8E-01
K02825	pyrimidine operon attenuation protein / uracil phosphoribosyltransferase [EC:2.4.2.9]	0.99	0.93	1.05	6.6E-01	7.8E-01
K00216	2,3-dihydro-2,3-dihydroxybenzoate dehydrogenase [EC:1.3.1.28]	1.01	0.95	1.08	6.6E-01	7.8E-01
K10150	cysteine synthase / O-phosphoserine sulfhydrylase / cystathionine beta-synthase [EC:2.5.1.47 2.5.1.65 4.2.1.22]	0.99	0.93	1.05	6.6E-01	7.8E-01
K07250	4-aminobutyrate aminotransferase / (S)-3-amino-2-methylpropionate transaminase / 5-aminovalerate transaminase [EC:2.6.1.19 2.6.1.22 2.6.1.48]	1.01	0.95	1.08	6.6E-01	7.8E-01
K05366	penicillin-binding protein 1A [EC:2.4.1.129 3.4.16.4]	1.01	0.95	1.08	6.6E-01	7.9E-01
K00842	NA	1.01	0.96	1.07	6.6E-01	7.9E-01
K01885	glutamyl-tRNA synthetase [EC:6.1.1.17]	0.99	0.93	1.05	6.7E-01	7.9E-01

Predictor	Description	HR	2.5% CI	97.5% CI	P	FDR-adjusted P
K00330	NADH-quinone oxidoreductase subunit A [EC:7.1.1.2]	1.01	0.95	1.08	6.7E-01	7.9E-01
K03800	lipoate---protein ligase [EC:6.3.1.20]	1.01	0.95	1.08	6.7E-01	7.9E-01
K00228	coproporphyrinogen III oxidase [EC:1.3.3.3]	1.01	0.95	1.08	6.7E-01	7.9E-01
K02769	fructose PTS system EIIB component [EC:2.7.1.202]	1.01	0.96	1.07	6.7E-01	7.9E-01
K01493	dCMP deaminase [EC:3.5.4.12]	0.99	0.93	1.05	6.7E-01	7.9E-01
K00950	2-amino-4-hydroxy-6-hydroxymethyldihydropteridine diphosphokinase [EC:2.7.6.3]	1.01	0.95	1.08	6.7E-01	7.9E-01
K00204	4Fe-4S ferredoxin	0.99	0.93	1.05	6.7E-01	7.9E-01
K01585	arginine decarboxylase [EC:4.1.1.19]	1.01	0.95	1.08	6.7E-01	7.9E-01
K01720	2-methylcitrate dehydratase [EC:4.2.1.79]	1.01	0.95	1.08	6.7E-01	7.9E-01
K03684	ribonuclease D [EC:3.1.13.5]	0.99	0.93	1.05	6.7E-01	7.9E-01
K04073	acetaldehyde dehydrogenase [EC:1.2.1.10]	1.01	0.95	1.08	6.7E-01	7.9E-01
K00287	dihydrofolate reductase [EC:1.5.1.3]	0.99	0.93	1.05	6.7E-01	7.9E-01
K00878	hydroxyethylthiazole kinase [EC:2.7.1.50]	0.99	0.93	1.05	6.7E-01	7.9E-01
K00088	IMP dehydrogenase [EC:1.1.1.205]	1.01	0.95	1.07	6.7E-01	7.9E-01
K08280	lipopolysaccharide O-acetyltransferase [EC:2.3.1.-]	0.99	0.93	1.05	6.8E-01	7.9E-01
K03273	D-glycero-D-manno-heptose 1,7-bisphosphate phosphatase [EC:3.1.3.82 3.1.3.83]	1.01	0.95	1.08	6.8E-01	7.9E-01
K01007	pyruvate, water dikinase [EC:2.7.9.2]	0.99	0.93	1.05	6.8E-01	8.0E-01
K06441	ferredoxin hydrogenase gamma subunit [EC:1.12.7.2]	0.99	0.93	1.05	6.8E-01	8.0E-01
K14260	alanine-synthesizing transaminase [EC:2.6.1.66 2.6.1.2]	1.01	0.95	1.08	6.8E-01	8.0E-01
K15241	tetrachlorohydroquinone reductive dehalogenase [EC:1.21.4.5]	1.01	0.96	1.07	6.8E-01	8.0E-01
K00334	NADH-quinone oxidoreductase subunit E [EC:7.1.1.2]	0.99	0.93	1.05	6.8E-01	8.0E-01
K00331	NADH-quinone oxidoreductase subunit B [EC:7.1.1.2]	1.01	0.95	1.08	6.8E-01	8.0E-01
K10806	acyl-CoA thioesterase YciA [EC:3.1.2.-]	1.01	0.95	1.08	6.8E-01	8.0E-01
K03185	2-octaprenyl-6-methoxyphenol hydroxylase [EC:1.14.13.-]	1.01	0.95	1.08	6.8E-01	8.0E-01
K04075	tRNA(Ile)-lysidine synthase [EC:6.3.4.19]	1.01	0.95	1.07	6.8E-01	8.0E-01
K01408	insulysin [EC:3.4.24.56]	1.01	0.95	1.07	6.8E-01	8.0E-01
K09023	aminoacrylate hydrolase [EC:3.5.1.-]	1.01	0.95	1.08	6.8E-01	8.0E-01
K06726	D-ribose pyranase [EC:5.4.99.62]	1.01	0.95	1.08	6.8E-01	8.0E-01
K01769	guanylate cyclase, other [EC:4.6.1.2]	0.99	0.93	1.05	6.8E-01	8.0E-01
K00034	glucose 1-dehydrogenase [EC:1.1.1.47]	0.99	0.93	1.05	6.8E-01	8.0E-01
K03770	peptidyl-prolyl cis-trans isomerase D [EC:5.2.1.8]	1.01	0.95	1.08	6.8E-01	8.0E-01
K00640	serine O-acetyltransferase [EC:2.3.1.30]	1.01	0.95	1.08	6.8E-01	8.0E-01
K13985	N-acyl-phosphatidylethanolamine-hydrolysing phospholipase D [EC:3.1.4.54]	1.01	0.95	1.07	6.8E-01	8.0E-01
K13967	N-acetylmannosamine-6-phosphate 2-epimerase / N-acetylmannosamine kinase [EC:5.1.3.9 2.7.1.60]	0.99	0.93	1.05	6.8E-01	8.0E-01
K05540	tRNA-dihydrouridine synthase B [EC:1.-.-.-]	1.01	0.95	1.07	6.8E-01	8.0E-01
K00758	thymidine phosphorylase [EC:2.4.2.4]	1.01	0.95	1.07	6.8E-01	8.0E-01
K03644	lipoyl synthase [EC:2.8.1.8]	1.01	0.95	1.08	6.8E-01	8.0E-01
K01226	trehalose-6-phosphate hydrolase [EC:3.2.1.93]	1.01	0.95	1.07	6.9E-01	8.0E-01
K07258	serine-type D-Ala-D-Ala carboxypeptidase (penicillin-binding protein 5/6) [EC:3.4.16.4]	1.01	0.95	1.07	6.9E-01	8.0E-01
K02777	sugar PTS system EIIA component [EC:2.7.1.-]	1.01	0.95	1.07	6.9E-01	8.1E-01
K07336	PKHD-type hydroxylase [EC:1.14.11.-]	1.01	0.95	1.07	6.9E-01	8.1E-01
K03841	fructose-1,6-bisphosphatase I [EC:3.1.3.11]	0.99	0.93	1.05	6.9E-01	8.1E-01
K00666	fatty-acyl-CoA synthase [EC:6.2.1.-]	1.01	0.95	1.07	6.9E-01	8.1E-01
K07964	heparanase [EC:3.2.1.166]	1.01	0.95	1.07	6.9E-01	8.1E-01

Predictor	Description	HR	2.5% CI	97.5% CI	P	FDR-adjusted P
K04084	thioredoxin:protein disulfide reductase [EC:1.8.4.16]	0.99	0.93	1.05	6.9E-01	8.1E-01
K16318	tRNA (guanine6-N2)-methyltransferase [EC:2.1.1.256]	1.01	0.95	1.07	7.0E-01	8.1E-01
K00138	aldehyde dehydrogenase [EC:1.2.1.-]	1.01	0.95	1.07	7.0E-01	8.1E-01
K01256	aminopeptidase N [EC:3.4.11.2]	1.01	0.95	1.07	7.0E-01	8.1E-01
K00655	1-acyl-sn-glycerol-3-phosphate acyltransferase [EC:2.3.1.51]	0.99	0.93	1.05	7.0E-01	8.1E-01
K07127	5-hydroxyisourate hydrolase [EC:3.5.2.17]	1.01	0.95	1.07	7.0E-01	8.1E-01
K06859	glucose-6-phosphate isomerase, archaeal [EC:5.3.1.9]	0.99	0.93	1.05	7.0E-01	8.1E-01
K01061	carboxymethylenebutenolidase [EC:3.1.1.45]	1.01	0.95	1.07	7.0E-01	8.1E-01
K01081	5'-nucleotidase [EC:3.1.3.5]	1.01	0.95	1.07	7.0E-01	8.1E-01
K03054	DNA-directed RNA polymerase subunit I [EC:2.7.7.6]	0.99	0.93	1.05	7.0E-01	8.1E-01
K02302	uroporphyrin-III C-methyltransferase / precorrin-2 dehydrogenase / sirohydrochlorin ferrochelatase [EC:2.1.1.107 1.3.1.76 4.99.1.4]	1.01	0.95	1.07	7.0E-01	8.1E-01
K00493	xanthocillin biosynthesis cytochrome P450 monooxygenase [EC:1.14.-.-]	0.99	0.93	1.05	7.0E-01	8.1E-01
K08352	thiosulfate reductase / polysulfide reductase chain A [EC:1.8.5.5]	0.99	0.93	1.05	7.0E-01	8.1E-01
K03574	8-oxo-dGTP diphosphatase [EC:3.6.1.55]	1.01	0.95	1.07	7.0E-01	8.1E-01
K01886	glutaminyl-tRNA synthetase [EC:6.1.1.18]	1.01	0.95	1.08	7.0E-01	8.1E-01
K01155	type II restriction enzyme [EC:3.1.21.4]	1.01	0.95	1.07	7.0E-01	8.1E-01
K09698	nondiscriminating glutamyl-tRNA synthetase [EC:6.1.1.24]	0.99	0.93	1.05	7.0E-01	8.1E-01
K00973	glucose-1-phosphate thymidylyltransferase [EC:2.7.7.24]	1.01	0.95	1.07	7.0E-01	8.1E-01
K01595	phosphoenolpyruvate carboxylase [EC:4.1.1.31]	0.99	0.93	1.05	7.1E-01	8.1E-01
K16593	pimeloyl-[acyl-carrier protein] synthase [EC:1.14.14.46]	1.01	0.95	1.07	7.1E-01	8.1E-01
K02339	DNA polymerase III subunit chi [EC:2.7.7.7]	1.01	0.95	1.07	7.1E-01	8.1E-01
K01524	exopolyphosphatase / guanosine-5'-triphosphate,3'-diphosphate pyrophosphatase [EC:3.6.1.11 3.6.1.40]	0.99	0.93	1.05	7.1E-01	8.2E-01
K00278	L-aspartate oxidase [EC:1.4.3.16]	0.99	0.93	1.05	7.1E-01	8.2E-01
K01920	glutathione synthase [EC:6.3.2.3]	1.01	0.95	1.07	7.1E-01	8.2E-01
K00123	formate dehydrogenase major subunit [EC:1.17.1.9]	1.01	0.95	1.07	7.1E-01	8.2E-01
K01807	ribose 5-phosphate isomerase A [EC:5.3.1.6]	1.01	0.95	1.07	7.1E-01	8.2E-01
K15863	NADH-quinone oxidoreductase subunit L/M [EC:7.1.1.2]	0.99	0.93	1.05	7.1E-01	8.2E-01
K00108	choline dehydrogenase [EC:1.1.99.1]	1.01	0.95	1.07	7.1E-01	8.2E-01
K13920	propanediol dehydratase small subunit [EC:4.2.1.28]	1.01	0.95	1.07	7.1E-01	8.2E-01
K12960	5-methylthioadenosine/S-adenosylhomocysteine deaminase [EC:3.5.4.31 3.5.4.28]	0.99	0.93	1.05	7.1E-01	8.2E-01
K00549	5-methyltetrahydropteroyltriglutamate--homocysteine methyltransferase [EC:2.1.1.14]	0.99	0.93	1.05	7.1E-01	8.2E-01
K00823	4-aminobutyrate aminotransferase [EC:2.6.1.19]	0.99	0.93	1.05	7.1E-01	8.2E-01
K00341	NADH-quinone oxidoreductase subunit L [EC:7.1.1.2]	1.01	0.95	1.08	7.1E-01	8.2E-01
K10222	2,6-dioxo-6-phenylhexa-3-enoate hydrolase [EC:3.7.1.8]	0.99	0.93	1.05	7.2E-01	8.2E-01
K08689	biphenyl 2,3-dioxygenase subunit alpha [EC:1.14.12.18]	0.99	0.93	1.05	7.2E-01	8.2E-01
K00462	biphenyl-2,3-diol 1,2-dioxygenase [EC:1.13.11.39]	0.99	0.93	1.05	7.2E-01	8.2E-01
K09994	(aminoalkyl)phosphonate N-acetyltransferase [EC:2.3.1.280]	1.01	0.95	1.07	7.2E-01	8.2E-01
K00339	NADH-quinone oxidoreductase subunit J [EC:7.1.1.2]	1.01	0.95	1.08	7.2E-01	8.2E-01
K00768	nicotinate-nucleotide--dimethylbenzimidazole phosphoribosyltransferase [EC:2.4.2.21]	1.01	0.95	1.08	7.2E-01	8.2E-01
K05396	D-cysteine desulfhydrase [EC:4.4.1.15]	1.01	0.95	1.07	7.2E-01	8.2E-01
K00609	aspartate carbamoyltransferase catalytic subunit [EC:2.1.3.2]	1.01	0.95	1.07	7.2E-01	8.2E-01
K01626	3-deoxy-7-phosphoheptulonate synthase [EC:2.5.1.54]	0.99	0.93	1.05	7.2E-01	8.2E-01
K13252	putrescine carbamoyltransferase [EC:2.1.3.6]	0.99	0.93	1.05	7.2E-01	8.2E-01
K01497	GTP cyclohydrolase II [EC:3.5.4.25]	1.01	0.95	1.07	7.2E-01	8.2E-01

Predictor	Description	HR	2.5% CI	97.5% CI	P	FDR-adjusted P
K00881	allose kinase [EC:2.7.1.55]	0.99	0.93	1.05	7.2E-01	8.2E-01
K00483	4-hydroxyphenylacetate 3-monooxygenase [EC:1.14.14.9]	1.01	0.95	1.07	7.2E-01	8.2E-01
K03981	thiol:disulfide interchange protein DsbC [EC:5.3.4.1]	1.01	0.95	1.07	7.2E-01	8.2E-01
K00337	NADH-quinone oxidoreductase subunit H [EC:7.1.1.2]	1.01	0.95	1.07	7.2E-01	8.2E-01
K01958	pyruvate carboxylase [EC:6.4.1.1]	0.99	0.93	1.05	7.2E-01	8.3E-01
K05921	5-oxopent-3-ene-1,2,5-tricarboxylate decarboxylase / 2-hydroxyhepta-2,4-diene-1,7-dioate isomerase [EC:4.1.1.68 5.3.3.-]	1.01	0.95	1.07	7.2E-01	8.3E-01
K03186	flavin prenyltransferase [EC:2.5.1.129]	1.01	0.95	1.07	7.2E-01	8.3E-01
K00222	Delta14-sterol reductase [EC:1.3.1.70]	0.99	0.93	1.05	7.2E-01	8.3E-01
K13542	uroporphyrinogen III methyltransferase / synthase [EC:2.1.1.107 4.2.1.75]	0.99	0.93	1.05	7.2E-01	8.3E-01
K04094	methylenetetrahydrofolate--tRNA-(uracil-5-)-methyltransferase [EC:2.1.1.74]	0.99	0.93	1.05	7.3E-01	8.3E-01
K01069	hydroxyacylglutathione hydrolase [EC:3.1.2.6]	1.01	0.95	1.07	7.3E-01	8.3E-01
K01547	potassium-transporting ATPase ATP-binding subunit [EC:7.2.2.6]	1.01	0.95	1.08	7.3E-01	8.3E-01
K03818	putative colanic acid biosynthesis acetyltransferase WcaF [EC:2.3.1.-]	1.01	0.95	1.07	7.3E-01	8.3E-01
K01816	hydroxypyruvate isomerase [EC:5.3.1.22]	0.99	0.93	1.05	7.3E-01	8.3E-01
K00641	homoserine O-acetyltransferase/O-succinyltransferase [EC:2.3.1.31 2.3.1.46]	0.99	0.93	1.05	7.3E-01	8.3E-01
K01751	diaminopropionate ammonia-lyase [EC:4.3.1.15]	1.01	0.95	1.07	7.3E-01	8.3E-01
K03784	purine-nucleoside phosphorylase [EC:2.4.2.1]	0.99	0.93	1.05	7.3E-01	8.3E-01
K13281	UV DNA damage endonuclease [EC:3.-.-.-]	0.99	0.93	1.05	7.3E-01	8.3E-01
K00611	ornithine carbamoyltransferase [EC:2.1.3.3]	0.99	0.93	1.05	7.3E-01	8.3E-01
K01903	succinyl-CoA synthetase beta subunit [EC:6.2.1.5]	1.01	0.95	1.07	7.3E-01	8.3E-01
K03060	DNA-directed RNA polymerase subunit omega [EC:2.7.7.6]	0.99	0.93	1.05	7.3E-01	8.3E-01
K00362	nitrite reductase (NADH) large subunit [EC:1.7.1.15]	0.99	0.93	1.05	7.3E-01	8.3E-01
K01974	RNA 3'-terminal phosphate cyclase (ATP) [EC:6.5.1.4]	1.01	0.95	1.07	7.4E-01	8.3E-01
K01419	ATP-dependent HslUV protease, peptidase subunit HslV [EC:3.4.25.2]	1.01	0.95	1.07	7.4E-01	8.3E-01
K00632	acetyl-CoA acyltransferase [EC:2.3.1.16]	1.01	0.95	1.07	7.4E-01	8.4E-01
K00705	4-alpha-glucanotransferase [EC:2.4.1.25]	0.99	0.93	1.05	7.4E-01	8.4E-01
K01584	arginine decarboxylase [EC:4.1.1.19]	1.01	0.95	1.07	7.4E-01	8.4E-01
K02554	2-keto-4-pentenoate hydratase [EC:4.2.1.80]	1.01	0.95	1.07	7.4E-01	8.4E-01
K11785	1,4-dihydroxy-6-naphthoate synthase [EC:1.14.-.-]	0.99	0.93	1.05	7.4E-01	8.4E-01
K05816	sn-glycerol 3-phosphate transport system ATP-binding protein [EC:7.6.2.10]	1.01	0.95	1.07	7.4E-01	8.4E-01
K00065	2-dehydro-3-deoxy-D-gluconate 5-dehydrogenase [EC:1.1.1.127]	0.99	0.93	1.05	7.4E-01	8.4E-01
K01902	succinyl-CoA synthetase alpha subunit [EC:6.2.1.5]	1.01	0.95	1.07	7.4E-01	8.4E-01
K01117	sphingomyelin phosphodiesterase [EC:3.1.4.12]	0.99	0.93	1.05	7.4E-01	8.4E-01
K01908	propionyl-CoA synthetase [EC:6.2.1.17]	1.01	0.95	1.07	7.4E-01	8.4E-01
K01461	N-acyl-D-glutamate deacylase [EC:3.5.1.82]	0.99	0.93	1.05	7.4E-01	8.4E-01
K01002	phosphoglycerol transferase [EC:2.7.8.20]	1.01	0.95	1.07	7.4E-01	8.4E-01
K13381	bifunctional chitinase/lysozyme [EC:3.2.1.14 3.2.1.17]	1.01	0.95	1.07	7.4E-01	8.4E-01
K01687	dihydroxy-acid dehydratase [EC:4.2.1.9]	0.99	0.93	1.05	7.4E-01	8.4E-01
K11949	4-(2-carboxyphenyl)-2-oxobut-3-enoate aldolase [EC:4.1.2.34]	1.01	0.95	1.07	7.5E-01	8.4E-01
K05308	gluconate/galactonate dehydratase [EC:4.2.1.140]	0.99	0.93	1.05	7.5E-01	8.4E-01
K14587	protein sgcE [EC:5.1.3.-]	1.01	0.95	1.07	7.5E-01	8.4E-01
K07559	putative RNA 2'-phosphotransferase [EC:2.7.1.-]	1.01	0.95	1.07	7.5E-01	8.4E-01
K01295	glutamate carboxypeptidase [EC:3.4.17.11]	1.01	0.95	1.07	7.5E-01	8.4E-01
K00293	saccharopine dehydrogenase (NADP+, L-glutamate forming) [EC:1.5.1.10]	0.99	0.93	1.05	7.5E-01	8.4E-01

Predictor	Description	HR	2.5% CI	97.5% CI	P	FDR-adjusted P
K13821	RHH-type transcriptional regulator, proline utilization regulon repressor / proline dehydrogenase / delta 1-pyrroline-5-carboxylate dehydrogenase [EC:1.5.5.2 1.2.1.88]	1.01	0.95	1.07	7.5E-01	8.4E-01
K00793	riboflavin synthase [EC:2.5.1.9]	1.01	0.95	1.08	7.5E-01	8.5E-01
K02517	Kdo2-lipid IVA lauroyltransferase/acyltransferase [EC:2.3.1.241 2.3.1.-]	0.99	0.93	1.05	7.5E-01	8.5E-01
K00568	2-polyprenyl-6-hydroxyphenyl methylase / 3-demethylubiquinone-9 3-methyltransferase [EC:2.1.1.222 2.1.1.64]	1.01	0.95	1.07	7.5E-01	8.5E-01
K02233	adenosylcobinamide-GDP ribazoletransferase [EC:2.7.8.26]	1.01	0.95	1.07	7.6E-01	8.5E-01
K00826	branched-chain amino acid aminotransferase [EC:2.6.1.42]	0.99	0.93	1.05	7.6E-01	8.5E-01
K06183	16S rRNA pseudouridine516 synthase [EC:5.4.99.19]	0.99	0.93	1.05	7.6E-01	8.5E-01
K01534	Zn2+/Cd2+-exporting ATPase [EC:7.2.2.12 7.2.2.21]	0.99	0.93	1.05	7.6E-01	8.5E-01
K10680	N-ethylmaleimide reductase [EC:1.-.-.-]	1.01	0.95	1.07	7.6E-01	8.5E-01
K11784	cyclic dehypoxanthinyl futalosine synthase [EC:1.21.98.1]	0.99	0.93	1.05	7.6E-01	8.5E-01
K01126	glycerophosphoryl diester phosphodiesterase [EC:3.1.4.46]	0.99	0.93	1.05	7.6E-01	8.6E-01
K06001	tryptophan synthase beta chain [EC:4.2.1.20]	1.01	0.95	1.07	7.6E-01	8.6E-01
K00684	leucyl/phenylalanyl-tRNA---protein transferase [EC:2.3.2.6]	1.01	0.95	1.07	7.6E-01	8.6E-01
K01095	phosphatidylglycerophosphatase A [EC:3.1.3.27]	1.01	0.95	1.07	7.6E-01	8.6E-01
K15018	3-hydroxypropionyl-coenzyme A synthetase [EC:6.2.1.36]	0.99	0.93	1.05	7.6E-01	8.6E-01
K09565	peptidyl-prolyl isomerase F (cyclophilin D) [EC:5.2.1.8]	0.99	0.93	1.05	7.6E-01	8.6E-01
K13000	mannosyltransferase [EC:2.4.1.-]	1.01	0.95	1.07	7.7E-01	8.6E-01
K01782	3-hydroxyacyl-CoA dehydrogenase / enoyl-CoA hydratase / 3-hydroxybutyryl-CoA epimerase [EC:1.1.1.35 4.2.1.17 5.1.2.3]	1.01	0.95	1.07	7.7E-01	8.6E-01
K13378	NADH-quinone oxidoreductase subunit C/D [EC:7.1.1.2]	0.99	0.93	1.05	7.7E-01	8.6E-01
K01429	urease subunit beta [EC:3.5.1.5]	1.01	0.95	1.07	7.7E-01	8.6E-01
K00112	glycerol-3-phosphate dehydrogenase subunit B [EC:1.1.5.3]	0.99	0.93	1.05	7.7E-01	8.6E-01
K01658	anthranilate synthase component II [EC:4.1.3.27]	0.99	0.93	1.05	7.7E-01	8.6E-01
K12986	1,5-rhamnosyltransferase [EC:2.4.1.-]	1.01	0.95	1.07	7.7E-01	8.6E-01
K16153	glycogen phosphorylase/synthase [EC:2.4.1.1 2.4.1.11]	0.99	0.93	1.05	7.7E-01	8.6E-01
K03501	16S rRNA (guanine527-N7)-methyltransferase [EC:2.1.1.170]	1.01	0.95	1.07	7.7E-01	8.6E-01
K00151	5-carboxymethyl-2-hydroxymuconic-semialdehyde dehydrogenase [EC:1.2.1.60]	1.01	0.95	1.07	7.7E-01	8.6E-01
K03431	phosphoglucosamine mutase [EC:5.4.2.10]	0.99	0.93	1.05	7.7E-01	8.6E-01
K02533	tRNA/rRNA methyltransferase [EC:2.1.1.-]	1.01	0.95	1.07	7.7E-01	8.6E-01
K02342	DNA polymerase III subunit epsilon [EC:2.7.7.7]	0.99	0.93	1.05	7.7E-01	8.6E-01
K02334	DNA polymerase bacteriophage-type [EC:2.7.7.7]	1.01	0.95	1.07	7.7E-01	8.6E-01
K07644	two-component system, OmpR family, heavy metal sensor histidine kinase CusS [EC:2.7.13.3]	1.01	0.95	1.07	7.7E-01	8.6E-01
K10620	2,3-dihydroxy-2,3-dihydro-p-cumate dehydrogenase [EC:1.3.1.58]	0.99	0.93	1.05	7.7E-01	8.6E-01
K00891	shikimate kinase [EC:2.7.1.71]	0.99	0.93	1.05	7.7E-01	8.6E-01
K00113	glycerol-3-phosphate dehydrogenase subunit C	0.99	0.93	1.05	7.7E-01	8.6E-01
K03119	taurine dioxygenase [EC:1.14.11.17]	1.01	0.95	1.07	7.7E-01	8.6E-01
K01223	6-phospho-beta-glucosidase [EC:3.2.1.86]	0.99	0.93	1.05	7.7E-01	8.6E-01
K03785	3-dehydroquinate dehydratase I [EC:4.2.1.10]	1.01	0.95	1.07	7.7E-01	8.6E-01
K00285	D-amino-acid dehydrogenase [EC:1.4.5.1]	1.01	0.95	1.07	7.7E-01	8.6E-01
K03276	UDP-glucose/galactose:(glucosyl)LPS alpha-1,2-glucosyl/galactosyltransferase [EC:2.4.1.-]	1.01	0.95	1.07	7.7E-01	8.6E-01
K00147	glutamate-5-semialdehyde dehydrogenase [EC:1.2.1.41]	1.01	0.95	1.07	7.8E-01	8.6E-01
K00223	Delta24(24(1))-sterol reductase [EC:1.3.1.71]	1.01	0.95	1.07	7.8E-01	8.6E-01
K03782	catalase-peroxidase [EC:1.11.1.21]	1.01	0.95	1.07	7.8E-01	8.6E-01
K00626	acetyl-CoA C-acetyltransferase [EC:2.3.1.9]	0.99	0.93	1.05	7.8E-01	8.6E-01
K01783	ribulose-phosphate 3-epimerase [EC:5.1.3.1]	0.99	0.93	1.05	7.8E-01	8.6E-01

Predictor	Description	HR	2.5% CI	97.5% CI	P	FDR-adjusted P
K00364	GMP reductase [EC:1.7.1.7]	1.01	0.95	1.07	7.8E-01	8.6E-01
K06879	7-cyano-7-deazaguanine reductase [EC:1.7.1.13]	0.99	0.93	1.05	7.8E-01	8.6E-01
K01464	dihydropyrimidinase [EC:3.5.2.2]	0.99	0.93	1.05	7.8E-01	8.7E-01
K01466	allantoinase [EC:3.5.2.5]	1.01	0.95	1.07	7.8E-01	8.7E-01
K02558	UDP-N-acetylmuramate: L-alanyl-gamma-D-glutamyl-meso-diaminopimelate ligase [EC:6.3.2.45]	1.01	0.95	1.07	7.8E-01	8.7E-01
K01637	isocitrate lyase [EC:4.1.3.1]	1.01	0.95	1.07	7.9E-01	8.7E-01
K03148	sulfur carrier protein ThiS adenylyltransferase [EC:2.7.7.73]	0.99	0.93	1.05	7.9E-01	8.7E-01
K03814	monofunctional glycosyltransferase [EC:2.4.1.129]	0.99	0.94	1.05	7.9E-01	8.7E-01
K00390	phosphoadenosine phosphosulfate reductase [EC:1.8.4.8 1.8.4.10]	1.01	0.95	1.07	7.9E-01	8.7E-01
K11178	xanthine dehydrogenase YagS FAD-binding subunit [EC:1.17.1.4]	0.99	0.93	1.05	7.9E-01	8.7E-01
K11177	xanthine dehydrogenase YagR molybdenum-binding subunit [EC:1.17.1.4]	0.99	0.93	1.05	7.9E-01	8.7E-01
K00767	nicotinate-nucleotide pyrophosphorylase (carboxylating) [EC:2.4.2.19]	0.99	0.94	1.05	7.9E-01	8.7E-01
K11175	phosphoribosylglycinamide formyltransferase 1 [EC:2.1.2.2]	1.01	0.95	1.07	7.9E-01	8.7E-01
K15633	2,3-bisphosphoglycerate-independent phosphoglycerate mutase [EC:5.4.2.12]	1.01	0.95	1.07	7.9E-01	8.7E-01
K14187	chorismate mutase / prephenate dehydrogenase [EC:5.4.99.5 1.3.1.12]	1.01	0.95	1.07	7.9E-01	8.7E-01
K11072	spermidine/putrescine transport system ATP-binding protein [EC:7.6.2.11]	0.99	0.94	1.05	7.9E-01	8.7E-01
K05916	nitric oxide dioxygenase [EC:1.14.12.17]	1.01	0.95	1.07	7.9E-01	8.7E-01
K05556	ketoreductase RED1 [EC:1.1.1.-]	0.99	0.94	1.05	7.9E-01	8.7E-01
K01389	neprilysin [EC:3.4.24.11]	1.01	0.95	1.07	8.0E-01	8.7E-01
K12524	bifunctional aspartokinase / homoserine dehydrogenase 1 [EC:2.7.2.4 1.1.1.3]	0.99	0.94	1.05	8.0E-01	8.7E-01
K00116	malate dehydrogenase (quinone) [EC:1.1.5.4]	1.01	0.95	1.07	8.0E-01	8.8E-01
K01222	6-phospho-beta-glucosidase [EC:3.2.1.86]	0.99	0.93	1.05	8.0E-01	8.8E-01
K11931	poly-beta-1,6-N-acetyl-D-glucosamine N-deacetylase [EC:3.5.1.-]	1.01	0.95	1.07	8.0E-01	8.8E-01
K01679	fumarate hydratase, class II [EC:4.2.1.2]	1.01	0.95	1.07	8.0E-01	8.8E-01
K02010	iron(III) transport system ATP-binding protein [EC:7.2.2.7]	0.99	0.93	1.05	8.0E-01	8.8E-01
K07246	tartrate dehydrogenase/decarboxylase / D-malate dehydrogenase [EC:1.1.1.93 4.1.1.73 1.1.1.83]	1.01	0.95	1.07	8.0E-01	8.8E-01
K00117	quinoprotein glucose dehydrogenase [EC:1.1.5.2]	1.01	0.95	1.07	8.0E-01	8.8E-01
K13689	beta-1,4-N-acetylgalactosaminyltransferase [EC:2.4.1.-]	0.99	0.94	1.05	8.0E-01	8.8E-01
K00101	L-lactate dehydrogenase (cytochrome) [EC:1.1.2.3]	1.01	0.95	1.07	8.0E-01	8.8E-01
K14977	(S)-ureidoglycine aminohydrolase [EC:3.5.3.26]	1.01	0.95	1.07	8.1E-01	8.8E-01
K04940	opine dehydrogenase [EC:1.5.1.28]	0.99	0.93	1.05	8.1E-01	8.8E-01
K02492	glutamyl-tRNA reductase [EC:1.2.1.70]	1.01	0.95	1.07	8.1E-01	8.8E-01
K08281	nicotinamidase/pyrazinamidase [EC:3.5.1.19 3.5.1.-]	1.01	0.95	1.07	8.1E-01	8.8E-01
K01451	hippurate hydrolase [EC:3.5.1.32]	0.99	0.94	1.05	8.1E-01	8.8E-01
K04091	alkanesulfonate monooxygenase [EC:1.14.14.5 1.14.14.34]	1.01	0.95	1.07	8.1E-01	8.8E-01
K14459	hexosaminidase [EC:3.2.1.52]	1.01	0.95	1.07	8.1E-01	8.8E-01
K15256	tRNA (cmo5U34)-methyltransferase [EC:2.1.1.-]	1.01	0.95	1.07	8.1E-01	8.8E-01
K00845	glucokinase [EC:2.7.1.2]	1.01	0.95	1.07	8.1E-01	8.8E-01
K10012	undecaprenyl-phosphate 4-deoxy-4-formamido-L-arabinose transferase [EC:2.4.2.53]	1.01	0.95	1.07	8.1E-01	8.8E-01
K02744	N-acetylgalactosamine PTS system EIIA component [EC:2.7.1.-]	0.99	0.94	1.05	8.1E-01	8.8E-01
K01759	lactoylglutathione lyase [EC:4.4.1.5]	1.01	0.95	1.07	8.1E-01	8.9E-01
K03801	lipoyl(octanoyl) transferase [EC:2.3.1.181]	1.01	0.95	1.07	8.1E-01	8.9E-01
K02654	leader peptidase (prepilin peptidase) / N-methyltransferase [EC:3.4.23.43 2.1.1.-]	0.99	0.93	1.05	8.1E-01	8.9E-01
K02377	GDP-L-fucose synthase [EC:1.1.1.271]	1.01	0.95	1.07	8.1E-01	8.9E-01

Predictor	Description	HR	2.5% CI	97.5% CI	P	FDR-adjusted P
K03769	peptidyl-prolyl cis-trans isomerase C [EC:5.2.1.8]	0.99	0.93	1.05	8.1E-01	8.9E-01
K03771	peptidyl-prolyl cis-trans isomerase SurA [EC:5.2.1.8]	0.99	0.94	1.05	8.1E-01	8.9E-01
K03077	L-ribulose-5-phosphate 4-epimerase [EC:5.1.3.4]	0.99	0.94	1.05	8.1E-01	8.9E-01
K13245	c-di-GMP-specific phosphodiesterase [EC:3.1.4.52]	0.99	0.94	1.05	8.1E-01	8.9E-01
K03517	quinolinate synthase [EC:2.5.1.72]	0.99	0.94	1.05	8.2E-01	8.9E-01
K00681	gamma-glutamyltranspeptidase / glutathione hydrolase [EC:2.3.2.2 3.4.19.13]	1.01	0.95	1.07	8.2E-01	8.9E-01
K02615	3-oxo-5,6-didehydrosuberyl-CoA/3-oxoadipyl-CoA thiolase [EC:2.3.1.223 2.3.1.174]	1.01	0.95	1.07	8.2E-01	8.9E-01
K01792	glucose-6-phosphate 1-epimerase [EC:5.1.3.15]	1.01	0.95	1.07	8.2E-01	8.9E-01
K01197	hyaluronoglucosaminidase [EC:3.2.1.35]	1.01	0.95	1.07	8.2E-01	8.9E-01
K10804	acyl-CoA thioesterase I [EC:3.1.2.- 3.1.2.2 3.1.1.2 3.1.1.5]	1.01	0.95	1.07	8.2E-01	8.9E-01
K02528	16S rRNA (adenine1518-N6/adenine1519-N6)-dimethyltransferase [EC:2.1.1.182]	1.01	0.95	1.07	8.2E-01	8.9E-01
K01082	3'(2'), 5'-bisphosphate nucleotidase [EC:3.1.3.7]	1.01	0.95	1.07	8.2E-01	8.9E-01
K11214	sedoheptulokinase [EC:2.7.1.14]	0.99	0.94	1.05	8.2E-01	8.9E-01
K00042	2-hydroxy-3-oxopropionate reductase [EC:1.1.1.60]	1.01	0.95	1.07	8.2E-01	8.9E-01
K12506	2-C-methyl-D-erythritol 4-phosphate cytidylyltransferase / 2-C-methyl-D-erythritol 2,4-cyclodiphosphate synthase [EC:2.7.7.60 4.6.1.12]	1.01	0.95	1.07	8.2E-01	8.9E-01
K14465	succinate semialdehyde reductase (NADPH) [EC:1.1.1.-]	1.01	0.95	1.07	8.2E-01	8.9E-01
K15376	gephyrin [EC:2.10.1.1 2.7.7.75]	0.99	0.94	1.05	8.2E-01	8.9E-01
K00306	sarcosine oxidase / L-pipecolate oxidase [EC:1.5.3.1 1.5.3.7]	0.99	0.94	1.05	8.2E-01	8.9E-01
K05982	deoxyribonuclease V [EC:3.1.21.7]	1.01	0.95	1.07	8.3E-01	8.9E-01
K07313	serine/threonine protein phosphatase 1 [EC:3.1.3.16]	1.01	0.95	1.07	8.3E-01	8.9E-01
K01579	aspartate 1-decarboxylase [EC:4.1.1.11]	1.01	0.95	1.07	8.3E-01	8.9E-01
K10026	7-carboxy-7-deazaguanine synthase [EC:4.3.99.3]	1.01	0.95	1.07	8.3E-01	9.0E-01
K15460	tRNA1Val (adenine37-N6)-methyltransferase [EC:2.1.1.223]	1.01	0.95	1.07	8.3E-01	9.0E-01
K01483	ureidoglycolate lyase [EC:4.3.2.3]	1.01	0.95	1.07	8.3E-01	9.0E-01
K00067	dTDP-4-dehydrorhamnose reductase [EC:1.1.1.133]	0.99	0.94	1.05	8.3E-01	9.0E-01
K03830	putative acetyltransferase [EC:2.3.1.-]	0.99	0.94	1.05	8.3E-01	9.0E-01
K02768	fructose PTS system EIIA component [EC:2.7.1.202]	1.01	0.95	1.07	8.3E-01	9.0E-01
K01035	acetate CoA/acetoacetate CoA-transferase beta subunit [EC:2.8.3.8 2.8.3.9]	0.99	0.94	1.05	8.3E-01	9.0E-01
K13953	alcohol dehydrogenase, propanol-preferring [EC:1.1.1.1]	1.01	0.95	1.07	8.4E-01	9.0E-01
K07806	UDP-4-amino-4-deoxy-L-arabinose-oxoglutarate aminotransferase [EC:2.6.1.87]	1.01	0.95	1.07	8.4E-01	9.0E-01
K09483	3-dehydroshikimate dehydratase [EC:4.2.1.118]	0.99	0.94	1.05	8.4E-01	9.0E-01
K01425	glutaminase [EC:3.5.1.2]	0.99	0.94	1.05	8.4E-01	9.0E-01
K13604	bacteriochlorophyllide d C-20 methyltransferase [EC:2.1.1.333]	0.99	0.94	1.05	8.4E-01	9.0E-01
K01058	phospholipase A1/A2 [EC:3.1.1.32 3.1.1.4]	0.99	0.94	1.05	8.4E-01	9.0E-01
K13954	alcohol dehydrogenase [EC:1.1.1.1]	1.01	0.95	1.07	8.4E-01	9.0E-01
K01218	mannan endo-1,4-beta-mannosidase [EC:3.2.1.78]	0.99	0.94	1.05	8.4E-01	9.0E-01
K00183	NA	1.01	0.95	1.07	8.4E-01	9.0E-01
K00387	sulfite oxidase [EC:1.8.3.1]	0.99	0.94	1.05	8.4E-01	9.1E-01
K09458	3-oxoacyl-[acyl-carrier-protein] synthase II [EC:2.3.1.179]	1.01	0.95	1.07	8.4E-01	9.1E-01
K01495	GTP cyclohydrolase IA [EC:3.5.4.16]	0.99	0.94	1.05	8.4E-01	9.1E-01
K03272	D-beta-D-heptose 7-phosphate kinase / D-beta-D-heptose 1-phosphate adenosyltransferase [EC:2.7.1.167 2.7.7.70]	1.01	0.95	1.07	8.4E-01	9.1E-01
K01810	glucose-6-phosphate isomerase [EC:5.3.1.9]	1.01	0.95	1.07	8.4E-01	9.1E-01
K16329	pseudouridylate synthase [EC:4.2.1.70]	1.01	0.95	1.07	8.5E-01	9.1E-01
K00563	23S rRNA (guanine745-N1)-methyltransferase [EC:2.1.1.187]	1.01	0.95	1.07	8.5E-01	9.1E-01

Predictor	Description	HR	2.5% CI	97.5% CI	P	FDR-adjusted P
K02000	glycine betaine/proline transport system ATP-binding protein [EC:7.6.2.9]	0.99	0.94	1.05	8.5E-01	9.1E-01
K00817	histidinol-phosphate aminotransferase [EC:2.6.1.9]	0.99	0.94	1.05	8.5E-01	9.1E-01
K00164	2-oxoglutarate dehydrogenase E1 component [EC:1.2.4.2]	0.99	0.94	1.06	8.5E-01	9.1E-01
K03781	catalase [EC:1.11.1.6]	1.01	0.95	1.07	8.5E-01	9.1E-01
K00239	succinate dehydrogenase / fumarate reductase, flavoprotein subunit [EC:1.3.5.1 1.3.5.4]	0.99	0.94	1.05	8.5E-01	9.1E-01
K00064	D-threo-aldose 1-dehydrogenase [EC:1.1.1.122]	1.01	0.95	1.07	8.5E-01	9.1E-01
K07405	alpha-amylase [EC:3.2.1.1]	0.99	0.94	1.05	8.5E-01	9.1E-01
K01255	leucyl aminopeptidase [EC:3.4.11.1]	1.01	0.95	1.07	8.5E-01	9.1E-01
K01845	glutamate-1-semialdehyde 2,1-aminomutase [EC:5.4.3.8]	0.99	0.94	1.06	8.5E-01	9.1E-01
K03367	D-alanine--poly(phosphoribitol) ligase subunit 1 [EC:6.1.1.13]	0.99	0.94	1.06	8.5E-01	9.1E-01
K00383	glutathione reductase (NADPH) [EC:1.8.1.7]	1.01	0.95	1.07	8.5E-01	9.1E-01
K08589	gingipain R [EC:3.4.22.37]	1.01	0.95	1.07	8.6E-01	9.1E-01
K00027	malate dehydrogenase (oxaloacetate-decarboxylating) [EC:1.1.1.38]	1.01	0.95	1.07	8.6E-01	9.1E-01
K06177	tRNA pseudouridine32 synthase / 23S rRNA pseudouridine746 synthase [EC:5.4.99.28 5.4.99.29]	0.99	0.94	1.05	8.6E-01	9.1E-01
K07683	two-component system, NarL family, sensor histidine kinase NreB [EC:2.7.13.3]	0.99	0.94	1.06	8.6E-01	9.2E-01
K00968	choline-phosphate cytidylyltransferase [EC:2.7.7.15]	0.99	0.94	1.06	8.6E-01	9.2E-01
K00773	queuine tRNA-ribosyltransferase [EC:2.4.2.29]	0.99	0.94	1.06	8.6E-01	9.2E-01
K00799	glutathione S-transferase [EC:2.5.1.18]	1.01	0.95	1.07	8.6E-01	9.2E-01
K00879	L-fuculokinase [EC:2.7.1.51]	0.99	0.94	1.06	8.6E-01	9.2E-01
K05714	2-hydroxy-6-oxonona-2,4-dienedioate hydrolase [EC:3.7.1.14]	1.01	0.95	1.07	8.6E-01	9.2E-01
K01232	maltose-6'-phosphate glucosidase [EC:3.2.1.122]	0.99	0.94	1.06	8.7E-01	9.2E-01
K13789	geranylgeranyl diphosphate synthase, type II [EC:2.5.1.1 2.5.1.10 2.5.1.29]	0.99	0.94	1.06	8.7E-01	9.2E-01
K03040	DNA-directed RNA polymerase subunit alpha [EC:2.7.7.6]	1.00	0.95	1.07	8.7E-01	9.2E-01
K01568	pyruvate decarboxylase [EC:4.1.1.1]	1.00	0.95	1.06	8.7E-01	9.2E-01
K00654	serine palmitoyltransferase [EC:2.3.1.50]	1.00	0.95	1.06	8.7E-01	9.2E-01
K00638	chloramphenicol O-acetyltransferase type B [EC:2.3.1.28]	1.00	0.94	1.06	8.7E-01	9.3E-01
K01620	threonine aldolase [EC:4.1.2.48]	1.00	0.94	1.06	8.7E-01	9.3E-01
K02036	phosphate transport system ATP-binding protein [EC:7.3.2.1]	1.00	0.94	1.06	8.7E-01	9.3E-01
K15756	2-hydroxy-6-oxo-6-(2'-aminophenyl)hexa-2,4-dienoate hydrolase [EC:3.7.1.13]	1.00	0.95	1.07	8.7E-01	9.3E-01
K03657	DNA helicase II / ATP-dependent DNA helicase PcrA [EC:3.6.4.12]	1.00	0.94	1.06	8.7E-01	9.3E-01
K01262	Xaa-Pro aminopeptidase [EC:3.4.11.9]	1.00	0.94	1.06	8.7E-01	9.3E-01
K00932	propionate kinase [EC:2.7.2.15]	1.00	0.95	1.07	8.8E-01	9.3E-01
K15524	mannosylglycerate hydrolase [EC:3.2.1.170]	1.00	0.94	1.06	8.8E-01	9.3E-01
K00625	phosphate acetyltransferase [EC:2.3.1.8]	1.00	0.95	1.07	8.8E-01	9.3E-01
K00748	lipid-A-disaccharide synthase [EC:2.4.1.182]	1.00	0.94	1.06	8.8E-01	9.3E-01
K00001	alcohol dehydrogenase [EC:1.1.1.1]	1.00	0.95	1.07	8.8E-01	9.3E-01
K08296	phosphohistidine phosphatase [EC:3.1.3.-]	1.00	0.95	1.07	8.8E-01	9.3E-01
K13292	phosphatidylglycerol---prolipoprotein diacylglyceryl transferase [EC:2.5.1.145]	1.00	0.95	1.07	8.8E-01	9.3E-01
K00382	dihydrolipoamide dehydrogenase [EC:1.8.1.4]	1.00	0.95	1.07	8.8E-01	9.3E-01
K00999	CDP-diacylglycerol--inositol 3-phosphatidyltransferase [EC:2.7.8.11]	1.00	0.94	1.06	8.8E-01	9.3E-01
K09568	FK506-binding protein 1 [EC:5.2.1.8]	1.00	0.94	1.06	8.8E-01	9.3E-01
K02041	phosphonate transport system ATP-binding protein [EC:7.3.2.2]	1.00	0.94	1.06	8.8E-01	9.3E-01
K10213	ribosylpyrimidine nucleosidase [EC:3.2.2.8]	1.00	0.94	1.06	8.8E-01	9.3E-01
K01008	selenide, water dikinase [EC:2.7.9.3]	1.00	0.94	1.06	8.8E-01	9.3E-01

Predictor	Description	HR	2.5% CI	97.5% CI	P	FDR-adjusted P
K00156	pyruvate dehydrogenase (quinone) [EC:1.2.5.1]	1.00	0.94	1.06	8.8E-01	9.3E-01
K05286	GPI mannosyltransferase 3 [EC:2.4.1.-]	1.00	0.94	1.06	8.8E-01	9.3E-01
K15467	27-O-demethylrifamycin SV methyltransferase [EC:2.1.1.315]	1.00	0.95	1.07	8.8E-01	9.3E-01
K01034	acetate CoA/acetoacetate CoA-transferase alpha subunit [EC:2.8.3.8 2.8.3.9]	1.00	0.95	1.07	8.8E-01	9.3E-01
K10742	DNA replication ATP-dependent helicase Dna2 [EC:3.6.4.12]	1.00	0.95	1.07	8.9E-01	9.3E-01
K00344	NADPH:quinone reductase [EC:1.6.5.5]	1.00	0.94	1.06	8.9E-01	9.3E-01
K02619	4-amino-4-deoxychorismate lyase [EC:4.1.3.38]	1.00	0.95	1.07	8.9E-01	9.4E-01
K00639	glycine C-acetyltransferase [EC:2.3.1.29]	1.00	0.94	1.06	8.9E-01	9.4E-01
K01814	phosphoribosylformimino-5-aminoimidazole carboxamide ribotide isomerase [EC:5.3.1.16]	1.00	0.94	1.07	8.9E-01	9.4E-01
K00979	3-deoxy-manno-octulosonate cytidylyltransferase (CMP-KDO synthetase) [EC:2.7.7.38]	1.00	0.94	1.06	8.9E-01	9.4E-01
K01964	acetyl-CoA/propionyl-CoA carboxylase [EC:6.4.1.2 6.4.1.3]	1.00	0.95	1.07	8.9E-01	9.4E-01
K04087	modulator of FtsH protease HflC	1.00	0.95	1.07	8.9E-01	9.4E-01
K01479	formiminoglutamase [EC:3.5.3.8]	1.00	0.95	1.07	8.9E-01	9.4E-01
K00299	FMN reductase [EC:1.5.1.38]	1.00	0.95	1.07	8.9E-01	9.4E-01
K16301	deferrochelatase/peroxidase EfeB [EC:1.11.1.-]	1.00	0.95	1.07	8.9E-01	9.4E-01
K10551	D-allose transport system ATP-binding protein [EC:7.5.2.8]	1.00	0.94	1.06	8.9E-01	9.4E-01
K00949	thiamine pyrophosphokinase [EC:2.7.6.2]	1.00	0.95	1.06	8.9E-01	9.4E-01
K00595	precorrin-6B C5,15-methyltransferase / cobalt-precorrin-6B C5,C15-methyltransferase [EC:2.1.1.132 2.1.1.289 2.1.1.196]	1.00	0.95	1.07	9.0E-01	9.4E-01
K05972	acetylxylan esterase [EC:3.1.1.72]	1.00	0.95	1.06	9.0E-01	9.4E-01
K15972	tetracenomycin A2 monooxygenase-dioxygenase [EC:1.14.13.200]	1.00	0.95	1.06	9.0E-01	9.4E-01
K00835	valine--pyruvate aminotransferase [EC:2.6.1.66]	1.00	0.95	1.07	9.0E-01	9.4E-01
K01012	biotin synthase [EC:2.8.1.6]	1.00	0.94	1.07	9.0E-01	9.4E-01
K03271	D-sedoheptulose 7-phosphate isomerase [EC:5.3.1.28]	1.00	0.94	1.06	9.0E-01	9.4E-01
K03216	tRNA (cytidine/uridine-2'-O-)-methyltransferase [EC:2.1.1.207]	1.00	0.95	1.07	9.0E-01	9.5E-01
K14982	two-component system, OmpR family, sensor histidine kinase CiaH [EC:2.7.13.3]	1.00	0.94	1.06	9.0E-01	9.5E-01
K05977	2'-hydroxybiphenyl-2-sulfinate desulfinase [EC:3.13.1.3]	1.00	0.95	1.06	9.0E-01	9.5E-01
K13529	AraC family transcriptional regulator, regulatory protein of adaptative response / DNA-3-methyladenine glycosylase II [EC:3.2.2.21]	1.00	0.95	1.07	9.0E-01	9.5E-01
K07677	two-component system, NarL family, capsular synthesis sensor histidine kinase RcsC [EC:2.7.13.3]	1.00	0.94	1.07	9.1E-01	9.5E-01
K13308	dTDP-4-amino-4,6-dideoxy-D-glucose transaminase [EC:2.6.1.33]	1.00	0.94	1.06	9.1E-01	9.5E-01
K08297	crotonobetainyl-CoA dehydrogenase [EC:1.3.8.13]	1.00	0.94	1.06	9.1E-01	9.5E-01
K07636	two-component system, OmpR family, phosphate regulon sensor histidine kinase PhoR [EC:2.7.13.3]	1.00	0.94	1.06	9.1E-01	9.5E-01
K01847	methylmalonyl-CoA mutase [EC:5.4.99.2]	1.00	0.94	1.06	9.1E-01	9.5E-01
K10677	inulin fructotransferase (DFA-I-forming) [EC:4.2.2.17]	1.00	0.94	1.06	9.1E-01	9.5E-01
K00627	pyruvate dehydrogenase E2 component (dihydrolipoamide acetyltransferase) [EC:2.3.1.12]	1.00	0.94	1.06	9.1E-01	9.5E-01
K01151	deoxyribonuclease IV [EC:3.1.21.2]	1.00	0.94	1.07	9.1E-01	9.5E-01
K00227	Delta7-sterol 5-desaturase [EC:1.14.19.20]	1.00	0.95	1.06	9.1E-01	9.5E-01
K00872	homoserine kinase [EC:2.7.1.39]	1.00	0.94	1.06	9.2E-01	9.5E-01
K02496	uroporphyrin-III C-methyltransferase [EC:2.1.1.107]	1.00	0.94	1.07	9.2E-01	9.6E-01
K09019	3-hydroxypropanoate dehydrogenase [EC:1.1.1.-]	1.00	0.94	1.07	9.2E-01	9.6E-01
K00564	16S rRNA (guanine1207-N2)-methyltransferase [EC:2.1.1.172]	1.00	0.94	1.06	9.2E-01	9.6E-01
K13497	anthranilate synthase/phosphoribosyltransferase [EC:4.1.3.27 2.4.2.18]	1.00	0.94	1.07	9.2E-01	9.6E-01
K00381	sulfite reductase (NADPH) hemoprotein beta-component [EC:1.8.1.2]	1.00	0.94	1.06	9.2E-01	9.6E-01
K06223	DNA adenine methylase [EC:2.1.1.72]	1.00	0.95	1.06	9.2E-01	9.6E-01
K02755	beta-glucoside PTS system EIIA component [EC:2.7.1.-]	1.00	0.94	1.06	9.2E-01	9.6E-01

Predictor	Description	HR	2.5% CI	97.5% CI	P	FDR-adjusted P
K01947	biotin---[acetyl-CoA-carboxylase] ligase / type III pantothenate kinase [EC:6.3.4.15 2.7.1.33]	1.00	0.95	1.06	9.2E-01	9.6E-01
K01087	trehalose 6-phosphate phosphatase [EC:3.1.3.12]	1.00	0.94	1.06	9.2E-01	9.6E-01
K08316	16S rRNA (guanine966-N2)-methyltransferase [EC:2.1.1.171]	1.00	0.94	1.06	9.2E-01	9.6E-01
K03827	putative acetyltransferase [EC:2.3.1.-]	1.00	0.94	1.06	9.2E-01	9.6E-01
K01624	fructose-bisphosphate aldolase, class II [EC:4.1.2.13]	1.00	0.95	1.06	9.3E-01	9.6E-01
K03579	ATP-dependent helicase HrpB [EC:3.6.4.13]	1.00	0.94	1.06	9.3E-01	9.6E-01
K01251	adenosylhomocysteinase [EC:3.3.1.1]	1.00	0.94	1.06	9.3E-01	9.6E-01
K13246	c-di-GMP phosphodiesterase [EC:3.1.4.52]	1.00	0.95	1.06	9.3E-01	9.6E-01
K08680	2-succinyl-6-hydroxy-2,4-cyclohexadiene-1-carboxylate synthase [EC:4.2.99.20]	1.00	0.94	1.07	9.3E-01	9.6E-01
K12267	peptide methionine sulfoxide reductase msrA/msrB [EC:1.8.4.11 1.8.4.12]	1.00	0.94	1.06	9.3E-01	9.6E-01
K01873	valyl-tRNA synthetase [EC:6.1.1.9]	1.00	0.95	1.06	9.3E-01	9.6E-01
K00818	acetylornithine aminotransferase [EC:2.6.1.11]	1.00	0.94	1.06	9.3E-01	9.6E-01
K01652	acetolactate synthase I/II/III large subunit [EC:2.2.1.6]	1.00	0.94	1.06	9.3E-01	9.7E-01
K10535	hydroxylamine dehydrogenase [EC:1.7.2.6]	1.00	0.94	1.06	9.3E-01	9.7E-01
K01011	thiosulfate/3-mercaptopyruvate sulfurtransferase [EC:2.8.1.1 2.8.1.2]	1.00	0.94	1.06	9.3E-01	9.7E-01
K05599	anthranilate 1,2-dioxygenase (deaminating, decarboxylating) large subunit [EC:1.14.12.1]	1.00	0.94	1.06	9.3E-01	9.7E-01
K05600	anthranilate 1,2-dioxygenase (deaminating, decarboxylating) small subunit [EC:1.14.12.1]	1.00	0.94	1.06	9.3E-01	9.7E-01
K00130	betaine-aldehyde dehydrogenase [EC:1.2.1.8]	1.00	0.94	1.06	9.4E-01	9.7E-01
K11945	extradiol dioxygenase [EC:1.13.11.-]	1.00	0.94	1.06	9.4E-01	9.7E-01
K00997	holo-[acyl-carrier protein] synthase [EC:2.7.8.7]	1.00	0.94	1.06	9.4E-01	9.7E-01
K00697	trehalose 6-phosphate synthase [EC:2.4.1.15 2.4.1.347]	1.00	0.94	1.06	9.4E-01	9.7E-01
K07406	alpha-galactosidase [EC:3.2.1.22]	1.00	0.94	1.06	9.4E-01	9.7E-01
K12251	N-carbamoylputrescine amidase [EC:3.5.1.53]	1.00	0.94	1.06	9.4E-01	9.7E-01
K06173	tRNA pseudouridine38-40 synthase [EC:5.4.99.12]	1.00	0.94	1.06	9.4E-01	9.7E-01
K00244	fumarate reductase flavoprotein subunit [EC:1.3.5.4]	1.00	0.94	1.06	9.4E-01	9.7E-01
K08310	dihydroneopterin triphosphate diphosphatase [EC:3.6.1.67]	1.00	0.94	1.06	9.4E-01	9.7E-01
K02371	enoyl-[acyl-carrier protein] reductase II [EC:1.3.1.9]	1.00	0.94	1.06	9.4E-01	9.7E-01
K06181	23S rRNA pseudouridine2457 synthase [EC:5.4.99.20]	1.00	0.94	1.06	9.4E-01	9.7E-01
K00163	pyruvate dehydrogenase E1 component [EC:1.2.4.1]	1.00	0.94	1.06	9.5E-01	9.7E-01
K01615	glutaconyl-CoA decarboxylase subunit alpha [EC:7.2.4.5]	1.00	0.94	1.06	9.5E-01	9.7E-01
K03500	16S rRNA (cytosine967-C5)-methyltransferase [EC:2.1.1.176]	1.00	0.94	1.06	9.5E-01	9.7E-01
K01644	citrate lyase subunit beta / citryl-CoA lyase [EC:4.1.3.34]	1.00	0.94	1.06	9.5E-01	9.7E-01
K00800	3-phosphoshikimate 1-carboxyvinyltransferase [EC:2.5.1.19]	1.00	0.94	1.06	9.5E-01	9.8E-01
K07568	S-adenosylmethionine:tRNA ribosyltransferase-isomerase [EC:2.4.99.17]	1.00	0.94	1.06	9.5E-01	9.8E-01
K03601	exodeoxyribonuclease VII large subunit [EC:3.1.11.6]	1.00	0.95	1.06	9.5E-01	9.8E-01
K08722	5'-deoxynucleotidase [EC:3.1.3.89]	1.00	0.94	1.06	9.5E-01	9.8E-01
K16363	UDP-3-O-[3-hydroxymyristoyl] N-acetylglucosamine deacetylase / 3-hydroxyacyl-[acyl-carrier-protein] dehydratase [EC:3.5.1.108 4.2.1.59]	1.00	0.94	1.06	9.5E-01	9.8E-01
K10679	nitroreductase / dihydropteridine reductase [EC:1.-.-.- 1.5.1.34]	1.00	0.94	1.06	9.5E-01	9.8E-01
K03340	diaminopimelate dehydrogenase [EC:1.4.1.16]	1.00	0.94	1.06	9.5E-01	9.8E-01
K00060	threonine 3-dehydrogenase [EC:1.1.1.103]	1.00	0.94	1.06	9.5E-01	9.8E-01
K00573	protein-L-isoaspartate(D-aspartate) O-methyltransferase [EC:2.1.1.77]	1.00	0.94	1.06	9.5E-01	9.8E-01
K03365	cytosine/creatinine deaminase [EC:3.5.4.1 3.5.4.21]	1.00	0.94	1.06	9.5E-01	9.8E-01
K01414	oligopeptidase A [EC:3.4.24.70]	1.00	0.94	1.06	9.6E-01	9.8E-01
K00087	xanthine dehydrogenase molybdenum-binding subunit [EC:1.17.1.4]	1.00	0.94	1.06	9.6E-01	9.8E-01

Predictor	Description	HR	2.5% CI	97.5% CI	P	FDR-adjusted P
K02301	NA	1.00	0.94	1.06	9.6E-01	9.8E-01
K02431	L-fucose mutarotase [EC:5.1.3.29]	1.00	0.94	1.06	9.6E-01	9.8E-01
K09001	anhydro-N-acetylmuramic acid kinase [EC:2.7.1.170]	1.00	0.94	1.06	9.6E-01	9.8E-01
K03773	FKBP-type peptidyl-prolyl cis-trans isomerase FklB [EC:5.2.1.8]	1.00	0.94	1.06	9.6E-01	9.8E-01
K01755	argininosuccinate lyase [EC:4.3.2.1]	1.00	0.94	1.06	9.6E-01	9.8E-01
K00265	glutamate synthase (NADPH) large chain [EC:1.4.1.13]	1.00	0.94	1.06	9.6E-01	9.8E-01
K13059	N-acetylhexosamine 1-kinase [EC:2.7.1.162]	1.00	0.94	1.06	9.6E-01	9.8E-01
K12373	hexosaminidase [EC:3.2.1.52]	1.00	0.94	1.06	9.6E-01	9.8E-01
K01703	3-isopropylmalate/(R)-2-methylmalate dehydratase large subunit [EC:4.2.1.33 4.2.1.35]	1.00	0.94	1.06	9.6E-01	9.8E-01
K00280	lysyl oxidase-like protein 2/3/4 [EC:1.4.3.-]	1.00	0.94	1.06	9.6E-01	9.8E-01
K01669	deoxyribodipyrimidine photo-lyase [EC:4.1.99.3]	1.00	0.94	1.06	9.6E-01	9.8E-01
K03722	ATP-dependent DNA helicase DinG [EC:3.6.4.12]	1.00	0.94	1.06	9.6E-01	9.8E-01
K00694	cellulose synthase (UDP-forming) [EC:2.4.1.12]	1.00	0.94	1.06	9.6E-01	9.8E-01
K01839	phosphopentomutase [EC:5.4.2.7]	1.00	0.94	1.06	9.6E-01	9.8E-01
K00336	NADH-quinone oxidoreductase subunit G [EC:7.1.1.2]	1.00	0.94	1.06	9.6E-01	9.8E-01
K04844	hypothetical glycosyl hydrolase [EC:3.2.1.-]	1.00	0.94	1.06	9.6E-01	9.8E-01
K07314	serine/threonine protein phosphatase 2 [EC:3.1.3.16]	1.00	0.94	1.06	9.6E-01	9.8E-01
K03527	4-hydroxy-3-methylbut-2-en-1-yl diphosphate reductase [EC:1.17.7.4]	1.00	0.94	1.06	9.6E-01	9.8E-01
K05522	endonuclease VIII [EC:3.2.2.- 4.2.99.18]	1.00	0.94	1.06	9.6E-01	9.8E-01
K14728	phthiodiolone/phenolphthiodiolone dimycocerosates ketoreductase [EC:1.2.-.-]	1.00	0.94	1.06	9.6E-01	9.8E-01
K00971	mannose-1-phosphate guanylyltransferase [EC:2.7.7.13]	1.00	0.94	1.06	9.6E-01	9.8E-01
K00853	L-ribulokinase [EC:2.7.1.16]	1.00	0.94	1.06	9.6E-01	9.8E-01
K02486	two-component system, sensor kinase [EC:2.7.13.3]	1.00	0.94	1.06	9.6E-01	9.8E-01
K00547	homocysteine S-methyltransferase [EC:2.1.1.10]	1.00	0.94	1.06	9.7E-01	9.8E-01
K00556	tRNA (guanosine-2'-O-)-methyltransferase [EC:2.1.1.34]	1.00	0.94	1.06	9.7E-01	9.8E-01
K14652	3,4-dihydroxy 2-butanone 4-phosphate synthase / GTP cyclohydrolase II [EC:4.1.99.12 3.5.4.25]	1.00	0.94	1.06	9.7E-01	9.8E-01
K02464	general secretion pathway protein O [EC:3.4.23.43 2.1.1.-]	1.00	0.94	1.06	9.7E-01	9.8E-01
K00380	sulfite reductase (NADPH) flavoprotein alpha-component [EC:1.8.1.2]	1.00	0.94	1.06	9.7E-01	9.8E-01
K01577	oxalyl-CoA decarboxylase [EC:4.1.1.8]	1.00	0.94	1.06	9.7E-01	9.8E-01
K00683	glutaminyl-peptide cyclotransferase [EC:2.3.2.5]	1.00	0.94	1.06	9.7E-01	9.8E-01
K01066	acetyl esterase [EC:3.1.1.-]	1.00	0.94	1.06	9.7E-01	9.8E-01
K10210	diapolycopene oxygenase [EC:1.14.99.44]	1.00	0.94	1.06	9.7E-01	9.8E-01
K03787	5'-nucleotidase [EC:3.1.3.5]	1.00	0.94	1.06	9.7E-01	9.8E-01
K00363	nitrite reductase (NADH) small subunit [EC:1.7.1.15]	1.00	0.94	1.06	9.7E-01	9.8E-01
K13664	acyltransferase [EC:2.3.1.-]	1.00	0.94	1.06	9.7E-01	9.8E-01
K13658	beta-1,4-mannosyltransferase [EC:2.4.1.251]	1.00	0.94	1.06	9.7E-01	9.8E-01
K05986	nuclease S1 [EC:3.1.30.1]	1.00	0.94	1.06	9.7E-01	9.8E-01
K14159	ribonuclease HI / DNA polymerase III subunit epsilon [EC:3.1.26.4 2.7.7.7]	1.00	0.94	1.06	9.7E-01	9.8E-01
K00858	NAD+ kinase [EC:2.7.1.23]	1.00	0.94	1.06	9.8E-01	9.9E-01
K07141	molybdenum cofactor cytidylyltransferase [EC:2.7.7.76]	1.00	0.94	1.06	9.8E-01	9.9E-01
K11944	PAH dioxygenase small subunit [EC:1.13.11.-]	1.00	0.94	1.06	9.8E-01	9.9E-01
K07389	cytolysin-activating lysine-acyltransferase [EC:2.3.1.-]	1.00	0.94	1.06	9.8E-01	9.9E-01
K00121	S-(hydroxymethyl)glutathione dehydrogenase / alcohol dehydrogenase [EC:1.1.1.284 1.1.1.1]	1.00	0.94	1.06	9.8E-01	9.9E-01
K03179	4-hydroxybenzoate polyprenyltransferase [EC:2.5.1.39]	1.00	0.94	1.06	9.8E-01	9.9E-01

Predictor	Description	HR	2.5% CI	97.5% CI	P	FDR-adjusted P
K01206	alpha-L-fucosidase [EC:3.2.1.51]	1.00	0.94	1.06	9.8E-01	9.9E-01
K11948	1-hydroxy-2-naphthoate dioxygenase [EC:1.13.11.38]	1.00	0.94	1.06	9.8E-01	9.9E-01
K11947	aldehyde dehydrogenase [EC:1.2.1.-]	1.00	0.94	1.06	9.8E-01	9.9E-01
K11946	hydratase-aldolase [EC:4.1.2.-]	1.00	0.94	1.06	9.8E-01	9.9E-01
K11943	PAH dioxygenase large subunit [EC:1.13.11.-]	1.00	0.94	1.06	9.8E-01	9.9E-01
K00630	glycerol-3-phosphate O-acyltransferase [EC:2.3.1.15]	1.00	0.94	1.06	9.8E-01	9.9E-01
K00789	S-adenosylmethionine synthetase [EC:2.5.1.6]	1.00	0.94	1.06	9.8E-01	9.9E-01
K03940	NADH dehydrogenase (ubiquinone) Fe-S protein 7 [EC:7.1.1.2]	0.76	0.00	84970754435	9.8E-01	9.9E-01
K01191	alpha-mannosidase [EC:3.2.1.24]	1.00	0.94	1.06	9.8E-01	9.9E-01
K01664	para-aminobenzoate synthetase component II [EC:2.6.1.85]	1.00	0.94	1.06	9.9E-01	9.9E-01
K02806	nitrogen PTS system EIIA component [EC:2.7.1.-]	1.00	0.94	1.06	9.9E-01	9.9E-01
K12993	O-antigen biosynthesis alpha-1,2-mannosyltransferase [EC:2.4.1.371 2.4.1.-]	1.00	0.94	1.06	9.9E-01	9.9E-01
K09251	putrescine aminotransferase [EC:2.6.1.82]	1.00	0.94	1.06	9.9E-01	9.9E-01
K07305	peptide-methionine (R)-S-oxide reductase [EC:1.8.4.12]	1.00	0.94	1.06	9.9E-01	1.0E+00
K00005	glycerol dehydrogenase [EC:1.1.1.6]	1.00	0.94	1.06	9.9E-01	1.0E+00
K04088	modulator of FtsH protease HflK	1.00	0.94	1.06	9.9E-01	1.0E+00
K00867	type I pantothenate kinase [EC:2.7.1.33]	1.00	0.94	1.06	9.9E-01	1.0E+00
K00946	thiamine-monophosphate kinase [EC:2.7.4.16]	1.00	0.94	1.06	9.9E-01	1.0E+00
K07318	adenine-specific DNA-methyltransferase [EC:2.1.1.72]	1.00	0.94	1.06	9.9E-01	1.0E+00
K01304	pyroglutamyl-peptidase [EC:3.4.19.3]	1.00	0.94	1.06	9.9E-01	1.0E+00
K00989	ribonuclease PH [EC:2.7.7.56]	1.00	0.94	1.06	1.0E+00	1.0E+00
K13479	xanthine dehydrogenase FAD-binding subunit [EC:1.17.1.4]	1.00	0.94	1.06	1.0E+00	1.0E+00
K00882	1-phosphofructokinase [EC:2.7.1.56]	1.00	0.94	1.06	1.0E+00	1.0E+00
K15019	3-hydroxypropionyl-coenzyme A dehydratase [EC:4.2.1.116]	1.00	0.94	1.06	1.0E+00	1.0E+00
K05592	ATP-dependent RNA helicase DeaD [EC:3.6.4.13]	1.00	0.94	1.06	1.0E+00	1.0E+00
K07749	formyl-CoA transferase [EC:2.8.3.16]	1.00	0.94	1.06	1.0E+00	1.0E+00
K01749	hydroxymethylbilane synthase [EC:2.5.1.61]	1.00	0.94	1.06	1.0E+00	1.0E+00
K00981	phosphatidate cytidylyltransferase [EC:2.7.7.41]	1.00	0.94	1.06	1.0E+00	1.0E+00
K01662	1-deoxy-D-xylulose-5-phosphate synthase [EC:2.2.1.7]	1.00	0.94	1.06	1.0E+00	1.0E+00

Supplementary Table 17. Top 25 negative associations between functional groups and risk of incident fractures

Predictor	Description	HR	2.5% CI	97.5% CI	P	FDR-adjusted P	Associated with fractures (p< 0.05)	Implicated in amino acid metabolism
K05587	bidirectional [NiFe] hydrogenase diaphorase subunit [EC:7.1.1.2]	0.88	0.83	0.93	5.5E-06	8.6E-03	yes	
K15518	deoxyguanosine kinase [EC:2.7.1.113]	0.88	0.84	0.93	8.9E-06	8.6E-03	yes	
K13853	3-deoxy-7-phosphoheptulonate synthase / chorismate mutase [EC:2.5.1.54 5.4.99.5]	0.88	0.83	0.93	1.2E-05	8.6E-03	yes	yes
K16242	phenol/toluene 2-monooxygenase (NADH) P3/A3 [EC:1.14.13.244 1.14.13.243]	0.89	0.84	0.94	1.9E-05	8.6E-03	yes	
K02829	cytochrome aa3-600 menaquinol oxidase subunit IV [EC:7.1.1.5]	0.89	0.84	0.94	2.5E-05	8.6E-03	yes	
K01003	oxaloacetate decarboxylase [EC:4.1.1.112]	0.89	0.84	0.94	2.5E-05	8.6E-03	yes	
K15629	fatty-acid peroxygenase [EC:1.11.2.4]	0.88	0.83	0.94	3.1E-05	8.6E-03	yes	
K00365	urate oxidase [EC:1.7.3.3]	0.89	0.84	0.94	3.2E-05	8.6E-03	yes	
K01431	beta-ureidopropionase [EC:3.5.1.6]	0.89	0.84	0.94	3.7E-05	8.6E-03	yes	
K05521	ADP-ribosyl-[dinitrogen reductase] hydrolase [EC:3.2.2.24]	0.88	0.83	0.94	3.7E-05	8.6E-03	yes	
K11693	peptidoglycan pentaglycine glycine transferase (the first glycine) [EC:2.3.2.16]	0.89	0.84	0.94	3.9E-05	8.6E-03	yes	
K07130	arylformamidase [EC:3.5.1.9]	0.88	0.83	0.94	4.1E-05	8.6E-03	yes	yes
K00643	5-aminolevulinate synthase [EC:2.3.1.37]	0.89	0.84	0.94	4.1E-05	8.6E-03	yes	yes
K01166	ribonuclease T2 [EC:4.6.1.19]	0.89	0.84	0.94	4.5E-05	8.6E-03	yes	
K13832	3-dehydroquinate dehydratase / shikimate dehydrogenase [EC:4.2.1.10 1.1.1.25]	0.88	0.83	0.94	4.7E-05	8.6E-03	yes	yes
K15761	toluene monooxygenase system protein B [EC:1.14.13.236 1.14.13.-]	0.89	0.84	0.94	5.3E-05	8.6E-03	yes	
K05586	bidirectional [NiFe] hydrogenase diaphorase subunit [EC:7.1.1.2]	0.89	0.84	0.94	5.5E-05	8.6E-03	yes	
K05588	bidirectional [NiFe] hydrogenase diaphorase subunit [EC:7.1.1.2]	0.89	0.84	0.94	5.5E-05	8.6E-03	yes	
K00831	phosphoserine aminotransferase [EC:2.6.1.52]	0.90	0.86	0.95	5.5E-05	8.6E-03	yes	yes
K14048	urease subunit gamma/beta [EC:3.5.1.5]	0.89	0.84	0.94	5.8E-05	8.6E-03	yes	yes
K00588	caffeoyl-CoA O-methyltransferase [EC:2.1.1.104]	0.89	0.84	0.94	6.0E-05	8.6E-03	yes	
K15632	23S rRNA (adenine-C8)-methyltransferase [EC:2.1.1.224]	0.89	0.84	0.94	7.3E-05	9.3E-03	yes	
K00491	nitric-oxide synthase, bacterial [EC:1.14.14.47]	0.89	0.84	0.94	8.0E-05	9.3E-03	yes	yes
K15534	beta-D-galactosyl-(1->4)-L-rhamnose phosphorylase [EC:2.4.1.247]	0.89	0.84	0.94	8.0E-05	9.3E-03	yes	
K01442	choloylglycine hydrolase [EC:3.5.1.24]	0.91	0.87	0.95	8.3E-05	9.3E-03	yes	

Cox regressions with any fractures as outcome, functional groups as exposure, and adjusted using the main model (including age, gender, medication, antibiotics, and previous fractures as covariates). n = 7043 (1092 cases). HR, hazard ratio; CI, confidence interval, FDR, false discovery rate

Supplementary Table 18. Positive associations between functional groups and risk of incident fractures

Predictor	Description	HR	2.5% CI	97.5% CI	P	FDR-adjusted P	Associated with fractures (P< 0.05)	Implicated in LPS biosynthesis
K03280	UDP-N-acetylglucosamine:(glucosyl)LPS alpha-1,2-N-acetylglucosaminyltransferase [EC:2.4.1.56]	1.12	1.06	1.19	1.1E-04	9.3E-03	yes	yes
K01518	bis(5'-nucleosidyl)-tetraphosphatase [EC:3.6.1.17]	1.10	1.04	1.15	4.0E-04	1.2E-02	yes	
K00326	cytochrome-b5 reductase [EC:1.6.2.2]	1.10	1.04	1.15	4.5E-04	1.2E-02	yes	
K05929	phosphoethanolamine N-methyltransferase [EC:2.1.1.103]	1.10	1.04	1.15	4.5E-04	1.2E-02	yes	
K01127	glycosylphosphatidylinositol phospholipase D [EC:3.1.4.50]	1.09	1.04	1.15	5.0E-04	1.2E-02	yes	
K06970	23S rRNA (adenine1618-N6)-methyltransferase [EC:2.1.1.181]	1.09	1.03	1.16	4.8E-03	2.4E-02	yes	
K00425	cytochrome bd ubiquinol oxidase subunit I [EC:7.1.1.7]	1.10	1.03	1.18	6.8E-03	3.0E-02	yes	
K00633	galactoside O-acetyltransferase [EC:2.3.1.18]	1.08	1.02	1.15	7.6E-03	3.2E-02	yes	
K00428	cytochrome c peroxidase [EC:1.11.1.5]	1.08	1.02	1.15	9.2E-03	3.8E-02	yes	
K00426	cytochrome bd ubiquinol oxidase subunit II [EC:7.1.1.7]	1.10	1.02	1.18	1.0E-02	4.1E-02	yes	

Cox regressions with incident fractures as outcome, functional groups as exposure, and adjusted using the main model (including age, gender, medication, antibiotics, and previous fractures as covariates). n = 7043 (1092 cases). HR, hazard ratio; CI, confidence interval; FDR, false discovery rate

Supplementary Table 19. Correlations between the relative abundance of Proteobacteria and functional groups

Pearson's correlations between the relative abundance of Proteobacteria and functional groups. n = 7043 (1092 cases). FDR, false discovery rate

Predictor	Description	Pearson's r	P	FDR-adjusted P	Spearman's rho	Associated with fractures (P<0.05)
K16149	1,4-alpha-glucan branching enzyme [EC:2.4.1.18]	-0.46	<5.9E-323	<1.8E-320	-0.47	yes
K06208	chorismate mutase [EC:5.4.99.5]	-0.45	<5.9E-323	<1.8E-320	-0.46	yes
K01869	leucyl-tRNA synthetase [EC:6.1.1.4]	-0.45	<5.9E-323	<1.8E-320	-0.41	
K13282	cyanophycinase [EC:3.4.15.6]	-0.45	<5.9E-323	<1.8E-320	-0.46	yes
K07651	two-component system, OmpR family, sensor histidine kinase ResE [EC:2.7.13.3]	-0.44	<5.9E-323	<1.8E-320	-0.45	yes
K00015	glyoxylate reductase [EC:1.1.1.26]	-0.43	<5.9E-323	<1.8E-320	-0.44	yes
K01474	N-methylhydantoinase B [EC:3.5.2.14]	-0.43	<5.9E-323	<1.8E-320	-0.44	yes
K09903	uridylate kinase [EC:2.7.4.22]	-0.43	<5.9E-323	<1.8E-320	-0.41	
K10843	DNA excision repair protein ERCC-3 [EC:3.6.4.12]	-0.43	<5.9E-323	<1.8E-320	-0.43	yes
K02636	cytochrome b6-f complex iron-sulfur subunit [EC:7.1.1.6]	-0.43	5.9E-323	1.8E-320	-0.44	yes
K11783	futalosine hydrolase [EC:3.2.2.26]	-0.43	4.3E-321	1.2E-318	-0.44	yes
K00762	orotate phosphoribosyltransferase [EC:2.4.2.10]	-0.43	1.4E-319	3.5E-317	-0.42	
K03519	aerobic carbon-monoxide dehydrogenase medium subunit [EC:1.2.5.3]	-0.43	1.6E-319	3.7E-317	-0.45	yes
K00303	sarcosine oxidase, subunit beta [EC:1.5.3.1]	-0.43	1.7E-319	3.7E-317	-0.45	yes
K02277	cytochrome c oxidase subunit IV [EC:7.1.1.9]	-0.43	6.1E-319	1.2E-316	-0.44	yes
K01556	kynureninase [EC:3.7.1.3]	-0.43	2.3E-318	4.3E-316	-0.44	yes
K01883	cysteinyl-tRNA synthetase [EC:6.1.1.16]	-0.43	2.5E-318	4.4E-316	-0.41	
K01866	tyrosyl-tRNA synthetase [EC:6.1.1.1]	-0.43	3.9E-318	6.5E-316	-0.41	
K07130	arylformamidase [EC:3.5.1.9]	-0.43	1.2E-317	1.9E-315	-0.44	yes
K13598	two-component system, NtrC family, nitrogen regulation sensor histidine kinase NtrY [EC:2.7.13.3]	-0.43	5.8E-316	8.7E-314	-0.44	yes
K01892	histidyl-tRNA synthetase [EC:6.1.1.21]	-0.43	2.2E-314	3.2E-312	-0.41	
K03392	aminocarboxymuconate-semialdehyde decarboxylase [EC:4.1.1.45]	-0.43	4.3E-314	5.9E-312	-0.43	yes
K01887	arginyl-tRNA synthetase [EC:6.1.1.19]	-0.43	5.3E-314	7.0E-312	-0.41	
K00263	leucine dehydrogenase [EC:1.4.1.9]	-0.43	6.8E-314	8.5E-312	-0.44	yes
K08097	phosphosulfolactate synthase [EC:4.4.1.19]	-0.43	3.5E-313	4.2E-311	-0.44	yes
K00958	sulfate adenylyltransferase [EC:2.7.7.4]	-0.43	6.1E-313	7.1E-311	-0.44	yes
K15921	arabinoxylan arabinofuranohydrolase [EC:3.2.1.55]	-0.43	1.7E-312	1.9E-310	-0.44	yes
K15924	glucuronoarabinoxylan endo-1,4-beta-xylanase [EC:3.2.1.136]	-0.43	3.2E-312	3.4E-310	-0.44	yes
K11180	dissimilatory sulfite reductase alpha subunit [EC:1.8.99.5]	-0.43	3.8E-312	4.0E-310	-0.43	yes
K04038	light-independent protochlorophyllide reductase subunit N [EC:1.3.7.7]	-0.43	4.5E-312	4.5E-310	-0.43	yes
K07469	aldehyde oxidoreductase [EC:1.2.99.7]	-0.43	5.4E-312	5.3E-310	-0.44	yes
K00533	ferredoxin hydrogenase large subunit [EC:1.12.7.2]	-0.43	4.6E-311	4.3E-309	-0.44	yes
K01945	phosphoribosylamine---glycine ligase [EC:6.3.4.13]	-0.43	6.6E-311	6.0E-309	-0.41	
K15856	GDP-4-dehydro-6-deoxy-D-mannose reductase [EC:1.1.1.281]	-0.43	6.9E-311	6.1E-309	-0.44	yes
K12454	CDP-paratose 2-epimerase [EC:5.1.3.10]	-0.43	7.3E-311	6.3E-309	-0.43	yes
K07094	heptaprenylglyceryl phosphate synthase [EC:2.5.1.-]	-0.43	9.4E-310	7.9E-308	-0.43	yes
K04037	light-independent protochlorophyllide reductase subunit L [EC:1.3.7.7]	-0.43	3.4E-308	2.8E-306	-0.43	yes

Predictor	Description	Pearson's r	P	FDR-adjusted P	Spearman's rho	Associated with fractures (P<0.05)
K01880	glycyl-tRNA synthetase [EC:6.1.1.14]	-0.43	3.7E-308	2.9E-306	-0.40	yes
K04039	light-independent protochlorophyllide reductase subunit B [EC:1.3.7.7]	-0.43	8.7E-308	6.7E-306	-0.43	yes
K01803	triosephosphate isomerase (TIM) [EC:5.3.1.1]	-0.43	9.4E-308	7.1E-306	-0.41	
K01881	prolyl-tRNA synthetase [EC:6.1.1.15]	-0.43	1.5E-307	1.1E-305	-0.41	
K03428	magnesium-protoporphyrin O-methyltransferase [EC:2.1.1.11]	-0.43	2.8E-307	2.0E-305	-0.43	yes
K00365	urate oxidase [EC:1.7.3.3]	-0.43	2.8E-307	2.0E-305	-0.43	yes
K00619	amino-acid N-acetyltransferase [EC:2.3.1.1]	-0.42	2.9E-306	2.0E-304	-0.43	yes
K01956	carbamoyl-phosphate synthase small subunit [EC:6.3.5.5]	-0.42	2.1E-305	1.4E-303	-0.40	
K01431	beta-ureidopropionase [EC:3.5.1.6]	-0.42	4.9E-305	3.2E-303	-0.44	yes
K00392	sulfite reductase (ferredoxin) [EC:1.8.7.1]	-0.42	6.5E-305	4.2E-303	-0.44	yes
K01889	phenylalanyl-tRNA synthetase alpha chain [EC:6.1.1.20]	-0.42	7.4E-305	4.6E-303	-0.41	
K02501	imidazole glycerol-phosphate synthase subunit HisH [EC:4.3.2.10]	-0.42	7.4E-305	4.6E-303	-0.42	
K00452	3-hydroxyanthranilate 3,4-dioxygenase [EC:1.13.11.6]	-0.42	4.4E-304	2.7E-302	-0.43	yes
K01736	chorismate synthase [EC:4.2.3.5]	-0.42	4.7E-304	2.8E-302	-0.41	
K13853	3-deoxy-7-phosphoheptulonate synthase / chorismate mutase [EC:2.5.1.54 5.4.99.5]	-0.42	5.7E-303	3.3E-301	-0.44	yes
K00038	3alpha(or 20beta)-hydroxysteroid dehydrogenase [EC:1.1.1.53]	-0.42	7.3E-303	4.2E-301	-0.43	yes
K02500	imidazole glycerol-phosphate synthase subunit HisF [EC:4.3.2.10]	-0.42	1.1E-302	6.1E-301	-0.40	
K11333	3,8-divinyl chlorophyllide a/chlorophyllide a reductase subunit X [EC:1.3.7.14 1.3.7.15]	-0.42	4.1E-302	2.2E-300	-0.42	yes
K14446	crotonyl-CoA carboxylase/reductase [EC:1.3.1.85]	-0.42	6.2E-302	3.3E-300	-0.43	yes
K02470	DNA gyrase subunit B [EC:5.6.2.2]	-0.42	8.0E-302	4.2E-300	-0.42	
K11614	two-component system, CitB family, sensor histidine kinase MalK [EC:2.7.13.3]	-0.42	1.0E-301	5.2E-300	-0.42	yes
K01473	N-methylhydantoinase A [EC:3.5.2.14]	-0.42	1.8E-301	9.2E-300	-0.44	yes
K00451	homogentisate 1,2-dioxygenase [EC:1.13.11.5]	-0.42	3.6E-301	1.8E-299	-0.43	yes
K01939	adenylosuccinate synthase [EC:6.3.4.4]	-0.42	5.2E-301	2.6E-299	-0.40	
K16011	mannose-1-phosphate guanylyltransferase / mannose-6-phosphate isomerase [EC:2.7.7.13 5.3.1.8]	-0.42	1.1E-300	5.4E-299	-0.43	yes
K00302	sarcosine oxidase, subunit alpha [EC:1.5.3.1]	-0.42	1.2E-300	5.7E-299	-0.43	yes
K01768	adenylate cyclase [EC:4.6.1.1]	-0.42	4.1E-300	1.9E-298	-0.43	yes
K01114	phospholipase C [EC:3.1.4.3]	-0.42	6.7E-300	3.1E-298	-0.44	yes
K01046	triacylglycerol lipase [EC:3.1.1.3]	-0.42	7.0E-300	3.2E-298	-0.43	yes
K02275	cytochrome c oxidase subunit II [EC:7.1.1.9]	-0.42	8.7E-300	3.9E-298	-0.43	yes
K13767	enoyl-CoA hydratase [EC:4.2.1.17]	-0.42	7.9E-299	3.5E-297	-0.43	yes
K01724	4a-hydroxytetrahydrobiopterin dehydratase [EC:4.2.1.96]	-0.42	8.3E-299	3.6E-297	-0.43	yes
K07516	3-hydroxyacyl-CoA dehydrogenase [EC:1.1.1.35]	-0.42	2.0E-298	8.6E-297	-0.43	yes
K03403	magnesium chelatase subunit H [EC:6.6.1.1]	-0.42	1.1E-297	4.7E-296	-0.42	yes
K11942	isobutyryl-CoA mutase [EC:5.4.99.13]	-0.42	1.5E-297	6.3E-296	-0.43	yes
K00059	3-oxoacyl-[acyl-carrier protein] reductase [EC:1.1.1.100]	-0.42	2.0E-297	8.3E-296	-0.28	yes
K00356	NA	-0.42	3.4E-297	1.4E-295	-0.43	yes
K13668	phosphatidyl-myo-inositol dimannoside synthase [EC:2.4.1.346]	-0.42	6.4E-297	2.6E-295	-0.43	yes
K00939	adenylate kinase [EC:2.7.4.3]	-0.42	8.1E-297	3.2E-295	-0.41	
K15067	2-aminomuconate deaminase [EC:3.5.99.5]	-0.42	1.9E-296	7.4E-295	-0.43	yes
K00856	adenosine kinase [EC:2.7.1.20]	-0.42	4.1E-296	1.6E-294	-0.43	yes
K01576	benzoylformate decarboxylase [EC:4.1.1.7]	-0.42	4.2E-296	1.6E-294	-0.44	yes

Predictor	Description	Pearson's r	P	FDR-adjusted P	Spearman's rho	Associated with fractures (P<0.05)
K02338	DNA polymerase III subunit beta [EC:2.7.7.7]	-0.42	1.4E-295	5.3E-294	-0.40	
K00232	acyl-CoA oxidase [EC:1.3.3.6]	-0.42	2.9E-295	1.1E-293	-0.43	yes
K01563	haloalkane dehalogenase [EC:3.8.1.5]	-0.42	3.7E-295	1.4E-293	-0.42	yes
K05299	formate dehydrogenase (NADP+) alpha subunit [EC:1.17.1.10]	-0.42	4.3E-295	1.6E-293	-0.42	yes
K01940	argininosuccinate synthase [EC:6.3.4.5]	-0.42	8.0E-295	2.9E-293	-0.39	
K04036	divinyl protochlorophyllide a 8-vinyl-reductase [EC:1.-.-.-]	-0.42	1.1E-294	3.9E-293	-0.42	yes
K00588	caffeoyl-CoA O-methyltransferase [EC:2.1.1.104]	-0.42	1.6E-294	5.6E-293	-0.43	yes
K02274	cytochrome c oxidase subunit I [EC:7.1.1.9]	-0.42	2.4E-294	8.3E-293	-0.43	yes
K01283	peptidyl-dipeptidase A [EC:3.4.15.1]	-0.42	2.7E-294	9.3E-293	-0.42	yes
K10217	aminomuconate-semialdehyde/2-hydroxymuconate-6-semialdehyde dehydrogenase [EC:1.2.1.32 1.2.1.85]	-0.42	3.6E-294	1.2E-292	-0.42	yes
K14162	error-prone DNA polymerase [EC:2.7.7.7]	-0.42	6.4E-294	2.1E-292	-0.43	yes
K00148	glutathione-independent formaldehyde dehydrogenase [EC:1.2.1.46]	-0.42	6.7E-294	2.2E-292	-0.43	yes
K00486	kynurenine 3-monooxygenase [EC:1.14.13.9]	-0.42	9.5E-294	3.1E-292	-0.42	yes
K07717	two-component system, sensor histidine kinase GlnK [EC:2.7.13.3]	-0.42	1.4E-293	4.5E-292	-0.41	yes
K00318	proline dehydrogenase [EC:1.5.5.2]	-0.42	1.6E-293	5.1E-292	-0.43	yes
K00354	NADPH2 dehydrogenase [EC:1.6.99.1]	-0.42	2.3E-293	7.3E-292	-0.44	yes
K01937	CTP synthase [EC:6.3.4.2]	-0.42	2.8E-293	8.8E-292	-0.40	
K11782	chorismate dehydratase [EC:4.2.1.151]	-0.42	1.1E-292	3.4E-291	-0.43	yes
K06118	UDP-sulfoquinovose synthase [EC:3.13.1.1]	-0.42	1.7E-292	5.2E-291	-0.42	yes
K13057	trehalose synthase [EC:2.4.1.245]	-0.42	3.7E-292	1.1E-290	-0.43	yes
K05827	[lysine-biosynthesis-protein LysW]---L-2-aminoadipate ligase [EC:6.3.2.43]	-0.42	4.7E-292	1.4E-290	-0.43	yes
K06012	spore protease [EC:3.4.24.78]	-0.41	2.8E-291	8.4E-290	-0.44	yes
K15629	fatty-acid peroxygenase [EC:1.11.2.4]	-0.41	3.1E-291	9.2E-290	-0.43	yes
K13924	two-component system, chemotaxis family, CheB/CheR fusion protein [EC:2.1.1.80 3.1.1.61]	-0.41	1.5E-290	4.4E-289	-0.43	yes
K00100	butanol dehydrogenase [EC:1.1.1.-]	-0.41	2.9E-290	8.4E-289	-0.45	yes
K03520	aerobic carbon-monoxide dehydrogenase large subunit [EC:1.2.5.3]	-0.41	3.1E-290	8.9E-289	-0.43	yes
K00531	nitrogenase delta subunit [EC:1.18.6.1]	-0.41	3.6E-290	1.0E-288	-0.42	yes
K13503	anthranilate synthase [EC:4.1.3.27]	-0.41	7.5E-290	2.1E-288	-0.44	yes
K01874	methionyl-tRNA synthetase [EC:6.1.1.10]	-0.41	2.1E-289	5.9E-288	-0.41	
K01777	proline racemase [EC:5.1.1.4]	-0.41	3.8E-289	1.1E-287	-0.44	yes
K01876	aspartyl-tRNA synthetase [EC:6.1.1.12]	-0.41	4.0E-289	1.1E-287	-0.40	
K04719	5,6-dimethylbenzimidazole synthase [EC:1.13.11.79]	-0.41	1.8E-288	4.9E-287	-0.43	yes
K01588	5-(carboxyamino)imidazole ribonucleotide mutase [EC:5.4.99.18]	-0.41	7.7E-288	2.1E-286	-0.40	
K07653	two-component system, OmpR family, sensor histidine kinase MprB [EC:2.7.13.3]	-0.41	1.1E-287	2.9E-286	-0.43	yes
K09809	CDP-glycerol glycerophosphotransferase [EC:2.7.8.12]	-0.41	3.2E-287	8.4E-286	-0.43	yes
K01965	propionyl-CoA carboxylase alpha chain [EC:6.4.1.3]	-0.41	3.2E-287	8.4E-286	-0.42	yes
K00133	aspartate-semialdehyde dehydrogenase [EC:1.2.1.11]	-0.41	9.6E-287	2.5E-285	-0.40	
K16188	tetraprenyl-beta-curcumene synthase [EC:4.2.3.130]	-0.41	1.3E-286	3.3E-285	-0.43	yes
K01907	acetoacetyl-CoA synthetase [EC:6.2.1.16]	-0.41	1.3E-286	3.3E-285	-0.42	yes
K03524	BirA family transcriptional regulator, biotin operon repressor / biotin---[acetyl-CoA-carboxylase] ligase [EC:6.3.4.15]	-0.41	1.4E-286	3.5E-285	-0.40	
K11779	FO synthase [EC:2.5.1.147 4.3.1.32]	-0.41	1.4E-286	3.5E-285	-0.42	yes
K09844	carotenoid 1,2-hydratase [EC:4.2.1.131]	-0.41	1.5E-286	3.7E-285	-0.42	yes

Predictor	Description	Pearson's r	P	FDR-adjusted P	Spearman's rho	Associated with fractures (P<0.05)
K00507	stearoyl-CoA desaturase (Delta-9 desaturase) [EC:1.14.19.1]	-0.41	1.6E-286	4.0E-285	-0.42	yes
K05521	ADP-ribosyl-[dinitrogen reductase] hydrolase [EC:3.2.2.24]	-0.41	1.2E-285	2.9E-284	-0.42	yes
K12995	O-antigen biosynthesis alpha-1,3-mannosyltransferase [EC:2.4.1.348 2.4.1.-]	-0.41	3.8E-285	9.2E-284	-0.43	yes
K10773	endonuclease III [EC:4.2.99.18]	-0.41	3.9E-285	9.4E-284	-0.39	
K00500	phenylalanine-4-hydroxylase [EC:1.14.16.1]	-0.41	5.9E-285	1.4E-283	-0.42	yes
K04766	acetoin utilization protein AcuA [EC:2.3.1.-]	-0.41	7.6E-285	1.8E-283	-0.42	yes
K01820	L-rhamnose isomerase / sugar isomerase [EC:5.3.1.14 5.3.1.-]	-0.41	8.5E-285	2.0E-283	-0.42	yes
K02340	DNA polymerase III subunit delta [EC:2.7.7.7]	-0.41	1.4E-284	3.3E-283	-0.40	yes
K01000	phospho-N-acetylmuramoyl-pentapeptide-transferase [EC:2.7.8.13]	-0.41	3.5E-284	8.1E-283	-0.40	
K00481	p-hydroxybenzoate 3-monooxygenase [EC:1.14.13.2]	-0.41	5.4E-284	1.2E-282	-0.43	yes
K10255	acyl-lipid omega-6 desaturase (Delta-12 desaturase) [EC:1.14.19.23 1.14.19.45]	-0.41	7.9E-284	1.8E-282	-0.42	yes
K01875	seryl-tRNA synthetase [EC:6.1.1.11]	-0.41	8.2E-284	1.9E-282	-0.40	
K14949	serine/threonine-protein kinase PknG [EC:2.7.11.1]	-0.41	1.2E-283	2.7E-282	-0.42	yes
K01113	alkaline phosphatase D [EC:3.1.3.1]	-0.41	3.4E-283	7.6E-282	-0.42	yes
K10187	germacradienol/geosmin synthase [EC:4.2.3.22 4.2.3.75 4.1.99.16]	-0.41	4.7E-283	1.0E-281	-0.42	yes
K01756	adenylosuccinate lyase [EC:4.3.2.2]	-0.41	4.9E-283	1.1E-281	-0.40	
K02276	cytochrome c oxidase subunit III [EC:7.1.1.9]	-0.41	1.1E-282	2.4E-281	-0.42	yes
K00082	5-amino-6-(5-phosphoribosylamino)uracil reductase [EC:1.1.1.193]	-0.41	1.3E-282	2.8E-281	-0.43	yes
K01555	fumarylacetoacetase [EC:3.7.1.2]	-0.41	4.2E-282	9.1E-281	-0.42	yes
K15011	two-component system, sensor histidine kinase RegB [EC:2.7.13.3]	-0.41	2.9E-281	6.2E-280	-0.42	yes
K10960	geranylgeranyl diphosphate/geranylgeranyl-bacteriochlorophyllide a reductase [EC:1.3.1.83 1.3.1.111]	-0.41	7.9E-281	1.7E-279	-0.42	yes
K13766	methylglutaconyl-CoA hydratase [EC:4.2.1.18]	-0.41	1.3E-280	2.7E-279	-0.42	yes
K01617	2-oxo-3-hexenedioate decarboxylase [EC:4.1.1.77]	-0.41	1.4E-280	2.9E-279	-0.42	yes
K01178	glucoamylase [EC:3.2.1.3]	-0.41	1.4E-280	2.9E-279	-0.42	yes
K01083	3-phytase [EC:3.1.3.8]	-0.41	9.2E-280	1.9E-278	-0.42	yes
K08651	thermitase [EC:3.4.21.66]	-0.41	9.9E-280	2.0E-278	-0.45	yes
K03333	cholesterol oxidase [EC:1.1.3.6]	-0.41	1.4E-279	2.9E-278	-0.42	yes
K06074	vitamin B12 transport system ATP-binding protein [EC:7.6.2.8]	0.41	2.2E-279	4.5E-278	0.30	
K01732	pectin lyase [EC:4.2.2.10]	-0.41	3.1E-279	6.2E-278	-0.43	yes
K04127	isopenicillin-N epimerase [EC:5.1.1.17]	-0.41	3.4E-279	6.7E-278	-0.42	yes
K03921	acyl-[acyl-carrier-protein] desaturase [EC:1.14.19.2 1.14.19.11 1.14.19.26]	-0.41	3.4E-279	6.7E-278	-0.41	yes
K00972	UDP-N-acetylglucosamine/UDP-N-acetylgalactosamine diphosphorylase [EC:2.7.7.23 2.7.7.83]	-0.41	7.9E-279	1.6E-277	-0.41	yes
K02853	enterobacterial common antigen polymerase [EC:2.4.1.-]	0.41	1.6E-278	3.1E-277	0.29	
K06399	stage IV sporulation protein B [EC:3.4.21.116]	-0.41	3.9E-278	7.6E-277	-0.41	yes
K06379	stage II sporulation protein AB (anti-sigma F factor) [EC:2.7.11.1]	-0.41	4.0E-278	7.7E-277	-0.41	yes
K06382	stage II sporulation protein E [EC:3.1.3.16]	-0.41	4.0E-278	7.7E-277	-0.41	yes
K00518	nickel superoxide dismutase [EC:1.15.1.1]	-0.41	6.3E-278	1.2E-276	-0.42	yes
K02345	DNA polymerase III subunit theta [EC:2.7.7.7]	0.41	7.0E-278	1.3E-276	0.30	
K02563	UDP-N-acetylglucosamine--N-acetylmuramyl-(pentapeptide) pyrophosphoryl-undecaprenol N-acetylglucosamine transferase [EC:2.4.1.227]	-0.41	1.1E-277	2.1E-276	-0.39	
K00643	5-aminolevulinate synthase [EC:2.3.1.37]	-0.41	4.3E-277	8.1E-276	-0.42	yes
K00446	catechol 2,3-dioxygenase [EC:1.13.11.2]	-0.41	6.4E-277	1.2E-275	-0.42	yes
K02335	DNA polymerase I [EC:2.7.7.7]	-0.41	1.2E-276	2.2E-275	-0.39	

Predictor	Description	Pearson's r	P	FDR-adjusted P	Spearman's rho	Associated with fractures (P<0.05)
K00167	2-oxoisovalerate dehydrogenase E1 component beta subunit [EC:1.2.4.4]	-0.41	1.8E-276	3.3E-275	-0.42	yes
K01476	arginase [EC:3.5.3.1]	-0.41	2.6E-276	4.7E-275	-0.43	yes
K12582	dTDP-N-acetylfucosamine:lipid II N-acetylfucosaminyltransferase [EC:2.4.1.325]	0.41	2.6E-276	4.7E-275	0.29	
K00849	galactokinase [EC:2.7.1.6]	-0.40	3.3E-276	6.0E-275	-0.36	
K03821	poly[(R)-3-hydroxyalkanoate] polymerase subunit PhaC [EC:2.3.1.304]	-0.40	3.7E-276	6.6E-275	-0.42	yes
K01096	phosphatidylglycerophosphatase B [EC:3.1.3.27 3.1.3.81 3.1.3.4 3.6.1.27]	0.40	5.6E-276	1.0E-274	0.29	
K07676	two-component system, NarL family, sensor histidine kinase RcsD [EC:2.7.13.3]	0.40	9.1E-276	1.6E-274	0.30	
K01969	3-methylcrotonyl-CoA carboxylase beta subunit [EC:6.4.1.4]	-0.40	3.6E-275	6.4E-274	-0.42	yes
K02344	DNA polymerase III subunit psi [EC:2.7.7.7]	0.40	5.6E-275	9.8E-274	0.28	
K06446	acyl-CoA dehydrogenase [EC:1.3.99.-]	-0.40	8.4E-275	1.5E-273	-0.42	yes
K16163	maleylpyruvate isomerase [EC:5.2.1.4]	-0.40	1.4E-274	2.4E-273	-0.42	
K12152	phosphatase NudJ [EC:3.6.1.-]	0.40	1.7E-274	2.9E-273	0.28	
K00301	sarcosine oxidase [EC:1.5.3.1]	-0.40	2.8E-274	4.8E-273	-0.43	yes
K07648	two-component system, OmpR family, aerobic respiration control sensor histidine kinase ArcB [EC:2.7.13.3]	0.40	2.8E-274	4.8E-273	0.28	
K08682	acyl carrier protein phosphodiesterase [EC:3.1.4.14]	0.40	3.2E-274	5.4E-273	0.29	
K00885	N-acylmannosamine kinase [EC:2.7.1.60]	0.40	4.2E-274	7.1E-273	0.30	
K00311	electron-transferring-flavoprotein dehydrogenase [EC:1.5.5.1]	-0.40	5.4E-274	9.1E-273	-0.42	yes
K00304	sarcosine oxidase, subunit delta [EC:1.5.3.1]	-0.40	2.0E-273	3.3E-272	-0.42	yes
K13015	UDP-N-acetyl-D-glucosamine dehydrogenase [EC:1.1.1.136]	-0.40	2.2E-273	3.6E-272	-0.42	yes
K00172	pyruvate ferredoxin oxidoreductase gamma subunit [EC:1.2.7.1]	-0.40	2.3E-273	3.8E-272	-0.41	yes
K13014	undecaprenyl phosphate-alpha-L-ara4FN deformylase [EC:3.5.1.-]	0.40	4.2E-273	6.9E-272	0.29	
K01407	protease III [EC:3.4.24.55]	0.40	4.4E-273	7.2E-272	0.28	
K10678	nitroreductase [EC:1.-.-.-]	0.40	4.6E-273	7.5E-272	0.28	
K02229	precorrin-3B synthase [EC:1.14.13.83]	-0.40	4.9E-273	7.9E-272	-0.42	yes
K02228	precorrin-6A synthase [EC:2.1.1.152]	-0.40	5.6E-273	9.0E-272	-0.42	yes
K12525	bifunctional aspartokinase / homoserine dehydrogenase 2 [EC:2.7.2.4 1.1.1.3]	0.40	8.8E-273	1.4E-271	0.28	
K08308	membrane-bound lytic murein transglycosylase E [EC:4.2.2.-]	0.40	1.2E-272	1.9E-271	0.30	
K07251	thiamine kinase [EC:2.7.1.89]	0.40	1.3E-272	2.1E-271	0.30	
K02482	two-component system, NtrC family, sensor kinase [EC:2.7.13.3]	-0.40	1.7E-272	2.7E-271	-0.41	yes
K01796	alpha-methylacyl-CoA racemase [EC:5.1.99.4]	-0.40	1.8E-272	2.8E-271	-0.42	yes
K00249	acyl-CoA dehydrogenase [EC:1.3.8.7]	-0.40	2.1E-272	3.3E-271	-0.42	yes
K05368	NAD(P)H-flavin reductase [EC:1.5.1.41]	0.40	3.6E-272	5.6E-271	0.28	
K01933	phosphoribosylformylglycinamidine cyclo-ligase [EC:6.3.3.1]	-0.40	5.8E-272	8.9E-271	-0.39	
K00594	alditol oxidase [EC:1.1.3.41]	-0.40	9.0E-272	1.4E-270	-0.42	yes
K00840	succinylornithine aminotransferase [EC:2.6.1.81]	0.40	1.0E-271	1.5E-270	0.31	
K15371	glutamate dehydrogenase [EC:1.4.1.2]	-0.40	2.2E-271	3.3E-270	-0.42	yes
K03793	pteridine reductase [EC:1.5.1.33]	-0.40	3.0E-271	4.5E-270	-0.41	yes
K12500	thioesterase III [EC:3.1.2.-]	0.40	3.1E-271	4.7E-270	0.27	
K08324	succinate-semialdehyde dehydrogenase [EC:1.2.1.16 1.2.1.24]	0.40	3.5E-271	5.2E-270	0.28	
K01567	peptidoglycan-N-acetylmuramic acid deacetylase [EC:3.5.1.-]	-0.40	4.2E-271	6.2E-270	-0.40	yes
K00892	inosine kinase [EC:2.7.1.73]	0.40	5.3E-271	7.8E-270	0.28	
K13810	transaldolase / glucose-6-phosphate isomerase [EC:2.2.1.2 5.3.1.9]	-0.40	1.5E-270	2.2E-269	-0.43	

Predictor	Description	Pearson's r	P	FDR-adjusted P	Spearman's rho	Associated with fractures (P<0.05)
K00305	sarcosine oxidase, subunit gamma [EC:1.5.3.1]	-0.40	1.6E-270	2.3E-269	-0.42	yes
K01457	allophanate hydrolase [EC:3.5.1.54]	-0.40	1.8E-270	2.6E-269	-0.42	yes
K13485	2-oxo-4-hydroxy-4-carboxy-5-ureidoimidazoline decarboxylase [EC:4.1.1.97]	-0.40	1.9E-270	2.8E-269	-0.43	yes
K00830	alanine-glyoxylate transaminase / serine-glyoxylate transaminase / serine-pyruvate transaminase [EC:2.6.1.44 2.6.1.45 2.6.1.51]	-0.40	2.1E-270	3.0E-269	-0.42	yes
K08723	5'-nucleotidase [EC:3.1.3.5]	0.40	2.9E-270	4.2E-269	0.28	
K01093	4-phytase / acid phosphatase [EC:3.1.3.26 3.1.3.2]	0.40	6.0E-270	8.6E-269	0.29	
K05590	ATP-dependent RNA helicase SrmB [EC:3.6.4.13]	0.40	6.8E-270	9.7E-269	0.27	
K06443	lycopene beta-cyclase [EC:5.5.1.19]	-0.40	7.6E-270	1.1E-268	-0.42	yes
K00685	arginyl-tRNA---protein transferase [EC:2.3.2.8]	-0.40	9.6E-270	1.4E-268	-0.42	yes
K02362	enterobactin synthetase component D [EC:6.3.2.14 2.7.8.-]	0.40	1.0E-269	1.4E-268	0.27	
K00884	N-acetylglucosamine kinase [EC:2.7.1.59]	0.40	1.0E-269	1.4E-268	0.28	
K03170	reverse gyrase [EC:5.6.2.2 3.6.4.12]	-0.40	1.2E-269	1.7E-268	-0.45	yes
K00947	molybdenum storage protein	-0.40	1.3E-269	1.8E-268	-0.42	yes
K03207	colanic acid biosynthesis protein WcaH [EC:3.6.1.-]	0.40	2.2E-269	3.0E-268	0.29	
K07751	PepB aminopeptidase [EC:3.4.11.23]	0.40	2.6E-269	3.6E-268	0.27	
K00998	CDP-diacylglycerol---serine O-phosphatidyltransferase [EC:2.7.8.8]	0.40	2.7E-269	3.7E-268	0.27	
K02341	DNA polymerase III subunit delta' [EC:2.7.7.7]	-0.40	2.8E-269	3.8E-268	-0.38	
K04343	streptomycin 6-kinase [EC:2.7.1.72]	-0.40	3.5E-269	4.7E-268	-0.42	yes
K13069	diguanylate cyclase [EC:2.7.7.65]	0.40	3.7E-269	5.0E-268	0.29	
K11938	HMP-PP phosphatase [EC:3.6.1.-]	0.40	4.0E-269	5.4E-268	0.31	
K08295	2-aminobenzoate-CoA ligase [EC:6.2.1.32]	-0.40	5.0E-269	6.7E-268	-0.42	yes
K00274	monoamine oxidase [EC:1.4.3.4]	-0.40	5.9E-269	7.8E-268	-0.41	yes
K00686	protein-glutamine gamma-glutamyltransferase [EC:2.3.2.13]	-0.40	1.0E-268	1.3E-267	-0.43	yes
K10011	UDP-4-amino-4-deoxy-L-arabinose formyltransferase / UDP-glucuronic acid dehydrogenase (UDP-4-keto-hexauronic acid decarboxylating) [EC:2.1.2.13 1.1.1.305]	0.40	1.6E-268	2.1E-267	0.28	
K07643	two-component system, OmpR family, sensor histidine kinase BasS [EC:2.7.13.3]	0.40	2.2E-268	2.9E-267	0.30	
K11933	NADH oxidoreductase Hcr [EC:1.-.-.-]	0.40	2.2E-268	2.9E-267	0.29	
K01460	glutathionylspermidine amidase/synthetase [EC:3.5.1.78 6.3.1.8]	0.40	3.0E-268	3.9E-267	0.31	
K14259	2-dehydro-3-deoxy-D-arabinonate dehydratase [EC:4.2.1.141]	-0.40	3.5E-268	4.5E-267	-0.41	yes
K07701	two-component system, CitB family, sensor histidine kinase DcuS [EC:2.7.13.3]	0.40	3.9E-268	5.0E-267	0.29	
K00702	cellobiose phosphorylase [EC:2.4.1.20]	-0.40	4.2E-268	5.4E-267	-0.40	yes
K12945	GDP-mannose pyrophosphatase NudK [EC:3.6.1.-]	0.40	2.0E-267	2.6E-266	0.30	
K07264	4-amino-4-deoxy-L-arabinose transferase [EC:2.4.2.43]	0.40	4.4E-267	5.6E-266	0.28	
K12974	KDO2-lipid IV(A) palmitoleoyltransferase [EC:2.3.1.242]	0.40	5.0E-267	6.3E-266	0.30	
K03411	chemotaxis protein CheD [EC:3.5.1.44]	-0.40	7.8E-267	9.8E-266	-0.40	yes
K12944	nucleoside triphosphatase [EC:3.6.1.-]	0.40	3.4E-266	4.3E-265	0.30	
K13938	dihydromonapterin reductase / dihydrofolate reductase [EC:1.5.1.50 1.5.1.3]	0.40	8.7E-266	1.1E-264	0.27	
K00760	hypoxanthine phosphoribosyltransferase [EC:2.4.2.8]	-0.40	1.0E-265	1.2E-264	-0.38	
K08320	(d)CTP diphosphatase [EC:3.6.1.65]	0.40	1.2E-265	1.5E-264	0.30	
K07811	trimethylamine-N-oxide reductase (cytochrome c) [EC:1.7.2.3]	0.40	1.8E-265	2.2E-264	0.28	
K05996	carboxypeptidase T [EC:3.4.17.18]	-0.40	1.8E-265	2.2E-264	-0.43	yes
K02560	lauroyl-Kdo2-lipid IVA myristoyltransferase [EC:2.3.1.243]	0.40	2.1E-265	2.6E-264	0.26	
K01090	protein phosphatase [EC:3.1.3.16]	-0.40	2.6E-265	3.2E-264	-0.41	yes

Predictor	Description	Pearson's r	P	FDR-adjusted P	Spearman's rho	Associated with fractures (P<0.05)
K07640	two-component system, OmpR family, sensor histidine kinase CpxA [EC:2.7.13.3]	0.40	4.7E-265	5.7E-264	0.25	
K01630	2-dehydro-3-deoxyglucarate aldolase [EC:4.1.2.20]	0.40	1.7E-264	2.1E-263	0.25	
K11258	acetolactate synthase II small subunit [EC:2.2.1.6]	0.40	2.2E-264	2.7E-263	0.26	
K00491	nitric-oxide synthase, bacterial [EC:1.14.14.47]	-0.40	2.7E-264	3.2E-263	-0.42	yes
K00253	isovaleryl-CoA dehydrogenase [EC:1.3.8.4]	-0.40	3.2E-264	3.8E-263	-0.41	yes
K01707	5-dehydro-4-deoxyglucarate dehydratase [EC:4.2.1.41]	-0.40	3.4E-264	4.1E-263	-0.41	yes
K02364	L-serine---[L-seryl-carrier protein] ligase [EC:6.3.2.14 6.2.1.72]	0.40	4.8E-264	5.7E-263	0.27	
K12957	alcohol/geraniol dehydrogenase (NADP+) [EC:1.1.1.2 1.1.1.183]	0.40	5.3E-264	6.3E-263	0.27	
K12973	lipid IVA palmitoyltransferase [EC:2.3.1.251]	0.40	5.5E-264	6.5E-263	0.28	
K00273	D-amino-acid oxidase [EC:1.4.3.3]	-0.40	5.6E-264	6.6E-263	-0.42	yes
K14048	urease subunit gamma/beta [EC:3.5.1.5]	-0.40	9.4E-264	1.1E-262	-0.41	yes
K07229	ferric-chelate reductase (NADPH) [EC:1.16.1.9]	0.40	1.2E-263	1.4E-262	0.31	
K05951	NAD+---dinitrogen-reductase ADP-D-ribosyltransferase [EC:2.4.2.37]	-0.40	1.4E-263	1.6E-262	-0.40	yes
K14051	c-di-GMP phosphodiesterase Gmr [EC:3.1.4.52]	0.40	1.7E-263	2.0E-262	0.31	
K12660	2-dehydro-3-deoxy-L-rhamnonate aldolase [EC:4.1.2.53]	0.40	2.2E-263	2.5E-262	0.31	
K09065	N-acetylornithine carbamoyltransferase [EC:2.1.3.9]	-0.40	2.2E-263	2.5E-262	-0.41	yes
K14744	prophage endopeptidase [EC:3.4.-.-]	0.40	2.5E-263	2.9E-262	0.31	
K09018	pyrimidine oxygenase [EC:1.14.99.46]	0.40	3.5E-263	4.0E-262	0.29	
K10017	histidine transport system ATP-binding protein [EC:7.4.2.1]	0.40	4.1E-263	4.7E-262	0.26	
K11201	fructose-like PTS system EIIA component [EC:2.7.1.-]	0.40	4.3E-263	4.9E-262	0.31	
K00166	2-oxoisovalerate dehydrogenase E1 component alpha subunit [EC:1.2.4.4]	-0.40	4.9E-263	5.5E-262	-0.41	yes
K11391	23S rRNA (guanine1835-N2)-methyltransferase [EC:2.1.1.174]	0.40	5.6E-263	6.3E-262	0.30	
K07757	sugar-phosphatase [EC:3.1.3.23]	0.40	6.5E-263	7.3E-262	0.29	
K00663	aminoglycoside 6'-N-acetyltransferase [EC:2.3.1.82]	-0.40	7.6E-263	8.5E-262	-0.41	yes
K11202	fructose-like PTS system EIIB component [EC:2.7.1.-]	0.40	7.8E-263	8.7E-262	0.29	
K00992	N-acetyl-alpha-D-muramate 1-phosphate uridylyltransferase [EC:2.7.7.99]	-0.40	1.1E-262	1.2E-261	-0.41	yes
K13540	precorrin-2 C20-methyltransferase / precorrin-3B C17-methyltransferase [EC:2.1.1.130 2.1.1.131]	-0.40	1.1E-262	1.2E-261	-0.42	yes
K16652	decaprenylphospho-beta-D-erythro-pentofuranosid-2-ulose 2-reductase [EC:1.1.1.333]	-0.40	1.6E-262	1.8E-261	-0.41	yes
K01867	tryptophanyl-tRNA synthetase [EC:6.1.1.2]	-0.40	2.3E-262	2.5E-261	-0.38	
K01477	allantoicase [EC:3.5.3.4]	-0.40	3.2E-262	3.5E-261	-0.41	yes
K05829	LysW-gamma-L-alpha-aminoadipyl-6-phosphate/LysW-L-glutamyl-5-phosphate reductase [EC:1.2.1.103 1.2.1.106]	-0.40	3.9E-262	4.2E-261	-0.42	yes
K01169	ribonuclease I (enterobacter ribonuclease) [EC:4.6.1.21]	0.40	4.0E-262	4.3E-261	0.29	
K01392	thimet oligopeptidase [EC:3.4.24.15]	-0.40	7.3E-262	7.9E-261	-0.41	yes
K09470	gamma-glutamylputrescine synthase [EC:6.3.1.11]	0.39	1.4E-261	1.5E-260	0.31	
K02506	leader peptidase HopD [EC:3.4.23.43]	0.39	1.5E-261	1.6E-260	0.30	
K00456	cysteine dioxygenase [EC:1.13.11.20]	-0.39	2.2E-261	2.3E-260	-0.41	yes
K10674	ectoine hydroxylase [EC:1.14.11.55]	-0.39	2.5E-261	2.7E-260	-0.41	yes
K10797	2-enoate reductase [EC:1.3.1.31]	-0.39	3.3E-261	3.5E-260	-0.40	yes
K00919	4-diphosphocytidyl-2-C-methyl-D-erythritol kinase [EC:2.7.1.148]	-0.39	5.0E-261	5.3E-260	-0.39	
K01746	formiminotetrahydrofolate cyclodeaminase [EC:4.3.1.4]	-0.39	7.4E-261	7.8E-260	-0.40	yes
K05994	bacterial leucyl aminopeptidase [EC:3.4.11.10]	-0.39	1.5E-260	1.6E-259	-0.41	yes
K00282	glycine dehydrogenase subunit 1 [EC:1.4.4.2]	-0.39	1.7E-260	1.8E-259	-0.40	yes

Predictor	Description	Pearson's r	P	FDR-adjusted P	Spearman's rho	Associated with fractures (P<0.05)
K03788	acid phosphatase (class B) [EC:3.1.3.2]	0.39	2.4E-260	2.5E-259	0.29	
K03184	3-demethoxyubiquinol 3-hydroxylase [EC:1.14.99.60]	0.39	2.5E-260	2.6E-259	0.25	
K03078	3-dehydro-L-gulonate-6-phosphate decarboxylase [EC:4.1.1.85]	0.39	2.8E-260	2.9E-259	0.26	
K11731	citronellyl-CoA dehydrogenase [EC:1.3.99.-]	-0.39	2.9E-260	3.0E-259	-0.40	yes
K15894	UDP-N-acetylglucosamine 4,6-dehydratase/5-epimerase [EC:4.2.1.115 5.1.3.-]	-0.39	5.4E-260	5.5E-259	-0.42	yes
K02846	N-methyl-L-tryptophan oxidase [EC:1.5.3.-]	0.39	7.2E-260	7.4E-259	0.28	
K07589	D-erythro-7,8-dihydroneopterin triphosphate epimerase [EC:5.1.99.7]	0.39	1.7E-259	1.7E-258	0.26	
K00283	glycine dehydrogenase subunit 2 [EC:1.4.4.2]	-0.39	1.9E-259	1.9E-258	-0.40	yes
K03212	23S rRNA (uracil747-C5)-methyltransferase [EC:2.1.1.189]	0.39	2.2E-259	2.2E-258	0.25	
K00883	2-dehydro-3-deoxygalactonokinase [EC:2.7.1.58]	0.39	4.2E-259	4.2E-258	0.26	
K02438	glycogen debranching enzyme [EC:3.2.1.196]	0.39	6.9E-259	6.9E-258	0.25	
K00765	ATP phosphoribosyltransferase [EC:2.4.2.17]	-0.39	8.5E-259	8.5E-258	-0.38	
K07674	two-component system, NarL family, nitrate/nitrite sensor histidine kinase NarQ [EC:2.7.13.3]	0.39	1.4E-258	1.4E-257	0.28	
K08348	formate dehydrogenase-N, alpha subunit [EC:1.17.5.3]	0.39	1.7E-258	1.7E-257	0.31	
K14267	N-succinyldiaminopimelate aminotransferase [EC:2.6.1.17]	-0.39	1.9E-258	1.9E-257	-0.41	yes
K09883	cobaltochelatase CobT [EC:6.6.1.2]	-0.39	2.1E-258	2.1E-257	-0.41	yes
K00102	D-lactate dehydrogenase (cytochrome) [EC:1.1.2.4]	-0.39	2.3E-258	2.3E-257	-0.41	yes
K15898	pseudaminic acid synthase [EC:2.5.1.97]	-0.39	2.8E-258	2.8E-257	-0.43	yes
K00094	galactitol-1-phosphate 5-dehydrogenase [EC:1.1.1.251]	0.39	3.0E-258	2.9E-257	0.30	
K00436	NAD-reducing hydrogenase large subunit [EC:1.12.1.2]	-0.39	3.3E-258	3.2E-257	-0.41	yes
K15510	coenzyme F420-dependent glucose-6-phosphate dehydrogenase [EC:1.1.98.2]	-0.39	3.8E-258	3.7E-257	-0.42	yes
K01521	CDP-diacylglycerol pyrophosphatase [EC:3.6.1.26]	0.39	6.5E-258	6.3E-257	0.25	
K12583	phosphatidylinositol alpha 1,6-mannosyltransferase [EC:2.4.1.-]	-0.39	1.4E-257	1.4E-256	-0.41	yes
K06957	tRNA(Met) cytidine acetyltransferase [EC:2.3.1.193]	0.39	1.8E-257	1.7E-256	0.26	
K03525	type III pantothenate kinase [EC:2.7.1.33]	-0.39	2.3E-257	2.2E-256	-0.34	
K08314	fructose-6-phosphate aldolase 2 [EC:4.1.2.-]	0.39	2.5E-257	2.4E-256	0.28	
K07319	adenine-specific DNA-methyltransferase [EC:2.1.1.72]	0.39	2.5E-257	2.4E-256	0.28	
K00813	aspartate aminotransferase [EC:2.6.1.1]	0.39	2.9E-257	2.8E-256	0.25	
K07700	two-component system, CitB family, cit operon sensor histidine kinase CitA [EC:2.7.13.3]	0.39	3.3E-257	3.1E-256	0.31	
K01968	3-methylcrotonyl-CoA carboxylase alpha subunit [EC:6.4.1.4]	-0.39	3.3E-257	3.1E-256	-0.41	yes
K13574	hydroxycarboxylate dehydrogenase B [EC:1.1.1.237 1.1.1.-]	0.39	3.8E-257	3.6E-256	0.27	
K03819	putative colanic acid biosynthesis acetyltransferase WcaB [EC:2.3.1.-]	0.39	4.0E-257	3.8E-256	0.30	
K00271	valine dehydrogenase (NAD+) [EC:1.4.1.23]	-0.39	4.6E-257	4.3E-256	-0.41	
K00453	tryptophan 2,3-dioxygenase [EC:1.13.11.11]	-0.39	5.7E-257	5.3E-256	-0.41	yes
K06134	3-demethoxyubiquinol 3-hydroxylase [EC:1.14.99.60]	-0.39	6.5E-257	6.1E-256	-0.41	yes
K01400	bacillolysin [EC:3.4.24.28]	-0.39	7.3E-257	6.8E-256	-0.41	yes
K01766	cysteine sulfinate desulfinase [EC:4.4.1.-]	0.39	9.1E-257	8.4E-256	0.26	
K07637	two-component system, OmpR family, sensor histidine kinase PhoQ [EC:2.7.13.3]	0.39	1.0E-256	9.2E-256	0.24	
K08322	L-gulonate 5-dehydrogenase [EC:1.1.1.380]	0.39	1.3E-256	1.2E-255	0.27	
K00091	dihydroflavonol-4-reductase [EC:1.1.1.219]	-0.39	1.4E-256	1.3E-255	-0.41	yes
K04040	chlorophyll/bacteriochlorophyll a synthase [EC:2.5.1.62 2.5.1.133]	-0.39	1.7E-256	1.6E-255	-0.41	yes
K08313	fructose-6-phosphate aldolase 1 [EC:4.1.2.-]	0.39	2.8E-256	2.6E-255	0.25	

Predictor	Description	Pearson's r	P	FDR-adjusted P	Spearman's rho	Associated with fractures (P<0.05)
K00696	sucrose-phosphate synthase [EC:2.4.1.14]	-0.39	4.3E-256	3.9E-255	-0.41	yes
K09020	ureidoacrylate peracid hydrolase [EC:3.5.1.110]	0.39	5.5E-256	5.0E-255	0.28	
K09473	gamma-glutamyl-gamma-aminobutyrate hydrolase [EC:3.5.1.94]	0.39	6.4E-256	5.8E-255	0.31	
K03472	D-erythrose 4-phosphate dehydrogenase [EC:1.2.1.72]	0.39	7.1E-256	6.4E-255	0.24	
K01825	3-hydroxyacyl-CoA dehydrogenase / enoyl-CoA hydratase / 3-hydroxybutyryl-CoA epimerase / enoyl-CoA isomerase [EC:1.1.1.35 4.2.1.17 5.1.2.3 5.3.3.8]	0.39	1.3E-255	1.2E-254	0.24	
K07026	mannosyl-3-phosphoglycerate phosphatase [EC:3.1.3.70]	0.39	2.7E-255	2.4E-254	0.26	
K01416	snapalysin [EC:3.4.24.77]	-0.39	3.3E-255	2.9E-254	-0.42	
K07806	UDP-4-amino-4-deoxy-L-arabinose-oxoglutarate aminotransferase [EC:2.6.1.87]	0.39	4.7E-255	4.2E-254	0.28	
K01449	N-acetylmuramoyl-L-alanine amidase [EC:3.5.1.28]	-0.39	4.8E-255	4.3E-254	-0.39	yes
K01150	deoxyribonuclease I [EC:3.1.21.1]	0.39	4.9E-255	4.3E-254	0.24	
K15408	cytochrome c oxidase subunit I+III [EC:7.1.1.9]	-0.39	5.1E-255	4.5E-254	-0.41	yes
K01505	1-aminocyclopropane-1-carboxylate deaminase [EC:3.5.99.7]	-0.39	7.3E-255	6.4E-254	-0.41	yes
K01704	3-isopropylmalate/(R)-2-methylmalate dehydratase small subunit [EC:4.2.1.33 4.2.1.35]	-0.39	5.1E-254	4.5E-253	-0.37	
K00496	alkane 1-monooxygenase [EC:1.14.15.3]	-0.39	5.9E-254	5.2E-253	-0.40	yes
K01067	acetyl-CoA hydrolase [EC:3.1.2.1]	-0.39	6.0E-254	5.2E-253	-0.40	yes
K16146	maltokinase [EC:2.7.1.175]	-0.39	6.5E-254	5.7E-253	-0.41	yes
K15526	L-cysteine:1D-myo-inositol 2-amino-2-deoxy-alpha-D-glucopyranoside ligase [EC:6.3.1.13]	-0.39	8.6E-254	7.5E-253	-0.41	yes
K00141	benzaldehyde dehydrogenase (NAD) [EC:1.2.1.28]	-0.39	9.6E-254	8.3E-253	-0.42	yes
K02848	heptose I phosphotransferase [EC:2.7.1.-]	0.39	1.2E-253	1.0E-252	0.25	
K07537	cyclohexa-1,5-dienecarbonyl-CoA hydratase [EC:4.2.1.100]	-0.39	1.3E-253	1.1E-252	-0.41	yes
K03815	xanthosine phosphorylase [EC:2.4.2.-]	0.39	1.6E-253	1.4E-252	0.30	
K12144	hydrogenase-4 component I [EC:1.-.-.-]	0.39	2.1E-253	1.8E-252	0.31	
K12508	feruloyl-CoA synthase [EC:6.2.1.34]	-0.39	3.6E-253	3.1E-252	-0.41	yes
K00171	pyruvate ferredoxin oxidoreductase delta subunit [EC:1.2.7.1]	-0.39	4.4E-253	3.7E-252	-0.40	yes
K11066	N-acetylmuramoyl-L-alanine amidase [EC:3.5.1.28]	0.39	4.8E-253	4.1E-252	0.24	
K14980	two-component system, OmpR family, sensor histidine kinase ChvG [EC:2.7.13.3]	-0.39	6.3E-253	5.3E-252	-0.42	yes
K08646	peptidyl-Lys metalloendopeptidase [EC:3.4.24.20]	-0.39	6.6E-253	5.6E-252	-0.42	yes
K02439	thiosulfate sulfurtransferase [EC:2.8.1.1]	0.39	7.0E-253	5.9E-252	0.24	
K05828	[amino group carrier protein]-L-2-aminoadipate 6-kinase [EC:2.7.2.17]	-0.39	8.7E-253	7.3E-252	-0.41	yes
K12142	hydrogenase-4 component G [EC:1.-.-.-]	0.39	9.0E-253	7.5E-252	0.31	
K05526	succinylglutamate desuccinylase [EC:3.5.1.96]	0.39	9.9E-253	8.3E-252	0.25	
K02336	DNA polymerase II [EC:2.7.7.7]	0.39	1.1E-252	9.1E-252	0.24	
K15922	sulfoquinovosidase [EC:3.2.1.199]	0.39	2.1E-252	1.7E-251	0.27	
K03214	RNA methyltransferase, TrmH family [EC:2.1.1.-]	0.39	3.1E-252	2.6E-251	0.25	
K13482	xanthine dehydrogenase large subunit [EC:1.17.1.4]	-0.39	3.4E-252	2.8E-251	-0.41	yes
K01056	peptidyl-tRNA hydrolase, PTH1 family [EC:3.1.1.29]	-0.39	3.6E-252	3.0E-251	-0.37	
K01469	5-oxoprolinase (ATP-hydrolysing) [EC:3.5.2.9]	-0.39	4.1E-252	3.4E-251	-0.40	yes
K14731	epsilon-lactone hydrolase [EC:3.1.1.83]	-0.39	5.5E-252	4.5E-251	-0.41	yes
K00569	thiopurine S-methyltransferase [EC:2.1.1.67]	-0.39	6.1E-252	5.0E-251	-0.40	yes
K01631	2-dehydro-3-deoxyphosphogalactonate aldolase [EC:4.1.2.21]	0.39	6.7E-252	5.4E-251	0.24	
K12145	hydrogenase-4 component J [EC:1.-.-.-]	0.39	7.8E-252	6.3E-251	0.31	
K05711	2,3-dihydroxy-2,3-dihydrophenylpropionate dehydrogenase [EC:1.3.1.87]	0.39	1.5E-251	1.2E-250	0.31	

Predictor	Description	Pearson's r	P	FDR-adjusted P	Spearman's rho	Associated with fractures (P<0.05)
K00126	formate dehydrogenase subunit delta [EC:1.17.1.9]	-0.39	1.9E-251	1.5E-250	-0.41	yes
K02476	two-component system, CitB family, sensor kinase [EC:2.7.13.3]	-0.39	2.2E-251	1.8E-250	-0.42	yes
K03080	NA	0.39	2.6E-251	2.1E-250	0.32	
K11337	bacteriochlorophyllide a dehydrogenase [EC:1.1.1.396]	-0.39	2.6E-251	2.1E-250	-0.40	yes
K09024	flavin reductase [EC:1.5.1.-]	0.39	2.7E-251	2.2E-250	0.26	
K02850	heptose II phosphotransferase [EC:2.7.1.-]	0.39	3.2E-251	2.5E-250	0.30	
K00019	3-hydroxybutyrate dehydrogenase [EC:1.1.1.30]	-0.39	7.0E-251	5.6E-250	-0.41	yes
K03046	DNA-directed RNA polymerase subunit beta' [EC:2.7.7.6]	-0.39	7.1E-251	5.6E-250	-0.37	
K12503	short-chain Z-isoprenyl diphosphate synthase [EC:2.5.1.68]	-0.39	2.6E-250	2.1E-249	-0.41	yes
K07539	6-oxocyclohex-1-ene-carbonyl-CoA hydrolase [EC:3.7.1.21]	-0.39	2.7E-250	2.1E-249	-0.42	yes
K00114	alcohol dehydrogenase (cytochrome c) [EC:1.1.2.8]	-0.39	2.9E-250	2.3E-249	-0.41	yes
K06222	2,5-diketo-D-gluconate reductase B [EC:1.1.1.346]	0.39	3.9E-250	3.1E-249	0.24	
K08306	membrane-bound lytic murein transglycosylase C [EC:4.2.2.-]	0.39	1.3E-249	1.0E-248	0.24	
K05708	3-phenylpropionate/trans-cinnamate dioxygenase subunit alpha [EC:1.14.12.19]	0.39	1.9E-249	1.5E-248	0.31	
K03405	magnesium chelatase subunit I [EC:6.6.1.1]	-0.39	2.0E-249	1.6E-248	-0.43	yes
K11264	methylmalonyl-CoA decarboxylase [EC:4.1.1.-]	0.39	2.3E-249	1.8E-248	0.28	
K11532	fructose-1,6-bisphosphatase II / sedoheptulose-1,7-bisphosphatase [EC:3.1.3.11 3.1.3.37]	-0.39	2.8E-249	2.2E-248	-0.42	yes
K07639	two-component system, OmpR family, sensor histidine kinase RstB [EC:2.7.13.3]	0.39	3.3E-249	2.5E-248	0.24	
K06447	succinylglutamic semialdehyde dehydrogenase [EC:1.2.1.71]	0.39	3.3E-249	2.5E-248	0.24	
K15372	taurine---2-oxoglutarate transaminase [EC:2.6.1.55]	-0.39	3.5E-249	2.7E-248	-0.42	yes
K01039	glutaconate CoA-transferase, subunit A [EC:2.8.3.12]	-0.39	4.3E-249	3.3E-248	-0.40	yes
K02079	N-acetylgalactosamine-6-phosphate deacetylase [EC:3.5.1.25]	0.39	5.7E-249	4.4E-248	0.30	
K02591	nitrogenase molybdenum-iron protein beta chain [EC:1.18.6.1]	-0.39	6.8E-249	5.2E-248	-0.42	yes
K01004	phosphatidylcholine synthase [EC:2.7.8.24]	-0.39	7.0E-249	5.3E-248	-0.41	yes
K16171	fumarylacetoacetate (FAA) hydrolase [EC:3.7.1.2]	-0.39	1.4E-248	1.1E-247	-0.41	yes
K07673	two-component system, NarL family, nitrate/nitrite sensor histidine kinase NarX [EC:2.7.13.3]	0.39	1.7E-248	1.3E-247	0.23	
K00132	acetaldehyde dehydrogenase (acetylating) [EC:1.2.1.10]	-0.39	2.1E-248	1.6E-247	-0.39	yes
K14136	decaprenyl-phosphate phosphoribosyltransferase [EC:2.4.2.45]	-0.39	2.8E-248	2.1E-247	-0.41	yes
K05851	adenylate cyclase, class 1 [EC:4.6.1.1]	0.39	3.0E-248	2.3E-247	0.23	
K15022	formate dehydrogenase (NADP+) beta subunit [EC:1.17.1.10]	-0.39	7.8E-248	5.8E-247	-0.41	yes
K01941	urea carboxylase [EC:6.3.4.6]	-0.39	8.5E-248	6.3E-247	-0.41	yes
K02480	two-component system, NarL family, sensor kinase [EC:2.7.13.3]	-0.39	9.6E-248	7.2E-247	-0.41	yes
K01870	isoleucyl-tRNA synthetase [EC:6.1.1.5]	-0.39	1.0E-247	7.4E-247	-0.37	
K05713	2,3-dihydroxyphenylpropionate 1,2-dioxygenase [EC:1.13.11.16]	0.38	2.7E-247	2.0E-246	0.25	
K13541	cobalt-precorrin 5A hydrolase / cobalt-factor III methyltransferase / precorrin-3B C17-methyltransferase [EC:3.7.1.12 2.1.1.272 2.1.1.131]	-0.38	6.8E-247	5.0E-246	-0.40	yes
K01160	crossover junction endodeoxyribonuclease RusA [EC:3.1.21.10]	0.38	8.4E-247	6.2E-246	0.29	
K01194	alpha,alpha-trehalase [EC:3.2.1.28]	0.38	9.6E-247	7.1E-246	0.25	
K03660	N-glycosylase/DNA lyase [EC:3.2.2.- 4.2.99.18]	-0.38	1.0E-246	7.3E-246	-0.42	yes
K08351	biotin/methionine sulfoxide reductase [EC:1.-.-.-]	0.38	1.0E-246	7.3E-246	0.25	
K08318	4-hydroxybutyrate dehydrogenase / sulfolactaldehyde 3-reductase [EC:1.1.1.61 1.1.1.373]	0.38	1.2E-246	8.8E-246	0.31	
K00437	[NiFe] hydrogenase large subunit [EC:1.12.2.1]	-0.38	1.3E-246	9.5E-246	-0.39	yes
K05973	poly(3-hydroxybutyrate) depolymerase [EC:3.1.1.75]	-0.38	1.6E-246	1.2E-245	-0.41	yes

Predictor	Description	Pearson's r	P	FDR-adjusted P	Spearman's rho	Associated with fractures (P<0.05)
K12139	hydrogenase-4 component D [EC:1.-.-.-]	0.38	2.1E-246	1.5E-245	0.29	
K02080	putative deaminase/isomerase [EC:3.5.99.-]	0.38	3.4E-246	2.5E-245	0.33	
K00404	cytochrome c oxidase cbb3-type subunit I [EC:7.1.1.9]	-0.38	3.7E-246	2.7E-245	-0.40	yes
K01266	D-aminopeptidase [EC:3.4.11.19]	-0.38	4.3E-246	3.1E-245	-0.41	yes
K09835	prolycopene isomerase [EC:5.2.1.13]	-0.38	8.3E-246	6.0E-245	-0.40	yes
K13777	geranyl-CoA carboxylase alpha subunit [EC:6.4.1.5]	-0.38	2.2E-245	1.6E-244	-0.40	yes
K07711	two-component system, NtrC family, sensor histidine kinase GlrK [EC:2.7.13.3]	0.38	2.9E-245	2.1E-244	0.23	
K08966	2-hydroxy-3-keto-5-methylthiopentenyl-1-phosphate phosphatase [EC:3.1.3.87]	-0.38	3.9E-245	2.8E-244	-0.40	yes
K00930	acetylglutamate kinase [EC:2.7.2.8]	-0.38	4.3E-245	3.1E-244	-0.40	
K16216	benzil reductase ((S)-benzoin forming) [EC:1.1.1.320]	-0.38	4.7E-245	3.3E-244	-0.41	yes
K01561	haloacetate dehalogenase [EC:3.8.1.3]	-0.38	5.3E-245	3.8E-244	-0.41	yes
K15736	(S)-2-hydroxyglutarate dehydrogenase [EC:1.1.5.13]	0.38	7.6E-245	5.4E-244	0.23	
K16066	3-hydroxy acid dehydrogenase / malonic semialdehyde reductase [EC:1.1.1.381 1.1.1.-]	0.38	7.7E-245	5.4E-244	0.23	
K01582	lysine decarboxylase [EC:4.1.1.18]	0.38	8.9E-245	6.3E-244	0.25	
K07679	two-component system, NarL family, sensor histidine kinase EvgS [EC:2.7.13.3]	0.38	2.0E-244	1.4E-243	0.25	
K07116	acyl-homoserine-lactone acylase [EC:3.5.1.97]	-0.38	2.1E-244	1.5E-243	-0.40	yes
K05303	O-methyltransferase [EC:2.1.1.-]	-0.38	2.2E-244	1.5E-243	-0.43	
K03829	putative acetyltransferase [EC:2.3.1.-]	0.38	2.4E-244	1.7E-243	0.23	
K05552	minimal PKS chain-length factor (CLF/KS beta) [EC:2.3.1.- 2.3.1.260 2.3.1.235]	-0.38	2.4E-244	1.7E-243	-0.41	
K10764	O-succinylhomoserine sulfhydrylase [EC:2.5.1.-]	-0.38	4.2E-244	2.9E-243	-0.40	yes
K05709	3-phenylpropionate/trans-cinnamate dioxygenase subunit beta [EC:1.14.12.19]	0.38	5.8E-244	4.0E-243	0.32	
K14080	[methyl-Co(III) methanol/glycine betaine-specific corrinoid protein]:coenzyme M methyltransferase [EC:2.1.1.246 2.1.1.377]	-0.38	7.7E-244	5.3E-243	-0.42	yes
K00153	S-(hydroxymethyl)mycothiol dehydrogenase [EC:1.1.1.306]	-0.38	7.9E-244	5.4E-243	-0.40	yes
K15632	23S rRNA (adenine-C8)-methyltransferase [EC:2.1.1.224]	-0.38	9.3E-244	6.4E-243	-0.40	yes
K02586	nitrogenase molybdenum-iron protein alpha chain [EC:1.18.6.1]	-0.38	1.3E-243	8.9E-243	-0.41	yes
K13778	geranyl-CoA carboxylase beta subunit [EC:6.4.1.5]	-0.38	1.3E-243	8.9E-243	-0.40	yes
K15739	D-alanine---(R)-lactate ligase [EC:6.1.2.1]	-0.38	1.7E-243	1.2E-242	-0.40	yes
K02803	N-acetylglucosamine PTS system EIIB component [EC:2.7.1.193]	-0.38	1.8E-243	1.2E-242	-0.40	yes
K08312	ADP-ribose diphosphatase [EC:3.6.1.-]	0.38	3.1E-243	2.1E-242	0.23	
K08092	3-dehydro-L-gulonate 2-dehydrogenase [EC:1.1.1.130]	0.38	4.3E-243	2.9E-242	0.22	
K01355	omptin [EC:3.4.23.49]	0.38	4.8E-243	3.2E-242	0.31	
K11336	3-vinyl bacteriochlorophyllide hydratase [EC:4.2.1.165]	-0.38	6.5E-243	4.4E-242	-0.40	yes
K07716	two-component system, cell cycle sensor histidine kinase PleC [EC:2.7.13.3]	-0.38	1.1E-242	7.4E-242	-0.40	yes
K07777	two-component system, NarL family, sensor histidine kinase DegS [EC:2.7.13.3]	-0.38	1.5E-242	1.0E-241	-0.38	yes
K05281	2'-hydroxyisoflavone reductase [EC:1.3.1.45]	-0.38	1.6E-242	1.1E-241	-0.42	yes
K11392	16S rRNA (cytosine1407-C5)-methyltransferase [EC:2.1.1.178]	0.38	2.9E-242	1.9E-241	0.24	
K10531	L-ornithine N5-monooxygenase [EC:1.14.13.195 1.14.13.196]	-0.38	3.8E-242	2.5E-241	-0.40	yes
K05541	tRNA-dihydrouridine synthase C [EC:1.-.-.-]	0.38	9.9E-242	6.6E-241	0.22	
K00252	glutaryl-CoA dehydrogenase [EC:1.3.8.6]	-0.38	1.1E-241	7.3E-241	-0.40	yes
K02668	two-component system, NtrC family, sensor histidine kinase PilS [EC:2.7.13.3]	-0.38	1.3E-241	8.6E-241	-0.40	yes
K00066	GDP-mannose 6-dehydrogenase [EC:1.1.1.132]	-0.38	1.7E-241	1.1E-240	-0.39	yes
K00906	isocitrate dehydrogenase kinase/phosphatase [EC:2.7.11.5 3.1.3.-]	0.38	1.9E-241	1.3E-240	0.23	

Predictor	Description	Pearson's r	P	FDR-adjusted P	Spearman's rho	Associated with fractures (P<0.05)
K03896	acetyl CoA:N6-hydroxylysine acetyl transferase [EC:2.3.1.102]	0.38	2.3E-241	1.5E-240	0.31	
K11261	formylmethanofuran dehydrogenase subunit E [EC:1.2.7.12]	-0.38	2.6E-241	1.7E-240	-0.41	yes
K11753	riboflavin kinase / FMN adenylyltransferase [EC:2.7.1.26 2.7.7.2]	-0.38	2.9E-241	1.9E-240	-0.37	
K00806	undecaprenyl diphosphate synthase [EC:2.5.1.31]	-0.38	7.5E-241	4.9E-240	-0.36	
K08691	malyl-CoA/(S)-citramalyl-CoA lyase [EC:4.1.3.24 4.1.3.25]	-0.38	1.1E-240	7.2E-240	-0.41	yes
K10551	D-allose transport system ATP-binding protein [EC:7.5.2.8]	0.38	1.3E-240	8.4E-240	0.27	
K13244	c-di-GMP-specific phosphodiesterase [EC:3.1.4.52]	0.38	1.3E-240	8.4E-240	0.32	
K05887	quinate/shikimate dehydrogenase [EC:1.1.1.282]	0.38	1.3E-240	8.4E-240	0.32	
K10670	glycine/sarcosine/betaine reductase complex component A [EC:1.21.4.2 1.21.4.3 1.21.4.4]	-0.38	1.4E-240	9.1E-240	-0.39	yes
K15534	beta-D-galactosyl-(1->4)-L-rhamnose phosphorylase [EC:2.4.1.247]	-0.38	1.8E-240	1.2E-239	-0.39	yes
K01308	g-D-glutamyl-meso-diaminopimelate peptidase [EC:3.4.19.11]	-0.38	1.8E-240	1.2E-239	-0.38	yes
K03181	chorismate lyase [EC:4.1.3.40]	0.38	2.0E-240	1.3E-239	0.22	
K03895	aerobactin synthase [EC:6.3.2.39]	0.38	3.1E-240	2.0E-239	0.30	
K01085	glucose-1-phosphatase [EC:3.1.3.10]	0.38	3.6E-240	2.3E-239	0.26	
K00200	formylmethanofuran dehydrogenase subunit A [EC:1.2.7.12]	-0.38	3.7E-240	2.4E-239	-0.41	yes
K01078	acid phosphatase [EC:3.1.3.2]	-0.38	4.9E-240	3.1E-239	-0.40	yes
K01602	ribulose-bisphosphate carboxylase small chain [EC:4.1.1.39]	-0.38	6.3E-240	4.0E-239	-0.40	yes
K01890	phenylalanyl-tRNA synthetase beta chain [EC:6.1.1.20]	-0.38	7.9E-240	5.0E-239	-0.37	
K05774	ribose 1,5-bisphosphokinase [EC:2.7.4.23]	0.38	8.7E-240	5.5E-239	0.22	
K15396	tRNA (cytidine32/uridine32-2'-O)-methyltransferase [EC:2.1.1.200]	0.38	1.9E-239	1.2E-238	0.22	
K01139	GTP diphosphokinase / guanosine-3',5'-bis(diphosphate) 3'-diphosphatase [EC:2.7.6.5 3.1.7.2]	0.38	2.0E-239	1.3E-238	0.23	
K02812	sorbose PTS system EIIA component [EC:2.7.1.206]	0.38	2.3E-239	1.4E-238	0.32	
K02813	sorbose PTS system EIIB component [EC:2.7.1.206]	0.38	2.3E-239	1.4E-238	0.32	
K16648	arabinofuranan 3-O-arabinosyltransferase [EC:2.4.2.-]	-0.38	2.7E-239	1.7E-238	-0.41	yes
K01484	succinylarginine dihydrolase [EC:3.5.3.23]	0.38	3.7E-239	2.3E-238	0.22	
K00809	deoxyhypusine synthase [EC:2.5.1.46]	-0.38	3.8E-239	2.4E-238	-0.42	yes
K00052	3-isopropylmalate dehydrogenase [EC:1.1.1.85]	-0.38	9.9E-239	6.2E-238	-0.36	
K16653	decaprenylphospho-beta-D-ribofuranose 2-oxidase [EC:1.1.98.3]	-0.38	3.2E-238	2.0E-237	-0.41	yes
K07654	two-component system, OmpR family, sensor histidine kinase MtrB [EC:2.7.13.3]	-0.38	6.4E-238	4.0E-237	-0.39	yes
K00220	cyclohexadieny/prephenate dehydrogenase [EC:1.3.1.43 1.3.1.12]	-0.38	8.7E-238	5.4E-237	-0.41	yes
K02278	prepilin peptidase CpaA [EC:3.4.23.43]	-0.38	1.3E-237	8.0E-237	-0.38	yes
K04566	lysyl-tRNA synthetase, class I [EC:6.1.1.6]	-0.38	1.3E-237	8.0E-237	-0.42	yes
K06020	energy-dependent translational throttle protein EttA	-0.38	2.4E-237	1.5E-236	-0.40	yes
K00090	glyoxylate/hydroxypyruvate/2-ketogluconate reductase [EC:1.1.1.79 1.1.1.81 1.1.1.215]	0.38	3.9E-237	2.4E-236	0.22	
K03894	N2-citryl-N6-acetyl-N6-hydroxylysine synthase [EC:6.3.2.38]	0.38	4.1E-237	2.5E-236	0.31	
K00557	tRNA (uracil-5-)-methyltransferase [EC:2.1.1.35]	0.38	4.1E-237	2.5E-236	0.22	
K07675	two-component system, NarL family, sensor histidine kinase UhpB [EC:2.7.13.3]	0.38	5.7E-237	3.5E-236	0.23	
K10125	two-component system, NtrC family, C4-dicarboxylate transport sensor histidine kinase DctB [EC:2.7.13.3]	-0.38	6.0E-237	3.7E-236	-0.40	yes
K11212	LPPG:FO 2-phospho-L-lactate transferase [EC:2.7.8.28]	-0.38	8.8E-237	5.4E-236	-0.42	yes
K00612	carbamoyltransferase [EC:2.1.3.-]	-0.38	1.4E-236	8.5E-236	-0.40	yes
K15778	phosphomannomutase / phosphoglucomutase [EC:5.4.2.8 5.4.2.2]	-0.38	1.6E-236	9.7E-236	-0.41	yes
K13607	cinnamoyl-CoA:phenyllactate CoA-transferase [EC:2.8.3.17]	-0.38	2.0E-236	1.2E-235	-0.40	yes

Predictor	Description	Pearson's r	P	FDR-adjusted P	Spearman's rho	Associated with fractures (P<0.05)
K16173	glutaryl-CoA dehydrogenase (non-decarboxylating) [EC:1.3.99.32]	-0.38	2.9E-236	1.7E-235	-0.41	yes
K01070	S-formylglutathione hydrolase [EC:3.1.2.12]	0.38	5.2E-236	3.1E-235	0.21	
K14128	F420-non-reducing hydrogenase small subunit [EC:1.12.99.- 1.8.98.5]	-0.38	5.5E-236	3.3E-235	-0.41	yes
K14335	alpha-1,6-mannosyltransferase [EC:2.4.1.-]	-0.38	6.5E-236	3.9E-235	-0.39	
K01952	phosphoribosylformylglycinamidine synthase [EC:6.3.5.3]	-0.38	7.3E-236	4.4E-235	-0.40	
K09023	aminoacrylate hydrolase [EC:3.5.1.-]	0.38	8.4E-236	5.0E-235	0.29	
K05714	2-hydroxy-6-oxonona-2,4-dienedioate hydrolase [EC:3.7.1.14]	0.38	1.7E-235	1.0E-234	0.25	
K05350	beta-glucosidase [EC:3.2.1.21]	-0.38	1.9E-235	1.1E-234	-0.37	yes
K12234	coenzyme F420-0:L-glutamate ligase / coenzyme F420-1:gamma-L-glutamate ligase [EC:6.3.2.31 6.3.2.34]	-0.38	3.6E-235	2.1E-234	-0.42	yes
K03716	spore photoproduct lyase [EC:4.1.99.14]	-0.38	5.4E-235	3.2E-234	-0.38	yes
K16649	rhamnopyranosyl-N-acetylglucosaminyl-diphospho-decaprenol beta-1,3/1,4-galactofuranosyltransferase [EC:2.4.1.287]	-0.38	6.0E-235	3.5E-234	-0.40	yes
K01535	H+-transporting ATPase [EC:7.1.2.1]	-0.38	8.8E-235	5.2E-234	-0.41	yes
K07677	two-component system, NarL family, capsular synthesis sensor histidine kinase RcsC [EC:2.7.13.3]	0.38	8.9E-235	5.2E-234	0.24	
K14287	methionine transaminase [EC:2.6.1.88]	0.38	9.3E-235	5.5E-234	0.21	
K13581	modification methylase [EC:2.1.1.72]	-0.38	9.6E-235	5.6E-234	-0.37	yes
K01252	bifunctional isochorismate lyase / aryl carrier protein [EC:3.3.2.1 6.3.2.14]	0.38	9.6E-235	5.6E-234	0.22	
K08302	tagatose 1,6-diphosphate aldolase GatY/KbaY [EC:4.1.2.40]	0.38	1.2E-234	7.0E-234	0.24	
K13522	bifunctional NMN adenylyltransferase/nudix hydrolase [EC:2.7.7.1 3.6.1.-]	-0.38	1.5E-234	8.8E-234	-0.40	yes
K05586	bidirectional [NiFe] hydrogenase diaphorase subunit [EC:7.1.1.2]	-0.38	1.7E-234	9.9E-234	-0.41	yes
K05588	bidirectional [NiFe] hydrogenase diaphorase subunit [EC:7.1.1.2]	-0.38	1.7E-234	9.9E-234	-0.41	yes
K09472	4-(gamma-glutamylamino)butanal dehydrogenase [EC:1.2.1.99]	0.38	3.1E-234	1.8E-233	0.22	
K00411	ubiquinol-cytochrome c reductase iron-sulfur subunit [EC:7.1.1.8]	-0.38	3.7E-234	2.1E-233	-0.40	yes
K07718	two-component system, sensor histidine kinase YesM [EC:2.7.13.3]	-0.37	1.3E-233	7.5E-233	-0.38	yes
K00197	acetyl-CoA decarbonylase/synthase, CODH/ACS complex subunit gamma [EC:2.1.1.245]	-0.37	1.4E-233	8.1E-233	-0.41	yes
K07647	two-component system, OmpR family, sensor histidine kinase TorS [EC:2.7.13.3]	0.37	2.4E-233	1.4E-232	0.29	
K00783	23S rRNA (pseudouridine1915-N3)-methyltransferase [EC:2.1.1.177]	-0.37	2.9E-233	1.7E-232	-0.38	yes
K03777	D-lactate dehydrogenase (quinone) [EC:1.1.5.12]	0.37	3.5E-233	2.0E-232	0.22	
K09759	nondiscriminating aspartyl-tRNA synthetase [EC:6.1.1.23]	-0.37	5.9E-233	3.4E-232	-0.38	yes
K01409	N6-L-threonylcarbamoyladenine synthase [EC:2.3.1.234]	-0.37	9.8E-233	5.6E-232	-0.37	
K04783	yersiniabactin salicyl-AMP ligase [EC:6.3.2.-]	0.37	1.2E-232	6.8E-232	0.26	
K06132	cardiolipin synthase C [EC:2.7.8.-]	0.37	1.7E-232	9.7E-232	0.22	
K08258	staphopain A [EC:3.4.22.48]	-0.37	2.2E-232	1.2E-231	-0.37	yes
K15358	enamidase [EC:3.5.2.18]	-0.37	2.3E-232	1.3E-231	-0.41	yes
K00194	acetyl-CoA decarbonylase/synthase, CODH/ACS complex subunit delta [EC:2.1.1.245]	-0.37	2.6E-232	1.5E-231	-0.41	yes
K00570	phosphatidylethanolamine/phosphatidyl-N-methylethanolamine N-methyltransferase [EC:2.1.1.17 2.1.1.71]	-0.37	2.8E-232	1.6E-231	-0.40	yes
K10856	acetone carboxylase, gamma subunit [EC:6.4.1.6]	-0.37	3.5E-232	2.0E-231	-0.41	yes
K00808	homospermidine synthase [EC:2.5.1.44]	-0.37	3.7E-232	2.1E-231	-0.40	yes
K16047	3-hydroxy-9,10-secoandrosta-1,3,5(10)-triene-9,17-dione monooxygenase [EC:1.14.14.12]	-0.37	4.3E-232	2.4E-231	-0.40	yes
K01301	N-acetylated-alpha-linked acidic dipeptidase [EC:3.4.17.21]	-0.37	4.3E-232	2.4E-231	-0.39	yes
K01655	homocitrate synthase [EC:2.3.3.14]	-0.37	1.4E-231	7.8E-231	-0.41	yes
K06175	tRNA pseudouridine65 synthase [EC:5.4.99.26]	0.37	1.4E-231	7.8E-231	0.21	
K07641	two-component system, OmpR family, sensor histidine kinase CreC [EC:2.7.13.3]	0.37	2.0E-231	1.1E-230	0.23	

Predictor	Description	Pearson's r	P	FDR-adjusted P	Spearman's rho	Associated with fractures (P<0.05)
K03862	vanillate monooxygenase [EC:1.14.13.82]	-0.37	2.2E-231	1.2E-230	-0.40	yes
K07710	two-component system, NtrC family, sensor histidine kinase AtoS [EC:2.7.13.3]	0.37	2.6E-231	1.4E-230	0.22	
K03276	UDP-glucose/galactose:(glucosyl)LPS alpha-1,2-glucosyl/galactosyltransferase [EC:2.4.1.-]	0.37	4.0E-231	2.2E-230	0.32	
K15916	glucose/mannose-6-phosphate isomerase [EC:5.3.1.9 5.3.1.8]	-0.37	4.4E-231	2.4E-230	-0.42	yes
K15733	dye decolorizing peroxidase [EC:1.11.1.19]	-0.37	5.9E-231	3.3E-230	-0.39	yes
K00045	mannitol 2-dehydrogenase [EC:1.1.1.67]	-0.37	6.3E-231	3.5E-230	-0.39	yes
K00803	alkyldihydroxyacetonephosphate synthase [EC:2.5.1.26]	-0.37	9.3E-231	5.1E-230	-0.39	yes
K06928	nucleoside-triphosphatase [EC:3.6.1.15]	-0.37	2.5E-230	1.4E-229	-0.38	yes
K00932	propionate kinase [EC:2.7.2.15]	0.37	5.5E-230	3.0E-229	0.32	
K00510	heme oxygenase 1 [EC:1.14.14.18]	-0.37	5.7E-230	3.1E-229	-0.39	yes
K00395	adenylylsulfate reductase, subunit B [EC:1.8.99.2]	-0.37	6.1E-230	3.3E-229	-0.43	
K10111	multiple sugar transport system ATP-binding protein [EC:7.5.2.-]	0.37	1.3E-229	7.1E-229	0.21	
K00448	protocatechuate 3,4-dioxygenase, alpha subunit [EC:1.13.11.3]	-0.37	1.5E-229	8.2E-229	-0.40	yes
K00477	phytanoyl-CoA hydroxylase [EC:1.14.11.18]	-0.37	1.9E-229	1.0E-228	-0.40	yes
K06445	acyl-CoA dehydrogenase [EC:1.3.99.-]	0.37	2.9E-229	1.6E-228	0.21	
K13063	2-amino-4-deoxychorismate synthase [EC:2.6.1.86]	-0.37	3.4E-229	1.8E-228	-0.40	yes
K14941	2-phospho-L-lactate/phosphoenolpyruvate guanylyltransferase [EC:2.7.7.68 2.7.7.105]	-0.37	6.8E-229	3.7E-228	-0.41	yes
K08321	3-hydroxy-5-phosphonooxypentane-2,4-dione thiolase [EC:2.3.1.245]	0.37	7.1E-229	3.8E-228	0.20	
K09699	2-oxoisovalerate dehydrogenase E2 component (dihydrolipoyl transacylase) [EC:2.3.1.168]	-0.37	1.4E-228	7.5E-228	-0.39	yes
K07645	two-component system, OmpR family, sensor histidine kinase QseC [EC:2.7.13.3]	0.37	1.5E-228	8.1E-228	0.20	
K00608	aspartate carbamoyltransferase [EC:2.1.3.2]	-0.37	2.2E-228	1.2E-227	-0.41	yes
K00449	protocatechuate 3,4-dioxygenase, beta subunit [EC:1.13.11.3]	-0.37	2.8E-228	1.5E-227	-0.40	yes
K15862	cytochrome c oxidase cbb3-type subunit I/II [EC:7.1.1.9]	-0.37	3.2E-228	1.7E-227	-0.38	
K00499	choline monooxygenase [EC:1.14.15.7]	-0.37	4.9E-228	2.6E-227	-0.42	yes
K13832	3-dehydroquinate dehydratase / shikimate dehydrogenase [EC:4.2.1.10 1.1.1.25]	-0.37	6.3E-228	3.4E-227	-0.42	yes
K00098	L-idonate 5-dehydrogenase [EC:1.1.1.264]	0.37	6.8E-228	3.6E-227	0.23	
K04034	anaerobic magnesium-protoporphyrin IX monomethyl ester cyclase [EC:1.21.98.3]	-0.37	9.4E-228	5.0E-227	-0.41	yes
K01273	membrane dipeptidase [EC:3.4.13.19]	-0.37	1.5E-227	8.0E-227	-0.38	yes
K03774	FKBP-type peptidyl-prolyl cis-trans isomerase SlpA [EC:5.2.1.8]	0.37	2.2E-227	1.2E-226	0.20	
K15230	ATP-citrate lyase alpha-subunit [EC:2.3.3.8]	-0.37	2.4E-227	1.3E-226	-0.41	yes
K15231	ATP-citrate lyase beta-subunit [EC:2.3.3.8]	-0.37	2.4E-227	1.3E-226	-0.41	yes
K00150	glyceraldehyde-3-phosphate dehydrogenase (NAD(P)) [EC:1.2.1.59]	-0.37	2.9E-227	1.5E-226	-0.41	yes
K00360	assimilatory nitrate reductase electron transfer subunit [EC:1.7.99.-]	-0.37	5.8E-227	3.0E-226	-0.41	yes
K12992	O-antigen biosynthesis alpha-1,3-rhamnosyltransferase [EC:2.4.1.377]	-0.37	6.1E-227	3.2E-226	-0.37	yes
K14126	F420-non-reducing hydrogenase large subunit [EC:1.12.99.- 1.8.98.5]	-0.37	7.3E-227	3.8E-226	-0.41	yes
K00604	methionyl-tRNA formyltransferase [EC:2.1.2.9]	-0.37	7.6E-227	4.0E-226	-0.36	
K00927	phosphoglycerate kinase [EC:2.7.2.3]	-0.37	1.1E-226	5.7E-226	-0.41	
K06221	2,5-diketo-D-gluconate reductase A [EC:1.1.1.346]	0.37	1.4E-226	7.3E-226	0.22	
K13481	xanthine dehydrogenase small subunit [EC:1.17.1.4]	-0.37	2.3E-226	1.2E-225	-0.40	yes
K07535	2-hydroxycyclohexanecarboxyl-CoA dehydrogenase [EC:1.1.1.-]	-0.37	2.5E-226	1.3E-225	-0.41	yes
K10747	DNA ligase 1 [EC:6.5.1.1 6.5.1.6 6.5.1.7]	-0.37	3.0E-226	1.6E-225	-0.41	yes
K09471	gamma-glutamylputrescine oxidase [EC:1.4.3.-]	0.37	3.5E-226	1.8E-225	0.20	

Predictor	Description	Pearson's r	P	FDR-adjusted P	Spearman's rho	Associated with fractures (P<0.05)
K16370	6-phosphofructokinase 2 [EC:2.7.1.11]	0.37	4.8E-226	2.5E-225	0.21	
K02189	cobalt-precorrin 5A hydrolase [EC:3.7.1.12]	-0.37	5.6E-226	2.9E-225	-0.39	
K11216	autoinducer-2 kinase [EC:2.7.1.189]	0.37	5.8E-226	3.0E-225	0.21	
K01224	arabinogalactan endo-1,4-beta-galactosidase [EC:3.2.1.89]	-0.37	5.8E-226	3.0E-225	-0.37	yes
K16164	acylpyruvate hydrolase [EC:3.7.1.5]	-0.37	5.9E-226	3.0E-225	-0.41	yes
K01499	methenyltetrahydromethanopterin cyclohydrolase [EC:3.5.4.27]	-0.37	9.7E-226	5.0E-225	-0.41	yes
K11931	poly-beta-1,6-N-acetyl-D-glucosamine N-deacetylase [EC:3.5.1.-]	0.37	1.0E-225	5.1E-225	0.22	
K12972	glyoxylate/hydroxypyruvate reductase [EC:1.1.1.79 1.1.1.81]	0.37	1.2E-225	6.1E-225	0.20	
K09845	1-hydroxycarotenoid 3,4-desaturase [EC:1.3.99.27]	-0.37	1.5E-225	7.6E-225	-0.39	yes
K05587	bidirectional [NiFe] hydrogenase diaphorase subunit [EC:7.1.1.2]	-0.37	2.6E-225	1.3E-224	-0.40	yes
K03153	glycine oxidase [EC:1.4.3.19]	-0.37	6.3E-225	3.2E-224	-0.39	yes
K06718	L-2,4-diaminobutyric acid acetyltransferase [EC:2.3.1.178]	-0.37	1.0E-224	5.1E-224	-0.39	yes
K00529	3-phenylpropionate/trans-cinnamate dioxygenase ferredoxin reductase component [EC:1.18.1.3]	0.37	1.3E-224	6.6E-224	0.21	
K03082	NA	0.37	1.3E-224	6.6E-224	0.21	
K12256	putrescine---pyruvate transaminase [EC:2.6.1.113]	-0.37	1.4E-224	7.1E-224	-0.39	yes
K00376	nitrous-oxide reductase [EC:1.7.2.4]	-0.37	1.5E-224	7.6E-224	-0.38	yes
K13243	c-di-GMP-specific phosphodiesterase [EC:3.1.4.52]	0.37	1.8E-224	9.1E-224	0.31	
K03433	proteasome beta subunit [EC:3.4.25.1]	-0.37	1.9E-224	9.5E-224	-0.41	yes
K15520	mycothiol synthase [EC:2.3.1.189]	-0.37	2.8E-224	1.4E-223	-0.39	yes
K14339	alpha-1,6-mannosyltransferase [EC:2.4.1.-]	-0.37	3.0E-224	1.5E-223	-0.40	yes
K02594	homocitrate synthase NifV [EC:2.3.3.14]	-0.37	3.2E-224	1.6E-223	-0.41	yes
K00713	UDP-glucose:(glucosyl)LPS alpha-1,2-glucosyltransferase [EC:2.4.1.-]	0.37	6.0E-224	3.0E-223	0.32	
K14337	alpha-1,6-mannosyltransferase [EC:2.4.1.-]	-0.37	6.6E-224	3.3E-223	-0.39	yes
K05365	penicillin-binding protein 1B [EC:2.4.1.129 3.4.16.4]	0.37	7.8E-224	3.9E-223	0.21	
K00457	4-hydroxyphenylpyruvate dioxygenase [EC:1.13.11.27]	-0.37	9.0E-224	4.5E-223	-0.40	yes
K12448	UDP-arabinose 4-epimerase [EC:5.1.3.5]	-0.37	1.4E-223	6.9E-223	-0.40	
K08688	creatinase [EC:3.5.3.3]	-0.37	1.5E-223	7.4E-223	-0.39	yes
K00186	2-oxoisovalerate ferredoxin oxidoreductase alpha subunit [EC:1.2.7.7]	-0.37	1.7E-223	8.4E-223	-0.41	yes
K04844	hypothetical glycosyl hydrolase [EC:3.2.1.-]	0.37	5.8E-223	2.9E-222	0.32	
K07314	serine/threonine protein phosphatase 2 [EC:3.1.3.16]	0.37	5.8E-223	2.9E-222	0.32	
K06015	N-acyl-D-amino-acid deacylase [EC:3.5.1.81]	-0.37	6.1E-223	3.0E-222	-0.39	yes
K05898	3-oxosteroid 1-dehydrogenase [EC:1.3.99.4]	-0.37	7.1E-223	3.5E-222	-0.40	yes
K01725	cyanate lyase [EC:4.2.1.104]	0.37	7.6E-223	3.7E-222	0.21	
K05824	homoisocitrate dehydrogenase [EC:1.1.1.87]	-0.37	1.1E-222	5.4E-222	-0.41	yes
K03432	proteasome alpha subunit [EC:3.4.25.1]	-0.37	1.9E-222	9.3E-222	-0.41	yes
K14138	acetyl-CoA synthase [EC:2.3.1.169]	-0.37	2.8E-222	1.4E-221	-0.40	yes
K05715	2-phosphoglycerate kinase [EC:2.7.2.16]	-0.37	3.0E-222	1.5E-221	-0.40	yes
K00073	ureidoglycolate dehydrogenase (NAD+) [EC:1.1.1.350]	0.37	4.3E-222	2.1E-221	0.23	
K03275	UDP-glucose:(glucosyl)LPS alpha-1,3-glucosyltransferase [EC:2.4.1.-]	0.37	6.9E-222	3.3E-221	0.28	
K03897	lysine N6-hydroxylase [EC:1.14.13.59]	0.37	8.3E-222	4.0E-221	0.20	
K00962	polyribonucleotide nucleotidyltransferase [EC:2.7.7.8]	-0.37	8.4E-222	4.1E-221	-0.32	
K01578	malonyl-CoA decarboxylase [EC:4.1.1.9]	-0.37	9.9E-222	4.8E-221	-0.40	yes

Predictor	Description	Pearson's r	P	FDR-adjusted P	Spearman's rho	Associated with fractures (P<0.05)
K02297	cytochrome o ubiquinol oxidase subunit II [EC:7.1.1.3]	0.37	1.4E-221	6.7E-221	0.20	
K00631	glycerol-3-phosphate O-acyltransferase [EC:2.3.1.15]	0.37	2.6E-221	1.3E-220	0.20	
K05934	precorrin-3B C17-methyltransferase / cobalt-factor III methyltransferase [EC:2.1.1.131 2.1.1.272]	-0.37	5.4E-221	2.6E-220	-0.39	
K04090	indolepyruvate ferredoxin oxidoreductase [EC:1.2.7.8]	-0.37	5.6E-221	2.7E-220	-0.40	yes
K05591	ATP-dependent RNA helicase DbpA [EC:3.6.4.13]	0.37	5.7E-221	2.7E-220	0.20	
K11434	type I protein arginine methyltransferase [EC:2.1.1.319]	-0.36	9.2E-221	4.4E-220	-0.40	
K03918	L-lysine 6-transaminase [EC:2.6.1.36]	-0.36	9.4E-221	4.5E-220	-0.39	
K12985	(galactosyl)LPS 1,2-glucosyltransferase [EC:2.4.1.-]	0.36	1.3E-220	6.2E-220	0.32	
K12983	UDP-glucose:(glucosyl)LPS beta-1,3-glucosyltransferase [EC:2.4.1.-]	0.36	1.3E-220	6.2E-220	0.32	
K00505	tyrosinase [EC:1.14.18.1]	-0.36	1.8E-220	8.6E-220	-0.40	yes
K16150	glycogen synthase [EC:2.4.1.11]	-0.36	2.7E-220	1.3E-219	-0.41	yes
K05555	cyclase [EC:4.-.-.-]	-0.36	2.8E-220	1.3E-219	-0.40	
K01727	hyaluronate lyase [EC:4.2.2.1]	-0.36	3.2E-220	1.5E-219	-0.37	yes
K00394	adenylylsulfate reductase, subunit A [EC:1.8.99.2]	-0.36	3.8E-220	1.8E-219	-0.42	
K01455	formamidase [EC:3.5.1.49]	-0.36	4.0E-220	1.9E-219	-0.39	yes
K06023	HPr kinase/phosphorylase [EC:2.7.11.- 2.7.4.-]	-0.36	4.5E-220	2.1E-219	-0.37	
K00863	triose/dihydroxyacetone kinase / FAD-AMP lyase (cyclizing) [EC:2.7.1.28 2.7.1.29 4.6.1.15]	-0.36	6.8E-220	3.2E-219	-0.39	yes
K01616	multifunctional 2-oxoglutarate metabolism enzyme [EC:2.2.1.5 4.1.1.71 1.2.4.2 2.3.1.61]	-0.36	7.4E-220	3.5E-219	-0.40	
K00441	coenzyme F420 hydrogenase subunit beta [EC:1.12.98.1]	-0.36	1.1E-219	5.2E-219	-0.41	yes
K03852	sulfoacetaldehyde acetyltransferase [EC:2.3.3.15]	-0.36	2.3E-219	1.1E-218	-0.38	yes
K02298	cytochrome o ubiquinol oxidase subunit I [EC:7.1.1.3]	0.36	5.8E-219	2.7E-218	0.19	
K00368	nitrite reductase (NO-forming) [EC:1.7.2.1]	-0.36	9.6E-219	4.5E-218	-0.39	yes
K07533	foldase protein PrsA [EC:5.2.1.8]	-0.36	1.3E-218	6.1E-218	-0.37	
K01822	steroid Delta-isomerase [EC:5.3.3.1]	-0.36	3.9E-218	1.8E-217	-0.39	
K15984	16S rRNA (guanine1516-N2)-methyltransferase [EC:2.1.1.242]	0.36	1.3E-217	6.0E-217	0.20	
K03652	DNA-3-methyladenine glycosylase [EC:3.2.2.21]	-0.36	1.3E-217	6.0E-217	-0.39	yes
K05602	histidinol-phosphatase [EC:3.1.3.15]	-0.36	1.3E-217	6.0E-217	-0.39	yes
K15461	tRNA 5-methylaminomethyl-2-thiouridine biosynthesis bifunctional protein [EC:2.1.1.61 1.5.-.-]	0.36	3.4E-217	1.6E-216	0.19	
K08484	phosphotransferase system, enzyme I, PtsP [EC:2.7.3.9]	0.36	3.5E-217	1.6E-216	0.19	
K00769	xanthine phosphoribosyltransferase [EC:2.4.2.22]	0.36	6.9E-217	3.2E-216	0.20	
K07031	D-glycero-alpha-D-manno-heptose-7-phosphate kinase [EC:2.7.1.168]	-0.36	1.1E-216	5.1E-216	-0.37	yes
K06137	pyrroloquinoline-quinone synthase [EC:1.3.3.11]	-0.36	1.2E-216	5.5E-216	-0.39	yes
K14187	chorismate mutase / prephenate dehydrogenase [EC:5.4.99.5 1.3.1.12]	0.36	3.4E-216	1.6E-215	0.24	
K10533	limonene-1,2-epoxide hydrolase [EC:3.3.2.8]	-0.36	6.0E-216	2.7E-215	-0.38	
K07151	dolichyl-diphosphooligosaccharide---protein glycosyltransferase [EC:2.4.99.18]	-0.36	6.7E-216	3.1E-215	-0.40	yes
K01208	cyclomaltodextrinase / maltogenic alpha-amylase / neopullulanase [EC:3.2.1.54 3.2.1.133 3.2.1.135]	-0.36	1.2E-215	5.5E-215	-0.37	yes
K01584	arginine decarboxylase [EC:4.1.1.19]	0.36	1.4E-215	6.4E-215	0.19	
K03919	DNA oxidative demethylase [EC:1.14.11.33]	0.36	3.6E-215	1.6E-214	0.20	
K00021	hydroxymethylglutaryl-CoA reductase (NADPH) [EC:1.1.1.34]	-0.36	3.8E-215	1.7E-214	-0.40	yes
K05539	tRNA-dihydrouridine synthase A [EC:1.-.-.-]	0.36	3.8E-215	1.7E-214	0.19	
K00673	arginine N-succinyltransferase [EC:2.3.1.109]	0.36	7.0E-215	3.2E-214	0.19	
K11383	two-component system, NtrC family, sensor histidine kinase KinB [EC:2.7.13.3]	-0.36	8.5E-215	3.8E-214	-0.38	yes

Predictor	Description	Pearson's r	P	FDR-adjusted P	Spearman's rho	Associated with fractures (P<0.05)
K00855	phosphoribulokinase [EC:2.7.1.19]	0.36	8.7E-215	3.9E-214	0.20	
K01622	fructose 1,6-bisphosphate aldolase/phosphatase [EC:4.1.2.13 3.1.3.11]	-0.36	9.6E-215	4.3E-214	-0.40	yes
K00620	glutamate N-acetyltransferase / amino-acid N-acetyltransferase [EC:2.3.1.35 2.3.1.1]	-0.36	1.2E-214	5.4E-214	-0.37	
K00002	alcohol dehydrogenase (NADP+) [EC:1.1.1.2]	-0.36	1.2E-214	5.4E-214	-0.40	yes
K06968	23S rRNA (cytidine2498-2'-O)-methyltransferase [EC:2.1.1.186]	0.36	1.3E-214	5.8E-214	0.19	
K05551	minimal PKS ketosynthase (KS/KS alpha) [EC:2.3.1.- 2.3.1.260 2.3.1.235]	-0.36	1.4E-214	6.3E-214	-0.40	
K01174	micrococcal nuclease [EC:3.1.31.1]	-0.36	1.9E-214	8.5E-214	-0.37	
K07823	3-oxoadipyl-CoA thiolase [EC:2.3.1.174]	-0.36	2.0E-214	8.9E-214	-0.39	yes
K00662	aminoglycoside 3-N-acetyltransferase [EC:2.3.1.81]	-0.36	2.0E-214	8.9E-214	-0.37	yes
K14986	two-component system, LuxR family, sensor kinase FixL [EC:2.7.13.3]	-0.36	2.4E-214	1.1E-213	-0.40	yes
K00125	formate dehydrogenase (coenzyme F420) beta subunit [EC:1.17.98.3 1.8.98.6]	-0.36	2.9E-214	1.3E-213	-0.40	yes
K11693	peptidoglycan pentaglycine glycine transferase (the first glycine) [EC:2.3.2.16]	-0.36	3.4E-214	1.5E-213	-0.38	yes
K15521	D-inositol-3-phosphate glycosyltransferase [EC:2.4.1.250]	-0.36	4.3E-214	1.9E-213	-0.38	yes
K11646	3-dehydroquinate synthase II [EC:1.4.1.24]	-0.36	5.2E-214	2.3E-213	-0.40	yes
K12989	mannosyltransferase [EC:2.4.1.-]	-0.36	6.1E-214	2.7E-213	-0.39	yes
K01928	UDP-N-acetylmuramoyl-L-alanyl-D-glutamate--2,6-diaminopimelate ligase [EC:6.3.2.13]	-0.36	8.4E-214	3.7E-213	-0.36	
K03279	UDP-glucose:(galactosyl)LPS alpha-1,2-glucosyltransferase [EC:2.4.1.58]	0.36	9.6E-214	4.2E-213	0.31	
K00508	linoleoyl-CoA desaturase [EC:1.14.19.3]	-0.36	1.3E-213	5.7E-213	-0.39	yes
K00624	carnitine O-acetyltransferase [EC:2.3.1.7]	-0.36	3.3E-213	1.5E-212	-0.40	yes
K14667	minimal PKS ketosynthase (KS/KS alpha) [EC:2.3.1.-]	-0.36	3.4E-213	1.5E-212	-0.40	
K10824	nickel transport system ATP-binding protein [EC:7.2.2.11]	0.36	3.7E-213	1.6E-212	0.18	
K00952	nicotinamide-nucleotide adenylyltransferase [EC:2.7.7.1]	-0.36	4.6E-213	2.0E-212	-0.40	yes
K00202	formylmethanofuran dehydrogenase subunit C [EC:1.2.7.12]	-0.36	6.2E-213	2.7E-212	-0.40	yes
K14733	limonene 1,2-monooxygenase [EC:1.14.13.107]	-0.36	9.2E-213	4.0E-212	-0.38	yes
K15509	sulfopropanediol 3-dehydrogenase [EC:1.1.1.308]	-0.36	9.2E-213	4.0E-212	-0.41	yes
K00822	beta-alanine--pyruvate transaminase [EC:2.6.1.18]	-0.36	1.0E-212	4.4E-212	-0.39	yes
K10907	aminotransferase [EC:2.6.1.-]	-0.36	4.4E-212	1.9E-211	-0.36	
K00169	pyruvate ferredoxin oxidoreductase alpha subunit [EC:1.2.7.1]	-0.36	5.1E-212	2.2E-211	-0.38	
K15525	N-acetyl-1-D-myo-inositol-2-amino-2-deoxy-alpha-D-glucopyranoside deacetylase [EC:3.5.1.103]	-0.36	1.3E-211	5.6E-211	-0.38	yes
K05889	polyvinyl alcohol dehydrogenase (cytochrome) [EC:1.1.2.6]	-0.36	1.5E-211	6.5E-211	-0.40	yes
K00154	coniferyl-aldehyde dehydrogenase [EC:1.2.1.68]	-0.36	2.2E-211	9.5E-211	-0.39	yes
K02844	UDP-glucose:(heptosyl)LPS alpha-1,3-glucosyltransferase [EC:2.4.1.-]	0.36	3.3E-211	1.4E-210	0.19	
K07508	acetyl-CoA acyltransferase 2 [EC:2.3.1.16]	-0.36	3.4E-211	1.5E-210	-0.40	yes
K14660	nodulation protein E [EC:2.3.1.-]	-0.36	3.5E-211	1.5E-210	-0.40	yes
K03795	sirohydrochlorin cobaltochelatase [EC:4.99.1.3]	-0.36	5.7E-211	2.4E-210	-0.41	yes
K10942	two-component system, sensor histidine kinase FlrB [EC:2.7.13.3]	-0.36	1.5E-210	6.4E-210	-0.38	yes
K00372	assimilatory nitrate reductase catalytic subunit [EC:1.7.99.-]	-0.36	1.7E-210	7.3E-210	-0.41	yes
K04116	cyclohexanecarboxylate-CoA ligase [EC:6.2.1.-]	-0.36	2.1E-210	9.0E-210	-0.41	yes
K01583	arginine decarboxylase [EC:4.1.1.19]	-0.36	7.2E-210	3.1E-209	-0.41	
K00514	zeta-carotene desaturase [EC:1.3.5.6]	-0.36	8.6E-210	3.7E-209	-0.39	yes
K13000	mannosyltransferase [EC:2.4.1.-]	0.36	1.4E-209	6.0E-209	0.28	
K03177	tRNA pseudouridine55 synthase [EC:5.4.99.25]	-0.36	1.6E-209	6.8E-209	-0.33	

Predictor	Description	Pearson's r	P	FDR-adjusted P	Spearman's rho	Associated with fractures (P<0.05)
K01141	exodeoxyribonuclease I [EC:3.1.11.1]	0.36	1.7E-209	7.2E-209	0.18	
K01690	phosphogluconate dehydratase [EC:4.2.1.12]	0.36	2.0E-209	8.5E-209	0.18	
K01693	imidazoleglycerol-phosphate dehydratase [EC:4.2.1.19]	-0.36	2.3E-209	9.7E-209	-0.36	
K11357	two-component system, cell cycle sensor histidine kinase DivJ [EC:2.7.13.3]	-0.36	2.8E-209	1.2E-208	-0.40	yes
K10231	kojibiose phosphorylase [EC:2.4.1.230]	-0.36	2.9E-209	1.2E-208	-0.36	yes
K03831	molybdopterin adenylyltransferase [EC:2.7.7.75]	0.36	3.6E-209	1.5E-208	0.19	
K03732	ATP-dependent RNA helicase RhlB [EC:3.6.4.13]	0.36	3.9E-209	1.6E-208	0.19	
K07678	two-component system, NarL family, sensor histidine kinase BarA [EC:2.7.13.3]	0.36	8.8E-209	3.7E-208	0.19	
K10984	galactosamine PTS system EIIB component [EC:2.7.1.-]	0.36	9.1E-209	3.8E-208	0.19	
K01299	carboxypeptidase Taq [EC:3.4.17.19]	-0.36	1.3E-208	5.5E-208	-0.37	yes
K11528	UDP-N-acetylglucosamine pyrophosphorylase [EC:2.7.7.23]	-0.36	1.4E-208	5.9E-208	-0.39	yes
K03167	DNA topoisomerase VI subunit B [EC:5.6.2.2]	-0.36	2.3E-208	9.6E-208	-0.41	yes
K10539	L-arabinose transport system ATP-binding protein [EC:7.5.2.12]	0.35	3.1E-208	1.3E-207	0.19	
K07638	two-component system, OmpR family, osmolarity sensor histidine kinase EnvZ [EC:2.7.13.3]	0.35	3.6E-208	1.5E-207	0.19	
K01525	bis(5'-nucleosyl)-tetraphosphatase (symmetrical) [EC:3.6.1.41]	0.35	4.4E-208	1.8E-207	0.18	
K00122	formate dehydrogenase [EC:1.17.1.9]	-0.35	5.3E-208	2.2E-207	-0.38	yes
K00320	5,10-methylenetetrahydromethanopterin reductase [EC:1.5.98.2]	-0.35	8.7E-208	3.6E-207	-0.40	yes
K07709	two-component system, NtrC family, sensor histidine kinase HydH [EC:2.7.13.3]	0.35	9.1E-208	3.8E-207	0.20	
K00805	heptaprenyl diphosphate synthase component 1 [EC:2.5.1.30]	-0.35	1.4E-207	5.8E-207	-0.36	
K13686	galactan 5-O-arabinofuranosyltransferase [EC:2.4.2.46]	-0.35	1.6E-207	6.6E-207	-0.39	
K01716	3-hydroxyacyl-[acyl-carrier protein] dehydratase / trans-2-decenoyl-[acyl-carrier protein] isomerase [EC:4.2.1.59 5.3.3.14]	0.35	2.8E-207	1.2E-206	0.18	
K02293	15-cis-phytoene desaturase [EC:1.3.5.5]	-0.35	3.4E-207	1.4E-206	-0.39	
K12979	beta-hydroxylase [EC:1.14.11.-]	-0.35	4.3E-207	1.8E-206	-0.38	yes
K00532	ferredoxin hydrogenase [EC:1.12.7.2]	-0.35	4.8E-207	2.0E-206	-0.41	yes
K11691	two-component system, CitB family, sensor histidine kinase DctS [EC:2.7.13.3]	-0.35	5.3E-207	2.2E-206	-0.39	yes
K02464	general secretion pathway protein O [EC:3.4.23.43 2.1.1.-]	0.35	6.6E-207	2.7E-206	0.28	
K01233	chitosanase [EC:3.2.1.132]	-0.35	2.6E-206	1.1E-205	-0.35	yes
K15513	benzoyl-CoA-dihydrodiol lyase [EC:4.1.2.44]	-0.35	7.1E-206	2.9E-205	-0.38	yes
K00700	1,4-alpha-glucan branching enzyme [EC:2.4.1.18]	-0.35	9.5E-206	3.9E-205	-0.37	yes
K00672	formylmethanofuran--tetrahydromethanopterin N-formyltransferase [EC:2.3.1.101]	-0.35	1.9E-205	7.8E-205	-0.39	yes
K13075	N-acyl homoserine lactone hydrolase [EC:3.1.1.81]	-0.35	2.0E-205	8.2E-205	-0.39	yes
K00322	NAD(P) transhydrogenase [EC:1.6.1.1]	0.35	2.2E-205	9.0E-205	0.18	
K13671	alpha-1,2-mannosyltransferase [EC:2.4.1.-]	-0.35	2.3E-205	9.4E-205	-0.37	yes
K00187	2-oxoisovalerate ferredoxin oxidoreductase beta subunit [EC:1.2.7.7]	-0.35	3.7E-205	1.5E-204	-0.39	yes
K04794	peptidyl-tRNA hydrolase, PTH2 family [EC:3.1.1.29]	-0.35	4.1E-205	1.7E-204	-0.39	yes
K15531	oligosaccharide reducing-end xylanase [EC:3.2.1.156]	-0.35	4.4E-205	1.8E-204	-0.34	yes
K12349	neutral ceramidase [EC:3.5.1.23]	-0.35	4.7E-205	1.9E-204	-0.37	yes
K13587	two-component system, cell cycle sensor histidine kinase and response regulator CckA [EC:2.7.13.3]	-0.35	4.8E-205	1.9E-204	-0.39	yes
K04480	methanol---5-hydroxybenzimidazolylcobamide Co-methyltransferase [EC:2.1.1.90]	-0.35	5.8E-205	2.3E-204	-0.39	yes
K02588	nitrogenase iron protein NifH	-0.35	1.6E-204	6.4E-204	-0.40	
K00076	7-alpha-hydroxysteroid dehydrogenase [EC:1.1.1.159]	0.35	1.9E-204	7.6E-204	0.22	
K02299	cytochrome o ubiquinol oxidase subunit III	0.35	2.1E-204	8.4E-204	0.18	

Predictor	Description	Pearson's r	P	FDR-adjusted P	Spearman's rho	Associated with fractures (P<0.05)
K15587	nickel transport system ATP-binding protein [EC:7.2.2.11]	0.35	4.3E-204	1.7E-203	0.17	
K04073	acetaldehyde dehydrogenase [EC:1.2.1.10]	0.35	7.7E-204	3.1E-203	0.18	
K13530	AraC family transcriptional regulator, regulatory protein of adaptative response / methylphosphotriester-DNA alkyltransferase methyltransferase [EC:2.1.1.-]	-0.35	9.3E-204	3.7E-203	-0.35	yes
K13491	two-component system, chemotaxis family, response regulator WspF [EC:3.1.1.61]	-0.35	1.1E-203	4.4E-203	-0.39	yes
K06180	23S rRNA pseudouridine1911/1915/1917 synthase [EC:5.4.99.23]	-0.35	1.4E-203	5.6E-203	-0.28	
K03166	DNA topoisomerase VI subunit A [EC:5.6.2.2]	-0.35	2.2E-203	8.8E-203	-0.39	yes
K01959	pyruvate carboxylase subunit A [EC:6.4.1.1]	-0.35	2.5E-203	1.0E-202	-0.39	yes
K00216	2,3-dihydro-2,3-dihydroxybenzoate dehydrogenase [EC:1.3.1.28]	0.35	2.6E-203	1.0E-202	0.19	
K09693	teichoic acid transport system ATP-binding protein [EC:7.5.2.4]	-0.35	3.2E-203	1.3E-202	-0.35	
K10220	4-oxalmesaconate hydratase [EC:4.2.1.83]	-0.35	3.8E-203	1.5E-202	-0.39	yes
K04781	salicylate synthetase [EC:5.4.4.2 4.2.99.21]	0.35	4.8E-203	1.9E-202	0.20	
K15429	tRNA (guanine37-N1)-methyltransferase [EC:2.1.1.228]	-0.35	7.0E-203	2.8E-202	-0.39	yes
K03825	L-phenylalanine/L-methionine N-acetyltransferase [EC:2.3.1.53 2.3.1.-]	0.35	7.5E-203	3.0E-202	0.19	
K03583	exodeoxyribonuclease V gamma subunit [EC:3.1.11.5]	0.35	8.4E-203	3.3E-202	0.17	
K02478	two-component system, LytTR family, sensor kinase [EC:2.7.13.3]	0.35	1.4E-202	5.5E-202	0.17	
K09846	demethylspheroidene O-methyltransferase [EC:2.1.1.210]	-0.35	6.3E-202	2.5E-201	-0.38	yes
K00980	glycerol-3-phosphate cytidylyltransferase [EC:2.7.7.39]	-0.35	1.1E-201	4.3E-201	-0.38	yes
K00459	nitronate monooxygenase [EC:1.13.12.16]	-0.35	1.3E-201	5.1E-201	-0.39	yes
K06982	pantoate kinase [EC:2.7.1.169]	-0.35	5.5E-201	2.2E-200	-0.39	yes
K00795	farnesyl diphosphate synthase [EC:2.5.1.1 2.5.1.10]	0.35	6.6E-201	2.6E-200	0.17	
K01147	exoribonuclease II [EC:3.1.13.1]	0.35	7.5E-201	2.9E-200	0.17	
K08310	dihydroneopterin triphosphate diphosphatase [EC:3.6.1.67]	0.35	7.9E-201	3.1E-200	0.18	
K02083	allantoate deiminase [EC:3.5.3.9]	0.35	1.0E-200	3.9E-200	0.18	
K14257	tetracycline 7-halogenase / FADH2 O2-dependent halogenase [EC:1.14.19.49 1.14.19.-]	-0.35	1.2E-200	4.7E-200	-0.39	
K00752	hyaluronan synthase [EC:2.4.1.212]	-0.35	1.9E-200	7.4E-200	-0.40	yes
K04835	methylaspartate ammonia-lyase [EC:4.3.1.2]	0.35	2.1E-200	8.2E-200	0.19	
K00201	formylmethanofuran dehydrogenase subunit B [EC:1.2.7.12]	-0.35	2.3E-200	8.9E-200	-0.39	yes
K15518	deoxyguanosine kinase [EC:2.7.1.113]	-0.35	4.3E-200	1.7E-199	-0.39	yes
K03863	vanillate monooxygenase ferredoxin subunit	-0.35	9.2E-200	3.6E-199	-0.39	yes
K13693	glucosyl-3-phosphoglycerate synthase [EC:2.4.1.266]	-0.35	1.2E-199	4.6E-199	-0.38	yes
K04799	flap endonuclease-1 [EC:3.-.-.-]	-0.35	1.4E-199	5.4E-199	-0.39	yes
K01659	2-methylcitrate synthase [EC:2.3.3.5]	0.35	2.4E-199	9.2E-199	0.18	
K02745	N-acetylgalactosamine PTS system EIIB component [EC:2.7.1.-]	0.35	2.6E-199	1.0E-198	0.20	
K00455	3,4-dihydroxyphenylacetate 2,3-dioxygenase [EC:1.13.11.15]	0.35	3.0E-199	1.2E-198	0.20	
K06981	isopentenyl phosphate kinase [EC:2.7.4.26]	-0.35	5.4E-199	2.1E-198	-0.39	yes
K01491	methylenetetrahydrofolate dehydrogenase (NADP+) / methenyltetrahydrofolate cyclohydrolase [EC:1.5.1.5 3.5.4.9]	-0.35	6.0E-199	2.3E-198	-0.33	
K14682	amino-acid N-acetyltransferase [EC:2.3.1.1]	0.35	8.1E-199	3.1E-198	0.17	
K00534	ferredoxin hydrogenase small subunit [EC:1.12.7.2]	-0.35	1.1E-198	4.2E-198	-0.39	yes
K00137	aminobutyraldehyde dehydrogenase [EC:1.2.1.19]	0.35	2.0E-198	7.6E-198	0.21	
K03050	DNA-directed RNA polymerase subunit E\ [EC:2.7.7.6]	-0.35	5.0E-198	1.9E-197	-0.39	yes
K07739	elongator complex protein 3 [EC:2.3.1.48]	-0.35	6.2E-198	2.4E-197	-0.34	yes
K12073	1,4-dihydroxy-2-naphthoyl-CoA hydrolase [EC:3.1.2.28]	-0.35	7.2E-198	2.7E-197	-0.37	

Predictor	Description	Pearson's r	P	FDR-adjusted P	Spearman's rho	Associated with fractures (P<0.05)
K13006	UDP-perosamine 4-acetyltransferase [EC:2.3.1.-]	-0.35	8.4E-198	3.2E-197	-0.40	yes
K13450	phosphothreonine lyase [EC:4.2.3.-]	0.35	9.6E-198	3.6E-197	0.25	
K07320	ribosomal protein L3 glutamine methyltransferase [EC:2.1.1.298]	0.35	9.7E-198	3.7E-197	0.17	
K15257	tRNA (mo5U34)-methyltransferase [EC:2.1.1.-]	0.35	1.2E-197	4.5E-197	0.18	
K02554	2-keto-4-pentenoate hydratase [EC:4.2.1.80]	0.35	1.2E-197	4.5E-197	0.18	
K11387	arabinosyltransferase C [EC:2.4.2.-]	-0.35	2.5E-197	9.4E-197	-0.38	yes
K10545	D-xylose transport system ATP-binding protein [EC:7.5.2.10]	0.35	2.6E-197	9.8E-197	0.18	
K11717	cysteine desulfurase / selenocysteine lyase [EC:2.8.1.7 4.4.1.16]	-0.35	2.7E-197	1.0E-196	-0.34	
K00577	tetrahydromethanopterin S-methyltransferase subunit A [EC:2.1.1.86]	-0.35	3.7E-197	1.4E-196	-0.39	yes
K02170	pimeloyl-[acyl-carrier protein] methyl ester esterase [EC:3.1.1.85]	0.35	4.0E-197	1.5E-196	0.17	
K05342	alpha,alpha-trehalose phosphorylase [EC:2.4.1.64]	-0.35	6.4E-197	2.4E-196	-0.40	yes
K16421	4-hydroxymandelate synthase [EC:1.13.11.46]	-0.35	6.9E-197	2.6E-196	-0.39	yes
K02827	cytochrome aa3-600 menaquinol oxidase subunit I [EC:7.1.1.5]	-0.35	8.9E-197	3.3E-196	-0.39	yes
K11623	two-component system, NarL family, sensor histidine kinase YdfH [EC:2.7.13.3]	-0.35	1.2E-196	4.5E-196	-0.41	yes
K03185	2-octaprenyl-6-methoxyphenol hydroxylase [EC:1.14.13.-]	0.35	1.3E-196	4.9E-196	0.17	
K03540	ribonuclease P protein subunit RPR2 [EC:3.1.26.5]	-0.35	1.4E-196	5.2E-196	-0.39	yes
K15864	nitrite reductase (NO-forming) / hydroxylamine reductase [EC:1.7.2.1 1.7.99.1]	-0.35	2.2E-196	8.2E-196	-0.38	yes
K03656	ATP-dependent DNA helicase Rep [EC:3.6.4.12]	0.35	3.0E-196	1.1E-195	0.17	
K05297	rubredoxin---NAD+ reductase [EC:1.18.1.1]	-0.34	5.0E-196	1.9E-195	-0.37	yes
K13688	cyclic beta-1,2-glucan synthetase [EC:2.4.1.-]	-0.34	6.0E-196	2.2E-195	-0.40	yes
K08600	sortase B [EC:3.4.22.71]	-0.34	6.4E-196	2.4E-195	-0.36	
K01800	maleylacetoacetate isomerase [EC:5.2.1.2]	-0.34	2.3E-195	8.5E-195	-0.38	yes
K05936	precorrin-4/cobalt-precorrin-4 C11-methyltransferase [EC:2.1.1.133 2.1.1.271]	-0.34	2.3E-195	8.5E-195	-0.37	
K15512	benzoyl-CoA 2,3-epoxidase subunit B [EC:1.14.13.208]	-0.34	2.3E-195	8.5E-195	-0.38	yes
K00003	homoserine dehydrogenase [EC:1.1.1.3]	-0.34	2.5E-195	9.2E-195	-0.35	
K11211	3-deoxy-D-manno-octulosonic acid kinase [EC:2.7.1.166]	-0.34	4.6E-195	1.7E-194	-0.37	yes
K12986	1,5-rhamnosyltransferase [EC:2.4.1.-]	0.34	1.6E-194	5.9E-194	0.30	
K00484	flavin reductase (NADH) [EC:1.5.1.36]	0.34	1.9E-194	7.0E-194	0.20	
K15981	cholest-4-en-3-one 26-monooxygenase [EC:1.14.15.29]	-0.34	2.1E-194	7.7E-194	-0.37	
K01687	dihydroxy-acid dehydratase [EC:4.2.1.9]	-0.34	3.7E-194	1.4E-193	-0.35	
K15731	carboxy-terminal domain RNA polymerase II polypeptide A small phosphatase [EC:3.1.3.16]	-0.34	3.9E-194	1.4E-193	-0.38	
K00020	3-hydroxyisobutyrate dehydrogenase [EC:1.1.1.31]	-0.34	6.5E-194	2.4E-193	-0.36	
K07336	PKHD-type hydroxylase [EC:1.14.11.-]	0.34	7.7E-194	2.8E-193	0.18	
K11181	dissimilatory sulfite reductase beta subunit [EC:1.8.99.5]	-0.34	8.0E-194	2.9E-193	-0.35	
K04568	elongation factor P--(R)-beta-lysine ligase [EC:6.3.1.-]	0.34	1.0E-193	3.6E-193	0.17	
K01465	dihydroorotase [EC:3.5.2.3]	-0.34	1.3E-193	4.7E-193	-0.33	
K08317	hydroxycarboxylate dehydrogenase A [EC:1.1.1.-]	0.34	1.6E-193	5.8E-193	0.22	
K02319	DNA polymerase, archaea type [EC:2.7.7.7]	-0.34	2.2E-193	8.0E-193	-0.38	yes
K04765	nucleoside triphosphate diphosphatase [EC:3.6.1.9]	0.34	2.3E-193	8.3E-193	0.17	
K10796	D-proline reductase (dithiol)-stabilizing protein PrdE	-0.34	2.4E-193	8.7E-193	-0.40	yes
K01358	ATP-dependent Clp protease, protease subunit [EC:3.4.21.92]	-0.34	2.8E-193	1.0E-192	-0.37	
K00203	formylmethanofuran dehydrogenase subunit D [EC:1.2.7.12]	-0.34	1.2E-192	4.3E-192	-0.38	yes

Predictor	Description	Pearson's r	P	FDR-adjusted P	Spearman's rho	Associated with fractures (P<0.05)
K03047	DNA-directed RNA polymerase subunit D [EC:2.7.7.6]	-0.34	1.3E-192	4.7E-192	-0.38	yes
K14127	F420-non-reducing hydrogenase iron-sulfur subunit [EC:1.12.99.- 1.8.98.5 1.8.98.6]	-0.34	1.3E-192	4.7E-192	-0.38	yes
K00555	tRNA (guanine26-N2/guanine27-N2)-dimethyltransferase [EC:2.1.1.215 2.1.1.216]	-0.34	1.4E-192	5.0E-192	-0.39	yes
K00948	ribose-phosphate pyrophosphokinase [EC:2.7.6.1]	-0.34	1.4E-192	5.0E-192	-0.38	
K01182	oligo-1,6-glucosidase [EC:3.2.1.10]	-0.34	1.6E-192	5.7E-192	-0.34	
K07557	archaeosine synthase alpha-subunit [EC:2.6.1.97 2.6.1.-]	-0.34	1.9E-192	6.8E-192	-0.39	yes
K05797	4-cresol dehydrogenase (hydroxylating) flavoprotein subunit [EC:1.17.9.1]	-0.34	2.2E-192	7.9E-192	-0.39	
K01623	fructose-bisphosphate aldolase, class I [EC:4.1.2.13]	-0.34	2.2E-192	7.9E-192	-0.37	yes
K14587	protein sgcE [EC:5.1.3.-]	0.34	2.4E-192	8.6E-192	0.17	
K13017	UDP-2-acetamido-2-deoxy-ribo-hexuluronate aminotransferase [EC:2.6.1.98]	-0.34	2.4E-192	8.6E-192	-0.38	yes
K08965	2,3-diketo-5-methylthiopentyl-1-phosphate enolase [EC:5.3.2.5]	-0.34	2.6E-192	9.3E-192	-0.39	yes
K03041	DNA-directed RNA polymerase subunit A' [EC:2.7.7.6]	-0.34	4.4E-192	1.6E-191	-0.39	yes
K03824	putative acetyltransferase [EC:2.3.1.-]	0.34	4.6E-192	1.6E-191	0.17	
K07254	tRNA (cytidine56-2'-O)-methyltransferase [EC:2.1.1.206]	-0.34	7.7E-192	2.7E-191	-0.38	yes
K02615	3-oxo-5,6-didehydrosuberyl-CoA/3-oxoadipyl-CoA thiolase [EC:2.3.1.223 2.3.1.174]	0.34	8.9E-192	3.2E-191	0.23	
K07558	tRNA nucleotidyltransferase (CCA-adding enzyme) [EC:2.7.7.72]	-0.34	1.1E-191	3.9E-191	-0.38	yes
K13004	galacturonosyltransferase WbtD [EC:2.4.1.-]	-0.34	1.3E-191	4.6E-191	-0.37	yes
K15669	D-glycero-alpha-D-manno-heptose 1-phosphate guanylyltransferase [EC:2.7.7.71]	-0.34	1.5E-191	5.3E-191	-0.34	yes
K04101	protocatechuate 4,5-dioxygenase, beta chain [EC:1.13.11.8]	-0.34	2.0E-191	7.1E-191	-0.38	yes
K01905	acetate---CoA ligase (ADP-forming) subunit alpha [EC:6.2.1.13]	-0.34	2.3E-191	8.1E-191	-0.40	
K07650	two-component system, OmpR family, sensor histidine kinase CssS [EC:2.7.13.3]	-0.34	4.9E-191	1.7E-190	-0.35	
K01387	microbial collagenase [EC:3.4.24.3]	-0.34	4.9E-191	1.7E-190	-0.40	yes
K16050	4,5:9,10-diseco-3-hydroxy-5,9,17-trioxoandrosta-1(10),2-diene-4-oate hydrolase [EC:3.7.1.17]	-0.34	5.2E-191	1.8E-190	-0.39	
K01571	oxaloacetate decarboxylase (Na+ extruding) subunit alpha [EC:7.2.4.2]	-0.34	9.0E-191	3.2E-190	-0.35	
K09482	glutamyl-tRNA(Gln) amidotransferase subunit D [EC:6.3.5.7]	-0.34	1.8E-190	6.3E-190	-0.38	yes
K03582	exodeoxyribonuclease V beta subunit [EC:3.1.11.5]	0.34	2.0E-190	7.0E-190	0.16	
K14654	2,5-diamino-6-(ribosylamino)-4(3H)-pyrimidinone 5'-phosphate reductase [EC:1.1.1.302]	-0.34	2.8E-190	9.8E-190	-0.38	yes
K00523	CDP-4-dehydro-6-deoxyglucose reductase, E3 [EC:1.17.1.1]	-0.34	2.9E-190	1.0E-189	-0.38	yes
K07178	RIO kinase 1 [EC:2.7.11.1]	-0.34	3.0E-190	1.0E-189	-0.39	yes
K07546	E-phenylitaconyl-CoA hydratase [EC:4.2.1.-]	-0.34	5.6E-190	2.0E-189	-0.39	yes
K07644	two-component system, OmpR family, heavy metal sensor histidine kinase CusS [EC:2.7.13.3]	0.34	5.8E-190	2.0E-189	0.17	
K01720	2-methylcitrate dehydratase [EC:4.2.1.79]	0.34	9.4E-190	3.3E-189	0.17	
K02434	aspartyl-tRNA(Asn)/glutamyl-tRNA(Gln) amidotransferase subunit B [EC:6.3.5.6 6.3.5.7]	-0.34	1.0E-189	3.5E-189	-0.35	
K03330	glutamyl-tRNA(Gln) amidotransferase subunit E [EC:6.3.5.7]	-0.34	1.1E-189	3.8E-189	-0.38	yes
K13531	methylated-DNA-[protein]-cysteine S-methyltransferase [EC:2.1.1.63]	-0.34	1.4E-189	4.9E-189	-0.40	yes
K08304	membrane-bound lytic murein transglycosylase A [EC:4.2.2.-]	0.34	1.7E-189	5.9E-189	0.16	
K14164	glycyl-tRNA synthetase [EC:6.1.1.14]	-0.34	1.8E-189	6.2E-189	-0.40	yes
K02339	DNA polymerase III subunit chi [EC:2.7.7.7]	0.34	2.1E-189	7.3E-189	0.16	
K02683	DNA primase small subunit [EC:2.7.7.102]	-0.34	2.4E-189	8.3E-189	-0.38	yes
K13280	signal peptidase I [EC:3.4.21.89]	-0.34	3.0E-189	1.0E-188	-0.38	yes
K01003	oxaloacetate decarboxylase [EC:4.1.1.112]	-0.34	3.2E-189	1.1E-188	-0.38	yes
K11131	H/ACA ribonucleoprotein complex subunit 4 [EC:5.4.99.-]	-0.34	3.2E-189	1.1E-188	-0.37	yes

Predictor	Description	Pearson's r	P	FDR-adjusted P	Spearman's rho	Associated with fractures (P<0.05)
K03051	DNA-directed RNA polymerase subunit F [EC:2.7.7.6]	-0.34	5.6E-189	1.9E-188	-0.38	yes
K01444	N4-(beta-N-acetylglucosaminyl)-L-asparaginase [EC:3.5.1.26]	-0.34	1.6E-188	5.5E-188	-0.35	yes
K07446	tRNA (guanine10-N2)-dimethyltransferase [EC:2.1.1.213]	-0.34	2.4E-188	8.2E-188	-0.38	yes
K03429	processive 1,2-diacylglycerol beta-glucosyltransferase [EC:2.4.1.315]	-0.34	3.0E-188	1.0E-187	-0.36	
K06209	chorismate mutase [EC:5.4.99.5]	-0.34	5.6E-188	1.9E-187	-0.35	
K07260	zinc D-Ala-D-Ala carboxypeptidase [EC:3.4.17.14]	-0.34	6.7E-188	2.3E-187	-0.34	
K08082	two-component system, LytTR family, sensor histidine kinase AlgZ [EC:2.7.13.3]	-0.34	8.9E-188	3.0E-187	-0.37	yes
K13381	bifunctional chitinase/lysozyme [EC:3.2.1.14 3.2.1.17]	0.34	9.5E-188	3.2E-187	0.29	
K16218	2-methylisoborneol synthase [EC:4.2.3.118]	-0.34	9.7E-188	3.3E-187	-0.39	yes
K16647	arabinofuranan 3-O-arabinosyltransferase [EC:2.4.2.47]	-0.34	1.0E-187	3.4E-187	-0.38	yes
K15983	3-ketosteroid 9alpha-monooxygenase subunit B [EC:1.14.15.30]	-0.34	1.2E-187	4.1E-187	-0.38	
K16217	geranyl diphosphate 2-C-methyltransferase [EC:2.1.1.255]	-0.34	1.8E-187	6.1E-187	-0.39	
K07055	tRNA wybutosine-synthesizing protein 2 [EC:2.5.1.114]	-0.34	3.0E-187	1.0E-186	-0.38	yes
K00440	coenzyme F420 hydrogenase subunit alpha [EC:1.12.98.1]	-0.34	4.7E-187	1.6E-186	-0.38	yes
K05939	acyl-[acyl-carrier-protein]-phospholipid O-acyltransferase / long-chain-fatty-acid--[acyl-carrier-protein] ligase [EC:2.3.1.40 6.2.1.20]	0.34	4.7E-187	1.6E-186	0.17	
K02204	homoserine kinase type II [EC:2.7.1.39]	-0.34	5.1E-187	1.7E-186	-0.37	
K01846	methylaspartate mutase sigma subunit [EC:5.4.99.1]	0.34	5.9E-187	2.0E-186	0.18	
K01721	nitrile hydratase subunit alpha [EC:4.2.1.84]	-0.34	7.3E-187	2.5E-186	-0.37	
K00443	coenzyme F420 hydrogenase subunit gamma [EC:1.12.98.1]	-0.34	7.7E-187	2.6E-186	-0.38	yes
K01682	aconitate hydratase 2 / 2-methylisocitrate dehydratase [EC:4.2.1.3 4.2.1.99]	0.34	7.9E-187	2.7E-186	0.16	
K07583	tRNA pseudouridine synthase 10 [EC:5.4.99.25]	-0.34	8.7E-187	2.9E-186	-0.40	yes
K08604	vibriolysin [EC:3.4.24.25]	-0.34	9.2E-187	3.1E-186	-0.39	
K12981	KDO transferase III [EC:2.4.99.-]	0.34	1.1E-186	3.7E-186	0.28	
K07708	two-component system, NtrC family, nitrogen regulation sensor histidine kinase GlnL [EC:2.7.13.3]	0.34	1.1E-186	3.7E-186	0.16	
K03538	ribonuclease P protein subunit POP4 [EC:3.1.26.5]	-0.34	1.2E-186	4.0E-186	-0.38	yes
K03056	DNA-directed RNA polymerase subunit L [EC:2.7.7.6]	-0.34	1.3E-186	4.3E-186	-0.38	yes
K03465	thymidylate synthase (FAD) [EC:2.1.1.148]	-0.34	1.8E-186	6.0E-186	-0.35	
K16049	3,4-dihydroxy-9,10-secoandrosta-1,3,5(10)-triene-9,17-dione 4,5-dioxygenase [EC:1.13.11.25]	-0.34	1.9E-186	6.3E-186	-0.39	yes
K02302	uroporphyrin-III C-methyltransferase / precorrin-2 dehydrogenase / sirohydrochlorin ferrochelatase [EC:2.1.1.107 1.3.1.76 4.99.1.4]	0.34	2.1E-186	7.0E-186	0.16	
K03417	methylisocitrate lyase [EC:4.1.3.30]	0.34	3.3E-186	1.1E-185	0.16	
K00008	L-iditol 2-dehydrogenase [EC:1.1.1.14]	-0.34	3.9E-186	1.3E-185	-0.38	yes
K14727	3-oxoadipate enol-lactonase / 4-carboxymuconolactone decarboxylase [EC:3.1.1.24 4.1.1.44]	-0.34	4.8E-186	1.6E-185	-0.38	yes
K03669	membrane glycosyltransferase [EC:2.4.1.-]	0.34	5.7E-186	1.9E-185	0.17	
K02626	arginine decarboxylase [EC:4.1.1.19]	-0.34	9.6E-186	3.2E-185	-0.37	yes
K00370	nitrate reductase / nitrite oxidoreductase, alpha subunit [EC:1.7.5.1 1.7.99.-]	0.34	1.1E-185	3.6E-185	0.19	
K03672	thioredoxin 2 [EC:1.8.1.8]	0.34	2.2E-185	7.3E-185	0.16	
K01908	propionyl-CoA synthetase [EC:6.2.1.17]	0.34	2.3E-185	7.6E-185	0.16	
K05830	LysW-gamma-L-lysine/LysW-L-ornithine aminotransferase [EC:2.6.1.118 2.6.1.-]	-0.34	2.8E-185	9.2E-185	-0.38	
K15519	deoxyadenosine/deoxycytidine kinase [EC:2.7.1.76 2.7.1.74]	-0.34	4.3E-185	1.4E-184	-0.39	yes
K00584	tetrahydromethanopterin S-methyltransferase subunit H [EC:2.1.1.86]	-0.34	5.0E-185	1.6E-184	-0.38	yes
K01678	fumarate hydratase subunit beta [EC:4.2.1.2]	-0.34	7.9E-185	2.6E-184	-0.36	
K14340	mannosyltransferase [EC:2.4.1.-]	-0.34	1.3E-184	4.3E-184	-0.39	

Predictor	Description	Pearson's r	P	FDR-adjusted P	Spearman's rho	Associated with fractures (P<0.05)
K02802	NA	-0.34	1.7E-184	5.6E-184	-0.37	
K10854	acetone carboxylase, alpha subunit [EC:6.4.1.6]	-0.34	2.8E-184	9.2E-184	-0.37	yes
K00014	shikimate dehydrogenase [EC:1.1.1.25]	-0.33	3.0E-184	9.8E-184	-0.35	
K00579	tetrahydromethanopterin S-methyltransferase subunit C [EC:2.1.1.86]	-0.33	3.2E-184	1.0E-183	-0.38	yes
K13085	phosphatidylinositol-4,5-bisphosphate 4-phosphatase [EC:3.1.3.78]	0.33	3.6E-184	1.2E-183	0.29	
K15871	bile acid CoA-transferase [EC:2.8.3.25]	-0.33	4.5E-184	1.5E-183	-0.36	yes
K15872	bile-acid 7alpha-dehydratase [EC:4.2.1.106]	-0.33	4.5E-184	1.5E-183	-0.36	yes
K00578	tetrahydromethanopterin S-methyltransferase subunit B [EC:2.1.1.86]	-0.33	6.0E-184	2.0E-183	-0.37	yes
K10978	methanogen homoisocitrate dehydrogenase [EC:1.1.1.87 1.1.1.-]	-0.33	6.0E-184	2.0E-183	-0.38	yes
K00758	thymidine phosphorylase [EC:2.4.2.4]	0.33	8.1E-184	2.6E-183	0.16	
K02778	glucose PTS system EIIB component [EC:2.7.1.199]	0.33	8.6E-184	2.8E-183	0.28	
K07284	sortase A [EC:3.4.22.70]	-0.33	8.6E-184	2.8E-183	-0.34	yes
K00319	methylenetetrahydromethanopterin dehydrogenase [EC:1.5.98.1]	-0.33	1.1E-183	3.6E-183	-0.37	yes
K03382	hydroxydechloroatrazine ethylaminohydrolase [EC:3.5.4.43]	-0.33	1.6E-183	5.2E-183	-0.33	yes
K13950	para-aminobenzoate synthetase [EC:2.6.1.85]	-0.33	2.8E-183	9.0E-183	-0.37	yes
K03539	ribonuclease P/MRP protein subunit RPP1 [EC:3.1.26.5]	-0.33	2.9E-183	9.4E-183	-0.38	yes
K08305	membrane-bound lytic murein transglycosylase B [EC:4.2.2.-]	0.33	4.2E-183	1.4E-182	0.15	
K00374	nitrate reductase gamma subunit [EC:1.7.5.1 1.7.99.-]	0.33	5.3E-183	1.7E-182	0.19	
K01212	levanase [EC:3.2.1.65]	-0.33	5.7E-183	1.8E-182	-0.39	yes
K00068	sorbitol-6-phosphate 2-dehydrogenase [EC:1.1.1.140]	0.33	5.9E-183	1.9E-182	0.21	
K03537	ribonuclease P/MRP protein subunit POP5 [EC:3.1.26.5]	-0.33	1.1E-182	3.5E-182	-0.38	yes
K05577	NAD(P)H-quinone oxidoreductase subunit 5 [EC:7.1.1.2]	-0.33	1.3E-182	4.2E-182	-0.37	yes
K01596	phosphoenolpyruvate carboxykinase (GTP) [EC:4.1.1.32]	-0.33	1.8E-182	5.8E-182	-0.33	
K00401	methyl-coenzyme M reductase beta subunit [EC:2.8.4.1]	-0.33	3.3E-182	1.1E-181	-0.37	yes
K11781	5-amino-6-(D-ribitylamino)uracil---L-tyrosine 4-hydroxyphenyl transferase [EC:2.5.1.147]	-0.33	4.9E-182	1.6E-181	-0.37	yes
K16306	fructose-bisphosphate aldolase / 2-amino-3,7-dideoxy-D-threo-hept-6-ulosonate synthase [EC:4.1.2.13 2.2.1.10]	-0.33	5.4E-182	1.7E-181	-0.38	yes
K00399	methyl-coenzyme M reductase alpha subunit [EC:2.8.4.1]	-0.33	5.5E-182	1.8E-181	-0.37	yes
K10855	acetone carboxylase, beta subunit [EC:6.4.1.6]	-0.33	5.6E-182	1.8E-181	-0.38	yes
K10622	HCOMODA/2-hydroxy-3-carboxy-muconic semialdehyde decarboxylase [EC:4.1.1.-]	-0.33	6.4E-182	2.0E-181	-0.38	
K02314	replicative DNA helicase [EC:3.6.4.12]	-0.33	1.7E-181	5.4E-181	-0.36	
K01638	malate synthase [EC:2.3.3.9]	0.33	1.9E-181	6.0E-181	0.15	
K13007	glycosyltransferase WbpL [EC:2.4.1.-]	-0.33	2.2E-181	7.0E-181	-0.37	
K00117	quinoprotein glucose dehydrogenase [EC:1.1.5.2]	0.33	2.4E-181	7.6E-181	0.17	
K10778	AraC family transcriptional regulator, regulatory protein of adaptative response / methylated-DNA-[protein]-cysteine methyltransferase [EC:2.1.1.63]	0.33	2.5E-181	7.9E-181	0.14	
K01560	2-haloacid dehalogenase [EC:3.8.1.2]	-0.33	3.5E-181	1.1E-180	-0.35	
K01677	fumarate hydratase subunit alpha [EC:4.2.1.2]	-0.33	4.2E-181	1.3E-180	-0.36	
K01415	endothelin-converting enzyme [EC:3.4.24.71]	-0.33	4.3E-181	1.4E-180	-0.39	yes
K01753	D-serine dehydratase [EC:4.3.1.18]	0.33	5.2E-181	1.6E-180	0.15	
K13602	bacteriochlorophyllide d C-12(1)-methyltransferase [EC:2.1.1.331]	-0.33	6.1E-181	1.9E-180	-0.37	
K03653	N-glycosylase/DNA lyase [EC:3.2.2.- 4.2.99.18]	-0.33	1.2E-180	3.8E-180	-0.39	yes
K00469	inositol oxygenase [EC:1.13.99.1]	-0.33	1.7E-180	5.3E-180	-0.36	yes
K07732	riboflavin kinase, archaea type [EC:2.7.1.161]	-0.33	2.8E-180	8.8E-180	-0.38	yes

Predictor	Description	Pearson's r	P	FDR-adjusted P	Spearman's rho	Associated with fractures (P<0.05)
K03058	DNA-directed RNA polymerase subunit N [EC:2.7.7.6]	-0.33	3.3E-180	1.0E-179	-0.37	yes
K03059	DNA-directed RNA polymerase subunit P [EC:2.7.7.6]	-0.33	3.3E-180	1.0E-179	-0.37	yes
K08311	putative (di)nucleoside polyphosphate hydrolase [EC:3.6.1.-]	0.33	5.4E-180	1.7E-179	0.15	
K02435	aspartyl-tRNA(Asn)/glutamyl-tRNA(Gln) amidotransferase subunit C [EC:6.3.5.6 6.3.5.7]	-0.33	5.9E-180	1.8E-179	-0.34	
K03416	methylmalonyl-CoA carboxyltransferase 5S subunit [EC:2.1.3.1]	-0.33	7.7E-180	2.4E-179	-0.37	
K00402	methyl-coenzyme M reductase gamma subunit [EC:2.8.4.1]	-0.33	8.8E-180	2.7E-179	-0.37	yes
K05873	adenylate cyclase, class 2 [EC:4.6.1.1]	-0.33	1.3E-179	4.1E-179	-0.38	yes
K07389	cytolysin-activating lysine-acyltransferase [EC:2.3.1.-]	0.33	2.4E-179	7.5E-179	0.20	
K15982	3-ketosteroid 9alpha-monooxygenase subunit A [EC:1.14.15.30]	-0.33	3.1E-179	9.6E-179	-0.38	yes
K14668	minimal PKS chain-length factor (CLF/KS beta) [EC:2.3.1.-]	-0.33	3.3E-179	1.0E-178	-0.37	
K01574	acetoacetate decarboxylase [EC:4.1.1.4]	-0.33	4.6E-179	1.4E-178	-0.37	
K00596	2,2-dialkylglycine decarboxylase (pyruvate) [EC:4.1.1.64]	-0.33	9.8E-179	3.0E-178	-0.36	yes
K14261	alanine-synthesizing transaminase [EC:2.6.1.-]	0.33	1.2E-178	3.7E-178	0.16	
K08744	cardiolipin synthase (CMP-forming) [EC:2.7.8.41]	-0.33	2.0E-178	6.2E-178	-0.33	
K07127	5-hydroxyisourate hydrolase [EC:3.5.2.17]	0.33	2.1E-178	6.5E-178	0.15	
K10806	acyl-CoA thioesterase YciA [EC:3.1.2.-]	0.33	2.5E-178	7.7E-178	0.17	
K01214	isoamylase [EC:3.2.1.68]	-0.33	2.6E-178	8.0E-178	-0.33	
K12254	4-guanidinobutyraldehyde dehydrogenase / NAD-dependent aldehyde dehydrogenase [EC:1.2.1.54 1.2.1.-]	-0.33	5.4E-178	1.7E-177	-0.38	yes
K03981	thiol:disulfide interchange protein DsbC [EC:5.3.4.1]	0.33	6.5E-178	2.0E-177	0.15	
K04517	prephenate dehydrogenase [EC:1.3.1.12]	-0.33	7.9E-178	2.4E-177	-0.32	
K05712	3-(3-hydroxy-phenyl)propionate hydroxylase [EC:1.14.13.127]	0.33	3.3E-177	1.0E-176	0.15	
K01663	imidazole glycerol-phosphate synthase [EC:4.3.2.10]	-0.33	5.0E-177	1.5E-176	-0.37	
K09019	3-hydroxypropanoate dehydrogenase [EC:1.1.1.-]	0.33	6.2E-177	1.9E-176	0.16	
K00371	nitrate reductase / nitrite oxidoreductase, beta subunit [EC:1.7.5.1 1.7.99.-]	0.33	6.7E-177	2.1E-176	0.19	
K07991	archaeal preflagellin peptidase FlaK [EC:3.4.23.52]	-0.33	8.0E-177	2.4E-176	-0.39	
K01170	tRNA-intron endonuclease, archaea type [EC:4.6.1.16]	-0.33	8.3E-177	2.5E-176	-0.37	yes
K02323	DNA polymerase II small subunit [EC:2.7.7.7]	-0.33	9.0E-177	2.7E-176	-0.37	yes
K01974	RNA 3'-terminal phosphate cyclase (ATP) [EC:6.5.1.4]	0.33	1.5E-176	4.6E-176	0.18	
K05916	nitric oxide dioxygenase [EC:1.14.12.17]	0.33	1.7E-176	5.2E-176	0.14	
K05825	2-aminoadipate transaminase [EC:2.6.1.-]	-0.33	1.7E-176	5.2E-176	-0.36	
K10253	DOPA 4,5-dioxygenase [EC:1.14.99.-]	-0.33	1.8E-176	5.5E-176	-0.38	yes
K10977	methanogen homocitrate synthase [EC:2.3.3.14 2.3.3.-]	-0.33	2.0E-176	6.1E-176	-0.37	yes
K13995	maleamate amidohydrolase [EC:3.5.1.107]	-0.33	2.1E-176	6.4E-176	-0.39	
K01286	D-alanyl-D-alanine carboxypeptidase [EC:3.4.16.4]	-0.33	2.1E-176	6.4E-176	-0.37	yes
K03518	aerobic carbon-monoxide dehydrogenase small subunit [EC:1.2.5.3]	-0.33	2.3E-176	7.0E-176	-0.36	
K07179	RIO kinase 2 [EC:2.7.11.1]	-0.33	2.7E-176	8.2E-176	-0.39	
K16328	pseudouridine kinase [EC:2.7.1.83]	0.33	3.6E-176	1.1E-175	0.15	
K05352	ribitol-5-phosphate 2-dehydrogenase (NADP+) [EC:1.1.1.405]	-0.33	5.6E-176	1.7E-175	-0.36	yes
K07540	benzylsuccinate synthase [EC:4.1.99.11]	-0.33	5.7E-176	1.7E-175	-0.38	yes
K13779	isohexenylglutaconyl-CoA hydratase [EC:4.2.1.57]	-0.33	6.5E-176	2.0E-175	-0.37	
K02122	V/A-type H+/Na+-transporting ATPase subunit F	-0.33	6.6E-176	2.0E-175	-0.35	
K03045	DNA-directed RNA polymerase subunit B\ [EC:2.7.7.6]	-0.33	8.6E-176	2.6E-175	-0.37	yes

Predictor	Description	Pearson's r	P	FDR-adjusted P	Spearman's rho	Associated with fractures (P<0.05)
K11176	IMP cyclohydrolase [EC:3.5.4.10]	-0.33	1.1E-175	3.3E-175	-0.37	yes
K13039	sulfopyruvate decarboxylase subunit beta [EC:4.1.1.79]	-0.33	1.1E-175	3.3E-175	-0.37	yes
K15855	exo-1,4-beta-D-glucosaminidase [EC:3.2.1.165]	-0.33	1.1E-175	3.3E-175	-0.34	
K13831	3-hexulose-6-phosphate synthase / 6-phospho-3-hexuloisomerase [EC:4.1.2.43 5.3.1.27]	-0.33	1.2E-175	3.6E-175	-0.39	yes
K08252	receptor protein-tyrosine kinase [EC:2.7.10.1]	-0.33	1.3E-175	3.9E-175	-0.37	yes
K00108	choline dehydrogenase [EC:1.1.99.1]	0.33	1.4E-175	4.2E-175	0.15	
K00580	tetrahydromethanopterin S-methyltransferase subunit D [EC:2.1.1.86]	-0.33	1.7E-175	5.1E-175	-0.37	yes
K03049	DNA-directed RNA polymerase subunit E' [EC:2.7.7.6]	-0.33	1.7E-175	5.1E-175	-0.39	yes
K00581	tetrahydromethanopterin S-methyltransferase subunit E [EC:2.1.1.86]	-0.33	4.4E-175	1.3E-174	-0.38	
K03044	DNA-directed RNA polymerase subunit B' [EC:2.7.7.6]	-0.33	5.3E-175	1.6E-174	-0.37	yes
K11444	two-component system, chemotaxis family, response regulator WspR [EC:2.7.7.65]	-0.33	8.0E-175	2.4E-174	-0.37	yes
K05540	tRNA-dihydrouridine synthase B [EC:1.-.-.-]	0.33	1.1E-174	3.3E-174	0.15	
K03383	cyanuric acid amidohydrolase [EC:3.5.2.15]	-0.33	2.7E-174	8.0E-174	-0.39	
K03053	DNA-directed RNA polymerase subunit H [EC:2.7.7.6]	-0.33	4.8E-174	1.4E-173	-0.37	yes
K14520	4-hydroxyacetophenone monooxygenase [EC:1.14.13.84]	-0.33	5.6E-174	1.7E-173	-0.37	yes
K15888	tritrans,polycis-undecaprenyl-diphosphate synthase [geranylgeranyl-diphosphate specific] [EC:2.5.1.89]	-0.33	6.5E-174	1.9E-173	-0.37	yes
K07538	6-hydroxycyclohex-1-ene-1-carbonyl-CoA dehydrogenase [EC:1.1.1.368]	-0.33	9.3E-174	2.8E-173	-0.37	yes
K05822	tetrahydrodipicolinate N-acetyltransferase [EC:2.3.1.89]	-0.33	1.7E-173	5.0E-173	-0.36	
K01708	galactarate dehydratase [EC:4.2.1.42]	0.33	1.9E-173	5.6E-173	0.17	
K00116	malate dehydrogenase (quinone) [EC:1.1.5.4]	0.33	2.3E-173	6.8E-173	0.15	
K00248	butyryl-CoA dehydrogenase [EC:1.3.8.1]	-0.33	3.2E-173	9.4E-173	-0.31	
K00817	histidinol-phosphate aminotransferase [EC:2.6.1.9]	-0.33	3.3E-173	9.7E-173	-0.34	
K08280	lipopolysaccharide O-acetyltransferase [EC:2.3.1.-]	0.33	3.4E-173	1.0E-172	0.25	
K09722	4-phosphopantoate---beta-alanine ligase [EC:6.3.2.36]	-0.33	3.6E-173	1.1E-172	-0.37	yes
K00043	4-hydroxybutyrate dehydrogenase [EC:1.1.1.61]	-0.33	4.9E-173	1.4E-172	-0.36	yes
K01668	tyrosine phenol-lyase [EC:4.1.99.2]	-0.32	1.0E-172	2.9E-172	-0.34	yes
K12553	penicillin-binding protein 3 [EC:3.4.-.-]	-0.32	1.5E-172	4.4E-172	-0.33	
K00694	cellulose synthase (UDP-forming) [EC:2.4.1.12]	0.32	1.6E-172	4.7E-172	0.15	
K03042	DNA-directed RNA polymerase subunit A\ [EC:2.7.7.6]	-0.32	1.8E-172	5.3E-172	-0.37	yes
K11711	two-component system, LuxR family, sensor histidine kinase DctS [EC:2.7.13.3]	-0.32	1.9E-172	5.5E-172	-0.37	yes
K06863	5-formaminoimidazole-4-carboxamide-1-(beta)-D-ribofuranosyl 5'-monophosphate synthetase [EC:6.3.4.23]	-0.32	2.0E-172	5.8E-172	-0.36	
K01857	3-carboxy-cis,cis-muconate cycloisomerase [EC:5.5.1.2]	-0.32	3.2E-172	9.3E-172	-0.37	yes
K10680	N-ethylmaleimide reductase [EC:1.-.-.-]	0.32	3.7E-172	1.1E-171	0.14	
K13603	3-vinyl bacteriochlorophyllide hydratase [EC:4.2.1.169]	-0.32	4.3E-172	1.3E-171	-0.36	yes
K00826	branched-chain amino acid aminotransferase [EC:2.6.1.42]	-0.32	4.7E-172	1.4E-171	-0.34	
K02322	DNA polymerase II large subunit [EC:2.7.7.7]	-0.32	5.5E-172	1.6E-171	-0.38	yes
K10844	DNA excision repair protein ERCC-2 [EC:3.6.4.12]	-0.32	6.4E-172	1.9E-171	-0.40	yes
K01920	glutathione synthase [EC:6.3.2.3]	0.32	8.9E-172	2.6E-171	0.14	
K01705	homoaconitate hydratase [EC:4.2.1.36]	-0.32	9.8E-172	2.8E-171	-0.35	yes
K02201	pantetheine-phosphate adenylyltransferase [EC:2.7.7.3]	-0.32	1.4E-171	4.0E-171	-0.37	yes
K13812	bifunctional enzyme Fae/Hps [EC:4.2.1.147 4.1.2.43]	-0.32	1.4E-171	4.0E-171	-0.36	yes
K00101	L-lactate dehydrogenase (cytochrome) [EC:1.1.2.3]	0.32	1.5E-171	4.3E-171	0.14	

Predictor	Description	Pearson's r	P	FDR-adjusted P	Spearman's rho	Associated with fractures (P<0.05)
K00042	2-hydroxy-3-oxopropionate reductase [EC:1.1.1.60]	0.32	1.7E-171	4.9E-171	0.14	
K03396	S-(hydroxymethyl)glutathione synthase [EC:4.4.1.22]	-0.32	7.0E-171	2.0E-170	-0.37	yes
K16317	tRNA (pseudouridine54-N1)-methyltransferase [EC:2.1.1.257]	-0.32	1.1E-170	3.2E-170	-0.39	yes
K00583	tetrahydromethanopterin S-methyltransferase subunit G [EC:2.1.1.86]	-0.32	1.2E-170	3.5E-170	-0.36	yes
K14028	methanol dehydrogenase (cytochrome c) subunit 1 [EC:1.1.2.7]	-0.32	1.5E-170	4.3E-170	-0.37	
K10795	D-proline reductase (dithiol)-stabilizing protein PrdD	-0.32	2.1E-170	6.0E-170	-0.39	yes
K15533	1,3-beta-galactosyl-N-acetylhexosamine phosphorylase [EC:2.4.1.211]	-0.32	2.1E-170	6.0E-170	-0.32	
K14083	trimethylamine---corrinoid protein Co-methyltransferase [EC:2.1.1.250]	-0.32	2.1E-170	6.0E-170	-0.35	yes
K07816	GTP pyrophosphokinase [EC:2.7.6.5]	-0.32	2.8E-170	8.0E-170	-0.33	
K00461	arachidonate 5-lipoxygenase [EC:1.13.11.34]	-0.32	2.9E-170	8.3E-170	-0.37	
K03081	NA	0.32	3.0E-170	8.6E-170	0.19	
K01782	3-hydroxyacyl-CoA dehydrogenase / enoyl-CoA hydratase / 3-hydroxybutyryl-CoA epimerase [EC:1.1.1.35 4.2.1.17 5.1.2.3]	0.32	6.7E-170	1.9E-169	0.14	
K00218	protochlorophyllide reductase [EC:1.3.1.33]	-0.32	8.2E-170	2.3E-169	-0.37	
K15913	UDP-N-acetylbacillosamine N-acetyltransferase [EC:2.3.1.203]	-0.32	9.8E-170	2.8E-169	-0.33	
K00789	S-adenosylmethionine synthetase [EC:2.5.1.6]	-0.32	1.2E-169	3.4E-169	-0.34	
K03380	phenol 2-monooxygenase (NADPH) [EC:1.14.13.7]	-0.32	1.5E-169	4.3E-169	-0.36	
K15746	beta-carotene 3-hydroxylase [EC:1.14.15.24]	-0.32	2.9E-169	8.2E-169	-0.35	yes
K01923	phosphoribosylaminoimidazole-succinocarboxamide synthase [EC:6.3.2.6]	-0.32	3.6E-169	1.0E-168	-0.31	
K10714	methylene-tetrahydromethanopterin dehydrogenase [EC:1.5.1.-]	-0.32	4.0E-169	1.1E-168	-0.37	
K00472	prolyl 4-hydroxylase [EC:1.14.11.2]	-0.32	1.2E-168	3.4E-168	-0.37	yes
K05922	quinone-reactive Ni/Fe-hydrogenase large subunit [EC:1.12.5.1]	-0.32	1.4E-168	4.0E-168	-0.36	yes
K14680	RNA ligase [EC:6.5.1.3]	-0.32	1.5E-168	4.2E-168	-0.37	
K00568	2-polyprenyl-6-hydroxyphenyl methylase / 3-demethylubiquinone-9 3-methyltransferase [EC:2.1.1.222 2.1.1.64]	0.32	2.1E-168	5.9E-168	0.14	
K07303	isoquinoline 1-oxidoreductase subunit beta [EC:1.3.99.16]	-0.32	2.3E-168	6.5E-168	-0.36	yes
K10221	2-pyrone-4,6-dicarboxylate lactonase [EC:3.1.1.57]	-0.32	2.5E-168	7.0E-168	-0.37	yes
K00228	coproporphyrinogen III oxidase [EC:1.3.3.3]	0.32	2.6E-168	7.3E-168	0.14	
K00632	acetyl-CoA acyltransferase [EC:2.3.1.16]	0.32	2.8E-168	7.9E-168	0.14	
K00198	anaerobic carbon-monoxide dehydrogenase catalytic subunit [EC:1.2.7.4]	-0.32	3.3E-168	9.3E-168	-0.37	yes
K16242	phenol/toluene 2-monooxygenase (NADH) P3/A3 [EC:1.14.13.244 1.14.13.243]	-0.32	1.1E-167	3.1E-167	-0.37	yes
K01497	GTP cyclohydrolase II [EC:3.5.4.25]	0.32	1.6E-167	4.5E-167	0.14	
K01586	diaminopimelate decarboxylase [EC:4.1.1.20]	-0.32	2.1E-167	5.9E-167	-0.23	yes
K01236	maltooligosyltrehalose trehalohydrolase [EC:3.2.1.141]	-0.32	4.2E-167	1.2E-166	-0.36	yes
K10216	2-hydroxymuconate-semialdehyde hydrolase [EC:3.7.1.9]	-0.32	4.6E-167	1.3E-166	-0.37	
K08319	L-threonate 2-dehydrogenase [EC:1.1.1.411]	0.32	5.0E-167	1.4E-166	0.19	
K03763	DNA polymerase III subunit alpha, Gram-positive type [EC:2.7.7.7]	-0.32	6.9E-167	1.9E-166	-0.34	
K00695	sucrose synthase [EC:2.4.1.13]	-0.32	7.7E-167	2.1E-166	-0.37	
K04110	benzoate-CoA ligase [EC:6.2.1.25]	-0.32	1.6E-166	4.5E-166	-0.37	
K10715	two-component system, sensor histidine kinase RpfC [EC:2.7.13.3]	-0.32	2.4E-166	6.7E-166	-0.36	yes
K00138	aldehyde dehydrogenase [EC:1.2.1.-]	0.32	2.7E-166	7.5E-166	0.13	
K01483	ureidoglycolate lyase [EC:4.3.2.3]	0.32	5.0E-166	1.4E-165	0.15	
K16514	4-oxalomesaconate tautomerase [EC:5.3.2.8]	-0.32	6.1E-166	1.7E-165	-0.37	yes
K14029	methanol dehydrogenase (cytochrome c) subunit 2 [EC:1.1.2.7]	-0.32	6.8E-166	1.9E-165	-0.37	

Predictor	Description	Pearson's r	P	FDR-adjusted P	Spearman's rho	Associated with fractures (P<0.05)
K02028	polar amino acid transport system ATP-binding protein [EC:7.4.2.1]	-0.32	7.9E-166	2.2E-165	-0.33	
K07642	two-component system, OmpR family, sensor histidine kinase BaeS [EC:2.7.13.3]	0.32	9.1E-166	2.5E-165	0.16	
K01442	choloylglycine hydrolase [EC:3.5.1.24]	-0.32	9.5E-166	2.6E-165	-0.24	yes
K01884	cysteinyl-tRNA synthetase, unknown class [EC:6.1.1.16]	-0.32	1.2E-165	3.3E-165	-0.38	
K10793	D-proline reductase (dithiol) PrdA [EC:1.21.4.1]	-0.32	1.3E-165	3.6E-165	-0.39	
K00205	4Fe-4S ferredoxin	-0.32	1.4E-165	3.9E-165	-0.36	yes
K03055	DNA-directed RNA polymerase subunit K [EC:2.7.7.6]	-0.32	2.0E-165	5.5E-165	-0.37	
K12132	eukaryotic-like serine/threonine-protein kinase [EC:2.7.11.1]	-0.32	2.3E-165	6.3E-165	-0.32	
K00384	thioredoxin reductase (NADPH) [EC:1.8.1.9]	-0.32	3.5E-165	9.6E-165	-0.32	
K04100	protocatechuate 4,5-dioxygenase, alpha chain [EC:1.13.11.8]	-0.32	3.7E-165	1.0E-164	-0.36	yes
K01466	allantoinase [EC:3.5.2.5]	0.32	7.1E-165	2.0E-164	0.14	
K05522	endonuclease VIII [EC:3.2.2.- 4.2.99.18]	0.32	7.2E-165	2.0E-164	0.14	
K01458	N-formylglutamate deformylase [EC:3.5.1.68]	-0.32	8.2E-165	2.2E-164	-0.36	yes
K10012	undecaprenyl-phosphate 4-deoxy-4-formamido-L-arabinose transferase [EC:2.4.2.53]	0.32	9.3E-165	2.5E-164	0.13	
K00720	ceramide glucosyltransferase [EC:2.4.1.80]	-0.32	1.2E-164	3.3E-164	-0.36	yes
K14977	(S)-ureidoglycine aminohydrolase [EC:3.5.3.26]	0.32	1.9E-164	5.2E-164	0.14	
K00317	dimethylamine/trimethylamine dehydrogenase [EC:1.5.8.1 1.5.8.2]	-0.32	2.4E-164	6.6E-164	-0.37	yes
K03851	taurine-pyruvate aminotransferase [EC:2.6.1.77]	-0.32	2.7E-164	7.4E-164	-0.36	yes
K00931	glutamate 5-kinase [EC:2.7.2.11]	-0.32	2.9E-164	7.9E-164	-0.30	
K01253	microsomal epoxide hydrolase [EC:3.3.2.9]	-0.32	3.1E-164	8.4E-164	-0.36	
K01133	choline-sulfatase [EC:3.1.6.6]	-0.32	3.5E-164	9.5E-164	-0.36	yes
K12111	evolved beta-galactosidase subunit alpha [EC:3.2.1.23]	0.32	5.3E-164	1.4E-163	0.17	
K02433	aspartyl-tRNA(Asn)/glutamyl-tRNA(Gln) amidotransferase subunit A [EC:6.3.5.6 6.3.5.7]	-0.32	9.6E-164	2.6E-163	-0.33	
K13601	bacteriochlorophyllide d C-8(2)-methyltransferase [EC:2.1.1.332]	-0.32	1.2E-163	3.3E-163	-0.35	yes
K07302	isoquinoline 1-oxidoreductase subunit alpha [EC:1.3.99.16]	-0.32	1.3E-163	3.5E-163	-0.36	yes
K03828	putative acetyltransferase [EC:2.3.1.-]	0.32	1.3E-163	3.5E-163	0.18	
K04098	hydroxyquinol 1,2-dioxygenase [EC:1.13.11.37]	-0.32	1.4E-163	3.8E-163	-0.37	
K02303	uroporphyrin-III C-methyltransferase [EC:2.1.1.107]	-0.32	1.9E-163	5.1E-163	-0.37	yes
K06967	tRNA (adenine22-N1)-methyltransferase [EC:2.1.1.217]	-0.32	2.0E-163	5.4E-163	-0.33	
K01166	ribonuclease T2 [EC:4.6.1.19]	-0.32	2.5E-163	6.8E-163	-0.35	yes
K04108	4-hydroxybenzoyl-CoA reductase subunit alpha [EC:1.1.7.1]	-0.32	2.7E-163	7.3E-163	-0.36	yes
K01069	hydroxyacylglutathione hydrolase [EC:3.1.2.6]	0.32	3.6E-163	9.7E-163	0.13	
K10831	taurine transport system ATP-binding protein [EC:7.6.2.7]	0.32	5.3E-163	1.4E-162	0.17	
K04032	ethanolamine utilization cobalamin adenosyltransferase [EC:2.5.1.17]	0.32	7.7E-163	2.1E-162	0.14	
K11260	4Fe-4S ferredoxin	-0.32	7.9E-163	2.1E-162	-0.36	yes
K05917	sterol 14alpha-demethylase [EC:1.14.14.154 1.14.15.36]	-0.32	1.7E-162	4.6E-162	-0.36	
K01929	UDP-N-acetylmuramoyl-tripeptide--D-alanyl-D-alanine ligase [EC:6.3.2.10]	-0.32	2.5E-162	6.7E-162	-0.31	
K03782	catalase-peroxidase [EC:1.11.1.21]	0.32	4.2E-162	1.1E-161	0.13	
K13605	bacteriochlorophyll c synthase [EC:2.5.1.-]	-0.31	8.8E-162	2.4E-161	-0.35	yes
K04070	putative pyruvate formate lyase activating enzyme [EC:1.97.1.4]	-0.31	1.1E-161	2.9E-161	-0.33	
K11358	aspartate aminotransferase [EC:2.6.1.1]	-0.31	1.2E-161	3.2E-161	-0.31	
K01778	diaminopimelate epimerase [EC:5.1.1.7]	-0.31	1.7E-161	4.5E-161	-0.36	

Predictor	Description	Pearson's r	P	FDR-adjusted P	Spearman's rho	Associated with fractures (P<0.05)
K05979	2-phosphosulfolactate phosphatase [EC:3.1.3.71]	-0.31	4.6E-161	1.2E-160	-0.37	
K07697	two-component system, sporulation sensor kinase B [EC:2.7.13.3]	-0.31	6.9E-161	1.8E-160	-0.37	yes
K03224	ATP synthase in type III secretion protein N [EC:7.4.2.8]	0.31	9.9E-161	2.6E-160	0.14	
K01179	endoglucanase [EC:3.2.1.4]	-0.31	3.8E-160	1.0E-159	-0.29	yes
K07587	O-phosphoseryl-tRNA synthetase [EC:6.1.1.27]	-0.31	8.3E-160	2.2E-159	-0.37	
K01795	mannuronan 5-epimerase [EC:5.1.3.37]	-0.31	8.6E-160	2.3E-159	-0.36	yes
K00118	glucose-fructose oxidoreductase [EC:1.1.99.28]	-0.31	9.0E-160	2.4E-159	-0.34	yes
K02533	tRNA/rRNA methyltransferase [EC:2.1.1.-]	0.31	9.1E-160	2.4E-159	0.13	
K03930	putative tributyrin esterase [EC:3.1.1.-]	-0.31	1.0E-159	2.7E-159	-0.33	yes
K13659	2-beta-glucuronyltransferase [EC:2.4.1.264]	-0.31	2.4E-159	6.4E-159	-0.37	
K04114	benzoyl-CoA reductase subunit A [EC:1.3.7.8]	-0.31	2.7E-159	7.1E-159	-0.35	yes
K04115	benzoyl-CoA reductase subunit D [EC:1.3.7.8]	-0.31	2.7E-159	7.1E-159	-0.35	yes
K00103	L-gulonolactone oxidase [EC:1.1.3.8]	-0.31	4.1E-159	1.1E-158	-0.37	
K00134	glyceraldehyde 3-phosphate dehydrogenase (phosphorylating) [EC:1.2.1.12]	-0.31	6.6E-159	1.7E-158	-0.34	
K01028	3-oxoacid CoA-transferase subunit A [EC:2.8.3.5]	-0.31	8.9E-159	2.3E-158	-0.36	yes
K03379	cyclohexanone monooxygenase [EC:1.14.13.22]	-0.31	1.2E-158	3.2E-158	-0.35	
K00582	tetrahydromethanopterin S-methyltransferase subunit F [EC:2.1.1.86]	-0.31	1.6E-158	4.2E-158	-0.35	yes
K00933	creatine kinase [EC:2.7.3.2]	-0.31	3.1E-158	8.2E-158	-0.36	yes
K05927	quinone-reactive Ni/Fe-hydrogenase small subunit [EC:1.12.5.1]	-0.31	3.9E-158	1.0E-157	-0.36	yes
K10676	2,4-dichlorophenol 6-monooxygenase [EC:1.14.13.20]	-0.31	7.3E-158	1.9E-157	-0.36	
K01593	aromatic-L-amino-acid/L-tryptophan decarboxylase [EC:4.1.1.28 4.1.1.105]	-0.31	1.0E-157	2.6E-157	-0.37	yes
K01265	methionyl aminopeptidase [EC:3.4.11.18]	-0.31	1.0E-157	2.6E-157	-0.06	yes
K05988	dextranase [EC:3.2.1.11]	-0.31	1.5E-157	3.9E-157	-0.37	yes
K08587	clostripain [EC:3.4.22.8]	-0.31	2.6E-157	6.8E-157	-0.38	yes
K16619	phospholipase C / alpha-toxin [EC:3.1.4.3]	-0.31	2.6E-157	6.8E-157	-0.38	yes
K05578	NAD(P)H-quinone oxidoreductase subunit 6 [EC:7.1.1.2]	-0.31	3.1E-157	8.1E-157	-0.37	
K00193	acetyl-CoA decarbonylase/synthase, CODH/ACS complex subunit beta [EC:2.3.1.169]	-0.31	3.1E-157	8.1E-157	-0.37	
K01434	penicillin G amidase [EC:3.5.1.11]	-0.31	3.9E-157	1.0E-156	-0.36	yes
K03119	taurine dioxygenase [EC:1.14.11.17]	0.31	4.8E-157	1.3E-156	0.13	
K01087	trehalose 6-phosphate phosphatase [EC:3.1.3.12]	0.31	1.4E-156	3.6E-156	0.14	
K05964	holo-ACP synthase [EC:2.7.7.61]	0.31	2.0E-156	5.2E-156	0.17	
K01496	phosphoribosyl-AMP cyclohydrolase [EC:3.5.4.19]	-0.31	2.5E-156	6.5E-156	-0.32	
K00140	malonate-semialdehyde dehydrogenase (acetylating) / methylmalonate-semialdehyde dehydrogenase [EC:1.2.1.18 1.2.1.27]	-0.31	3.3E-156	8.6E-156	-0.35	yes
K13421	uridine monophosphate synthetase [EC:2.4.2.10 4.1.1.23]	-0.31	3.6E-156	9.3E-156	-0.37	
K14748	ethylbenzene dioxygenase subunit alpha [EC:1.14.12.-]	-0.31	4.1E-156	1.1E-155	-0.36	yes
K14749	ethylbenzene dioxygenase subunit beta [EC:1.14.12.-]	-0.31	4.1E-156	1.1E-155	-0.36	yes
K04113	benzoyl-CoA reductase subunit B [EC:1.3.7.8]	-0.31	4.5E-156	1.2E-155	-0.35	yes
K04112	benzoyl-CoA reductase subunit C [EC:1.3.7.8]	-0.31	4.9E-156	1.3E-155	-0.35	yes
K09251	putrescine aminotransferase [EC:2.6.1.82]	0.31	1.0E-155	2.6E-155	0.13	
K07246	tartrate dehydrogenase/decarboxylase / D-malate dehydrogenase [EC:1.1.1.93 4.1.1.73 1.1.1.83]	0.31	1.9E-155	4.9E-155	0.14	
K01167	ribonuclease T1 [EC:4.6.1.24]	-0.31	3.1E-155	8.0E-155	-0.37	
K03462	nicotinamide phosphoribosyltransferase [EC:2.4.2.12]	-0.31	4.2E-155	1.1E-154	-0.37	yes

Predictor	Description	Pearson's r	P	FDR-adjusted P	Spearman's rho	Associated with fractures (P<0.05)
K01263	NA	-0.31	9.4E-155	2.4E-154	-0.36	
K03725	archaea-specific helicase [EC:3.6.4.-]	-0.31	1.1E-154	2.8E-154	-0.35	yes
K03715	1,2-diacylglycerol 3-beta-galactosyltransferase [EC:2.4.1.46]	-0.31	1.5E-154	3.9E-154	-0.37	
K08260	adenosylcobinamide hydrolase [EC:3.5.1.90]	-0.31	2.3E-154	5.9E-154	-0.37	
K06034	sulfopyruvate decarboxylase subunit alpha [EC:4.1.1.79]	-0.31	3.2E-154	8.2E-154	-0.35	yes
K02840	UDP-D-galactose:(glucosyl)LPS alpha-1,6-D-galactosyltransferase [EC:2.4.1.-]	0.31	4.4E-154	1.1E-153	0.25	
K13005	O-antigen biosynthesis alpha-1,3-abequosyltransferase [EC:2.4.1.60]	-0.31	1.1E-153	2.8E-153	-0.35	
K05982	deoxyribonuclease V [EC:3.1.21.7]	0.31	1.2E-153	3.1E-153	0.14	
K04782	isochorismate pyruvate lyase [EC:4.2.99.21]	-0.31	9.3E-153	2.4E-152	-0.37	
K03476	L-ascorbate 6-phosphate lactonase [EC:3.1.1.-]	0.31	1.5E-152	3.8E-152	0.16	
K06868	Sep-tRNA:Cys-tRNA synthetase [EC:2.5.1.73]	-0.31	5.1E-152	1.3E-151	-0.37	
K00333	NADH-quinone oxidoreductase subunit D [EC:7.1.1.2]	-0.31	7.1E-152	1.8E-151	-0.31	
K06044	(1->4)-alpha-D-glucan 1-alpha-D-glucosylmutase [EC:5.4.99.15]	-0.31	9.5E-152	2.4E-151	-0.35	yes
K01637	isocitrate lyase [EC:4.1.3.1]	0.31	1.5E-151	3.8E-151	0.12	
K01591	orotidine-5'-phosphate decarboxylase [EC:4.1.1.23]	-0.30	1.8E-151	4.6E-151	-0.36	
K00188	2-oxoisovalerate ferredoxin oxidoreductase delta subunit [EC:1.2.7.7]	-0.30	3.0E-151	7.6E-151	-0.35	
K00299	FMN reductase [EC:1.5.1.38]	0.30	3.1E-151	7.9E-151	0.12	
K10219	2-hydroxy-4-carboxymuconate semialdehyde hemiacetal dehydrogenase [EC:1.1.1.312]	-0.30	5.1E-151	1.3E-150	-0.36	yes
K16319	anthranilate 1,2-dioxygenase large subunit [EC:1.14.12.1]	-0.30	6.8E-151	1.7E-150	-0.36	yes
K16320	anthranilate 1,2-dioxygenase small subunit [EC:1.14.12.1]	-0.30	9.1E-151	2.3E-150	-0.37	yes
K05576	NAD(P)H-quinone oxidoreductase subunit 4L [EC:7.1.1.2]	-0.30	1.0E-150	2.5E-150	-0.36	
K14268	5-aminovalerate/4-aminobutyrate aminotransferase [EC:2.6.1.48 2.6.1.19]	-0.30	1.1E-150	2.8E-150	-0.36	yes
K01095	phosphatidylglycerophosphatase A [EC:3.1.3.27]	0.30	1.7E-150	4.3E-150	0.12	
K04091	alkanesulfonate monooxygenase [EC:1.14.14.5 1.14.14.34]	0.30	1.9E-150	4.8E-150	0.12	
K01706	glucarate dehydratase [EC:4.2.1.40]	0.30	2.7E-150	6.8E-150	0.17	
K13380	NADH-quinone oxidoreductase subunit B/C/D [EC:7.1.1.2]	-0.30	2.9E-150	7.3E-150	-0.34	
K08298	L-carnitine CoA-transferase [EC:2.8.3.21]	0.30	3.9E-150	9.8E-150	0.19	
K13927	holo-ACP synthase / triphosphoribosyl-dephospho-CoA synthase [EC:2.7.7.61 2.4.2.52]	-0.30	4.2E-150	1.1E-149	-0.33	
K00035	D-galactose 1-dehydrogenase [EC:1.1.1.48]	-0.30	7.3E-150	1.8E-149	-0.36	
K00285	D-amino-acid dehydrogenase [EC:1.4.5.1]	0.30	8.8E-150	2.2E-149	0.11	
K03779	L(+)-tartrate dehydratase alpha subunit [EC:4.2.1.32]	0.30	1.4E-149	3.5E-149	0.19	
K01029	3-oxoacid CoA-transferase subunit B [EC:2.8.3.5]	-0.30	1.5E-149	3.8E-149	-0.36	yes
K08692	malate-CoA ligase subunit alpha [EC:6.2.1.9]	-0.30	1.5E-149	3.8E-149	-0.35	
K14067	malate-CoA ligase subunit beta [EC:6.2.1.9]	-0.30	1.5E-149	3.8E-149	-0.35	
K08644	tentoxilysin [EC:3.4.24.68]	-0.30	2.5E-149	6.3E-149	-0.35	
K00942	guanylate kinase [EC:2.7.4.8]	-0.30	2.6E-149	6.5E-149	-0.30	
K15780	bifunctional protein TilS/HprT [EC:6.3.4.19 2.4.2.8]	-0.30	4.1E-149	1.0E-148	-0.36	
K12960	5-methylthioadenosine/S-adenosylhomocysteine deaminase [EC:3.5.4.31 3.5.4.28]	-0.30	8.1E-149	2.0E-148	-0.29	
K12994	O-antigen biosynthesis alpha-1,3-mannosyltransferase [EC:2.4.1.349 2.4.1.-]	-0.30	9.4E-149	2.3E-148	-0.36	
K00332	NADH-quinone oxidoreductase subunit C [EC:7.1.1.2]	-0.30	1.6E-148	4.0E-148	-0.31	
K08096	GTP cyclohydrolase IIa [EC:3.5.4.29]	-0.30	2.0E-148	5.0E-148	-0.34	
K15357	N-formylmaleamate deformylase [EC:3.5.1.106]	-0.30	2.1E-148	5.2E-148	-0.37	

Predictor	Description	Pearson's r	P	FDR-adjusted P	Spearman's rho	Associated with fractures (P<0.05)
K00257	acyl-ACP dehydrogenase [EC:1.3.99.-]	-0.30	3.7E-148	9.2E-148	-0.35	
K01014	aryl sulfotransferase [EC:2.8.2.1]	-0.30	5.1E-148	1.3E-147	-0.35	yes
K16329	pseudouridylate synthase [EC:4.2.1.70]	0.30	1.2E-147	3.0E-147	0.12	
K13533	two-component system, sporulation sensor kinase E [EC:2.7.13.3]	-0.30	8.8E-147	2.2E-146	-0.37	yes
K01896	medium-chain acyl-CoA synthetase [EC:6.2.1.2]	-0.30	1.1E-146	2.7E-146	-0.36	yes
K16301	deferrochelatase/peroxidase EfeB [EC:1.11.1.-]	0.30	1.3E-146	3.2E-146	0.15	
K01267	aspartyl aminopeptidase [EC:3.4.11.21]	-0.30	1.6E-146	4.0E-146	-0.31	
K07468	putative ATP-dependent DNA ligase [EC:6.5.1.1]	-0.30	2.2E-146	5.4E-146	-0.37	
K03526	(E)-4-hydroxy-3-methylbut-2-enyl-diphosphate synthase [EC:1.17.7.1 1.17.7.3]	-0.30	2.6E-146	6.4E-146	-0.32	
K12252	arginine:pyruvate transaminase [EC:2.6.1.84]	-0.30	3.0E-146	7.4E-146	-0.35	
K00284	glutamate synthase (ferredoxin) [EC:1.4.7.1]	-0.30	3.0E-146	7.4E-146	-0.35	
K11629	two-component system, OmpR family, bacitracin resistance sensor histidine kinase BceS [EC:2.7.13.3]	-0.30	4.6E-146	1.1E-145	-0.37	yes
K14627	dehydratase [EC:4.2.1.-]	-0.30	8.4E-146	2.1E-145	-0.36	
K14266	tryptophan 7-halogenase [EC:1.14.19.9]	-0.30	1.6E-145	3.9E-145	-0.34	
K05597	glutamin-(asparagin-)ase [EC:3.5.1.38]	-0.30	2.9E-145	7.1E-145	-0.36	
K01055	3-oxoadipate enol-lactonase [EC:3.1.1.24]	-0.30	3.6E-145	8.8E-145	-0.35	yes
K01715	enoyl-CoA hydratase [EC:4.2.1.17]	-0.30	5.6E-145	1.4E-144	-0.29	
K08851	TP53 regulating kinase and related kinases [EC:2.7.11.1]	-0.30	2.6E-144	6.4E-144	-0.35	
K01684	galactonate dehydratase [EC:4.2.1.6]	0.30	3.5E-144	8.6E-144	0.14	
K08256	phosphatidyl-myo-inositol alpha-mannosyltransferase [EC:2.4.1.345]	-0.30	4.1E-144	1.0E-143	-0.32	yes
K01523	phosphoribosyl-ATP pyrophosphohydrolase [EC:3.6.1.31]	-0.30	4.5E-144	1.1E-143	-0.31	
K13543	uroporphyrinogen III methyltransferase / synthase [EC:2.1.1.107 4.2.1.75]	-0.30	6.9E-144	1.7E-143	-0.33	
K08659	dipeptidase [EC:3.4.-.-]	-0.30	7.4E-144	1.8E-143	-0.30	
K12987	alpha-1,6-rhamnosyltransferase [EC:2.4.1.-]	-0.30	9.8E-144	2.4E-143	-0.35	
K01577	oxalyl-CoA decarboxylase [EC:4.1.1.8]	0.30	1.5E-143	3.7E-143	0.17	
K02496	uroporphyrin-III C-methyltransferase [EC:2.1.1.107]	0.30	2.0E-143	4.9E-143	0.12	
K01001	UDP-N-acetylglucosamine--dolichyl-phosphate N-acetylglucosaminephosphotransferase [EC:2.7.8.15]	-0.30	5.8E-143	1.4E-142	-0.33	yes
K00887	undecaprenol kinase [EC:2.7.1.66]	-0.30	6.6E-143	1.6E-142	-0.31	
K04117	cyclohexanecarboxyl-CoA dehydrogenase [EC:1.3.99.-]	-0.30	6.8E-143	1.7E-142	-0.36	yes
K14633	ketoreductase RED2 [EC:1.1.1.-]	-0.30	7.1E-143	1.7E-142	-0.34	
K06215	pyridoxal 5'-phosphate synthase pdxS subunit [EC:4.3.3.6]	-0.30	1.0E-142	2.4E-142	-0.30	
K05991	endoglycosylceramidase [EC:3.2.1.123]	-0.30	1.0E-142	2.4E-142	-0.36	
K07310	Tat-targeted selenate reductase subunit YnfF [EC:1.97.1.9]	0.30	2.5E-142	6.1E-142	0.19	
K06027	vesicle-fusing ATPase [EC:3.6.4.6]	-0.30	3.9E-142	9.4E-142	-0.34	
K00170	pyruvate ferredoxin oxidoreductase beta subunit [EC:1.2.7.1]	-0.30	3.9E-142	9.4E-142	-0.33	
K15652	3-dehydroshikimate dehydratase [EC:4.2.1.118]	-0.30	6.3E-142	1.5E-141	-0.36	yes
K10811	thiamine pyridinylase [EC:2.5.1.2]	-0.30	8.4E-142	2.0E-141	-0.35	
K12661	L-rhamnonate dehydratase [EC:4.2.1.90]	0.30	8.6E-142	2.1E-141	0.16	
K03343	putrescine oxidase [EC:1.4.3.10]	-0.30	1.0E-141	2.4E-141	-0.34	
K07655	two-component system, OmpR family, sensor histidine kinase PrrB [EC:2.7.13.3]	-0.29	2.8E-141	6.7E-141	-0.34	
K01026	propionate CoA-transferase [EC:2.8.3.1]	-0.29	4.2E-141	1.0E-140	-0.33	
K00130	betaine-aldehyde dehydrogenase [EC:1.2.1.8]	0.29	4.3E-141	1.0E-140	0.11	

Predictor	Description	Pearson's r	P	FDR-adjusted P	Spearman's rho	Associated with fractures (P<0.05)
K01761	methionine-gamma-lyase [EC:4.4.1.11]	-0.29	5.3E-141	1.3E-140	-0.34	
K16650	galactofuranosylgalactofuranosylrhamnosyl-N-acetylglucosaminyl-diphospho-decaprenol beta-1,5/1,6-galactofuranosyltransferase [EC:2.4.1.288]	-0.29	6.6E-141	1.6E-140	-0.33	yes
K00363	nitrite reductase (NADH) small subunit [EC:1.7.1.15]	0.29	6.7E-141	1.6E-140	0.11	
K01784	UDP-glucose 4-epimerase [EC:5.1.3.2]	-0.29	8.0E-141	1.9E-140	-0.34	
K12138	hydrogenase-4 component C [EC:1.-.-.-]	0.29	8.7E-141	2.1E-140	0.19	
K06442	23S rRNA (cytidine1920-2'-O)/16S rRNA (cytidine1409-2'-O)-methyltransferase [EC:2.1.1.226 2.1.1.227]	-0.29	1.3E-140	3.1E-140	-0.31	
K00163	pyruvate dehydrogenase E1 component [EC:1.2.4.1]	0.29	1.8E-140	4.3E-140	0.11	
K07536	2-ketocyclohexanecarboxyl-CoA hydrolase [EC:3.1.2.-]	-0.29	1.9E-140	4.5E-140	-0.35	yes
K10672	glycine reductase complex component B subunit gamma [EC:1.21.4.2]	-0.29	2.0E-140	4.8E-140	-0.34	yes
K04109	4-hydroxybenzoyl-CoA reductase subunit beta [EC:1.1.7.1]	-0.29	4.8E-140	1.1E-139	-0.33	yes
K04107	4-hydroxybenzoyl-CoA reductase subunit gamma [EC:1.1.7.1]	-0.29	4.8E-140	1.1E-139	-0.33	yes
K00955	bifunctional enzyme CysN/CysC [EC:2.7.7.4 2.7.1.25]	-0.29	6.0E-140	1.4E-139	-0.30	
K10780	enoyl-[acyl-carrier protein] reductase III [EC:1.3.1.104]	-0.29	9.7E-140	2.3E-139	-0.36	yes
K01061	carboxymethylenebutenolidase [EC:3.1.1.45]	0.29	1.1E-139	2.6E-139	0.11	
K01608	tartronate-semialdehyde synthase [EC:4.1.1.47]	0.29	1.2E-139	2.8E-139	0.13	
K00655	1-acyl-sn-glycerol-3-phosphate acyltransferase [EC:2.3.1.51]	-0.29	1.2E-139	2.8E-139	-0.31	
K13687	arabinofuranosyltransferase [EC:2.4.2.-]	-0.29	1.5E-139	3.6E-139	-0.35	
K15256	tRNA (cmo5U34)-methyltransferase [EC:2.1.1.-]	0.29	2.0E-139	4.7E-139	0.11	
K13942	5,10-methenyltetrahydromethanopterin hydrogenase [EC:1.12.98.2]	-0.29	3.0E-139	7.1E-139	-0.33	yes
K00016	L-lactate dehydrogenase [EC:1.1.1.27]	-0.29	3.1E-139	7.3E-139	-0.30	
K00058	D-3-phosphoglycerate dehydrogenase / 2-oxoglutarate reductase [EC:1.1.1.95 1.1.1.399]	-0.29	5.7E-139	1.3E-138	-0.33	
K04105	4-hydroxybenzoate-CoA ligase [EC:6.2.1.27 6.2.1.25]	-0.29	6.0E-139	1.4E-138	-0.34	
K08068	UDP-N-acetylglucosamine 2-epimerase (hydrolysing) [EC:3.2.1.183]	-0.29	7.2E-139	1.7E-138	-0.35	yes
K12996	rhamnosyltransferase [EC:2.4.1.-]	-0.29	1.0E-138	2.4E-138	-0.31	yes
K05305	fucokinase [EC:2.7.1.52]	-0.29	1.2E-138	2.8E-138	-0.35	yes
K02337	DNA polymerase III subunit alpha [EC:2.7.7.7]	-0.29	8.2E-138	1.9E-137	-0.30	
K01569	oxalate decarboxylase [EC:4.1.1.2]	-0.29	1.0E-137	2.4E-137	-0.34	yes
K07549	benzoylsuccinyl-CoA thiolase BbsA subunit [EC:2.3.1.-]	-0.29	1.9E-137	4.5E-137	-0.33	
K07550	benzoylsuccinyl-CoA thiolase BbsB subunit [EC:2.3.1.-]	-0.29	1.9E-137	4.5E-137	-0.33	
K00050	NA	0.29	3.5E-137	8.2E-137	0.23	
K08095	cutinase [EC:3.1.1.74]	-0.29	6.7E-137	1.6E-136	-0.33	
K01851	salicylate biosynthesis isochorismate synthase [EC:5.4.4.2]	-0.29	6.9E-137	1.6E-136	-0.33	
K15228	methylamine dehydrogenase light chain [EC:1.4.9.1]	-0.29	7.8E-137	1.8E-136	-0.35	
K05884	L-2-hydroxycarboxylate dehydrogenase (NAD+) [EC:1.1.1.337]	-0.29	9.1E-137	2.1E-136	-0.32	yes
K00162	pyruvate dehydrogenase E1 component beta subunit [EC:1.2.4.1]	-0.29	1.2E-136	2.8E-136	-0.29	
K10218	4-hydroxy-4-methyl-2-oxoglutarate aldolase [EC:4.1.3.17]	-0.29	1.6E-136	3.7E-136	-0.35	yes
K00161	pyruvate dehydrogenase E1 component alpha subunit [EC:1.2.4.1]	-0.29	2.1E-136	4.9E-136	-0.29	
K00023	acetoacetyl-CoA reductase [EC:1.1.1.36]	-0.29	3.8E-136	8.9E-136	-0.34	yes
K13669	alpha-1,2-mannosyltransferase [EC:2.4.1.-]	-0.29	6.7E-136	1.6E-135	-0.34	
K01501	nitrilase [EC:3.5.5.1]	-0.29	1.3E-135	3.0E-135	-0.35	
K00966	mannose-1-phosphate guanylyltransferase [EC:2.7.7.13]	-0.29	2.2E-135	5.1E-135	-0.29	
K02774	galactitol PTS system EIIB component [EC:2.7.1.200]	0.29	2.6E-135	6.0E-135	0.13	

Predictor	Description	Pearson's r	P	FDR-adjusted P	Spearman's rho	Associated with fractures (P<0.05)
K14653	2-amino-5-formylamino-6-ribosylaminopyrimidin-4(3H)-one 5'-monophosphate deformylase [EC:3.5.1.102]	-0.29	2.7E-135	6.3E-135	-0.32	yes
K00697	trehalose 6-phosphate synthase [EC:2.4.1.15 2.4.1.347]	0.29	3.5E-135	8.1E-135	0.12	
K08681	pyridoxal 5'-phosphate synthase pdxT subunit [EC:4.3.3.6]	-0.29	5.1E-135	1.2E-134	-0.30	
K07544	benzylsuccinate CoA-transferase BbsF subunit [EC:2.8.3.15]	-0.29	5.4E-135	1.3E-134	-0.34	
K07545	(R)-benzylsuccinyl-CoA dehydrogenase [EC:1.3.8.3]	-0.29	5.8E-135	1.3E-134	-0.34	
K00262	glutamate dehydrogenase (NADP+) [EC:1.4.1.4]	-0.29	6.5E-135	1.5E-134	-0.11	
K00869	mevalonate kinase [EC:2.7.1.36]	-0.29	1.1E-134	2.5E-134	-0.31	yes
K07680	two-component system, NarL family, sensor histidine kinase ComP [EC:2.7.13.3]	-0.29	1.3E-134	3.0E-134	-0.34	yes
K05919	superoxide reductase [EC:1.15.1.2]	-0.29	1.4E-134	3.2E-134	-0.31	
K07548	2-[hydroxy(phenyl)methyl]-succinyl-CoA dehydrogenase BbsD subunit [EC:1.1.1.35]	-0.29	1.7E-134	3.9E-134	-0.33	
K02641	ferredoxin--NADP+ reductase [EC:1.18.1.2]	-0.29	2.5E-134	5.8E-134	-0.34	
K15784	N2-acetyl-L-2,4-diaminobutanoate deacetylase [EC:3.5.1.125]	-0.29	2.7E-134	6.2E-134	-0.35	
K11389	glyceraldehyde-3-phosphate dehydrogenase (ferredoxin) [EC:1.2.7.6]	-0.29	3.1E-134	7.1E-134	-0.36	yes
K01781	mandelate racemase [EC:5.1.2.2]	-0.29	3.7E-134	8.5E-134	-0.35	
K13954	alcohol dehydrogenase [EC:1.1.1.1]	0.29	5.2E-134	1.2E-133	0.10	
K01848	methylmalonyl-CoA mutase, N-terminal domain [EC:5.4.99.2]	-0.29	5.7E-134	1.3E-133	-0.34	
K06042	precorrin-8X/cobalt-precorrin-8 methylmutase [EC:5.4.99.61 5.4.99.60]	-0.29	6.3E-134	1.4E-133	-0.34	
K13657	alpha-1,3-mannosyltransferase [EC:2.4.1.252]	-0.29	7.3E-134	1.7E-133	-0.34	
K08355	arsenite oxidase small subunit [EC:1.20.2.1 1.20.9.1]	-0.29	1.1E-133	2.5E-133	-0.35	yes
K06121	glycerol dehydratase medium subunit [EC:4.2.1.30]	-0.29	1.1E-133	2.5E-133	-0.37	yes
K07543	benzylsuccinate CoA-transferase BbsE subunit [EC:2.8.3.15]	-0.29	1.7E-133	3.9E-133	-0.34	
K05518	phosphoserine phosphatase RsbX [EC:3.1.3.3]	-0.29	2.0E-133	4.6E-133	-0.35	yes
K15511	benzoyl-CoA 2,3-epoxidase subunit A [EC:1.14.13.208]	-0.29	2.5E-133	5.7E-133	-0.35	yes
K01432	arylformamidase [EC:3.5.1.9]	-0.29	5.2E-133	1.2E-132	-0.34	yes
K00466	tryptophan 2-monooxygenase [EC:1.13.12.3]	-0.29	1.5E-132	3.4E-132	-0.34	yes
K01537	P-type Ca2+ transporter type 2C [EC:7.2.2.10]	-0.29	1.7E-132	3.9E-132	-0.23	
K00756	pyrimidine-nucleoside phosphorylase [EC:2.4.2.2]	-0.29	1.8E-132	4.1E-132	-0.31	
K00799	glutathione S-transferase [EC:2.5.1.18]	0.29	2.2E-132	5.0E-132	0.10	
K11780	7,8-didemethyl-8-hydroxy-5-deazariboflavin synthase [EC:4.3.1.32]	-0.29	3.1E-132	7.1E-132	-0.35	yes
K05882	aryl-alcohol dehydrogenase (NADP+) [EC:1.1.1.91]	-0.29	3.4E-132	7.7E-132	-0.33	
K00146	phenylacetaldehyde dehydrogenase [EC:1.2.1.39]	0.29	3.5E-132	8.0E-132	0.11	
K05716	cyclic 2,3-diphosphoglycerate synthase [EC:6.5.1.9]	-0.29	5.5E-132	1.2E-131	-0.32	
K11611	meromycolic acid enoyl-[acyl-carrier-protein] reductase [EC:1.3.1.118]	-0.29	6.7E-132	1.5E-131	-0.33	
K15785	L-2,4-diaminobutyrate transaminase [EC:2.6.1.76]	-0.28	1.3E-131	2.9E-131	-0.35	
K04087	modulator of FtsH protease HflC	0.28	1.3E-131	2.9E-131	0.09	
K02773	galactitol PTS system EIIA component [EC:2.7.1.200]	0.28	1.7E-131	3.8E-131	0.13	
K00032	phosphogluconate 2-dehydrogenase [EC:1.1.1.43]	-0.28	5.1E-131	1.2E-130	-0.33	
K11386	arabinosyltransferase B [EC:2.4.2.-]	-0.28	6.4E-131	1.4E-130	-0.33	
K02304	precorrin-2 dehydrogenase / sirohydrochlorin ferrochelatase [EC:1.3.1.76 4.99.1.4]	-0.28	8.5E-131	1.9E-130	-0.33	
K15792	MurE/MurF fusion protein [EC:6.3.2.13 6.3.2.10]	-0.28	1.3E-130	2.9E-130	-0.33	yes
K06045	squalene-hopene/tetraprenyl-beta-curcumene cyclase [EC:5.4.99.17 4.2.1.129]	-0.28	2.7E-130	6.1E-130	-0.29	
K14630	two-component flavin-dependent monooxygenase [EC:1.14.14.-]	-0.28	4.4E-130	9.9E-130	-0.31	

Predictor	Description	Pearson's r	P	FDR-adjusted P	Spearman's rho	Associated with fractures (P<0.05)
K13787	geranylgeranyl diphosphate synthase, type I [EC:2.5.1.1 2.5.1.10 2.5.1.29]	-0.28	5.7E-130	1.3E-129	-0.29	
K15229	methylamine dehydrogenase heavy chain [EC:1.4.9.1]	-0.28	6.1E-130	1.4E-129	-0.35	
K12308	beta-galactosidase [EC:3.2.1.23]	-0.28	9.8E-130	2.2E-129	-0.20	
K03856	3-deoxy-7-phosphoheptulonate synthase [EC:2.5.1.54]	-0.28	1.4E-129	3.1E-129	-0.31	
K16558	succinoglycan biosynthesis protein ExoL [EC:2.-.-.-]	-0.28	2.7E-129	6.1E-129	-0.32	
K01436	amidohydrolase [EC:3.5.1.-]	-0.28	4.0E-129	9.0E-129	-0.28	
K04711	dihydroceramidase [EC:3.5.1.-]	-0.28	4.0E-129	9.0E-129	-0.33	yes
K14974	6-hydroxynicotinate 3-monooxygenase [EC:1.14.13.114]	-0.28	4.1E-129	9.2E-129	-0.35	
K01649	2-isopropylmalate synthase [EC:2.3.3.13]	-0.28	1.2E-128	2.7E-128	-0.33	
K01741	DNA-(apurinic or apyrimidinic site) lyase [EC:4.2.99.18]	-0.28	1.2E-128	2.7E-128	-0.31	
K15986	manganese-dependent inorganic pyrophosphatase [EC:3.6.1.1]	-0.28	3.1E-128	6.9E-128	-0.31	
K00881	allose kinase [EC:2.7.1.55]	0.28	7.1E-128	1.6E-127	0.17	
K00790	UDP-N-acetylglucosamine 1-carboxyvinyltransferase [EC:2.5.1.7]	-0.28	7.9E-128	1.8E-127	-0.36	
K00055	aryl-alcohol dehydrogenase [EC:1.1.1.90]	-0.28	8.0E-128	1.8E-127	-0.34	
K01641	hydroxymethylglutaryl-CoA synthase [EC:2.3.3.10]	-0.28	1.3E-127	2.9E-127	-0.31	yes
K00221	alkylmercury lyase [EC:4.99.1.2]	-0.28	1.4E-127	3.1E-127	-0.32	
K06375	stage 0 sporulation protein B (sporulation initiation phosphotransferase) [EC:2.7.-.-]	-0.28	6.5E-127	1.4E-126	-0.34	yes
K04787	mycobactin salicyl-AMP ligase [EC:6.3.2.-]	-0.28	7.5E-127	1.7E-126	-0.34	
K05921	5-oxopent-3-ene-1,2,5-tricarboxylate decarboxylase / 2-hydroxyhepta-2,4-diene-1,7-dioate isomerase [EC:4.1.1.68 5.3.3.-]	0.28	2.0E-126	4.4E-126	0.11	
K14338	cytochrome P450 / NADPH-cytochrome P450 reductase [EC:1.14.14.1 1.6.2.4]	-0.28	2.3E-126	5.1E-126	-0.34	yes
K10794	D-proline reductase (dithiol) PrdB [EC:1.21.4.1]	-0.28	6.1E-126	1.4E-125	-0.33	
K00121	S-(hydroxymethyl)glutathione dehydrogenase / alcohol dehydrogenase [EC:1.1.1.284 1.1.1.1]	0.28	7.8E-126	1.7E-125	0.09	
K13919	propanediol dehydratase medium subunit [EC:4.2.1.28]	0.28	1.4E-125	3.1E-125	0.11	
K01910	[citrate (pro-3S)-lyase] ligase [EC:6.2.1.22]	0.28	1.6E-125	3.5E-125	0.16	
K02509	2-oxo-hept-3-ene-1,7-dioate hydratase [EC:4.2.1.-]	0.28	2.7E-125	6.0E-125	0.12	
K15232	citryl-CoA synthetase large subunit [EC:6.2.1.18]	-0.28	3.0E-125	6.6E-125	-0.31	
K01259	proline iminopeptidase [EC:3.4.11.5]	-0.28	3.7E-125	8.2E-125	-0.28	
K13940	dihydroneopterin aldolase / 2-amino-4-hydroxy-6-hydroxymethyldihydropteridine diphosphokinase [EC:4.1.2.25 2.7.6.3]	-0.28	5.6E-125	1.2E-124	-0.28	
K15566	tRNA (adenine9-N1/guanine9-N1)-methyltransferase [EC:2.1.1.218 2.1.1.221]	-0.28	6.9E-125	1.5E-124	-0.30	
K04093	chorismate mutase [EC:5.4.99.5]	-0.28	1.0E-124	2.2E-124	-0.34	yes
K01885	glutamyl-tRNA synthetase [EC:6.1.1.17]	-0.28	1.4E-124	3.1E-124	-0.31	
K13498	indole-3-glycerol phosphate synthase / phosphoribosylanthranilate isomerase [EC:4.1.1.48 5.3.1.24]	0.28	2.1E-124	4.6E-124	0.17	
K05554	aromatase [EC:4.2.1.-]	-0.28	2.1E-124	4.6E-124	-0.33	
K08077	UDP-sugar diphosphatase [EC:3.6.1.45]	-0.28	2.8E-124	6.2E-124	-0.33	yes
K01462	peptide deformylase [EC:3.5.1.88]	-0.28	2.9E-124	6.4E-124	-0.30	
K13479	xanthine dehydrogenase FAD-binding subunit [EC:1.17.1.4]	0.28	4.4E-124	9.7E-124	0.09	
K06016	beta-ureidopropionase / N-carbamoyl-L-amino-acid hydrolase [EC:3.5.1.6 3.5.1.87]	-0.28	9.2E-124	2.0E-123	-0.31	
K03813	molybdenum transport protein [EC:2.4.2.-]	0.28	1.2E-123	2.6E-123	0.15	
K12420	ketoreductase [EC:1.1.1.-]	-0.28	1.7E-123	3.7E-123	-0.33	yes
K14379	tartrate-resistant acid phosphatase type 5 [EC:3.1.3.2]	-0.28	5.2E-123	1.1E-122	-0.32	
K00988	sulfate adenylyltransferase (ADP) / ATP adenylyltransferase [EC:2.7.7.5 2.7.7.53]	-0.28	5.3E-123	1.2E-122	-0.34	
K04712	sphingolipid 4-desaturase/C4-monooxygenase [EC:1.14.19.17 1.14.18.5]	-0.28	6.1E-123	1.3E-122	-0.33	

Predictor	Description	Pearson's r	P	FDR-adjusted P	Spearman's rho	Associated with fractures (P<0.05)
K01810	glucose-6-phosphate isomerase [EC:5.3.1.9]	-0.28	9.8E-123	2.1E-122	-0.29	
K01060	cephalosporin-C deacetylase [EC:3.1.1.41]	-0.28	9.9E-123	2.2E-122	-0.32	yes
K00366	ferredoxin-nitrite reductase [EC:1.7.7.1]	-0.28	1.0E-122	2.2E-122	-0.31	
K08081	tropinone reductase I [EC:1.1.1.206]	-0.28	1.8E-122	3.9E-122	-0.32	yes
K01342	subtilisin [EC:3.4.21.62]	-0.28	1.9E-122	4.1E-122	-0.34	yes
K14658	nodulation protein A [EC:2.3.1.-]	-0.27	2.0E-122	4.4E-122	-0.32	yes
K01849	methylmalonyl-CoA mutase, C-terminal domain [EC:5.4.99.2]	-0.27	2.3E-122	5.0E-122	-0.33	
K00151	5-carboxymethyl-2-hydroxymuconic-semialdehyde dehydrogenase [EC:1.2.1.60]	0.27	3.4E-122	7.4E-122	0.10	
K08282	non-specific serine/threonine protein kinase [EC:2.7.11.1]	-0.27	5.3E-122	1.2E-121	-0.28	
K00544	betaine-homocysteine S-methyltransferase [EC:2.1.1.5]	-0.27	6.0E-122	1.3E-121	-0.29	yes
K13874	L-arabinonolactonase [EC:3.1.1.15]	-0.27	7.1E-122	1.5E-121	-0.30	
K11915	serine/threonine protein phosphatase Stp1 [EC:3.1.3.16]	-0.27	9.2E-122	2.0E-121	-0.33	yes
K00801	farnesyl-diphosphate farnesyltransferase [EC:2.5.1.21]	-0.27	9.5E-122	2.1E-121	-0.28	
K05363	serine/alanine adding enzyme [EC:2.3.2.10]	-0.27	9.8E-122	2.1E-121	-0.28	
K08722	5'-deoxynucleotidase [EC:3.1.3.89]	0.27	1.1E-121	2.4E-121	0.09	
K00087	xanthine dehydrogenase molybdenum-binding subunit [EC:1.17.1.4]	0.27	1.1E-121	2.4E-121	0.09	
K09761	16S rRNA (uracil1498-N3)-methyltransferase [EC:2.1.1.193]	-0.27	2.7E-121	5.8E-121	-0.28	
K10621	2,3-dihydroxy-p-cumate/2,3-dihydroxybenzoate 3,4-dioxygenase [EC:1.13.11.- 1.13.11.14]	-0.27	3.9E-121	8.4E-121	-0.32	
K00276	primary-amine oxidase [EC:1.4.3.21]	0.27	4.0E-121	8.6E-121	0.11	
K02826	cytochrome aa3-600 menaquinol oxidase subunit II [EC:7.1.1.5]	-0.27	7.7E-121	1.7E-120	-0.34	yes
K16190	glucuronokinase [EC:2.7.1.43]	-0.27	1.2E-120	2.6E-120	-0.32	
K09001	anhydro-N-acetylmuramic acid kinase [EC:2.7.1.170]	0.27	1.4E-120	3.0E-120	0.10	
K03602	exodeoxyribonuclease VII small subunit [EC:3.1.11.6]	-0.27	3.8E-120	8.2E-120	-0.35	
K04099	gallate dioxygenase [EC:1.13.11.57]	-0.27	9.7E-120	2.1E-119	-0.34	
K01865	(hydroxyamino)benzene mutase [EC:5.4.4.1]	-0.27	1.0E-119	2.1E-119	-0.32	yes
K03780	L(+)-tartrate dehydratase beta subunit [EC:4.2.1.32]	0.27	1.4E-119	3.0E-119	0.19	
K16043	scyllo-inositol 2-dehydrogenase (NAD+) [EC:1.1.1.370]	-0.27	1.6E-119	3.4E-119	-0.34	
K04088	modulator of FtsH protease HflK	0.27	2.0E-119	4.3E-119	0.08	
K13660	beta-1,4-glucosyltransferase [EC:2.4.1.-]	-0.27	2.8E-119	6.0E-119	-0.32	
K10815	hydrogen cyanide synthase HcnB [EC:1.4.99.5]	-0.27	8.8E-119	1.9E-118	-0.33	
K10816	hydrogen cyanide synthase HcnC [EC:1.4.99.5]	-0.27	8.8E-119	1.9E-118	-0.33	
K00862	erythritol kinase (D-erythritol 1-phosphate-forming) [EC:2.7.1.215]	-0.27	1.6E-118	3.4E-118	-0.33	
K00764	amidophosphoribosyltransferase [EC:2.4.2.14]	-0.27	2.2E-118	4.7E-118	0.00	yes
K02119	V/A-type H+/Na+-transporting ATPase subunit C	-0.27	2.5E-118	5.3E-118	-0.31	
K15786	aspartate-semialdehyde dehydrogenase [EC:1.2.1.-]	-0.27	2.8E-118	6.0E-118	-0.33	
K07749	formyl-CoA transferase [EC:2.8.3.16]	0.27	4.0E-117	8.5E-117	0.12	
K01640	hydroxymethylglutaryl-CoA lyase [EC:4.1.3.4]	-0.27	5.0E-117	1.1E-116	-0.33	
K01027	3-oxoacid CoA-transferase [EC:2.8.3.5]	-0.27	9.4E-117	2.0E-116	-0.32	yes
K00635	diacylglycerol O-acyltransferase / wax synthase [EC:2.3.1.20 2.3.1.75]	-0.27	1.3E-116	2.8E-116	-0.30	
K00526	ribonucleoside-diphosphate reductase beta chain [EC:1.17.4.1]	0.27	4.4E-116	9.3E-116	0.37	
K02301	NA	0.27	9.5E-116	2.0E-115	0.08	
K15897	UDP-2,4-diacetamido-2,4,6-trideoxy-beta-L-altropyranose hydrolase [EC:3.6.1.57]	-0.27	1.1E-115	2.3E-115	-0.33	

Predictor	Description	Pearson's r	P	FDR-adjusted P	Spearman's rho	Associated with fractures (P<0.05)
K10944	methane/ammonia monooxygenase subunit A [EC:1.14.18.3 1.14.99.39]	-0.27	2.0E-115	4.2E-115	-0.30	
K15320	6-methylsalicylic acid synthase [EC:2.3.1.165]	-0.27	2.2E-115	4.7E-115	-0.30	
K14215	trans,polycis-decaprenyl diphosphate synthase [EC:2.5.1.86]	-0.27	3.5E-115	7.4E-115	-0.30	
K01215	glucan 1,6-alpha-glucosidase [EC:3.2.1.70]	-0.27	4.0E-115	8.5E-115	-0.29	
K05999	xanthomonalisin [EC:3.4.21.101]	-0.27	4.5E-115	9.5E-115	-0.32	
K01699	propanediol dehydratase large subunit [EC:4.2.1.28]	0.27	4.5E-115	9.5E-115	0.09	
K02082	D-galactosamine 6-phosphate deaminase/isomerase [EC:3.5.99.-]	0.27	1.6E-114	3.4E-114	0.13	
K12526	bifunctional diaminopimelate decarboxylase / aspartate kinase [EC:4.1.1.20 2.7.2.4]	-0.27	1.9E-114	4.0E-114	-0.33	yes
K00245	fumarate reductase iron-sulfur subunit [EC:1.3.5.4]	0.27	2.1E-114	4.4E-114	0.14	
K15895	UDP-4-amino-4,6-dideoxy-L-N-acetyl-beta-L-altrosamine transaminase [EC:2.6.1.92]	-0.27	2.6E-114	5.5E-114	-0.31	
K07406	alpha-galactosidase [EC:3.2.1.22]	0.27	2.7E-114	5.7E-114	0.07	
K07155	quercetin 2,3-dioxygenase [EC:1.13.11.24]	-0.27	3.1E-114	6.5E-114	-0.33	yes
K15904	bifunctional N6-L-threonylcarbamoyladenine synthase / protein kinase Bud32 [EC:2.3.1.234 2.7.11.1]	-0.27	4.4E-114	9.2E-114	-0.33	
K01137	N-acetylglucosamine-6-sulfatase [EC:3.1.6.14]	-0.27	4.8E-114	1.0E-113	-0.28	
K03040	DNA-directed RNA polymerase subunit alpha [EC:2.7.7.6]	-0.27	8.7E-114	1.8E-113	-0.29	
K10562	rhamnose transport system ATP-binding protein [EC:7.5.2.-]	-0.27	1.2E-113	2.5E-113	-0.33	yes
K00004	(R,R)-butanediol dehydrogenase / meso-butanediol dehydrogenase / diacetyl reductase [EC:1.1.1.4 1.1.1.- 1.1.1.303]	-0.26	2.6E-113	5.4E-113	-0.30	
K01467	beta-lactamase class C [EC:3.5.2.6]	0.26	2.9E-113	6.1E-113	0.16	
K13920	propanediol dehydratase small subunit [EC:4.2.1.28]	0.26	4.7E-113	9.8E-113	0.09	
K00527	ribonucleoside-triphosphate reductase (thioredoxin) [EC:1.17.4.2]	-0.26	8.8E-113	1.8E-112	-0.09	yes
K01703	3-isopropylmalate/(R)-2-methylmalate dehydratase large subunit [EC:4.2.1.33 4.2.1.35]	-0.26	9.2E-113	1.9E-112	-0.26	
K01826	5-carboxymethyl-2-hydroxymuconate isomerase [EC:5.3.3.10]	0.26	1.8E-112	3.8E-112	0.08	
K05556	ketoreductase RED1 [EC:1.1.1.-]	-0.26	2.8E-112	5.8E-112	-0.30	
K13663	acyltransferase [EC:2.3.1.-]	-0.26	3.8E-112	7.9E-112	-0.31	yes
K13058	mannosylfructose-phosphate synthase [EC:2.4.1.246]	-0.26	3.9E-112	8.1E-112	-0.29	
K13020	UDP-N-acetyl-2-amino-2-deoxyglucuronate dehydrogenase [EC:1.1.1.335]	-0.26	4.0E-112	8.3E-112	-0.31	
K02828	cytochrome aa3-600 menaquinol oxidase subunit III [EC:7.1.1.5]	-0.26	4.8E-112	1.0E-111	-0.33	yes
K00057	glycerol-3-phosphate dehydrogenase (NAD(P)+) [EC:1.1.1.94]	-0.26	5.5E-112	1.1E-111	-0.28	
K06970	23S rRNA (adenine1618-N6)-methyltransferase [EC:2.1.1.181]	0.26	6.3E-112	1.3E-111	0.23	yes
K00483	4-hydroxyphenylacetate 3-monooxygenase [EC:1.14.14.9]	0.26	9.4E-112	2.0E-111	0.09	
K02825	pyrimidine operon attenuation protein / uracil phosphoribosyltransferase [EC:2.4.2.9]	-0.26	1.1E-111	2.3E-111	-0.28	
K03929	para-nitrobenzyl esterase [EC:3.1.1.-]	-0.26	1.5E-111	3.1E-111	-0.27	
K14155	cysteine-S-conjugate beta-lyase [EC:4.4.1.13]	-0.26	2.1E-111	4.4E-111	-0.17	
K00053	ketol-acid reductoisomerase [EC:1.1.1.86]	-0.26	2.2E-111	4.6E-111	-0.30	
K00841	aminotransferase [EC:2.6.1.-]	-0.26	3.1E-111	6.4E-111	-0.30	
K00493	xanthocillin biosynthesis cytochrome P450 monooxygenase [EC:1.14.-.-]	-0.26	6.7E-111	1.4E-110	-0.31	
K14153	hydroxymethylpyrimidine kinase / phosphomethylpyrimidine kinase / thiamine-phosphate diphosphorylase [EC:2.7.1.49 2.7.4.7 2.5.1.3]	-0.26	7.3E-111	1.5E-110	-0.32	
K05895	precorrin-6A/cobalt-precorrin-6A reductase [EC:1.3.1.54 1.3.1.106]	-0.26	2.1E-110	4.3E-110	-0.32	
K01601	ribulose-bisphosphate carboxylase large chain [EC:4.1.1.39]	-0.26	2.3E-110	4.7E-110	-0.32	
K13776	citronellyl-CoA synthetase [EC:6.2.1.-]	-0.26	2.5E-110	5.2E-110	-0.31	
K01426	amidase [EC:3.5.1.4]	-0.26	2.8E-110	5.8E-110	-0.29	
K15734	all-trans-retinol dehydrogenase (NAD+) [EC:1.1.1.105]	-0.26	4.1E-110	8.4E-110	-0.28	

Predictor	Description	Pearson's r	P	FDR-adjusted P	Spearman's rho	Associated with fractures (P<0.05)
K01850	chorismate mutase [EC:5.4.99.5]	-0.26	4.1E-110	8.4E-110	-0.28	
K16318	tRNA (guanine6-N2)-methyltransferase [EC:2.1.1.256]	-0.26	5.2E-110	1.1E-109	-0.29	
K02829	cytochrome aa3-600 menaquinol oxidase subunit IV [EC:7.1.1.5]	-0.26	6.8E-110	1.4E-109	-0.33	yes
K02193	heme exporter protein A [EC:7.6.2.5]	0.26	7.1E-110	1.5E-109	0.09	
K07547	2-[hydroxy(phenyl)methyl]-succinyl-CoA dehydrogenase BbsC subunit [EC:1.1.1.35]	-0.26	9.4E-110	1.9E-109	-0.29	
K15761	toluene monooxygenase system protein B [EC:1.14.13.236 1.14.13.-]	-0.26	1.2E-109	2.5E-109	-0.30	yes
K00638	chloramphenicol O-acetyltransferase type B [EC:2.3.1.28]	0.26	4.4E-109	9.0E-109	0.10	
K12990	rhamnosyltransferase [EC:2.4.1.-]	-0.26	4.8E-109	9.8E-109	-0.32	
K01185	lysozyme [EC:3.2.1.17]	0.26	7.7E-109	1.6E-108	0.21	
K02469	DNA gyrase subunit A [EC:5.6.2.2]	-0.26	1.2E-108	2.5E-108	-0.28	
K12136	hydrogenase-4 component A [EC:1.-.-.-]	0.26	1.7E-108	3.5E-108	0.14	
K12297	23S rRNA (guanine2069-N7)-methyltransferase / 23S rRNA (guanine2445-N2)-methyltransferase [EC:2.1.1.264 2.1.1.173]	0.26	2.3E-108	4.7E-108	0.13	
K13677	1,2-diacylglycerol-3-alpha-glucose alpha-1,2-glucosyltransferase [EC:2.4.1.208]	-0.26	3.0E-108	6.1E-108	-0.28	
K14940	gamma-F420-2:alpha-L-glutamate ligase [EC:6.3.2.32]	-0.26	3.0E-108	6.1E-108	-0.32	
K01581	ornithine decarboxylase [EC:4.1.1.17]	0.26	3.3E-108	6.7E-108	0.14	
K11912	serine/threonine-protein kinase PpkA [EC:2.7.11.1]	-0.26	4.9E-108	1.0E-107	-0.31	
K05341	amylosucrase [EC:2.4.1.4]	-0.26	5.2E-108	1.1E-107	-0.28	yes
K00298	N5-(carboxyethyl)ornithine synthase [EC:1.5.1.24]	-0.26	7.4E-108	1.5E-107	-0.29	
K03438	16S rRNA (cytosine1402-N4)-methyltransferase [EC:2.1.1.199]	-0.26	1.1E-107	2.2E-107	-0.31	yes
K00772	5'-methylthioadenosine phosphorylase [EC:2.4.2.28]	-0.26	1.4E-107	2.8E-107	-0.30	
K14534	4-hydroxybutyryl-CoA dehydratase / vinylacetyl-CoA-Delta-isomerase [EC:4.2.1.120 5.3.3.3]	-0.26	1.4E-107	2.8E-107	-0.24	
K15764	toluene monooxygenase system protein E [EC:1.14.13.236 1.14.13.-]	-0.26	2.7E-107	5.5E-107	-0.31	yes
K01922	phosphopantothenate---cysteine ligase (ATP) [EC:6.3.2.51]	-0.26	2.8E-107	5.7E-107	-0.26	
K01669	deoxyribodipyrimidine photo-lyase [EC:4.1.99.3]	0.26	3.3E-107	6.7E-107	0.07	
K01779	aspartate racemase [EC:5.1.1.13]	-0.26	4.7E-107	9.5E-107	-0.27	
K00219	2,4-dienoyl-CoA reductase (NADPH2) [EC:1.3.1.34]	0.26	4.7E-107	9.5E-107	0.13	
K07407	alpha-galactosidase [EC:3.2.1.22]	-0.26	5.5E-107	1.1E-106	-0.23	
K11610	beta-ketoacyl ACP reductase [EC:1.1.1.100]	-0.26	9.2E-107	1.9E-106	-0.30	
K00917	tagatose 6-phosphate kinase [EC:2.7.1.144]	-0.26	2.6E-106	5.3E-106	-0.29	
K01303	acylaminoacyl-peptidase [EC:3.4.19.1]	-0.26	3.2E-106	6.5E-106	-0.28	yes
K03707	thiaminase (transcriptional activator TenA) [EC:3.5.99.2]	-0.26	3.7E-106	7.5E-106	-0.26	
K00344	NADPH:quinone reductase [EC:1.6.5.5]	0.26	3.9E-106	7.9E-106	0.07	
K01817	phosphoribosylanthranilate isomerase [EC:5.3.1.24]	-0.26	1.1E-105	2.2E-105	-0.27	
K00428	cytochrome c peroxidase [EC:1.11.1.5]	0.26	2.0E-105	4.0E-105	0.21	yes
K00433	non-heme chloroperoxidase [EC:1.11.1.10]	-0.26	2.0E-105	4.0E-105	-0.28	yes
K01612	vanillate/4-hydroxybenzoate decarboxylase subunit C [EC:4.1.1.- 4.1.1.61]	-0.26	2.3E-105	4.6E-105	-0.28	
K02567	nitrate reductase (cytochrome) [EC:1.9.6.1]	0.26	2.3E-105	4.6E-105	0.13	
K07652	two-component system, OmpR family, sensor histidine kinase VicK [EC:2.7.13.3]	-0.26	2.9E-105	5.8E-105	-0.28	
K01621	xylulose-5-phosphate/fructose-6-phosphate phosphoketolase [EC:4.1.2.9 4.1.2.22]	-0.26	5.3E-105	1.1E-104	-0.26	
K00675	N-hydroxyarylamine O-acetyltransferase [EC:2.3.1.118]	0.26	5.9E-105	1.2E-104	0.10	
K01459	N-carbamoyl-D-amino-acid hydrolase [EC:3.5.1.77]	-0.25	7.3E-105	1.5E-104	-0.28	
K11177	xanthine dehydrogenase YagR molybdenum-binding subunit [EC:1.17.1.4]	0.25	8.0E-105	1.6E-104	0.07	

Predictor	Description	Pearson's r	P	FDR-adjusted P	Spearman's rho	Associated with fractures (P<0.05)
K03657	DNA helicase II / ATP-dependent DNA helicase PcrA [EC:3.6.4.12]	-0.25	9.4E-105	1.9E-104	-0.33	
K11210	metallothiol transferase [EC:2.5.1.-]	-0.25	1.1E-104	2.2E-104	-0.32	yes
K01227	mannosyl-glycoprotein endo-beta-N-acetylglucosaminidase [EC:3.2.1.96]	-0.25	2.3E-104	4.6E-104	-0.28	
K00064	D-threo-aldose 1-dehydrogenase [EC:1.1.1.122]	0.25	3.2E-104	6.4E-104	0.08	
K15918	D-glycerate 3-kinase [EC:2.7.1.31]	-0.25	3.4E-104	6.8E-104	-0.33	
K02334	DNA polymerase bacteriophage-type [EC:2.7.7.7]	-0.25	3.7E-104	7.4E-104	-0.26	
K15450	tRNA wybutosine-synthesizing protein 3 [EC:2.1.1.282]	-0.25	4.1E-104	8.2E-104	-0.27	
K01799	maleate isomerase [EC:5.2.1.1]	-0.25	6.3E-104	1.3E-103	-0.32	
K00464	all-trans-8'-apo-beta-carotenal 15,15'-oxygenase [EC:1.13.11.75]	-0.25	1.4E-103	2.8E-103	-0.30	
K07442	tRNA (adenine57-N1/adenine58-N1)-methyltransferase catalytic subunit [EC:2.1.1.219 2.1.1.220]	-0.25	1.5E-103	3.0E-103	-0.26	
K01261	glutamyl aminopeptidase [EC:3.4.11.7]	-0.25	1.9E-103	3.8E-103	-0.26	
K01829	disulfide reductase [EC:1.8.-.-]	-0.25	2.5E-103	5.0E-103	-0.31	yes
K13542	uroporphyrinogen III methyltransferase / synthase [EC:2.1.1.107 4.2.1.75]	-0.25	3.4E-102	6.7E-102	-0.27	
K00543	acetylserotonin O-methyltransferase [EC:2.1.1.4]	-0.25	4.7E-102	9.3E-102	-0.31	
K10213	ribosylpyrimidine nucleosidase [EC:3.2.2.8]	0.25	4.9E-102	9.7E-102	0.08	
K01598	phosphopantothenoylcysteine decarboxylase [EC:4.1.1.36]	-0.25	9.1E-102	1.8E-101	-0.25	
K05985	ribonuclease M5 [EC:3.1.26.8]	-0.25	1.5E-101	3.0E-101	-0.28	
K01040	glutaconate CoA-transferase, subunit B [EC:2.8.3.12]	-0.25	2.4E-101	4.7E-101	-0.32	
K05913	2,4'-dihydroxyacetophenone dioxygenase [EC:1.13.11.41]	-0.25	3.6E-101	7.1E-101	-0.30	
K00217	maleylacetate reductase [EC:1.3.1.32]	-0.25	4.6E-101	9.1E-101	-0.30	
K15054	(S)-mandelate dehydrogenase [EC:1.1.99.31]	-0.25	5.2E-101	1.0E-100	-0.31	
K03388	heterodisulfide reductase subunit A2 [EC:1.8.7.3 1.8.98.4 1.8.98.5 1.8.98.6]	-0.25	6.5E-101	1.3E-100	-0.22	
K01713	cyclohexadienyl dehydratase [EC:4.2.1.51 4.2.1.91]	-0.25	7.1E-101	1.4E-100	-0.30	
K13798	DNA-directed RNA polymerase subunit B [EC:2.7.7.6]	-0.25	9.0E-101	1.8E-100	-0.29	
K11178	xanthine dehydrogenase YagS FAD-binding subunit [EC:1.17.1.4]	0.25	1.1E-100	2.2E-100	0.06	
K15781	putative phosphoserine phosphatase / 1-acylglycerol-3-phosphate O-acyltransferase [EC:3.1.3.3 2.3.1.51]	-0.25	1.1E-100	2.2E-100	-0.31	yes
K06441	ferredoxin hydrogenase gamma subunit [EC:1.12.7.2]	-0.25	1.6E-100	3.1E-100	-0.30	
K07272	rhamnosyltransferase [EC:2.4.1.-]	-0.25	2.9E-100	5.7E-100	-0.26	
K11609	beta-ketoacyl ACP synthase [EC:2.3.1.293 2.3.1.294]	-0.25	3.6E-100	7.1E-100	-0.29	
K16515	4-oxalomesaconate hydratase [EC:4.2.1.83]	-0.25	4.1E-100	8.0E-100	-0.32	yes
K00633	galactoside O-acetyltransferase [EC:2.3.1.18]	0.25	4.4E-100	8.6E-100	0.22	yes
K01909	long-chain-fatty-acid--[acyl-carrier-protein] ligase [EC:6.2.1.20]	-0.25	5.2E-100	1.0E-99	-0.28	
K00011	aldehyde reductase [EC:1.1.1.21]	-0.25	5.3E-100	1.0E-99	-0.27	yes
K00256	NA	-0.25	5.3E-100	1.0E-99	-0.27	yes
K05364	penicillin-binding protein A	-0.25	7.5E-100	1.5E-99	-0.25	
K02618	oxepin-CoA hydrolase / 3-oxo-5,6-dehydrosuberyl-CoA semialdehyde dehydrogenase [EC:3.3.2.12 1.2.1.91]	0.25	1.1E-99	2.2E-99	0.06	
K00511	squalene monooxygenase [EC:1.14.14.17]	-0.25	2.0E-99	3.9E-99	-0.31	
K15755	2'-aminobiphenyl-2,3-diol 1,2-dioxygenase, large subunit [EC:1.13.11.-]	-0.25	2.6E-99	5.1E-99	-0.29	yes
K00315	dimethylglycine dehydrogenase [EC:1.5.8.4]	-0.25	4.3E-99	8.4E-99	-0.28	
K10713	5,6,7,8-tetrahydromethanopterin hydro-lyase [EC:4.2.1.147]	-0.25	2.2E-98	4.3E-98	-0.32	
K08690	cis-2,3-dihydrobiphenyl-2,3-diol dehydrogenase [EC:1.3.1.56]	-0.25	2.4E-98	4.7E-98	-0.29	
K16147	starch synthase (maltosyl-transferring) [EC:2.4.99.16]	-0.25	5.2E-98	1.0E-97	-0.25	

Predictor	Description	Pearson's r	P	FDR-adjusted P	Spearman's rho	Associated with fractures (P<0.05)
K15896	UDP-4-amino-4,6-dideoxy-N-acetyl-beta-L-altrosamine N-acetyltransferase [EC:2.3.1.202]	-0.25	8.0E-98	1.6E-97	-0.28	
K00426	cytochrome bd ubiquinol oxidase subunit II [EC:7.1.1.7]	0.25	9.0E-98	1.8E-97	0.31	yes
K00573	protein-L-isoaspartate(D-aspartate) O-methyltransferase [EC:2.1.1.77]	0.25	1.0E-97	1.9E-97	0.06	
K00886	polyphosphate glucokinase [EC:2.7.1.63]	-0.25	1.7E-97	3.3E-97	-0.25	
K01210	glucan 1,3-beta-glucosidase [EC:3.2.1.58]	-0.25	2.4E-97	4.7E-97	-0.25	
K01751	diaminopropionate ammonia-lyase [EC:4.3.1.15]	0.25	5.5E-97	1.1E-96	0.09	
K13571	proteasome accessory factor A [EC:6.3.1.19]	-0.25	7.2E-97	1.4E-96	-0.25	
K15754	2'-aminobiphenyl-2,3-diol 1,2-dioxygenase, small subunit [EC:1.13.11.-]	-0.25	7.6E-97	1.5E-96	-0.29	yes
K11385	arabinosyltransferase A [EC:2.4.2.-]	-0.25	8.1E-97	1.6E-96	-0.28	
K14165	atypical dual specificity phosphatase [EC:3.1.3.16 3.1.3.48]	-0.24	9.3E-97	1.8E-96	-0.30	
K05296	3(or 17)beta-hydroxysteroid dehydrogenase [EC:1.1.1.51]	-0.24	1.1E-96	2.1E-96	-0.31	
K01971	bifunctional non-homologous end joining protein LigD [EC:6.5.1.1]	-0.24	1.3E-96	2.5E-96	-0.29	
K03738	aldehyde:ferredoxin oxidoreductase [EC:1.2.7.5]	-0.24	2.1E-96	4.1E-96	-0.30	
K05275	pyridoxine 4-dehydrogenase [EC:1.1.1.65]	-0.24	2.7E-96	5.2E-96	-0.27	
K01539	sodium/potassium-transporting ATPase subunit alpha [EC:7.2.2.13]	-0.24	3.6E-96	6.9E-96	-0.30	
K03339	6-phospho-5-dehydro-2-deoxy-D-gluconate aldolase [EC:4.1.2.29]	-0.24	4.2E-96	8.1E-96	-0.29	
K05358	quinate dehydrogenase (quinone) [EC:1.1.5.8]	-0.24	5.6E-96	1.1E-95	-0.31	
K05351	D-xylulose reductase [EC:1.1.1.9]	-0.24	6.4E-96	1.2E-95	-0.25	
K03335	inosose dehydratase [EC:4.2.1.44]	-0.24	8.2E-96	1.6E-95	-0.32	
K00846	ketohexokinase [EC:2.7.1.3]	-0.24	1.1E-95	2.1E-95	-0.31	
K13941	2-amino-4-hydroxy-6-hydroxymethyldihydropteridine diphosphokinase / dihydropteroate synthase [EC:2.7.6.3 2.5.1.15]	-0.24	3.4E-95	6.5E-95	-0.25	
K00832	aromatic-amino-acid transaminase [EC:2.6.1.57]	0.24	4.7E-95	9.0E-95	0.13	
K01452	chitin deacetylase [EC:3.5.1.41]	-0.24	5.0E-95	9.6E-95	-0.27	
K03101	signal peptidase II [EC:3.4.23.36]	-0.24	6.3E-95	1.2E-94	-0.36	
K16303	p-cumate 2,3-dioxygenase subunit beta [EC:1.14.12.25]	-0.24	1.6E-94	3.1E-94	-0.28	
K01643	citrate lyase subunit alpha / citrate CoA-transferase [EC:2.8.3.10]	0.24	2.0E-94	3.8E-94	0.14	
K00192	anaerobic carbon-monoxide dehydrogenase, CODH/ACS complex subunit alpha [EC:1.2.7.4]	-0.24	2.5E-94	4.8E-94	-0.30	
K00425	cytochrome bd ubiquinol oxidase subunit I [EC:7.1.1.7]	0.24	2.5E-94	4.8E-94	0.30	yes
K04128	hydroxymethyl cephem carbamoyltransferase [EC:2.1.3.7]	-0.24	3.4E-94	6.5E-94	-0.28	
K12255	guanidinobutyrase [EC:3.5.3.7]	-0.24	4.4E-94	8.4E-94	-0.31	yes
K03390	heterodisulfide reductase subunit C2 [EC:1.8.7.3 1.8.98.4 1.8.98.5 1.8.98.6]	-0.24	4.4E-94	8.4E-94	-0.21	
K03389	heterodisulfide reductase subunit B2 [EC:1.8.7.3 1.8.98.4 1.8.98.5 1.8.98.6]	-0.24	5.2E-94	9.9E-94	-0.21	
K05928	tocopherol O-methyltransferase [EC:2.1.1.95]	-0.24	5.6E-94	1.1E-93	-0.27	
K00969	nicotinate-nucleotide adenylyltransferase [EC:2.7.7.18]	-0.24	1.1E-93	2.1E-93	-0.32	
K15763	toluene monooxygenase system protein D [EC:1.14.13.236 1.14.13.-]	-0.24	1.1E-93	2.1E-93	-0.29	yes
K00287	dihydrofolate reductase [EC:1.5.1.3]	-0.24	1.6E-93	3.0E-93	-0.28	
K00288	methylenetetrahydrofolate dehydrogenase (NADP+) / methenyltetrahydrofolate cyclohydrolase / formyltetrahydrofolate synthetase [EC:1.5.1.5 3.5.4.9 6.3.4.3]	-0.24	1.6E-93	3.0E-93	-0.27	
K01053	gluconolactonase [EC:3.1.1.17]	-0.24	1.9E-93	3.6E-93	-0.31	yes
K01801	maleylpyruvate isomerase [EC:5.2.1.4]	-0.24	4.1E-93	7.8E-93	-0.30	
K07313	serine/threonine protein phosphatase 1 [EC:3.1.3.16]	0.24	6.1E-93	1.2E-92	0.09	
K01710	dTDP-glucose 4,6-dehydratase [EC:4.2.1.46]	-0.24	8.6E-93	1.6E-92	-0.17	
K14659	chitooligosaccharide deacetylase [EC:3.5.1.-]	-0.24	9.1E-93	1.7E-92	-0.29	

Predictor	Description	Pearson's r	P	FDR-adjusted P	Spearman's rho	Associated with fractures (P<0.05)
K14666	N-acetylglucosaminyltransferase [EC:2.4.1.-]	-0.24	9.1E-93	1.7E-92	-0.29	
K03404	magnesium chelatase subunit D [EC:6.6.1.1]	-0.24	1.2E-92	2.3E-92	-0.29	
K01207	beta-N-acetylhexosaminidase [EC:3.2.1.52]	-0.24	1.2E-92	2.3E-92	-0.29	
K00362	nitrite reductase (NADH) large subunit [EC:1.7.1.15]	0.24	1.3E-92	2.5E-92	0.06	
K13028	aldoxime dehydratase [EC:4.99.1.5]	-0.24	1.5E-92	2.8E-92	-0.29	
K00099	1-deoxy-D-xylulose-5-phosphate reductoisomerase [EC:1.1.1.267]	-0.24	1.5E-92	2.8E-92	-0.25	
K01915	glutamine synthetase [EC:6.3.1.2]	-0.24	1.9E-92	3.6E-92	-0.08	yes
K13875	L-arabonate dehydrase [EC:4.2.1.25]	-0.24	2.2E-92	4.2E-92	-0.31	
K01073	acyl-CoA hydrolase [EC:3.1.2.20]	-0.24	2.3E-92	4.3E-92	-0.30	
K02291	15-cis-phytoene synthase [EC:2.5.1.32]	-0.24	2.4E-92	4.5E-92	-0.25	
K10673	streptomycin 3\-kinase [EC:2.7.1.87]	-0.24	3.9E-92	7.3E-92	-0.29	
K05816	sn-glycerol 3-phosphate transport system ATP-binding protein [EC:7.6.2.10]	0.24	7.4E-92	1.4E-91	0.10	
K02528	16S rRNA (adenine1518-N6/adenine1519-N6)-dimethyltransferase [EC:2.1.1.182]	-0.24	1.1E-91	2.1E-91	-0.24	
K01101	4-nitrophenyl phosphatase [EC:3.1.3.41]	-0.24	1.1E-91	2.1E-91	-0.28	
K06607	myo-inositol catabolism protein IolS [EC:1.1.1.-]	-0.24	1.1E-91	2.1E-91	-0.27	yes
K05947	mannosyl-3-phosphoglycerate synthase [EC:2.4.1.217]	-0.24	1.4E-91	2.6E-91	-0.28	
K00641	homoserine O-acetyltransferase/O-succinyltransferase [EC:2.3.1.31 2.3.1.46]	-0.24	2.3E-91	4.3E-91	-0.22	
K10619	p-cumate 2,3-dioxygenase subunit alpha [EC:1.14.12.25]	-0.24	4.0E-91	7.5E-91	-0.27	
K07768	two-component system, OmpR family, sensor histidine kinase SenX3 [EC:2.7.13.3]	-0.24	4.2E-91	7.9E-91	-0.25	
K07318	adenine-specific DNA-methyltransferase [EC:2.1.1.72]	-0.24	1.3E-90	2.4E-90	-0.24	
K00074	3-hydroxybutyryl-CoA dehydrogenase [EC:1.1.1.157]	-0.24	1.5E-90	2.8E-90	-0.26	
K10212	glycosyl-4,4'-diaponeurosporenoate acyltransferase [EC:2.3.1.-]	-0.24	3.1E-90	5.8E-90	-0.28	
K00293	saccharopine dehydrogenase (NADP+, L-glutamate forming) [EC:1.5.1.10]	-0.24	3.3E-90	6.2E-90	-0.25	
K07812	trimethylamine-N-oxide reductase (cytochrome c) [EC:1.7.2.3]	0.24	3.4E-90	6.4E-90	0.10	
K00039	ribitol 2-dehydrogenase [EC:1.1.1.56]	-0.24	3.6E-90	6.7E-90	-0.30	
K01646	citrate lyase subunit gamma (acyl carrier protein)	0.24	4.2E-90	7.8E-90	0.13	
K03337	5-deoxy-glucuronate isomerase [EC:5.3.1.30]	-0.24	7.0E-90	1.3E-89	-0.31	
K15765	toluene monooxygenase electron transfer component [EC:1.18.1.3]	-0.24	1.3E-89	2.4E-89	-0.29	
K13678	1,2-diacylglycerol-3-alpha-glucose alpha-1,2-galactosyltransferase [EC:2.4.1.-]	-0.24	1.4E-89	2.6E-89	-0.27	
K06726	D-ribose pyranase [EC:5.4.99.62]	0.24	1.4E-89	2.6E-89	0.08	
K15242	2,6-dichloro-p-hydroquinone 1,2-dioxygenase [EC:1.13.11.-]	-0.24	2.2E-89	4.1E-89	-0.25	
K03399	cobalt-precorrin-7 (C5)-methyltransferase [EC:2.1.1.289]	-0.24	2.5E-89	4.6E-89	-0.30	
K05580	NAD(P)H-quinone oxidoreductase subunit I [EC:7.1.1.2]	-0.24	4.0E-89	7.4E-89	-0.27	yes
K02473	UDP-N-acetylglucosamine/UDP-N-acetylgalactosamine 4-epimerase [EC:5.1.3.7 5.1.3.-]	-0.24	4.1E-89	7.6E-89	-0.21	yes
K11263	acetyl-CoA/propionyl-CoA carboxylase, biotin carboxylase, biotin carboxyl carrier protein [EC:6.4.1.2 6.4.1.3 6.3.4.14]	-0.24	4.3E-89	8.0E-89	-0.25	
K00656	formate C-acetyltransferase [EC:2.3.1.54]	0.23	1.0E-88	1.9E-88	0.31	
K10536	agmatine deiminase [EC:3.5.3.12]	-0.23	1.3E-88	2.4E-88	-0.25	
K01653	acetolactate synthase I/III small subunit [EC:2.2.1.6]	-0.23	2.0E-88	3.7E-88	-0.27	
K04092	chorismate mutase [EC:5.4.99.5]	-0.23	2.6E-88	4.8E-88	-0.24	
K14519	NADP-dependent aldehyde dehydrogenase [EC:1.2.1.4]	-0.23	6.2E-88	1.1E-87	-0.30	yes
K02793	mannose PTS system EIIA component [EC:2.7.1.191]	-0.23	9.2E-88	1.7E-87	-0.29	
K15915	undecaprenyl phosphate N,N'-diacetylbacillosamine 1-phosphate transferase [EC:2.7.8.36]	-0.23	1.4E-87	2.6E-87	-0.27	

Predictor	Description	Pearson's r	P	FDR-adjusted P	Spearman's rho	Associated with fractures (P<0.05)
K02045	sulfate/thiosulfate transport system ATP-binding protein [EC:7.3.2.3]	0.23	1.4E-87	2.6E-87	0.07	
K00131	glyceraldehyde-3-phosphate dehydrogenase (NADP+) [EC:1.2.1.9]	-0.23	1.8E-87	3.3E-87	-0.26	
K14205	phosphatidylglycerol lysyltransferase [EC:2.3.2.3]	-0.23	3.3E-87	6.1E-87	-0.26	
K13074	biflaviolin synthase [EC:1.14.19.69]	-0.23	3.4E-87	6.3E-87	-0.27	
K04102	4,5-dihydroxyphthalate decarboxylase [EC:4.1.1.55]	-0.23	3.9E-87	7.2E-87	-0.29	
K01958	pyruvate carboxylase [EC:6.4.1.1]	-0.23	3.9E-87	7.2E-87	-0.27	
K11441	dehydrogluconokinase [EC:2.7.1.13]	-0.23	6.2E-87	1.1E-86	-0.28	
K00467	lactate 2-monooxygenase [EC:1.13.12.4]	-0.23	7.2E-87	1.3E-86	-0.26	
K11533	fatty acid synthase, bacteria type [EC:2.3.1.-]	-0.23	1.3E-86	2.4E-86	-0.24	
K01728	pectate lyase [EC:4.2.2.2]	-0.23	2.1E-86	3.9E-86	-0.31	yes
K13609	delta1-piperideine-2-carboxylate reductase [EC:1.5.1.21]	-0.23	3.3E-86	6.1E-86	-0.29	
K00873	pyruvate kinase [EC:2.7.1.40]	-0.23	3.6E-86	6.6E-86	-0.27	
K03418	N,N-dimethylformamidase large subunit [EC:3.5.1.56]	-0.23	3.7E-86	6.8E-86	-0.29	yes
K11529	glycerate 2-kinase [EC:2.7.1.165]	-0.23	4.1E-86	7.5E-86	-0.31	
K00480	salicylate hydroxylase [EC:1.14.13.1]	-0.23	5.0E-86	9.2E-86	-0.30	
K03336	3D-(3,5/4)-trihydroxycyclohexane-1,2-dione acylhydrolase (decyclizing) [EC:3.7.1.22]	-0.23	6.4E-86	1.2E-85	-0.30	
K08963	methylthioribose-1-phosphate isomerase [EC:5.3.1.23]	-0.23	6.8E-86	1.2E-85	-0.27	
K00476	aspartate beta-hydroxylase [EC:1.14.11.16]	-0.23	9.5E-86	1.7E-85	-0.24	
K05998	pseudomonalisin [EC:3.4.21.100]	-0.23	9.5E-86	1.7E-85	-0.24	
K00128	aldehyde dehydrogenase (NAD+) [EC:1.2.1.3]	-0.23	1.5E-85	2.7E-85	-0.22	
K00261	glutamate dehydrogenase (NAD(P)+) [EC:1.4.1.3]	-0.23	2.2E-85	4.0E-85	-0.30	
K01697	cystathionine beta-synthase [EC:4.2.1.22]	-0.23	2.9E-85	5.3E-85	-0.24	
K04035	magnesium-protoporphyrin IX monomethyl ester (oxidative) cyclase [EC:1.14.13.81]	-0.23	2.9E-85	5.3E-85	-0.28	
K11608	mycobacterial beta-ketoacyl-[acyl-carrier-protein] synthase III [EC:2.3.1.301]	-0.23	3.4E-85	6.2E-85	-0.27	
K01852	lanosterol synthase [EC:5.4.99.7]	-0.23	4.1E-85	7.5E-85	-0.28	
K02191	cobalt-precorrin-6B (C15)-methyltransferase [EC:2.1.1.196]	-0.23	4.4E-85	8.0E-85	-0.30	
K02781	glucitol/sorbitol PTS system EIIA component [EC:2.7.1.198]	0.23	5.5E-85	1.0E-84	0.10	
K02799	NA	-0.23	1.2E-84	2.2E-84	-0.27	
K02777	sugar PTS system EIIA component [EC:2.7.1.-]	0.23	1.4E-84	2.5E-84	0.09	
K00065	2-dehydro-3-deoxy-D-gluconate 5-dehydrogenase [EC:1.1.1.127]	0.23	2.0E-84	3.6E-84	0.06	
K10215	monooxygenase [EC:1.14.13.-]	-0.23	2.6E-84	4.7E-84	-0.27	yes
K05886	serine 3-dehydrogenase (NADP+) [EC:1.1.1.276]	-0.23	5.2E-84	9.4E-84	-0.28	yes
K06941	23S rRNA (adenine2503-C2)-methyltransferase [EC:2.1.1.192]	-0.23	5.5E-84	1.0E-83	-0.30	
K00367	ferredoxin-nitrate reductase [EC:1.7.7.2]	-0.23	8.7E-84	1.6E-83	-0.26	
K00943	dTMP kinase [EC:2.7.4.9]	-0.23	1.2E-83	2.2E-83	-0.27	
K02798	mannitol PTS system EIIA component [EC:2.7.1.197]	0.23	1.4E-83	2.5E-83	0.09	
K16165	fumarylpyruvate hydrolase [EC:3.7.1.20]	-0.23	1.4E-83	2.5E-83	-0.30	yes
K00471	gamma-butyrobetaine dioxygenase [EC:1.14.11.1]	-0.23	3.1E-83	5.6E-83	-0.28	
K00737	beta-1,4-mannosyl-glycoprotein beta-1,4-N-acetylglucosaminyltransferase [EC:2.4.1.144]	-0.23	5.4E-83	9.7E-83	-0.26	
K00824	D-alanine transaminase [EC:2.6.1.21]	-0.23	1.2E-82	2.2E-82	-0.29	
K13245	c-di-GMP-specific phosphodiesterase [EC:3.1.4.52]	-0.23	1.3E-82	2.3E-82	-0.24	
K06720	L-ectoine synthase [EC:4.2.1.108]	-0.23	3.8E-82	6.8E-82	-0.29	yes

Predictor	Description	Pearson's r	P	FDR-adjusted P	Spearman's rho	Associated with fractures (P<0.05)
K04094	methylenetetrahydrofolate--tRNA-(uracil-5-)-methyltransferase [EC:2.1.1.74]	-0.23	4.5E-82	8.1E-82	-0.26	
K07518	hydroxybutyrate-dimer hydrolase [EC:3.1.1.22]	-0.23	4.9E-82	8.8E-82	-0.29	yes
K01219	beta-agarase [EC:3.2.1.81]	-0.23	7.8E-82	1.4E-81	-0.25	yes
K01222	6-phospho-beta-glucosidase [EC:3.2.1.86]	0.22	2.6E-81	4.7E-81	0.06	
K01091	phosphoglycolate phosphatase [EC:3.1.3.18]	-0.22	3.1E-81	5.6E-81	-0.19	
K07250	4-aminobutyrate aminotransferase / (S)-3-amino-2-methylpropionate transaminase / 5-aminovalerate transaminase [EC:2.6.1.19 2.6.1.22 2.6.1.48]	0.22	7.5E-81	1.3E-80	0.08	
K00975	glucose-1-phosphate adenylyltransferase [EC:2.7.7.27]	-0.22	8.9E-81	1.6E-80	-0.27	
K13604	bacteriochlorophyllide d C-20 methyltransferase [EC:2.1.1.333]	-0.22	9.3E-81	1.7E-80	-0.27	
K00609	aspartate carbamoyltransferase catalytic subunit [EC:2.1.3.2]	-0.22	1.2E-80	2.1E-80	-0.26	
K07317	adenine-specific DNA-methyltransferase [EC:2.1.1.72]	-0.22	1.3E-80	2.3E-80	-0.25	
K03928	carboxylesterase [EC:3.1.1.1]	-0.22	1.6E-80	2.9E-80	-0.29	
K03338	5-dehydro-2-deoxygluconokinase [EC:2.7.1.92]	-0.22	3.4E-80	6.1E-80	-0.29	
K00294	1-pyrroline-5-carboxylate dehydrogenase [EC:1.2.1.88]	-0.22	6.2E-80	1.1E-79	-0.20	
K15514	3,4-dehydroadipyl-CoA semialdehyde dehydrogenase [EC:1.2.1.77]	-0.22	7.0E-80	1.3E-79	-0.29	yes
K16305	fructose-bisphosphate aldolase / 6-deoxy-5-ketofructose 1-phosphate synthase [EC:4.1.2.13 2.2.1.11]	-0.22	8.3E-80	1.5E-79	-0.28	
K15751	carbazole 1,9a-dioxygenase [EC:1.14.12.22]	-0.22	1.1E-79	2.0E-79	-0.25	
K13797	DNA-directed RNA polymerase subunit beta-beta' [EC:2.7.7.6]	-0.22	1.4E-79	2.5E-79	-0.25	
K15976	putative NAD(P)H nitroreductase [EC:1.-.-.-]	-0.22	1.4E-79	2.5E-79	-0.26	
K00554	tRNA (guanine37-N1)-methyltransferase [EC:2.1.1.228]	-0.22	2.6E-79	4.6E-79	-0.28	yes
K05343	maltose alpha-D-glucosyltransferase / alpha-amylase [EC:5.4.99.16 3.2.1.1]	-0.22	3.6E-79	6.4E-79	-0.20	yes
K13419	serine/threonine-protein kinase PknK [EC:2.7.11.1]	-0.22	4.0E-79	7.1E-79	-0.26	
K07778	two-component system, NarL family, sensor histidine kinase DesK [EC:2.7.13.3]	-0.22	7.3E-79	1.3E-78	-0.25	
K00255	long-chain-acyl-CoA dehydrogenase [EC:1.3.8.8]	-0.22	1.6E-78	2.8E-78	-0.25	
K01816	hydroxypyruvate isomerase [EC:5.3.1.22]	0.22	2.0E-78	3.6E-78	0.03	
K03060	DNA-directed RNA polymerase subunit omega [EC:2.7.7.6]	-0.22	2.3E-78	4.1E-78	-0.27	
K16039	N-glycosyltransferase [EC:2.4.1.-]	-0.22	2.4E-78	4.3E-78	-0.26	yes
K16157	methane monooxygenase component A alpha chain [EC:1.14.13.25]	-0.22	3.6E-78	6.4E-78	-0.26	
K16158	methane monooxygenase component A beta chain [EC:1.14.13.25]	-0.22	3.6E-78	6.4E-78	-0.26	
K15538	glycoprotein endo-alpha-1,2-mannosidase [EC:3.2.1.130]	-0.22	4.0E-78	7.1E-78	-0.23	
K13065	shikimate O-hydroxycinnamoyltransferase [EC:2.3.1.133]	-0.22	4.0E-78	7.1E-78	-0.23	
K01487	guanine deaminase [EC:3.5.4.3]	0.22	5.1E-78	9.0E-78	0.09	
K14415	tRNA-splicing ligase RtcB (3'-phosphate/5'-hydroxy nucleic acid ligase) [EC:6.5.1.8]	0.22	7.0E-78	1.2E-77	0.16	
K09516	all-trans-retinol 13,14-reductase [EC:1.3.99.23]	-0.22	8.4E-78	1.5E-77	-0.27	
K03601	exodeoxyribonuclease VII large subunit [EC:3.1.11.6]	-0.22	1.1E-77	1.9E-77	-0.23	
K00995	CDP-diacylglycerol---glycerol-3-phosphate 3-phosphatidyltransferase [EC:2.7.8.5]	-0.22	1.7E-77	3.0E-77	-0.26	
K09828	Delta24-sterol reductase [EC:1.3.1.72 1.3.1.-]	-0.22	2.4E-77	4.2E-77	-0.23	
K00878	hydroxyethylthiazole kinase [EC:2.7.1.50]	-0.22	2.7E-77	4.8E-77	-0.27	
K13713	fusion protein PurCD [EC:6.3.2.6 6.3.4.13]	-0.22	3.4E-77	6.0E-77	-0.24	yes
K16159	methane monooxygenase component A gamma chain [EC:1.14.13.25]	-0.22	4.1E-77	7.2E-77	-0.25	
K16161	methane monooxygenase component C [EC:1.14.13.25]	-0.22	4.1E-77	7.2E-77	-0.25	
K08687	N-carbamoylsarcosine amidase [EC:3.5.1.59]	-0.22	1.1E-76	1.9E-76	-0.24	yes
K01356	repressor LexA [EC:3.4.21.88]	-0.22	2.3E-76	4.0E-76	-0.26	

Predictor	Description	Pearson's r	P	FDR-adjusted P	Spearman's rho	Associated with fractures (P<0.05)
K06912	alpha-ketoglutarate-dependent 2,4-dichlorophenoxyacetate dioxygenase [EC:1.14.11.-]	-0.22	3.1E-76	5.4E-76	-0.25	
K03786	3-dehydroquinate dehydratase II [EC:4.2.1.10]	-0.22	3.4E-76	6.0E-76	-0.14	yes
K16215	2-ketoarginine methyltransferase [EC:2.1.1.243]	-0.22	3.8E-76	6.7E-76	-0.23	
K00054	hydroxymethylglutaryl-CoA reductase [EC:1.1.1.88]	-0.22	4.7E-76	8.2E-76	-0.24	
K14188	D-alanine--poly(phosphoribitol) ligase subunit 2 [EC:6.1.1.13]	-0.22	6.0E-76	1.1E-75	-0.23	
K09461	anthraniloyl-CoA monooxygenase [EC:1.14.13.40]	-0.22	8.3E-76	1.5E-75	-0.29	yes
K00622	arylamine N-acetyltransferase [EC:2.3.1.5]	-0.22	8.9E-76	1.6E-75	-0.24	
K13979	alcohol dehydrogenase (NADP+) [EC:1.1.1.2]	0.22	9.1E-76	1.6E-75	0.03	
K07309	Tat-targeted selenate reductase subunit YnfE [EC:1.97.1.9]	0.22	1.0E-75	1.7E-75	0.12	
K03769	peptidyl-prolyl cis-trans isomerase C [EC:5.2.1.8]	0.22	1.2E-75	2.1E-75	0.04	
K01493	dCMP deaminase [EC:3.5.4.12]	-0.22	1.6E-75	2.8E-75	-0.11	
K03692	glucosylglycerol-phosphate synthase [EC:2.4.1.213]	-0.22	1.8E-75	3.1E-75	-0.24	
K00010	myo-inositol 2-dehydrogenase / D-chiro-inositol 1-dehydrogenase [EC:1.1.1.18 1.1.1.369]	-0.22	1.8E-75	3.1E-75	-0.24	
K15912	UDP-N-acetylglucosamine 4,6-dehydratase [EC:4.2.1.135]	-0.22	5.7E-75	9.9E-75	-0.25	
K08261	D-sorbitol dehydrogenase (acceptor) [EC:1.1.99.21]	-0.22	6.4E-75	1.1E-74	-0.25	
K01750	ornithine cyclodeaminase [EC:4.3.1.12]	-0.22	7.6E-75	1.3E-74	-0.20	
K01142	exodeoxyribonuclease III [EC:3.1.11.2]	-0.22	8.7E-75	1.5E-74	-0.25	
K00105	alpha-glycerophosphate oxidase [EC:1.1.3.21]	-0.22	9.5E-75	1.7E-74	-0.24	
K13308	dTDP-4-amino-4,6-dideoxy-D-glucose transaminase [EC:2.6.1.33]	-0.22	1.1E-74	1.9E-74	-0.24	
K13786	cob(II)yrinic acid a,c-diamide reductase [EC:1.16.8.-]	-0.21	2.2E-74	3.8E-74	-0.23	
K02036	phosphate transport system ATP-binding protein [EC:7.3.2.1]	-0.21	3.7E-74	6.4E-74	-0.31	
K05304	sialic acid synthase [EC:2.5.1.56 2.5.1.57 2.5.1.132]	-0.21	3.9E-74	6.8E-74	-0.24	
K02535	UDP-3-O-[3-hydroxymyristoyl] N-acetylglucosamine deacetylase [EC:3.5.1.108]	0.21	4.3E-74	7.5E-74	0.10	
K01184	polygalacturonase [EC:3.2.1.15]	-0.21	4.8E-74	8.3E-74	-0.29	yes
K00561	23S rRNA (adenine-N6)-dimethyltransferase [EC:2.1.1.184]	-0.21	6.3E-74	1.1E-73	-0.20	
K00364	GMP reductase [EC:1.7.1.7]	0.21	7.0E-74	1.2E-73	0.10	
K00316	spermidine dehydrogenase [EC:1.5.99.6]	-0.21	7.8E-74	1.3E-73	-0.30	
K16559	endo-1,3-1,4-beta-glycanase ExoK [EC:3.2.1.-]	-0.21	8.7E-74	1.5E-73	-0.22	
K01479	formiminoglutamase [EC:3.5.3.8]	0.21	1.0E-73	1.7E-73	0.09	
K00918	ADP-dependent phosphofructokinase/glucokinase [EC:2.7.1.146 2.7.1.147]	-0.21	1.5E-73	2.6E-73	-0.27	
K00693	glycogen synthase [EC:2.4.1.11]	-0.21	1.5E-73	2.6E-73	-0.26	
K03822	putative long chain acyl-CoA synthase [EC:6.2.1.-]	-0.21	1.5E-73	2.6E-73	-0.23	
K01950	NAD+ synthase (glutamine-hydrolysing) [EC:6.3.5.1]	-0.21	1.6E-73	2.8E-73	-0.12	
K14728	phthiodiolone/phenolphthiodiolone dimycocerosates ketoreductase [EC:1.2.-.-]	-0.21	1.6E-73	2.8E-73	-0.24	
K00802	spermine synthase [EC:2.5.1.22]	-0.21	6.2E-73	1.1E-72	-0.25	
K08591	acyl phosphate:glycerol-3-phosphate acyltransferase [EC:2.3.1.275]	-0.21	1.1E-72	1.9E-72	-0.24	
K00938	phosphomevalonate kinase [EC:2.7.4.2]	-0.21	1.6E-72	2.8E-72	-0.23	
K06606	2-keto-myo-inositol isomerase [EC:5.3.99.11]	-0.21	1.7E-72	2.9E-72	-0.27	
K01002	phosphoglycerol transferase [EC:2.7.8.20]	0.21	1.8E-72	3.1E-72	0.06	
K15914	N,N'-diacetylbacillosaminyl-diphospho-undecaprenol alpha-1,3-N-acetylgalactosaminyltransferase [EC:2.4.1.290]	-0.21	2.1E-72	3.6E-72	-0.24	
K10804	acyl-CoA thioesterase I [EC:3.1.2.- 3.1.2.2 3.1.1.2 3.1.1.5]	0.21	2.5E-72	4.3E-72	0.05	
K14333	2,3-dihydroxybenzoate decarboxylase [EC:4.1.1.46]	-0.21	2.8E-72	4.8E-72	-0.25	

Predictor	Description	Pearson's r	P	FDR-adjusted P	Spearman's rho	Associated with fractures (P<0.05)
K07305	peptide-methionine (R)-S-oxide reductase [EC:1.8.4.12]	0.21	1.1E-71	1.9E-71	0.04	
K14469	acrylyl-CoA reductase (NADPH) / 3-hydroxypropionyl-CoA dehydratase / 3-hydroxypropionyl-CoA synthetase [EC:1.3.1.84 4.2.1.116 6.2.1.36]	-0.21	1.4E-71	2.4E-71	-0.22	
K02491	two-component system, sporulation sensor kinase A [EC:2.7.13.3]	-0.21	1.6E-71	2.7E-71	-0.27	
K03922	acyl-[acyl-carrier-protein] desaturase [EC:1.14.19.2]	-0.21	2.4E-71	4.1E-71	-0.24	
K07704	two-component system, LytTR family, sensor histidine kinase LytS [EC:2.7.13.3]	-0.21	4.5E-71	7.7E-71	-0.24	
K01399	pseudolysin [EC:3.4.24.26]	-0.21	5.7E-71	9.7E-71	-0.26	
K09994	(aminoalkyl)phosphonate N-acetyltransferase [EC:2.3.1.280]	0.21	7.6E-71	1.3E-70	0.06	
K01924	UDP-N-acetylmuramate--alanine ligase [EC:6.3.2.8]	-0.21	1.9E-70	3.2E-70	-0.26	yes
K02236	leader peptidase (prepilin peptidase) / N-methyltransferase [EC:3.4.23.43 2.1.1.-]	-0.21	2.1E-70	3.6E-70	-0.24	
K05574	NAD(P)H-quinone oxidoreductase subunit 3 [EC:7.1.1.2]	-0.21	2.3E-70	3.9E-70	-0.23	
K03574	8-oxo-dGTP diphosphatase [EC:3.6.1.55]	-0.21	2.3E-70	3.9E-70	-0.25	
K06013	STE24 endopeptidase [EC:3.4.24.84]	-0.21	3.5E-70	6.0E-70	-0.19	
K14170	chorismate mutase / prephenate dehydratase [EC:5.4.99.5 4.2.1.51]	-0.21	4.1E-70	7.0E-70	-0.26	
K01597	diphosphomevalonate decarboxylase [EC:4.1.1.33]	-0.21	6.3E-70	1.1E-69	-0.23	
K05301	sulfite dehydrogenase (cytochrome) subunit A [EC:1.8.2.1]	-0.21	9.8E-70	1.7E-69	-0.26	
K12700	non-specific riboncleoside hydrolase [EC:3.2.-.-]	0.21	1.4E-69	2.4E-69	0.09	
K03278	UDP-D-galactose:(glucosyl)LPS alpha-1,3-D-galactosyltransferase [EC:2.4.1.44]	-0.21	1.6E-69	2.7E-69	-0.23	
K11440	choline dehydrogenase [EC:1.1.1.1]	-0.21	1.9E-69	3.2E-69	-0.27	
K03794	sirohydrochlorin ferrochelatase [EC:4.99.1.4]	-0.21	2.1E-69	3.6E-69	-0.27	yes
K10535	hydroxylamine dehydrogenase [EC:1.7.2.6]	-0.21	3.3E-69	5.6E-69	-0.24	
K01819	galactose-6-phosphate isomerase [EC:5.3.1.26]	-0.21	3.4E-69	5.8E-69	-0.23	
K10798	poly [ADP-ribose] polymerase 2/3/4 [EC:2.4.2.30]	-0.21	4.7E-69	8.0E-69	-0.24	
K15899	pseudaminic acid cytidylyltransferase [EC:2.7.7.81]	-0.21	5.5E-69	9.3E-69	-0.22	
K16045	3beta-hydroxy-Delta5-steroid dehydrogenase / steroid Delta-isomerase [EC:1.1.1.145 5.3.3.1]	-0.21	6.0E-69	1.0E-68	-0.23	
K01740	O-acetylhomoserine (thiol)-lyase [EC:2.5.1.49]	-0.21	6.2E-69	1.0E-68	-0.05	
K00800	3-phosphoshikimate 1-carboxyvinyltransferase [EC:2.5.1.19]	-0.21	1.1E-68	1.9E-68	-0.24	
K03280	UDP-N-acetylglucosamine:(glucosyl)LPS alpha-1,2-N-acetylglucosaminyltransferase [EC:2.4.1.56]	0.21	1.1E-68	1.9E-68	0.14	yes
K13953	alcohol dehydrogenase, propanol-preferring [EC:1.1.1.1]	0.21	1.3E-68	2.2E-68	0.09	
K04479	DNA polymerase IV (archaeal DinB-like DNA polymerase) [EC:2.7.7.7]	-0.21	1.5E-68	2.5E-68	-0.24	
K00158	pyruvate oxidase [EC:1.2.3.3]	-0.21	2.3E-68	3.9E-68	-0.24	
K13990	glutamate formiminotransferase / formiminotetrahydrofolate cyclodeaminase [EC:2.1.2.5 4.3.1.4]	-0.21	2.8E-68	4.7E-68	-0.17	
K07649	two-component system, OmpR family, sensor histidine kinase TctE [EC:2.7.13.3]	-0.21	2.8E-68	4.7E-68	-0.28	
K06153	undecaprenyl-diphosphatase [EC:3.6.1.27]	-0.21	4.2E-68	7.1E-68	-0.23	
K01676	fumarate hydratase, class I [EC:4.2.1.2]	0.21	4.3E-68	7.2E-68	0.25	
K02786	lactose PTS system EIIA component [EC:2.7.1.207]	-0.21	4.5E-68	7.6E-68	-0.23	
K14368	3-alpha-mycarosylerythronolide B desosaminyl transferase [EC:2.4.1.278]	-0.21	4.9E-68	8.2E-68	-0.22	
K14366	6-deoxyerythronolide B hydroxylase [EC:1.14.15.35]	-0.21	4.9E-68	8.2E-68	-0.22	
K13311	dTDP-3-amino-3,4,6-trideoxy-alpha-D-glucopyranose N,N-dimethyltransferase [EC:2.1.1.234]	-0.21	4.9E-68	8.2E-68	-0.22	
K14370	erythromycin 12 hydroxylase [EC:1.14.13.154]	-0.21	4.9E-68	8.2E-68	-0.22	
K14369	erythromycin 3''-O-methyltransferase [EC:2.1.1.254]	-0.21	4.9E-68	8.2E-68	-0.22	
K12997	rhamnosyltransferase [EC:2.4.1.-]	-0.21	7.6E-68	1.3E-67	-0.22	
K14681	NA	-0.20	1.3E-67	2.2E-67	-0.26	

Predictor	Description	Pearson's r	P	FDR-adjusted P	Spearman's rho	Associated with fractures (P<0.05)
K03885	NADH:ubiquinone reductase (H+-translocating) [EC:7.1.1.2]	0.20	1.3E-67	2.2E-67	0.16	
K03621	phosphate acyltransferase [EC:2.3.1.274]	-0.20	2.0E-67	3.3E-67	-0.24	
K03464	muconolactone D-isomerase [EC:5.3.3.4]	-0.20	3.5E-67	5.9E-67	-0.28	
K00096	glycerol-1-phosphate dehydrogenase [NAD(P)+] [EC:1.1.1.261]	-0.20	8.0E-67	1.3E-66	-0.16	
K03079	L-ribulose-5-phosphate 3-epimerase [EC:5.1.3.22]	0.20	1.2E-66	2.0E-66	0.11	
K04564	superoxide dismutase, Fe-Mn family [EC:1.15.1.1]	0.20	1.7E-66	2.8E-66	0.23	
K03439	tRNA (guanine-N7-)-methyltransferase [EC:2.1.1.33]	-0.20	2.2E-66	3.7E-66	-0.30	
K10817	6-deoxyerythronolide-B synthase EryAI [EC:2.3.1.94]	-0.20	2.3E-66	3.8E-66	-0.22	
K03391	pentachlorophenol monooxygenase [EC:1.14.13.50]	-0.20	2.3E-66	3.8E-66	-0.22	
K08884	serine/threonine protein kinase, bacterial [EC:2.7.11.1]	-0.20	3.8E-66	6.3E-66	-0.26	
K01006	pyruvate, orthophosphate dikinase [EC:2.7.9.1]	-0.20	5.4E-66	9.0E-66	-0.13	
K06173	tRNA pseudouridine38-40 synthase [EC:5.4.99.12]	-0.20	8.3E-66	1.4E-65	-0.23	
K12993	O-antigen biosynthesis alpha-1,2-mannosyltransferase [EC:2.4.1.371 2.4.1.-]	-0.20	8.6E-66	1.4E-65	-0.22	
K01226	trehalose-6-phosphate hydrolase [EC:3.2.1.93]	0.20	1.1E-65	1.8E-65	0.09	
K13656	undecaprenyl-phosphate glucose phosphotransferase [EC:2.7.8.31]	-0.20	1.2E-65	2.0E-65	-0.22	
K02115	F-type H+-transporting ATPase subunit gamma	-0.20	1.2E-65	2.0E-65	-0.31	
K08969	L-glutamine---4-(methylsulfanyl)-2-oxobutanoate aminotransferase [EC:2.6.1.117]	-0.20	1.4E-65	2.3E-65	-0.21	
K10742	DNA replication ATP-dependent helicase Dna2 [EC:3.6.4.12]	-0.20	1.8E-65	3.0E-65	-0.22	
K01635	tagatose 1,6-diphosphate aldolase [EC:4.1.2.40]	-0.20	2.0E-65	3.3E-65	-0.24	
K00981	phosphatidate cytidylyltransferase [EC:2.7.7.41]	0.20	2.0E-65	3.3E-65	0.23	
K05396	D-cysteine desulfhydrase [EC:4.4.1.15]	0.20	2.5E-65	4.1E-65	0.05	
K10211	4,4'-diaponeurosporenoate glycosyltransferase [EC:2.4.1.-]	-0.20	3.9E-65	6.5E-65	-0.22	
K01354	oligopeptidase B [EC:3.4.21.83]	0.20	5.4E-65	8.9E-65	0.11	
K01104	protein-tyrosine phosphatase [EC:3.1.3.48]	-0.20	7.4E-65	1.2E-64	-0.22	
K01220	6-phospho-beta-galactosidase [EC:3.2.1.85]	-0.20	1.2E-64	2.0E-64	-0.23	
K00875	D-ribulokinase [EC:2.7.1.47]	-0.20	1.6E-64	2.6E-64	-0.22	
K04757	serine/threonine-protein kinase RsbW [EC:2.7.11.1]	-0.20	3.2E-64	5.3E-64	-0.19	
K11173	hydroxyacid-oxoacid transhydrogenase [EC:1.1.99.24]	-0.20	3.3E-64	5.4E-64	-0.23	
K00611	ornithine carbamoyltransferase [EC:2.1.3.3]	-0.20	3.8E-64	6.3E-64	-0.25	
K14661	nodulation protein F [EC:2.3.1.-]	-0.20	5.0E-64	8.2E-64	-0.21	
K14631	flavin reductase ActVB [EC:1.5.1.-]	-0.20	5.1E-64	8.4E-64	-0.23	
K05823	N-acetyldiaminopimelate deacetylase [EC:3.5.1.47]	-0.20	6.0E-64	9.9E-64	-0.21	
K12991	rhamnosyltransferase [EC:2.4.1.-]	-0.20	6.7E-64	1.1E-63	-0.20	
K02111	F-type H+/Na+-transporting ATPase subunit alpha [EC:7.1.2.2 7.2.2.1]	-0.20	7.0E-64	1.2E-63	-0.30	
K13529	AraC family transcriptional regulator, regulatory protein of adaptative response / DNA-3-methyladenine glycosylase II [EC:3.2.2.21]	-0.20	7.0E-64	1.2E-63	-0.26	
K00463	indoleamine 2,3-dioxygenase [EC:1.13.11.52]	-0.20	7.4E-64	1.2E-63	-0.23	
K15467	27-O-demethylrifamycin SV methyltransferase [EC:2.1.1.315]	-0.20	1.2E-63	2.0E-63	-0.21	
K01239	purine nucleosidase [EC:3.2.2.1]	-0.20	2.3E-63	3.8E-63	-0.22	
K06152	gluconate 2-dehydrogenase gamma chain [EC:1.1.99.3]	-0.20	2.5E-63	4.1E-63	-0.27	
K00823	4-aminobutyrate aminotransferase [EC:2.6.1.19]	0.20	6.2E-63	1.0E-62	0.05	
K13019	UDP-GlcNAc3NAcA epimerase [EC:5.1.3.23]	-0.20	6.4E-63	1.0E-62	-0.18	
K01232	maltose-6'-phosphate glucosidase [EC:3.2.1.122]	0.20	7.1E-63	1.2E-62	0.05	

Predictor	Description	Pearson's r	P	FDR-adjusted P	Spearman's rho	Associated with fractures (P<0.05)
K04042	bifunctional UDP-N-acetylglucosamine pyrophosphorylase / glucosamine-1-phosphate N-acetyltransferase [EC:2.7.7.23 2.3.1.157]	-0.20	7.4E-63	1.2E-62	-0.24	
K02611	ring-1,2-phenylacetyl-CoA epoxidase subunit PaaC [EC:1.14.13.149]	0.20	1.6E-62	2.6E-62	0.01	
K01281	X-Pro dipeptidyl-peptidase [EC:3.4.14.11]	-0.20	2.2E-62	3.6E-62	-0.21	
K12555	penicillin-binding protein 2A [EC:2.4.1.129 3.4.16.4]	-0.20	2.8E-62	4.6E-62	-0.21	
K12453	CDP-paratose synthetase [EC:1.1.1.342]	-0.20	9.4E-62	1.5E-61	-0.17	yes
K01428	urease subunit alpha [EC:3.5.1.5]	-0.20	1.4E-61	2.3E-61	-0.16	
K01071	medium-chain acyl-[acyl-carrier-protein] hydrolase [EC:3.1.2.21]	-0.20	1.5E-61	2.4E-61	-0.21	
K08070	2-alkenal reductase [EC:1.3.1.74]	-0.20	1.7E-61	2.8E-61	-0.21	
K00880	L-xylulokinase [EC:2.7.1.53]	0.20	1.7E-61	2.8E-61	0.09	
K00260	glutamate dehydrogenase [EC:1.4.1.2]	-0.20	2.0E-61	3.3E-61	-0.29	
K03385	nitrite reductase (cytochrome c-552) [EC:1.7.2.2]	0.20	2.5E-61	4.1E-61	0.15	
K01448	N-acetylmuramoyl-L-alanine amidase [EC:3.5.1.28]	0.19	2.6E-61	4.2E-61	0.28	
K08685	quinohemoprotein amine dehydrogenase [EC:1.4.9.1]	-0.19	4.1E-61	6.7E-61	-0.24	
K01430	urease subunit gamma [EC:3.5.1.5]	-0.19	4.4E-61	7.1E-61	-0.16	
K08961	chondroitin-sulfate-ABC endolyase/exolyase [EC:4.2.2.20 4.2.2.21]	-0.19	5.8E-61	9.4E-61	-0.17	yes
K01689	enolase [EC:4.2.1.11]	-0.19	6.3E-61	1.0E-60	-0.24	yes
K01674	carbonic anhydrase [EC:4.2.1.1]	-0.19	1.0E-60	1.6E-60	-0.21	
K08309	soluble lytic murein transglycosylase [EC:4.2.2.-]	0.19	1.2E-60	1.9E-60	0.06	
K15910	UDP-N-acetylbacillosamine transaminase [EC:2.6.1.34]	-0.19	1.2E-60	1.9E-60	-0.22	
K09698	nondiscriminating glutamyl-tRNA synthetase [EC:6.1.1.24]	-0.19	1.7E-60	2.8E-60	-0.21	
K05972	acetylxylan esterase [EC:3.1.1.72]	-0.19	2.0E-60	3.2E-60	-0.19	
K15972	tetracenomycin A2 monooxygenase-dioxygenase [EC:1.14.13.200]	-0.19	2.0E-60	3.2E-60	-0.19	
K02114	F-type H+-transporting ATPase subunit epsilon	-0.19	2.1E-60	3.4E-60	-0.30	
K06193	protein PhnA	0.19	2.2E-60	3.6E-60	0.07	
K05525	linalool 8-monooxygenase [EC:1.14.14.84]	-0.19	3.9E-60	6.3E-60	-0.23	
K13524	4-aminobutyrate aminotransferase / (S)-3-amino-2-methylpropionate transaminase [EC:2.6.1.19 2.6.1.22]	-0.19	7.5E-60	1.2E-59	-0.20	
K05573	NAD(P)H-quinone oxidoreductase subunit 2 [EC:7.1.1.2]	-0.19	8.5E-60	1.4E-59	-0.20	
K01075	4-hydroxybenzoyl-CoA thioesterase [EC:3.1.2.23]	-0.19	9.2E-60	1.5E-59	-0.27	yes
K03789	[ribosomal protein S18]-alanine N-acetyltransferase [EC:2.3.1.266]	-0.19	1.3E-59	2.1E-59	-0.24	
K03342	para-aminobenzoate synthetase / 4-amino-4-deoxychorismate lyase [EC:2.6.1.85 4.1.3.38]	-0.19	1.4E-59	2.3E-59	-0.21	
K01011	thiosulfate/3-mercaptopyruvate sulfurtransferase [EC:2.8.1.1 2.8.1.2]	0.19	2.1E-59	3.4E-59	0.04	
K00270	phenylalanine dehydrogenase [EC:1.4.1.20]	-0.19	3.7E-59	6.0E-59	-0.20	yes
K13018	UDP-2-acetamido-3-amino-2,3-dideoxy-glucuronate N-acetyltransferase [EC:2.3.1.201]	-0.19	6.0E-59	9.6E-59	-0.18	
K13747	carboxynorspermidine decarboxylase [EC:4.1.1.96]	-0.19	6.1E-59	9.8E-59	-0.11	
K15376	gephyrin [EC:2.10.1.1 2.7.7.75]	-0.19	6.2E-59	1.0E-58	-0.20	
K00306	sarcosine oxidase / L-pipecolate oxidase [EC:1.5.3.1 1.5.3.7]	-0.19	6.2E-59	1.0E-58	-0.20	
K01274	beta-Ala-Xaa dipeptidase [EC:3.4.13.-]	-0.19	7.2E-59	1.2E-58	-0.21	
K15016	enoyl-CoA hydratase / 3-hydroxyacyl-CoA dehydrogenase [EC:4.2.1.17 1.1.1.35]	-0.19	1.5E-58	2.4E-58	-0.20	
K00204	4Fe-4S ferredoxin	-0.19	1.5E-58	2.4E-58	-0.20	
K00640	serine O-acetyltransferase [EC:2.3.1.30]	0.19	1.7E-58	2.7E-58	0.26	
K10681	two-component system, OmpR family, sensor histidine kinase SaeS [EC:2.7.13.3]	-0.19	1.9E-58	3.0E-58	-0.21	
K13829	shikimate kinase / 3-dehydroquinate synthase [EC:2.7.1.71 4.2.3.4]	-0.19	2.3E-58	3.7E-58	-0.20	

Predictor	Description	Pearson's r	P	FDR-adjusted P	Spearman's rho	Associated with fractures (P<0.05)
K01575	acetolactate decarboxylase [EC:4.1.1.5]	-0.19	2.7E-58	4.3E-58	-0.20	
K02609	ring-1,2-phenylacetyl-CoA epoxidase subunit PaaA [EC:1.14.13.149]	0.19	2.9E-58	4.6E-58	0.00	
K01531	P-type Mg2+ transporter [EC:7.2.2.14]	0.19	3.9E-58	6.2E-58	0.14	
K01008	selenide, water dikinase [EC:2.7.9.3]	0.19	6.3E-58	1.0E-57	0.02	
K04561	nitric oxide reductase subunit B [EC:1.7.2.5]	-0.19	6.9E-58	1.1E-57	-0.26	
K00290	saccharopine dehydrogenase (NAD+, L-lysine forming) [EC:1.5.1.7]	-0.19	7.8E-58	1.2E-57	-0.11	
K00949	thiamine pyrophosphokinase [EC:2.7.6.2]	-0.19	1.1E-57	1.8E-57	-0.20	
K09568	FK506-binding protein 1 [EC:5.2.1.8]	-0.19	1.8E-57	2.9E-57	-0.20	
K06021	NA	-0.19	1.9E-57	3.0E-57	-0.20	
K05926	23S rRNA (adenosine1067-2'-O)-methyltransferase [EC:2.1.1.230]	-0.19	3.2E-57	5.1E-57	-0.20	
K00355	NAD(P)H dehydrogenase (quinone) [EC:1.6.5.2]	-0.19	4.1E-57	6.5E-57	-0.20	
K00681	gamma-glutamyltranspeptidase / glutathione hydrolase [EC:2.3.2.2 3.4.19.13]	0.19	4.3E-57	6.8E-57	0.03	
K04940	opine dehydrogenase [EC:1.5.1.28]	-0.19	5.8E-57	9.2E-57	-0.26	
K00836	diaminobutyrate-2-oxoglutarate transaminase [EC:2.6.1.76]	-0.19	7.3E-57	1.2E-56	-0.27	yes
K15760	toluene monooxygenase system protein A [EC:1.14.13.236 1.14.13.-]	-0.19	7.4E-57	1.2E-56	-0.22	
K01437	aspartoacylase [EC:3.5.1.15]	-0.19	9.4E-57	1.5E-56	-0.24	
K13532	two-component system, sporulation sensor kinase D [EC:2.7.13.3]	-0.19	1.0E-56	1.6E-56	-0.25	
K02446	fructose-1,6-bisphosphatase II [EC:3.1.3.11]	0.19	1.1E-56	1.7E-56	0.05	
K02486	two-component system, sensor kinase [EC:2.7.13.3]	-0.19	1.1E-56	1.7E-56	-0.19	
K02654	leader peptidase (prepilin peptidase) / N-methyltransferase [EC:3.4.23.43 2.1.1.-]	-0.19	1.6E-56	2.5E-56	-0.24	
K13714	bifunctional autolysin [EC:3.5.1.28 3.2.1.96]	-0.19	2.2E-56	3.5E-56	-0.20	
K08315	hydrogenase 3 maturation protease [EC:3.4.23.51]	0.19	2.2E-56	3.5E-56	0.09	
K12527	putative selenate reductase [EC:1.97.1.9]	0.19	2.9E-56	4.6E-56	0.04	
K01844	beta-lysine 5,6-aminomutase alpha subunit [EC:5.4.3.3]	-0.19	3.4E-56	5.4E-56	-0.17	
K01840	phosphomannomutase [EC:5.4.2.8]	0.19	3.4E-56	5.4E-56	0.27	
K16148	alpha-maltose-1-phosphate synthase [EC:2.4.1.342]	-0.19	3.8E-56	6.0E-56	-0.20	
K13016	UDP-N-acetyl-2-amino-2-deoxyglucuronate dehydrogenase [EC:1.1.1.335]	-0.19	6.0E-56	9.5E-56	-0.17	
K01935	dethiobiotin synthetase [EC:6.3.3.3]	0.19	7.0E-56	1.1E-55	0.30	
K08100	bilirubin oxidase [EC:1.3.3.5]	-0.19	1.5E-55	2.4E-55	-0.22	
K04486	histidinol-phosphatase (PHP family) [EC:3.1.3.15]	-0.19	1.5E-55	2.4E-55	-0.20	
K03206	azobenzene reductase [EC:1.7.1.6]	-0.19	1.6E-55	2.5E-55	-0.24	
K01731	pectate disaccharide-lyase [EC:4.2.2.9]	0.19	2.4E-55	3.8E-55	0.12	
K00831	phosphoserine aminotransferase [EC:2.6.1.52]	-0.19	2.4E-55	3.8E-55	-0.23	yes
K02113	F-type H+-transporting ATPase subunit delta	-0.19	2.6E-55	4.1E-55	-0.29	
K01304	pyroglutamyl-peptidase [EC:3.4.19.3]	-0.18	3.3E-55	5.2E-55	-0.20	
K00748	lipid-A-disaccharide synthase [EC:2.4.1.182]	0.18	3.5E-55	5.5E-55	0.29	
K01488	adenosine deaminase [EC:3.5.4.4]	0.18	4.4E-55	6.9E-55	0.14	
K07656	two-component system, OmpR family, sensor histidine kinase TrcS [EC:2.7.13.3]	-0.18	4.5E-55	7.1E-55	-0.20	
K01639	N-acetylneuraminate lyase [EC:4.1.3.3]	0.18	9.0E-55	1.4E-54	0.25	
K16178	dimethylamine---corrinoid protein Co-methyltransferase [EC:2.1.1.249]	-0.18	1.6E-54	2.5E-54	-0.19	
K01904	4-coumarate--CoA ligase [EC:6.2.1.12]	-0.18	1.7E-54	2.7E-54	-0.20	yes
K10774	tyrosine ammonia-lyase [EC:4.3.1.23]	-0.18	1.7E-54	2.7E-54	-0.20	yes

Predictor	Description	Pearson's r	P	FDR-adjusted P	Spearman's rho	Associated with fractures (P<0.05)
K06183	16S rRNA pseudouridine516 synthase [EC:5.4.99.19]	-0.18	2.3E-54	3.6E-54	-0.24	
K12253	5-guanidino-2-oxopentanoate decarboxylase [EC:4.1.1.75]	-0.18	2.4E-54	3.8E-54	-0.20	
K08356	arsenite oxidase large subunit [EC:1.20.2.1 1.20.9.1]	-0.18	3.5E-54	5.5E-54	-0.23	
K02000	glycine betaine/proline transport system ATP-binding protein [EC:7.6.2.9]	0.18	3.8E-54	5.9E-54	0.28	
K01322	prolyl oligopeptidase [EC:3.4.21.26]	-0.18	6.0E-54	9.4E-54	-0.13	
K03787	5'-nucleotidase [EC:3.1.3.5]	0.18	7.4E-54	1.2E-53	0.27	
K01644	citrate lyase subunit beta / citryl-CoA lyase [EC:4.1.3.34]	0.18	9.1E-54	1.4E-53	0.04	
K06177	tRNA pseudouridine32 synthase / 23S rRNA pseudouridine746 synthase [EC:5.4.99.28 5.4.99.29]	0.18	1.2E-53	1.9E-53	0.28	
K02108	F-type H+-transporting ATPase subunit a	-0.18	1.3E-53	2.0E-53	-0.26	
K15527	cysteate synthase [EC:2.5.1.76]	-0.18	1.3E-53	2.0E-53	-0.14	
K00858	NAD+ kinase [EC:2.7.1.23]	-0.18	1.4E-53	2.2E-53	-0.21	
K00525	ribonucleoside-diphosphate reductase alpha chain [EC:1.17.4.1]	0.18	2.0E-53	3.1E-53	0.27	
K11637	two-component system, CitB family, sensor histidine kinase CitS [EC:2.7.13.3]	-0.18	2.5E-53	3.9E-53	-0.24	
K01198	xylan 1,4-beta-xylosidase [EC:3.2.1.37]	-0.18	2.8E-53	4.4E-53	-0.22	
K03550	holliday junction DNA helicase RuvA [EC:3.6.4.12]	-0.18	3.7E-53	5.7E-53	-0.22	yes
K11395	2-dehydro-3-deoxy-phosphogluconate/2-dehydro-3-deoxy-6-phosphogalactonate aldolase [EC:4.1.2.55]	-0.18	4.8E-53	7.5E-53	-0.18	
K15059	2-aminophenol/2-amino-5-chlorophenol 1,6-dioxygenase subunit beta [EC:1.13.11.74 1.13.11.76]	-0.18	5.7E-53	8.8E-53	-0.20	
K15253	chlorocatechol 1,2-dioxygenase [EC:1.13.11.-]	-0.18	5.7E-53	8.8E-53	-0.20	
K01860	chloromuconate cycloisomerase [EC:5.5.1.7]	-0.18	5.7E-53	8.8E-53	-0.20	
K00297	methylenetetrahydrofolate reductase (NADH) [EC:1.5.1.54]	-0.18	5.9E-53	9.1E-53	0.01	
K10814	hydrogen cyanide synthase HcnA [EC:1.4.99.5]	-0.18	1.1E-52	1.7E-52	-0.22	
K00965	UDPglucose--hexose-1-phosphate uridylyltransferase [EC:2.7.7.12]	-0.18	1.6E-52	2.5E-52	-0.23	
K13292	phosphatidylglycerol---prolipoprotein diacylglyceryl transferase [EC:2.5.1.145]	-0.18	3.4E-52	5.3E-52	-0.23	
K12554	alanine adding enzyme [EC:2.3.2.-]	-0.18	3.6E-52	5.6E-52	-0.19	
K03814	monofunctional glycosyltransferase [EC:2.4.1.129]	0.18	5.5E-52	8.5E-52	0.28	
K03768	peptidyl-prolyl cis-trans isomerase B (cyclophilin B) [EC:5.2.1.8]	-0.18	6.2E-52	9.6E-52	-0.23	
K00897	kanamycin kinase [EC:2.7.1.95]	-0.18	8.1E-52	1.3E-51	-0.20	
K08728	nucleoside deoxyribosyltransferase [EC:2.4.2.6]	-0.18	1.0E-51	1.5E-51	-0.19	
K01117	sphingomyelin phosphodiesterase [EC:3.1.4.12]	-0.18	1.2E-51	1.9E-51	-0.18	
K00984	streptomycin 3\-adenylyltransferase [EC:2.7.7.47]	0.18	1.3E-51	2.0E-51	0.03	
K11065	thioredoxin-dependent peroxiredoxin [EC:1.11.1.24]	0.18	1.5E-51	2.3E-51	0.25	
K00088	IMP dehydrogenase [EC:1.1.1.205]	-0.18	1.8E-51	2.8E-51	-0.21	
K01769	guanylate cyclase, other [EC:4.6.1.2]	-0.18	1.9E-51	2.9E-51	-0.19	
K05841	sterol 3beta-glucosyltransferase [EC:2.4.1.173]	-0.18	3.0E-51	4.6E-51	-0.20	
K02316	DNA primase [EC:2.7.7.101]	-0.18	5.1E-51	7.8E-51	-0.22	yes
K12524	bifunctional aspartokinase / homoserine dehydrogenase 1 [EC:2.7.2.4 1.1.1.3]	0.18	7.0E-51	1.1E-50	0.28	
K13500	chondroitin synthase [EC:2.4.1.175 2.4.1.226]	-0.18	9.5E-51	1.5E-50	-0.21	yes
K01451	hippurate hydrolase [EC:3.5.1.32]	-0.18	1.3E-50	2.0E-50	-0.17	
K01401	aureolysin [EC:3.4.24.29]	-0.18	1.4E-50	2.1E-50	-0.18	
K04478	monofunctional glycosyltransferase [EC:2.4.1.129]	-0.18	1.4E-50	2.1E-50	-0.18	
K11695	peptidoglycan pentaglycine glycine transferase (the fourth and fifth glycine) [EC:2.3.2.18]	-0.18	1.4E-50	2.1E-50	-0.18	
K11694	peptidoglycan pentaglycine glycine transferase (the second and third glycine) [EC:2.3.2.17]	-0.18	1.4E-50	2.1E-50	-0.18	

Predictor	Description	Pearson's r	P	FDR-adjusted P	Spearman's rho	Associated with fractures (P<0.05)
K11442	putative uridylyltransferase [EC:2.7.7.-]	-0.18	1.4E-50	2.1E-50	-0.18	
K07681	two-component system, NarL family, vancomycin resistance sensor histidine kinase VraS [EC:2.7.13.3]	-0.18	1.4E-50	2.1E-50	-0.18	
K07306	anaerobic dimethyl sulfoxide reductase subunit A [EC:1.8.5.3]	0.18	1.4E-50	2.1E-50	0.07	
K00901	diacylglycerol kinase (ATP) [EC:2.7.1.107]	0.18	1.6E-50	2.4E-50	0.26	
K10915	CAI-1 autoinducer synthase [EC:2.3.-.-]	-0.18	2.3E-50	3.5E-50	-0.20	
K01580	glutamate decarboxylase [EC:4.1.1.15]	0.18	3.0E-50	4.6E-50	0.20	
K08640	zinc D-Ala-D-Ala carboxypeptidase [EC:3.4.17.14]	-0.18	5.8E-50	8.9E-50	-0.19	
K00185	dimethyl sulfoxide reductase membrane subunit	-0.18	6.5E-50	9.9E-50	-0.20	
K11617	two-component system, NarL family, sensor histidine kinase LiaS [EC:2.7.13.3]	-0.18	1.2E-49	1.8E-49	-0.19	
K00925	acetate kinase [EC:2.7.2.1]	-0.18	1.3E-49	2.0E-49	-0.21	yes
K01599	uroporphyrinogen decarboxylase [EC:4.1.1.37]	0.18	1.5E-49	2.3E-49	0.26	
K01251	adenosylhomocysteinase [EC:3.3.1.1]	-0.17	3.0E-49	4.6E-49	-0.17	
K04487	cysteine desulfurase [EC:2.8.1.7]	-0.17	3.7E-49	5.6E-49	-0.24	
K01031	3-oxoadipate CoA-transferase, alpha subunit [EC:2.8.3.6]	-0.17	4.8E-49	7.3E-49	-0.26	
K15064	syringate O-demethylase [EC:2.1.1.-]	-0.17	6.8E-49	1.0E-48	-0.18	
K04084	thioredoxin:protein disulfide reductase [EC:1.8.4.16]	0.17	1.1E-48	1.7E-48	0.28	
K05910	NADH peroxidase [EC:1.11.1.1]	-0.17	1.3E-48	2.0E-48	-0.20	
K03216	tRNA (cytidine/uridine-2'-O-)-methyltransferase [EC:2.1.1.207]	-0.17	1.5E-48	2.3E-48	-0.22	
K14581	naphthalene 1,2-dioxygenase ferredoxin reductase component [EC:1.18.1.7]	0.17	1.6E-48	2.4E-48	0.12	
K01607	4-carboxymuconolactone decarboxylase [EC:4.1.1.44]	-0.17	1.9E-48	2.9E-48	-0.20	
K02523	octaprenyl-diphosphate synthase [EC:2.5.1.90]	0.17	3.5E-48	5.3E-48	0.24	
K10916	two-component system, CAI-1 autoinducer sensor kinase/phosphatase CqsS [EC:2.7.13.3 3.1.3.-]	-0.17	3.9E-48	5.9E-48	-0.23	
K02493	release factor glutamine methyltransferase [EC:2.1.1.297]	-0.17	6.7E-48	1.0E-47	-0.21	yes
K00652	8-amino-7-oxononanoate synthase [EC:2.3.1.47]	0.17	6.7E-48	1.0E-47	0.26	
K08264	heterodisulfide reductase subunit D [EC:1.8.98.1]	-0.17	8.1E-48	1.2E-47	-0.18	
K13378	NADH-quinone oxidoreductase subunit C/D [EC:7.1.1.2]	0.17	8.7E-48	1.3E-47	0.28	
K15234	citryl-CoA lyase [EC:4.1.3.34]	-0.17	9.0E-48	1.4E-47	-0.17	
K08265	heterodisulfide reductase subunit E [EC:1.8.98.1]	-0.17	1.5E-47	2.3E-47	-0.18	
K12267	peptide methionine sulfoxide reductase msrA/msrB [EC:1.8.4.11 1.8.4.12]	-0.17	2.3E-47	3.5E-47	-0.17	
K00383	glutathione reductase (NADPH) [EC:1.8.1.7]	0.17	2.5E-47	3.8E-47	0.07	
K00960	DNA-directed RNA polymerase subunit 13 [EC:2.7.7.6]	-0.17	2.8E-47	4.2E-47	-0.19	
K01424	L-asparaginase [EC:3.5.1.1]	0.17	3.9E-47	5.9E-47	0.25	
K01341	kexin [EC:3.4.21.61]	-0.17	5.9E-47	8.9E-47	-0.18	
K04342	streptomycin-6-phosphatase [EC:3.1.3.39]	-0.17	1.1E-46	1.7E-46	-0.18	
K03775	FKBP-type peptidyl-prolyl cis-trans isomerase SlyD [EC:5.2.1.8]	0.17	1.1E-46	1.7E-46	0.27	
K11194	fructose PTS system EIIA component [EC:2.7.1.202]	-0.17	1.2E-46	1.8E-46	-0.17	
K11195	fructose PTS system EIIB component [EC:2.7.1.202]	-0.17	1.2E-46	1.8E-46	-0.17	
K10027	phytoene desaturase [EC:1.3.99.26 1.3.99.28 1.3.99.29 1.3.99.31]	-0.17	1.4E-46	2.1E-46	-0.23	
K01032	3-oxoadipate CoA-transferase, beta subunit [EC:2.8.3.6]	-0.17	1.6E-46	2.4E-46	-0.25	
K00997	holo-[acyl-carrier protein] synthase [EC:2.7.8.7]	-0.17	1.7E-46	2.6E-46	-0.22	
K01758	cystathionine gamma-lyase [EC:4.4.1.1]	-0.17	1.9E-46	2.9E-46	-0.26	
K01255	leucyl aminopeptidase [EC:3.4.11.1]	0.17	2.1E-46	3.2E-46	0.01	

Predictor	Description	Pearson's r	P	FDR-adjusted P	Spearman's rho	Associated with fractures (P<0.05)
K15515	sulfoacetaldehyde dehydrogenase [EC:1.2.1.81]	-0.17	2.6E-46	3.9E-46	-0.28	
K02203	phosphoserine / homoserine phosphotransferase [EC:3.1.3.3 2.7.1.39]	-0.17	5.8E-46	8.7E-46	-0.19	
K09887	dCTP deaminase (dUMP-forming) [EC:3.5.4.30]	-0.17	6.4E-46	9.6E-46	-0.17	
K03771	peptidyl-prolyl cis-trans isomerase SurA [EC:5.2.1.8]	0.17	7.1E-46	1.1E-45	0.27	
K00712	poly(glycerol-phosphate) alpha-glucosyltransferase [EC:2.4.1.52]	-0.17	7.4E-46	1.1E-45	-0.18	
K11754	dihydrofolate synthase / folylpolyglutamate synthase [EC:6.3.2.12 6.3.2.17]	-0.17	8.3E-46	1.2E-45	-0.20	yes
K13985	N-acyl-phosphatidylethanolamine-hydrolysing phospholipase D [EC:3.1.4.54]	-0.17	8.8E-46	1.3E-45	-0.17	
K12506	2-C-methyl-D-erythritol 4-phosphate cytidylyltransferase / 2-C-methyl-D-erythritol 2,4-cyclodiphosphate synthase [EC:2.7.7.60 4.6.1.12]	-0.17	1.0E-45	1.5E-45	-0.22	
K08253	non-specific protein-tyrosine kinase [EC:2.7.10.2]	-0.17	1.4E-45	2.1E-45	-0.19	
K13664	acyltransferase [EC:2.3.1.-]	-0.17	1.4E-45	2.1E-45	-0.18	
K13658	beta-1,4-mannosyltransferase [EC:2.4.1.251]	-0.17	1.4E-45	2.1E-45	-0.18	
K05986	nuclease S1 [EC:3.1.30.1]	-0.17	1.4E-45	2.1E-45	-0.18	
K08300	ribonuclease E [EC:3.1.26.12]	0.17	1.7E-45	2.5E-45	0.07	
K13439	cysteine protease avirulence protein AvrRpt2 [EC:3.4.22.-]	-0.17	2.2E-45	3.3E-45	-0.17	
K00982	[glutamine synthetase] adenylyltransferase / [glutamine synthetase]-adenylyl-L-tyrosine phosphorylase [EC:2.7.7.42 2.7.7.89]	0.17	2.3E-45	3.4E-45	0.07	
K10837	O-phosphoseryl-tRNA(Sec) kinase [EC:2.7.1.164]	-0.17	2.7E-45	4.0E-45	-0.17	
K01856	muconate cycloisomerase [EC:5.5.1.1]	-0.17	2.9E-45	4.3E-45	-0.25	
K15740	tetrahydromethanopterin:alpha-L-glutamate ligase [EC:6.3.2.33]	-0.17	3.7E-45	5.5E-45	-0.18	
K01482	dimethylargininase [EC:3.5.3.18]	-0.17	4.0E-45	5.9E-45	-0.19	
K00432	glutathione peroxidase [EC:1.11.1.9]	0.17	4.2E-45	6.2E-45	0.22	
K10222	2,6-dioxo-6-phenylhexa-3-enoate hydrolase [EC:3.7.1.8]	-0.17	4.7E-45	7.0E-45	-0.18	
K08689	biphenyl 2,3-dioxygenase subunit alpha [EC:1.14.12.18]	-0.17	4.7E-45	7.0E-45	-0.18	
K00462	biphenyl-2,3-diol 1,2-dioxygenase [EC:1.13.11.39]	-0.17	4.7E-45	7.0E-45	-0.18	
K02517	Kdo2-lipid IVA lauroyltransferase/acyltransferase [EC:2.3.1.241 2.3.1.-]	0.17	1.0E-44	1.5E-44	0.24	
K12466	(+)-trans-carveol dehydrogenase [EC:1.1.1.275]	-0.17	1.2E-44	1.8E-44	-0.18	
K01361	lactocepin [EC:3.4.21.96]	-0.17	1.4E-44	2.1E-44	-0.18	
K00674	2,3,4,5-tetrahydropyridine-2,6-dicarboxylate N-succinyltransferase [EC:2.3.1.117]	-0.17	1.6E-44	2.4E-44	-0.20	
K01115	phospholipase D1/2 [EC:3.1.4.4]	-0.17	1.8E-44	2.7E-44	-0.21	
K01624	fructose-bisphosphate aldolase, class II [EC:4.1.2.13]	-0.17	3.6E-44	5.3E-44	-0.20	
K00705	4-alpha-glucanotransferase [EC:2.4.1.25]	-0.16	4.0E-44	5.9E-44	-0.16	
K01218	mannan endo-1,4-beta-mannosidase [EC:3.2.1.78]	-0.16	4.0E-44	5.9E-44	-0.16	
K00563	23S rRNA (guanine745-N1)-methyltransferase [EC:2.1.1.187]	0.16	5.0E-44	7.4E-44	0.04	
K07683	two-component system, NarL family, sensor histidine kinase NreB [EC:2.7.13.3]	-0.16	7.3E-44	1.1E-43	-0.23	
K03827	putative acetyltransferase [EC:2.3.1.-]	0.16	1.4E-43	2.1E-43	0.26	
K00598	trans-aconitate 2-methyltransferase [EC:2.1.1.144]	0.16	1.7E-43	2.5E-43	0.09	
K01921	D-alanine-D-alanine ligase [EC:6.3.2.4]	-0.16	1.8E-43	2.7E-43	-0.22	
K10530	L-lactate oxidase [EC:1.1.3.2]	-0.16	4.4E-43	6.5E-43	-0.20	
K12988	alpha-1,3-rhamnosyltransferase [EC:2.4.1.-]	-0.16	4.4E-43	6.5E-43	-0.18	
K04118	pimeloyl-CoA dehydrogenase [EC:1.3.1.62]	-0.16	4.7E-43	6.9E-43	-0.17	
K01446	peptidoglycan recognition protein	-0.16	6.1E-43	9.0E-43	-0.17	
K12570	streptomycin 6-kinase [EC:2.7.1.72]	-0.16	6.1E-43	9.0E-43	-0.17	
K01312	trypsin [EC:3.4.21.4]	-0.16	7.0E-43	1.0E-42	-0.17	

Predictor	Description	Pearson's r	P	FDR-adjusted P	Spearman's rho	Associated with fractures (P<0.05)
K08641	zinc D-Ala-D-Ala dipeptidase [EC:3.4.13.22]	0.16	1.8E-42	2.6E-42	0.27	
K14596	zeaxanthin glucosyltransferase [EC:2.4.1.276]	-0.16	2.2E-42	3.2E-42	-0.24	
K10830	manganese/zinc transport system ATP-binding protein [EC:7.2.2.5]	-0.16	2.3E-42	3.4E-42	-0.18	
K00867	type I pantothenate kinase [EC:2.7.1.33]	0.16	2.6E-42	3.8E-42	0.07	
K16363	UDP-3-O-[3-hydroxymyristoyl] N-acetylglucosamine deacetylase / 3-hydroxyacyl-[acyl-carrier-protein] dehydratase [EC:3.5.1.108 4.2.1.59]	-0.16	2.8E-42	4.1E-42	-0.14	
K05535	alpha 1,2-mannosyltransferase [EC:2.4.1.-]	-0.16	3.3E-42	4.8E-42	-0.16	
K05308	gluconate/galactonate dehydratase [EC:4.2.1.140]	-0.16	4.5E-42	6.6E-42	-0.16	
K02858	3,4-dihydroxy 2-butanone 4-phosphate synthase [EC:4.1.99.12]	0.16	4.8E-42	7.0E-42	0.04	
K12507	acyl-CoA synthetase [EC:6.2.1.-]	0.16	6.6E-42	9.7E-42	0.09	
K01439	succinyl-diaminopimelate desuccinylase [EC:3.5.1.18]	-0.16	7.1E-42	1.0E-41	-0.21	
K01213	galacturan 1,4-alpha-galacturonidase [EC:3.2.1.67]	-0.16	9.2E-42	1.3E-41	-0.16	yes
K01873	valyl-tRNA synthetase [EC:6.1.1.9]	-0.16	1.4E-41	2.0E-41	-0.17	
K10834	heme transport system ATP-binding protein [EC:7.6.2.5]	-0.16	1.6E-41	2.3E-41	-0.17	
K00954	pantetheine-phosphate adenylyltransferase [EC:2.7.7.3]	-0.16	1.7E-41	2.5E-41	-0.18	yes
K15038	succinyl-CoA reductase [EC:1.2.1.76]	-0.16	1.8E-41	2.6E-41	-0.16	
K13501	anthranilate synthase / indole-3-glycerol phosphate synthase / phosphoribosylanthranilate isomerase [EC:4.1.3.27 4.1.1.48 5.3.1.24]	-0.16	1.9E-41	2.8E-41	-0.16	
K00086	1,3-propanediol dehydrogenase [EC:1.1.1.202]	-0.16	4.9E-41	7.1E-41	-0.25	
K01906	6-carboxyhexanoate--CoA ligase [EC:6.2.1.14]	-0.16	6.6E-41	9.6E-41	-0.19	
K11214	sedoheptulokinase [EC:2.7.1.14]	-0.16	8.3E-41	1.2E-40	-0.19	
K08281	nicotinamidase/pyrazinamidase [EC:3.5.1.19 3.5.1.-]	0.16	9.2E-41	1.3E-40	0.06	
K13059	N-acetylhexosamine 1-kinase [EC:2.7.1.162]	-0.16	1.0E-40	1.5E-40	-0.17	
K00848	rhamnulokinase [EC:2.7.1.5]	0.16	1.1E-40	1.6E-40	0.23	
K00956	sulfate adenylyltransferase subunit 1 [EC:2.7.7.4]	0.16	1.4E-40	2.0E-40	0.26	
K03341	O-phospho-L-seryl-tRNASec:L-selenocysteinyl-tRNA synthase [EC:2.9.1.2]	-0.16	1.6E-40	2.3E-40	-0.16	
K03273	D-glycero-D-manno-heptose 1,7-bisphosphate phosphatase [EC:3.1.3.82 3.1.3.83]	-0.16	1.7E-40	2.5E-40	-0.21	
K01241	AMP nucleosidase [EC:3.2.2.4]	0.16	1.9E-40	2.8E-40	0.26	
K15777	4,5-DOPA dioxygenase extradiol [EC:1.13.11.-]	0.16	2.3E-40	3.3E-40	0.04	
K00860	adenylylsulfate kinase [EC:2.7.1.25]	0.16	2.5E-40	3.6E-40	0.26	
K07636	two-component system, OmpR family, phosphate regulon sensor histidine kinase PhoR [EC:2.7.13.3]	-0.16	2.6E-40	3.8E-40	-0.18	
K15460	tRNA1Val (adenine37-N6)-methyltransferase [EC:2.1.1.223]	0.16	2.7E-40	3.9E-40	0.24	
K01191	alpha-mannosidase [EC:3.2.1.24]	-0.16	2.8E-40	4.1E-40	-0.15	
K01786	NA	-0.16	3.4E-40	4.9E-40	-0.21	
K01729	poly(beta-D-mannuronate) lyase [EC:4.2.2.3]	-0.16	4.0E-40	5.8E-40	-0.23	
K03473	erythronate-4-phosphate dehydrogenase [EC:1.1.1.290]	0.16	4.3E-40	6.2E-40	0.24	
K02371	enoyl-[acyl-carrier protein] reductase II [EC:1.3.1.9]	-0.16	4.5E-40	6.5E-40	-0.15	
K00820	glutamine---fructose-6-phosphate transaminase (isomerizing) [EC:2.6.1.16]	-0.16	4.9E-40	7.1E-40	-0.18	yes
K00657	diamine N-acetyltransferase [EC:2.3.1.57]	0.16	5.4E-40	7.8E-40	0.24	
K01872	alanyl-tRNA synthetase [EC:6.1.1.7]	-0.16	7.1E-40	1.0E-39	-0.19	
K07698	two-component system, sporulation sensor kinase C [EC:2.7.13.3]	-0.16	7.9E-40	1.1E-39	-0.21	
K12552	penicillin-binding protein 1 [EC:3.4.-.-]	-0.16	8.2E-40	1.2E-39	-0.16	
K05575	NAD(P)H-quinone oxidoreductase subunit 4 [EC:7.1.1.2]	-0.16	1.4E-39	2.0E-39	-0.16	
K07255	taurine dehydrogenase small subunit [EC:1.4.2.-]	-0.16	1.6E-39	2.3E-39	-0.16	

Predictor	Description	Pearson's r	P	FDR-adjusted P	Spearman's rho	Associated with fractures (P<0.05)
K07106	N-acetylmuramic acid 6-phosphate etherase [EC:4.2.1.126]	0.16	1.7E-39	2.4E-39	0.24	
K02361	isochorismate synthase [EC:5.4.4.2]	0.16	1.8E-39	2.6E-39	0.26	
K01048	lysophospholipase [EC:3.1.1.5]	-0.16	3.4E-39	4.9E-39	-0.20	
K03271	D-sedoheptulose 7-phosphate isomerase [EC:5.3.1.28]	-0.15	4.2E-39	6.0E-39	-0.21	
K04708	3-dehydrosphinganine reductase [EC:1.1.1.102]	-0.15	6.1E-39	8.8E-39	-0.15	
K00230	menaquinone-dependent protoporphyrinogen oxidase [EC:1.3.5.3]	0.15	7.8E-39	1.1E-38	0.02	
K01534	Zn2+/Cd2+-exporting ATPase [EC:7.2.2.12 7.2.2.21]	0.15	9.9E-39	1.4E-38	0.20	
K02769	fructose PTS system EIIB component [EC:2.7.1.202]	0.15	1.1E-38	1.6E-38	0.03	
K03501	16S rRNA (guanine527-N7)-methyltransferase [EC:2.1.1.170]	0.15	3.9E-38	5.6E-38	0.21	
K01611	S-adenosylmethionine decarboxylase [EC:4.1.1.50]	0.15	5.7E-38	8.2E-38	0.05	
K15907	pentalenene oxygenase [EC:1.14.15.32]	-0.15	5.7E-38	8.2E-38	-0.15	
K00382	dihydrolipoamide dehydrogenase [EC:1.8.1.4]	-0.15	5.9E-38	8.4E-38	-0.18	
K00545	catechol O-methyltransferase [EC:2.1.1.6]	-0.15	7.4E-38	1.1E-37	-0.16	
K12292	ATP-binding cassette, subfamily C, bacterial, competence factor transporting protein [EC:3.4.22.-]	-0.15	7.9E-38	1.1E-37	-0.19	
K15756	2-hydroxy-6-oxo-6-(2'-aminophenyl)hexa-2,4-dienoate hydrolase [EC:3.7.1.13]	-0.15	9.2E-38	1.3E-37	-0.17	
K01609	indole-3-glycerol phosphate synthase [EC:4.1.1.48]	-0.15	1.2E-37	1.7E-37	-0.06	
K16651	L-threonine kinase [EC:2.7.1.177]	-0.15	1.4E-37	2.0E-37	-0.22	
K15929	glycosyltransferase [EC:2.4.1.-]	-0.15	1.5E-37	2.1E-37	-0.16	
K05550	benzoate/toluate 1,2-dioxygenase subunit beta [EC:1.14.12.10 1.14.12.-]	-0.15	1.5E-37	2.1E-37	-0.24	
K14585	trans-o-hydroxybenzylidenepyruvate hydratase-aldolase [EC:4.1.2.45]	-0.15	1.8E-37	2.6E-37	-0.17	
K00030	isocitrate dehydrogenase (NAD+) [EC:1.1.1.41]	-0.15	2.6E-37	3.7E-37	-0.20	
K00036	glucose-6-phosphate 1-dehydrogenase [EC:1.1.1.49 1.1.1.363]	0.15	2.7E-37	3.8E-37	0.25	
K01250	pyrimidine-specific ribonucleoside hydrolase [EC:3.2.-.-]	0.15	3.0E-37	4.3E-37	0.13	
K05593	aminoglycoside 6-adenylyltransferase [EC:2.7.7.-]	-0.15	3.1E-37	4.4E-37	-0.16	
K03830	putative acetyltransferase [EC:2.3.1.-]	0.15	3.3E-37	4.7E-37	0.01	
K01615	glutaconyl-CoA decarboxylase subunit alpha [EC:7.2.4.5]	-0.15	6.5E-37	9.2E-37	-0.21	
K08483	phosphoenolpyruvate-protein phosphotransferase (PTS system enzyme I) [EC:2.7.3.9]	-0.15	9.6E-37	1.4E-36	-0.19	
K03471	ribonuclease HIII [EC:3.1.26.4]	-0.15	9.7E-37	1.4E-36	-0.17	
K05549	benzoate/toluate 1,2-dioxygenase subunit alpha [EC:1.14.12.10 1.14.12.-]	-0.15	1.1E-36	1.6E-36	-0.24	yes
K14583	1,2-dihydroxynaphthalene dioxygenase [EC:1.13.11.56]	-0.15	1.2E-36	1.7E-36	-0.15	
K14584	2-hydroxychromene-2-carboxylate isomerase [EC:5.99.1.4]	-0.15	1.2E-36	1.7E-36	-0.15	
K14582	cis-1,2-dihydro-1,2-dihydroxynaphthalene/dibenzothiophene dihydrodiol dehydrogenase [EC:1.3.1.29 1.3.1.60]	-0.15	1.2E-36	1.7E-36	-0.15	
K14580	naphthalene 1,2-dioxygenase subunit beta [EC:1.14.12.12 1.14.12.23 1.14.12.24]	-0.15	1.2E-36	1.7E-36	-0.15	
K15020	acryloyl-coenzyme A reductase [EC:1.3.1.84]	-0.15	1.6E-36	2.3E-36	-0.15	
K00595	precorrin-6B C5,15-methyltransferase / cobalt-precorrin-6B C5,C15-methyltransferase [EC:2.1.1.132 2.1.1.289 2.1.1.196]	-0.15	1.8E-36	2.5E-36	-0.15	
K03823	phosphinothricin acetyltransferase [EC:2.3.1.183]	-0.15	2.0E-36	2.8E-36	-0.18	
K05362	UDP-N-acetylmuramoyl-L-alanyl-D-glutamate-L-lysine ligase [EC:6.3.2.7]	-0.15	2.0E-36	2.8E-36	-0.16	
K02821	ascorbate PTS system EIIA or EIIAB component [EC:2.7.1.194]	0.15	2.1E-36	3.0E-36	0.06	
K01507	inorganic pyrophosphatase [EC:3.6.1.1]	-0.15	3.2E-36	4.5E-36	-0.20	
K00152	salicylaldehyde dehydrogenase [EC:1.2.1.65]	-0.15	3.5E-36	4.9E-36	-0.17	
K01647	citrate synthase [EC:2.3.3.1]	-0.15	4.6E-36	6.5E-36	-0.24	
K01744	aspartate ammonia-lyase [EC:4.3.1.1]	0.15	9.0E-36	1.3E-35	0.22	

Predictor	Description	Pearson's r	P	FDR-adjusted P	Spearman's rho	Associated with fractures (P<0.05)
K04071	NA	-0.15	1.0E-35	1.4E-35	-0.16	
K01664	para-aminobenzoate synthetase component II [EC:2.6.1.85]	0.15	1.1E-35	1.6E-35	0.05	
K01271	Xaa-Pro dipeptidase [EC:3.4.13.9]	0.15	1.3E-35	1.8E-35	0.05	
K00184	dimethyl sulfoxide reductase iron-sulfur subunit	-0.15	1.7E-35	2.4E-35	-0.17	
K01625	2-dehydro-3-deoxyphosphogluconate aldolase / (4S)-4-hydroxy-2-oxoglutarate aldolase [EC:4.1.2.14 4.1.3.42]	0.15	1.7E-35	2.4E-35	0.22	
K08967	1,2-dihydroxy-3-keto-5-methylthiopentene dioxygenase [EC:1.13.11.53 1.13.11.54]	-0.15	2.2E-35	3.1E-35	-0.24	
K00215	4-hydroxy-tetrahydrodipicolinate reductase [EC:1.17.1.8]	-0.15	2.4E-35	3.4E-35	-0.18	
K01461	N-acyl-D-glutamate deacylase [EC:3.5.1.82]	-0.15	2.5E-35	3.5E-35	-0.16	
K13062	acyl homoserine lactone synthase [EC:2.3.1.184]	-0.15	4.5E-35	6.3E-35	-0.16	
K04075	tRNA(Ile)-lysidine synthase [EC:6.3.4.19]	-0.15	5.5E-35	7.7E-35	-0.17	
K00600	glycine hydroxymethyltransferase [EC:2.1.2.1]	-0.15	6.7E-35	9.4E-35	-0.18	yes
K10908	DNA-directed RNA polymerase, mitochondrial [EC:2.7.7.6]	-0.15	8.4E-35	1.2E-34	-0.15	
K06176	tRNA pseudouridine13 synthase [EC:5.4.99.27]	0.15	1.3E-34	1.8E-34	0.02	
K12567	titin [EC:2.7.11.1]	-0.15	1.3E-34	1.8E-34	-0.14	
K05956	geranylgeranyl transferase type-2 subunit beta [EC:2.5.1.60]	-0.15	1.5E-34	2.1E-34	-0.14	
K01202	galactosylceramidase [EC:3.2.1.46]	-0.15	1.6E-34	2.2E-34	-0.15	
K14652	3,4-dihydroxy 2-butanone 4-phosphate synthase / GTP cyclohydrolase II [EC:4.1.99.12 3.5.4.25]	-0.15	1.7E-34	2.4E-34	0.00	
K03395	aminoglycoside 3-N-acetyltransferase I [EC:2.3.1.60]	0.14	2.8E-34	3.9E-34	0.05	
K00630	glycerol-3-phosphate O-acyltransferase [EC:2.3.1.15]	-0.14	2.8E-34	3.9E-34	-0.14	
K00009	mannitol-1-phosphate 5-dehydrogenase [EC:1.1.1.17]	0.14	3.1E-34	4.3E-34	0.12	
K02822	ascorbate PTS system EIIB component [EC:2.7.1.194]	0.14	3.2E-34	4.5E-34	0.04	
K01406	serralysin [EC:3.4.24.40]	-0.14	4.3E-34	6.0E-34	-0.22	
K08325	NADP-dependent alcohol dehydrogenase [EC:1.1.-.-]	0.14	4.4E-34	6.1E-34	0.20	
K00872	homoserine kinase [EC:2.7.1.39]	-0.14	5.3E-34	7.4E-34	-0.18	
K01719	uroporphyrinogen-III synthase [EC:4.2.1.75]	0.14	5.6E-34	7.8E-34	0.24	
K01776	glutamate racemase [EC:5.1.1.3]	-0.14	5.8E-34	8.1E-34	-0.10	
K05966	triphosphoribosyl-dephospho-CoA synthase [EC:2.4.2.52]	0.14	6.2E-34	8.6E-34	0.03	
K00147	glutamate-5-semialdehyde dehydrogenase [EC:1.2.1.41]	-0.14	7.6E-34	1.1E-33	-0.15	
K11323	histone arginine demethylase JMJD6 [EC:1.14.11.-]	0.14	8.7E-34	1.2E-33	0.09	
K15524	mannosylglycerate hydrolase [EC:3.2.1.170]	0.14	2.1E-33	2.9E-33	0.04	
K00558	DNA (cytosine-5)-methyltransferase 1 [EC:2.1.1.37]	-0.14	2.1E-33	2.9E-33	-0.17	
K01347	IgA-specific serine endopeptidase [EC:3.4.21.72]	-0.14	2.3E-33	3.2E-33	-0.16	
K07559	putative RNA 2'-phosphotransferase [EC:2.7.1.-]	0.14	2.5E-33	3.5E-33	0.03	
K16044	scyllo-inositol 2-dehydrogenase (NADP+) [EC:1.1.1.371]	-0.14	5.0E-33	6.9E-33	-0.22	
K01481	protein-arginine deiminase [EC:3.5.3.15]	-0.14	7.0E-33	9.7E-33	-0.15	
K12250	pentalenene synthase [EC:4.2.3.7]	-0.14	8.4E-33	1.2E-32	-0.14	
K04340	scyllo-inosamine-4-phosphate amidinotransferase 1 [EC:2.1.4.2]	-0.14	8.4E-33	1.2E-32	-0.14	
K02427	23S rRNA (uridine2552-2'-O)-methyltransferase [EC:2.1.1.166]	0.14	9.0E-33	1.2E-32	0.01	
K03635	molybdopterin synthase catalytic subunit [EC:2.8.1.12]	0.14	1.4E-32	1.9E-32	0.00	
K00013	histidinol dehydrogenase [EC:1.1.1.23]	-0.14	1.5E-32	2.1E-32	-0.17	
K01318	glutamyl endopeptidase [EC:3.4.21.19]	-0.14	1.6E-32	2.2E-32	-0.14	yes
K01438	acetylornithine deacetylase [EC:3.5.1.16]	0.14	3.2E-32	4.4E-32	0.26	

Predictor	Description	Pearson's r	P	FDR-adjusted P	Spearman's rho	Associated with fractures (P<0.05)
K05599	anthranilate 1,2-dioxygenase (deaminating, decarboxylating) large subunit [EC:1.14.12.1]	0.14	3.7E-32	5.1E-32	0.08	
K05600	anthranilate 1,2-dioxygenase (deaminating, decarboxylating) small subunit [EC:1.14.12.1]	0.14	3.7E-32	5.1E-32	0.08	
K13967	N-acetylmannosamine-6-phosphate 2-epimerase / N-acetylmannosamine kinase [EC:5.1.3.9 2.7.1.60]	-0.14	5.7E-32	7.9E-32	-0.14	
K03684	ribonuclease D [EC:3.1.13.5]	-0.14	5.8E-32	8.0E-32	-0.19	
K13086	mannosylfructose-6-phosphate phosphatase [EC:3.1.3.79]	-0.14	7.0E-32	9.7E-32	-0.14	
K10710	fructoselysine 6-kinase [EC:2.7.1.218]	-0.14	7.2E-32	9.9E-32	-0.23	yes
K12556	penicillin-binding protein 2X	-0.14	9.7E-32	1.3E-31	-0.15	
K00852	ribokinase [EC:2.7.1.15]	-0.14	1.0E-31	1.4E-31	-0.18	
K11072	spermidine/putrescine transport system ATP-binding protein [EC:7.6.2.11]	0.14	1.0E-31	1.4E-31	0.22	
K01895	acetyl-CoA synthetase [EC:6.2.1.1]	0.14	1.5E-31	2.1E-31	0.19	
K12978	lipid A 4'-phosphatase [EC:3.1.3.-]	-0.14	1.6E-31	2.2E-31	-0.18	
K00209	enoyl-[acyl-carrier protein] reductase / trans-2-enoyl-CoA reductase (NAD+) [EC:1.3.1.9 1.3.1.44]	-0.14	1.8E-31	2.5E-31	-0.25	
K00222	Delta14-sterol reductase [EC:1.3.1.70]	-0.14	1.9E-31	2.6E-31	-0.14	
K00839	(S)-ureidoglycine---glyoxylate transaminase [EC:2.6.1.112]	-0.14	2.0E-31	2.8E-31	-0.23	yes
K00687	penicillin-binding protein 2B	-0.14	2.3E-31	3.2E-31	-0.15	
K00340	NADH-quinone oxidoreductase subunit K [EC:7.1.1.2]	0.14	2.9E-31	4.0E-31	0.21	
K08296	phosphohistidine phosphatase [EC:3.1.3.-]	0.14	3.6E-31	4.9E-31	0.04	
K02109	F-type H+-transporting ATPase subunit b	-0.14	3.8E-31	5.2E-31	-0.23	
K07316	adenine-specific DNA-methyltransferase [EC:2.1.1.72]	-0.14	4.1E-31	5.6E-31	-0.15	
K05977	2'-hydroxybiphenyl-2-sulfinate desulfinase [EC:3.13.1.3]	-0.14	5.3E-31	7.3E-31	-0.14	
K00240	succinate dehydrogenase / fumarate reductase, iron-sulfur subunit [EC:1.3.5.1 1.3.5.4]	-0.14	5.7E-31	7.8E-31	-0.20	
K01408	insulysin [EC:3.4.24.56]	-0.14	5.9E-31	8.1E-31	-0.14	
K03431	phosphoglucosamine mutase [EC:5.4.2.10]	-0.14	7.5E-31	1.0E-30	-0.18	
K01429	urease subunit beta [EC:3.5.1.5]	-0.14	7.5E-31	1.0E-30	-0.10	
K00818	acetylornithine aminotransferase [EC:2.6.1.11]	-0.14	8.0E-31	1.1E-30	-0.01	
K01963	acetyl-CoA carboxylase carboxyl transferase subunit beta [EC:6.4.1.2 2.1.3.15]	-0.14	8.3E-31	1.1E-30	-0.22	
K14468	malonyl-CoA reductase / 3-hydroxypropionate dehydrogenase (NADP+) [EC:1.2.1.75 1.1.1.298]	-0.14	1.1E-30	1.5E-30	-0.13	
K15052	propionyl-CoA carboxylase [EC:6.4.1.3 2.1.3.15]	-0.14	1.1E-30	1.5E-30	-0.13	
K14472	succinyl-CoA:(S)-malate CoA-transferase subunit B [EC:2.8.3.22]	-0.14	1.1E-30	1.5E-30	-0.13	
K03270	3-deoxy-D-manno-octulosonate 8-phosphate phosphatase (KDO 8-P phosphatase) [EC:3.1.3.45]	0.14	1.2E-30	1.6E-30	0.21	
K00842	NA	-0.14	1.2E-30	1.6E-30	-0.14	
K01295	glutamate carboxypeptidase [EC:3.4.17.11]	-0.14	2.9E-30	4.0E-30	-0.24	
K01934	5-formyltetrahydrofolate cyclo-ligase [EC:6.3.3.2]	-0.14	2.9E-30	4.0E-30	-0.16	
K00007	D-arabinitol 4-dehydrogenase [EC:1.1.1.11]	-0.14	3.0E-30	4.1E-30	-0.23	
K10210	diapolycopene oxygenase [EC:1.14.99.44]	-0.14	3.2E-30	4.4E-30	-0.15	
K16214	UDP-N-acetylglucosamine kinase [EC:2.7.1.176]	-0.14	3.4E-30	4.6E-30	-0.15	
K00996	undecaprenyl-phosphate galactose phosphotransferase [EC:2.7.8.6]	-0.14	3.8E-30	5.2E-30	-0.20	
K08566	plasminogen activator [EC:3.4.23.48]	0.14	5.1E-30	6.9E-30	0.07	
K01772	protoporphyrin/coproporphyrin ferrochelatase [EC:4.99.1.1 4.99.1.9]	0.13	5.9E-30	8.0E-30	0.02	
K00034	glucose 1-dehydrogenase [EC:1.1.1.47]	-0.13	9.1E-30	1.2E-29	-0.17	
K14260	alanine-synthesizing transaminase [EC:2.6.1.66 2.6.1.2]	0.13	1.1E-29	1.5E-29	0.05	
K15634	2,3-bisphosphoglycerate-dependent phosphoglycerate mutase [EC:5.4.2.11]	-0.13	1.1E-29	1.5E-29	-0.19	

Predictor	Description	Pearson's r	P	FDR-adjusted P	Spearman's rho	Associated with fractures (P<0.05)
K01489	cytidine deaminase [EC:3.5.4.5]	0.13	1.2E-29	1.6E-29	0.20	
K01494	dCTP deaminase [EC:3.5.4.13]	-0.13	1.5E-29	2.0E-29	-0.19	
K13876	2-keto-3-deoxy-L-arabinonate dehydratase [EC:4.2.1.43]	-0.13	2.2E-29	3.0E-29	-0.15	
K01626	3-deoxy-7-phosphoheptulonate synthase [EC:2.5.1.54]	-0.13	4.2E-29	5.7E-29	-0.19	
K07259	serine-type D-Ala-D-Ala carboxypeptidase/endopeptidase (penicillin-binding protein 4) [EC:3.4.16.4 3.4.21.-]	0.13	4.9E-29	6.6E-29	0.24	
K05879	phosphoenolpyruvate---glycerone phosphotransferase subunit DhaL [EC:2.7.1.121]	0.13	7.8E-29	1.1E-28	0.00	
K01385	thermopsin [EC:3.4.23.42]	-0.13	8.5E-29	1.2E-28	-0.13	
K14082	[methyl-Co(III) methylamine-specific corrinoid protein]:coenzyme M methyltransferase [EC:2.1.1.247]	-0.13	8.8E-29	1.2E-28	-0.13	
K14982	two-component system, OmpR family, sensor histidine kinase CiaH [EC:2.7.13.3]	-0.13	8.9E-29	1.2E-28	-0.15	
K04072	acetaldehyde dehydrogenase / alcohol dehydrogenase [EC:1.2.1.10 1.1.1.1]	-0.13	1.1E-28	1.5E-28	-0.18	
K06151	gluconate 2-dehydrogenase alpha chain [EC:1.1.99.3]	-0.13	1.4E-28	1.9E-28	-0.22	
K13037	L-alanine-L-anticapsin ligase [EC:6.3.2.49]	-0.13	1.6E-28	2.2E-28	-0.13	
K00031	isocitrate dehydrogenase [EC:1.1.1.42]	-0.13	2.1E-28	2.8E-28	-0.16	
K00993	ethanolaminephosphotransferase [EC:2.7.8.1]	-0.13	2.6E-28	3.5E-28	-0.13	yes
K14742	tRNA threonylcarbamoyladenosine biosynthesis protein TsaB	0.13	5.2E-28	7.0E-28	0.21	
K01419	ATP-dependent HslUV protease, peptidase subunit HslV [EC:3.4.25.2]	0.13	6.9E-28	9.3E-28	0.01	
K03816	xanthine phosphoribosyltransferase [EC:2.4.2.22]	-0.13	7.7E-28	1.0E-27	-0.07	
K01246	DNA-3-methyladenine glycosylase I [EC:3.2.2.20]	0.13	8.9E-28	1.2E-27	0.18	
K01951	GMP synthase (glutamine-hydrolysing) [EC:6.3.5.2]	-0.13	9.1E-28	1.2E-27	-0.06	
K00767	nicotinate-nucleotide pyrophosphorylase (carboxylating) [EC:2.4.2.19]	-0.13	1.0E-27	1.3E-27	-0.15	
K03215	23S rRNA (uracil1939-C5)-methyltransferase [EC:2.1.1.190]	-0.13	1.3E-27	1.7E-27	-0.18	
K11627	pyrrolysyl-tRNA synthetase [EC:6.1.1.26]	-0.13	1.5E-27	2.0E-27	-0.14	
K06033	arylmalonate decarboxylase [EC:4.1.1.76]	-0.13	1.7E-27	2.3E-27	-0.13	
K01156	type III restriction enzyme [EC:3.1.21.5]	-0.13	1.7E-27	2.3E-27	-0.14	
K02551	2-succinyl-5-enolpyruvyl-6-hydroxy-3-cyclohexene-1-carboxylate synthase [EC:2.2.1.9]	0.13	1.8E-27	2.4E-27	0.18	
K00757	uridine phosphorylase [EC:2.4.2.3]	0.13	3.0E-27	4.0E-27	0.20	
K01315	plasminogen [EC:3.4.21.7]	-0.13	3.6E-27	4.8E-27	-0.13	
K01495	GTP cyclohydrolase IA [EC:3.5.4.16]	-0.13	3.8E-27	5.1E-27	-0.16	
K00940	nucleoside-diphosphate kinase [EC:2.7.4.6]	0.13	5.0E-27	6.7E-27	0.18	
K11784	cyclic dehypoxanthinyl futalosine synthase [EC:1.21.98.1]	-0.13	5.1E-27	6.8E-27	-0.08	
K11785	1,4-dihydroxy-6-naphthoate synthase [EC:1.14.-.-]	-0.13	5.2E-27	7.0E-27	-0.08	
K01955	carbamoyl-phosphate synthase large subunit [EC:6.3.5.5]	-0.13	6.7E-27	9.0E-27	0.02	
K01633	7,8-dihydroneopterin aldolase/epimerase/oxygenase [EC:4.1.2.25 5.1.99.8 1.13.11.81]	0.13	7.4E-27	9.9E-27	0.18	
K00239	succinate dehydrogenase / fumarate reductase, flavoprotein subunit [EC:1.3.5.1 1.3.5.4]	-0.13	9.7E-27	1.3E-26	-0.16	
K00145	N-acetyl-gamma-glutamyl-phosphate reductase [EC:1.2.1.38]	-0.13	1.0E-26	1.3E-26	-0.15	
K01661	naphthoate synthase [EC:4.1.3.36]	0.13	1.0E-26	1.3E-26	0.18	
K08307	membrane-bound lytic murein transglycosylase D [EC:4.2.2.-]	0.13	1.0E-26	1.3E-26	0.20	
K03269	UDP-2,3-diacylglucosamine hydrolase [EC:3.6.1.54]	0.13	1.1E-26	1.5E-26	0.20	
K01089	imidazoleglycerol-phosphate dehydratase / histidinol-phosphatase [EC:4.2.1.19 3.1.3.15]	0.13	1.2E-26	1.6E-26	0.20	
K01119	2',3'-cyclic-nucleotide 2'-phosphodiesterase / 3'-nucleotidase [EC:3.1.4.16 3.1.3.6]	0.13	1.2E-26	1.6E-26	0.20	
K01187	alpha-glucosidase [EC:3.2.1.20]	-0.13	1.3E-26	1.7E-26	0.03	
K01818	L-fucose/D-arabinose isomerase [EC:5.3.1.25 5.3.1.3]	0.13	2.4E-26	3.2E-26	0.21	

Predictor	Description	Pearson's r	P	FDR-adjusted P	Spearman's rho	Associated with fractures (P<0.05)
K00613	glycine amidinotransferase [EC:2.1.4.1]	-0.13	2.5E-26	3.3E-26	-0.14	
K00689	dextransucrase [EC:2.4.1.5]	-0.13	2.8E-26	3.7E-26	-0.17	
K02536	UDP-3-O-[3-hydroxymyristoyl] glucosamine N-acyltransferase [EC:2.3.1.191]	0.13	2.9E-26	3.9E-26	0.20	
K06133	4'-phosphopantetheinyl transferase [EC:2.7.8.-]	-0.13	3.0E-26	4.0E-26	-0.18	
K12745	deacetoxycephalosporin-C hydroxylase [EC:1.14.11.26]	-0.13	3.3E-26	4.4E-26	-0.12	
K12744	deacetoxycephalosporin-C synthase [EC:1.14.20.1]	-0.13	3.3E-26	4.4E-26	-0.12	
K04126	isopenicillin-N synthase [EC:1.21.3.1]	-0.13	3.3E-26	4.4E-26	-0.12	
K12743	N-(5-amino-5-carboxypentanoyl)-L-cysteinyl-D-valine synthase [EC:6.3.2.26]	-0.13	3.3E-26	4.4E-26	-0.12	
K00213	7-dehydrocholesterol reductase [EC:1.3.1.21]	-0.13	5.8E-26	7.7E-26	-0.13	
K12447	UDP-sugar pyrophosphorylase [EC:2.7.7.64]	-0.13	6.2E-26	8.2E-26	-0.13	
K12984	(heptosyl)LPS beta-1,4-glucosyltransferase [EC:2.4.1.-]	0.12	8.3E-26	1.1E-25	0.01	
K01914	aspartate--ammonia ligase [EC:6.3.1.1]	0.12	9.0E-26	1.2E-25	0.19	
K01815	4-deoxy-L-threo-5-hexosulose-uronate ketol-isomerase [EC:5.3.1.17]	0.12	1.1E-25	1.5E-25	0.17	
K00692	levansucrase [EC:2.4.1.10]	-0.12	1.8E-25	2.4E-25	-0.23	
K06019	pyrophosphatase PpaX [EC:3.6.1.1]	-0.12	2.1E-25	2.8E-25	-0.16	
K00046	gluconate 5-dehydrogenase [EC:1.1.1.69]	0.12	2.6E-25	3.4E-25	0.15	
K10620	2,3-dihydroxy-2,3-dihydro-p-cumate dehydrogenase [EC:1.3.1.58]	-0.12	2.7E-25	3.6E-25	-0.12	
K01696	tryptophan synthase beta chain [EC:4.2.1.20]	-0.12	2.9E-25	3.8E-25	-0.22	
K00891	shikimate kinase [EC:2.7.1.71]	0.12	4.3E-25	5.7E-25	0.12	
K00548	5-methyltetrahydrofolate--homocysteine methyltransferase [EC:2.1.1.13]	-0.12	4.5E-25	5.9E-25	-0.15	
K02363	2,3-dihydroxybenzoate---[aryl-carrier protein] ligase [EC:6.3.2.14 6.2.1.71]	0.12	4.8E-25	6.3E-25	-0.01	
K00135	succinate-semialdehyde dehydrogenase / glutarate-semialdehyde dehydrogenase [EC:1.2.1.16 1.2.1.79 1.2.1.20]	0.12	4.9E-25	6.5E-25	-0.01	
K01652	acetolactate synthase I/II/III large subunit [EC:2.2.1.6]	0.12	6.0E-25	7.9E-25	0.07	
K11755	phosphoribosyl-AMP cyclohydrolase / phosphoribosyl-ATP pyrophosphohydrolase [EC:3.5.4.19 3.6.1.31]	0.12	7.9E-25	1.0E-24	0.19	
K01258	tripeptide aminopeptidase [EC:3.4.11.4]	-0.12	9.6E-25	1.3E-24	-0.15	
K07256	taurine dehydrogenase large subunit [EC:1.4.2.-]	-0.12	9.7E-25	1.3E-24	-0.12	
K02527	3-deoxy-D-manno-octulosonic-acid transferase [EC:2.4.99.12 2.4.99.13 2.4.99.14 2.4.99.15]	0.12	9.9E-25	1.3E-24	0.19	
K03820	apolipoprotein N-acyltransferase [EC:2.3.1.269]	0.12	1.0E-24	1.3E-24	0.09	
K00950	2-amino-4-hydroxy-6-hydroxymethyldihydropteridine diphosphokinase [EC:2.7.6.3]	0.12	1.6E-24	2.1E-24	0.24	
K13281	UV DNA damage endonuclease [EC:3.-.-.-]	-0.12	1.6E-24	2.1E-24	-0.19	
K03648	uracil-DNA glycosylase [EC:3.2.2.27]	-0.12	1.6E-24	2.1E-24	-0.15	yes
K05783	dihydroxycyclohexadiene carboxylate dehydrogenase [EC:1.3.1.25 1.3.1.-]	-0.12	1.8E-24	2.4E-24	-0.21	
K02226	alpha-ribazole phosphatase [EC:3.1.3.73]	0.12	1.9E-24	2.5E-24	0.18	
K00123	formate dehydrogenase major subunit [EC:1.17.1.9]	0.12	2.0E-24	2.6E-24	0.00	
K12977	lipid A 1-phosphatase [EC:3.1.3.-]	-0.12	2.1E-24	2.8E-24	-0.12	
K03517	quinolinate synthase [EC:2.5.1.72]	-0.12	2.8E-24	3.7E-24	-0.14	
K01961	acetyl-CoA carboxylase, biotin carboxylase subunit [EC:6.4.1.2 6.3.4.14]	0.12	2.9E-24	3.8E-24	0.22	
K01812	glucuronate isomerase [EC:5.3.1.12]	0.12	3.6E-24	4.7E-24	0.18	
K01749	hydroxymethylbilane synthase [EC:2.5.1.61]	-0.12	5.3E-24	6.9E-24	-0.18	
K01834	2,3-bisphosphoglycerate-dependent phosphoglycerate mutase [EC:5.4.2.11]	-0.12	5.6E-24	7.3E-24	-0.08	yes
K01159	crossover junction endodeoxyribonuclease RuvC [EC:3.1.21.10]	-0.12	6.5E-24	8.5E-24	-0.17	
K06969	23S rRNA (cytosine1962-C5)-methyltransferase [EC:2.1.1.191]	-0.12	9.2E-24	1.2E-23	-0.11	

Predictor	Description	Pearson's r	P	FDR-adjusted P	Spearman's rho	Associated with fractures (P<0.05)
K03186	flavin prenyltransferase [EC:2.5.1.129]	0.12	9.7E-24	1.3E-23	0.07	
K01247	DNA-3-methyladenine glycosylase II [EC:3.2.2.21]	0.12	1.0E-23	1.3E-23	0.00	
K00387	sulfite oxidase [EC:1.8.3.1]	-0.12	1.0E-23	1.3E-23	-0.12	
K02472	UDP-N-acetyl-D-mannosaminuronic acid dehydrogenase [EC:1.1.1.336]	0.12	1.2E-23	1.6E-23	0.23	
K01502	aliphatic nitrilase [EC:3.5.5.7]	-0.12	1.5E-23	2.0E-23	-0.20	yes
K00796	dihydropteroate synthase [EC:2.5.1.15]	0.12	2.8E-23	3.7E-23	0.17	
K00278	L-aspartate oxidase [EC:1.4.3.16]	-0.12	2.9E-23	3.8E-23	-0.12	
K10677	inulin fructotransferase (DFA-I-forming) [EC:4.2.2.17]	-0.12	3.0E-23	3.9E-23	-0.11	
K01681	aconitate hydratase [EC:4.2.1.3]	-0.12	3.2E-23	4.2E-23	-0.17	
K10909	two-component system, autoinducer 2 sensor kinase/phosphatase LuxQ [EC:2.7.13.3 3.1.3.-]	-0.12	4.1E-23	5.3E-23	-0.12	
K11175	phosphoribosylglycinamide formyltransferase 1 [EC:2.1.2.2]	-0.12	6.5E-23	8.5E-23	-0.16	
K12658	4-hydroxyproline epimerase [EC:5.1.1.8]	-0.12	6.7E-23	8.7E-23	-0.21	
K00494	alkanal monooxygenase alpha chain [EC:1.14.14.3]	0.12	7.0E-23	9.1E-23	0.06	
K15854	alkanal monooxygenase beta chain [EC:1.14.14.3]	0.12	7.0E-23	9.1E-23	0.06	
K03400	long-chain-fatty-acyl-CoA reductase [EC:1.2.1.50]	0.12	7.0E-23	9.1E-23	0.06	
K11633	two-component system, OmpR family, sensor histidine kinase YxdK [EC:2.7.13.3]	-0.12	7.7E-23	1.0E-22	-0.16	
K01524	exopolyphosphatase / guanosine-5'-triphosphate,3'-diphosphate pyrophosphatase [EC:3.6.1.11 3.6.1.40]	-0.12	1.4E-22	1.8E-22	-0.17	
K02474	UDP-N-acetyl-D-glucosamine/UDP-N-acetyl-D-galactosamine dehydrogenase [EC:1.1.1.136 1.1.1.-]	-0.12	1.6E-22	2.1E-22	-0.10	
K01972	DNA ligase (NAD+) [EC:6.5.1.2]	0.12	1.9E-22	2.5E-22	0.10	
K03367	D-alanine--poly(phosphoribitol) ligase subunit 1 [EC:6.1.1.13]	-0.12	1.9E-22	2.5E-22	-0.14	
K01486	adenine deaminase [EC:3.5.4.2]	-0.12	2.0E-22	2.6E-22	-0.19	
K00974	tRNA nucleotidyltransferase (CCA-adding enzyme) [EC:2.7.7.72 3.1.3.- 3.1.4.-]	-0.12	2.4E-22	3.1E-22	-0.20	
K00928	aspartate kinase [EC:2.7.2.4]	-0.12	2.8E-22	3.6E-22	-0.13	
K11640	two-component system, LytTR family, sensor histidine kinase NatK [EC:2.7.13.3]	-0.11	3.8E-22	4.9E-22	-0.11	
K05369	15,16-dihydrobiliverdin:ferredoxin oxidoreductase [EC:1.3.7.2]	-0.11	3.8E-22	4.9E-22	-0.11	
K05356	all-trans-nonaprenyl-diphosphate synthase [EC:2.5.1.84 2.5.1.85]	-0.11	3.8E-22	4.9E-22	-0.11	
K15226	arogenate dehydrogenase (NADP+) [EC:1.3.1.78]	-0.11	3.8E-22	4.9E-22	-0.11	
K14331	fatty aldehyde decarbonylase [EC:4.1.99.5]	-0.11	3.8E-22	4.9E-22	-0.11	
K14330	fatty aldehyde-generating acyl-ACP reductase [EC:1.2.1.80]	-0.11	3.8E-22	4.9E-22	-0.11	
K05572	NAD(P)H-quinone oxidoreductase subunit 1 [EC:7.1.1.2]	-0.11	3.8E-22	4.9E-22	-0.11	
K05585	NAD(P)H-quinone oxidoreductase subunit N [EC:7.1.1.2]	-0.11	3.8E-22	4.9E-22	-0.11	
K02288	phycocyanobilin lyase subunit alpha [EC:4.4.1.32]	-0.11	3.8E-22	4.9E-22	-0.11	
K05370	phycoerythrobilin:ferredoxin oxidoreductase [EC:1.3.7.3]	-0.11	3.8E-22	4.9E-22	-0.11	
K11520	two-component system, OmpR family, manganese sensing sensor histidine kinase [EC:2.7.13.3]	-0.11	3.8E-22	4.9E-22	-0.11	
K03867	UDP-glucose:tetrahydrobiopterin glucosyltransferase [EC:2.4.1.-]	-0.11	3.8E-22	4.9E-22	-0.11	
K08475	two-component system, NtrC family, phosphoglycerate transport system sensor histidine kinase PgtB [EC:2.7.13.3]	-0.11	4.4E-22	5.7E-22	-0.17	
K01658	anthranilate synthase component II [EC:4.1.3.27]	-0.11	4.4E-22	5.7E-22	-0.01	
K01533	P-type Cu2+ transporter [EC:7.2.2.9]	-0.11	6.7E-22	8.6E-22	-0.02	yes
K12137	hydrogenase-4 component B [EC:1.-.-.-]	0.11	8.1E-22	1.0E-21	0.09	
K02759	cellobiose PTS system EIIA component [EC:2.7.1.196 2.7.1.205]	-0.11	8.7E-22	1.1E-21	-0.19	
K01193	beta-fructofuranosidase [EC:3.2.1.26]	-0.11	1.3E-21	1.7E-21	-0.16	
K01565	N-sulfoglucosamine sulfohydrolase [EC:3.10.1.1]	-0.11	1.8E-21	2.3E-21	-0.13	

Predictor	Description	Pearson's r	P	FDR-adjusted P	Spearman's rho	Associated with fractures (P<0.05)
K03752	molybdenum cofactor guanylyltransferase [EC:2.7.7.77]	0.11	1.9E-21	2.4E-21	-0.04	
K05286	GPI mannosyltransferase 3 [EC:2.4.1.-]	-0.11	2.0E-21	2.6E-21	-0.12	
K05579	NAD(P)H-quinone oxidoreductase subunit H [EC:7.1.1.2]	-0.11	2.1E-21	2.7E-21	-0.11	
K05581	NAD(P)H-quinone oxidoreductase subunit J [EC:7.1.1.2]	-0.11	2.1E-21	2.7E-21	-0.11	
K05582	NAD(P)H-quinone oxidoreductase subunit K [EC:7.1.1.2]	-0.11	2.1E-21	2.7E-21	-0.11	
K00281	glycine dehydrogenase [EC:1.4.4.2]	0.11	2.4E-21	3.1E-21	0.19	
K16176	methylamine---corrinoid protein Co-methyltransferase [EC:2.1.1.248]	-0.11	2.6E-21	3.3E-21	-0.10	
K08289	phosphoribosylglycinamide formyltransferase 2 [EC:6.3.1.21]	0.11	2.7E-21	3.5E-21	0.17	
K14159	ribonuclease HI / DNA polymerase III subunit epsilon [EC:3.1.26.4 2.7.7.7]	-0.11	3.0E-21	3.8E-21	-0.11	
K03658	DNA helicase IV [EC:3.6.4.12]	-0.11	3.4E-21	4.4E-21	-0.15	
K05592	ATP-dependent RNA helicase DeaD [EC:3.6.4.13]	0.11	3.5E-21	4.5E-21	0.21	
K02768	fructose PTS system EIIA component [EC:2.7.1.202]	0.11	4.1E-21	5.2E-21	-0.01	
K13789	geranylgeranyl diphosphate synthase, type II [EC:2.5.1.1 2.5.1.10 2.5.1.29]	-0.11	4.4E-21	5.6E-21	0.03	
K07588	GTPase [EC:3.6.5.-]	0.11	4.7E-21	6.0E-21	0.14	
K16153	glycogen phosphorylase/synthase [EC:2.4.1.1 2.4.1.11]	-0.11	5.4E-21	6.9E-21	-0.06	
K10353	deoxyadenosine kinase [EC:2.7.1.76]	-0.11	8.9E-21	1.1E-20	-0.14	
K13689	beta-1,4-N-acetylgalactosaminyltransferase [EC:2.4.1.-]	-0.11	1.1E-20	1.4E-20	-0.11	
K01814	phosphoribosylformimino-5-aminoimidazole carboxamide ribotide isomerase [EC:5.3.1.16]	-0.11	1.1E-20	1.4E-20	-0.16	
K08352	thiosulfate reductase / polysulfide reductase chain A [EC:1.8.5.5]	-0.11	1.6E-20	2.0E-20	-0.15	
K01739	cystathionine gamma-synthase [EC:2.5.1.48]	0.11	2.2E-20	2.8E-20	0.03	
K13252	putrescine carbamoyltransferase [EC:2.1.3.6]	-0.11	2.3E-20	2.9E-20	-0.14	
K16038	N-methyltransferase [EC:2.1.1.-]	-0.11	2.3E-20	2.9E-20	-0.11	yes
K00979	3-deoxy-manno-octulosonate cytidylyltransferase (CMP-KDO synthetase) [EC:2.7.7.38]	0.11	2.4E-20	3.1E-20	0.19	
K01845	glutamate-1-semialdehyde 2,1-aminomutase [EC:5.4.3.8]	-0.11	4.4E-20	5.6E-20	-0.17	
K06178	23S rRNA pseudouridine2605 synthase [EC:5.4.99.22]	-0.11	6.1E-20	7.8E-20	-0.14	yes
K01337	lysyl endopeptidase [EC:3.4.21.50]	-0.11	7.4E-20	9.4E-20	-0.12	
K05878	phosphoenolpyruvate---glycerone phosphotransferase subunit DhaK [EC:2.7.1.121]	0.11	7.5E-20	9.5E-20	-0.01	
K00338	NADH-quinone oxidoreductase subunit I [EC:7.1.1.2]	0.11	7.5E-20	9.5E-20	0.18	
K03182	4-hydroxy-3-polyprenylbenzoate decarboxylase [EC:4.1.1.98]	0.11	7.8E-20	9.9E-20	0.08	
K00005	glycerol dehydrogenase [EC:1.1.1.6]	0.11	8.2E-20	1.0E-19	0.00	
K03735	ethanolamine ammonia-lyase large subunit [EC:4.3.1.7]	-0.11	8.6E-20	1.1E-19	-0.19	
K01752	L-serine dehydratase [EC:4.3.1.17]	-0.11	9.0E-20	1.1E-19	-0.16	
K01546	potassium-transporting ATPase potassium-binding subunit	0.11	9.8E-20	1.2E-19	0.15	
K00342	NADH-quinone oxidoreductase subunit M [EC:7.1.1.2]	0.11	1.1E-19	1.4E-19	0.18	
K01277	dipeptidyl-peptidase III [EC:3.4.14.4]	-0.11	1.2E-19	1.5E-19	-0.08	
K16035	7-O-carbamoyltransferase [EC:2.1.3.-]	-0.11	1.2E-19	1.5E-19	-0.11	yes
K00097	4-hydroxythreonine-4-phosphate dehydrogenase [EC:1.1.1.262]	0.11	1.3E-19	1.6E-19	0.18	
K02231	adenosylcobinamide kinase / adenosylcobinamide-phosphate guanylyltransferase [EC:2.7.1.156 2.7.7.62]	0.11	1.6E-19	2.0E-19	0.18	
K01897	long-chain acyl-CoA synthetase [EC:6.2.1.3]	-0.11	1.6E-19	2.0E-19	-0.02	yes
K01821	4-oxalocrotonate tautomerase [EC:5.3.2.6]	0.11	2.1E-19	2.7E-19	0.06	
K03052	DNA-directed RNA polymerase subunit G [EC:2.7.7.6]	-0.11	2.2E-19	2.8E-19	-0.10	
K01058	phospholipase A1/A2 [EC:3.1.1.32 3.1.1.4]	0.11	2.4E-19	3.0E-19	0.04	

Predictor	Description	Pearson's r	P	FDR-adjusted P	Spearman's rho	Associated with fractures (P<0.05)
K00625	phosphate acetyltransferase [EC:2.3.1.8]	-0.11	2.7E-19	3.4E-19	0.02	
K00850	6-phosphofructokinase 1 [EC:2.7.1.11]	-0.11	3.2E-19	4.0E-19	0.03	
K13307	dTDP-3-amino-3,6-dideoxy-alpha-D-glucopyranose N,N-dimethyltransferase [EC:2.1.1.235]	-0.11	3.5E-19	4.4E-19	-0.10	
K15944	nogalaviketone/aklaviketone reductase [EC:1.1.1.- 1.1.1.362]	-0.11	3.5E-19	4.4E-19	-0.10	
K12674	(carboxyethyl)arginine beta-lactam-synthase [EC:6.3.3.4]	-0.11	3.6E-19	4.5E-19	-0.10	
K12675	clavaminate synthase [EC:1.14.11.21]	-0.11	3.6E-19	4.5E-19	-0.10	
K12673	N2-(2-carboxyethyl)arginine synthase [EC:2.5.1.66]	-0.11	3.6E-19	4.5E-19	-0.10	
K13061	acyl homoserine lactone synthase [EC:2.3.1.184]	-0.11	3.9E-19	4.9E-19	-0.18	
K06041	arabinose-5-phosphate isomerase [EC:5.3.1.13]	0.11	4.2E-19	5.3E-19	0.12	
K00971	mannose-1-phosphate guanylyltransferase [EC:2.7.7.13]	0.11	4.7E-19	5.9E-19	0.20	
K00590	site-specific DNA-methyltransferase (cytosine-N4-specific) [EC:2.1.1.113]	0.11	4.8E-19	6.0E-19	0.10	
K13930	triphosphoribosyl-dephospho-CoA synthase [EC:2.4.2.52]	-0.11	5.7E-19	7.2E-19	-0.20	
K06879	7-cyano-7-deazaguanine reductase [EC:1.7.1.13]	0.11	5.8E-19	7.3E-19	0.04	
K03800	lipoate---protein ligase [EC:6.3.1.20]	-0.11	5.9E-19	7.4E-19	-0.15	
K07964	heparanase [EC:3.2.1.166]	-0.11	7.4E-19	9.3E-19	-0.10	
K03407	two-component system, chemotaxis family, sensor kinase CheA [EC:2.7.13.3]	-0.11	9.8E-19	1.2E-18	-0.19	
K05367	penicillin-binding protein 1C [EC:2.4.1.129]	0.10	1.0E-18	1.3E-18	0.08	
K00645	[acyl-carrier-protein] S-malonyltransferase [EC:2.3.1.39]	-0.10	1.1E-18	1.4E-18	-0.13	
K09880	enolase-phosphatase E1 [EC:3.1.3.77]	-0.10	1.2E-18	1.5E-18	-0.21	
K01620	threonine aldolase [EC:4.1.2.48]	-0.10	1.6E-18	2.0E-18	-0.17	
K03381	catechol 1,2-dioxygenase [EC:1.13.11.1]	-0.10	1.6E-18	2.0E-18	-0.20	
K12999	glucosyltransferase [EC:2.4.1.-]	-0.10	1.7E-18	2.1E-18	-0.11	
K03077	L-ribulose-5-phosphate 4-epimerase [EC:5.1.3.4]	0.10	2.1E-18	2.6E-18	0.04	
K02548	1,4-dihydroxy-2-naphthoate polyprenyltransferase [EC:2.5.1.74]	0.10	2.2E-18	2.7E-18	0.16	
K06001	tryptophan synthase beta chain [EC:4.2.1.20]	-0.10	2.2E-18	2.7E-18	-0.03	
K00821	acetylornithine/N-succinyldiaminopimelate aminotransferase [EC:2.6.1.11 2.6.1.17]	-0.10	2.9E-18	3.6E-18	-0.18	
K01390	IgA-specific metalloendopeptidase [EC:3.4.24.13]	-0.10	3.1E-18	3.9E-18	-0.14	
K08693	2',3'-cyclic-nucleotide 2'-phosphodiesterase / 3'-nucleotidase / 5'-nucleotidase [EC:3.1.4.16 3.1.3.6 3.1.3.5]	-0.10	3.6E-18	4.5E-18	-0.14	
K01911	o-succinylbenzoate---CoA ligase [EC:6.2.1.26]	0.10	5.3E-18	6.6E-18	0.19	
K10150	cysteine synthase / O-phosphoserine sulfhydrylase / cystathionine beta-synthase [EC:2.5.1.47 2.5.1.65 4.2.1.22]	-0.10	5.7E-18	7.1E-18	-0.10	
K00324	H+-translocating NAD(P) transhydrogenase subunit alpha [EC:1.6.1.2 7.1.1.1]	-0.10	5.8E-18	7.2E-18	-0.15	
K01787	N-acylglucosamine 2-epimerase [EC:5.1.3.8]	-0.10	6.5E-18	8.1E-18	0.01	
K00528	ferredoxin/flavodoxin---NADP+ reductase [EC:1.18.1.2 1.19.1.1]	-0.10	9.0E-18	1.1E-17	-0.13	
K08964	methylthioribulose-1-phosphate dehydratase [EC:4.2.1.109]	-0.10	1.0E-17	1.2E-17	-0.21	
K00113	glycerol-3-phosphate dehydrogenase subunit C	0.10	1.0E-17	1.2E-17	0.04	
K00112	glycerol-3-phosphate dehydrogenase subunit B [EC:1.1.5.3]	0.10	1.0E-17	1.2E-17	0.04	
K02492	glutamyl-tRNA reductase [EC:1.2.1.70]	-0.10	1.1E-17	1.4E-17	-0.17	
K01209	alpha-L-arabinofuranosidase [EC:3.2.1.55]	-0.10	1.2E-17	1.5E-17	0.03	yes
K00208	enoyl-[acyl-carrier protein] reductase I [EC:1.3.1.9 1.3.1.10]	0.10	1.3E-17	1.6E-17	0.18	
K01755	argininosuccinate lyase [EC:4.3.2.1]	0.10	1.4E-17	1.7E-17	0.15	
K02851	UDP-GlcNAc:undecaprenyl-phosphate/decaprenyl-phosphate GlcNAc-1-phosphate transferase [EC:2.7.8.33 2.7.8.35]	-0.10	1.5E-17	1.9E-17	-0.15	
K10441	ribose transport system ATP-binding protein [EC:7.5.2.7]	0.10	1.5E-17	1.9E-17	-0.05	

Predictor	Description	Pearson's r	P	FDR-adjusted P	Spearman's rho	Associated with fractures (P<0.05)
K01791	UDP-N-acetylglucosamine 2-epimerase (non-hydrolysing) [EC:5.1.3.14]	0.10	1.8E-17	2.2E-17	0.23	
K08255	CoA-disulfide reductase [EC:1.8.1.14]	-0.10	2.5E-17	3.1E-17	-0.10	
K12551	monofunctional glycosyltransferase [EC:2.4.1.129]	-0.10	2.5E-17	3.1E-17	-0.10	
K00111	glycerol-3-phosphate dehydrogenase [EC:1.1.5.3]	-0.10	2.5E-17	3.1E-17	-0.14	
K01433	formyltetrahydrofolate deformylase [EC:3.5.1.10]	0.10	3.1E-17	3.8E-17	0.20	
K01902	succinyl-CoA synthetase alpha subunit [EC:6.2.1.5]	-0.10	4.5E-17	5.6E-17	-0.14	
K15866	2-(1,2-epoxy-1,2-dihydrophenyl)acetyl-CoA isomerase [EC:5.3.3.18]	0.10	4.9E-17	6.1E-17	-0.08	
K01035	acetate CoA/acetoacetate CoA-transferase beta subunit [EC:2.8.3.8 2.8.3.9]	0.10	5.1E-17	6.3E-17	0.04	
K00537	arsenate reductase (glutaredoxin) [EC:1.20.4.1]	0.10	5.6E-17	6.9E-17	0.04	
K01903	succinyl-CoA synthetase beta subunit [EC:6.2.1.5]	-0.10	9.1E-17	1.1E-16	-0.14	
K13745	L-2,4-diaminobutyrate decarboxylase [EC:4.1.1.86]	-0.10	1.0E-16	1.2E-16	-0.20	
K01151	deoxyribonuclease IV [EC:3.1.21.2]	-0.10	1.2E-16	1.5E-16	-0.13	
K09458	3-oxoacyl-[acyl-carrier-protein] synthase II [EC:2.3.1.179]	-0.10	1.2E-16	1.5E-16	-0.15	
K03469	ribonuclease HI [EC:3.1.26.4]	-0.10	1.3E-16	1.6E-16	-0.13	
K08589	gingipain R [EC:3.4.22.37]	-0.10	1.7E-16	2.1E-16	-0.14	
K11987	prostaglandin-endoperoxide synthase 2 [EC:1.14.99.1]	-0.10	1.7E-16	2.1E-16	-0.11	
K01665	para-aminobenzoate synthetase component I [EC:2.6.1.85]	0.10	1.9E-16	2.3E-16	0.18	
K01042	L-seryl-tRNA(Ser) seleniumtransferase [EC:2.9.1.1]	0.10	2.4E-16	3.0E-16	-0.02	
K01759	lactoylglutathione lyase [EC:4.4.1.5]	0.10	2.7E-16	3.3E-16	0.11	
K03412	two-component system, chemotaxis family, protein-glutamate methylesterase/glutaminase [EC:3.1.1.61 3.5.1.44]	0.10	3.4E-16	4.2E-16	-0.07	
K01788	N-acylglucosamine-6-phosphate 2-epimerase [EC:5.1.3.9]	0.10	3.5E-16	4.3E-16	-0.02	
K02744	N-acetylgalactosamine PTS system EIIA component [EC:2.7.1.-]	0.10	3.9E-16	4.8E-16	-0.03	
K01733	threonine synthase [EC:4.2.3.1]	-0.10	4.1E-16	5.0E-16	-0.09	
K01737	6-pyruvoyltetrahydropterin/6-carboxytetrahydropterin synthase [EC:4.2.3.12 4.1.2.50]	0.10	4.4E-16	5.4E-16	0.15	
K00280	lysyl oxidase-like protein 2/3/4 [EC:1.4.3.-]	-0.10	4.9E-16	6.0E-16	-0.10	
K01893	asparaginyl-tRNA synthetase [EC:6.1.1.22]	0.10	4.9E-16	6.0E-16	0.14	
K03587	cell division protein FtsI (penicillin-binding protein 3) [EC:3.4.16.4]	-0.10	6.9E-16	8.5E-16	-0.12	
K00797	spermidine synthase [EC:2.5.1.16]	-0.10	7.3E-16	9.0E-16	-0.19	
K01372	bleomycin hydrolase [EC:3.4.22.40]	-0.10	9.1E-16	1.1E-15	-0.01	
K03183	demethylmenaquinone methyltransferase / 2-methoxy-6-polyprenyl-1,4-benzoquinol methylase [EC:2.1.1.163 2.1.1.201]	0.10	9.2E-16	1.1E-15	0.12	
K03500	16S rRNA (cytosine967-C5)-methyltransferase [EC:2.1.1.176]	-0.10	1.1E-15	1.3E-15	-0.17	
K06169	tRNA 2-(methylsulfanyl)-N6-isopentenyladenosine37 hydroxylase [EC:1.14.99.69]	-0.10	1.2E-15	1.5E-15	-0.20	
K01547	potassium-transporting ATPase ATP-binding subunit [EC:7.2.2.6]	0.10	1.2E-15	1.5E-15	0.15	
K01947	biotin---[acetyl-CoA-carboxylase] ligase / type III pantothenate kinase [EC:6.3.4.15 2.7.1.33]	-0.09	1.5E-15	1.8E-15	-0.09	
K01858	myo-inositol-1-phosphate synthase [EC:5.5.1.4]	-0.09	1.6E-15	2.0E-15	-0.01	
K09650	rhomboid-like protein [EC:3.4.21.105]	-0.09	2.0E-15	2.4E-15	-0.09	
K01284	peptidyl-dipeptidase Dcp [EC:3.4.15.5]	0.09	2.1E-15	2.6E-15	0.21	
K00690	sucrose phosphorylase [EC:2.4.1.7]	-0.09	2.2E-15	2.7E-15	-0.14	
K14466	4-hydroxybutyrate---CoA ligase (AMP-forming) [EC:6.2.1.40]	-0.09	3.1E-15	3.8E-15	-0.10	
K01154	type I restriction enzyme, S subunit [EC:3.1.21.3]	-0.09	4.4E-15	5.4E-15	0.04	
K02110	F-type H+-transporting ATPase subunit c	-0.09	5.4E-15	6.6E-15	-0.16	
K02010	iron(III) transport system ATP-binding protein [EC:7.2.2.7]	-0.09	6.3E-15	7.7E-15	-0.17	

Predictor	Description	Pearson's r	P	FDR-adjusted P	Spearman's rho	Associated with fractures (P<0.05)
K03536	ribonuclease P protein component [EC:3.1.26.5]	-0.09	8.0E-15	9.8E-15	-0.12	yes
K01485	cytosine/creatinine deaminase [EC:3.5.4.1 3.5.4.21]	0.09	8.4E-15	1.0E-14	-0.03	
K00845	glucokinase [EC:2.7.1.2]	-0.09	1.1E-14	1.3E-14	-0.08	
K00549	5-methyltetrahydropteroyltriglutamate--homocysteine methyltransferase [EC:2.1.1.14]	-0.09	1.2E-14	1.5E-14	-0.14	
K03474	pyridoxine 5-phosphate synthase [EC:2.6.99.2]	0.09	1.2E-14	1.5E-14	0.14	
K02756	NA	-0.09	1.9E-14	2.3E-14	-0.11	
K03534	L-rhamnose mutarotase [EC:5.1.3.32]	0.09	2.5E-14	3.0E-14	0.18	
K03054	DNA-directed RNA polymerase subunit I [EC:2.7.7.6]	-0.09	4.0E-14	4.9E-14	-0.10	
K00029	malate dehydrogenase (oxaloacetate-decarboxylating)(NADP+) [EC:1.1.1.40]	0.09	4.2E-14	5.1E-14	0.14	
K14465	succinate semialdehyde reductase (NADPH) [EC:1.1.1.-]	-0.09	5.4E-14	6.6E-14	-0.08	
K01966	propionyl-CoA carboxylase beta chain [EC:6.4.1.3 2.1.3.15]	-0.09	5.9E-14	7.2E-14	-0.08	
K02227	adenosylcobinamide-phosphate synthase [EC:6.3.1.10]	-0.09	6.8E-14	8.3E-14	0.02	
K01878	glycyl-tRNA synthetase alpha chain [EC:6.1.1.14]	0.09	7.5E-14	9.1E-14	0.01	
K00743	N-acetyllactosaminide 3-alpha-galactosyltransferase [EC:2.4.1.87]	-0.09	9.9E-14	1.2E-13	-0.10	
K03366	meso-butanediol dehydrogenase / (S,S)-butanediol dehydrogenase / diacetyl reductase [EC:1.1.1.- 1.1.1.76 1.1.1.304]	-0.09	1.0E-13	1.2E-13	-0.15	
K11927	ATP-dependent RNA helicase RhlE [EC:3.6.4.13]	0.09	1.7E-13	2.1E-13	0.11	
K00211	prephenate dehydrogenase (NADP+) [EC:1.3.1.13]	-0.09	1.7E-13	2.1E-13	-0.11	
K00564	16S rRNA (guanine1207-N2)-methyltransferase [EC:2.1.1.172]	0.09	1.8E-13	2.2E-13	-0.01	
K03841	fructose-1,6-bisphosphatase I [EC:3.1.3.11]	0.09	1.9E-13	2.3E-13	0.02	
K00227	Delta7-sterol 5-desaturase [EC:1.14.19.20]	-0.09	2.0E-13	2.4E-13	-0.10	
K13873	L-arabinose 1-dehydrogenase [EC:1.1.1.376]	-0.09	3.0E-13	3.6E-13	-0.08	
K02377	GDP-L-fucose synthase [EC:1.1.1.271]	0.09	3.4E-13	4.1E-13	0.18	
K03941	NADH dehydrogenase (ubiquinone) Fe-S protein 8 [EC:7.1.1.2]	-0.09	4.5E-13	5.4E-13	-0.09	
K11023	pertussis toxin subunit 1 [EC:2.4.2.-]	0.09	4.6E-13	5.6E-13	0.03	
K14471	succinyl-CoA:(S)-malate CoA-transferase subunit A [EC:2.8.3.22]	-0.09	5.9E-13	7.1E-13	-0.08	
K10775	phenylalanine ammonia-lyase [EC:4.3.1.24]	-0.09	6.0E-13	7.2E-13	-0.14	
K01480	agmatinase [EC:3.5.3.11]	-0.09	6.1E-13	7.4E-13	-0.11	
K00024	malate dehydrogenase [EC:1.1.1.37]	0.09	7.1E-13	8.6E-13	0.22	
K02614	acyl-CoA thioesterase [EC:3.1.2.-]	0.09	7.3E-13	8.8E-13	0.20	
K16593	pimeloyl-[acyl-carrier protein] synthase [EC:1.14.14.46]	-0.09	8.8E-13	1.1E-12	-0.12	
K01760	cysteine-S-conjugate beta-lyase [EC:4.4.1.13]	-0.08	9.5E-13	1.1E-12	-0.14	
K07304	peptide-methionine (S)-S-oxide reductase [EC:1.8.4.11]	0.08	9.6E-13	1.2E-12	0.19	
K02169	malonyl-CoA O-methyltransferase [EC:2.1.1.197]	0.08	1.0E-12	1.2E-12	0.18	
K05606	methylmalonyl-CoA/ethylmalonyl-CoA epimerase [EC:5.1.99.1]	-0.08	1.2E-12	1.4E-12	-0.08	
K01200	pullulanase [EC:3.2.1.41]	-0.08	1.2E-12	1.4E-12	0.07	
K03785	3-dehydroquinate dehydratase I [EC:4.2.1.10]	-0.08	1.3E-12	1.6E-12	-0.14	
K01478	arginine deiminase [EC:3.5.3.6]	-0.08	1.7E-12	2.0E-12	-0.13	
K00189	2-oxoisovalerate/pyruvate ferredoxin oxidoreductase gamma subunit [EC:1.2.7.7 1.2.7.1]	-0.08	1.9E-12	2.3E-12	-0.08	
K02510	4-hydroxy-2-oxoheptanedioate aldolase [EC:4.1.2.52]	0.08	2.2E-12	2.6E-12	-0.07	
K01201	glucosylceramidase [EC:3.2.1.45]	-0.08	2.6E-12	3.1E-12	0.06	
K01190	beta-galactosidase [EC:3.2.1.23]	-0.08	3.1E-12	3.7E-12	0.16	
K00627	pyruvate dehydrogenase E2 component (dihydrolipoamide acetyltransferase) [EC:2.3.1.12]	-0.08	3.3E-12	4.0E-12	-0.14	

Predictor	Description	Pearson's r	P	FDR-adjusted P	Spearman's rho	Associated with fractures (P<0.05)
K06179	23S rRNA pseudouridine955/2504/2580 synthase [EC:5.4.99.24]	-0.08	4.2E-12	5.0E-12	-0.18	
K03773	FKBP-type peptidyl-prolyl cis-trans isomerase FklB [EC:5.2.1.8]	0.08	5.1E-12	6.1E-12	0.21	
K00180	indolepyruvate ferredoxin oxidoreductase, beta subunit [EC:1.2.7.8]	-0.08	5.2E-12	6.2E-12	0.04	
K01879	glycyl-tRNA synthetase beta chain [EC:6.1.1.14]	0.08	5.3E-12	6.3E-12	0.00	
K08838	serine/threonine-protein kinase 24/25/MST4 [EC:2.7.11.1]	-0.08	5.6E-12	6.7E-12	-0.07	
K00791	tRNA dimethylallyltransferase [EC:2.5.1.75]	-0.08	6.4E-12	7.7E-12	0.15	
K01515	ADP-ribose pyrophosphatase [EC:3.6.1.13]	-0.08	7.5E-12	9.0E-12	-0.13	
K00616	transaldolase [EC:2.2.1.2]	0.08	1.1E-11	1.3E-11	0.06	
K01595	phosphoenolpyruvate carboxylase [EC:4.1.1.31]	-0.08	1.1E-11	1.3E-11	-0.13	
K00968	choline-phosphate cytidylyltransferase [EC:2.7.7.15]	-0.08	1.2E-11	1.4E-11	-0.13	
K00691	maltose phosphorylase [EC:2.4.1.8]	-0.08	1.4E-11	1.7E-11	-0.07	
K05349	beta-glucosidase [EC:3.2.1.21]	-0.08	1.6E-11	1.9E-11	0.03	yes
K15863	NADH-quinone oxidoreductase subunit L/M [EC:7.1.1.2]	-0.08	1.6E-11	1.9E-11	-0.08	
K15019	3-hydroxypropionyl-coenzyme A dehydratase [EC:4.2.1.116]	-0.08	1.9E-11	2.3E-11	-0.08	
K03783	purine-nucleoside phosphorylase [EC:2.4.2.1]	-0.08	2.1E-11	2.5E-11	0.05	
K01297	muramoyltetrapeptide carboxypeptidase [EC:3.4.17.13]	0.08	2.6E-11	3.1E-11	0.05	
K01176	alpha-amylase [EC:3.2.1.1]	0.08	2.7E-11	3.2E-11	0.03	
K01735	3-dehydroquinate synthase [EC:4.2.3.4]	-0.08	2.7E-11	3.2E-11	-0.08	
K01092	myo-inositol-1(or 4)-monophosphatase [EC:3.1.3.25]	-0.08	3.6E-11	4.3E-11	0.08	
K03551	holliday junction DNA helicase RuvB [EC:3.6.4.12]	-0.08	3.6E-11	4.3E-11	-0.09	
K02760	cellobiose PTS system EIIB component [EC:2.7.1.196 2.7.1.205]	-0.08	4.0E-11	4.8E-11	-0.16	
K03778	D-lactate dehydrogenase [EC:1.1.1.28]	0.08	4.1E-11	4.9E-11	0.12	
K03365	cytosine/creatinine deaminase [EC:3.5.4.1 3.5.4.21]	-0.08	4.1E-11	4.9E-11	-0.07	
K00957	sulfate adenylyltransferase subunit 2 [EC:2.7.7.4]	0.08	4.2E-11	5.0E-11	0.15	
K01964	acetyl-CoA/propionyl-CoA carboxylase [EC:6.4.1.2 6.4.1.3]	-0.08	4.2E-11	5.0E-11	-0.08	
K11645	fructose-bisphosphate aldolase, class I [EC:4.1.2.13]	0.08	4.3E-11	5.1E-11	0.16	
K15018	3-hydroxypropionyl-coenzyme A synthetase [EC:6.2.1.36]	-0.08	4.9E-11	5.8E-11	-0.07	
K00857	thymidine kinase [EC:2.7.1.21]	0.08	5.3E-11	6.3E-11	0.10	
K02428	XTP/dITP diphosphohydrolase [EC:3.6.1.66]	-0.08	5.6E-11	6.6E-11	0.02	
K03578	ATP-dependent helicase HrpA [EC:3.6.4.13]	-0.08	6.5E-11	7.7E-11	-0.13	
K04567	lysyl-tRNA synthetase, class II [EC:6.1.1.6]	0.08	7.3E-11	8.7E-11	0.04	
K11410	short-chain 2-methylacyl-CoA dehydrogenase [EC:1.3.8.5]	-0.08	9.5E-11	1.1E-10	-0.07	
K00868	pyridoxine kinase [EC:2.7.1.35]	0.08	1.2E-10	1.4E-10	0.08	
K05366	penicillin-binding protein 1A [EC:2.4.1.129 3.4.16.4]	-0.08	1.6E-10	1.9E-10	-0.11	
K01425	glutaminase [EC:3.5.1.2]	0.08	1.9E-10	2.2E-10	0.17	
K01568	pyruvate decarboxylase [EC:4.1.1.1]	-0.08	2.2E-10	2.6E-10	-0.07	
K00654	serine palmitoyltransferase [EC:2.3.1.50]	-0.08	2.2E-10	2.6E-10	-0.07	
K01629	rhamnulose-1-phosphate aldolase [EC:4.1.2.19]	0.08	2.3E-10	2.7E-10	0.16	
K03767	peptidyl-prolyl cis-trans isomerase A (cyclophilin A) [EC:5.2.1.8]	-0.08	2.8E-10	3.3E-10	-0.13	
K04771	serine protease Do [EC:3.4.21.107]	-0.08	2.9E-10	3.4E-10	-0.16	
K01216	licheninase [EC:3.2.1.73]	0.08	2.9E-10	3.4E-10	0.08	
K01854	UDP-galactopyranose mutase [EC:5.4.99.9]	-0.07	3.1E-10	3.7E-10	-0.04	

Predictor	Description	Pearson's r	P	FDR-adjusted P	Spearman's rho	Associated with fractures (P<0.05)
K01512	acylphosphatase [EC:3.6.1.7]	-0.07	3.4E-10	4.0E-10	-0.15	
K08080	CMP-N-acetylneuraminate monooxygenase [EC:1.14.18.2]	-0.07	3.6E-10	4.2E-10	-0.08	yes
K00666	fatty-acyl-CoA synthase [EC:6.2.1.-]	-0.07	3.6E-10	4.2E-10	-0.10	
K01590	histidine decarboxylase [EC:4.1.1.22]	0.07	3.7E-10	4.4E-10	0.07	
K03722	ATP-dependent DNA helicase DinG [EC:3.6.4.12]	-0.07	3.8E-10	4.5E-10	-0.15	
K03564	thioredoxin-dependent peroxiredoxin [EC:1.11.1.24]	0.07	4.5E-10	5.3E-10	0.11	
K03527	4-hydroxy-3-methylbut-2-en-1-yl diphosphate reductase [EC:1.17.7.4]	-0.07	4.8E-10	5.6E-10	-0.12	
K01711	GDPmannose 4,6-dehydratase [EC:4.2.1.47]	0.07	5.0E-10	5.9E-10	0.13	
K02782	glucitol/sorbitol PTS system EIIB component [EC:2.7.1.198]	-0.07	5.9E-10	6.9E-10	-0.07	
K13727	phenolic acid decarboxylase [EC:4.1.1.-]	-0.07	6.1E-10	7.2E-10	-0.16	
K00129	aldehyde dehydrogenase (NAD(P)+) [EC:1.2.1.5]	-0.07	9.2E-10	1.1E-09	-0.08	
K00390	phosphoadenosine phosphosulfate reductase [EC:1.8.4.8 1.8.4.10]	-0.07	9.8E-10	1.2E-09	-0.10	
K13060	acyl homoserine lactone synthase [EC:2.3.1.184]	0.07	9.9E-10	1.2E-09	-0.04	
K03790	[ribosomal protein S5]-alanine N-acetyltransferase [EC:2.3.1.267]	-0.07	1.4E-09	1.6E-09	-0.15	
K06120	glycerol dehydratase large subunit [EC:4.2.1.30]	-0.07	1.5E-09	1.8E-09	-0.18	
K01770	2-C-methyl-D-erythritol 2,4-cyclodiphosphate synthase [EC:4.6.1.12]	-0.07	1.7E-09	2.0E-09	-0.08	yes
K01051	pectinesterase [EC:3.1.1.11]	0.07	1.9E-09	2.2E-09	0.17	
K01610	phosphoenolpyruvate carboxykinase (ATP) [EC:4.1.1.49]	0.07	2.3E-09	2.7E-09	0.17	
K08599	YopT peptidase [EC:3.4.22.-]	-0.07	2.7E-09	3.2E-09	-0.12	
K00926	carbamate kinase [EC:2.7.2.2]	0.07	2.8E-09	3.3E-09	-0.05	
K00854	xylulokinase [EC:2.7.1.17]	-0.07	3.3E-09	3.9E-09	-0.03	
K00575	chemotaxis protein methyltransferase CheR [EC:2.1.1.80]	-0.07	3.6E-09	4.2E-09	-0.17	
K00647	3-oxoacyl-[acyl-carrier-protein] synthase I [EC:2.3.1.41]	0.07	3.7E-09	4.3E-09	0.18	
K00773	queuine tRNA-ribosyltransferase [EC:2.4.2.29]	-0.07	3.8E-09	4.4E-09	-0.11	
K03654	ATP-dependent DNA helicase RecQ [EC:3.6.4.12]	0.07	3.9E-09	4.6E-09	0.20	
K00275	pyridoxamine 5'-phosphate oxidase [EC:1.4.3.5]	0.07	5.6E-09	6.5E-09	0.03	
K09483	3-dehydroshikimate dehydratase [EC:4.2.1.118]	-0.07	5.8E-09	6.8E-09	-0.07	
K00067	dTDP-4-dehydrorhamnose reductase [EC:1.1.1.133]	0.07	6.1E-09	7.1E-09	0.20	
K11948	1-hydroxy-2-naphthoate dioxygenase [EC:1.13.11.38]	-0.07	6.3E-09	7.3E-09	-0.06	
K11947	aldehyde dehydrogenase [EC:1.2.1.-]	-0.07	6.3E-09	7.3E-09	-0.06	
K11946	hydratase-aldolase [EC:4.1.2.-]	-0.07	6.3E-09	7.3E-09	-0.06	
K11943	PAH dioxygenase large subunit [EC:1.13.11.-]	-0.07	6.3E-09	7.3E-09	-0.06	
K01813	L-rhamnose isomerase [EC:5.3.1.14]	0.07	6.7E-09	7.8E-09	0.16	
K00761	uracil phosphoribosyltransferase [EC:2.4.2.9]	-0.07	7.8E-09	9.1E-09	0.06	yes
K02852	UDP-N-acetyl-D-mannosaminouronate:lipid I N-acetyl-D-mannosaminouronosyltransferase [EC:2.4.1.180]	0.07	8.4E-09	9.8E-09	0.18	
K00768	nicotinate-nucleotide--dimethylbenzimidazole phosphoribosyltransferase [EC:2.4.2.21]	0.07	8.8E-09	1.0E-08	0.11	
K00077	2-dehydropantoate 2-reductase [EC:1.1.1.169]	0.07	8.8E-09	1.0E-08	0.12	
K02017	molybdate transport system ATP-binding protein [EC:7.3.2.5]	0.07	9.7E-09	1.1E-08	-0.05	
K00210	NA	0.07	9.8E-09	1.1E-08	0.07	
K03430	2-aminoethylphosphonate-pyruvate transaminase [EC:2.6.1.37]	0.07	1.0E-08	1.2E-08	0.22	
K11944	PAH dioxygenase small subunit [EC:1.13.11.-]	-0.07	1.1E-08	1.3E-08	-0.06	
K01470	creatinine amidohydrolase [EC:3.5.2.10]	0.07	1.2E-08	1.4E-08	0.22	

Predictor	Description	Pearson's r	P	FDR-adjusted P	Spearman's rho	Associated with fractures (P<0.05)
K01034	acetate CoA/acetoacetate CoA-transferase alpha subunit [EC:2.8.3.8 2.8.3.9]	0.07	1.2E-08	1.4E-08	0.01	
K01667	tryptophanase [EC:4.1.99.1]	-0.07	1.4E-08	1.6E-08	-0.10	
K01771	1-phosphatidylinositol phosphodiesterase [EC:4.6.1.13]	0.07	1.5E-08	1.7E-08	0.07	
K01686	mannonate dehydratase [EC:4.2.1.8]	0.07	1.6E-08	1.9E-08	0.15	
K02112	F-type H+/Na+-transporting ATPase subunit beta [EC:7.1.2.2 7.2.2.1]	-0.07	1.8E-08	2.1E-08	-0.11	
K00254	dihydroorotate dehydrogenase [EC:1.3.5.2]	0.07	1.9E-08	2.2E-08	0.03	
K01619	deoxyribose-phosphate aldolase [EC:4.1.2.4]	0.07	1.9E-08	2.2E-08	0.06	
K07248	lactaldehyde dehydrogenase / glycolaldehyde dehydrogenase [EC:1.2.1.22 1.2.1.21]	0.07	2.1E-08	2.4E-08	0.18	
K00335	NADH-quinone oxidoreductase subunit F [EC:7.1.1.2]	-0.07	2.1E-08	2.4E-08	-0.13	
K01666	4-hydroxy 2-oxovalerate aldolase [EC:4.1.3.39]	0.07	2.4E-08	2.8E-08	0.18	
K01627	2-dehydro-3-deoxyphosphooctonate aldolase (KDO 8-P synthase) [EC:2.5.1.55]	0.07	2.5E-08	2.9E-08	0.08	
K00336	NADH-quinone oxidoreductase subunit G [EC:7.1.1.2]	0.07	3.0E-08	3.5E-08	-0.05	
K11945	extradiol dioxygenase [EC:1.13.11.-]	-0.07	3.2E-08	3.7E-08	-0.06	
K08316	16S rRNA (guanine966-N2)-methyltransferase [EC:2.1.1.171]	0.07	3.6E-08	4.2E-08	-0.03	
K02484	two-component system, OmpR family, sensor kinase [EC:2.7.13.3]	0.07	4.2E-08	4.8E-08	0.11	
K00334	NADH-quinone oxidoreductase subunit E [EC:7.1.1.2]	-0.06	5.5E-08	6.3E-08	-0.13	
K03685	ribonuclease III [EC:3.1.26.3]	-0.06	7.0E-08	8.1E-08	-0.11	
K01256	aminopeptidase N [EC:3.4.11.2]	-0.06	7.2E-08	8.3E-08	-0.11	
K01953	asparagine synthase (glutamine-hydrolysing) [EC:6.3.5.4]	-0.06	7.3E-08	8.4E-08	-0.06	
K00912	tetraacyldisaccharide 4'-kinase [EC:2.7.1.130]	0.06	7.4E-08	8.5E-08	0.07	
K01712	urocanate hydratase [EC:4.2.1.49]	0.06	7.8E-08	9.0E-08	0.18	
K03427	type I restriction enzyme M protein [EC:2.1.1.72]	-0.06	9.3E-08	1.1E-07	0.09	
K00766	anthranilate phosphoribosyltransferase [EC:2.4.2.18]	-0.06	1.0E-07	1.2E-07	0.06	
K00658	2-oxoglutarate dehydrogenase E2 component (dihydrolipoamide succinyltransferase) [EC:2.3.1.61]	0.06	1.1E-07	1.3E-07	0.03	
K01783	ribulose-phosphate 3-epimerase [EC:5.1.3.1]	-0.06	1.1E-07	1.3E-07	-0.09	
K00784	ribonuclease Z [EC:3.1.26.11]	0.06	1.3E-07	1.5E-07	0.10	
K01468	imidazolonepropionase [EC:3.5.2.7]	0.06	1.3E-07	1.5E-07	0.18	
K02120	V/A-type H+/Na+-transporting ATPase subunit D	-0.06	1.4E-07	1.6E-07	0.08	
K00104	glycolate oxidase [EC:1.1.3.15]	-0.06	1.6E-07	1.8E-07	-0.11	
K01938	formate--tetrahydrofolate ligase [EC:6.3.4.3]	-0.06	1.6E-07	1.8E-07	0.05	
K14467	4-hydroxybutyrate---CoA ligase (AMP-forming) [EC:6.2.1.40]	-0.06	2.8E-07	3.2E-07	-0.06	
K00520	mercuric reductase [EC:1.16.1.1]	-0.06	2.8E-07	3.2E-07	-0.17	
K00983	N-acylneuraminate cytidylyltransferase [EC:2.7.7.43]	0.06	3.4E-07	3.9E-07	0.17	
K00874	2-dehydro-3-deoxygluconokinase [EC:2.7.1.45]	0.06	3.4E-07	3.9E-07	0.14	
K07173	S-ribosylhomocysteine lyase [EC:4.4.1.21]	-0.06	3.8E-07	4.4E-07	-0.09	
K02755	beta-glucoside PTS system EIIA component [EC:2.7.1.-]	-0.06	3.8E-07	4.4E-07	-0.06	
K12248	beta-galactoside alpha-2,6-sialyltransferase (sialyltransferase 0160) [EC:2.4.99.1]	-0.06	4.4E-07	5.0E-07	-0.07	
K00450	gentisate 1,2-dioxygenase [EC:1.13.11.4]	-0.06	4.4E-07	5.0E-07	-0.16	
K01129	dGTPase [EC:3.1.5.1]	-0.06	4.6E-07	5.3E-07	-0.10	
K00785	N-acetyllactosaminide alpha-2,3-sialyltransferase [EC:2.4.99.6]	-0.06	4.6E-07	5.3E-07	-0.13	
K00788	thiamine-phosphate pyrophosphorylase [EC:2.5.1.3]	0.06	5.0E-07	5.7E-07	0.18	
K01685	altronate hydrolase [EC:4.2.1.7]	0.06	5.6E-07	6.4E-07	0.15	

Predictor	Description	Pearson's r	P	FDR-adjusted P	Spearman's rho	Associated with fractures (P<0.05)
K01153	type I restriction enzyme, R subunit [EC:3.1.21.3]	-0.06	6.0E-07	6.9E-07	0.08	
K01450	NA	-0.06	6.7E-07	7.6E-07	-0.06	
K03644	lipoyl synthase [EC:2.8.1.8]	0.06	8.0E-07	9.1E-07	0.08	
K01775	alanine racemase [EC:5.1.1.1]	-0.06	8.4E-07	9.6E-07	-0.14	
K01054	acylglycerol lipase [EC:3.1.1.23]	-0.06	9.0E-07	1.0E-06	-0.05	
K00986	RNA-directed DNA polymerase [EC:2.7.7.49]	-0.06	1.0E-06	1.1E-06	0.12	
K03386	peroxiredoxin 2/4 [EC:1.11.1.24]	0.06	1.1E-06	1.3E-06	0.05	
K06989	aspartate dehydrogenase [EC:1.4.1.21]	-0.06	1.2E-06	1.4E-06	0.10	
K09882	cobaltochelatase CobS [EC:6.6.1.2]	-0.06	1.2E-06	1.4E-06	-0.18	
K01192	beta-mannosidase [EC:3.2.1.25]	0.06	1.3E-06	1.5E-06	0.22	
K02013	iron complex transport system ATP-binding protein [EC:7.2.2.-]	-0.06	1.6E-06	1.8E-06	-0.08	
K00176	2-oxoglutarate ferredoxin oxidoreductase subunit delta [EC:1.2.7.3]	-0.06	1.6E-06	1.8E-06	0.10	
K03770	peptidyl-prolyl cis-trans isomerase D [EC:5.2.1.8]	0.06	1.7E-06	1.9E-06	0.08	
K01613	phosphatidylserine decarboxylase [EC:4.1.1.65]	0.06	1.7E-06	1.9E-06	0.05	
K01585	arginine decarboxylase [EC:4.1.1.19]	0.06	1.9E-06	2.2E-06	0.08	
K13246	c-di-GMP phosphodiesterase [EC:3.1.4.52]	-0.06	2.0E-06	2.3E-06	-0.06	
K09457	7-cyano-7-deazaguanine reductase [EC:1.7.1.13]	0.06	2.0E-06	2.3E-06	0.22	
K15532	unsaturated rhamnogalacturonyl hydrolase [EC:3.2.1.172]	-0.06	2.1E-06	2.4E-06	0.12	
K03148	sulfur carrier protein ThiS adenylyltransferase [EC:2.7.7.73]	-0.06	2.2E-06	2.5E-06	-0.11	
K00325	H+-translocating NAD(P) transhydrogenase subunit beta [EC:1.6.1.2 7.1.1.1]	-0.06	2.3E-06	2.6E-06	-0.11	
K00330	NADH-quinone oxidoreductase subunit A [EC:7.1.1.2]	0.06	2.3E-06	2.6E-06	0.08	
K07408	cytochrome P450 family 1 subfamily A1 [EC:1.14.14.1]	-0.06	2.8E-06	3.2E-06	-0.09	
K00485	dimethylaniline monooxygenase (N-oxide forming) [EC:1.14.13.8]	-0.06	2.8E-06	3.2E-06	-0.09	
K00331	NADH-quinone oxidoreductase subunit B [EC:7.1.1.2]	0.06	3.1E-06	3.5E-06	0.07	
K00156	pyruvate dehydrogenase (quinone) [EC:1.2.5.1]	0.06	3.1E-06	3.5E-06	0.02	
K00763	nicotinate phosphoribosyltransferase [EC:6.3.4.21]	-0.06	3.2E-06	3.6E-06	-0.11	
K00001	alcohol dehydrogenase [EC:1.1.1.1]	-0.06	3.2E-06	3.6E-06	0.09	
K14189	uncharacterized oxidoreductase [EC:1.-.-.-]	-0.06	3.4E-06	3.8E-06	-0.15	
K04565	superoxide dismutase, Cu-Zn family [EC:1.15.1.1]	-0.06	3.4E-06	3.8E-06	-0.12	
K01270	dipeptidase D [EC:3.4.13.-]	0.06	3.5E-06	4.0E-06	0.14	
K14665	amidohydrolase [EC:3.5.1.-]	0.06	3.6E-06	4.1E-06	-0.05	
K01754	threonine dehydratase [EC:4.3.1.19]	-0.06	3.7E-06	4.2E-06	-0.11	
K02558	UDP-N-acetylmuramate: L-alanyl-gamma-D-glutamyl-meso-diaminopimelate ligase [EC:6.3.2.45]	-0.05	4.0E-06	4.5E-06	-0.11	
K00093	methanol dehydrogenase [EC:1.1.1.244]	-0.05	4.0E-06	4.5E-06	-0.05	
K00677	UDP-N-acetylglucosamine acyltransferase [EC:2.3.1.129]	0.05	4.5E-06	5.1E-06	0.07	
K06122	glycerol dehydratase small subunit [EC:4.2.1.30]	-0.05	4.5E-06	5.1E-06	-0.15	
K03818	putative colanic acid biosynthesis acetyltransferase WcaF [EC:2.3.1.-]	0.05	4.6E-06	5.2E-06	0.14	
K01919	glutamate--cysteine ligase [EC:6.3.2.2]	0.05	4.7E-06	5.3E-06	-0.03	
K13821	RHH-type transcriptional regulator, proline utilization regulon repressor / proline dehydrogenase / delta 1-pyrroline-5-carboxylate dehydrogenase [EC:1.5.5.2 1.2.1.88]	0.05	5.4E-06	6.1E-06	0.14	
K00945	CMP/dCMP kinase [EC:2.7.4.25]	-0.05	5.8E-06	6.5E-06	-0.05	
K00341	NADH-quinone oxidoreductase subunit L [EC:7.1.1.2]	0.05	6.9E-06	7.8E-06	0.07	
K00339	NADH-quinone oxidoreductase subunit J [EC:7.1.1.2]	0.05	7.7E-06	8.7E-06	0.07	

Predictor	Description	Pearson's r	P	FDR-adjusted P	Spearman's rho	Associated with fractures (P<0.05)
K06269	serine/threonine-protein phosphatase PP1 catalytic subunit [EC:3.1.3.16]	-0.05	7.9E-06	8.9E-06	-0.06	
K01066	acetyl esterase [EC:3.1.1.-]	-0.05	8.1E-06	9.1E-06	-0.13	
K01807	ribose 5-phosphate isomerase A [EC:5.3.1.6]	-0.05	8.3E-06	9.3E-06	-0.09	
K03801	lipoyl(octanoyl) transferase [EC:2.3.1.181]	-0.05	8.5E-06	9.5E-06	-0.07	
K02230	cobaltochelatase CobN [EC:6.6.1.2]	0.05	9.0E-06	1.0E-05	0.21	
K00337	NADH-quinone oxidoreductase subunit H [EC:7.1.1.2]	0.05	9.2E-06	1.0E-05	0.07	
K11991	tRNA(adenine34) deaminase [EC:3.5.4.33]	-0.05	9.9E-06	1.1E-05	0.03	
K13788	phosphate acetyltransferase [EC:2.3.1.8]	-0.05	1.0E-05	1.1E-05	-0.11	
K00684	leucyl/phenylalanyl-tRNA---protein transferase [EC:2.3.2.6]	0.05	1.0E-05	1.1E-05	0.14	
K00990	[protein-PII] uridylyltransferase [EC:2.7.7.59]	-0.05	1.0E-05	1.1E-05	-0.11	
K14459	hexosaminidase [EC:3.2.1.52]	-0.05	1.1E-05	1.2E-05	-0.05	
K01745	histidine ammonia-lyase [EC:4.3.1.3]	0.05	1.1E-05	1.2E-05	0.14	
K09758	aspartate 4-decarboxylase [EC:4.1.1.12]	0.05	1.2E-05	1.3E-05	0.22	
K04041	fructose-1,6-bisphosphatase III [EC:3.1.3.11]	0.05	1.3E-05	1.5E-05	0.21	
K01823	isopentenyl-diphosphate Delta-isomerase [EC:5.3.3.2]	0.05	1.3E-05	1.5E-05	-0.04	
K02233	adenosylcobinamide-GDP ribazoletransferase [EC:2.7.8.26]	0.05	1.3E-05	1.5E-05	0.08	
K15241	tetrachlorohydroquinone reductive dehalogenase [EC:1.21.4.5]	-0.05	1.4E-05	1.6E-05	-0.05	
K01695	tryptophan synthase alpha chain [EC:4.2.1.20]	-0.05	1.5E-05	1.7E-05	-0.11	
K13934	phosphoribosyl-dephospho-CoA transferase [EC:2.7.7.66]	-0.05	1.5E-05	1.7E-05	-0.16	
K15036	acetyl-CoA/propionyl-CoA carboxylase [EC:6.4.1.2 6.4.1.3 2.1.3.15]	-0.05	1.5E-05	1.7E-05	-0.05	
K00882	1-phosphofructokinase [EC:2.7.1.56]	-0.05	1.5E-05	1.7E-05	-0.13	
K00683	glutaminyl-peptide cyclotransferase [EC:2.3.2.5]	0.05	1.6E-05	1.8E-05	-0.02	
K15017	malonyl-CoA/succinyl-CoA reductase (NADPH) [EC:1.2.1.75 1.2.1.76]	-0.05	1.7E-05	1.9E-05	-0.05	
K12251	N-carbamoylputrescine amidase [EC:3.5.1.53]	-0.05	1.9E-05	2.1E-05	0.10	
K01464	dihydropyrimidinase [EC:3.5.2.2]	0.05	2.1E-05	2.3E-05	-0.07	
K11628	mycocerosic acid synthase [EC:2.3.1.111]	-0.05	2.1E-05	2.3E-05	-0.05	
K12445	trans enoyl reductase [EC:1.3.1.-]	-0.05	2.1E-05	2.3E-05	-0.05	
K07682	two-component system, NarL family, sensor histidine kinase DevS [EC:2.7.13.3]	-0.05	2.1E-05	2.3E-05	-0.05	
K00574	cyclopropane-fatty-acyl-phospholipid synthase [EC:2.1.1.79]	0.05	2.2E-05	2.4E-05	-0.03	
K10805	acyl-CoA thioesterase II [EC:3.1.2.-]	-0.05	2.2E-05	2.4E-05	-0.11	
K00603	glutamate formiminotransferase / 5-formyltetrahydrofolate cyclo-ligase [EC:2.1.2.5 6.3.3.2]	0.05	2.4E-05	2.7E-05	0.23	
K00793	riboflavin synthase [EC:2.5.1.9]	-0.05	2.4E-05	2.7E-05	-0.08	
K01912	phenylacetate-CoA ligase [EC:6.2.1.30]	0.05	2.5E-05	2.8E-05	0.18	
K00177	2-oxoglutarate ferredoxin oxidoreductase subunit gamma [EC:1.2.7.3]	-0.05	2.6E-05	2.9E-05	0.11	
K13040	two-component system, LuxR family, sensor histidine kinase TtrS [EC:2.7.13.3]	0.05	2.6E-05	2.9E-05	-0.05	
K00701	cyclomaltodextrin glucanotransferase [EC:2.4.1.19]	-0.05	2.7E-05	3.0E-05	-0.04	
K00605	aminomethyltransferase [EC:2.1.2.10]	0.05	2.7E-05	3.0E-05	0.07	
K01589	5-(carboxyamino)imidazole ribonucleotide synthase [EC:6.3.4.18]	-0.05	3.0E-05	3.3E-05	-0.07	
K05520	protease I [EC:3.5.1.124]	0.05	3.1E-05	3.4E-05	-0.01	
K05989	alpha-L-rhamnosidase [EC:3.2.1.40]	0.05	3.5E-05	3.9E-05	0.20	
K01130	arylsulfatase [EC:3.1.6.1]	0.05	3.7E-05	4.1E-05	0.02	
K00999	CDP-diacylglycerol--inositol 3-phosphatidyltransferase [EC:2.7.8.11]	-0.05	4.3E-05	4.8E-05	-0.04	

Predictor	Description	Pearson's r	P	FDR-adjusted P	Spearman's rho	Associated with fractures (P<0.05)
K00602	phosphoribosylaminoimidazolecarboxamide formyltransferase / IMP cyclohydrolase [EC:2.1.2.3 3.5.4.10]	-0.05	5.0E-05	5.5E-05	-0.06	
K00027	malate dehydrogenase (oxaloacetate-decarboxylating) [EC:1.1.1.38]	0.05	5.1E-05	5.6E-05	-0.05	
K04518	prephenate dehydratase [EC:4.2.1.51]	-0.05	5.2E-05	5.8E-05	0.11	
K07107	acyl-CoA thioester hydrolase [EC:3.1.2.-]	0.05	5.3E-05	5.9E-05	0.16	
K01573	oxaloacetate decarboxylase (Na+ extruding) subunit gamma	0.05	6.2E-05	6.9E-05	0.22	
K01492	phosphoribosylglycinamide/phosphoribosylaminoimidazolecarboxamide formyltransferase [EC:2.1.2.2 2.1.2.3]	0.05	6.6E-05	7.3E-05	0.22	
K00567	methylated-DNA-[protein]-cysteine S-methyltransferase [EC:2.1.1.63]	0.05	6.9E-05	7.6E-05	0.06	
K00286	pyrroline-5-carboxylate reductase [EC:1.5.1.2]	-0.05	6.9E-05	7.6E-05	-0.04	
K01144	NA	0.05	7.5E-05	8.3E-05	0.22	
K01679	fumarate hydratase, class II [EC:4.2.1.2]	0.05	7.5E-05	8.3E-05	-0.05	
K10708	fructoselysine 6-phosphate deglycase [EC:3.5.-.-]	-0.05	8.3E-05	9.2E-05	-0.15	
K01548	potassium-transporting ATPase KdpC subunit	0.05	9.4E-05	1.0E-04	0.06	
K00963	UTP--glucose-1-phosphate uridylyltransferase [EC:2.7.7.9]	-0.05	9.5E-05	1.0E-04	-0.11	
K01808	ribose 5-phosphate isomerase B [EC:5.3.1.6]	0.05	9.6E-05	1.1E-04	0.04	
K03332	fructan beta-fructosidase [EC:3.2.1.80]	0.05	1.0E-04	1.1E-04	0.05	
K05306	phosphonoacetaldehyde hydrolase [EC:3.11.1.1]	0.05	1.0E-04	1.1E-04	0.21	
K11949	4-(2-carboxyphenyl)-2-oxobut-3-enoate aldolase [EC:4.1.2.34]	-0.05	1.1E-04	1.2E-04	-0.04	
K01657	anthranilate synthase component I [EC:4.1.3.27]	-0.05	1.1E-04	1.2E-04	-0.11	
K02342	DNA polymerase III subunit epsilon [EC:2.7.7.7]	-0.05	1.1E-04	1.2E-04	0.06	
K00183	NA	-0.05	1.3E-04	1.4E-04	-0.07	
K02343	DNA polymerase III subunit gamma/tau [EC:2.7.7.7]	-0.05	1.4E-04	1.5E-04	-0.11	
K00812	aspartate aminotransferase [EC:2.6.1.1]	-0.04	1.7E-04	1.9E-04	0.17	
K01007	pyruvate, water dikinase [EC:2.7.9.2]	0.04	1.9E-04	2.1E-04	-0.08	
K01520	dUTP pyrophosphatase [EC:3.6.1.23]	0.04	2.0E-04	2.2E-04	0.09	
K01447	N-acetylmuramoyl-L-alanine amidase [EC:3.5.1.28]	-0.04	2.0E-04	2.2E-04	0.06	
K01079	phosphoserine phosphatase [EC:3.1.3.3]	0.04	2.1E-04	2.3E-04	0.08	
K10026	7-carboxy-7-deazaguanine synthase [EC:4.3.99.3]	0.04	2.2E-04	2.4E-04	-0.02	
K00231	protoporphyrinogen/coproporphyrinogen III oxidase [EC:1.3.3.4 1.3.3.15]	0.04	2.2E-04	2.4E-04	0.03	
K13001	mannosyltransferase [EC:2.4.1.-]	0.04	2.3E-04	2.5E-04	-0.03	
K00556	tRNA (guanosine-2'-O-)-methyltransferase [EC:2.1.1.34]	0.04	2.5E-04	2.7E-04	0.02	
K00639	glycine C-acetyltransferase [EC:2.3.1.29]	0.04	2.7E-04	3.0E-04	0.13	
K00615	transketolase [EC:2.2.1.1]	-0.04	2.7E-04	3.0E-04	0.03	
K01838	beta-phosphoglucomutase [EC:5.4.2.6]	-0.04	2.8E-04	3.1E-04	-0.08	
K15853	acyl transferase [EC:2.3.1.-]	0.04	2.9E-04	3.2E-04	-0.01	
K00895	diphosphate-dependent phosphofructokinase [EC:2.7.1.90]	0.04	3.0E-04	3.3E-04	0.21	
K01925	UDP-N-acetylmuramoylalanine--D-glutamate ligase [EC:6.3.2.9]	-0.04	3.1E-04	3.4E-04	-0.06	
K00028	malate dehydrogenase (decarboxylating) [EC:1.1.1.39]	-0.04	3.4E-04	3.7E-04	-0.05	
K00223	Delta24(24(1))-sterol reductase [EC:1.3.1.71]	-0.04	3.8E-04	4.1E-04	-0.04	
K00853	L-ribulokinase [EC:2.7.1.16]	0.04	3.9E-04	4.3E-04	0.01	
K01839	phosphopentomutase [EC:5.4.2.7]	-0.04	4.0E-04	4.4E-04	-0.13	
K01804	L-arabinose isomerase [EC:5.3.1.4]	-0.04	4.5E-04	4.9E-04	-0.04	
K07404	6-phosphogluconolactonase [EC:3.1.1.31]	-0.04	4.6E-04	5.0E-04	-0.13	

Predictor	Description	Pearson's r	P	FDR-adjusted P	Spearman's rho	Associated with fractures (P<0.05)
K01835	phosphoglucomutase [EC:5.4.2.2]	-0.04	5.0E-04	5.4E-04	-0.05	
K00626	acetyl-CoA C-acetyltransferase [EC:2.3.1.9]	-0.04	5.2E-04	5.7E-04	-0.14	
K12904	phosphonoacetaldehyde reductase [EC:1.1.1.309]	-0.04	5.3E-04	5.8E-04	-0.04	
K08303	U32 family peptidase [EC:3.4.-.-]	-0.04	5.7E-04	6.2E-04	-0.09	
K03781	catalase [EC:1.11.1.6]	-0.04	5.8E-04	6.3E-04	-0.07	
K02182	carnitine-CoA ligase [EC:6.2.1.48]	-0.04	5.9E-04	6.4E-04	-0.13	
K12339	S-sulfo-L-cysteine synthase (O-acetyl-L-serine-dependent) [EC:2.5.1.144]	0.04	6.0E-04	6.5E-04	0.00	
K01698	porphobilinogen synthase [EC:4.2.1.24]	0.04	6.3E-04	6.8E-04	-0.08	
K00266	glutamate synthase (NADPH) small chain [EC:1.4.1.13]	-0.04	6.5E-04	7.1E-04	0.05	yes
K05603	formimidoylglutamate deiminase [EC:3.5.3.13]	-0.04	6.5E-04	7.1E-04	-0.15	
K02056	simple sugar transport system ATP-binding protein [EC:7.5.2.-]	0.04	6.6E-04	7.2E-04	-0.08	
K02217	ferritin [EC:1.16.3.2]	0.04	6.9E-04	7.5E-04	0.13	
K13929	malonate decarboxylase alpha subunit [EC:2.3.1.187]	-0.04	7.1E-04	7.7E-04	-0.15	
K02564	glucosamine-6-phosphate deaminase [EC:3.5.99.6]	-0.04	7.3E-04	7.9E-04	0.05	
K03579	ATP-dependent helicase HrpB [EC:3.6.4.13]	-0.04	7.7E-04	8.3E-04	-0.09	
K01057	6-phosphogluconolactonase [EC:3.1.1.31]	0.04	7.7E-04	8.3E-04	0.20	
K05995	dipeptidase E [EC:3.4.13.21]	0.04	7.7E-04	8.3E-04	0.12	
K00606	3-methyl-2-oxobutanoate hydroxymethyltransferase [EC:2.1.2.11]	0.04	8.8E-04	9.5E-04	0.09	
K01389	neprilysin [EC:3.4.24.11]	-0.04	8.9E-04	9.6E-04	-0.03	
K15633	2,3-bisphosphoglycerate-independent phosphoglycerate mutase [EC:5.4.2.12]	-0.04	9.3E-04	1.0E-03	-0.09	
K04516	chorismate mutase [EC:5.4.99.5]	-0.04	1.0E-03	1.1E-03	0.13	
K01443	N-acetylglucosamine-6-phosphate deacetylase [EC:3.5.1.25]	-0.04	1.0E-03	1.1E-03	-0.05	
K04103	indolepyruvate decarboxylase [EC:4.1.1.74]	-0.04	1.0E-03	1.1E-03	-0.14	
K02794	mannose PTS system EIIAB component [EC:2.7.1.191]	-0.04	1.0E-03	1.1E-03	-0.12	
K01023	arylsulfate sulfotransferase [EC:2.8.2.22]	0.04	1.1E-03	1.2E-03	0.16	
K11381	2-oxoisovalerate dehydrogenase E1 component [EC:1.2.4.4]	0.04	1.4E-03	1.5E-03	0.03	
K02121	V/A-type H+/Na+-transporting ATPase subunit E	-0.04	1.4E-03	1.5E-03	0.13	
K01886	glutaminyl-tRNA synthetase [EC:6.1.1.18]	0.04	1.5E-03	1.6E-03	0.05	
K06131	cardiolipin synthase A/B [EC:2.7.8.-]	0.04	1.7E-03	1.8E-03	0.02	
K06949	ribosome biogenesis GTPase / thiamine phosphate phosphatase [EC:3.6.1.- 3.1.3.100]	0.04	1.7E-03	1.8E-03	0.05	
K01280	tripeptidyl-peptidase II [EC:3.4.14.10]	-0.04	1.7E-03	1.8E-03	-0.03	
K03817	ribosomal-protein-serine acetyltransferase [EC:2.3.1.-]	-0.04	1.8E-03	1.9E-03	-0.05	
K13935	malonate decarboxylase epsilon subunit [EC:2.3.1.39]	0.04	1.9E-03	2.0E-03	-0.07	
K02806	nitrogen PTS system EIIA component [EC:2.7.1.-]	-0.04	1.9E-03	2.0E-03	-0.09	
K02117	V/A-type H+/Na+-transporting ATPase subunit A [EC:7.1.2.2 7.2.2.1]	-0.04	2.0E-03	2.2E-03	0.13	
K08093	3-hexulose-6-phosphate synthase [EC:4.1.2.43]	0.04	2.1E-03	2.3E-03	-0.06	
K09474	acid phosphatase (class A) [EC:3.1.3.2]	0.04	2.3E-03	2.5E-03	0.16	
K01082	3'(2'), 5'-bisphosphate nucleotidase [EC:3.1.3.7]	0.04	2.6E-03	2.8E-03	0.02	
K03826	putative acetyltransferase [EC:2.3.1.-]	-0.04	2.7E-03	2.9E-03	-0.09	
K01592	tyrosine decarboxylase [EC:4.1.1.25]	-0.04	2.9E-03	3.1E-03	-0.03	
K15373	sulfoacetaldehyde reductase [EC:1.1.1.313]	0.04	3.0E-03	3.2E-03	-0.04	
K00648	3-oxoacyl-[acyl-carrier-protein] synthase III [EC:2.3.1.180]	0.04	3.2E-03	3.4E-03	0.01	

Predictor	Description	Pearson's r	P	FDR-adjusted P	Spearman's rho	Associated with fractures (P<0.05)
K12373	hexosaminidase [EC:3.2.1.52]	-0.04	3.3E-03	3.5E-03	0.16	
K01628	L-fuculose-phosphate aldolase [EC:4.1.2.17]	0.03	3.5E-03	3.8E-03	-0.10	
K03168	DNA topoisomerase I [EC:5.6.2.1]	-0.03	3.6E-03	3.9E-03	-0.03	
K07405	alpha-amylase [EC:3.2.1.1]	-0.03	3.6E-03	3.9E-03	0.13	
K00819	ornithine--oxo-acid transaminase [EC:2.6.1.13]	-0.03	3.6E-03	3.9E-03	0.13	
K04068	anaerobic ribonucleoside-triphosphate reductase activating protein [EC:1.97.1.4]	-0.03	3.7E-03	4.0E-03	-0.04	
K00033	6-phosphogluconate dehydrogenase [EC:1.1.1.44 1.1.1.343]	0.03	4.2E-03	4.5E-03	0.04	
K00259	alanine dehydrogenase [EC:1.4.1.1]	-0.03	4.3E-03	4.6E-03	0.13	
K00951	GTP pyrophosphokinase [EC:2.7.6.5]	0.03	4.6E-03	4.9E-03	0.14	
K09011	(R)-citramalate synthase [EC:2.3.1.182]	0.03	4.9E-03	5.2E-03	0.23	
K07141	molybdenum cofactor cytidylyltransferase [EC:2.7.7.76]	0.03	4.9E-03	5.2E-03	-0.09	
K01802	peptidylprolyl isomerase [EC:5.2.1.8]	-0.03	4.9E-03	5.2E-03	0.07	
K01181	endo-1,4-beta-xylanase [EC:3.2.1.8]	-0.03	5.0E-03	5.3E-03	0.13	
K01338	ATP-dependent Lon protease [EC:3.4.21.53]	0.03	5.3E-03	5.7E-03	0.02	
K00244	fumarate reductase flavoprotein subunit [EC:1.3.5.4]	0.03	5.8E-03	6.2E-03	-0.02	
K08678	UDP-glucuronate decarboxylase [EC:4.1.1.35]	-0.03	6.1E-03	6.5E-03	0.13	
K01811	alpha-D-xyloside xylohydrolase [EC:3.2.1.177]	0.03	6.2E-03	6.6E-03	0.16	
K01916	NAD+ synthase [EC:6.3.1.5]	0.03	6.5E-03	6.9E-03	-0.06	
K00844	hexokinase [EC:2.7.1.1]	0.03	6.6E-03	7.0E-03	0.04	
K02549	o-succinylbenzoate synthase [EC:4.2.1.113]	-0.03	7.2E-03	7.7E-03	-0.09	
K02346	DNA polymerase IV [EC:2.7.7.7]	-0.03	8.0E-03	8.5E-03	-0.01	
K11752	diaminohydroxyphosphoribosylaminopyrimidine deaminase / 5-amino-6-(5-phosphoribosylamino)uracil reductase [EC:3.5.4.26 1.1.1.193]	0.03	8.6E-03	9.2E-03	0.04	
K13038	phosphopantothenoylcysteine decarboxylase / phosphopantothenate---cysteine ligase [EC:4.1.1.36 6.3.2.5]	-0.03	9.2E-03	9.8E-03	-0.06	
K01843	lysine 2,3-aminomutase [EC:5.4.3.2]	-0.03	9.5E-03	1.0E-02	0.14	
K01962	acetyl-CoA carboxylase carboxyl transferase subunit alpha [EC:6.4.1.2 2.1.3.15]	-0.03	9.5E-03	1.0E-02	-0.13	
K02123	V/A-type H+/Na+-transporting ATPase subunit I	-0.03	9.6E-03	1.0E-02	0.13	
K01692	enoyl-CoA hydratase [EC:4.2.1.17]	-0.03	1.1E-02	1.2E-02	-0.17	
K01960	pyruvate carboxylase subunit B [EC:6.4.1.1]	0.03	1.1E-02	1.2E-02	0.22	
K14157	alpha-aminoadipic semialdehyde synthase [EC:1.5.1.8 1.5.1.9]	-0.03	1.2E-02	1.3E-02	-0.02	
K01243	adenosylhomocysteine nucleosidase [EC:3.2.2.9]	-0.03	1.2E-02	1.3E-02	-0.07	
K06282	hydrogenase small subunit [EC:1.12.99.6]	0.03	1.3E-02	1.4E-02	-0.04	
K15987	K(+)-stimulated pyrophosphate-energized sodium pump [EC:7.1.3.2]	-0.03	1.4E-02	1.5E-02	0.13	
K00075	UDP-N-acetylmuramate dehydrogenase [EC:1.3.1.98]	0.03	1.4E-02	1.5E-02	0.02	
K05946	N-acetylglucosaminyldiphosphoundecaprenol N-acetyl-beta-D-mannosaminyltransferase [EC:2.4.1.187]	0.03	1.4E-02	1.5E-02	0.16	
K07258	serine-type D-Ala-D-Ala carboxypeptidase (penicillin-binding protein 5/6) [EC:3.4.16.4]	0.03	1.8E-02	1.9E-02	-0.08	
K03784	purine-nucleoside phosphorylase [EC:2.4.2.1]	-0.03	2.2E-02	2.3E-02	-0.13	
K03272	D-beta-D-heptose 7-phosphate kinase / D-beta-D-heptose 1-phosphate adenosyltransferase [EC:2.7.1.167 2.7.7.70]	-0.03	2.2E-02	2.3E-02	-0.08	
K03394	precorrin-2/cobalt-factor-2 C20-methyltransferase [EC:2.1.1.130 2.1.1.151]	0.03	2.5E-02	2.6E-02	0.18	
K00989	ribonuclease PH [EC:2.7.7.56]	-0.03	2.5E-02	2.6E-02	-0.09	
K03179	4-hydroxybenzoate polyprenyltransferase [EC:2.5.1.39]	0.03	2.7E-02	2.9E-02	0.00	
K00041	tagaturonate reductase [EC:1.1.1.58]	0.03	2.7E-02	2.9E-02	0.12	
K03169	DNA topoisomerase III [EC:5.6.2.1]	-0.03	2.8E-02	3.0E-02	0.11	

Predictor	Description	Pearson's r	P	FDR-adjusted P	Spearman's rho	Associated with fractures (P<0.05)
K10206	LL-diaminopimelate aminotransferase [EC:2.6.1.83]	-0.03	2.9E-02	3.1E-02	0.18	
K01518	bis(5'-nucleosidyl)-tetraphosphatase [EC:3.6.1.17]	-0.03	3.1E-02	3.3E-02	-0.02	yes
K01186	sialidase-1 [EC:3.2.1.18]	0.03	3.1E-02	3.3E-02	0.16	
K00326	cytochrome-b5 reductase [EC:1.6.2.2]	-0.03	3.1E-02	3.3E-02	-0.02	yes
K05929	phosphoethanolamine N-methyltransferase [EC:2.1.1.103]	-0.03	3.1E-02	3.3E-02	-0.02	yes
K02495	oxygen-independent coproporphyrinogen III oxidase [EC:1.3.98.3]	-0.03	3.2E-02	3.4E-02	-0.11	
K00661	maltose O-acetyltransferase [EC:2.3.1.79]	-0.03	3.5E-02	3.7E-02	0.10	yes
K01127	glycosylphosphatidylinositol phospholipase D [EC:3.1.4.50]	-0.03	3.5E-02	3.7E-02	-0.02	yes
K01868	threonyl-tRNA synthetase [EC:6.1.1.3]	0.03	3.5E-02	3.7E-02	0.05	
K00634	phosphate butyryltransferase [EC:2.3.1.19]	0.03	3.5E-02	3.7E-02	0.20	
K00721	dolichol-phosphate mannosyltransferase [EC:2.4.1.83]	-0.02	3.6E-02	3.8E-02	0.13	
K06046	long-chain-fatty-acid---luciferin-component ligase [EC:6.2.1.19]	-0.02	3.9E-02	4.1E-02	-0.08	
K00864	glycerol kinase [EC:2.7.1.30]	0.02	3.9E-02	4.1E-02	0.11	
K01714	4-hydroxy-tetrahydrodipicolinate synthase [EC:4.3.3.7]	0.02	4.1E-02	4.3E-02	0.11	
K01738	cysteine synthase [EC:2.5.1.47]	-0.02	4.2E-02	4.4E-02	-0.04	
K12140	hydrogenase-4 component E [EC:1.-.-.-]	0.02	4.4E-02	4.6E-02	0.01	
K00349	Na+-transporting NADH:ubiquinone oxidoreductase subunit D [EC:7.2.1.1]	-0.02	4.4E-02	4.6E-02	0.14	
K07568	S-adenosylmethionine:tRNA ribosyltransferase-isomerase [EC:2.4.99.17]	0.02	4.5E-02	4.7E-02	0.17	
K02412	flagellum-specific ATP synthase [EC:7.4.2.8]	-0.02	4.9E-02	5.2E-02	-0.13	
K03581	exodeoxyribonuclease V alpha subunit [EC:3.1.11.5]	-0.02	5.5E-02	5.8E-02	0.03	
K00899	5-methylthioribose kinase [EC:2.7.1.100]	0.02	5.5E-02	5.8E-02	-0.11	
K00346	Na+-transporting NADH:ubiquinone oxidoreductase subunit A [EC:7.2.1.1]	-0.02	6.2E-02	6.5E-02	0.15	
K06281	hydrogenase large subunit [EC:1.12.99.6]	0.02	6.3E-02	6.6E-02	-0.05	
K00759	adenine phosphoribosyltransferase [EC:2.4.2.7]	0.02	6.3E-02	6.6E-02	0.03	
K15923	alpha-L-fucosidase 2 [EC:3.2.1.51]	0.02	6.5E-02	6.8E-02	0.17	
K07646	two-component system, OmpR family, sensor histidine kinase KdpD [EC:2.7.13.3]	0.02	6.5E-02	6.8E-02	0.03	
K00347	Na+-transporting NADH:ubiquinone oxidoreductase subunit B [EC:7.2.1.1]	-0.02	6.6E-02	6.9E-02	0.14	
K00794	6,7-dimethyl-8-ribityllumazine synthase [EC:2.5.1.78]	-0.02	6.9E-02	7.2E-02	-0.06	
K00973	glucose-1-phosphate thymidylyltransferase [EC:2.7.7.24]	-0.02	7.0E-02	7.3E-02	0.04	
K03737	pyruvate-ferredoxin/flavodoxin oxidoreductase [EC:1.2.7.1 1.2.7.-]	0.02	7.7E-02	8.1E-02	0.02	
K00929	butyrate kinase [EC:2.7.2.7]	-0.02	7.7E-02	8.1E-02	0.16	
K01805	xylose isomerase [EC:5.3.1.5]	0.02	8.1E-02	8.5E-02	0.09	
K00350	Na+-transporting NADH:ubiquinone oxidoreductase subunit E [EC:7.2.1.1]	0.02	8.1E-02	8.5E-02	0.22	
K00348	Na+-transporting NADH:ubiquinone oxidoreductase subunit C [EC:7.2.1.1]	-0.02	8.4E-02	8.8E-02	0.15	
K03426	NAD+ diphosphatase [EC:3.6.1.22]	0.02	8.4E-02	8.8E-02	0.03	
K01195	beta-glucuronidase [EC:3.2.1.31]	0.02	8.7E-02	9.1E-02	-0.02	
K02552	menaquinone-specific isochorismate synthase [EC:5.4.4.2]	-0.02	8.8E-02	9.2E-02	-0.08	
K00164	2-oxoglutarate dehydrogenase E1 component [EC:1.2.4.2]	-0.02	8.8E-02	9.2E-02	-0.08	
K00351	Na+-transporting NADH:ubiquinone oxidoreductase subunit F [EC:7.2.1.1]	0.02	9.2E-02	9.6E-02	0.22	
K08297	crotonobetainyl-CoA dehydrogenase [EC:1.3.8.13]	0.02	9.4E-02	9.8E-02	-0.08	
K00876	uridine kinase [EC:2.7.1.48]	-0.02	1.0E-01	1.0E-01	0.05	
K09680	type II pantothenate kinase [EC:2.7.1.33]	-0.02	1.0E-01	1.0E-01	0.15	

Predictor	Description	Pearson's r	P	FDR-adjusted P	Spearman's rho	Associated with fractures (P<0.05)
K01223	6-phospho-beta-glucosidase [EC:3.2.1.86]	-0.02	1.0E-01	1.0E-01	-0.11	
K14441	ribosomal protein S12 methylthiotransferase [EC:2.8.4.4]	0.02	1.1E-01	1.1E-01	0.03	
K01785	aldose 1-epimerase [EC:5.1.3.3]	-0.02	1.1E-01	1.1E-01	0.15	
K07154	serine/threonine-protein kinase HipA [EC:2.7.11.1]	-0.02	1.1E-01	1.1E-01	0.08	
K04069	pyruvate formate lyase activating enzyme [EC:1.97.1.4]	0.02	1.1E-01	1.1E-01	0.07	
K03741	arsenate reductase (thioredoxin) [EC:1.20.4.4]	-0.02	1.2E-01	1.2E-01	0.15	
K01790	dTDP-4-dehydrorhamnose 3,5-epimerase [EC:5.1.3.13]	-0.02	1.2E-01	1.2E-01	0.13	
K00991	2-C-methyl-D-erythritol 4-phosphate cytidylyltransferase [EC:2.7.7.60]	-0.02	1.3E-01	1.4E-01	0.11	
K16212	4-O-beta-D-mannosyl-D-glucose phosphorylase [EC:2.4.1.281]	0.02	1.3E-01	1.4E-01	0.18	
K01077	alkaline phosphatase [EC:3.1.3.1]	-0.02	1.3E-01	1.4E-01	0.00	
K01709	CDP-glucose 4,6-dehydratase [EC:4.2.1.45]	0.02	1.3E-01	1.4E-01	0.17	
K13497	anthranilate synthase/phosphoribosyltransferase [EC:4.1.3.27 2.4.2.18]	0.02	1.4E-01	1.5E-01	-0.03	
K00012	UDPglucose 6-dehydrogenase [EC:1.1.1.22]	-0.02	1.4E-01	1.5E-01	-0.04	
K00978	glucose-1-phosphate cytidylyltransferase [EC:2.7.7.33]	0.02	1.5E-01	1.6E-01	0.17	
K00175	2-oxoglutarate/2-oxoacid ferredoxin oxidoreductase subunit beta [EC:1.2.7.3 1.2.7.11]	-0.02	1.5E-01	1.6E-01	0.17	
K12410	NAD-dependent deacetylase [EC:2.3.1.286]	0.02	1.5E-01	1.6E-01	0.02	
K02372	3-hydroxyacyl-[acyl-carrier-protein] dehydratase [EC:4.2.1.59]	0.02	1.6E-01	1.7E-01	0.09	
K00847	fructokinase [EC:2.7.1.4]	0.02	1.6E-01	1.7E-01	0.16	
K00265	glutamate synthase (NADPH) large chain [EC:1.4.1.13]	0.02	1.7E-01	1.8E-01	0.10	
K01155	type II restriction enzyme [EC:3.1.21.4]	-0.02	1.7E-01	1.8E-01	0.15	
K01278	dipeptidyl-peptidase 4 [EC:3.4.14.5]	-0.02	1.7E-01	1.8E-01	0.17	
K06182	23S rRNA pseudouridine2604 synthase [EC:5.4.99.21]	-0.02	1.8E-01	1.9E-01	-0.12	
K02619	4-amino-4-deoxychorismate lyase [EC:4.1.3.38]	0.02	1.8E-01	1.9E-01	0.02	
K11751	5'-nucleotidase / UDP-sugar diphosphatase [EC:3.1.3.5 3.6.1.45]	0.02	1.8E-01	1.9E-01	-0.04	
K07056	16S rRNA (cytidine1402-2'-O)-methyltransferase [EC:2.1.1.198]	-0.02	1.9E-01	2.0E-01	-0.01	
K06223	DNA adenine methylase [EC:2.1.1.72]	-0.02	1.9E-01	2.0E-01	-0.05	
K02431	L-fucose mutarotase [EC:5.1.3.29]	-0.02	1.9E-01	2.0E-01	-0.07	
K01235	alpha-glucuronidase [EC:3.2.1.139]	-0.02	1.9E-01	2.0E-01	0.15	
K03043	DNA-directed RNA polymerase subunit beta [EC:2.7.7.6]	0.02	2.1E-01	2.2E-01	-0.01	
K01572	NA	0.02	2.1E-01	2.2E-01	0.21	
K01841	phosphoenolpyruvate phosphomutase [EC:5.4.2.9]	0.02	2.1E-01	2.2E-01	0.01	
K03274	ADP-L-glycero-D-manno-heptose 6-epimerase [EC:5.1.3.20]	-0.01	2.1E-01	2.2E-01	-0.07	
K01792	glucose-6-phosphate 1-epimerase [EC:5.1.3.15]	-0.01	2.2E-01	2.3E-01	-0.06	
K08679	UDP-glucuronate 4-epimerase [EC:5.1.3.6]	0.01	2.2E-01	2.3E-01	0.01	
K00560	thymidylate synthase [EC:2.1.1.45]	-0.01	2.3E-01	2.4E-01	0.00	
K02232	adenosylcobyric acid synthase [EC:6.3.5.10]	-0.01	2.4E-01	2.5E-01	0.12	
K00865	glycerate 2-kinase [EC:2.7.1.165]	-0.01	2.4E-01	2.5E-01	-0.02	
K11527	two-component system, sensor histidine kinase and response regulator [EC:2.7.13.3]	0.01	2.5E-01	2.6E-01	0.22	
K13051	L-asparaginase / beta-aspartyl-peptidase [EC:3.5.1.1 3.4.19.5]	0.01	2.5E-01	2.6E-01	0.05	
K09459	phosphonopyruvate decarboxylase [EC:4.1.1.82]	0.01	2.6E-01	2.7E-01	0.01	
K03340	diaminopimelate dehydrogenase [EC:1.4.1.16]	-0.01	2.7E-01	2.8E-01	0.13	
K01143	exodeoxyribonuclease (lambda-induced) [EC:3.1.11.3]	0.01	2.9E-01	3.0E-01	0.01	

Predictor	Description	Pearson's r	P	FDR-adjusted P	Spearman's rho	Associated with fractures (P<0.05)
K00941	hydroxymethylpyrimidine/phosphomethylpyrimidine kinase [EC:2.7.1.49 2.7.4.7]	0.01	3.0E-01	3.1E-01	0.00	
K13002	glycosyltransferase [EC:2.4.1.-]	-0.01	3.0E-01	3.1E-01	0.15	
K00798	cob(I)alamin adenosyltransferase [EC:2.5.1.17]	-0.01	3.0E-01	3.1E-01	0.14	
K01847	methylmalonyl-CoA mutase [EC:5.4.99.2]	0.01	3.1E-01	3.2E-01	0.05	
K02124	V/A-type H+/Na+-transporting ATPase subunit K	-0.01	3.1E-01	3.2E-01	0.19	
K00937	polyphosphate kinase [EC:2.7.4.1]	-0.01	3.1E-01	3.2E-01	0.11	
K02118	V/A-type H+/Na+-transporting ATPase subunit B	-0.01	3.2E-01	3.3E-01	0.19	
K01197	hyaluronoglucosaminidase [EC:3.2.1.35]	-0.01	3.2E-01	3.3E-01	0.14	
K01183	chitinase [EC:3.2.1.14]	-0.01	3.3E-01	3.4E-01	0.15	
K01081	5'-nucleotidase [EC:3.1.3.5]	-0.01	3.3E-01	3.4E-01	0.15	
K01654	N-acetylneuraminate synthase [EC:2.5.1.56]	0.01	3.3E-01	3.4E-01	0.11	
K00946	thiamine-monophosphate kinase [EC:2.7.4.16]	-0.01	3.3E-01	3.4E-01	-0.02	
K08680	2-succinyl-6-hydroxy-2,4-cyclohexadiene-1-carboxylate synthase [EC:4.2.99.20]	-0.01	3.3E-01	3.4E-01	-0.07	
K00048	lactaldehyde reductase [EC:1.1.1.77]	0.01	3.4E-01	3.5E-01	0.03	
K00343	NADH-quinone oxidoreductase subunit N [EC:7.1.1.2]	-0.01	3.4E-01	3.5E-01	-0.04	
K03218	23S rRNA (guanosine2251-2'-O)-methyltransferase [EC:2.1.1.185]	-0.01	3.4E-01	3.5E-01	-0.02	
K03772	FKBP-type peptidyl-prolyl cis-trans isomerase FkpA [EC:5.2.1.8]	0.01	3.4E-01	3.5E-01	0.00	
K02188	cobalt-precorrin-5B (C1)-methyltransferase [EC:2.1.1.195]	-0.01	3.5E-01	3.6E-01	0.13	
K10563	formamidopyrimidine-DNA glycosylase [EC:3.2.2.23 4.2.99.18]	-0.01	3.5E-01	3.6E-01	-0.05	
K00879	L-fuculokinase [EC:2.7.1.51]	-0.01	3.6E-01	3.7E-01	-0.06	
K03655	ATP-dependent DNA helicase RecG [EC:3.6.4.12]	0.01	3.6E-01	3.7E-01	0.13	
K00970	poly(A) polymerase [EC:2.7.7.19]	-0.01	3.6E-01	3.7E-01	-0.04	
K05970	sialate O-acetylesterase [EC:3.1.1.53]	-0.01	3.6E-01	3.7E-01	0.16	
K00381	sulfite reductase (NADPH) hemoprotein beta-component [EC:1.8.1.2]	-0.01	3.8E-01	3.9E-01	-0.07	
K01205	alpha-N-acetylglucosaminidase [EC:3.2.1.50]	0.01	3.9E-01	4.0E-01	0.19	
K01012	biotin synthase [EC:2.8.1.6]	-0.01	4.0E-01	4.1E-01	-0.04	
K01662	1-deoxy-D-xylulose-5-phosphate synthase [EC:2.2.1.7]	0.01	4.1E-01	4.2E-01	0.16	
K00179	indolepyruvate ferredoxin oxidoreductase, alpha subunit [EC:1.2.7.8]	-0.01	4.1E-01	4.2E-01	0.17	
K10679	nitroreductase / dihydropteridine reductase [EC:1.-.-.- 1.5.1.34]	0.01	4.2E-01	4.3E-01	-0.04	
K01551	arsenite/tail-anchored protein-transporting ATPase [EC:7.3.2.7 7.3.-.-]	0.01	4.3E-01	4.4E-01	0.14	
K00835	valine--pyruvate aminotransferase [EC:2.6.1.66]	-0.01	4.4E-01	4.5E-01	-0.06	
K12141	hydrogenase-4 component F [EC:1.-.-.-]	0.01	4.6E-01	4.7E-01	-0.01	
K07271	lipopolysaccharide cholinephosphotransferase [EC:2.7.8.-]	0.01	4.7E-01	4.8E-01	0.13	
K00688	glycogen phosphorylase [EC:2.4.1.1]	-0.01	4.8E-01	4.9E-01	0.04	
K02190	sirohydrochlorin cobaltochelatase [EC:4.99.1.3]	0.01	5.1E-01	5.2E-01	0.14	
K00040	fructuronate reductase [EC:1.1.1.57]	-0.01	5.1E-01	5.2E-01	-0.11	
K01262	Xaa-Pro aminopeptidase [EC:3.4.11.9]	-0.01	5.1E-01	5.2E-01	0.11	
K01120	3',5'-cyclic-nucleotide phosphodiesterase [EC:3.1.4.17]	-0.01	5.1E-01	5.2E-01	-0.09	
K00380	sulfite reductase (NADPH) flavoprotein alpha-component [EC:1.8.1.2]	-0.01	5.3E-01	5.4E-01	-0.06	
K02224	cobyrinic acid a,c-diamide synthase [EC:6.3.5.9 6.3.5.11]	-0.01	5.3E-01	5.4E-01	0.13	
K01579	aspartate 1-decarboxylase [EC:4.1.1.11]	0.01	5.6E-01	5.7E-01	-0.01	
K01730	oligogalacturonide lyase [EC:4.2.2.6]	-0.01	5.6E-01	5.7E-01	0.15	

Predictor	Description	Pearson's r	P	FDR-adjusted P	Spearman's rho	Associated with fractures (P<0.05)
K01809	mannose-6-phosphate isomerase [EC:5.3.1.8]	-0.01	6.0E-01	6.1E-01	0.01	
K03940	NADH dehydrogenase (ubiquinone) Fe-S protein 7 [EC:7.1.1.2]	-0.01	6.1E-01	6.2E-01	-0.01	
K06859	glucose-6-phosphate isomerase, archaeal [EC:5.3.1.9]	-0.01	6.1E-01	6.2E-01	-0.11	
K00547	homocysteine S-methyltransferase [EC:2.1.1.10]	-0.01	6.2E-01	6.3E-01	-0.08	
K02687	ribosomal protein L11 methyltransferase [EC:2.1.1.-]	0.01	6.3E-01	6.4E-01	0.02	
K01126	glycerophosphoryl diester phosphodiesterase [EC:3.1.4.46]	-0.01	6.4E-01	6.5E-01	0.00	
K09565	peptidyl-prolyl isomerase F (cyclophilin D) [EC:5.2.1.8]	-0.01	6.6E-01	6.7E-01	-0.01	
K08299	crotonobetainyl-CoA hydratase [EC:4.2.1.149]	0.01	6.7E-01	6.8E-01	-0.10	
K03750	molybdopterin molybdotransferase [EC:2.10.1.1]	0.00	7.0E-01	7.1E-01	-0.11	
K00226	dihydroorotate dehydrogenase (fumarate) [EC:1.3.98.1]	0.00	7.3E-01	7.4E-01	0.21	
K03736	ethanolamine ammonia-lyase small subunit [EC:4.3.1.7]	0.00	7.3E-01	7.4E-01	-0.11	
K08094	6-phospho-3-hexuloisomerase [EC:5.3.1.27]	0.00	7.3E-01	7.4E-01	-0.10	
K15635	2,3-bisphosphoglycerate-independent phosphoglycerate mutase [EC:5.4.2.12]	0.00	7.4E-01	7.5E-01	0.15	
K01414	oligopeptidase A [EC:3.4.24.70]	0.00	7.6E-01	7.7E-01	-0.06	
K03797	carboxyl-terminal processing protease [EC:3.4.21.102]	0.00	7.6E-01	7.7E-01	0.12	
K05601	hydroxylamine reductase [EC:1.7.99.1]	0.00	7.6E-01	7.7E-01	-0.05	
K01918	pantoate--beta-alanine ligase [EC:6.3.2.1]	0.00	7.7E-01	7.8E-01	-0.02	
K07444	putative N6-adenine-specific DNA methylase [EC:2.1.1.-]	0.00	8.0E-01	8.1E-01	0.15	
K00174	2-oxoglutarate/2-oxoacid ferredoxin oxidoreductase subunit alpha [EC:1.2.7.3 1.2.7.11]	0.00	8.0E-01	8.1E-01	0.19	
K03100	signal peptidase I [EC:3.4.21.89]	0.00	8.1E-01	8.2E-01	0.11	
K10542	methyl-galactoside transport system ATP-binding protein [EC:7.5.2.11]	0.00	8.1E-01	8.2E-01	-0.10	
K06181	23S rRNA pseudouridine2457 synthase [EC:5.4.99.20]	0.00	8.2E-01	8.2E-01	-0.06	
K01206	alpha-L-fucosidase [EC:3.2.1.51]	0.00	8.2E-01	8.2E-01	0.21	
K01673	carbonic anhydrase [EC:4.2.1.1]	0.00	8.2E-01	8.2E-01	-0.05	
K00859	dephospho-CoA kinase [EC:2.7.1.24]	0.00	8.3E-01	8.3E-01	0.01	
K03470	ribonuclease HII [EC:3.1.26.4]	0.00	8.3E-01	8.3E-01	-0.01	
K00571	site-specific DNA-methyltransferase (adenine-specific) [EC:2.1.1.72]	0.00	8.5E-01	8.5E-01	-0.04	
K02041	phosphonate transport system ATP-binding protein [EC:7.3.2.2]	0.00	8.5E-01	8.5E-01	-0.10	
K13043	N-succinyl-L-ornithine transcarbamylase [EC:2.1.3.11]	0.00	8.7E-01	8.7E-01	0.18	
K00833	adenosylmethionine---8-amino-7-oxononanoate aminotransferase [EC:2.6.1.62]	0.00	8.8E-01	8.8E-01	-0.04	
K00703	starch synthase [EC:2.4.1.21]	0.00	9.0E-01	9.0E-01	0.00	
K01734	methylglyoxal synthase [EC:4.2.3.3]	0.00	9.0E-01	9.0E-01	0.00	
K00851	gluconokinase [EC:2.7.1.12]	0.00	9.3E-01	9.3E-01	-0.06	
K16055	trehalose 6-phosphate synthase/phosphatase [EC:2.4.1.15 3.1.3.12]	0.00	9.4E-01	9.4E-01	0.15	
K00018	glycerate dehydrogenase [EC:1.1.1.29]	0.00	9.4E-01	9.4E-01	0.16	
K06113	arabinan endo-1,5-alpha-L-arabinosidase [EC:3.2.1.99]	0.00	9.4E-01	9.4E-01	0.15	
K00651	homoserine O-succinyltransferase/O-acetyltransferase [EC:2.3.1.46 2.3.1.31]	0.00	9.6E-01	9.6E-01	-0.01	
K00060	threonine 3-dehydrogenase [EC:1.1.1.103]	0.00	9.6E-01	9.6E-01	-0.05	
K04720	threonine-phosphate decarboxylase [EC:4.1.1.81]	0.00	9.8E-01	9.8E-01	0.14	
K12343	3-oxo-5-alpha-steroid 4-dehydrogenase 1 [EC:1.3.1.22]	0.00	9.9E-01	9.9E-01	0.18	

Supplementary Table 20. Top 25 functional groups correlated with the relative abundance of Proteobacteria and associated with fractures

Predictor	Description	Pearson's r	P	FDR-adjusted P	Spearman's rho	Associated with fractures (P< 0.05)	Implicated in amino acid metabolism
K16149	1,4-alpha-glucan branching enzyme [EC:2.4.1.18]	-0.46	<5.9E-323	<1.8E-320	-0.47	yes	
K06208	chorismate mutase [EC:5.4.99.5]	-0.45	<5.9E-323	<1.8E-320	-0.46	yes	yes
K13282	cyanophycinase [EC:3.4.15.6]	-0.45	<5.9E-323	<1.8E-320	-0.46	yes	
K07651	two-component system, OmpR family, sensor histidine kinase ResE [EC:2.7.13.3]	-0.44	<5.9E-323	<1.8E-320	-0.45	yes	
K02636	cytochrome b6-f complex iron-sulfur subunit [EC:7.1.1.6]	-0.43	5.9E-323	1.8E-320	-0.44	yes	
K11783	futalosine hydrolase [EC:3.2.2.26]	-0.43	4.3E-321	1.2E-318	-0.44	yes	
K03519	aerobic carbon-monoxide dehydrogenase medium subunit [EC:1.2.5.3]	-0.43	1.6E-319	3.7E-317	-0.45	yes	
K00303	sarcosine oxidase, subunit beta [EC:1.5.3.1]	-0.43	1.7E-319	3.7E-317	-0.45	yes	yes
K02277	cytochrome c oxidase subunit IV [EC:7.1.1.9]	-0.43	6.1E-319	1.2E-316	-0.44	yes	
K01556	kynureninase [EC:3.7.1.3]	-0.43	2.3E-318	4.3E-316	-0.44	yes	yes
K07130	arylformamidase [EC:3.5.1.9]	-0.43	1.2E-317	1.9E-315	-0.44	yes	yes
K13598	two-component system, NtrC family, nitrogen regulation sensor histidine kinase NtrY [EC:2.7.13.3]	-0.43	5.8E-316	8.7E-314	-0.44	yes	
K03392	aminocarboxymuconate-semialdehyde decarboxylase [EC:4.1.1.45]	-0.43	4.3E-314	5.9E-312	-0.43	yes	yes
K00263	leucine dehydrogenase [EC:1.4.1.9]	-0.43	6.8E-314	8.5E-312	-0.44	yes	yes
K08097	phosphosulfolactate synthase [EC:4.4.1.19]	-0.43	3.5E-313	4.2E-311	-0.44	yes	
K00958	sulfate adenylyltransferase [EC:2.7.7.4]	-0.43	6.1E-313	7.1E-311	-0.44	yes	
K15921	arabinoxylan arabinofuranohydrolase [EC:3.2.1.55]	-0.43	1.7E-312	1.9E-310	-0.44	yes	
K15924	glucuronoarabinoxylan endo-1,4-beta-xylanase [EC:3.2.1.136]	-0.43	3.2E-312	3.4E-310	-0.44	yes	
K11180	dissimilatory sulfite reductase alpha subunit [EC:1.8.99.5]	-0.43	3.8E-312	4.0E-310	-0.43	yes	
K04038	light-independent protochlorophyllide reductase subunit N [EC:1.3.7.7]	-0.43	4.5E-312	4.5E-310	-0.43	yes	
K07469	aldehyde oxidoreductase [EC:1.2.99.7]	-0.43	5.4E-312	5.3E-310	-0.44	yes	
K00533	ferredoxin hydrogenase large subunit [EC:1.12.7.2]	-0.43	4.6E-311	4.3E-309	-0.44	yes	
K15856	GDP-4-dehydro-6-deoxy-D-mannose reductase [EC:1.1.1.281]	-0.43	6.9E-311	6.1E-309	-0.44	yes	
K12454	CDP-paratose 2-epimerase [EC:5.1.3.10]	-0.43	7.3E-311	6.3E-309	-0.43	yes	
K07094	heptaprenylglyceryl phosphate synthase [EC:2.5.1.-]	-0.43	9.4E-310	7.9E-308	-0.43	yes	

Pearson's correlation between functional groups and relative abundance of Proteobacteria. n = 7043 (1092 cases). FDR, false discovery rate

Supplementary Table 21. Correlations between the relative abundance of Tenericutes and functional groups

Pearson's correlation between functional groups and the relative abundance of Tenericutes. n = 7043 (1092 cases). FDR, false discovery rate

Predictor	Description	Pearson's r	P	FDR-adjusted P	Spearman's rho	Associated with fractures (P<0.05)
K08651	thermitase [EC:3.4.21.66]	0.45	<5.9E-323	8.9E-320	0.52	yes
K15898	pseudaminic acid synthase [EC:2.5.1.97]	0.44	<5.9E-323	8.9E-320	0.48	yes
K00100	butanol dehydrogenase [EC:1.1.1.-]	0.43	4.4E-318	4.4E-315	0.47	yes
K00354	NADPH2 dehydrogenase [EC:1.6.99.1]	0.42	4.7E-304	3.5E-301	0.45	yes
K14128	F420-non-reducing hydrogenase small subunit [EC:1.12.99.- 1.8.98.5]	0.42	6.2E-294	3.7E-291	0.46	yes
K14080	[methyl-Co(III) methanol/glycine betaine-specific corrinoid protein]:coenzyme M methyltransferase [EC:2.1.1.246 2.1.1.377]	0.42	2.2E-292	1.1E-289	0.45	yes
K14126	F420-non-reducing hydrogenase large subunit [EC:1.12.99.- 1.8.98.5]	0.42	3.5E-292	1.5E-289	0.46	yes
K00532	ferredoxin hydrogenase [EC:1.12.7.2]	0.41	1.3E-288	4.9E-286	0.44	yes
K01959	pyruvate carboxylase subunit A [EC:6.4.1.1]	0.41	3.3E-286	1.1E-283	0.45	yes
K03462	nicotinamide phosphoribosyltransferase [EC:2.4.2.12]	0.41	1.3E-283	3.9E-281	0.44	yes
K05979	2-phosphosulfolactate phosphatase [EC:3.1.3.71]	0.41	6.0E-283	1.6E-280	0.44	
K00021	hydroxymethylglutaryl-CoA reductase (NADPH) [EC:1.1.1.34]	0.41	6.9E-281	1.7E-278	0.45	yes
K00150	glyceraldehyde-3-phosphate dehydrogenase (NAD(P)) [EC:1.2.1.59]	0.41	1.4E-280	3.2E-278	0.44	yes
K00263	leucine dehydrogenase [EC:1.4.1.9]	0.41	3.7E-278	8.0E-276	0.43	yes
K15862	cytochrome c oxidase cbb3-type subunit I/II [EC:7.1.1.9]	0.41	2.1E-276	4.2E-274	0.42	
K12454	CDP-paratose 2-epimerase [EC:5.1.3.10]	0.40	1.6E-274	3.0E-272	0.43	yes
K00404	cytochrome c oxidase cbb3-type subunit I [EC:7.1.1.9]	0.40	2.6E-274	4.6E-272	0.42	yes
K07651	two-component system, OmpR family, sensor histidine kinase ResE [EC:2.7.13.3]	0.40	5.7E-274	9.6E-272	0.43	yes
K04566	lysyl-tRNA synthetase, class I [EC:6.1.1.6]	0.40	1.3E-273	2.1E-271	0.44	yes
K00809	deoxyhypusine synthase [EC:2.5.1.46]	0.40	2.4E-273	3.6E-271	0.44	yes
K10747	DNA ligase 1 [EC:6.5.1.1 6.5.1.6 6.5.1.7]	0.40	4.5E-273	6.5E-271	0.44	yes
K13282	cyanophycinase [EC:3.4.15.6]	0.40	1.6E-272	2.2E-270	0.43	yes
K01622	fructose 1,6-bisphosphate aldolase/phosphatase [EC:4.1.2.13 3.1.3.11]	0.40	2.7E-272	3.5E-270	0.44	yes
K00202	formylmethanofuran dehydrogenase subunit C [EC:1.2.7.12]	0.40	3.7E-271	4.7E-269	0.44	yes
K02277	cytochrome c oxidase subunit IV [EC:7.1.1.9]	0.40	1.7E-269	2.1E-267	0.42	yes
K00372	assimilatory nitrate reductase catalytic subunit [EC:1.7.99.-]	0.40	5.7E-269	6.6E-267	0.42	yes
K00952	nicotinamide-nucleotide adenylyltransferase [EC:2.7.7.1]	0.40	3.1E-268	3.5E-266	0.43	yes
K00672	formylmethanofuran--tetrahydromethanopterin N-formyltransferase [EC:2.3.1.101]	0.40	1.3E-267	1.4E-265	0.44	yes
K00441	coenzyme F420 hydrogenase subunit beta [EC:1.12.98.1]	0.40	5.0E-267	5.2E-265	0.43	yes
K00201	formylmethanofuran dehydrogenase subunit B [EC:1.2.7.12]	0.40	2.7E-265	2.7E-263	0.43	yes
K15778	phosphomannomutase / phosphoglucomutase [EC:5.4.2.8 5.4.2.2]	0.40	2.9E-265	2.8E-263	0.43	yes
K07094	heptaprenylglyceryl phosphate synthase [EC:2.5.1.-]	0.40	2.0E-264	1.9E-262	0.42	yes
K01499	methenyltetrahydromethanopterin cyclohydrolase [EC:3.5.4.27]	0.40	7.8E-264	7.1E-262	0.44	yes
K02275	cytochrome c oxidase subunit II [EC:7.1.1.9]	0.40	1.4E-263	1.2E-261	0.41	yes
K15856	GDP-4-dehydro-6-deoxy-D-mannose reductase [EC:1.1.1.281]	0.40	3.1E-263	2.7E-261	0.42	yes
K07178	RIO kinase 1 [EC:2.7.11.1]	0.40	5.2E-263	4.4E-261	0.43	yes
K15921	arabinoxylan arabinofuranohydrolase [EC:3.2.1.55]	0.40	1.8E-262	1.5E-260	0.42	yes
K08097	phosphosulfolactate synthase [EC:4.4.1.19]	0.40	4.3E-262	3.4E-260	0.42	yes

Predictor	Description	Pearson's r	P	FDR-adjusted P	Spearman's rho	Associated with fractures (P<0.05)
K05873	adenylate cyclase, class 2 [EC:4.6.1.1]	0.39	2.0E-261	1.5E-259	0.43	yes
K15372	taurine---2-oxoglutarate transaminase [EC:2.6.1.55]	0.39	9.1E-261	6.9E-259	0.42	yes
K15924	glucuronoarabinoxylan endo-1,4-beta-xylanase [EC:3.2.1.136]	0.39	1.8E-260	1.3E-258	0.42	yes
K07151	dolichyl-diphosphooligosaccharide---protein glycosyltransferase [EC:2.4.99.18]	0.39	7.1E-260	5.1E-258	0.43	yes
K11212	LPPG:FO 2-phospho-L-lactate transferase [EC:2.7.8.28]	0.39	8.4E-260	5.9E-258	0.43	yes
K13280	signal peptidase I [EC:3.4.21.89]	0.39	1.2E-259	8.2E-258	0.42	yes
K03795	sirohydrochlorin cobaltochelatase [EC:4.99.1.3]	0.39	3.4E-259	2.3E-257	0.43	yes
K11646	3-dehydroquinate synthase II [EC:1.4.1.24]	0.39	8.6E-259	5.6E-257	0.43	yes
K14259	2-dehydro-3-deoxy-D-arabinonate dehydratase [EC:4.2.1.141]	0.39	5.2E-258	3.3E-256	0.42	yes
K06982	pantoate kinase [EC:2.7.1.169]	0.39	1.8E-257	1.1E-255	0.43	yes
K01046	triacylglycerol lipase [EC:3.1.1.3]	0.39	1.2E-256	7.4E-255	0.41	yes
K16216	benzil reductase ((S)-benzoin forming) [EC:1.1.1.320]	0.39	1.4E-256	8.4E-255	0.42	yes
K14941	2-phospho-L-lactate/phosphoenolpyruvate guanylyltransferase [EC:2.7.7.68 2.7.7.105]	0.39	3.0E-256	1.8E-254	0.42	yes
K04093	chorismate mutase [EC:5.4.99.5]	0.39	8.9E-256	5.2E-254	0.42	yes
K12234	coenzyme F420-0:L-glutamate ligase / coenzyme F420-1:gamma-L-glutamate ligase [EC:6.3.2.31 6.3.2.34]	0.39	1.9E-255	1.1E-253	0.42	yes
K00612	carbamoyltransferase [EC:2.1.3.-]	0.39	6.5E-255	3.6E-253	0.41	yes
K03433	proteasome beta subunit [EC:3.4.25.1]	0.39	3.2E-254	1.8E-252	0.42	yes
K03432	proteasome alpha subunit [EC:3.4.25.1]	0.39	1.8E-253	9.7E-252	0.42	yes
K02303	uroporphyrin-III C-methyltransferase [EC:2.1.1.107]	0.39	2.0E-253	1.1E-251	0.42	yes
K04799	flap endonuclease-1 [EC:3.-.-.-]	0.39	5.2E-252	2.7E-250	0.43	yes
K11261	formylmethanofuran dehydrogenase subunit E [EC:1.2.7.12]	0.39	7.0E-252	3.6E-250	0.42	yes
K16149	1,4-alpha-glucan branching enzyme [EC:2.4.1.18]	0.39	2.3E-251	1.2E-249	0.41	yes
K12995	O-antigen biosynthesis alpha-1,3-mannosyltransferase [EC:2.4.1.348 2.4.1.-]	0.39	3.0E-251	1.5E-249	0.41	yes
K15429	tRNA (guanine37-N1)-methyltransferase [EC:2.1.1.228]	0.39	3.2E-251	1.6E-249	0.43	yes
K09809	CDP-glycerol glycerophosphotransferase [EC:2.7.8.12]	0.39	4.7E-251	2.3E-249	0.41	yes
K01563	haloalkane dehalogenase [EC:3.8.1.5]	0.39	7.1E-251	3.3E-249	0.40	yes
K04794	peptidyl-tRNA hydrolase, PTH2 family [EC:3.1.1.29]	0.39	7.8E-251	3.6E-249	0.42	yes
K13767	enoyl-CoA hydratase [EC:4.2.1.17]	0.39	3.9E-250	1.8E-248	0.41	yes
K06012	spore protease [EC:3.4.24.78]	0.39	6.1E-250	2.7E-248	0.41	yes
K01556	kynureninase [EC:3.7.1.3]	0.39	8.9E-250	3.9E-248	0.41	yes
K06981	isopentenyl phosphate kinase [EC:2.7.4.26]	0.39	1.7E-249	7.4E-248	0.43	yes
K03041	DNA-directed RNA polymerase subunit A' [EC:2.7.7.6]	0.39	2.1E-249	9.1E-248	0.43	yes
K03050	DNA-directed RNA polymerase subunit E\ [EC:2.7.7.6]	0.39	6.0E-249	2.5E-247	0.43	yes
K00125	formate dehydrogenase (coenzyme F420) beta subunit [EC:1.17.98.3 1.8.98.6]	0.39	9.2E-249	3.9E-247	0.42	yes
K03170	reverse gyrase [EC:5.6.2.2 3.6.4.12]	0.39	1.8E-248	7.4E-247	0.47	yes
K03166	DNA topoisomerase VI subunit A [EC:5.6.2.2]	0.39	1.9E-248	7.7E-247	0.43	yes
K01724	4a-hydroxytetrahydrobiopterin dehydratase [EC:4.2.1.96]	0.39	3.7E-248	1.5E-246	0.40	yes
K00584	tetrahydromethanopterin S-methyltransferase subunit H [EC:2.1.1.86]	0.38	1.3E-247	5.2E-246	0.42	yes
K00303	sarcosine oxidase, subunit beta [EC:1.5.3.1]	0.38	1.4E-247	5.5E-246	0.41	yes
K03047	DNA-directed RNA polymerase subunit D [EC:2.7.7.6]	0.38	1.6E-247	6.2E-246	0.43	yes
K07254	tRNA (cytidine56-2'-O)-methyltransferase [EC:2.1.1.206]	0.38	3.0E-247	1.1E-245	0.43	yes
K03660	N-glycosylase/DNA lyase [EC:3.2.2.- 4.2.99.18]	0.38	4.9E-247	1.8E-245	0.41	yes

Predictor	Description	Pearson's r	P	FDR-adjusted P	Spearman's rho	Associated with fractures (P<0.05)
K00507	stearoyl-CoA desaturase (Delta-9 desaturase) [EC:1.14.19.1]	0.38	6.0E-247	2.2E-245	0.41	yes
K04480	methanol---5-hydroxybenzimidazolylcobamide Co-methyltransferase [EC:2.1.1.90]	0.38	7.9E-247	2.9E-245	0.42	yes
K13668	phosphatidyl-myo-inositol dimannoside synthase [EC:2.4.1.346]	0.38	1.8E-246	6.5E-245	0.40	yes
K00555	tRNA (guanine26-N2/guanine27-N2)-dimethyltransferase [EC:2.1.1.215 2.1.1.216]	0.38	7.2E-246	2.6E-244	0.43	yes
K07558	tRNA nucleotidyltransferase (CCA-adding enzyme) [EC:2.7.7.72]	0.38	8.0E-246	2.8E-244	0.43	yes
K09482	glutamyl-tRNA(Gln) amidotransferase subunit D [EC:6.3.5.7]	0.38	1.1E-245	3.8E-244	0.43	yes
K03538	ribonuclease P protein subunit POP4 [EC:3.1.26.5]	0.38	1.1E-245	3.8E-244	0.42	yes
K14446	crotonyl-CoA carboxylase/reductase [EC:1.3.1.85]	0.38	1.2E-245	4.1E-244	0.41	yes
K05715	2-phosphoglycerate kinase [EC:2.7.2.16]	0.38	3.3E-245	1.1E-243	0.42	yes
K03058	DNA-directed RNA polymerase subunit N [EC:2.7.7.6]	0.38	9.3E-245	3.1E-243	0.42	yes
K03540	ribonuclease P protein subunit RPR2 [EC:3.1.26.5]	0.38	9.4E-245	3.1E-243	0.43	yes
K02626	arginine decarboxylase [EC:4.1.1.19]	0.38	1.0E-244	3.3E-243	0.42	yes
K01090	protein phosphatase [EC:3.1.3.16]	0.38	1.7E-244	5.5E-243	0.40	yes
K07557	archaeosine synthase alpha-subunit [EC:2.6.1.97 2.6.1.-]	0.38	2.5E-244	8.0E-243	0.42	yes
K00320	5,10-methylenetetrahydromethanopterin reductase [EC:1.5.98.2]	0.38	3.6E-244	1.1E-242	0.42	yes
K06443	lycopene beta-cyclase [EC:5.5.1.19]	0.38	7.3E-244	2.3E-242	0.40	yes
K15916	glucose/mannose-6-phosphate isomerase [EC:5.3.1.9 5.3.1.8]	0.38	1.1E-243	3.4E-242	0.44	yes
K08587	clostripain [EC:3.4.22.8]	0.38	1.2E-243	3.7E-242	0.43	yes
K16619	phospholipase C / alpha-toxin [EC:3.1.4.3]	0.38	1.2E-243	3.7E-242	0.43	yes
K02683	DNA primase small subunit [EC:2.7.7.102]	0.38	1.5E-243	4.5E-242	0.42	yes
K00302	sarcosine oxidase, subunit alpha [EC:1.5.3.1]	0.38	1.7E-243	5.1E-242	0.40	yes
K06208	chorismate mutase [EC:5.4.99.5]	0.38	2.0E-243	5.9E-242	0.41	yes
K01768	adenylate cyclase [EC:4.6.1.1]	0.38	2.2E-243	6.4E-242	0.40	yes
K00304	sarcosine oxidase, subunit delta [EC:1.5.3.1]	0.38	2.9E-243	8.4E-242	0.40	yes
K02319	DNA polymerase, archaea type [EC:2.7.7.7]	0.38	3.9E-243	1.1E-241	0.42	yes
K01732	pectin lyase [EC:4.2.2.10]	0.38	1.8E-242	5.1E-241	0.41	yes
K03330	glutamyl-tRNA(Gln) amidotransferase subunit E [EC:6.3.5.7]	0.38	6.1E-242	1.7E-240	0.42	yes
K02274	cytochrome c oxidase subunit I [EC:7.1.1.9]	0.38	1.1E-241	3.1E-240	0.40	yes
K02594	homocitrate synthase NifV [EC:2.3.3.14]	0.38	1.6E-241	4.4E-240	0.41	yes
K03056	DNA-directed RNA polymerase subunit L [EC:2.7.7.6]	0.38	2.4E-241	6.6E-240	0.42	yes
K03051	DNA-directed RNA polymerase subunit F [EC:2.7.7.6]	0.38	4.8E-241	1.3E-239	0.42	yes
K00305	sarcosine oxidase, subunit gamma [EC:1.5.3.1]	0.38	8.1E-241	2.2E-239	0.40	yes
K01114	phospholipase C [EC:3.1.4.3]	0.38	4.9E-240	1.3E-238	0.40	yes
K02591	nitrogenase molybdenum-iron protein beta chain [EC:1.18.6.1]	0.38	3.8E-239	1.0E-237	0.40	yes
K00187	2-oxoisovalerate ferredoxin oxidoreductase beta subunit [EC:1.2.7.7]	0.38	5.4E-239	1.4E-237	0.43	yes
K07446	tRNA (guanine10-N2)-dimethyltransferase [EC:2.1.1.213]	0.38	8.6E-239	2.2E-237	0.42	yes
K00577	tetrahydromethanopterin S-methyltransferase subunit A [EC:2.1.1.86]	0.38	6.1E-238	1.6E-236	0.42	yes
K07055	tRNA wybutosine-synthesizing protein 2 [EC:2.5.1.114]	0.38	2.2E-237	5.6E-236	0.42	yes
K00486	kynurenine 3-monooxygenase [EC:1.14.13.9]	0.38	3.5E-237	8.9E-236	0.40	yes
K05521	ADP-ribosyl-[dinitrogen reductase] hydrolase [EC:3.2.2.24]	0.38	5.3E-237	1.3E-235	0.41	yes
K15408	cytochrome c oxidase subunit I+III [EC:7.1.1.9]	0.38	1.9E-236	4.7E-235	0.40	yes
K00356	NA	0.38	2.0E-236	4.9E-235	0.40	yes

Predictor	Description	Pearson's r	P	FDR-adjusted P	Spearman's rho	Associated with fractures (P<0.05)
K03537	ribonuclease P/MRP protein subunit POP5 [EC:3.1.26.5]	0.38	7.2E-236	1.8E-234	0.42	yes
K07732	riboflavin kinase, archaea type [EC:2.7.1.161]	0.38	8.1E-236	2.0E-234	0.42	yes
K01434	penicillin G amidase [EC:3.5.1.11]	0.38	9.9E-236	2.4E-234	0.40	yes
K00452	3-hydroxyanthranilate 3,4-dioxygenase [EC:1.13.11.6]	0.38	2.5E-235	6.0E-234	0.40	yes
K14654	2,5-diamino-6-(ribosylamino)-4(3H)-pyrimidinone 5'-phosphate reductase [EC:1.1.1.302]	0.38	3.2E-235	7.6E-234	0.42	yes
K05303	O-methyltransferase [EC:2.1.1.-]	0.38	4.0E-235	9.4E-234	0.43	
K13503	anthranilate synthase [EC:4.1.3.27]	0.38	6.4E-235	1.5E-233	0.41	yes
K02586	nitrogenase molybdenum-iron protein alpha chain [EC:1.18.6.1]	0.38	1.1E-234	2.6E-233	0.39	yes
K03539	ribonuclease P/MRP protein subunit RPP1 [EC:3.1.26.5]	0.38	4.1E-234	9.4E-233	0.42	yes
K01941	urea carboxylase [EC:6.3.4.6]	0.38	4.6E-234	1.1E-232	0.39	yes
K11781	5-amino-6-(D-ribitylamino)uracil---L-tyrosine 4-hydroxyphenyl transferase [EC:2.5.1.147]	0.37	5.1E-234	1.2E-232	0.42	yes
K01170	tRNA-intron endonuclease, archaea type [EC:4.6.1.16]	0.37	2.1E-233	4.7E-232	0.42	yes
K01236	maltooligosyltrehalose trehalohydrolase [EC:3.2.1.141]	0.37	3.3E-233	7.4E-232	0.39	yes
K00114	alcohol dehydrogenase (cytochrome c) [EC:1.1.2.8]	0.37	4.4E-233	9.8E-232	0.42	yes
K11131	H/ACA ribonucleoprotein complex subunit 4 [EC:5.4.99.-]	0.37	4.6E-233	1.0E-231	0.42	yes
K03042	DNA-directed RNA polymerase subunit A\ [EC:2.7.7.6]	0.37	4.5E-232	9.8E-231	0.42	yes
K02323	DNA polymerase II small subunit [EC:2.7.7.7]	0.37	5.0E-232	1.1E-230	0.41	yes
K02276	cytochrome c oxidase subunit III [EC:7.1.1.9]	0.37	5.6E-232	1.2E-230	0.40	yes
K03921	acyl-[acyl-carrier-protein] desaturase [EC:1.14.19.2 1.14.19.11 1.14.19.26]	0.37	7.6E-232	1.6E-230	0.39	yes
K07130	arylformamidase [EC:3.5.1.9]	0.37	2.2E-231	4.7E-230	0.40	yes
K11782	chorismate dehydratase [EC:4.2.1.151]	0.37	5.6E-231	1.2E-229	0.40	yes
K00808	homospermidine synthase [EC:2.5.1.44]	0.37	6.1E-231	1.3E-229	0.40	yes
K14162	error-prone DNA polymerase [EC:2.7.7.7]	0.37	9.4E-231	2.0E-229	0.39	yes
K07302	isoquinoline 1-oxidoreductase subunit alpha [EC:1.3.99.16]	0.37	4.8E-230	9.9E-229	0.39	yes
K07303	isoquinoline 1-oxidoreductase subunit beta [EC:1.3.99.16]	0.37	8.4E-229	1.7E-227	0.39	yes
K00443	coenzyme F420 hydrogenase subunit gamma [EC:1.12.98.1]	0.37	1.8E-228	3.7E-227	0.42	yes
K03053	DNA-directed RNA polymerase subunit H [EC:2.7.7.6]	0.37	2.5E-228	5.1E-227	0.41	yes
K00500	phenylalanine-4-hydroxylase [EC:1.14.16.1]	0.37	4.5E-228	9.1E-227	0.40	yes
K00401	methyl-coenzyme M reductase beta subunit [EC:2.8.4.1]	0.37	6.4E-228	1.3E-226	0.41	yes
K00203	formylmethanofuran dehydrogenase subunit D [EC:1.2.7.12]	0.37	9.7E-228	1.9E-226	0.41	yes
K00958	sulfate adenylyltransferase [EC:2.7.7.4]	0.37	1.5E-227	3.0E-226	0.40	yes
K03059	DNA-directed RNA polymerase subunit P [EC:2.7.7.6]	0.37	1.8E-227	3.5E-226	0.42	yes
K16188	tetraprenyl-beta-curcumene synthase [EC:4.2.3.130]	0.37	2.6E-227	5.1E-226	0.40	yes
K10978	methanogen homoisocitrate dehydrogenase [EC:1.1.1.87 1.1.1.-]	0.37	7.2E-227	1.4E-225	0.41	yes
K00440	coenzyme F420 hydrogenase subunit alpha [EC:1.12.98.1]	0.37	8.0E-227	1.5E-225	0.41	yes
K00578	tetrahydromethanopterin S-methyltransferase subunit B [EC:2.1.1.86]	0.37	9.7E-227	1.9E-225	0.41	yes
K00579	tetrahydromethanopterin S-methyltransferase subunit C [EC:2.1.1.86]	0.37	1.1E-226	2.1E-225	0.41	yes
K03055	DNA-directed RNA polymerase subunit K [EC:2.7.7.6]	0.37	1.1E-226	2.1E-225	0.41	
K02588	nitrogenase iron protein NifH	0.37	1.2E-226	2.2E-225	0.41	
K14127	F420-non-reducing hydrogenase iron-sulfur subunit [EC:1.12.99.- 1.8.98.5 1.8.98.6]	0.37	1.5E-226	2.8E-225	0.41	yes
K13531	methylated-DNA-[protein]-cysteine S-methyltransferase [EC:2.1.1.63]	0.37	1.5E-226	2.8E-225	0.43	yes
K00399	methyl-coenzyme M reductase alpha subunit [EC:2.8.4.1]	0.37	2.8E-226	5.2E-225	0.41	yes

Predictor	Description	Pearson's r	P	FDR-adjusted P	Spearman's rho	Associated with fractures (P<0.05)
K00392	sulfite reductase (ferredoxin) [EC:1.8.7.1]	0.37	9.6E-226	1.8E-224	0.39	yes
K00531	nitrogenase delta subunit [EC:1.18.6.1]	0.37	1.8E-225	3.3E-224	0.38	yes
K06044	(1->4)-alpha-D-glucan 1-alpha-D-glucosylmutase [EC:5.4.99.15]	0.37	7.1E-225	1.3E-223	0.38	yes
K05342	alpha,alpha-trehalose phosphorylase [EC:2.4.1.64]	0.37	4.0E-224	7.2E-223	0.39	yes
K14519	NADP-dependent aldehyde dehydrogenase [EC:1.2.1.4]	0.37	1.3E-223	2.3E-222	0.38	yes
K13015	UDP-N-acetyl-D-glucosamine dehydrogenase [EC:1.1.1.136]	0.37	1.6E-223	2.8E-222	0.39	yes
K15888	tritrans,polycis-undecaprenyl-diphosphate synthase [geranylgeranyl-diphosphate specific] [EC:2.5.1.89]	0.37	1.7E-223	3.0E-222	0.41	yes
K03392	aminocarboxymuconate-semialdehyde decarboxylase [EC:4.1.1.45]	0.37	2.1E-223	3.7E-222	0.40	yes
K09722	4-phosphopantoate---beta-alanine ligase [EC:6.3.2.36]	0.37	2.1E-223	3.7E-222	0.41	yes
K00200	formylmethanofuran dehydrogenase subunit A [EC:1.2.7.12]	0.37	3.3E-223	5.7E-222	0.41	yes
K03044	DNA-directed RNA polymerase subunit B' [EC:2.7.7.6]	0.37	5.7E-223	9.8E-222	0.41	yes
K13057	trehalose synthase [EC:2.4.1.245]	0.37	7.1E-223	1.2E-221	0.40	yes
K00696	sucrose-phosphate synthase [EC:2.4.1.14]	0.37	8.2E-223	1.4E-221	0.40	yes
K00318	proline dehydrogenase [EC:1.5.5.2]	0.37	1.3E-222	2.2E-221	0.39	yes
K00368	nitrite reductase (NO-forming) [EC:1.7.2.1]	0.37	2.5E-222	4.2E-221	0.38	yes
K01476	arginase [EC:3.5.3.1]	0.37	2.6E-222	4.4E-221	0.40	yes
K00583	tetrahydromethanopterin S-methyltransferase subunit G [EC:2.1.1.86]	0.37	3.7E-222	6.2E-221	0.41	yes
K00319	methylenetetrahydromethanopterin dehydrogenase [EC:1.5.98.1]	0.37	5.6E-222	9.3E-221	0.41	yes
K01820	L-rhamnose isomerase / sugar isomerase [EC:5.3.1.14 5.3.1.-]	0.37	1.9E-221	3.1E-220	0.39	yes
K01576	benzoylformate decarboxylase [EC:4.1.1.7]	0.36	9.2E-221	1.5E-219	0.39	yes
K00411	ubiquinol-cytochrome c reductase iron-sulfur subunit [EC:7.1.1.8]	0.36	2.9E-220	4.7E-219	0.39	yes
K13853	3-deoxy-7-phosphoheptulonate synthase / chorismate mutase [EC:2.5.1.54 5.4.99.5]	0.36	1.3E-219	2.1E-218	0.40	yes
K03333	cholesterol oxidase [EC:1.1.3.6]	0.36	1.6E-219	2.6E-218	0.38	yes
K03045	DNA-directed RNA polymerase subunit B\ [EC:2.7.7.6]	0.36	2.2E-219	3.5E-218	0.41	yes
K00685	arginyl-tRNA---protein transferase [EC:2.3.2.8]	0.36	3.6E-219	5.7E-218	0.39	yes
K00402	methyl-coenzyme M reductase gamma subunit [EC:2.8.4.1]	0.36	4.3E-219	6.8E-218	0.41	yes
K00459	nitronate monooxygenase [EC:1.13.12.16]	0.36	1.2E-218	1.9E-217	0.39	yes
K12073	1,4-dihydroxy-2-naphthoyl-CoA hydrolase [EC:3.1.2.28]	0.36	1.3E-218	2.0E-217	0.43	
K16011	mannose-1-phosphate guanylyltransferase / mannose-6-phosphate isomerase [EC:2.7.7.13 5.3.1.8]	0.36	1.3E-218	2.0E-217	0.39	yes
K00686	protein-glutamine gamma-glutamyltransferase [EC:2.3.2.13]	0.36	3.7E-218	5.8E-217	0.38	yes
K10844	DNA excision repair protein ERCC-2 [EC:3.6.4.12]	0.36	8.2E-218	1.3E-216	0.42	yes
K00311	electron-transferring-flavoprotein dehydrogenase [EC:1.5.5.1]	0.36	5.3E-217	8.2E-216	0.39	yes
K00533	ferredoxin hydrogenase large subunit [EC:1.12.7.2]	0.36	1.1E-216	1.7E-215	0.39	yes
K06863	5-formaminoimidazole-4-carboxamide-1-(beta)-D-ribofuranosyl 5'-monophosphate synthetase [EC:6.3.4.23]	0.36	2.5E-216	3.8E-215	0.41	
K13812	bifunctional enzyme Fae/Hps [EC:4.2.1.147 4.1.2.43]	0.36	3.1E-216	4.7E-215	0.40	yes
K00232	acyl-CoA oxidase [EC:1.3.3.6]	0.36	3.4E-216	5.1E-215	0.38	yes
K02201	pantetheine-phosphate adenylyltransferase [EC:2.7.7.3]	0.36	5.6E-216	8.4E-215	0.41	yes
K15746	beta-carotene 3-hydroxylase [EC:1.14.15.24]	0.36	5.6E-216	8.4E-215	0.39	yes
K11434	type I protein arginine methyltransferase [EC:2.1.1.319]	0.36	6.6E-216	9.8E-215	0.41	
K00167	2-oxoisovalerate dehydrogenase E1 component beta subunit [EC:1.2.4.4]	0.36	1.0E-215	1.5E-214	0.39	yes
K01053	gluconolactonase [EC:3.1.1.17]	0.36	1.3E-215	1.9E-214	0.38	yes
K00453	tryptophan 2,3-dioxygenase [EC:1.13.11.11]	0.36	2.6E-215	3.8E-214	0.39	yes

Predictor	Description	Pearson's r	P	FDR-adjusted P	Spearman's rho	Associated with fractures (P<0.05)
K13598	two-component system, NtrC family, nitrogen regulation sensor histidine kinase NtrY [EC:2.7.13.3]	0.36	4.0E-215	5.8E-214	0.38	yes
K00451	homogentisate 1,2-dioxygenase [EC:1.13.11.5]	0.36	5.3E-215	7.7E-214	0.38	yes
K11176	IMP cyclohydrolase [EC:3.5.4.10]	0.36	6.5E-215	9.4E-214	0.41	yes
K00580	tetrahydromethanopterin S-methyltransferase subunit D [EC:2.1.1.86]	0.36	8.2E-215	1.2E-213	0.40	yes
K05352	ribitol-5-phosphate 2-dehydrogenase (NADP+) [EC:1.1.1.405]	0.36	9.3E-215	1.3E-213	0.39	yes
K04719	5,6-dimethylbenzimidazole synthase [EC:1.13.11.79]	0.36	1.1E-214	1.6E-213	0.38	yes
K11942	isobutyryl-CoA mutase [EC:5.4.99.13]	0.36	1.3E-214	1.8E-213	0.39	yes
K01387	microbial collagenase [EC:3.4.24.3]	0.36	3.2E-214	4.5E-213	0.39	yes
K05797	4-cresol dehydrogenase (hydroxylating) flavoprotein subunit [EC:1.17.9.1]	0.36	5.0E-214	7.0E-213	0.42	
K03405	magnesium chelatase subunit I [EC:6.6.1.1]	0.36	5.2E-214	7.3E-213	0.40	yes
K11614	two-component system, CitB family, sensor histidine kinase MalK [EC:2.7.13.3]	0.36	6.5E-214	9.0E-213	0.38	yes
K00015	glyoxylate reductase [EC:1.1.1.26]	0.36	1.5E-213	2.1E-212	0.40	yes
K13039	sulfopyruvate decarboxylase subunit beta [EC:4.1.1.79]	0.36	2.0E-213	2.8E-212	0.40	yes
K00836	diaminobutyrate-2-oxoglutarate transaminase [EC:2.6.1.76]	0.36	4.2E-213	5.8E-212	0.38	yes
K10977	methanogen homocitrate synthase [EC:2.3.3.14 2.3.3.-]	0.36	1.1E-212	1.5E-211	0.40	yes
K01113	alkaline phosphatase D [EC:3.1.3.1]	0.36	1.2E-212	1.6E-211	0.38	yes
K10764	O-succinylhomoserine sulfhydrylase [EC:2.5.1.-]	0.36	3.5E-212	4.7E-211	0.38	yes
K07516	3-hydroxyacyl-CoA dehydrogenase [EC:1.1.1.35]	0.36	6.7E-212	9.0E-211	0.39	yes
K13766	methylglutaconyl-CoA hydratase [EC:4.2.1.18]	0.36	1.0E-211	1.3E-210	0.38	yes
K00619	amino-acid N-acetyltransferase [EC:2.3.1.1]	0.36	1.6E-211	2.1E-210	0.38	yes
K10255	acyl-lipid omega-6 desaturase (Delta-12 desaturase) [EC:1.14.19.23 1.14.19.45]	0.36	3.8E-211	5.1E-210	0.38	yes
K00209	enoyl-[acyl-carrier protein] reductase / trans-2-enoyl-CoA reductase (NAD+) [EC:1.3.1.9 1.3.1.44]	0.36	9.0E-211	1.2E-209	0.38	
K00992	N-acetyl-alpha-D-muramate 1-phosphate uridylyltransferase [EC:2.7.7.99]	0.36	2.2E-210	2.9E-209	0.38	yes
K07469	aldehyde oxidoreductase [EC:1.2.99.7]	0.36	2.5E-210	3.3E-209	0.38	yes
K01555	fumarylacetoacetase [EC:3.7.1.2]	0.36	2.9E-210	3.8E-209	0.38	yes
K00126	formate dehydrogenase subunit delta [EC:1.17.1.9]	0.36	2.9E-210	3.8E-209	0.38	yes
K11783	futalosine hydrolase [EC:3.2.2.26]	0.36	7.6E-210	9.8E-209	0.38	yes
K01431	beta-ureidopropionase [EC:3.5.1.6]	0.36	1.8E-209	2.3E-208	0.38	yes
K00019	3-hydroxybutyrate dehydrogenase [EC:1.1.1.30]	0.36	4.0E-209	5.1E-208	0.38	yes
K03519	aerobic carbon-monoxide dehydrogenase medium subunit [EC:1.2.5.3]	0.36	7.5E-209	9.6E-208	0.38	yes
K06121	glycerol dehydratase medium subunit [EC:4.2.1.30]	0.36	7.6E-209	9.7E-208	0.42	yes
K00205	4Fe-4S ferredoxin	0.36	1.3E-208	1.6E-207	0.40	yes
K04034	anaerobic magnesium-protoporphyrin IX monomethyl ester cyclase [EC:1.21.98.3]	0.35	7.8E-208	9.8E-207	0.39	yes
K01457	allophanate hydrolase [EC:3.5.1.54]	0.35	1.1E-207	1.4E-206	0.38	yes
K00491	nitric-oxide synthase, bacterial [EC:1.14.14.47]	0.35	2.4E-206	3.0E-205	0.38	yes
K03399	cobalt-precorrin-7 (C5)-methyltransferase [EC:2.1.1.289]	0.35	8.3E-205	1.0E-203	0.38	
K15358	enamidase [EC:3.5.2.18]	0.35	2.5E-204	3.1E-203	0.38	yes
K10713	5,6,7,8-tetrahydromethanopterin hydro-lyase [EC:4.2.1.147]	0.35	3.0E-204	3.7E-203	0.39	
K01178	glucoamylase [EC:3.2.1.3]	0.35	4.9E-204	6.0E-203	0.39	yes
K15894	UDP-N-acetylglucosamine 4,6-dehydratase/5-epimerase [EC:4.2.1.115 5.1.3.-]	0.35	5.3E-204	6.5E-203	0.41	yes
K06137	pyrroloquinoline-quinone synthase [EC:1.3.3.11]	0.35	6.8E-204	8.3E-203	0.38	yes
K02191	cobalt-precorrin-6B (C15)-methyltransferase [EC:2.1.1.196]	0.35	1.6E-203	1.9E-202	0.38	

Predictor	Description	Pearson's r	P	FDR-adjusted P	Spearman's rho	Associated with fractures (P<0.05)
K09699	2-oxoisovalerate dehydrogenase E2 component (dihydrolipoyl transacylase) [EC:2.3.1.168]	0.35	1.8E-203	2.2E-202	0.38	yes
K00457	4-hydroxyphenylpyruvate dioxygenase [EC:1.13.11.27]	0.35	3.2E-203	3.9E-202	0.38	yes
K01029	3-oxoacid CoA-transferase subunit B [EC:2.8.3.5]	0.35	4.1E-203	4.9E-202	0.37	yes
K15067	2-aminomuconate deaminase [EC:3.5.99.5]	0.35	2.4E-202	2.9E-201	0.39	yes
K00166	2-oxoisovalerate dehydrogenase E1 component alpha subunit [EC:1.2.4.4]	0.35	4.1E-202	4.9E-201	0.38	yes
K11180	dissimilatory sulfite reductase alpha subunit [EC:1.8.99.5]	0.35	5.4E-202	6.4E-201	0.37	yes
K00002	alcohol dehydrogenase (NADP+) [EC:1.1.1.2]	0.35	6.5E-202	7.7E-201	0.42	yes
K00508	linoleoyl-CoA desaturase [EC:1.14.19.3]	0.35	2.3E-201	2.7E-200	0.37	yes
K03725	archaea-specific helicase [EC:3.6.4.-]	0.35	3.6E-201	4.2E-200	0.39	yes
K00822	beta-alanine--pyruvate transaminase [EC:2.6.1.18]	0.35	1.7E-200	2.0E-199	0.37	yes
K06134	3-demethoxyubiquinol 3-hydroxylase [EC:1.14.99.60]	0.35	2.9E-200	3.4E-199	0.38	yes
K10217	aminomuconate-semialdehyde/2-hydroxymuconate-6-semialdehyde dehydrogenase [EC:1.2.1.32 1.2.1.85]	0.35	8.2E-200	9.5E-199	0.38	yes
K13924	two-component system, chemotaxis family, CheB/CheR fusion protein [EC:2.1.1.80 3.1.1.61]	0.35	1.2E-199	1.4E-198	0.37	yes
K06034	sulfopyruvate decarboxylase subunit alpha [EC:4.1.1.79]	0.35	1.7E-199	2.0E-198	0.39	yes
K00856	adenosine kinase [EC:2.7.1.20]	0.35	3.6E-199	4.1E-198	0.37	yes
K03652	DNA-3-methyladenine glycosylase [EC:3.2.2.21]	0.35	1.6E-198	1.8E-197	0.37	yes
K03821	poly[(R)-3-hydroxyalkanoate] polymerase subunit PhaC [EC:2.3.1.304]	0.35	2.8E-198	3.2E-197	0.38	yes
K01283	peptidyl-dipeptidase A [EC:3.4.15.1]	0.35	3.3E-198	3.7E-197	0.38	yes
K01473	N-methylhydantoinase A [EC:3.5.2.14]	0.35	6.9E-198	7.8E-197	0.37	yes
K01474	N-methylhydantoinase B [EC:3.5.2.14]	0.35	9.0E-198	1.0E-196	0.37	yes
K01505	1-aminocyclopropane-1-carboxylate deaminase [EC:3.5.99.7]	0.35	2.1E-197	2.4E-196	0.37	yes
K11260	4Fe-4S ferredoxin	0.35	2.3E-197	2.6E-196	0.39	yes
K01535	H+-transporting ATPase [EC:7.1.2.1]	0.35	4.8E-197	5.3E-196	0.38	yes
K03653	N-glycosylase/DNA lyase [EC:3.2.2.- 4.2.99.18]	0.35	3.3E-196	3.7E-195	0.41	yes
K15629	fatty-acid peroxygenase [EC:1.11.2.4]	0.34	5.7E-196	6.3E-195	0.37	yes
K08096	GTP cyclohydrolase IIa [EC:3.5.4.29]	0.34	1.5E-195	1.6E-194	0.39	
K02482	two-component system, NtrC family, sensor kinase [EC:2.7.13.3]	0.34	1.5E-195	1.6E-194	0.37	yes
K03153	glycine oxidase [EC:1.4.3.19]	0.34	2.8E-195	3.1E-194	0.37	yes
K15371	glutamate dehydrogenase [EC:1.4.1.2]	0.34	3.4E-195	3.7E-194	0.37	yes
K15011	two-component system, sensor histidine kinase RegB [EC:2.7.13.3]	0.34	4.1E-195	4.4E-194	0.37	yes
K01028	3-oxoacid CoA-transferase subunit A [EC:2.8.3.5]	0.34	4.7E-195	5.1E-194	0.37	yes
K07717	two-component system, sensor histidine kinase GlnK [EC:2.7.13.3]	0.34	7.9E-195	8.5E-194	0.37	yes
K03793	pteridine reductase [EC:1.5.1.33]	0.34	3.2E-194	3.4E-193	0.37	yes
K03520	aerobic carbon-monoxide dehydrogenase large subunit [EC:1.2.5.3]	0.34	4.1E-194	4.4E-193	0.37	yes
K10843	DNA excision repair protein ERCC-3 [EC:3.6.4.12]	0.34	6.3E-194	6.7E-193	0.36	yes
K00436	NAD-reducing hydrogenase large subunit [EC:1.12.1.2]	0.34	3.5E-193	3.7E-192	0.37	yes
K01400	bacillolysin [EC:3.4.24.28]	0.34	3.6E-193	3.8E-192	0.37	yes
K00376	nitrous-oxide reductase [EC:1.7.2.4]	0.34	4.1E-193	4.3E-192	0.37	yes
K05922	quinone-reactive Ni/Fe-hydrogenase large subunit [EC:1.12.5.1]	0.34	4.7E-193	4.9E-192	0.40	yes
K00188	2-oxoisovalerate ferredoxin oxidoreductase delta subunit [EC:1.2.7.7]	0.34	1.0E-192	1.0E-191	0.39	
K02476	two-component system, CitB family, sensor kinase [EC:2.7.13.3]	0.34	1.5E-192	1.6E-191	0.38	yes
K00252	glutaryl-CoA dehydrogenase [EC:1.3.8.6]	0.34	1.6E-192	1.7E-191	0.37	yes

Predictor	Description	Pearson's r	P	FDR-adjusted P	Spearman's rho	Associated with fractures (P<0.05)
K01728	pectate lyase [EC:4.2.2.2]	0.34	3.4E-192	3.5E-191	0.36	yes
K00148	glutathione-independent formaldehyde dehydrogenase [EC:1.2.1.46]	0.34	3.8E-192	3.9E-191	0.37	yes
K00038	3alpha(or 20beta)-hydroxysteroid dehydrogenase [EC:1.1.1.53]	0.34	9.0E-192	9.3E-191	0.37	yes
K00249	acyl-CoA dehydrogenase [EC:1.3.8.7]	0.34	2.0E-191	2.1E-190	0.37	yes
K01083	3-phytase [EC:3.1.3.8]	0.34	2.1E-191	2.1E-190	0.37	yes
K02122	V/A-type H+/Na+-transporting ATPase subunit F	0.34	7.1E-191	7.2E-190	0.37	
K01266	D-aminopeptidase [EC:3.4.11.19]	0.34	7.6E-191	7.7E-190	0.37	yes
K01799	maleate isomerase [EC:5.2.1.1]	0.34	1.1E-190	1.1E-189	0.37	
K05895	precorrin-6A/cobalt-precorrin-6A reductase [EC:1.3.1.54 1.3.1.106]	0.34	1.3E-190	1.3E-189	0.37	
K12256	putrescine---pyruvate transaminase [EC:2.6.1.113]	0.34	1.4E-190	1.4E-189	0.37	yes
K15519	deoxyadenosine/deoxycytidine kinase [EC:2.7.1.76 2.7.1.74]	0.34	2.2E-190	2.2E-189	0.41	yes
K00122	formate dehydrogenase [EC:1.17.1.9]	0.34	2.3E-190	2.3E-189	0.37	yes
K13481	xanthine dehydrogenase small subunit [EC:1.17.1.4]	0.34	1.4E-189	1.4E-188	0.37	yes
K00582	tetrahydromethanopterin S-methyltransferase subunit F [EC:2.1.1.86]	0.34	6.3E-189	6.3E-188	0.38	yes
K04343	streptomycin 6-kinase [EC:2.7.1.72]	0.34	8.4E-189	8.3E-188	0.37	yes
K00481	p-hydroxybenzoate 3-monooxygenase [EC:1.14.13.2]	0.34	5.7E-188	5.6E-187	0.37	yes
K00102	D-lactate dehydrogenase (cytochrome) [EC:1.1.2.4]	0.34	2.5E-187	2.5E-186	0.36	yes
K01746	formiminotetrahydrofolate cyclodeaminase [EC:4.3.1.4]	0.34	4.6E-187	4.5E-186	0.36	yes
K13485	2-oxo-4-hydroxy-4-carboxy-5-ureidoimidazoline decarboxylase [EC:4.1.1.97]	0.34	6.2E-187	6.1E-186	0.39	yes
K00197	acetyl-CoA decarbonylase/synthase, CODH/ACS complex subunit gamma [EC:2.1.1.245]	0.34	8.8E-187	8.6E-186	0.37	yes
K02293	15-cis-phytoene desaturase [EC:1.3.5.5]	0.34	3.1E-186	3.0E-185	0.39	
K07583	tRNA pseudouridine synthase 10 [EC:5.4.99.25]	0.34	3.3E-186	3.2E-185	0.41	yes
K03167	DNA topoisomerase VI subunit B [EC:5.6.2.2]	0.34	4.6E-186	4.4E-185	0.40	yes
K00446	catechol 2,3-dioxygenase [EC:1.13.11.2]	0.34	4.7E-186	4.5E-185	0.36	yes
K01965	propionyl-CoA carboxylase alpha chain [EC:6.4.1.3]	0.34	8.8E-186	8.4E-185	0.36	yes
K08646	peptidyl-Lys metalloendopeptidase [EC:3.4.24.20]	0.34	9.7E-186	9.3E-185	0.39	yes
K05927	quinone-reactive Ni/Fe-hydrogenase small subunit [EC:1.12.5.1]	0.34	2.8E-185	2.7E-184	0.41	yes
K00194	acetyl-CoA decarbonylase/synthase, CODH/ACS complex subunit delta [EC:2.1.1.245]	0.34	3.7E-185	3.5E-184	0.37	yes
K01166	ribonuclease T2 [EC:4.6.1.19]	0.34	6.1E-185	5.8E-184	0.37	yes
K13482	xanthine dehydrogenase large subunit [EC:1.17.1.4]	0.34	7.3E-185	6.9E-184	0.37	yes
K01055	3-oxoadipate enol-lactonase [EC:3.1.1.24]	0.34	1.6E-184	1.5E-183	0.37	yes
K01301	N-acetylated-alpha-linked acidic dipeptidase [EC:3.4.17.21]	0.34	2.5E-184	2.3E-183	0.38	yes
K13006	UDP-perosamine 4-acetyltransferase [EC:2.3.1.-]	0.33	7.6E-184	7.1E-183	0.41	yes
K00523	CDP-4-dehydro-6-deoxyglucose reductase, E3 [EC:1.17.1.1]	0.33	7.6E-184	7.1E-183	0.36	yes
K01455	formamidase [EC:3.5.1.49]	0.33	8.0E-184	7.4E-183	0.36	yes
K13693	glucosyl-3-phosphoglycerate synthase [EC:2.4.1.266]	0.33	1.1E-183	1.0E-182	0.35	yes
K14267	N-succinyldiaminopimelate aminotransferase [EC:2.6.1.17]	0.33	1.2E-183	1.1E-182	0.36	yes
K01133	choline-sulfatase [EC:3.1.6.6]	0.33	1.8E-183	1.7E-182	0.37	yes
K10562	rhamnose transport system ATP-binding protein [EC:7.5.2.-]	0.33	1.9E-183	1.7E-182	0.36	yes
K00569	thiopurine S-methyltransferase [EC:2.1.1.67]	0.33	2.1E-183	1.9E-182	0.36	yes
K00091	dihydroflavonol-4-reductase [EC:1.1.1.219]	0.33	3.5E-183	3.2E-182	0.36	yes
K02636	cytochrome b6-f complex iron-sulfur subunit [EC:7.1.1.6]	0.33	4.1E-183	3.7E-182	0.36	yes

Predictor	Description	Pearson's r	P	FDR-adjusted P	Spearman's rho	Associated with fractures (P<0.05)
K06446	acyl-CoA dehydrogenase [EC:1.3.99.-]	0.33	5.7E-183	5.2E-182	0.36	yes
K00365	urate oxidase [EC:1.7.3.3]	0.33	9.0E-183	8.1E-182	0.37	yes
K10960	geranylgeranyl diphosphate/geranylgeranyl-bacteriochlorophyllide a reductase [EC:1.3.1.83 1.3.1.111]	0.33	1.2E-182	1.1E-181	0.37	yes
K00066	GDP-mannose 6-dehydrogenase [EC:1.1.1.132]	0.33	1.6E-182	1.4E-181	0.36	yes
K05827	[lysine-biosynthesis-protein LysW]---L-2-aminoadipate ligase [EC:6.3.2.43]	0.33	2.6E-182	2.3E-181	0.37	yes
K06169	tRNA 2-(methylsulfanyl)-N6-isopentenyladenosine37 hydroxylase [EC:1.14.99.69]	0.33	4.7E-182	4.2E-181	0.36	
K08077	UDP-sugar diphosphatase [EC:3.6.1.45]	0.33	5.2E-182	4.6E-181	0.42	yes
K01969	3-methylcrotonyl-CoA carboxylase beta subunit [EC:6.4.1.4]	0.33	6.2E-182	5.5E-181	0.36	yes
K05884	L-2-hydroxycarboxylate dehydrogenase (NAD+) [EC:1.1.1.337]	0.33	6.5E-182	5.8E-181	0.37	yes
K05299	formate dehydrogenase (NADP+) alpha subunit [EC:1.17.1.10]	0.33	7.1E-182	6.3E-181	0.36	yes
K16171	fumarylacetoacetate (FAA) hydrolase [EC:3.7.1.2]	0.33	1.8E-181	1.6E-180	0.36	yes
K00008	L-iditol 2-dehydrogenase [EC:1.1.1.14]	0.33	1.9E-181	1.7E-180	0.36	yes
K04766	acetoin utilization protein AcuA [EC:2.3.1.-]	0.33	5.6E-181	4.9E-180	0.36	yes
K00830	alanine-glyoxylate transaminase / serine-glyoxylate transaminase / serine-pyruvate transaminase [EC:2.6.1.44 2.6.1.45 2.6.1.51]	0.33	8.3E-181	7.2E-180	0.36	yes
K00023	acetoacetyl-CoA reductase [EC:1.1.1.36]	0.33	1.0E-180	8.7E-180	0.36	yes
K10218	4-hydroxy-4-methyl-2-oxoglutarate aldolase [EC:4.1.3.17]	0.33	1.3E-180	1.1E-179	0.36	yes
K01001	UDP-N-acetylglucosamine--dolichyl-phosphate N-acetylglucosaminephosphotransferase [EC:2.7.8.15]	0.33	2.6E-180	2.2E-179	0.38	yes
K00171	pyruvate ferredoxin oxidoreductase delta subunit [EC:1.2.7.1]	0.33	1.0E-179	8.6E-179	0.35	yes
K01602	ribulose-bisphosphate carboxylase small chain [EC:4.1.1.39]	0.33	1.8E-179	1.5E-178	0.35	yes
K02229	precorrin-3B synthase [EC:1.14.13.83]	0.33	4.7E-179	4.0E-178	0.36	yes
K01777	proline racemase [EC:5.1.1.4]	0.33	4.8E-179	4.1E-178	0.35	yes
K13017	UDP-2-acetamido-2-deoxy-ribo-hexuluronate aminotransferase [EC:2.6.1.98]	0.33	1.1E-178	9.4E-178	0.36	yes
K00643	5-aminolevulinate synthase [EC:2.3.1.37]	0.33	2.5E-178	2.1E-177	0.36	yes
K00448	protocatechuate 3,4-dioxygenase, alpha subunit [EC:1.13.11.3]	0.33	2.8E-178	2.4E-177	0.35	yes
K04090	indolepyruvate ferredoxin oxidoreductase [EC:1.2.7.8]	0.33	3.1E-178	2.6E-177	0.36	yes
K15518	deoxyguanosine kinase [EC:2.7.1.113]	0.33	8.4E-178	7.1E-177	0.38	yes
K11779	FO synthase [EC:2.5.1.147 4.3.1.32]	0.33	2.9E-177	2.4E-176	0.37	yes
K14138	acetyl-CoA synthase [EC:2.3.1.169]	0.33	4.9E-177	4.1E-176	0.36	yes
K07116	acyl-homoserine-lactone acylase [EC:3.5.1.97]	0.33	5.9E-177	4.9E-176	0.35	yes
K11623	two-component system, NarL family, sensor histidine kinase YdfH [EC:2.7.13.3]	0.33	8.5E-177	7.1E-176	0.40	yes
K14653	2-amino-5-formylamino-6-ribosylaminopyrimidin-4(3H)-one 5'-monophosphate deformylase [EC:3.5.1.102]	0.33	9.0E-177	7.5E-176	0.37	yes
K13075	N-acyl homoserine lactone hydrolase [EC:3.1.1.81]	0.33	2.9E-176	2.4E-175	0.35	yes
K00153	S-(hydroxymethyl)mycothiol dehydrogenase [EC:1.1.1.306]	0.33	3.9E-176	3.2E-175	0.35	yes
K03464	muconolactone D-isomerase [EC:5.3.3.4]	0.33	5.0E-176	4.1E-175	0.35	
K00469	inositol oxygenase [EC:1.13.99.1]	0.33	6.4E-176	5.3E-175	0.39	yes
K09883	cobaltochelatase CobT [EC:6.6.1.2]	0.33	7.8E-176	6.4E-175	0.37	yes
K00253	isovaleryl-CoA dehydrogenase [EC:1.3.8.4]	0.33	8.5E-176	6.9E-175	0.36	yes
K02228	precorrin-6A synthase [EC:2.1.1.152]	0.33	8.5E-175	6.9E-174	0.35	yes
K01444	N4-(beta-N-acetylglucosaminyl)-L-asparaginase [EC:3.5.1.26]	0.33	1.8E-174	1.5E-173	0.35	yes
K01907	acetoacetyl-CoA synthetase [EC:6.2.1.16]	0.33	2.1E-174	1.7E-173	0.35	yes
K00086	1,3-propanediol dehydrogenase [EC:1.1.1.202]	0.33	2.3E-174	1.9E-173	0.37	
K13950	para-aminobenzoate synthetase [EC:2.6.1.85]	0.33	3.2E-174	2.6E-173	0.35	yes

Predictor	Description	Pearson's r	P	FDR-adjusted P	Spearman's rho	Associated with fractures (P<0.05)
K01968	3-methylcrotonyl-CoA carboxylase alpha subunit [EC:6.4.1.4]	0.33	5.0E-174	4.0E-173	0.36	yes
K15510	coenzyme F420-dependent glucose-6-phosphate dehydrogenase [EC:1.1.98.2]	0.33	5.5E-174	4.4E-173	0.37	yes
K07653	two-component system, OmpR family, sensor histidine kinase MprB [EC:2.7.13.3]	0.33	5.7E-174	4.6E-173	0.35	yes
K00141	benzaldehyde dehydrogenase (NAD) [EC:1.2.1.28]	0.33	2.1E-173	1.7E-172	0.36	yes
K12979	beta-hydroxylase [EC:1.14.11.-]	0.33	2.4E-173	1.9E-172	0.35	yes
K01707	5-dehydro-4-deoxyglucarate dehydratase [EC:4.2.1.41]	0.33	4.8E-173	3.8E-172	0.35	yes
K00499	choline monooxygenase [EC:1.14.15.7]	0.32	1.7E-172	1.3E-171	0.39	yes
K04039	light-independent protochlorophyllide reductase subunit B [EC:1.3.7.7]	0.32	5.0E-172	3.9E-171	0.35	yes
K02480	two-component system, NarL family, sensor kinase [EC:2.7.13.3]	0.32	5.3E-172	4.2E-171	0.35	yes
K05586	bidirectional [NiFe] hydrogenase diaphorase subunit [EC:7.1.1.2]	0.32	8.1E-172	6.3E-171	0.37	yes
K05588	bidirectional [NiFe] hydrogenase diaphorase subunit [EC:7.1.1.2]	0.32	8.1E-172	6.3E-171	0.37	yes
K01004	phosphatidylcholine synthase [EC:2.7.8.24]	0.32	1.4E-171	1.1E-170	0.36	yes
K00394	adenylylsulfate reductase, subunit A [EC:1.8.99.2]	0.32	2.1E-171	1.6E-170	0.39	
K03428	magnesium-protoporphyrin O-methyltransferase [EC:2.1.1.11]	0.32	2.5E-171	1.9E-170	0.35	yes
K06120	glycerol dehydratase large subunit [EC:4.2.1.30]	0.32	5.5E-171	4.3E-170	0.37	
K00395	adenylylsulfate reductase, subunit B [EC:1.8.99.2]	0.32	6.2E-171	4.8E-170	0.39	
K04038	light-independent protochlorophyllide reductase subunit N [EC:1.3.7.7]	0.32	1.8E-170	1.4E-169	0.35	yes
K12503	short-chain Z-isoprenyl diphosphate synthase [EC:2.5.1.68]	0.32	2.4E-170	1.8E-169	0.34	yes
K10670	glycine/sarcosine/betaine reductase complex component A [EC:1.21.4.2 1.21.4.3 1.21.4.4]	0.32	2.9E-170	2.2E-169	0.34	yes
K00510	heme oxygenase 1 [EC:1.14.14.18]	0.32	3.0E-170	2.3E-169	0.34	yes
K03049	DNA-directed RNA polymerase subunit E' [EC:2.7.7.6]	0.32	3.6E-170	2.7E-169	0.39	yes
K04037	light-independent protochlorophyllide reductase subunit L [EC:1.3.7.7]	0.32	1.1E-169	8.4E-169	0.35	yes
K11731	citronellyl-CoA dehydrogenase [EC:1.3.99.-]	0.32	1.4E-169	1.1E-168	0.36	yes
K11532	fructose-1,6-bisphosphatase II / sedoheptulose-1,7-bisphosphatase [EC:3.1.3.11 3.1.3.37]	0.32	1.5E-169	1.1E-168	0.37	yes
K00518	nickel superoxide dismutase [EC:1.15.1.1]	0.32	2.8E-169	2.1E-168	0.35	yes
K07654	two-component system, OmpR family, sensor histidine kinase MtrB [EC:2.7.13.3]	0.32	4.5E-169	3.4E-168	0.34	yes
K00456	cysteine dioxygenase [EC:1.13.11.20]	0.32	7.3E-169	5.5E-168	0.36	yes
K00301	sarcosine oxidase [EC:1.5.3.1]	0.32	9.0E-169	6.8E-168	0.37	yes
K05587	bidirectional [NiFe] hydrogenase diaphorase subunit [EC:7.1.1.2]	0.32	1.2E-168	9.0E-168	0.37	yes
K06118	UDP-sulfoquinovose synthase [EC:3.13.1.1]	0.32	1.2E-168	9.0E-168	0.35	yes
K05973	poly(3-hydroxybutyrate) depolymerase [EC:3.1.1.75]	0.32	1.7E-168	1.3E-167	0.35	yes
K00045	mannitol 2-dehydrogenase [EC:1.1.1.67]	0.32	5.2E-168	3.9E-167	0.34	yes
K05994	bacterial leucyl aminopeptidase [EC:3.4.11.10]	0.32	5.7E-168	4.2E-167	0.37	yes
K00480	salicylate hydroxylase [EC:1.14.13.1]	0.32	6.0E-168	4.4E-167	0.35	
K15780	bifunctional protein TilS/HprT [EC:6.3.4.19 2.4.2.8]	0.32	7.4E-168	5.5E-167	0.40	
K07649	two-component system, OmpR family, sensor histidine kinase TctE [EC:2.7.13.3]	0.32	7.6E-168	5.6E-167	0.35	
K10125	two-component system, NtrC family, C4-dicarboxylate transport sensor histidine kinase DctB [EC:2.7.13.3]	0.32	1.3E-167	9.5E-167	0.35	yes
K15526	L-cysteine:1D-myo-inositol 2-amino-2-deoxy-alpha-D-glucopyranoside ligase [EC:6.3.1.13]	0.32	1.5E-166	1.1E-165	0.34	yes
K01796	alpha-methylacyl-CoA racemase [EC:5.1.99.4]	0.32	2.0E-166	1.5E-165	0.35	yes
K00118	glucose-fructose oxidoreductase [EC:1.1.99.28]	0.32	1.0E-165	7.3E-165	0.37	yes
K13942	5,10-methenyltetrahydromethanopterin hydrogenase [EC:1.12.98.2]	0.32	1.1E-165	8.0E-165	0.36	yes
K03862	vanillate monooxygenase [EC:1.14.13.82]	0.32	2.0E-165	1.5E-164	0.35	yes

Predictor	Description	Pearson's r	P	FDR-adjusted P	Spearman's rho	Associated with fractures (P<0.05)
K05996	carboxypeptidase T [EC:3.4.17.18]	0.32	4.1E-165	3.0E-164	0.37	yes
K00720	ceramide glucosyltransferase [EC:2.4.1.80]	0.32	1.0E-164	7.2E-164	0.34	yes
K03863	vanillate monooxygenase ferredoxin subunit	0.32	1.7E-164	1.2E-163	0.35	yes
K01392	thimet oligopeptidase [EC:3.4.24.15]	0.32	4.0E-164	2.9E-163	0.36	yes
K13004	galacturonosyltransferase WbtD [EC:2.4.1.-]	0.32	6.0E-164	4.3E-163	0.34	yes
K10797	2-enoate reductase [EC:1.3.1.31]	0.32	8.1E-164	5.8E-163	0.33	yes
K01067	acetyl-CoA hydrolase [EC:3.1.2.1]	0.32	1.1E-163	7.8E-163	0.35	yes
K00154	coniferyl-aldehyde dehydrogenase [EC:1.2.1.68]	0.32	1.3E-163	9.3E-163	0.35	yes
K08966	2-hydroxy-3-keto-5-methylthiopentenyl-1-phosphate phosphatase [EC:3.1.3.87]	0.32	1.8E-163	1.3E-162	0.35	yes
K06042	precorrin-8X/cobalt-precorrin-8 methylmutase [EC:5.4.99.61 5.4.99.60]	0.32	2.5E-163	1.8E-162	0.34	
K00663	aminoglycoside 6'-N-acetyltransferase [EC:2.3.1.82]	0.32	2.9E-163	2.0E-162	0.35	yes
K05577	NAD(P)H-quinone oxidoreductase subunit 5 [EC:7.1.1.2]	0.32	3.0E-163	2.1E-162	0.34	yes
K13777	geranyl-CoA carboxylase alpha subunit [EC:6.4.1.5]	0.32	5.3E-163	3.7E-162	0.36	yes
K05829	LysW-gamma-L-alpha-aminoadipyl-6-phosphate/LysW-L-glutamyl-5-phosphate reductase [EC:1.2.1.103 1.2.1.106]	0.32	7.3E-163	5.1E-162	0.36	yes
K00449	protocatechuate 3,4-dioxygenase, beta subunit [EC:1.13.11.3]	0.32	1.6E-162	1.1E-161	0.34	yes
K02668	two-component system, NtrC family, sensor histidine kinase PilS [EC:2.7.13.3]	0.32	3.3E-162	2.3E-161	0.35	yes
K12990	rhamnosyltransferase [EC:2.4.1.-]	0.32	3.7E-162	2.6E-161	0.36	
K03335	inosose dehydratase [EC:4.2.1.44]	0.31	8.1E-162	5.6E-161	0.35	
K11333	3,8-divinyl chlorophyllide a/chlorophyllide a reductase subunit X [EC:1.3.7.14 1.3.7.15]	0.31	1.2E-161	8.3E-161	0.34	yes
K01856	muconate cycloisomerase [EC:5.5.1.1]	0.31	2.0E-161	1.4E-160	0.35	
K13832	3-dehydroquinate dehydratase / shikimate dehydrogenase [EC:4.2.1.10 1.1.1.25]	0.31	2.5E-161	1.7E-160	0.38	yes
K15915	undecaprenyl phosphate N,N'-diacetylbacillosamine 1-phosphate transferase [EC:2.7.8.36]	0.31	3.0E-161	2.1E-160	0.36	
K01623	fructose-bisphosphate aldolase, class I [EC:4.1.2.13]	0.31	4.0E-161	2.7E-160	0.34	yes
K13778	geranyl-CoA carboxylase beta subunit [EC:6.4.1.5]	0.31	4.6E-161	3.2E-160	0.35	yes
K00082	5-amino-6-(5-phosphoribosylamino)uracil reductase [EC:1.1.1.193]	0.31	6.7E-161	4.6E-160	0.35	yes
K05825	2-aminoadipate transaminase [EC:2.6.1.-]	0.31	7.2E-161	4.9E-160	0.35	
K05602	histidinol-phosphatase [EC:3.1.3.15]	0.31	1.2E-160	8.2E-160	0.33	yes
K13810	transaldolase / glucose-6-phosphate isomerase [EC:2.2.1.2 5.3.1.9]	0.31	2.6E-160	1.8E-159	0.37	
K08295	2-aminobenzoate-CoA ligase [EC:6.2.1.32]	0.31	3.9E-160	2.6E-159	0.35	yes
K12583	phosphatidylinositol alpha 1,6-mannosyltransferase [EC:2.4.1.-]	0.31	5.4E-160	3.7E-159	0.34	yes
K16652	decaprenylphospho-beta-D-erythro-pentofuranosid-2-ulose 2-reductase [EC:1.1.1.333]	0.31	1.4E-159	9.4E-159	0.34	yes
K08093	3-hexulose-6-phosphate synthase [EC:4.1.2.43]	0.31	2.0E-159	1.3E-158	0.33	
K00496	alkane 1-monooxygenase [EC:1.14.15.3]	0.31	3.1E-159	2.1E-158	0.34	yes
K12508	feruloyl-CoA synthase [EC:6.2.1.34]	0.31	6.0E-159	4.0E-158	0.35	yes
K09065	N-acetylornithine carbamoyltransferase [EC:2.1.3.9]	0.31	8.6E-159	5.8E-158	0.35	yes
K00980	glycerol-3-phosphate cytidylyltransferase [EC:2.7.7.39]	0.31	1.4E-158	9.3E-158	0.34	yes
K08644	tentoxilysin [EC:3.4.24.68]	0.31	2.2E-158	1.5E-157	0.36	
K06122	glycerol dehydratase small subunit [EC:4.2.1.30]	0.31	4.9E-158	3.3E-157	0.36	
K14980	two-component system, OmpR family, sensor histidine kinase ChvG [EC:2.7.13.3]	0.31	7.6E-158	5.0E-157	0.36	yes
K00186	2-oxoisovalerate ferredoxin oxidoreductase alpha subunit [EC:1.2.7.7]	0.31	7.9E-158	5.2E-157	0.39	yes
K06382	stage II sporulation protein E [EC:3.1.3.16]	0.31	2.2E-157	1.5E-156	0.33	yes
K05716	cyclic 2,3-diphosphoglycerate synthase [EC:6.5.1.9]	0.31	2.6E-157	1.7E-156	0.35	

Predictor	Description	Pearson's r	P	FDR-adjusted P	Spearman's rho	Associated with fractures (P<0.05)
K00477	phytanoyl-CoA hydroxylase [EC:1.14.11.18]	0.31	3.7E-157	2.4E-156	0.35	yes
K09844	carotenoid 1,2-hydratase [EC:4.2.1.131]	0.31	8.7E-157	5.7E-156	0.35	yes
K06379	stage II sporulation protein AB (anti-sigma F factor) [EC:2.7.11.1]	0.31	1.1E-156	7.2E-156	0.33	yes
K00947	molybdenum storage protein	0.31	1.8E-156	1.2E-155	0.34	yes
K05828	[amino group carrier protein]-L-2-aminoadipate 6-kinase [EC:2.7.2.17]	0.31	2.0E-156	1.3E-155	0.36	yes
K02827	cytochrome aa3-600 menaquinol oxidase subunit I [EC:7.1.1.5]	0.31	2.0E-156	1.3E-155	0.35	yes
K15520	mycothiol synthase [EC:2.3.1.189]	0.31	7.7E-156	5.0E-155	0.33	yes
K15509	sulfopropanediol 3-dehydrogenase [EC:1.1.1.308]	0.31	8.0E-156	5.2E-155	0.37	yes
K15521	D-inositol-3-phosphate glycosyltransferase [EC:2.4.1.250]	0.31	1.1E-155	7.1E-155	0.33	yes
K11211	3-deoxy-D-manno-octulosonic acid kinase [EC:2.7.1.166]	0.31	1.2E-155	7.7E-155	0.34	yes
K01561	haloacetate dehalogenase [EC:3.8.1.3]	0.31	1.2E-155	7.7E-155	0.35	yes
K12991	rhamnosyltransferase [EC:2.4.1.-]	0.31	3.2E-155	2.1E-154	0.27	
K09461	anthraniloyl-CoA monooxygenase [EC:1.14.13.40]	0.31	3.6E-155	2.3E-154	0.34	yes
K16653	decaprenylphospho-beta-D-ribofuranose 2-oxidase [EC:1.1.98.3]	0.31	5.2E-155	3.3E-154	0.34	yes
K02322	DNA polymerase II large subunit [EC:2.7.7.7]	0.31	1.1E-154	7.0E-154	0.38	yes
K00588	caffeoyl-CoA O-methyltransferase [EC:2.1.1.104]	0.31	1.5E-154	9.5E-154	0.34	yes
K01583	arginine decarboxylase [EC:4.1.1.19]	0.31	2.4E-154	1.5E-153	0.37	
K00437	[NiFe] hydrogenase large subunit [EC:1.12.2.1]	0.31	2.8E-154	1.8E-153	0.34	yes
K00282	glycine dehydrogenase subunit 1 [EC:1.4.4.2]	0.31	3.2E-154	2.0E-153	0.33	yes
K16515	4-oxalomesaconate hydratase [EC:4.2.1.83]	0.31	3.3E-154	2.1E-153	0.35	yes
K10710	fructoselysine 6-kinase [EC:2.7.1.218]	0.31	3.6E-154	2.3E-153	0.33	yes
K01458	N-formylglutamate deformylase [EC:3.5.1.68]	0.31	3.7E-154	2.3E-153	0.37	yes
K06399	stage IV sporulation protein B [EC:3.4.21.116]	0.31	5.6E-154	3.5E-153	0.33	yes
K00283	glycine dehydrogenase subunit 2 [EC:1.4.4.2]	0.31	6.0E-154	3.8E-153	0.33	yes
K03403	magnesium chelatase subunit H [EC:6.6.1.1]	0.31	1.0E-153	6.2E-153	0.35	yes
K16648	arabinofuranan 3-O-arabinosyltransferase [EC:2.4.2.-]	0.31	1.2E-153	7.5E-153	0.35	yes
K06720	L-ectoine synthase [EC:4.2.1.108]	0.31	1.3E-153	8.1E-153	0.33	yes
K01578	malonyl-CoA decarboxylase [EC:4.1.1.9]	0.31	1.3E-153	8.1E-153	0.35	yes
K14048	urease subunit gamma/beta [EC:3.5.1.5]	0.31	1.5E-153	9.3E-153	0.36	yes
K01212	levanase [EC:3.2.1.65]	0.31	2.0E-153	1.2E-152	0.35	yes
K03852	sulfoacetaldehyde acetyltransferase [EC:2.3.3.15]	0.31	2.3E-153	1.4E-152	0.35	yes
K06152	gluconate 2-dehydrogenase gamma chain [EC:1.1.99.3]	0.31	2.4E-153	1.5E-152	0.34	
K06718	L-2,4-diaminobutyric acid acetyltransferase [EC:2.3.1.178]	0.31	2.6E-153	1.6E-152	0.34	yes
K13671	alpha-1,2-mannosyltransferase [EC:2.4.1.-]	0.31	3.6E-153	2.2E-152	0.33	yes
K14731	epsilon-lactone hydrolase [EC:3.1.1.83]	0.31	4.5E-153	2.8E-152	0.33	yes
K07716	two-component system, cell cycle sensor histidine kinase PleC [EC:2.7.13.3]	0.31	1.9E-152	1.2E-151	0.35	yes
K03930	putative tributyrin esterase [EC:3.1.1.-]	0.31	4.6E-152	2.8E-151	0.32	yes
K00317	dimethylamine/trimethylamine dehydrogenase [EC:1.5.8.1 1.5.8.2]	0.31	5.0E-152	3.0E-151	0.37	yes
K01617	2-oxo-3-hexenedioate decarboxylase [EC:4.1.1.77]	0.31	6.7E-152	4.1E-151	0.34	yes
K00505	tyrosinase [EC:1.14.18.1]	0.31	8.1E-152	4.9E-151	0.35	yes
K01801	maleylpyruvate isomerase [EC:5.2.1.4]	0.31	9.0E-152	5.4E-151	0.34	
K00752	hyaluronan synthase [EC:2.4.1.212]	0.31	1.0E-151	6.0E-151	0.36	yes

Predictor	Description	Pearson's r	P	FDR-adjusted P	Spearman's rho	Associated with fractures (P<0.05)
K04782	isochorismate pyruvate lyase [EC:4.2.99.21]	0.31	1.4E-151	8.4E-151	0.35	
K01477	allantoicase [EC:3.5.3.4]	0.31	1.5E-151	9.0E-151	0.35	yes
K05951	NAD+---dinitrogen-reductase ADP-D-ribosyltransferase [EC:2.4.2.37]	0.31	1.5E-151	9.0E-151	0.35	yes
K01857	3-carboxy-cis,cis-muconate cycloisomerase [EC:5.5.1.2]	0.30	2.1E-151	1.3E-150	0.33	yes
K03715	1,2-diacylglycerol 3-beta-galactosyltransferase [EC:2.4.1.46]	0.30	5.4E-151	3.2E-150	0.39	
K01286	D-alanyl-D-alanine carboxypeptidase [EC:3.4.16.4]	0.30	2.1E-150	1.3E-149	0.32	yes
K16163	maleylpyruvate isomerase [EC:5.2.1.4]	0.30	3.0E-150	1.8E-149	0.33	
K12254	4-guanidinobutyraldehyde dehydrogenase / NAD-dependent aldehyde dehydrogenase [EC:1.2.1.54 1.2.1.-]	0.30	6.9E-150	4.1E-149	0.35	yes
K14949	serine/threonine-protein kinase PknG [EC:2.7.11.1]	0.30	1.4E-149	8.3E-149	0.33	yes
K10531	L-ornithine N5-monooxygenase [EC:1.14.13.195 1.14.13.196]	0.30	2.4E-149	1.4E-148	0.33	yes
K15525	N-acetyl-1-D-myo-inositol-2-amino-2-deoxy-alpha-D-glucopyranoside deacetylase [EC:3.5.1.103]	0.30	3.0E-149	1.8E-148	0.33	yes
K10715	two-component system, sensor histidine kinase RpfC [EC:2.7.13.3]	0.30	6.3E-149	3.7E-148	0.36	yes
K00570	phosphatidylethanolamine/phosphatidyl-N-methylethanolamine N-methyltransferase [EC:2.1.1.17 2.1.1.71]	0.30	6.4E-149	3.8E-148	0.34	yes
K04040	chlorophyll/bacteriochlorophyll a synthase [EC:2.5.1.62 2.5.1.133]	0.30	6.6E-149	3.9E-148	0.35	yes
K14136	decaprenyl-phosphate phosphoribosyltransferase [EC:2.4.2.45]	0.30	1.0E-148	5.9E-148	0.35	yes
K00220	cyclohexadieny/prephenate dehydrogenase [EC:1.3.1.43 1.3.1.12]	0.30	1.1E-148	6.4E-148	0.35	yes
K01567	peptidoglycan-N-acetylmuramic acid deacetylase [EC:3.5.1.-]	0.30	1.1E-148	6.4E-148	0.32	yes
K01593	aromatic-L-amino-acid/L-tryptophan decarboxylase [EC:4.1.1.28 4.1.1.105]	0.30	1.5E-148	8.7E-148	0.34	yes
K07991	archaeal preflagellin peptidase FlaK [EC:3.4.23.52]	0.30	1.7E-148	9.9E-148	0.37	
K15022	formate dehydrogenase (NADP+) beta subunit [EC:1.17.1.10]	0.30	1.7E-148	9.9E-148	0.36	yes
K00972	UDP-N-acetylglucosamine/UDP-N-acetylgalactosamine diphosphorylase [EC:2.7.7.23 2.7.7.83]	0.30	4.3E-148	2.5E-147	0.32	yes
K13941	2-amino-4-hydroxy-6-hydroxymethyldihydropteridine diphosphokinase / dihydropteroate synthase [EC:2.7.6.3 2.5.1.15]	0.30	7.9E-148	4.6E-147	0.32	
K04116	cyclohexanecarboxylate-CoA ligase [EC:6.2.1.-]	0.30	1.4E-147	8.1E-147	0.36	yes
K01031	3-oxoadipate CoA-transferase, alpha subunit [EC:2.8.3.6]	0.30	1.9E-147	1.1E-146	0.34	
K08967	1,2-dihydroxy-3-keto-5-methylthiopentene dioxygenase [EC:1.13.11.53 1.13.11.54]	0.30	1.1E-146	6.3E-146	0.33	
K10187	germacradienol/geosmin synthase [EC:4.2.3.22 4.2.3.75 4.1.99.16]	0.30	1.2E-146	6.9E-146	0.34	yes
K16165	fumarylpyruvate hydrolase [EC:3.7.1.20]	0.30	1.8E-146	1.0E-145	0.33	yes
K00803	alkyldihydroxyacetonephosphate synthase [EC:2.5.1.26]	0.30	3.9E-146	2.2E-145	0.32	yes
K08691	malyl-CoA/(S)-citramalyl-CoA lyase [EC:4.1.3.24 4.1.3.25]	0.30	4.1E-146	2.3E-145	0.35	yes
K13522	bifunctional NMN adenylyltransferase/nudix hydrolase [EC:2.7.7.1 3.6.1.-]	0.30	5.2E-146	3.0E-145	0.36	yes
K11693	peptidoglycan pentaglycine glycine transferase (the first glycine) [EC:2.3.2.16]	0.30	5.4E-146	3.1E-145	0.34	yes
K01039	glutaconate CoA-transferase, subunit A [EC:2.8.3.12]	0.30	9.5E-146	5.4E-145	0.33	yes
K00274	monoamine oxidase [EC:1.4.3.4]	0.30	1.3E-145	7.4E-145	0.32	yes
K05934	precorrin-3B C17-methyltransferase / cobalt-factor III methyltransferase [EC:2.1.1.131 2.1.1.272]	0.30	1.9E-145	1.1E-144	0.32	
K00198	anaerobic carbon-monoxide dehydrogenase catalytic subunit [EC:1.2.7.4]	0.30	2.2E-145	1.2E-144	0.33	yes
K10714	methylene-tetrahydromethanopterin dehydrogenase [EC:1.5.1.-]	0.30	2.7E-145	1.5E-144	0.37	
K02189	cobalt-precorrin 5A hydrolase [EC:3.7.1.12]	0.30	3.2E-145	1.8E-144	0.32	
K06133	4'-phosphopantetheinyl transferase [EC:2.7.8.-]	0.30	5.7E-145	3.2E-144	0.32	
K05898	3-oxosteroid 1-dehydrogenase [EC:1.3.99.4]	0.30	7.3E-145	4.1E-144	0.34	yes
K13745	L-2,4-diaminobutyrate decarboxylase [EC:4.1.1.86]	0.30	8.1E-145	4.5E-144	0.32	
K01758	cystathionine gamma-lyase [EC:4.4.1.1]	0.30	8.3E-145	4.6E-144	0.33	
K15230	ATP-citrate lyase alpha-subunit [EC:2.3.3.8]	0.30	1.1E-144	6.1E-144	0.39	yes

Predictor	Description	Pearson's r	P	FDR-adjusted P	Spearman's rho	Associated with fractures (P<0.05)
K15231	ATP-citrate lyase beta-subunit [EC:2.3.3.8]	0.30	1.1E-144	6.1E-144	0.39	yes
K15632	23S rRNA (adenine-C8)-methyltransferase [EC:2.1.1.224]	0.30	1.1E-144	6.1E-144	0.32	yes
K04127	isopenicillin-N epimerase [EC:5.1.1.17]	0.30	1.4E-144	7.8E-144	0.33	yes
K13727	phenolic acid decarboxylase [EC:4.1.1.-]	0.30	1.6E-144	8.8E-144	0.34	
K12349	neutral ceramidase [EC:3.5.1.23]	0.30	3.6E-144	2.0E-143	0.33	yes
K05281	2'-hydroxyisoflavone reductase [EC:1.3.1.45]	0.30	6.3E-144	3.5E-143	0.35	yes
K08094	6-phospho-3-hexuloisomerase [EC:5.3.1.27]	0.30	1.3E-143	7.1E-143	0.32	
K01078	acid phosphatase [EC:3.1.3.2]	0.30	1.7E-143	9.3E-143	0.34	yes
K04036	divinyl protochlorophyllide a 8-vinyl-reductase [EC:1.-.-.-]	0.30	2.2E-143	1.2E-142	0.34	yes
K06015	N-acyl-D-amino-acid deacylase [EC:3.5.1.81]	0.30	2.6E-143	1.4E-142	0.33	yes
K00863	triose/dihydroxyacetone kinase / FAD-AMP lyase (cyclizing) [EC:2.7.1.28 2.7.1.29 4.6.1.15]	0.30	2.7E-143	1.5E-142	0.33	yes
K00702	cellobiose phosphorylase [EC:2.4.1.20]	0.30	4.2E-143	2.3E-142	0.32	yes
K00544	betaine-homocysteine S-methyltransferase [EC:2.1.1.5]	0.30	8.2E-143	4.5E-142	0.34	yes
K10674	ectoine hydroxylase [EC:1.14.11.55]	0.30	1.5E-142	8.1E-142	0.33	yes
K04101	protocatechuate 4,5-dioxygenase, beta chain [EC:1.13.11.8]	0.30	4.2E-142	2.3E-141	0.35	yes
K16514	4-oxalomesaconate tautomerase [EC:5.3.2.8]	0.30	4.3E-142	2.3E-141	0.34	yes
K13058	mannosylfructose-phosphate synthase [EC:2.4.1.246]	0.30	4.4E-142	2.4E-141	0.34	
K01415	endothelin-converting enzyme [EC:3.4.24.71]	0.30	4.8E-142	2.6E-141	0.39	yes
K15733	dye decolorizing peroxidase [EC:1.11.1.19]	0.30	8.7E-142	4.7E-141	0.32	yes
K14335	alpha-1,6-mannosyltransferase [EC:2.4.1.-]	0.30	1.0E-141	5.4E-141	0.33	
K11337	bacteriochlorophyllide a dehydrogenase [EC:1.1.1.396]	0.29	3.0E-141	1.6E-140	0.34	yes
K16317	tRNA (pseudouridine54-N1)-methyltransferase [EC:2.1.1.257]	0.29	3.7E-141	2.0E-140	0.36	yes
K11691	two-component system, CitB family, sensor histidine kinase DctS [EC:2.7.13.3]	0.29	5.1E-141	2.7E-140	0.35	yes
K16306	fructose-bisphosphate aldolase / 2-amino-3,7-dideoxy-D-threo-hept-6-ulosonate synthase [EC:4.1.2.13 2.2.1.10]	0.29	1.4E-140	7.5E-140	0.36	yes
K01469	5-oxoprolinase (ATP-hydrolysing) [EC:3.5.2.9]	0.29	2.3E-140	1.2E-139	0.32	yes
K00172	pyruvate ferredoxin oxidoreductase gamma subunit [EC:1.2.7.1]	0.29	3.4E-140	1.8E-139	0.32	yes
K14266	tryptophan 7-halogenase [EC:1.14.19.9]	0.29	4.0E-140	2.1E-139	0.34	
K01761	methionine-gamma-lyase [EC:4.4.1.11]	0.29	1.1E-139	5.8E-139	0.33	
K02119	V/A-type H+/Na+-transporting ATPase subunit C	0.29	1.1E-139	5.8E-139	0.32	
K00273	D-amino-acid oxidase [EC:1.4.3.3]	0.29	1.3E-139	6.9E-139	0.33	yes
K01075	4-hydroxybenzoyl-CoA thioesterase [EC:3.1.2.23]	0.29	1.7E-139	9.0E-139	0.32	yes
K13541	cobalt-precorrin 5A hydrolase / cobalt-factor III methyltransferase / precorrin-3B C17-methyltransferase [EC:3.7.1.12 2.1.1.272 2.1.1.131]	0.29	1.9E-139	1.0E-138	0.33	yes
K05936	precorrin-4/cobalt-precorrin-4 C11-methyltransferase [EC:2.1.1.133 2.1.1.271]	0.29	1.9E-139	1.0E-138	0.32	
K00772	5'-methylthioadenosine phosphorylase [EC:2.4.2.28]	0.29	2.5E-139	1.3E-138	0.32	
K08688	creatinase [EC:3.5.3.3]	0.29	2.7E-139	1.4E-138	0.33	yes
K07031	D-glycero-alpha-D-manno-heptose-7-phosphate kinase [EC:2.7.1.168]	0.29	2.8E-139	1.5E-138	0.30	yes
K08082	two-component system, LytTR family, sensor histidine kinase AlgZ [EC:2.7.13.3]	0.29	2.8E-139	1.5E-138	0.33	yes
K06020	energy-dependent translational throttle protein EttA	0.29	4.7E-139	2.4E-138	0.33	yes
K08964	methylthioribulose-1-phosphate dehydratase [EC:4.2.1.109]	0.29	6.6E-139	3.4E-138	0.32	
K01641	hydroxymethylglutaryl-CoA synthase [EC:2.3.3.10]	0.29	8.1E-139	4.2E-138	0.31	yes
K15513	benzoyl-CoA-dihydrodiol lyase [EC:4.1.2.44]	0.29	1.6E-138	8.3E-138	0.34	yes
K00140	malonate-semialdehyde dehydrogenase (acetylating) / methylmalonate-semialdehyde dehydrogenase [EC:1.2.1.18 1.2.1.27]	0.29	2.6E-138	1.3E-137	0.32	yes

Predictor	Description	Pearson's r	P	FDR-adjusted P	Spearman's rho	Associated with fractures (P<0.05)
K00261	glutamate dehydrogenase (NAD(P)+) [EC:1.4.1.3]	0.29	6.7E-138	3.5E-137	0.33	
K16173	glutaryl-CoA dehydrogenase (non-decarboxylating) [EC:1.3.99.32]	0.29	1.3E-137	6.7E-137	0.34	yes
K09880	enolase-phosphatase E1 [EC:3.1.3.77]	0.29	1.3E-137	6.7E-137	0.32	
K08965	2,3-diketo-5-methylthiopentyl-1-phosphate enolase [EC:5.3.2.5]	0.29	2.2E-137	1.1E-136	0.35	yes
K01032	3-oxoadipate CoA-transferase, beta subunit [EC:2.8.3.6]	0.29	2.3E-137	1.2E-136	0.33	
K16164	acylpyruvate hydrolase [EC:3.7.1.5]	0.29	3.4E-137	1.7E-136	0.34	yes
K11336	3-vinyl bacteriochlorophyllide hydratase [EC:4.2.1.165]	0.29	3.8E-137	1.9E-136	0.33	yes
K14660	nodulation protein E [EC:2.3.1.-]	0.29	4.4E-137	2.2E-136	0.37	yes
K01884	cysteinyl-tRNA synthetase, unknown class [EC:6.1.1.16]	0.29	8.3E-137	4.2E-136	0.36	
K07535	2-hydroxycyclohexanecarboxyl-CoA dehydrogenase [EC:1.1.1.-]	0.29	1.3E-136	6.6E-136	0.34	yes
K00534	ferredoxin hydrogenase small subunit [EC:1.12.7.2]	0.29	1.7E-136	8.6E-136	0.35	yes
K16047	3-hydroxy-9,10-secoandrosta-1,3,5(10)-triene-9,17-dione monooxygenase [EC:1.14.14.12]	0.29	1.1E-135	5.6E-135	0.33	yes
K15739	D-alanine---(R)-lactate ligase [EC:6.1.2.1]	0.29	1.3E-135	6.6E-135	0.32	yes
K00608	aspartate carbamoyltransferase [EC:2.1.3.2]	0.29	1.6E-135	8.1E-135	0.36	yes
K07539	6-oxocyclohex-1-ene-carbonyl-CoA hydrolase [EC:3.7.1.21]	0.29	1.9E-135	9.6E-135	0.35	yes
K10672	glycine reductase complex component B subunit gamma [EC:1.21.4.2]	0.29	3.8E-135	1.9E-134	0.35	yes
K02204	homoserine kinase type II [EC:2.7.1.39]	0.29	5.8E-135	2.9E-134	0.33	
K12448	UDP-arabinose 4-epimerase [EC:5.1.3.5]	0.29	1.1E-134	5.5E-134	0.35	
K03465	thymidylate synthase (FAD) [EC:2.1.1.148]	0.29	3.1E-134	1.6E-133	0.30	
K01574	acetoacetate decarboxylase [EC:4.1.1.4]	0.29	3.9E-134	1.9E-133	0.33	
K04110	benzoate-CoA ligase [EC:6.2.1.25]	0.29	8.2E-134	4.1E-133	0.34	
K13540	precorrin-2 C20-methyltransferase / precorrin-3B C17-methyltransferase [EC:2.1.1.130 2.1.1.131]	0.29	8.9E-134	4.4E-133	0.32	yes
K00218	protochlorophyllide reductase [EC:1.3.1.33]	0.29	1.5E-133	7.5E-133	0.37	
K00756	pyrimidine-nucleoside phosphorylase [EC:2.4.2.2]	0.29	3.4E-133	1.7E-132	0.31	
K01502	aliphatic nitrilase [EC:3.5.5.7]	0.29	3.4E-133	1.7E-132	0.31	yes
K13607	cinnamoyl-CoA:phenyllactate CoA-transferase [EC:2.8.3.17]	0.29	7.9E-133	3.9E-132	0.32	yes
K15864	nitrite reductase (NO-forming) / hydroxylamine reductase [EC:1.7.2.1 1.7.99.1]	0.29	1.8E-132	8.9E-132	0.34	yes
K05297	rubredoxin---NAD+ reductase [EC:1.18.1.1]	0.29	2.0E-132	9.9E-132	0.32	yes
K06151	gluconate 2-dehydrogenase alpha chain [EC:1.1.99.3]	0.29	2.3E-132	1.1E-131	0.32	
K10220	4-oxalmesaconate hydratase [EC:4.2.1.83]	0.29	3.4E-132	1.7E-131	0.33	yes
K03381	catechol 1,2-dioxygenase [EC:1.13.11.1]	0.29	3.4E-132	1.7E-131	0.32	
K00869	mevalonate kinase [EC:2.7.1.36]	0.29	5.2E-132	2.5E-131	0.31	yes
K01174	micrococcal nuclease [EC:3.1.31.1]	0.29	5.2E-132	2.5E-131	0.30	
K05889	polyvinyl alcohol dehydrogenase (cytochrome) [EC:1.1.2.6]	0.29	5.6E-132	2.7E-131	0.37	yes
K15512	benzoyl-CoA 2,3-epoxidase subunit B [EC:1.14.13.208]	0.29	6.1E-132	3.0E-131	0.33	yes
K05550	benzoate/toluate 1,2-dioxygenase subunit beta [EC:1.14.12.10 1.14.12.-]	0.29	9.3E-132	4.5E-131	0.32	
K10796	D-proline reductase (dithiol)-stabilizing protein PrdE	0.28	1.4E-131	6.8E-131	0.35	yes
K00635	diacylglycerol O-acyltransferase / wax synthase [EC:2.3.1.20 2.3.1.75]	0.28	1.6E-131	7.8E-131	0.35	
K01416	snapalysin [EC:3.4.24.77]	0.28	3.1E-131	1.5E-130	0.33	
K14339	alpha-1,6-mannosyltransferase [EC:2.4.1.-]	0.28	3.3E-131	1.6E-130	0.33	yes
K07537	cyclohexa-1,5-dienecarbonyl-CoA hydratase [EC:4.2.1.100]	0.28	4.5E-131	2.2E-130	0.35	yes
K07739	elongator complex protein 3 [EC:2.3.1.48]	0.28	7.0E-131	3.4E-130	0.29	yes

Predictor	Description	Pearson's r	P	FDR-adjusted P	Spearman's rho	Associated with fractures (P<0.05)
K12996	rhamnosyltransferase [EC:2.4.1.-]	0.28	7.4E-131	3.6E-130	0.30	yes
K00074	3-hydroxybutyryl-CoA dehydrogenase [EC:1.1.1.157]	0.28	1.2E-130	5.8E-130	0.30	
K03752	molybdenum cofactor guanylyltransferase [EC:2.7.7.77]	0.28	2.2E-130	1.1E-129	0.31	
K00839	(S)-ureidoglycine---glyoxylate transaminase [EC:2.6.1.112]	0.28	4.2E-130	2.0E-129	0.31	yes
K10942	two-component system, sensor histidine kinase FlrB [EC:2.7.13.3]	0.28	8.6E-130	4.1E-129	0.32	yes
K01628	L-fuculose-phosphate aldolase [EC:4.1.2.17]	0.28	9.7E-130	4.6E-129	0.31	
K05549	benzoate/toluate 1,2-dioxygenase subunit alpha [EC:1.14.12.10 1.14.12.-]	0.28	1.1E-129	5.2E-129	0.32	yes
K12658	4-hydroxyproline epimerase [EC:5.1.1.8]	0.28	1.5E-129	7.1E-129	0.31	
K16649	rhamnopyranosyl-N-acetylglucosaminyl-diphospho-decaprenol beta-1,3/1,4-galactofuranosyltransferase [EC:2.4.1.287]	0.28	4.1E-129	1.9E-128	0.32	yes
K02803	N-acetylglucosamine PTS system EIIB component [EC:2.7.1.193]	0.28	6.5E-129	3.1E-128	0.31	yes
K06016	beta-ureidopropionase / N-carbamoyl-L-amino-acid hydrolase [EC:3.5.1.6 3.5.1.87]	0.28	2.1E-128	9.9E-128	0.31	
K10253	DOPA 4,5-dioxygenase [EC:1.14.99.-]	0.28	2.2E-128	1.0E-127	0.34	yes
K00594	alditol oxidase [EC:1.1.3.41]	0.28	5.4E-128	2.5E-127	0.31	yes
K01848	methylmalonyl-CoA mutase, N-terminal domain [EC:5.4.99.2]	0.28	7.0E-128	3.3E-127	0.33	
K00360	assimilatory nitrate reductase electron transfer subunit [EC:1.7.99.-]	0.28	7.7E-128	3.6E-127	0.33	yes
K08604	vibriolysin [EC:3.4.24.25]	0.28	1.8E-127	8.5E-127	0.35	
K01822	steroid Delta-isomerase [EC:5.3.3.1]	0.28	2.3E-127	1.1E-126	0.32	
K13587	two-component system, cell cycle sensor histidine kinase and response regulator CckA [EC:2.7.13.3]	0.28	4.3E-127	2.0E-126	0.33	yes
K07697	two-component system, sporulation sensor kinase B [EC:2.7.13.3]	0.28	5.6E-127	2.6E-126	0.34	yes
K01677	fumarate hydratase subunit alpha [EC:4.2.1.2]	0.28	1.0E-126	4.7E-126	0.31	
K01729	poly(beta-D-mannuronate) lyase [EC:4.2.2.3]	0.28	1.9E-126	8.9E-126	0.31	
K14727	3-oxoadipate enol-lactonase / 4-carboxymuconolactone decarboxylase [EC:3.1.1.24 4.1.1.44]	0.28	2.7E-126	1.3E-125	0.33	yes
K06179	23S rRNA pseudouridine955/2504/2580 synthase [EC:5.4.99.24]	0.28	4.9E-126	2.3E-125	0.30	
K10793	D-proline reductase (dithiol) PrdA [EC:1.21.4.1]	0.28	5.3E-126	2.5E-125	0.36	
K01616	multifunctional 2-oxoglutarate metabolism enzyme [EC:2.2.1.5 4.1.1.71 1.2.4.2 2.3.1.61]	0.28	1.3E-125	6.0E-125	0.32	
K09845	1-hydroxycarotenoid 3,4-desaturase [EC:1.3.99.27]	0.28	1.4E-125	6.5E-125	0.33	yes
K05824	homoisocitrate dehydrogenase [EC:1.1.1.87]	0.28	1.7E-125	7.9E-125	0.34	yes
K16050	4,5:9,10-diseco-3-hydroxy-5,9,17-trioxoandrosta-1(10),2-diene-4-oate hydrolase [EC:3.7.1.17]	0.28	1.9E-125	8.8E-125	0.33	
K13831	3-hexulose-6-phosphate synthase / 6-phospho-3-hexuloisomerase [EC:4.1.2.43 5.3.1.27]	0.28	2.3E-125	1.1E-124	0.34	yes
K01003	oxaloacetate decarboxylase [EC:4.1.1.112]	0.28	2.7E-125	1.2E-124	0.33	yes
K13686	galactan 5-O-arabinofuranosyltransferase [EC:2.4.2.46]	0.28	5.8E-125	2.7E-124	0.33	
K09846	demethylspheroidene O-methyltransferase [EC:2.1.1.210]	0.28	6.4E-125	2.9E-124	0.33	yes
K16146	maltokinase [EC:2.7.1.175]	0.28	7.3E-125	3.3E-124	0.31	yes
K01905	acetate---CoA ligase (ADP-forming) subunit alpha [EC:6.2.1.13]	0.28	2.3E-124	1.1E-123	0.36	
K00797	spermidine synthase [EC:2.5.1.16]	0.28	2.7E-124	1.2E-123	0.29	
K03411	chemotaxis protein CheD [EC:3.5.1.44]	0.28	6.6E-124	3.0E-123	0.30	yes
K14153	hydroxymethylpyrimidine kinase / phosphomethylpyrimidine kinase / thiamine-phosphate diphosphorylase [EC:2.7.1.49 2.7.4.7 2.5.1.3]	0.28	6.7E-124	3.0E-123	0.33	
K11629	two-component system, OmpR family, bacitracin resistance sensor histidine kinase BceS [EC:2.7.13.3]	0.28	7.7E-124	3.5E-123	0.36	yes
K00581	tetrahydromethanopterin S-methyltransferase subunit E [EC:2.1.1.86]	0.28	8.6E-124	3.9E-123	0.35	
K12989	mannosyltransferase [EC:2.4.1.-]	0.28	9.6E-124	4.3E-123	0.34	yes
K15895	UDP-4-amino-4,6-dideoxy-L-N-acetyl-beta-L-altrosamine transaminase [EC:2.6.1.92]	0.28	1.5E-123	6.8E-123	0.34	
K01464	dihydropyrimidinase [EC:3.5.2.2]	0.28	2.7E-123	1.2E-122	0.30	

Predictor	Description	Pearson's r	P	FDR-adjusted P	Spearman's rho	Associated with fractures (P<0.05)
K00043	4-hydroxybutyrate dehydrogenase [EC:1.1.1.61]	0.28	3.3E-123	1.5E-122	0.33	yes
K09835	prolycopene isomerase [EC:5.2.1.13]	0.28	4.0E-123	1.8E-122	0.32	yes
K01800	maleylacetoacetate isomerase [EC:5.2.1.2]	0.28	6.9E-123	3.1E-122	0.32	yes
K16049	3,4-dihydroxy-9,10-secoandrosta-1,3,5(10)-triene-9,17-dione 4,5-dioxygenase [EC:1.13.11.25]	0.28	1.0E-122	4.5E-122	0.33	yes
K16150	glycogen synthase [EC:2.4.1.11]	0.27	2.4E-122	1.1E-121	0.32	yes
K13491	two-component system, chemotaxis family, response regulator WspF [EC:3.1.1.61]	0.27	3.8E-122	1.7E-121	0.32	yes
K01449	N-acetylmuramoyl-L-alanine amidase [EC:3.5.1.28]	0.27	4.6E-122	2.1E-121	0.30	yes
K01601	ribulose-bisphosphate carboxylase large chain [EC:4.1.1.39]	0.27	5.8E-122	2.6E-121	0.31	
K01692	enoyl-CoA hydratase [EC:4.2.1.17]	0.27	1.2E-121	5.3E-121	0.31	
K04771	serine protease Do [EC:3.4.21.107]	0.27	1.2E-121	5.3E-121	0.29	
K10856	acetone carboxylase, gamma subunit [EC:6.4.1.6]	0.27	2.2E-121	9.8E-121	0.33	yes
K14337	alpha-1,6-mannosyltransferase [EC:2.4.1.-]	0.27	2.3E-121	1.0E-120	0.31	yes
K01060	cephalosporin-C deacetylase [EC:3.1.1.41]	0.27	2.3E-121	1.0E-120	0.30	yes
K05518	phosphoserine phosphatase RsbX [EC:3.1.3.3]	0.27	2.9E-121	1.3E-120	0.35	yes
K03416	methylmalonyl-CoA carboxyltransferase 5S subunit [EC:2.1.3.1]	0.27	3.1E-121	1.4E-120	0.33	
K01678	fumarate hydratase subunit beta [EC:4.2.1.2]	0.27	8.1E-121	3.6E-120	0.30	
K05783	dihydroxycyclohexadiene carboxylate dehydrogenase [EC:1.3.1.25 1.3.1.-]	0.27	9.1E-121	4.0E-120	0.31	
K03337	5-deoxy-glucuronate isomerase [EC:5.3.1.30]	0.27	1.4E-120	6.2E-120	0.30	
K00132	acetaldehyde dehydrogenase (acetylating) [EC:1.2.1.10]	0.27	1.9E-120	8.3E-120	0.29	yes
K03412	two-component system, chemotaxis family, protein-glutamate methylesterase/glutaminase [EC:3.1.1.61 3.5.1.44]	0.27	2.8E-120	1.2E-119	0.30	
K01501	nitrilase [EC:3.5.5.1]	0.27	3.1E-120	1.4E-119	0.34	
K13779	isohexenylglutaconyl-CoA hydratase [EC:4.2.1.57]	0.27	4.5E-120	2.0E-119	0.33	
K15982	3-ketosteroid 9alpha-monooxygenase subunit A [EC:1.14.15.30]	0.27	7.6E-120	3.3E-119	0.31	yes
K00821	acetylornithine/N-succinyldiaminopimelate aminotransferase [EC:2.6.1.11 2.6.1.17]	0.27	1.2E-119	5.2E-119	0.29	
K05830	LysW-gamma-L-lysine/LysW-L-ornithine aminotransferase [EC:2.6.1.118 2.6.1.-]	0.27	1.8E-119	7.8E-119	0.34	
K04103	indolepyruvate decarboxylase [EC:4.1.1.74]	0.27	1.9E-119	8.3E-119	0.31	
K08963	methylthioribose-1-phosphate isomerase [EC:5.3.1.23]	0.27	2.4E-119	1.0E-118	0.30	
K01713	cyclohexadienyl dehydratase [EC:4.2.1.51 4.2.1.91]	0.27	3.0E-119	1.3E-118	0.31	
K05988	dextranase [EC:3.2.1.11]	0.27	2.0E-118	8.7E-118	0.34	yes
K10219	2-hydroxy-4-carboxymuconate semialdehyde hemiacetal dehydrogenase [EC:1.1.1.312]	0.27	5.1E-118	2.2E-117	0.33	yes
K00248	butyryl-CoA dehydrogenase [EC:1.3.8.1]	0.27	5.6E-118	2.4E-117	0.29	
K03336	3D-(3,5/4)-trihydroxycyclohexane-1,2-dione acylhydrolase (decyclizing) [EC:3.7.1.22]	0.27	9.7E-118	4.2E-117	0.29	
K16650	galactofuranosylgalactofuranosylrhamnosyl-N-acetylglucosaminyl-diphospho-decaprenol beta-1,5/1,6-galactofuranosyltransferase [EC:2.4.1.288]	0.27	1.1E-117	4.7E-117	0.30	yes
K05822	tetrahydrodipicolinate N-acetyltransferase [EC:2.3.1.89]	0.27	1.3E-117	5.6E-117	0.31	
K04100	protocatechuate 4,5-dioxygenase, alpha chain [EC:1.13.11.8]	0.27	1.4E-117	6.0E-117	0.32	yes
K03635	molybdopterin synthase catalytic subunit [EC:2.8.1.12]	0.27	1.5E-117	6.4E-117	0.30	
K01179	endoglucanase [EC:3.2.1.4]	0.27	2.1E-117	9.0E-117	0.29	yes
K13930	triphosphoribosyl-dephospho-CoA synthase [EC:2.4.2.52]	0.27	2.6E-117	1.1E-116	0.30	
K05552	minimal PKS chain-length factor (CLF/KS beta) [EC:2.3.1.- 2.3.1.260 2.3.1.235]	0.27	3.8E-117	1.6E-116	0.32	
K01849	methylmalonyl-CoA mutase, C-terminal domain [EC:5.4.99.2]	0.27	4.5E-117	1.9E-116	0.32	
K00284	glutamate synthase (ferredoxin) [EC:1.4.7.1]	0.27	9.2E-117	3.9E-116	0.30	
K13934	phosphoribosyl-dephospho-CoA transferase [EC:2.7.7.66]	0.27	1.2E-116	5.1E-116	0.30	

Predictor	Description	Pearson's r	P	FDR-adjusted P	Spearman's rho	Associated with fractures (P<0.05)
K01781	mandelate racemase [EC:5.1.2.2]	0.27	1.9E-116	8.1E-116	0.36	
K00805	heptaprenyl diphosphate synthase component 1 [EC:2.5.1.30]	0.27	3.6E-116	1.5E-115	0.28	
K05555	cyclase [EC:4.-.-.-]	0.27	6.1E-116	2.6E-115	0.32	
K03396	S-(hydroxymethyl)glutathione synthase [EC:4.4.1.22]	0.27	7.9E-116	3.3E-115	0.33	yes
K02363	2,3-dihydroxybenzoate---[aryl-carrier protein] ligase [EC:6.3.2.14 6.2.1.71]	0.27	1.2E-115	5.1E-115	0.29	
K15534	beta-D-galactosyl-(1->4)-L-rhamnose phosphorylase [EC:2.4.1.247]	0.27	1.3E-115	5.5E-115	0.29	yes
K13929	malonate decarboxylase alpha subunit [EC:2.3.1.187]	0.27	2.0E-115	8.4E-115	0.30	
K00450	gentisate 1,2-dioxygenase [EC:1.13.11.4]	0.27	2.7E-115	1.1E-114	0.30	
K16318	tRNA (guanine6-N2)-methyltransferase [EC:2.1.1.256]	0.27	2.0E-114	8.4E-114	0.31	
K15910	UDP-N-acetylbacillosamine transaminase [EC:2.6.1.34]	0.27	2.0E-114	8.4E-114	0.30	
K14986	two-component system, LuxR family, sensor kinase FixL [EC:2.7.13.3]	0.27	2.6E-114	1.1E-113	0.32	yes
K00520	mercuric reductase [EC:1.16.1.1]	0.27	3.7E-114	1.5E-113	0.30	
K11387	arabinosyltransferase C [EC:2.4.2.-]	0.27	5.4E-114	2.3E-113	0.32	yes
K07508	acetyl-CoA acyltransferase 2 [EC:2.3.1.16]	0.27	1.6E-113	6.7E-113	0.34	yes
K13995	maleamate amidohydrolase [EC:3.5.1.107]	0.26	1.8E-113	7.5E-113	0.34	
K15731	carboxy-terminal domain RNA polymerase II polypeptide A small phosphatase [EC:3.1.3.16]	0.26	3.4E-113	1.4E-112	0.32	
K01962	acetyl-CoA carboxylase carboxyl transferase subunit alpha [EC:6.4.1.2 2.1.3.15]	0.26	4.9E-113	2.0E-112	0.28	
K02427	23S rRNA (uridine2552-2'-O)-methyltransferase [EC:2.1.1.166]	0.26	8.1E-113	3.4E-112	0.29	
K10221	2-pyrone-4,6-dicarboxylate lactonase [EC:3.1.1.57]	0.26	1.3E-112	5.4E-112	0.32	yes
K03338	5-dehydro-2-deoxygluconokinase [EC:2.7.1.92]	0.26	1.5E-112	6.2E-112	0.29	
K07538	6-hydroxycyclohex-1-ene-1-carbonyl-CoA dehydrogenase [EC:1.1.1.368]	0.26	2.0E-112	8.3E-112	0.34	yes
K00472	prolyl 4-hydroxylase [EC:1.14.11.2]	0.26	3.8E-112	1.6E-111	0.32	yes
K11528	UDP-N-acetylglucosamine pyrophosphorylase [EC:2.7.7.23]	0.26	6.1E-112	2.5E-111	0.31	yes
K01963	acetyl-CoA carboxylase carboxyl transferase subunit beta [EC:6.4.1.2 2.1.3.15]	0.26	8.3E-112	3.4E-111	0.28	
K01569	oxalate decarboxylase [EC:4.1.1.2]	0.26	1.0E-111	4.1E-111	0.30	yes
K15912	UDP-N-acetylglucosamine 4,6-dehydratase [EC:4.2.1.135]	0.26	2.4E-111	9.8E-111	0.31	
K01655	homocitrate synthase [EC:2.3.3.14]	0.26	3.8E-111	1.6E-110	0.33	yes
K13688	cyclic beta-1,2-glucan synthetase [EC:2.4.1.-]	0.26	5.0E-111	2.0E-110	0.33	yes
K01432	arylformamidase [EC:3.5.1.9]	0.26	5.1E-111	2.1E-110	0.31	yes
K06176	tRNA pseudouridine13 synthase [EC:5.4.99.27]	0.26	5.7E-111	2.3E-110	0.29	
K01727	hyaluronate lyase [EC:4.2.2.1]	0.26	1.0E-110	4.1E-110	0.27	yes
K01007	pyruvate, water dikinase [EC:2.7.9.2]	0.26	1.1E-110	4.5E-110	0.28	
K03383	cyanuric acid amidohydrolase [EC:3.5.2.15]	0.26	1.1E-110	4.5E-110	0.34	
K00271	valine dehydrogenase (NAD+) [EC:1.4.1.23]	0.26	2.7E-110	1.1E-109	0.30	
K11383	two-component system, NtrC family, sensor histidine kinase KinB [EC:2.7.13.3]	0.26	3.4E-110	1.4E-109	0.32	yes
K05985	ribonuclease M5 [EC:3.1.26.8]	0.26	5.7E-110	2.3E-109	0.28	
K07179	RIO kinase 2 [EC:2.7.11.1]	0.26	6.9E-110	2.8E-109	0.34	
K03918	L-lysine 6-transaminase [EC:2.6.1.36]	0.26	9.0E-110	3.6E-109	0.30	
K10231	kojibiose phosphorylase [EC:2.4.1.230]	0.26	1.5E-109	6.0E-109	0.28	yes
K13007	glycosyltransferase WbpL [EC:2.4.1.-]	0.26	1.7E-109	6.8E-109	0.32	
K15914	N,N'-diacetylbacillosaminyl-diphospho-undecaprenol alpha-1,3-N-acetylgalactosaminyltransferase [EC:2.4.1.290]	0.26	3.0E-109	1.2E-108	0.31	
K16217	geranyl diphosphate 2-C-methyltransferase [EC:2.1.1.255]	0.26	3.4E-109	1.4E-108	0.32	

Predictor	Description	Pearson's r	P	FDR-adjusted P	Spearman's rho	Associated with fractures (P<0.05)
K00169	pyruvate ferredoxin oxidoreductase alpha subunit [EC:1.2.7.1]	0.26	4.4E-109	1.8E-108	0.29	
K01486	adenine deaminase [EC:3.5.4.2]	0.26	1.3E-108	5.2E-108	0.28	
K01769	guanylate cyclase, other [EC:4.6.1.2]	0.26	1.8E-108	7.2E-108	0.25	
K00298	N5-(carboxyethyl)ornithine synthase [EC:1.5.1.24]	0.26	2.1E-108	8.4E-108	0.29	
K00466	tryptophan 2-monooxygenase [EC:1.13.12.3]	0.26	4.2E-108	1.7E-107	0.30	yes
K00624	carnitine O-acetyltransferase [EC:2.3.1.7]	0.26	4.4E-108	1.8E-107	0.31	yes
K13927	holo-ACP synthase / triphosphoribosyl-dephospho-CoA synthase [EC:2.7.7.61 2.4.2.52]	0.26	1.7E-107	6.8E-107	0.30	
K14534	4-hydroxybutyryl-CoA dehydratase / vinylacetyl-CoA-Delta-isomerase [EC:4.2.1.120 5.3.3.3]	0.26	2.1E-107	8.3E-107	0.28	
K13020	UDP-N-acetyl-2-amino-2-deoxyglucuronate dehydrogenase [EC:1.1.1.335]	0.26	2.5E-107	9.9E-107	0.30	
K00193	acetyl-CoA decarbonylase/synthase, CODH/ACS complex subunit beta [EC:2.3.1.169]	0.26	4.1E-107	1.6E-106	0.34	
K06023	HPr kinase/phosphorylase [EC:2.7.11.- 2.7.4.-]	0.26	6.0E-107	2.4E-106	0.27	
K03716	spore photoproduct lyase [EC:4.1.99.14]	0.26	6.2E-107	2.4E-106	0.28	yes
K06209	chorismate mutase [EC:5.4.99.5]	0.26	7.1E-107	2.8E-106	0.27	
K00055	aryl-alcohol dehydrogenase [EC:1.1.1.90]	0.26	1.0E-106	3.9E-106	0.30	
K15855	exo-1,4-beta-D-glucosaminidase [EC:3.2.1.165]	0.26	1.8E-106	7.1E-106	0.31	
K09882	cobaltochelatase CobS [EC:6.6.1.2]	0.26	1.8E-106	7.1E-106	0.30	
K10622	HCOMODA/2-hydroxy-3-carboxy-muconic semialdehyde decarboxylase [EC:4.1.1.-]	0.26	2.4E-106	9.4E-106	0.32	
K04561	nitric oxide reductase subunit B [EC:1.7.2.5]	0.26	5.2E-106	2.0E-105	0.30	
K04487	cysteine desulfurase [EC:2.8.1.7]	0.26	5.6E-106	2.2E-105	0.27	
K01715	enoyl-CoA hydratase [EC:4.2.1.17]	0.26	8.5E-106	3.3E-105	0.27	
K03784	purine-nucleoside phosphorylase [EC:2.4.2.1]	0.26	1.0E-105	3.9E-105	0.27	
K01596	phosphoenolpyruvate carboxykinase (GTP) [EC:4.1.1.32]	0.26	1.1E-105	4.3E-105	0.27	
K05305	fucokinase [EC:2.7.1.52]	0.26	1.1E-105	4.3E-105	0.35	yes
K03763	DNA polymerase III subunit alpha, Gram-positive type [EC:2.7.7.7]	0.26	1.3E-105	5.1E-105	0.27	
K08252	receptor protein-tyrosine kinase [EC:2.7.10.1]	0.26	2.1E-105	8.2E-105	0.34	yes
K07260	zinc D-Ala-D-Ala carboxypeptidase [EC:3.4.17.14]	0.26	2.9E-105	1.1E-104	0.27	
K14164	glycyl-tRNA synthetase [EC:6.1.1.14]	0.26	4.1E-105	1.6E-104	0.32	yes
K07823	3-oxoadipyl-CoA thiolase [EC:2.3.1.174]	0.26	5.2E-105	2.0E-104	0.30	yes
K03518	aerobic carbon-monoxide dehydrogenase small subunit [EC:1.2.5.3]	0.26	5.9E-105	2.3E-104	0.28	
K14083	trimethylamine---corrinoid protein Co-methyltransferase [EC:2.1.1.250]	0.25	6.7E-105	2.6E-104	0.28	yes
K16218	2-methylisoborneol synthase [EC:4.2.3.118]	0.25	6.8E-105	2.6E-104	0.32	yes
K00230	menaquinone-dependent protoporphyrinogen oxidase [EC:1.3.5.3]	0.25	8.0E-105	3.1E-104	0.28	
K01299	carboxypeptidase Taq [EC:3.4.17.19]	0.25	1.4E-104	5.4E-104	0.28	yes
K10795	D-proline reductase (dithiol)-stabilizing protein PrdD	0.25	2.2E-104	8.4E-104	0.32	yes
K08355	arsenite oxidase small subunit [EC:1.20.2.1 1.20.9.1]	0.25	5.5E-104	2.1E-103	0.32	yes
K15871	bile acid CoA-transferase [EC:2.8.3.25]	0.25	7.3E-104	2.8E-103	0.27	yes
K15872	bile-acid 7alpha-dehydratase [EC:4.2.1.106]	0.25	7.3E-104	2.8E-103	0.27	yes
K06967	tRNA (adenine22-N1)-methyltransferase [EC:2.1.1.217]	0.25	1.3E-103	5.0E-103	0.27	
K13533	two-component system, sporulation sensor kinase E [EC:2.7.13.3]	0.25	1.6E-103	6.1E-103	0.32	yes
K02510	4-hydroxy-2-oxoheptanedioate aldolase [EC:4.1.2.52]	0.25	2.1E-103	8.0E-103	0.29	
K13421	uridine monophosphate synthetase [EC:2.4.2.10 4.1.1.23]	0.25	2.2E-103	8.4E-103	0.33	
K05350	beta-glucosidase [EC:3.2.1.21]	0.25	2.8E-103	1.1E-102	0.26	yes

Predictor	Description	Pearson's r	P	FDR-adjusted P	Spearman's rho	Associated with fractures (P<0.05)
K04565	superoxide dismutase, Cu-Zn family [EC:1.15.1.1]	0.25	8.8E-103	3.3E-102	0.27	
K12994	O-antigen biosynthesis alpha-1,3-mannosyltransferase [EC:2.4.1.349 2.4.1.-]	0.25	9.5E-103	3.6E-102	0.30	
K07718	two-component system, sensor histidine kinase YesM [EC:2.7.13.3]	0.25	1.1E-102	4.2E-102	0.27	yes
K10855	acetone carboxylase, beta subunit [EC:6.4.1.6]	0.25	1.2E-102	4.5E-102	0.32	yes
K13063	2-amino-4-deoxychorismate synthase [EC:2.6.1.86]	0.25	1.9E-102	7.2E-102	0.29	yes
K13660	beta-1,4-glucosyltransferase [EC:2.4.1.-]	0.25	2.8E-102	1.1E-101	0.32	
K14340	mannosyltransferase [EC:2.4.1.-]	0.25	3.3E-102	1.2E-101	0.30	
K08256	phosphatidyl-myo-inositol alpha-mannosyltransferase [EC:2.4.1.345]	0.25	8.9E-102	3.3E-101	0.27	yes
K03380	phenol 2-monooxygenase (NADPH) [EC:1.14.13.7]	0.25	9.9E-102	3.7E-101	0.30	
K02826	cytochrome aa3-600 menaquinol oxidase subunit II [EC:7.1.1.5]	0.25	1.2E-101	4.5E-101	0.33	yes
K01182	oligo-1,6-glucosidase [EC:3.2.1.10]	0.25	1.3E-101	4.9E-101	0.26	
K07468	putative ATP-dependent DNA ligase [EC:6.5.1.1]	0.25	2.6E-101	9.7E-101	0.33	
K07777	two-component system, NarL family, sensor histidine kinase DegS [EC:2.7.13.3]	0.25	3.9E-101	1.5E-100	0.27	yes
K05603	formimidoylglutamate deiminase [EC:3.5.3.13]	0.25	5.2E-101	1.9E-100	0.30	
K01308	g-D-glutamyl-meso-diaminopimelate peptidase [EC:3.4.19.11]	0.25	7.4E-101	2.8E-100	0.27	yes
K10533	limonene-1,2-epoxide hydrolase [EC:3.3.2.8]	0.25	7.4E-101	2.8E-100	0.29	
K06928	nucleoside-triphosphatase [EC:3.6.1.15]	0.25	1.0E-100	3.7E-100	0.27	yes
K10907	aminotransferase [EC:2.6.1.-]	0.25	1.0E-100	3.7E-100	0.26	
K05551	minimal PKS ketosynthase (KS/KS alpha) [EC:2.3.1.- 2.3.1.260 2.3.1.235]	0.25	1.0E-100	3.7E-100	0.30	
K15983	3-ketosteroid 9alpha-monooxygenase subunit B [EC:1.14.15.30]	0.25	1.7E-100	6.3E-100	0.30	
K03429	processive 1,2-diacylglycerol beta-glucosyltransferase [EC:2.4.1.315]	0.25	2.8E-100	1.0E-99	0.27	
K02858	3,4-dihydroxy 2-butanone 4-phosphate synthase [EC:4.1.99.12]	0.25	3.0E-100	1.1E-99	0.27	
K12255	guanidinobutyrase [EC:3.5.3.7]	0.25	3.5E-100	1.3E-99	0.32	yes
K01208	cyclomaltodextrinase / maltogenic alpha-amylase / neopullulanase [EC:3.2.1.54 3.2.1.133 3.2.1.135]	0.25	5.2E-100	1.9E-99	0.27	yes
K02278	prepilin peptidase CpaA [EC:3.4.23.43]	0.25	5.3E-100	2.0E-99	0.27	yes
K00824	D-alanine transaminase [EC:2.6.1.21]	0.25	5.6E-100	2.1E-99	0.28	
K14257	tetracycline 7-halogenase / FADH2 O2-dependent halogenase [EC:1.14.19.49 1.14.19.-]	0.25	6.5E-100	2.4E-99	0.29	
K10854	acetone carboxylase, alpha subunit [EC:6.4.1.6]	0.25	7.2E-100	2.6E-99	0.31	yes
K09759	nondiscriminating aspartyl-tRNA synthetase [EC:6.1.1.23]	0.25	1.8E-99	6.6E-99	0.27	yes
K00004	(R,R)-butanediol dehydrogenase / meso-butanediol dehydrogenase / diacetyl reductase [EC:1.1.1.4 1.1.1.- 1.1.1.303]	0.25	2.0E-99	7.3E-99	0.27	
K05966	triphosphoribosyl-dephospho-CoA synthase [EC:2.4.2.52]	0.25	2.0E-99	7.3E-99	0.27	
K04757	serine/threonine-protein kinase RsbW [EC:2.7.11.1]	0.25	2.3E-99	8.4E-99	0.27	
K03404	magnesium chelatase subunit D [EC:6.6.1.1]	0.25	2.5E-99	9.1E-99	0.29	
K01916	NAD+ synthase [EC:6.3.1.5]	0.25	2.7E-99	9.8E-99	0.26	
K15866	2-(1,2-epoxy-1,2-dihydrophenyl)acetyl-CoA isomerase [EC:5.3.3.18]	0.25	4.2E-99	1.5E-98	0.29	
K00975	glucose-1-phosphate adenylyltransferase [EC:2.7.7.27]	0.25	2.6E-98	9.5E-98	0.25	
K01273	membrane dipeptidase [EC:3.4.13.19]	0.25	6.4E-98	2.3E-97	0.27	yes
K03271	D-sedoheptulose 7-phosphate isomerase [EC:5.3.1.28]	0.25	1.1E-97	4.0E-97	0.26	
K12992	O-antigen biosynthesis alpha-1,3-rhamnosyltransferase [EC:2.4.1.377]	0.25	2.0E-97	7.2E-97	0.27	yes
K01668	tyrosine phenol-lyase [EC:4.1.99.2]	0.25	2.4E-97	8.7E-97	0.26	yes
K02793	mannose PTS system EIIA component [EC:2.7.1.191]	0.24	1.4E-96	5.1E-96	0.27	
K01750	ornithine cyclodeaminase [EC:4.3.1.12]	0.24	2.0E-96	7.2E-96	0.27	

Predictor	Description	Pearson's r	P	FDR-adjusted P	Spearman's rho	Associated with fractures (P<0.05)
K00974	tRNA nucleotidyltransferase (CCA-adding enzyme) [EC:2.7.7.72 3.1.3.- 3.1.4.-]	0.24	2.2E-96	7.9E-96	0.26	
K13530	AraC family transcriptional regulator, regulatory protein of adaptative response / methylphosphotriester-DNA alkyltransferase methyltransferase [EC:2.1.1.-]	0.24	3.7E-96	1.3E-95	0.25	yes
K07536	2-ketocyclohexanecarboxyl-CoA hydrolase [EC:3.1.2.-]	0.24	1.1E-95	4.0E-95	0.30	yes
K16190	glucuronokinase [EC:2.7.1.43]	0.24	2.0E-95	7.2E-95	0.31	
K01795	mannuronan 5-epimerase [EC:5.1.3.37]	0.24	3.1E-95	1.1E-94	0.30	yes
K00367	ferredoxin-nitrate reductase [EC:1.7.7.2]	0.24	5.4E-95	1.9E-94	0.30	
K01507	inorganic pyrophosphatase [EC:3.6.1.1]	0.24	5.6E-95	2.0E-94	0.26	
K05520	protease I [EC:3.5.1.124]	0.24	6.7E-95	2.4E-94	0.26	
K01224	arabinogalactan endo-1,4-beta-galactosidase [EC:3.2.1.89]	0.24	7.0E-95	2.5E-94	0.26	yes
K13581	modification methylase [EC:2.1.1.72]	0.24	8.8E-95	3.1E-94	0.26	yes
K04099	gallate dioxygenase [EC:1.13.11.57]	0.24	8.9E-95	3.2E-94	0.30	
K05576	NAD(P)H-quinone oxidoreductase subunit 4L [EC:7.1.1.2]	0.24	1.1E-94	3.9E-94	0.30	
K16320	anthranilate 1,2-dioxygenase small subunit [EC:1.14.12.1]	0.24	1.9E-94	6.8E-94	0.31	yes
K04098	hydroxyquinol 1,2-dioxygenase [EC:1.13.11.37]	0.24	3.1E-94	1.1E-93	0.30	
K14667	minimal PKS ketosynthase (KS/KS alpha) [EC:2.3.1.-]	0.24	3.6E-94	1.3E-93	0.29	
K10780	enoyl-[acyl-carrier protein] reductase III [EC:1.3.1.104]	0.24	3.9E-94	1.4E-93	0.31	yes
K01679	fumarate hydratase, class II [EC:4.2.1.2]	0.24	5.5E-94	1.9E-93	0.25	
K14680	RNA ligase [EC:6.5.1.3]	0.24	1.2E-93	4.2E-93	0.31	
K04070	putative pyruvate formate lyase activating enzyme [EC:1.97.1.4]	0.24	1.2E-93	4.2E-93	0.26	
K00620	glutamate N-acetyltransferase / amino-acid N-acetyltransferase [EC:2.3.1.35 2.3.1.1]	0.24	1.6E-93	5.6E-93	0.25	
K02304	precorrin-2 dehydrogenase / sirohydrochlorin ferrochelatase [EC:1.3.1.76 4.99.1.4]	0.24	3.0E-93	1.1E-92	0.27	
K04112	benzoyl-CoA reductase subunit C [EC:1.3.7.8]	0.24	3.7E-93	1.3E-92	0.30	yes
K08258	staphopain A [EC:3.4.22.48]	0.24	3.8E-93	1.3E-92	0.26	yes
K15899	pseudaminic acid cytidylyltransferase [EC:2.7.7.81]	0.24	3.8E-93	1.3E-92	0.26	
K04113	benzoyl-CoA reductase subunit B [EC:1.3.7.8]	0.24	4.7E-93	1.6E-92	0.30	yes
K07141	molybdenum cofactor cytidylyltransferase [EC:2.7.7.76]	0.24	5.3E-93	1.9E-92	0.26	
K00270	phenylalanine dehydrogenase [EC:1.4.1.20]	0.24	5.8E-93	2.0E-92	0.24	yes
K11711	two-component system, LuxR family, sensor histidine kinase DctS [EC:2.7.13.3]	0.24	6.0E-93	2.1E-92	0.30	yes
K01640	hydroxymethylglutaryl-CoA lyase [EC:4.1.3.4]	0.24	6.7E-93	2.3E-92	0.30	
K07587	O-phosphoseryl-tRNA synthetase [EC:6.1.1.27]	0.24	7.3E-93	2.5E-92	0.32	
K00170	pyruvate ferredoxin oxidoreductase beta subunit [EC:1.2.7.1]	0.24	8.4E-93	2.9E-92	0.26	
K01485	cytosine/creatinine deaminase [EC:3.5.4.1 3.5.4.21]	0.24	1.1E-92	3.8E-92	0.26	
K16242	phenol/toluene 2-monooxygenase (NADH) P3/A3 [EC:1.14.13.244 1.14.13.243]	0.24	2.2E-92	7.6E-92	0.31	yes
K00294	1-pyrroline-5-carboxylate dehydrogenase [EC:1.2.1.88]	0.24	2.3E-92	8.0E-92	0.26	
K00995	CDP-diacylglycerol---glycerol-3-phosphate 3-phosphatidyltransferase [EC:2.7.8.5]	0.24	3.1E-92	1.1E-91	0.24	
K08851	TP53 regulating kinase and related kinases [EC:2.7.11.1]	0.24	3.1E-92	1.1E-91	0.31	
K05991	endoglycosylceramidase [EC:3.2.1.123]	0.24	4.8E-92	1.7E-91	0.32	
K02412	flagellum-specific ATP synthase [EC:7.4.2.8]	0.24	6.7E-92	2.3E-91	0.26	
K00003	homoserine dehydrogenase [EC:1.1.1.3]	0.24	6.9E-92	2.4E-91	0.24	
K01721	nitrile hydratase subunit alpha [EC:4.2.1.84]	0.24	8.4E-92	2.9E-91	0.28	
K06013	STE24 endopeptidase [EC:3.4.24.84]	0.24	1.0E-91	3.4E-91	0.26	
K00611	ornithine carbamoyltransferase [EC:2.1.3.3]	0.24	1.2E-91	4.1E-91	0.25	

Predictor	Description	Pearson's r	P	FDR-adjusted P	Spearman's rho	Associated with fractures (P<0.05)
K01406	serralysin [EC:3.4.24.40]	0.24	1.3E-91	4.5E-91	0.28	
K01693	imidazoleglycerol-phosphate dehydratase [EC:4.2.1.19]	0.24	1.5E-91	5.1E-91	0.24	
K12420	ketoreductase [EC:1.1.1.-]	0.24	1.7E-91	5.8E-91	0.29	yes
K01571	oxaloacetate decarboxylase (Na+ extruding) subunit alpha [EC:7.2.4.2]	0.24	2.2E-91	7.5E-91	0.25	
K13005	O-antigen biosynthesis alpha-1,3-abequosyltransferase [EC:2.4.1.60]	0.24	2.2E-91	7.5E-91	0.31	
K00626	acetyl-CoA C-acetyltransferase [EC:2.3.1.9]	0.24	2.5E-91	8.5E-91	0.25	
K16421	4-hydroxymandelate synthase [EC:1.13.11.46]	0.24	2.6E-91	8.9E-91	0.30	yes
K03851	taurine-pyruvate aminotransferase [EC:2.6.1.77]	0.24	3.3E-91	1.1E-90	0.31	yes
K01356	repressor LexA [EC:3.4.21.88]	0.24	4.5E-91	1.5E-90	0.24	
K01845	glutamate-1-semialdehyde 2,1-aminomutase [EC:5.4.3.8]	0.24	1.0E-90	3.4E-90	0.25	
K06183	16S rRNA pseudouridine516 synthase [EC:5.4.99.19]	0.24	1.2E-90	4.1E-90	0.24	
K00596	2,2-dialkylglycine decarboxylase (pyruvate) [EC:4.1.1.64]	0.24	1.5E-90	5.1E-90	0.26	yes
K01167	ribonuclease T1 [EC:4.6.1.24]	0.24	1.7E-90	5.8E-90	0.30	
K15511	benzoyl-CoA 2,3-epoxidase subunit A [EC:1.14.13.208]	0.24	1.9E-90	6.4E-90	0.32	yes
K11357	two-component system, cell cycle sensor histidine kinase DivJ [EC:2.7.13.3]	0.24	1.9E-90	6.4E-90	0.31	yes
K15669	D-glycero-alpha-D-manno-heptose 1-phosphate guanylyltransferase [EC:2.7.7.71]	0.24	3.1E-90	1.0E-89	0.25	yes
K06375	stage 0 sporulation protein B (sporulation initiation phosphotransferase) [EC:2.7.-.-]	0.24	5.6E-90	1.9E-89	0.31	yes
K03379	cyclohexanone monooxygenase [EC:1.14.13.22]	0.24	9.8E-90	3.3E-89	0.29	
K03407	two-component system, chemotaxis family, sensor kinase CheA [EC:2.7.13.3]	0.24	1.0E-89	3.4E-89	0.26	
K06182	23S rRNA pseudouridine2604 synthase [EC:5.4.99.21]	0.24	1.0E-89	3.4E-89	0.25	
K02828	cytochrome aa3-600 menaquinol oxidase subunit III [EC:7.1.1.5]	0.24	2.4E-89	8.1E-89	0.31	yes
K15897	UDP-2,4-diacetamido-2,4,6-trideoxy-beta-L-altropyranose hydrolase [EC:3.6.1.57]	0.24	4.1E-89	1.4E-88	0.31	
K03789	[ribosomal protein S18]-alanine N-acetyltransferase [EC:2.3.1.266]	0.23	1.2E-88	4.0E-88	0.23	
K14189	uncharacterized oxidoreductase [EC:1.-.-.-]	0.23	1.5E-88	5.0E-88	0.26	
K03273	D-glycero-D-manno-heptose 1,7-bisphosphate phosphatase [EC:3.1.3.82 3.1.3.83]	0.23	1.9E-88	6.3E-88	0.25	
K03621	phosphate acyltransferase [EC:2.3.1.274]	0.23	2.1E-88	7.0E-88	0.24	
K11444	two-component system, chemotaxis family, response regulator WspR [EC:2.7.7.65]	0.23	2.4E-88	8.0E-88	0.28	yes
K03750	molybdopterin molybdotransferase [EC:2.10.1.1]	0.23	2.8E-88	9.3E-88	0.25	
K03735	ethanolamine ammonia-lyase large subunit [EC:4.3.1.7]	0.23	2.8E-88	9.3E-88	0.25	
K16319	anthranilate 1,2-dioxygenase large subunit [EC:1.14.12.1]	0.23	3.1E-88	1.0E-87	0.30	yes
K11181	dissimilatory sulfite reductase beta subunit [EC:1.8.99.5]	0.23	3.3E-88	1.1E-87	0.25	
K05597	glutamin-(asparagin-)ase [EC:3.5.1.38]	0.23	3.4E-88	1.1E-87	0.30	
K00575	chemotaxis protein methyltransferase CheR [EC:2.1.1.80]	0.23	5.5E-88	1.8E-87	0.25	
K14733	limonene 1,2-monooxygenase [EC:1.14.13.107]	0.23	8.6E-88	2.8E-87	0.28	yes
K16651	L-threonine kinase [EC:2.7.1.177]	0.23	1.1E-87	3.6E-87	0.27	
K04517	prephenate dehydrogenase [EC:1.3.1.12]	0.23	1.1E-87	3.6E-87	0.24	
K04114	benzoyl-CoA reductase subunit A [EC:1.3.7.8]	0.23	2.7E-87	8.9E-87	0.29	yes
K04115	benzoyl-CoA reductase subunit D [EC:1.3.7.8]	0.23	2.7E-87	8.9E-87	0.29	yes
K10811	thiamine pyridinylase [EC:2.5.1.2]	0.23	3.1E-87	1.0E-86	0.30	
K01233	chitosanase [EC:3.2.1.132]	0.23	3.3E-87	1.1E-86	0.24	yes
K00917	tagatose 6-phosphate kinase [EC:2.7.1.144]	0.23	4.5E-87	1.5E-86	0.26	
K14028	methanol dehydrogenase (cytochrome c) subunit 1 [EC:1.1.2.7]	0.23	4.7E-87	1.5E-86	0.29	

Predictor	Description	Pearson's r	P	FDR-adjusted P	Spearman's rho	Associated with fractures (P<0.05)
K00662	aminoglycoside 3-N-acetyltransferase [EC:2.3.1.81]	0.23	9.7E-87	3.2E-86	0.25	yes
K15531	oligosaccharide reducing-end xylanase [EC:3.2.1.156]	0.23	1.9E-86	6.2E-86	0.23	yes
K08068	UDP-N-acetylglucosamine 2-epimerase (hydrolysing) [EC:3.2.1.183]	0.23	2.0E-86	6.5E-86	0.30	yes
K00899	5-methylthioribose kinase [EC:2.7.1.100]	0.23	2.3E-86	7.5E-86	0.25	
K13979	alcohol dehydrogenase (NADP+) [EC:1.1.1.2]	0.23	3.9E-86	1.3E-85	0.27	
K02434	aspartyl-tRNA(Asn)/glutamyl-tRNA(Gln) amidotransferase subunit B [EC:6.3.5.6 6.3.5.7]	0.23	5.7E-86	1.9E-85	0.24	
K15566	tRNA (adenine9-N1/guanine9-N1)-methyltransferase [EC:2.1.1.218 2.1.1.221]	0.23	8.3E-86	2.7E-85	0.27	
K01255	leucyl aminopeptidase [EC:3.4.11.1]	0.23	8.9E-86	2.9E-85	0.27	
K15792	MurE/MurF fusion protein [EC:6.3.2.13 6.3.2.10]	0.23	1.2E-85	3.9E-85	0.29	yes
K10212	glycosyl-4,4'-diaponeurosporenoate acyltransferase [EC:2.3.1.-]	0.23	1.4E-85	4.5E-85	0.28	
K10794	D-proline reductase (dithiol) PrdB [EC:1.21.4.1]	0.23	1.7E-85	5.5E-85	0.28	
K13380	NADH-quinone oxidoreductase subunit B/C/D [EC:7.1.1.2]	0.23	2.3E-85	7.4E-85	0.30	
K01494	dCTP deaminase [EC:3.5.4.13]	0.23	3.6E-85	1.2E-84	0.24	
K07258	serine-type D-Ala-D-Ala carboxypeptidase (penicillin-binding protein 5/6) [EC:3.4.16.4]	0.23	4.2E-85	1.4E-84	0.25	
K14748	ethylbenzene dioxygenase subunit alpha [EC:1.14.12.-]	0.23	4.3E-85	1.4E-84	0.31	yes
K14749	ethylbenzene dioxygenase subunit beta [EC:1.14.12.-]	0.23	4.3E-85	1.4E-84	0.31	yes
K14974	6-hydroxynicotinate 3-monooxygenase [EC:1.14.13.114]	0.23	4.8E-85	1.5E-84	0.31	
K10026	7-carboxy-7-deazaguanine synthase [EC:4.3.99.3]	0.23	5.7E-85	1.8E-84	0.25	
K11751	5'-nucleotidase / UDP-sugar diphosphatase [EC:3.1.3.5 3.6.1.45]	0.23	1.0E-84	3.2E-84	0.25	
K01014	aryl sulfotransferase [EC:2.8.2.1]	0.23	1.5E-84	4.8E-84	0.31	yes
K15896	UDP-4-amino-4,6-dideoxy-N-acetyl-beta-L-altrosamine N-acetyltransferase [EC:2.3.1.202]	0.23	1.5E-84	4.8E-84	0.28	
K12132	eukaryotic-like serine/threonine-protein kinase [EC:2.7.11.1]	0.23	1.6E-84	5.1E-84	0.23	
K15918	D-glycerate 3-kinase [EC:2.7.1.31]	0.23	1.8E-84	5.8E-84	0.32	
K10742	DNA replication ATP-dependent helicase Dna2 [EC:3.6.4.12]	0.23	1.8E-84	5.8E-84	0.25	
K04117	cyclohexanecarboxyl-CoA dehydrogenase [EC:1.3.99.-]	0.23	4.5E-84	1.4E-83	0.29	yes
K00111	glycerol-3-phosphate dehydrogenase [EC:1.1.5.3]	0.23	4.9E-84	1.6E-83	0.24	
K08260	adenosylcobinamide hydrolase [EC:3.5.1.90]	0.23	5.2E-84	1.7E-83	0.30	
K03856	3-deoxy-7-phosphoheptulonate synthase [EC:2.5.1.54]	0.23	5.3E-84	1.7E-83	0.24	
K16647	arabinofuranan 3-O-arabinosyltransferase [EC:2.4.2.47]	0.23	5.4E-84	1.7E-83	0.28	yes
K00332	NADH-quinone oxidoreductase subunit C [EC:7.1.1.2]	0.23	6.0E-84	1.9E-83	0.25	
K03500	16S rRNA (cytosine967-C5)-methyltransferase [EC:2.1.1.176]	0.23	7.7E-84	2.4E-83	0.24	
K08681	pyridoxal 5'-phosphate synthase pdxT subunit [EC:4.3.3.6]	0.23	9.9E-84	3.1E-83	0.25	
K00461	arachidonate 5-lipoxygenase [EC:1.13.11.34]	0.23	1.0E-83	3.2E-83	0.30	
K01663	imidazole glycerol-phosphate synthase [EC:4.3.2.10]	0.23	1.1E-83	3.5E-83	0.30	
K13798	DNA-directed RNA polymerase subunit B [EC:2.7.7.6]	0.23	2.0E-83	6.3E-83	0.27	
K04108	4-hydroxybenzoyl-CoA reductase subunit alpha [EC:1.1.7.1]	0.23	2.1E-83	6.6E-83	0.29	yes
K00333	NADH-quinone oxidoreductase subunit D [EC:7.1.1.2]	0.23	2.1E-83	6.6E-83	0.25	
K04042	bifunctional UDP-N-acetylglucosamine pyrophosphorylase / glucosamine-1-phosphate N-acetyltransferase [EC:2.7.7.23 2.3.1.157]	0.23	2.6E-83	8.2E-83	0.23	
K01214	isoamylase [EC:3.2.1.68]	0.23	3.2E-83	1.0E-82	0.23	
K05341	amylosucrase [EC:2.4.1.4]	0.23	3.3E-83	1.0E-82	0.24	yes
K14630	two-component flavin-dependent monooxygenase [EC:1.14.14.-]	0.23	4.2E-83	1.3E-82	0.26	
K03769	peptidyl-prolyl cis-trans isomerase C [EC:5.2.1.8]	0.23	4.4E-83	1.4E-82	0.26	

Predictor	Description	Pearson's r	P	FDR-adjusted P	Spearman's rho	Associated with fractures (P<0.05)
K08600	sortase B [EC:3.4.22.71]	0.23	5.0E-83	1.6E-82	0.25	
K16044	scyllo-inositol 2-dehydrogenase (NADP+) [EC:1.1.1.371]	0.23	5.9E-83	1.8E-82	0.28	
K03343	putrescine oxidase [EC:1.4.3.10]	0.23	9.5E-83	3.0E-82	0.28	
K02435	aspartyl-tRNA(Asn)/glutamyl-tRNA(Gln) amidotransferase subunit C [EC:6.3.5.6 6.3.5.7]	0.23	1.5E-82	4.7E-82	0.23	
K03388	heterodisulfide reductase subunit A2 [EC:1.8.7.3 1.8.98.4 1.8.98.5 1.8.98.6]	0.23	2.3E-82	7.2E-82	0.26	
K13687	arabinofuranosyltransferase [EC:2.4.2.-]	0.23	2.4E-82	7.5E-82	0.31	
K15904	bifunctional N6-L-threonylcarbamoyladenine synthase / protein kinase Bud32 [EC:2.3.1.234 2.7.11.1]	0.23	2.4E-82	7.5E-82	0.30	
K13019	UDP-GlcNAc3NAcA epimerase [EC:5.1.3.23]	0.23	2.5E-82	7.8E-82	0.25	
K02654	leader peptidase (prepilin peptidase) / N-methyltransferase [EC:3.4.23.43 2.1.1.-]	0.23	3.7E-82	1.1E-81	0.24	
K10441	ribose transport system ATP-binding protein [EC:7.5.2.7]	0.23	3.7E-82	1.1E-81	0.25	
K14029	methanol dehydrogenase (cytochrome c) subunit 2 [EC:1.1.2.7]	0.23	3.9E-82	1.2E-81	0.29	
K07533	foldase protein PrsA [EC:5.2.1.8]	0.23	4.1E-82	1.3E-81	0.24	
K01207	beta-N-acetylhexosaminidase [EC:3.2.1.52]	0.23	4.6E-82	1.4E-81	0.23	
K07680	two-component system, NarL family, sensor histidine kinase ComP [EC:2.7.13.3]	0.23	5.9E-82	1.8E-81	0.28	yes
K13940	dihydroneopterin aldolase / 2-amino-4-hydroxy-6-hydroxymethyldihydropteridine diphosphokinase [EC:4.1.2.25 2.7.6.3]	0.23	6.4E-82	2.0E-81	0.22	
K00878	hydroxyethylthiazole kinase [EC:2.7.1.50]	0.23	7.9E-82	2.4E-81	0.23	
K15734	all-trans-retinol dehydrogenase (NAD+) [EC:1.1.1.105]	0.23	9.0E-82	2.8E-81	0.25	
K01850	chorismate mutase [EC:5.4.99.5]	0.23	9.0E-82	2.8E-81	0.25	
K02473	UDP-N-acetylglucosamine/UDP-N-acetylgalactosamine 4-epimerase [EC:5.1.3.7 5.1.3.-]	0.23	9.3E-82	2.9E-81	0.25	yes
K03389	heterodisulfide reductase subunit B2 [EC:1.8.7.3 1.8.98.4 1.8.98.5 1.8.98.6]	0.23	1.3E-81	4.0E-81	0.25	
K15981	cholest-4-en-3-one 26-monooxygenase [EC:1.14.15.29]	0.23	1.5E-81	4.6E-81	0.27	
K15652	3-dehydroshikimate dehydratase [EC:4.2.1.118]	0.22	1.6E-81	4.9E-81	0.30	yes
K13040	two-component system, LuxR family, sensor histidine kinase TtrS [EC:2.7.13.3]	0.22	1.7E-81	5.2E-81	0.26	
K02028	polar amino acid transport system ATP-binding protein [EC:7.4.2.1]	0.22	1.8E-81	5.5E-81	0.23	
K01698	porphobilinogen synthase [EC:4.2.1.24]	0.22	2.4E-81	7.3E-81	0.24	
K14268	5-aminovalerate/4-aminobutyrate aminotransferase [EC:2.6.1.48 2.6.1.19]	0.22	2.5E-81	7.6E-81	0.28	yes
K13018	UDP-2-acetamido-3-amino-2,3-dideoxy-glucuronate N-acetyltransferase [EC:2.3.1.201]	0.22	2.5E-81	7.6E-81	0.25	
K00131	glyceraldehyde-3-phosphate dehydrogenase (NADP+) [EC:1.2.1.9]	0.22	2.9E-81	8.8E-81	0.25	
K11389	glyceraldehyde-3-phosphate dehydrogenase (ferredoxin) [EC:1.2.7.6]	0.22	3.7E-81	1.1E-80	0.30	yes
K08315	hydrogenase 3 maturation protease [EC:3.4.23.51]	0.22	5.6E-81	1.7E-80	0.24	
K08591	acyl phosphate:glycerol-3-phosphate acyltransferase [EC:2.3.1.275]	0.22	7.4E-81	2.2E-80	0.23	
K01560	2-haloacid dehalogenase [EC:3.8.1.2]	0.22	8.3E-81	2.5E-80	0.24	
K07284	sortase A [EC:3.4.22.70]	0.22	1.0E-80	3.0E-80	0.23	yes
K01034	acetate CoA/acetoacetate CoA-transferase alpha subunit [EC:2.8.3.8 2.8.3.9]	0.22	1.5E-80	4.5E-80	0.25	
K14940	gamma-F420-2:alpha-L-glutamate ligase [EC:6.3.2.32]	0.22	1.6E-80	4.8E-80	0.30	
K14668	minimal PKS chain-length factor (CLF/KS beta) [EC:2.3.1.-]	0.22	1.9E-80	5.7E-80	0.28	
K06868	Sep-tRNA:Cys-tRNA synthetase [EC:2.5.1.73]	0.22	2.3E-80	6.9E-80	0.30	
K01040	glutaconate CoA-transferase, subunit B [EC:2.8.3.12]	0.22	2.5E-80	7.5E-80	0.29	
K03841	fructose-1,6-bisphosphatase I [EC:3.1.3.11]	0.22	2.7E-80	8.1E-80	0.25	
K01026	propionate CoA-transferase [EC:2.8.3.1]	0.22	2.8E-80	8.4E-80	0.25	
K00035	D-galactose 1-dehydrogenase [EC:1.1.1.48]	0.22	2.9E-80	8.7E-80	0.29	
K07404	6-phosphogluconolactonase [EC:3.1.1.31]	0.22	3.1E-80	9.3E-80	0.24	

Predictor	Description	Pearson's r	P	FDR-adjusted P	Spearman's rho	Associated with fractures (P<0.05)
K02802	NA	0.22	3.6E-80	1.1E-79	0.27	
K02609	ring-1,2-phenylacetyl-CoA epoxidase subunit PaaA [EC:1.14.13.149]	0.22	4.6E-80	1.4E-79	0.27	
K01816	hydroxypyruvate isomerase [EC:5.3.1.22]	0.22	6.4E-80	1.9E-79	0.27	
K11915	serine/threonine protein phosphatase Stp1 [EC:3.1.3.16]	0.22	6.9E-80	2.1E-79	0.28	yes
K13292	phosphatidylglycerol---prolipoprotein diacylglyceryl transferase [EC:2.5.1.145]	0.22	1.3E-79	3.9E-79	0.23	
K10216	2-hydroxymuconate-semialdehyde hydrolase [EC:3.7.1.9]	0.22	1.5E-79	4.5E-79	0.29	
K02182	carnitine-CoA ligase [EC:6.2.1.48]	0.22	1.8E-79	5.4E-79	0.24	
K05296	3(or 17)beta-hydroxysteroid dehydrogenase [EC:1.1.1.51]	0.22	2.1E-79	6.3E-79	0.30	
K00841	aminotransferase [EC:2.6.1.-]	0.22	3.6E-79	1.1E-78	0.25	
K14170	chorismate mutase / prephenate dehydratase [EC:5.4.99.5 4.2.1.51]	0.22	3.7E-79	1.1E-78	0.22	
K01844	beta-lysine 5,6-aminomutase alpha subunit [EC:5.4.3.3]	0.22	4.2E-79	1.2E-78	0.25	
K03736	ethanolamine ammonia-lyase small subunit [EC:4.3.1.7]	0.22	6.3E-79	1.9E-78	0.24	
K00103	L-gulonolactone oxidase [EC:1.1.3.8]	0.22	8.3E-79	2.5E-78	0.30	
K12960	5-methylthioadenosine/S-adenosylhomocysteine deaminase [EC:3.5.4.31 3.5.4.28]	0.22	8.8E-79	2.6E-78	0.24	
K15357	N-formylmaleamate deformylase [EC:3.5.1.106]	0.22	1.0E-78	3.0E-78	0.30	
K02433	aspartyl-tRNA(Asn)/glutamyl-tRNA(Gln) amidotransferase subunit A [EC:6.3.5.6 6.3.5.7]	0.22	2.7E-78	8.0E-78	0.22	
K00316	spermidine dehydrogenase [EC:1.5.99.6]	0.22	3.6E-78	1.1E-77	0.29	
K01259	proline iminopeptidase [EC:3.4.11.5]	0.22	4.8E-78	1.4E-77	0.22	
K02829	cytochrome aa3-600 menaquinol oxidase subunit IV [EC:7.1.1.5]	0.22	5.9E-78	1.7E-77	0.30	yes
K01669	deoxyribodipyrimidine photo-lyase [EC:4.1.99.3]	0.22	6.5E-78	1.9E-77	0.26	
K00040	fructuronate reductase [EC:1.1.1.57]	0.22	8.6E-78	2.5E-77	0.24	
K00355	NAD(P)H dehydrogenase (quinone) [EC:1.6.5.2]	0.22	1.3E-77	3.8E-77	0.24	
K00573	protein-L-isoaspartate(D-aspartate) O-methyltransferase [EC:2.1.1.77]	0.22	1.4E-77	4.1E-77	0.26	
K15228	methylamine dehydrogenase light chain [EC:1.4.9.1]	0.22	1.4E-77	4.1E-77	0.29	
K02558	UDP-N-acetylmuramate: L-alanyl-gamma-D-glutamyl-meso-diaminopimelate ligase [EC:6.3.2.45]	0.22	1.9E-77	5.6E-77	0.24	
K00135	succinate-semialdehyde dehydrogenase / glutarate-semialdehyde dehydrogenase [EC:1.2.1.16 1.2.1.79 1.2.1.20]	0.22	2.0E-77	5.9E-77	0.24	
K01267	aspartyl aminopeptidase [EC:3.4.11.21]	0.22	2.6E-77	7.6E-77	0.23	
K10676	2,4-dichlorophenol 6-monooxygenase [EC:1.14.13.20]	0.22	3.2E-77	9.4E-77	0.28	
K01523	phosphoribosyl-ATP pyrophosphohydrolase [EC:3.6.1.31]	0.22	4.0E-77	1.2E-76	0.22	
K00020	3-hydroxyisobutyrate dehydrogenase [EC:1.1.1.31]	0.22	4.2E-77	1.2E-76	0.23	
K00366	ferredoxin-nitrite reductase [EC:1.7.7.1]	0.22	4.9E-77	1.4E-76	0.23	
K01101	4-nitrophenyl phosphatase [EC:3.1.3.41]	0.22	6.5E-77	1.9E-76	0.25	
K00943	dTMP kinase [EC:2.7.4.9]	0.22	6.5E-77	1.9E-76	0.22	
K01496	phosphoribosyl-AMP cyclohydrolase [EC:3.5.4.19]	0.22	7.4E-77	2.2E-76	0.23	
K01436	amidohydrolase [EC:3.5.1.-]	0.22	1.1E-76	3.2E-76	0.21	
K03707	thiaminase (transcriptional activator TenA) [EC:3.5.99.2]	0.22	1.6E-76	4.6E-76	0.22	
K00514	zeta-carotene desaturase [EC:1.3.5.6]	0.22	1.7E-76	4.9E-76	0.29	yes
K11210	metallothiol transferase [EC:2.5.1.-]	0.22	2.5E-76	7.2E-76	0.31	yes
K15229	methylamine dehydrogenase heavy chain [EC:1.4.9.1]	0.22	3.0E-76	8.7E-76	0.28	
K04094	methylenetetrahydrofolate--tRNA-(uracil-5-)-methyltransferase [EC:2.1.1.74]	0.22	4.8E-76	1.4E-75	0.23	
K01741	DNA-(apurinic or apyrimidinic site) lyase [EC:4.2.99.18]	0.22	5.3E-76	1.5E-75	0.26	
K15232	citryl-CoA synthetase large subunit [EC:6.2.1.18]	0.22	6.4E-76	1.8E-75	0.26	

Predictor	Description	Pearson's r	P	FDR-adjusted P	Spearman's rho	Associated with fractures (P<0.05)
K00674	2,3,4,5-tetrahydropyridine-2,6-dicarboxylate N-succinyltransferase [EC:2.3.1.117]	0.22	7.7E-76	2.2E-75	0.23	
K10542	methyl-galactoside transport system ATP-binding protein [EC:7.5.2.11]	0.22	9.7E-76	2.8E-75	0.23	
K03722	ATP-dependent DNA helicase DinG [EC:3.6.4.12]	0.22	1.1E-75	3.2E-75	0.22	
K05917	sterol 14alpha-demethylase [EC:1.14.14.154 1.14.15.36]	0.22	1.4E-75	4.0E-75	0.28	
K03060	DNA-directed RNA polymerase subunit omega [EC:2.7.7.6]	0.22	2.0E-75	5.7E-75	0.22	
K01480	agmatinase [EC:3.5.3.11]	0.22	2.2E-75	6.3E-75	0.23	
K02611	ring-1,2-phenylacetyl-CoA epoxidase subunit PaaC [EC:1.14.13.149]	0.22	2.5E-75	7.2E-75	0.26	
K00965	UDPglucose--hexose-1-phosphate uridylyltransferase [EC:2.7.7.12]	0.22	3.7E-75	1.1E-74	0.22	
K00966	mannose-1-phosphate guanylyltransferase [EC:2.7.7.13]	0.22	4.4E-75	1.3E-74	0.23	
K10211	4,4'-diaponeurosporenoate glycosyltransferase [EC:2.4.1.-]	0.22	4.4E-75	1.3E-74	0.23	
K01958	pyruvate carboxylase [EC:6.4.1.1]	0.22	4.8E-75	1.4E-74	0.25	
K01786	NA	0.22	5.2E-75	1.5E-74	0.22	
K07652	two-component system, OmpR family, sensor histidine kinase VicK [EC:2.7.13.3]	0.22	5.2E-75	1.5E-74	0.23	
K09693	teichoic acid transport system ATP-binding protein [EC:7.5.2.4]	0.22	5.6E-75	1.6E-74	0.22	
K01851	salicylate biosynthesis isochorismate synthase [EC:5.4.4.2]	0.22	1.6E-74	4.5E-74	0.28	
K06215	pyridoxal 5'-phosphate synthase pdxS subunit [EC:4.3.3.6]	0.22	1.8E-74	5.1E-74	0.23	
K11780	7,8-didemethyl-8-hydroxy-5-deazariboflavin synthase [EC:4.3.1.32]	0.21	2.0E-74	5.7E-74	0.30	yes
K02056	simple sugar transport system ATP-binding protein [EC:7.5.2.-]	0.21	2.0E-74	5.7E-74	0.23	
K03390	heterodisulfide reductase subunit C2 [EC:1.8.7.3 1.8.98.4 1.8.98.5 1.8.98.6]	0.21	3.0E-74	8.5E-74	0.24	
K03929	para-nitrobenzyl esterase [EC:3.1.1.-]	0.21	3.3E-74	9.3E-74	0.21	
K11386	arabinosyltransferase B [EC:2.4.2.-]	0.21	3.9E-74	1.1E-73	0.27	
K00996	undecaprenyl-phosphate galactose phosphotransferase [EC:2.7.8.6]	0.21	4.9E-74	1.4E-73	0.25	
K08692	malate-CoA ligase subunit alpha [EC:6.2.1.9]	0.21	5.0E-74	1.4E-73	0.28	
K00681	gamma-glutamyltranspeptidase / glutathione hydrolase [EC:2.3.2.2 3.4.19.13]	0.21	7.3E-74	2.1E-73	0.25	
K13714	bifunctional autolysin [EC:3.5.1.28 3.2.1.96]	0.21	7.4E-74	2.1E-73	0.22	
K00955	bifunctional enzyme CysN/CysC [EC:2.7.7.4 2.7.1.25]	0.21	8.5E-74	2.4E-73	0.23	
K03928	carboxylesterase [EC:3.1.1.1]	0.21	8.8E-74	2.5E-73	0.28	
K02759	cellobiose PTS system EIIA component [EC:2.7.1.196 2.7.1.205]	0.21	1.2E-73	3.4E-73	0.22	
K01035	acetate CoA/acetoacetate CoA-transferase beta subunit [EC:2.8.3.8 2.8.3.9]	0.21	1.4E-73	3.9E-73	0.24	
K04072	acetaldehyde dehydrogenase / alcohol dehydrogenase [EC:1.2.1.10 1.1.1.1]	0.21	1.5E-73	4.2E-73	0.21	
K13016	UDP-N-acetyl-2-amino-2-deoxyglucuronate dehydrogenase [EC:1.1.1.335]	0.21	2.2E-73	6.2E-73	0.25	
K01823	isopentenyl-diphosphate Delta-isomerase [EC:5.3.3.2]	0.21	2.9E-73	8.1E-73	0.22	
K01253	microsomal epoxide hydrolase [EC:3.3.2.9]	0.21	4.3E-73	1.2E-72	0.27	
K13602	bacteriochlorophyllide d C-12(1)-methyltransferase [EC:2.1.1.331]	0.21	4.7E-73	1.3E-72	0.29	
K08299	crotonobetainyl-CoA hydratase [EC:4.2.1.149]	0.21	5.8E-73	1.6E-72	0.23	
K01142	exodeoxyribonuclease III [EC:3.1.11.2]	0.21	6.3E-73	1.8E-72	0.21	
K00039	ribitol 2-dehydrogenase [EC:1.1.1.56]	0.21	8.3E-73	2.3E-72	0.28	
K01048	lysophospholipase [EC:3.1.1.5]	0.21	8.9E-73	2.5E-72	0.21	
K00762	orotate phosphoribosyltransferase [EC:2.4.2.10]	0.21	1.4E-72	3.9E-72	0.26	
K00335	NADH-quinone oxidoreductase subunit F [EC:7.1.1.2]	0.21	1.4E-72	3.9E-72	0.22	
K14665	amidohydrolase [EC:3.5.1.-]	0.21	2.5E-72	6.9E-72	0.28	
K00933	creatine kinase [EC:2.7.3.2]	0.21	3.2E-72	8.9E-72	0.28	yes

Predictor	Description	Pearson's r	P	FDR-adjusted P	Spearman's rho	Associated with fractures (P<0.05)
K07546	E-phenylitaconyl-CoA hydratase [EC:4.2.1.-]	0.21	4.0E-72	1.1E-71	0.27	yes
K13776	citronellyl-CoA synthetase [EC:6.2.1.-]	0.21	4.6E-72	1.3E-71	0.27	
K01198	xylan 1,4-beta-xylosidase [EC:3.2.1.37]	0.21	7.3E-72	2.0E-71	0.21	
K15785	L-2,4-diaminobutyrate transaminase [EC:2.6.1.76]	0.21	1.2E-71	3.3E-71	0.30	
K00065	2-dehydro-3-deoxy-D-gluconate 5-dehydrogenase [EC:1.1.1.127]	0.21	1.5E-71	4.1E-71	0.24	
K00362	nitrite reductase (NADH) large subunit [EC:1.7.1.15]	0.21	1.9E-71	5.2E-71	0.25	
K01263	NA	0.21	2.7E-71	7.4E-71	0.27	
K03790	[ribosomal protein S5]-alanine N-acetyltransferase [EC:2.3.1.267]	0.21	3.8E-71	1.0E-70	0.22	
K00627	pyruvate dehydrogenase E2 component (dihydrolipoamide acetyltransferase) [EC:2.3.1.12]	0.21	4.3E-71	1.2E-70	0.22	
K04787	mycobactin salicyl-AMP ligase [EC:6.3.2.-]	0.21	5.5E-71	1.5E-70	0.28	
K00293	saccharopine dehydrogenase (NADP+, L-glutamate forming) [EC:1.5.1.10]	0.21	6.1E-71	1.7E-70	0.22	
K00334	NADH-quinone oxidoreductase subunit E [EC:7.1.1.2]	0.21	6.6E-71	1.8E-70	0.22	
K01705	homoaconitate hydratase [EC:4.2.1.36]	0.21	7.4E-71	2.0E-70	0.27	yes
K00221	alkylmercury lyase [EC:4.99.1.2]	0.21	8.1E-71	2.2E-70	0.28	
K01749	hydroxymethylbilane synthase [EC:2.5.1.61]	0.21	8.6E-71	2.4E-70	0.22	
K03077	L-ribulose-5-phosphate 4-epimerase [EC:5.1.3.4]	0.21	8.9E-71	2.4E-70	0.24	
K01439	succinyl-diaminopimelate desuccinylase [EC:3.5.1.18]	0.21	9.5E-71	2.6E-70	0.21	
K00113	glycerol-3-phosphate dehydrogenase subunit C	0.21	9.9E-71	2.7E-70	0.24	
K01058	phospholipase A1/A2 [EC:3.1.1.32 3.1.1.4]	0.21	1.0E-70	2.7E-70	0.24	
K02641	ferredoxin--NADP+ reductase [EC:1.18.1.2]	0.21	1.1E-70	3.0E-70	0.29	
K03339	6-phospho-5-dehydro-2-deoxy-D-gluconate aldolase [EC:4.1.2.29]	0.21	1.4E-70	3.8E-70	0.25	
K08095	cutinase [EC:3.1.1.74]	0.21	1.5E-70	4.1E-70	0.25	
K00112	glycerol-3-phosphate dehydrogenase subunit B [EC:1.1.5.3]	0.21	1.7E-70	4.6E-70	0.24	
K06879	7-cyano-7-deazaguanine reductase [EC:1.7.1.13]	0.21	1.8E-70	4.9E-70	0.24	
K15450	tRNA wybutosine-synthesizing protein 3 [EC:2.1.1.282]	0.21	2.2E-70	6.0E-70	0.23	
K00016	L-lactate dehydrogenase [EC:1.1.1.27]	0.21	2.7E-70	7.3E-70	0.21	
K03382	hydroxydechloroatrazine ethylaminohydrolase [EC:3.5.4.43]	0.21	2.7E-70	7.3E-70	0.22	yes
K00213	7-dehydrocholesterol reductase [EC:1.3.1.21]	0.21	4.2E-70	1.1E-69	0.21	
K07442	tRNA (adenine57-N1/adenine58-N1)-methyltransferase catalytic subunit [EC:2.1.1.219 2.1.1.220]	0.21	1.1E-69	3.0E-69	0.20	
K14205	phosphatidylglycerol lysyltransferase [EC:2.3.2.3]	0.21	1.2E-69	3.2E-69	0.23	
K06442	23S rRNA (cytidine1920-2'-O)/16S rRNA (cytidine1409-2'-O)-methyltransferase [EC:2.1.1.226 2.1.1.227]	0.21	1.3E-69	3.5E-69	0.21	
K02486	two-component system, sensor kinase [EC:2.7.13.3]	0.21	1.5E-69	4.0E-69	0.21	
K07305	peptide-methionine (R)-S-oxide reductase [EC:1.8.4.12]	0.21	1.6E-69	4.3E-69	0.24	
K04105	4-hydroxybenzoate-CoA ligase [EC:6.2.1.27 6.2.1.25]	0.21	1.8E-69	4.8E-69	0.27	
K00800	3-phosphoshikimate 1-carboxyvinyltransferase [EC:2.5.1.19]	0.21	2.2E-69	5.9E-69	0.20	
K00862	erythritol kinase (D-erythritol 1-phosphate-forming) [EC:2.7.1.215]	0.21	2.4E-69	6.4E-69	0.28	
K00882	1-phosphofructokinase [EC:2.7.1.56]	0.21	2.4E-69	6.4E-69	0.21	
K01008	selenide, water dikinase [EC:2.7.9.3]	0.21	2.7E-69	7.2E-69	0.24	
K01874	methionyl-tRNA synthetase [EC:6.1.1.10]	0.21	2.9E-69	7.8E-69	0.26	
K13935	malonate decarboxylase epsilon subunit [EC:2.3.1.39]	0.21	3.3E-69	8.8E-69	0.25	
K05956	geranylgeranyl transferase type-2 subunit beta [EC:2.5.1.60]	0.21	4.0E-69	1.1E-68	0.18	
K07816	GTP pyrophosphokinase [EC:2.7.6.5]	0.21	4.7E-69	1.3E-68	0.21	

Predictor	Description	Pearson's r	P	FDR-adjusted P	Spearman's rho	Associated with fractures (P<0.05)
K01539	sodium/potassium-transporting ATPase subunit alpha [EC:7.2.2.13]	0.21	5.4E-69	1.4E-68	0.28	
K14338	cytochrome P450 / NADPH-cytochrome P450 reductase [EC:1.14.14.1 1.6.2.4]	0.21	6.3E-69	1.7E-68	0.26	yes
K01838	beta-phosphoglucomutase [EC:5.4.2.6]	0.21	8.4E-69	2.2E-68	0.21	
K01342	subtilisin [EC:3.4.21.62]	0.21	9.6E-69	2.6E-68	0.28	yes
K01512	acylphosphatase [EC:3.6.1.7]	0.21	9.9E-69	2.6E-68	0.22	
K07155	quercetin 2,3-dioxygenase [EC:1.13.11.24]	0.21	1.3E-68	3.5E-68	0.28	yes
K08744	cardiolipin synthase (CMP-forming) [EC:2.7.8.41]	0.21	1.4E-68	3.7E-68	0.21	
K01892	histidyl-tRNA synthetase [EC:6.1.1.21]	0.21	1.4E-68	3.7E-68	0.26	
K05882	aryl-alcohol dehydrogenase (NADP+) [EC:1.1.1.91]	0.21	1.6E-68	4.2E-68	0.26	
K01624	fructose-bisphosphate aldolase, class II [EC:4.1.2.13]	0.21	1.9E-68	5.0E-68	0.20	
K01210	glucan 1,3-beta-glucosidase [EC:3.2.1.58]	0.21	2.1E-68	5.6E-68	0.20	
K03431	phosphoglucosamine mutase [EC:5.4.2.10]	0.21	2.4E-68	6.3E-68	0.21	
K11529	glycerate 2-kinase [EC:2.7.1.165]	0.21	3.0E-68	7.9E-68	0.28	
K14520	4-hydroxyacetophenone monooxygenase [EC:1.14.13.84]	0.21	3.4E-68	9.0E-68	0.28	yes
K02760	cellobiose PTS system EIIB component [EC:2.7.1.196 2.7.1.205]	0.21	6.8E-68	1.8E-67	0.21	
K01952	phosphoribosylformylglycinamidine synthase [EC:6.3.5.3]	0.21	8.4E-68	2.2E-67	0.27	
K05343	maltose alpha-D-glucosyltransferase / alpha-amylase [EC:5.4.99.16 3.2.1.1]	0.21	9.1E-68	2.4E-67	0.21	yes
K02492	glutamyl-tRNA reductase [EC:1.2.1.70]	0.20	1.1E-67	2.9E-67	0.21	
K00162	pyruvate dehydrogenase E1 component beta subunit [EC:1.2.4.1]	0.20	1.6E-67	4.2E-67	0.21	
K01215	glucan 1,6-alpha-glucosidase [EC:3.2.1.70]	0.20	1.8E-67	4.7E-67	0.22	
K07650	two-component system, OmpR family, sensor histidine kinase CssS [EC:2.7.13.3]	0.20	2.7E-67	7.1E-67	0.22	
K02301	NA	0.20	3.2E-67	8.4E-67	0.24	
K01419	ATP-dependent HslUV protease, peptidase subunit HslV [EC:3.4.25.2]	0.20	4.9E-67	1.3E-66	0.23	
K01839	phosphopentomutase [EC:5.4.2.7]	0.20	5.1E-67	1.3E-66	0.21	
K00336	NADH-quinone oxidoreductase subunit G [EC:7.1.1.2]	0.20	5.3E-67	1.4E-66	0.22	
K10804	acyl-CoA thioesterase I [EC:3.1.2.- 3.1.2.2 3.1.1.2 3.1.1.5]	0.20	6.0E-67	1.6E-66	0.24	
K00344	NADPH:quinone reductase [EC:1.6.5.5]	0.20	6.1E-67	1.6E-66	0.24	
K01644	citrate lyase subunit beta / citryl-CoA lyase [EC:4.1.3.34]	0.20	6.4E-67	1.7E-66	0.23	
K01621	xylulose-5-phosphate/fructose-6-phosphate phosphoketolase [EC:4.1.2.9 4.1.2.22]	0.20	6.4E-67	1.7E-66	0.20	
K01736	chorismate synthase [EC:4.2.3.5]	0.20	8.0E-67	2.1E-66	0.26	
K01876	aspartyl-tRNA synthetase [EC:6.1.1.12]	0.20	1.1E-66	2.9E-66	0.25	
K06045	squalene-hopene/tetraprenyl-beta-curcumene cyclase [EC:5.4.99.17 4.2.1.129]	0.20	1.2E-66	3.1E-66	0.22	
K12252	arginine:pyruvate transaminase [EC:2.6.1.84]	0.20	1.4E-66	3.6E-66	0.28	
K01193	beta-fructofuranosidase [EC:3.2.1.26]	0.20	2.9E-66	7.5E-66	0.20	
K00858	NAD+ kinase [EC:2.7.1.23]	0.20	4.0E-66	1.0E-65	0.20	
K05919	superoxide reductase [EC:1.15.1.2]	0.20	4.5E-66	1.2E-65	0.22	
K06173	tRNA pseudouridine38-40 synthase [EC:5.4.99.12]	0.20	4.8E-66	1.2E-65	0.20	
K02470	DNA gyrase subunit B [EC:5.6.2.2]	0.20	5.1E-66	1.3E-65	0.26	
K01779	aspartate racemase [EC:5.1.1.13]	0.20	6.9E-66	1.8E-65	0.20	
K01881	prolyl-tRNA synthetase [EC:6.1.1.15]	0.20	7.6E-66	2.0E-65	0.25	
K14067	malate-CoA ligase subunit beta [EC:6.2.1.9]	0.20	7.8E-66	2.0E-65	0.27	
K01451	hippurate hydrolase [EC:3.5.1.32]	0.20	1.0E-65	2.6E-65	0.22	

Predictor	Description	Pearson's r	P	FDR-adjusted P	Spearman's rho	Associated with fractures (P<0.05)
K13677	1,2-diacylglycerol-3-alpha-glucose alpha-1,2-glucosyltransferase [EC:2.4.1.208]	0.20	1.0E-65	2.6E-65	0.22	
K06606	2-keto-myo-inositol isomerase [EC:5.3.99.11]	0.20	1.3E-65	3.3E-65	0.25	
K04109	4-hydroxybenzoyl-CoA reductase subunit beta [EC:1.1.7.1]	0.20	1.5E-65	3.8E-65	0.25	yes
K04107	4-hydroxybenzoyl-CoA reductase subunit gamma [EC:1.1.7.1]	0.20	1.5E-65	3.8E-65	0.25	yes
K13659	2-beta-glucuronyltransferase [EC:2.4.1.264]	0.20	1.5E-65	3.8E-65	0.28	
K01889	phenylalanyl-tRNA synthetase alpha chain [EC:6.1.1.20]	0.20	1.5E-65	3.8E-65	0.26	
K07540	benzylsuccinate synthase [EC:4.1.99.11]	0.20	1.7E-65	4.4E-65	0.28	yes
K00161	pyruvate dehydrogenase E1 component alpha subunit [EC:1.2.4.1]	0.20	1.8E-65	4.6E-65	0.21	
K00801	farnesyl-diphosphate farnesyltransferase [EC:2.5.1.21]	0.20	1.9E-65	4.9E-65	0.21	
K01227	mannosyl-glycoprotein endo-beta-N-acetylglucosaminidase [EC:3.2.1.96]	0.20	2.0E-65	5.1E-65	0.23	
K01137	N-acetylglucosamine-6-sulfatase [EC:3.1.6.14]	0.20	2.0E-65	5.1E-65	0.22	
K00695	sucrose synthase [EC:2.4.1.13]	0.20	2.4E-65	6.1E-65	0.27	
K03785	3-dehydroquinate dehydratase I [EC:4.2.1.10]	0.20	3.8E-65	9.7E-65	0.20	
K06027	vesicle-fusing ATPase [EC:3.6.4.6]	0.20	4.1E-65	1.0E-64	0.27	
K07559	putative RNA 2'-phosphotransferase [EC:2.7.1.-]	0.20	4.1E-65	1.0E-64	0.22	
K02045	sulfate/thiosulfate transport system ATP-binding protein [EC:7.3.2.3]	0.20	4.5E-65	1.1E-64	0.23	
K00823	4-aminobutyrate aminotransferase [EC:2.6.1.19]	0.20	5.9E-65	1.5E-64	0.23	
K11177	xanthine dehydrogenase YagR molybdenum-binding subunit [EC:1.17.1.4]	0.20	6.7E-65	1.7E-64	0.24	
K00007	D-arabinitol 4-dehydrogenase [EC:1.1.1.11]	0.20	6.8E-65	1.7E-64	0.27	
K12984	(heptosyl)LPS beta-1,4-glucosyltransferase [EC:2.4.1.-]	0.20	8.0E-65	2.0E-64	0.24	
K06223	DNA adenine methylase [EC:2.1.1.72]	0.20	8.7E-65	2.2E-64	0.22	
K13678	1,2-diacylglycerol-3-alpha-glucose alpha-1,2-galactosyltransferase [EC:2.4.1.-]	0.20	8.9E-65	2.2E-64	0.23	
K00887	undecaprenol kinase [EC:2.7.1.66]	0.20	9.2E-65	2.3E-64	0.21	
K01452	chitin deacetylase [EC:3.5.1.41]	0.20	1.1E-64	2.8E-64	0.23	
K11178	xanthine dehydrogenase YagS FAD-binding subunit [EC:1.17.1.4]	0.20	1.3E-64	3.3E-64	0.24	
K11263	acetyl-CoA/propionyl-CoA carboxylase, biotin carboxylase, biotin carboxyl carrier protein [EC:6.4.1.2 6.4.1.3 6.3.4.14]	0.20	1.4E-64	3.5E-64	0.20	
K01223	6-phospho-beta-glucosidase [EC:3.2.1.86]	0.20	1.4E-64	3.5E-64	0.21	
K00005	glycerol dehydrogenase [EC:1.1.1.6]	0.20	1.7E-64	4.3E-64	0.22	
K02500	imidazole glycerol-phosphate synthase subunit HisF [EC:4.3.2.10]	0.20	1.9E-64	4.8E-64	0.25	
K16147	starch synthase (maltosyl-transferring) [EC:2.4.99.16]	0.20	2.4E-64	6.0E-64	0.20	
K11358	aspartate aminotransferase [EC:2.6.1.1]	0.20	2.5E-64	6.3E-64	0.20	
K14627	dehydratase [EC:4.2.1.-]	0.20	2.8E-64	7.0E-64	0.27	
K14633	ketoreductase RED2 [EC:1.1.1.-]	0.20	3.2E-64	8.0E-64	0.26	
K08282	non-specific serine/threonine protein kinase [EC:2.7.11.1]	0.20	3.9E-64	9.8E-64	0.21	
K13603	3-vinyl bacteriochlorophyllide hydratase [EC:4.2.1.169]	0.20	4.0E-64	1.0E-63	0.27	yes
K08659	dipeptidase [EC:3.4.-.-]	0.20	4.2E-64	1.0E-63	0.20	
K00852	ribokinase [EC:2.7.1.15]	0.20	5.3E-64	1.3E-63	0.20	
K01939	adenylosuccinate synthase [EC:6.3.4.4]	0.20	7.6E-64	1.9E-63	0.25	
K00926	carbamate kinase [EC:2.7.2.2]	0.20	8.9E-64	2.2E-63	0.23	
K05351	D-xylulose reductase [EC:1.1.1.9]	0.20	9.3E-64	2.3E-63	0.20	
K00846	ketohexokinase [EC:2.7.1.3]	0.20	1.2E-63	3.0E-63	0.27	
K13787	geranylgeranyl diphosphate synthase, type I [EC:2.5.1.1 2.5.1.10 2.5.1.29]	0.20	1.2E-63	3.0E-63	0.20	

Predictor	Description	Pearson's r	P	FDR-adjusted P	Spearman's rho	Associated with fractures (P<0.05)
K00105	alpha-glycerophosphate oxidase [EC:1.1.3.21]	0.20	1.7E-63	4.2E-63	0.22	
K00549	5-methyltetrahydropteroyltriglutamate--homocysteine methyltransferase [EC:2.1.1.14]	0.20	1.8E-63	4.5E-63	0.19	
K02618	oxepin-CoA hydrolase / 3-oxo-5,6-dehydrosuberyl-CoA semialdehyde dehydrogenase [EC:3.3.2.12 1.2.1.91]	0.20	1.9E-63	4.7E-63	0.24	
K01095	phosphatidylglycerophosphatase A [EC:3.1.3.27]	0.20	2.3E-63	5.7E-63	0.24	
K01697	cystathionine beta-synthase [EC:4.2.1.22]	0.20	2.9E-63	7.2E-63	0.20	
K13571	proteasome accessory factor A [EC:6.3.1.19]	0.20	3.0E-63	7.4E-63	0.19	
K00133	aspartate-semialdehyde dehydrogenase [EC:1.2.1.11]	0.20	3.2E-63	7.9E-63	0.26	
K15533	1,3-beta-galactosyl-N-acetylhexosamine phosphorylase [EC:2.4.1.211]	0.20	3.6E-63	8.9E-63	0.21	
K03574	8-oxo-dGTP diphosphatase [EC:3.6.1.55]	0.20	3.7E-63	9.1E-63	0.20	
K01887	arginyl-tRNA synthetase [EC:6.1.1.19]	0.20	4.0E-63	9.9E-63	0.25	
K01875	seryl-tRNA synthetase [EC:6.1.1.11]	0.20	4.1E-63	1.0E-62	0.25	
K02794	mannose PTS system EIIAB component [EC:2.7.1.191]	0.20	4.8E-63	1.2E-62	0.21	
K02446	fructose-1,6-bisphosphatase II [EC:3.1.3.11]	0.20	6.7E-63	1.6E-62	0.22	
K01626	3-deoxy-7-phosphoheptulonate synthase [EC:2.5.1.54]	0.20	7.4E-63	1.8E-62	0.19	
K01883	cysteinyl-tRNA synthetase [EC:6.1.1.16]	0.20	7.5E-63	1.8E-62	0.26	
K01803	triosephosphate isomerase (TIM) [EC:5.3.1.1]	0.20	8.1E-63	2.0E-62	0.25	
K04712	sphingolipid 4-desaturase/C4-monooxygenase [EC:1.14.19.17 1.14.18.5]	0.20	8.6E-63	2.1E-62	0.27	
K00027	malate dehydrogenase (oxaloacetate-decarboxylating) [EC:1.1.1.38]	0.20	1.1E-62	2.7E-62	0.21	
K05358	quinate dehydrogenase (quinone) [EC:1.1.5.8]	0.20	1.1E-62	2.7E-62	0.28	
K01184	polygalacturonase [EC:3.2.1.15]	0.20	1.3E-62	3.2E-62	0.27	yes
K00886	polyphosphate glucokinase [EC:2.7.1.63]	0.20	1.6E-62	3.9E-62	0.19	
K01303	acylaminoacyl-peptidase [EC:3.4.19.1]	0.20	1.6E-62	3.9E-62	0.22	yes
K01756	adenylosuccinate lyase [EC:4.3.2.2]	0.20	2.3E-62	5.6E-62	0.25	
K07768	two-component system, OmpR family, sensor histidine kinase SenX3 [EC:2.7.13.3]	0.20	4.1E-62	1.0E-61	0.19	
K02501	imidazole glycerol-phosphate synthase subunit HisH [EC:4.3.2.10]	0.20	4.4E-62	1.1E-61	0.25	
K01042	L-seryl-tRNA(Ser) seleniumtransferase [EC:2.9.1.1]	0.20	5.4E-62	1.3E-61	0.22	
K10673	streptomycin 3\-kinase [EC:2.7.1.87]	0.20	8.0E-62	1.9E-61	0.25	
K01866	tyrosyl-tRNA synthetase [EC:6.1.1.1]	0.20	1.3E-61	3.2E-61	0.25	
K00064	D-threo-aldose 1-dehydrogenase [EC:1.1.1.122]	0.20	2.2E-61	5.3E-61	0.23	
K15784	N2-acetyl-L-2,4-diaminobutanoate deacetylase [EC:3.5.1.125]	0.19	3.0E-61	7.3E-61	0.28	
K09903	uridylate kinase [EC:2.7.4.22]	0.19	3.5E-61	8.5E-61	0.24	
K12526	bifunctional diaminopimelate decarboxylase / aspartate kinase [EC:4.1.1.20 2.7.2.4]	0.19	3.5E-61	8.5E-61	0.27	yes
K00121	S-(hydroxymethyl)glutathione dehydrogenase / alcohol dehydrogenase [EC:1.1.1.284 1.1.1.1]	0.19	5.1E-61	1.2E-60	0.23	
K00433	non-heme chloroperoxidase [EC:1.11.1.10]	0.19	5.8E-61	1.4E-60	0.23	yes
K01653	acetolactate synthase I/III small subunit [EC:2.2.1.6]	0.19	7.6E-61	1.8E-60	0.24	
K11912	serine/threonine-protein kinase PpkA [EC:2.7.11.1]	0.19	8.7E-61	2.1E-60	0.26	
K00939	adenylate kinase [EC:2.7.4.3]	0.19	9.0E-61	2.2E-60	0.24	
K00997	holo-[acyl-carrier protein] synthase [EC:2.7.8.7]	0.19	1.2E-60	2.9E-60	0.19	
K01940	argininosuccinate synthase [EC:6.3.4.5]	0.19	1.2E-60	2.9E-60	0.24	
K02236	leader peptidase (prepilin peptidase) / N-methyltransferase [EC:3.4.23.43 2.1.1.-]	0.19	1.4E-60	3.4E-60	0.21	
K00324	H+-translocating NAD(P) transhydrogenase subunit alpha [EC:1.6.1.2 7.1.1.1]	0.19	1.4E-60	3.4E-60	0.19	
K11533	fatty acid synthase, bacteria type [EC:2.3.1.-]	0.19	1.4E-60	3.4E-60	0.19	

Predictor	Description	Pearson's r	P	FDR-adjusted P	Spearman's rho	Associated with fractures (P<0.05)
K15524	mannosylglycerate hydrolase [EC:3.2.1.170]	0.19	1.7E-60	4.1E-60	0.21	
K03658	DNA helicase IV [EC:3.6.4.12]	0.19	1.9E-60	4.6E-60	0.20	
K01937	CTP synthase [EC:6.3.4.2]	0.19	2.4E-60	5.8E-60	0.24	
K02851	UDP-GlcNAc:undecaprenyl-phosphate/decaprenyl-phosphate GlcNAc-1-phosphate transferase [EC:2.7.8.33 2.7.8.35]	0.19	2.7E-60	6.5E-60	0.19	
K04092	chorismate mutase [EC:5.4.99.5]	0.19	2.8E-60	6.7E-60	0.19	
K01635	tagatose 1,6-diphosphate aldolase [EC:4.1.2.40]	0.19	3.1E-60	7.4E-60	0.21	
K01478	arginine deiminase [EC:3.5.3.6]	0.19	3.1E-60	7.4E-60	0.21	
K01956	carbamoyl-phosphate synthase small subunit [EC:6.3.5.5]	0.19	3.2E-60	7.6E-60	0.24	
K04087	modulator of FtsH protease HflC	0.19	4.0E-60	9.5E-60	0.23	
K04102	4,5-dihydroxyphthalate decarboxylase [EC:4.1.1.55]	0.19	4.1E-60	9.8E-60	0.26	
K09516	all-trans-retinol 13,14-reductase [EC:1.3.99.23]	0.19	5.0E-60	1.2E-59	0.26	
K13605	bacteriochlorophyll c synthase [EC:2.5.1.-]	0.19	6.9E-60	1.6E-59	0.27	yes
K01760	cysteine-S-conjugate beta-lyase [EC:4.4.1.13]	0.19	7.9E-60	1.9E-59	0.19	
K05913	2,4'-dihydroxyacetophenone dioxygenase [EC:1.13.11.41]	0.19	8.2E-60	1.9E-59	0.26	
K02806	nitrogen PTS system EIIA component [EC:2.7.1.-]	0.19	1.1E-59	2.6E-59	0.20	
K00873	pyruvate kinase [EC:2.7.1.40]	0.19	1.1E-59	2.6E-59	0.24	
K13875	L-arabonate dehydrase [EC:4.2.1.25]	0.19	1.2E-59	2.8E-59	0.26	
K01219	beta-agarase [EC:3.2.1.81]	0.19	1.8E-59	4.3E-59	0.23	yes
K15986	manganese-dependent inorganic pyrophosphatase [EC:3.6.1.1]	0.19	2.0E-59	4.7E-59	0.20	
K03578	ATP-dependent helicase HrpA [EC:3.6.4.13]	0.19	2.0E-59	4.7E-59	0.19	
K00851	gluconokinase [EC:2.7.1.12]	0.19	2.3E-59	5.4E-59	0.19	
K00128	aldehyde dehydrogenase (NAD+) [EC:1.2.1.3]	0.19	2.3E-59	5.4E-59	0.19	
K00192	anaerobic carbon-monoxide dehydrogenase, CODH/ACS complex subunit alpha [EC:1.2.7.4]	0.19	2.4E-59	5.7E-59	0.28	
K00638	chloramphenicol O-acetyltransferase type B [EC:2.3.1.28]	0.19	2.7E-59	6.4E-59	0.24	
K01195	beta-glucuronidase [EC:3.2.1.31]	0.19	2.7E-59	6.4E-59	0.20	
K00483	4-hydroxyphenylacetate 3-monooxygenase [EC:1.14.14.9]	0.19	3.9E-59	9.2E-59	0.23	
K01945	phosphoribosylamine---glycine ligase [EC:6.3.4.13]	0.19	4.6E-59	1.1E-58	0.24	
K14165	atypical dual specificity phosphatase [EC:3.1.3.16 3.1.3.48]	0.19	5.0E-59	1.2E-58	0.27	
K06726	D-ribose pyranase [EC:5.4.99.62]	0.19	1.2E-58	2.8E-58	0.21	
K02825	pyrimidine operon attenuation protein / uracil phosphoribosyltransferase [EC:2.4.2.9]	0.19	1.8E-58	4.2E-58	0.20	
K01865	(hydroxyamino)benzene mutase [EC:5.4.4.1]	0.19	2.0E-58	4.7E-58	0.27	yes
K00325	H+-translocating NAD(P) transhydrogenase subunit beta [EC:1.6.1.2 7.1.1.1]	0.19	2.0E-58	4.7E-58	0.19	
K01933	phosphoribosylformylglycinamidine cyclo-ligase [EC:6.3.3.1]	0.19	2.4E-58	5.6E-58	0.25	
K10944	methane/ammonia monooxygenase subunit A [EC:1.14.18.3 1.14.99.39]	0.19	3.3E-58	7.7E-58	0.25	
K13542	uroporphyrinogen III methyltransferase / synthase [EC:2.1.1.107 4.2.1.75]	0.19	4.2E-58	9.8E-58	0.20	
K00134	glyceraldehyde 3-phosphate dehydrogenase (phosphorylating) [EC:1.2.1.12]	0.19	4.5E-58	1.1E-57	0.24	
K13601	bacteriochlorophyllide d C-8(2)-methyltransferase [EC:2.1.1.332]	0.19	4.7E-58	1.1E-57	0.26	yes
K00299	FMN reductase [EC:1.5.1.38]	0.19	5.1E-58	1.2E-57	0.22	
K00104	glycolate oxidase [EC:1.1.3.15]	0.19	6.8E-58	1.6E-57	0.19	
K13669	alpha-1,2-mannosyltransferase [EC:2.4.1.-]	0.19	7.2E-58	1.7E-57	0.26	
K13060	acyl homoserine lactone synthase [EC:2.3.1.184]	0.19	7.6E-58	1.8E-57	0.25	
K00690	sucrose phosphorylase [EC:2.4.1.7]	0.19	7.8E-58	1.8E-57	0.19	

Predictor	Description	Pearson's r	P	FDR-adjusted P	Spearman's rho	Associated with fractures (P<0.05)
K01922	phosphopantothenate---cysteine ligase (ATP) [EC:6.3.2.51]	0.19	8.2E-58	1.9E-57	0.19	
K03216	tRNA (cytidine/uridine-2'-O-)-methyltransferase [EC:2.1.1.207]	0.19	8.6E-58	2.0E-57	0.19	
K00217	maleylacetate reductase [EC:1.3.1.32]	0.19	9.1E-58	2.1E-57	0.25	
K01027	3-oxoacid CoA-transferase [EC:2.8.3.5]	0.19	1.0E-57	2.3E-57	0.26	yes
K03366	meso-butanediol dehydrogenase / (S,S)-butanediol dehydrogenase / diacetyl reductase [EC:1.1.1.- 1.1.1.76 1.1.1.304]	0.19	1.3E-57	3.0E-57	0.21	
K00032	phosphogluconate 2-dehydrogenase [EC:1.1.1.43]	0.19	1.7E-57	3.9E-57	0.26	
K02041	phosphonate transport system ATP-binding protein [EC:7.3.2.2]	0.19	2.6E-57	6.0E-57	0.21	
K02334	DNA polymerase bacteriophage-type [EC:2.7.7.7]	0.19	3.3E-57	7.6E-57	0.20	
K13543	uroporphyrinogen III methyltransferase / synthase [EC:2.1.1.107 4.2.1.75]	0.19	3.6E-57	8.3E-57	0.24	
K00164	2-oxoglutarate dehydrogenase E1 component [EC:1.2.4.2]	0.19	3.7E-57	8.5E-57	0.20	
K05999	xanthomonalisin [EC:3.4.21.101]	0.19	3.9E-57	9.0E-57	0.25	
K12987	alpha-1,6-rhamnosyltransferase [EC:2.4.1.-]	0.19	6.2E-57	1.4E-56	0.26	
K13788	phosphate acetyltransferase [EC:2.3.1.8]	0.19	7.4E-57	1.7E-56	0.18	
K00014	shikimate dehydrogenase [EC:1.1.1.25]	0.19	7.4E-57	1.7E-56	0.24	
K00990	[protein-PII] uridylyltransferase [EC:2.7.7.59]	0.19	7.9E-57	1.8E-56	0.18	
K02563	UDP-N-acetylglucosamine--N-acetylmuramyl-(pentapeptide) pyrophosphoryl-undecaprenol N-acetylglucosamine transferase [EC:2.4.1.227]	0.19	9.0E-57	2.1E-56	0.24	
K04075	tRNA(Ile)-lysidine synthase [EC:6.3.4.19]	0.19	9.9E-57	2.3E-56	0.19	
K01896	medium-chain acyl-CoA synthetase [EC:6.2.1.2]	0.19	1.0E-56	2.3E-56	0.25	yes
K01909	long-chain-fatty-acid--[acyl-carrier-protein] ligase [EC:6.2.1.20]	0.19	1.7E-56	3.9E-56	0.25	
K08483	phosphoenolpyruvate-protein phosphotransferase (PTS system enzyme I) [EC:2.7.3.9]	0.19	2.3E-56	5.3E-56	0.19	
K03767	peptidyl-prolyl cis-trans isomerase A (cyclophilin A) [EC:5.2.1.8]	0.19	2.3E-56	5.3E-56	0.18	
K07272	rhamnosyltransferase [EC:2.4.1.-]	0.19	3.3E-56	7.5E-56	0.18	
K00315	dimethylglycine dehydrogenase [EC:1.5.8.4]	0.19	4.8E-56	1.1E-55	0.23	
K15786	aspartate-semialdehyde dehydrogenase [EC:1.2.1.-]	0.19	4.8E-56	1.1E-55	0.27	
K00988	sulfate adenylyltransferase (ADP) / ATP adenylyltransferase [EC:2.7.7.5 2.7.7.53]	0.19	5.0E-56	1.1E-55	0.26	
K00476	aspartate beta-hydroxylase [EC:1.14.11.16]	0.19	6.0E-56	1.4E-55	0.20	
K05998	pseudomonalisin [EC:3.4.21.100]	0.19	6.0E-56	1.4E-55	0.20	
K02777	sugar PTS system EIIA component [EC:2.7.1.-]	0.19	6.6E-56	1.5E-55	0.21	
K15913	UDP-N-acetylbacillosamine N-acetyltransferase [EC:2.3.1.203]	0.19	7.2E-56	1.6E-55	0.20	
K00260	glutamate dehydrogenase [EC:1.4.1.2]	0.19	1.2E-55	2.7E-55	0.27	
K00363	nitrite reductase (NADH) small subunit [EC:1.7.1.15]	0.19	1.2E-55	2.7E-55	0.22	
K10805	acyl-CoA thioesterase II [EC:3.1.2.-]	0.19	1.6E-55	3.6E-55	0.18	
K12527	putative selenate reductase [EC:1.97.1.9]	0.19	2.1E-55	4.8E-55	0.21	
K00054	hydroxymethylglutaryl-CoA reductase [EC:1.1.1.88]	0.19	2.1E-55	4.8E-55	0.20	
K01869	leucyl-tRNA synthetase [EC:6.1.1.4]	0.19	2.3E-55	5.2E-55	0.25	
K11395	2-dehydro-3-deoxy-phosphogluconate/2-dehydro-3-deoxy-6-phosphogalactonate aldolase [EC:4.1.2.55]	0.19	2.3E-55	5.2E-55	0.19	
K09001	anhydro-N-acetylmuramic acid kinase [EC:2.7.1.170]	0.18	3.3E-55	7.5E-55	0.22	
K07778	two-component system, NarL family, sensor histidine kinase DesK [EC:2.7.13.3]	0.18	3.4E-55	7.7E-55	0.20	
K04088	modulator of FtsH protease HflK	0.18	3.7E-55	8.3E-55	0.22	
K00390	phosphoadenosine phosphosulfate reductase [EC:1.8.4.8 1.8.4.10]	0.18	3.8E-55	8.6E-55	0.20	
K03579	ATP-dependent helicase HrpB [EC:3.6.4.13]	0.18	5.1E-55	1.1E-54	0.19	
K01598	phosphopantothenoylcysteine decarboxylase [EC:4.1.1.36]	0.18	8.6E-55	1.9E-54	0.19	

Predictor	Description	Pearson's r	P	FDR-adjusted P	Spearman's rho	Associated with fractures (P<0.05)
K12506	2-C-methyl-D-erythritol 4-phosphate cytidylyltransferase / 2-C-methyl-D-erythritol 2,4-cyclodiphosphate synthase [EC:2.7.7.60 4.6.1.12]	0.18	1.0E-54	2.2E-54	0.23	
K00228	coproporphyrinogen III oxidase [EC:1.3.3.3]	0.18	1.1E-54	2.5E-54	0.22	
K07655	two-component system, OmpR family, sensor histidine kinase PrrB [EC:2.7.13.3]	0.18	1.1E-54	2.5E-54	0.25	
K01826	5-carboxymethyl-2-hydroxymuconate isomerase [EC:5.3.3.10]	0.18	1.5E-54	3.4E-54	0.22	
K12339	S-sulfo-L-cysteine synthase (O-acetyl-L-serine-dependent) [EC:2.5.1.144]	0.18	1.5E-54	3.4E-54	0.19	
K07406	alpha-galactosidase [EC:3.2.1.22]	0.18	1.6E-54	3.6E-54	0.22	
K01934	5-formyltetrahydrofolate cyclo-ligase [EC:6.3.3.2]	0.18	1.6E-54	3.6E-54	0.19	
K13657	alpha-1,3-mannosyltransferase [EC:2.4.1.252]	0.18	1.7E-54	3.8E-54	0.26	
K00117	quinoprotein glucose dehydrogenase [EC:1.1.5.2]	0.18	2.0E-54	4.5E-54	0.21	
K07547	2-[hydroxy(phenyl)methyl]-succinyl-CoA dehydrogenase BbsC subunit [EC:1.1.1.35]	0.18	2.3E-54	5.1E-54	0.23	
K08722	5'-deoxynucleotidase [EC:3.1.3.89]	0.18	3.4E-54	7.6E-54	0.22	
K00130	betaine-aldehyde dehydrogenase [EC:1.2.1.8]	0.18	4.2E-54	9.4E-54	0.22	
K01061	carboxymethylenebutenolidase [EC:3.1.1.45]	0.18	4.4E-54	9.8E-54	0.22	
K04711	dihydroceramidase [EC:3.5.1.-]	0.18	7.8E-54	1.7E-53	0.25	yes
K02496	uroporphyrin-III C-methyltransferase [EC:2.1.1.107]	0.18	9.1E-54	2.0E-53	0.22	
K03272	D-beta-D-heptose 7-phosphate kinase / D-beta-D-heptose 1-phosphate adenosyltransferase [EC:2.7.1.167 2.7.7.70]	0.18	1.1E-53	2.4E-53	0.18	
K00163	pyruvate dehydrogenase E1 component [EC:1.2.4.1]	0.18	1.3E-53	2.9E-53	0.22	
K07518	hydroxybutyrate-dimer hydrolase [EC:3.1.1.22]	0.18	1.7E-53	3.8E-53	0.26	yes
K00984	streptomycin 3\-adenylyltransferase [EC:2.7.7.47]	0.18	1.7E-53	3.8E-53	0.21	
K08309	soluble lytic murein transglycosylase [EC:4.2.2.-]	0.18	2.6E-53	5.8E-53	0.21	
K01906	6-carboxyhexanoate--CoA ligase [EC:6.2.1.14]	0.18	3.0E-53	6.7E-53	0.20	
K05396	D-cysteine desulfhydrase [EC:4.4.1.15]	0.18	4.1E-53	9.1E-53	0.20	
K02744	N-acetylgalactosamine PTS system EIIA component [EC:2.7.1.-]	0.18	4.6E-53	1.0E-52	0.19	
K08690	cis-2,3-dihydrobiphenyl-2,3-diol dehydrogenase [EC:1.3.1.56]	0.18	5.4E-53	1.2E-52	0.24	
K00381	sulfite reductase (NADPH) hemoprotein beta-component [EC:1.8.1.2]	0.18	5.8E-53	1.3E-52	0.19	
K12553	penicillin-binding protein 3 [EC:3.4.-.-]	0.18	6.8E-53	1.5E-52	0.19	
K00799	glutathione S-transferase [EC:2.5.1.18]	0.18	7.9E-53	1.7E-52	0.22	
K00088	IMP dehydrogenase [EC:1.1.1.205]	0.18	8.5E-53	1.9E-52	0.18	
K06181	23S rRNA pseudouridine2457 synthase [EC:5.4.99.20]	0.18	8.9E-53	2.0E-52	0.19	
K01495	GTP cyclohydrolase IA [EC:3.5.4.16]	0.18	9.0E-53	2.0E-52	0.18	
K02340	DNA polymerase III subunit delta [EC:2.7.7.7]	0.18	1.2E-52	2.6E-52	0.24	yes
K01462	peptide deformylase [EC:3.5.1.88]	0.18	1.3E-52	2.9E-52	0.22	
K00963	UTP--glucose-1-phosphate uridylyltransferase [EC:2.7.7.9]	0.18	1.4E-52	3.1E-52	0.18	
K00783	23S rRNA (pseudouridine1915-N3)-methyltransferase [EC:2.1.1.177]	0.18	1.6E-52	3.5E-52	0.24	yes
K03684	ribonuclease D [EC:3.1.13.5]	0.18	1.6E-52	3.5E-52	0.18	
K00239	succinate dehydrogenase / fumarate reductase, flavoprotein subunit [EC:1.3.5.1 1.3.5.4]	0.18	1.8E-52	3.9E-52	0.18	
K06193	protein PhnA	0.18	2.5E-52	5.5E-52	0.19	
K00380	sulfite reductase (NADPH) flavoprotein alpha-component [EC:1.8.1.2]	0.18	2.5E-52	5.5E-52	0.19	
K00737	beta-1,4-mannosyl-glycoprotein beta-1,4-N-acetylglucosaminyltransferase [EC:2.4.1.144]	0.18	2.7E-52	5.9E-52	0.24	
K05580	NAD(P)H-quinone oxidoreductase subunit I [EC:7.1.1.2]	0.18	2.8E-52	6.1E-52	0.22	yes
K01459	N-carbamoyl-D-amino-acid hydrolase [EC:3.5.1.77]	0.18	3.5E-52	7.6E-52	0.22	
K00697	trehalose 6-phosphate synthase [EC:2.4.1.15 2.4.1.347]	0.18	3.9E-52	8.5E-52	0.21	

Predictor	Description	Pearson's r	P	FDR-adjusted P	Spearman's rho	Associated with fractures (P<0.05)
K01414	oligopeptidase A [EC:3.4.24.70]	0.18	4.3E-52	9.4E-52	0.19	
K05982	deoxyribonuclease V [EC:3.1.21.7]	0.18	4.5E-52	9.8E-52	0.21	
K01597	diphosphomevalonate decarboxylase [EC:4.1.1.33]	0.18	4.7E-52	1.0E-51	0.19	
K01258	tripeptide aminopeptidase [EC:3.4.11.4]	0.18	5.2E-52	1.1E-51	0.17	
K02768	fructose PTS system EIIA component [EC:2.7.1.202]	0.18	7.7E-52	1.7E-51	0.19	
K01312	trypsin [EC:3.4.21.4]	0.18	8.8E-52	1.9E-51	0.17	
K08680	2-succinyl-6-hydroxy-2,4-cyclohexadiene-1-carboxylate synthase [EC:4.2.99.20]	0.18	9.2E-52	2.0E-51	0.18	
K10708	fructoselysine 6-phosphate deglycase [EC:3.5.-.-]	0.18	1.1E-51	2.4E-51	0.19	
K06859	glucose-6-phosphate isomerase, archaeal [EC:5.3.1.9]	0.18	1.4E-51	3.0E-51	0.19	
K13874	L-arabinonolactonase [EC:3.1.1.15]	0.18	1.7E-51	3.7E-51	0.21	
K01011	thiosulfate/3-mercaptopyruvate sulfurtransferase [EC:2.8.1.1 2.8.1.2]	0.18	2.0E-51	4.3E-51	0.20	
K00693	glycogen synthase [EC:2.4.1.11]	0.18	3.2E-51	6.9E-51	0.24	
K10012	undecaprenyl-phosphate 4-deoxy-4-formamido-L-arabinose transferase [EC:2.4.2.53]	0.18	5.5E-51	1.2E-50	0.21	
K01066	acetyl esterase [EC:3.1.1.-]	0.18	5.9E-51	1.3E-50	0.19	
K00285	D-amino-acid dehydrogenase [EC:1.4.5.1]	0.18	6.2E-51	1.3E-50	0.21	
K05554	aromatase [EC:4.2.1.-]	0.18	7.8E-51	1.7E-50	0.24	
K01069	hydroxyacylglutathione hydrolase [EC:3.1.2.6]	0.18	8.4E-51	1.8E-50	0.21	
K00158	pyruvate oxidase [EC:1.2.3.3]	0.18	8.8E-51	1.9E-50	0.19	
K01213	galacturan 1,4-alpha-galacturonidase [EC:3.2.1.67]	0.18	1.1E-50	2.4E-50	0.17	yes
K01667	tryptophanase [EC:4.1.99.1]	0.18	1.2E-50	2.6E-50	0.19	
K01611	S-adenosylmethionine decarboxylase [EC:4.1.1.50]	0.18	1.3E-50	2.8E-50	0.19	
K05947	mannosyl-3-phosphoglycerate synthase [EC:2.4.1.217]	0.18	1.3E-50	2.8E-50	0.23	
K01637	isocitrate lyase [EC:4.1.3.1]	0.18	1.4E-50	3.0E-50	0.21	
K02335	DNA polymerase I [EC:2.7.7.7]	0.18	1.5E-50	3.2E-50	0.23	
K05364	penicillin-binding protein A	0.18	1.8E-50	3.9E-50	0.18	
K07545	(R)-benzylsuccinyl-CoA dehydrogenase [EC:1.3.8.3]	0.18	1.9E-50	4.1E-50	0.25	
K03524	BirA family transcriptional regulator, biotin operon repressor / biotin---[acetyl-CoA-carboxylase] ligase [EC:6.3.4.15]	0.18	2.2E-50	4.7E-50	0.23	
K02431	L-fucose mutarotase [EC:5.1.3.29]	0.18	2.4E-50	5.1E-50	0.18	
K10773	endonuclease III [EC:4.2.99.18]	0.18	2.5E-50	5.3E-50	0.23	
K00548	5-methyltetrahydrofolate--homocysteine methyltransferase [EC:2.1.1.13]	0.18	2.6E-50	5.6E-50	0.18	
K00927	phosphoglycerate kinase [EC:2.7.2.3]	0.18	4.5E-50	9.6E-50	0.24	
K07250	4-aminobutyrate aminotransferase / (S)-3-amino-2-methylpropionate transaminase / 5-aminovalerate transaminase [EC:2.6.1.19 2.6.1.22 2.6.1.48]	0.18	4.9E-50	1.0E-49	0.20	
K01000	phospho-N-acetylmuramoyl-pentapeptide-transferase [EC:2.7.8.13]	0.18	4.9E-50	1.0E-49	0.23	
K00558	DNA (cytosine-5)-methyltransferase 1 [EC:2.1.1.37]	0.18	5.4E-50	1.2E-49	0.18	
K16329	pseudouridylate synthase [EC:4.2.1.70]	0.18	5.7E-50	1.2E-49	0.21	
K07549	benzoylsuccinyl-CoA thiolase BbsA subunit [EC:2.3.1.-]	0.18	5.9E-50	1.3E-49	0.24	
K07550	benzoylsuccinyl-CoA thiolase BbsB subunit [EC:2.3.1.-]	0.18	5.9E-50	1.3E-49	0.24	
K05921	5-oxopent-3-ene-1,2,5-tricarboxylate decarboxylase / 2-hydroxyhepta-2,4-diene-1,7-dioate isomerase [EC:4.1.1.68 5.3.3.-]	0.18	6.5E-50	1.4E-49	0.21	
K00138	aldehyde dehydrogenase [EC:1.2.1.-]	0.18	7.0E-50	1.5E-49	0.21	
K01752	L-serine dehydratase [EC:4.3.1.17]	0.18	1.3E-49	2.8E-49	0.17	
K03782	catalase-peroxidase [EC:1.11.1.21]	0.18	1.5E-49	3.2E-49	0.21	
K05939	acyl-[acyl-carrier-protein]-phospholipid O-acyltransferase / long-chain-fatty-acid--[acyl-carrier-protein] ligase [EC:2.3.1.40 6.2.1.20]	0.17	1.5E-49	3.2E-49	0.20	

Predictor	Description	Pearson's r	P	FDR-adjusted P	Spearman's rho	Associated with fractures (P<0.05)
K01497	GTP cyclohydrolase II [EC:3.5.4.25]	0.17	1.7E-49	3.6E-49	0.21	
K08297	crotonobetainyl-CoA dehydrogenase [EC:1.3.8.13]	0.17	1.7E-49	3.6E-49	0.19	
K01295	glutamate carboxypeptidase [EC:3.4.17.11]	0.17	2.0E-49	4.2E-49	0.27	
K00010	myo-inositol 2-dehydrogenase / D-chiro-inositol 1-dehydrogenase [EC:1.1.1.18 1.1.1.369]	0.17	2.3E-49	4.9E-49	0.19	
K00563	23S rRNA (guanine745-N1)-methyltransferase [EC:2.1.1.187]	0.17	2.4E-49	5.1E-49	0.19	
K05578	NAD(P)H-quinone oxidoreductase subunit 6 [EC:7.1.1.2]	0.17	2.8E-49	5.9E-49	0.24	
K15234	citryl-CoA lyase [EC:4.1.3.34]	0.17	3.5E-49	7.4E-49	0.18	
K00464	all-trans-8'-apo-beta-carotenal 15,15'-oxygenase [EC:1.13.11.75]	0.17	3.6E-49	7.6E-49	0.24	
K01872	alanyl-tRNA synthetase [EC:6.1.1.7]	0.17	4.5E-49	9.5E-49	0.17	
K07313	serine/threonine protein phosphatase 1 [EC:3.1.3.16]	0.17	4.7E-49	9.9E-49	0.20	
K01222	6-phospho-beta-glucosidase [EC:3.2.1.86]	0.17	6.6E-49	1.4E-48	0.19	
K00528	ferredoxin/flavodoxin---NADP+ reductase [EC:1.18.1.2 1.19.1.1]	0.17	7.6E-49	1.6E-48	0.18	
K01247	DNA-3-methyladenine glycosylase II [EC:3.2.2.21]	0.17	8.6E-49	1.8E-48	0.19	
K00938	phosphomevalonate kinase [EC:2.7.4.2]	0.17	9.3E-49	2.0E-48	0.18	
K01239	purine nucleosidase [EC:3.2.2.1]	0.17	1.5E-48	3.1E-48	0.18	
K02338	DNA polymerase III subunit beta [EC:2.7.7.7]	0.17	1.5E-48	3.1E-48	0.22	
K10621	2,3-dihydroxy-p-cumate/2,3-dihydroxybenzoate 3,4-dioxygenase [EC:1.13.11.- 1.13.11.14]	0.17	1.5E-48	3.1E-48	0.24	
K05540	tRNA-dihydrouridine synthase B [EC:1.-.-.-]	0.17	1.6E-48	3.3E-48	0.21	
K09698	nondiscriminating glutamyl-tRNA synthetase [EC:6.1.1.24]	0.17	2.4E-48	5.0E-48	0.19	
K01487	guanine deaminase [EC:3.5.4.3]	0.17	3.0E-48	6.3E-48	0.19	
K02786	lactose PTS system EIIA component [EC:2.7.1.207]	0.17	3.2E-48	6.7E-48	0.18	
K04091	alkanesulfonate monooxygenase [EC:1.14.14.5 1.14.14.34]	0.17	3.4E-48	7.1E-48	0.21	
K01588	5-(carboxyamino)imidazole ribonucleotide mutase [EC:5.4.99.18]	0.17	3.9E-48	8.1E-48	0.23	
K15256	tRNA (cmo5U34)-methyltransferase [EC:2.1.1.-]	0.17	4.3E-48	9.0E-48	0.20	
K00151	5-carboxymethyl-2-hydroxymuconic-semialdehyde dehydrogenase [EC:1.2.1.60]	0.17	5.3E-48	1.1E-47	0.21	
K01819	galactose-6-phosphate isomerase [EC:5.3.1.26]	0.17	5.4E-48	1.1E-47	0.18	
K01479	formiminoglutamase [EC:3.5.3.8]	0.17	6.0E-48	1.2E-47	0.19	
K08961	chondroitin-sulfate-ABC endolyase/exolyase [EC:4.2.2.20 4.2.2.21]	0.17	9.5E-48	2.0E-47	0.18	yes
K01232	maltose-6'-phosphate glucosidase [EC:3.2.1.122]	0.17	9.6E-48	2.0E-47	0.19	
K00872	homoserine kinase [EC:2.7.1.39]	0.17	9.9E-48	2.0E-47	0.17	
K01401	aureolysin [EC:3.4.24.29]	0.17	9.9E-48	2.0E-47	0.17	
K04478	monofunctional glycosyltransferase [EC:2.4.1.129]	0.17	9.9E-48	2.0E-47	0.17	
K11695	peptidoglycan pentaglycine glycine transferase (the fourth and fifth glycine) [EC:2.3.2.18]	0.17	9.9E-48	2.0E-47	0.17	
K11694	peptidoglycan pentaglycine glycine transferase (the second and third glycine) [EC:2.3.2.17]	0.17	9.9E-48	2.0E-47	0.17	
K11442	putative uridylyltransferase [EC:2.7.7.-]	0.17	9.9E-48	2.0E-47	0.17	
K07681	two-component system, NarL family, vancomycin resistance sensor histidine kinase VraS [EC:2.7.13.3]	0.17	9.9E-48	2.0E-47	0.17	
K08311	putative (di)nucleoside polyphosphate hydrolase [EC:3.6.1.-]	0.17	1.0E-47	2.1E-47	0.21	
K16215	2-ketoarginine methyltransferase [EC:2.1.1.243]	0.17	1.1E-47	2.3E-47	0.18	
K00116	malate dehydrogenase (quinone) [EC:1.1.5.4]	0.17	1.5E-47	3.1E-47	0.20	
K12552	penicillin-binding protein 1 [EC:3.4.-.-]	0.17	1.6E-47	3.3E-47	0.17	
K03148	sulfur carrier protein ThiS adenylyltransferase [EC:2.7.7.73]	0.17	1.7E-47	3.5E-47	0.17	
K14379	tartrate-resistant acid phosphatase type 5 [EC:3.1.3.2]	0.17	1.8E-47	3.7E-47	0.23	

Predictor	Description	Pearson's r	P	FDR-adjusted P	Spearman's rho	Associated with fractures (P<0.05)
K00060	threonine 3-dehydrogenase [EC:1.1.1.103]	0.17	1.9E-47	3.9E-47	0.18	
K00257	acyl-ACP dehydrogenase [EC:1.3.99.-]	0.17	2.3E-47	4.7E-47	0.24	
K08296	phosphohistidine phosphatase [EC:3.1.3.-]	0.17	2.3E-47	4.7E-47	0.18	
K00666	fatty-acyl-CoA synthase [EC:6.2.1.-]	0.17	2.4E-47	4.9E-47	0.19	
K05556	ketoreductase RED1 [EC:1.1.1.-]	0.17	2.9E-47	5.9E-47	0.22	
K13529	AraC family transcriptional regulator, regulatory protein of adaptative response / DNA-3-methyladenine glycosylase II [EC:3.2.2.21]	0.17	3.2E-47	6.6E-47	0.22	
K00632	acetyl-CoA acyltransferase [EC:2.3.1.16]	0.17	3.8E-47	7.8E-47	0.21	
K00087	xanthine dehydrogenase molybdenum-binding subunit [EC:1.17.1.4]	0.17	4.7E-47	9.6E-47	0.20	
K01595	phosphoenolpyruvate carboxylase [EC:4.1.1.31]	0.17	5.2E-47	1.1E-46	0.17	
K13479	xanthine dehydrogenase FAD-binding subunit [EC:1.17.1.4]	0.17	6.3E-47	1.3E-46	0.20	
K04940	opine dehydrogenase [EC:1.5.1.28]	0.17	6.4E-47	1.3E-46	0.24	
K13990	glutamate formiminotransferase / formiminotetrahydrofolate cyclodeaminase [EC:2.1.2.5 4.3.1.4]	0.17	8.6E-47	1.8E-46	0.19	
K02017	molybdate transport system ATP-binding protein [EC:7.3.2.5]	0.17	1.0E-46	2.0E-46	0.19	
K00760	hypoxanthine phosphoribosyltransferase [EC:2.4.2.8]	0.17	1.0E-46	2.0E-46	0.22	
K02533	tRNA/rRNA methyltransferase [EC:2.1.1.-]	0.17	1.2E-46	2.4E-46	0.20	
K00763	nicotinate phosphoribosyltransferase [EC:6.3.4.21]	0.17	1.4E-46	2.9E-46	0.18	
K00879	L-fuculokinase [EC:2.7.1.51]	0.17	1.7E-46	3.5E-46	0.17	
K05916	nitric oxide dioxygenase [EC:1.14.12.17]	0.17	2.0E-46	4.1E-46	0.20	
K03179	4-hydroxybenzoate polyprenyltransferase [EC:2.5.1.39]	0.17	2.3E-46	4.7E-46	0.18	
K13954	alcohol dehydrogenase [EC:1.1.1.1]	0.17	2.4E-46	4.9E-46	0.20	
K10815	hydrogen cyanide synthase HcnB [EC:1.4.99.5]	0.17	2.5E-46	5.1E-46	0.24	
K10816	hydrogen cyanide synthase HcnC [EC:1.4.99.5]	0.17	2.5E-46	5.1E-46	0.24	
K12453	CDP-paratose synthetase [EC:1.1.1.342]	0.17	2.6E-46	5.3E-46	0.18	yes
K00547	homocysteine S-methyltransferase [EC:2.1.1.10]	0.17	2.6E-46	5.3E-46	0.19	
K09251	putrescine aminotransferase [EC:2.6.1.82]	0.17	2.8E-46	5.7E-46	0.20	
K00574	cyclopropane-fatty-acyl-phospholipid synthase [EC:2.1.1.79]	0.17	3.5E-46	7.1E-46	0.17	
K00948	ribose-phosphate pyrophosphokinase [EC:2.7.6.1]	0.17	3.7E-46	7.5E-46	0.23	
K01920	glutathione synthase [EC:6.3.2.3]	0.17	4.5E-46	9.1E-46	0.20	
K07544	benzylsuccinate CoA-transferase BbsF subunit [EC:2.8.3.15]	0.17	4.7E-46	9.5E-46	0.24	
K15016	enoyl-CoA hydratase / 3-hydroxyacyl-CoA dehydrogenase [EC:4.2.1.17 1.1.1.35]	0.17	4.7E-46	9.5E-46	0.17	
K11637	two-component system, CitB family, sensor histidine kinase CitS [EC:2.7.13.3]	0.17	6.0E-46	1.2E-45	0.25	
K00968	choline-phosphate cytidylyltransferase [EC:2.7.7.15]	0.17	6.4E-46	1.3E-45	0.18	
K15257	tRNA (mo5U34)-methyltransferase [EC:2.1.1.-]	0.17	6.9E-46	1.4E-45	0.20	
K03981	thiol:disulfide interchange protein DsbC [EC:5.3.4.1]	0.17	8.0E-46	1.6E-45	0.20	
K10213	ribosylpyrimidine nucleosidase [EC:3.2.2.8]	0.17	8.5E-46	1.7E-45	0.19	
K00276	primary-amine oxidase [EC:1.4.3.21]	0.17	9.4E-46	1.9E-45	0.20	
K01465	dihydroorotase [EC:3.5.2.3]	0.17	9.8E-46	2.0E-45	0.22	
K10798	poly [ADP-ribose] polymerase 2/3/4 [EC:2.4.2.30]	0.17	1.2E-45	2.4E-45	0.21	
K01056	peptidyl-tRNA hydrolase, PTH1 family [EC:3.1.1.29]	0.17	1.2E-45	2.4E-45	0.22	
K03830	putative acetyltransferase [EC:2.3.1.-]	0.17	1.2E-45	2.4E-45	0.18	
K13663	acyltransferase [EC:2.3.1.-]	0.17	1.3E-45	2.6E-45	0.23	yes
K02798	mannitol PTS system EIIA component [EC:2.7.1.197]	0.17	1.6E-45	3.2E-45	0.19	

Predictor	Description	Pearson's r	P	FDR-adjusted P	Spearman's rho	Associated with fractures (P<0.05)
K00042	2-hydroxy-3-oxopropionate reductase [EC:1.1.1.60]	0.17	1.7E-45	3.4E-45	0.20	
K11611	meromycolic acid enoyl-[acyl-carrier-protein] reductase [EC:1.3.1.118]	0.17	1.9E-45	3.8E-45	0.24	
K16558	succinoglycan biosynthesis protein ExoL [EC:2.-.-.-]	0.17	1.9E-45	3.8E-45	0.22	
K02341	DNA polymerase III subunit delta' [EC:2.7.7.7]	0.17	1.9E-45	3.8E-45	0.22	
K10679	nitroreductase / dihydropteridine reductase [EC:1.-.-.- 1.5.1.34]	0.17	2.1E-45	4.2E-45	0.17	
K07318	adenine-specific DNA-methyltransferase [EC:2.1.1.72]	0.17	2.5E-45	5.0E-45	0.17	
K02010	iron(III) transport system ATP-binding protein [EC:7.2.2.7]	0.17	2.6E-45	5.2E-45	0.19	
K03738	aldehyde:ferredoxin oxidoreductase [EC:1.2.7.5]	0.17	2.7E-45	5.4E-45	0.21	
K00108	choline dehydrogenase [EC:1.1.99.1]	0.17	3.1E-45	6.2E-45	0.20	
K01682	aconitate hydratase 2 / 2-methylisocitrate dehydratase [EC:4.2.1.3 4.2.1.99]	0.17	3.1E-45	6.2E-45	0.20	
K07320	ribosomal protein L3 glutamine methyltransferase [EC:2.1.1.298]	0.17	4.1E-45	8.1E-45	0.20	
K02314	replicative DNA helicase [EC:3.6.4.12]	0.17	4.2E-45	8.3E-45	0.23	
K01971	bifunctional non-homologous end joining protein LigD [EC:6.5.1.1]	0.17	4.4E-45	8.7E-45	0.20	
K03119	taurine dioxygenase [EC:1.14.11.17]	0.17	4.6E-45	9.1E-45	0.20	
K15320	6-methylsalicylic acid synthase [EC:2.3.1.165]	0.17	4.7E-45	9.3E-45	0.22	
K07543	benzylsuccinate CoA-transferase BbsE subunit [EC:2.8.3.15]	0.17	4.8E-45	9.5E-45	0.23	
K01428	urease subunit alpha [EC:3.5.1.5]	0.17	4.8E-45	9.5E-45	0.18	
K02509	2-oxo-hept-3-ene-1,7-dioate hydratase [EC:4.2.1.-]	0.17	5.4E-45	1.1E-44	0.20	
K00564	16S rRNA (guanine1207-N2)-methyltransferase [EC:2.1.1.172]	0.17	5.5E-45	1.1E-44	0.17	
K01409	N6-L-threonylcarbamoyladenine synthase [EC:2.3.1.234]	0.17	5.8E-45	1.1E-44	0.22	
K00568	2-polyprenyl-6-hydroxyphenyl methylase / 3-demethylubiquinone-9 3-methyltransferase [EC:2.1.1.222 2.1.1.64]	0.17	6.2E-45	1.2E-44	0.20	
K03274	ADP-L-glycero-D-manno-heptose 6-epimerase [EC:5.1.3.20]	0.17	6.6E-45	1.3E-44	0.16	
K00096	glycerol-1-phosphate dehydrogenase [NAD(P)+] [EC:1.1.1.261]	0.17	7.5E-45	1.5E-44	0.17	
K00561	23S rRNA (adenine-N6)-dimethyltransferase [EC:2.1.1.184]	0.17	7.8E-45	1.5E-44	0.17	
K08081	tropinone reductase I [EC:1.1.1.206]	0.17	8.1E-45	1.6E-44	0.23	yes
K00949	thiamine pyrophosphokinase [EC:2.7.6.2]	0.17	9.0E-45	1.8E-44	0.17	
K00641	homoserine O-acetyltransferase/O-succinyltransferase [EC:2.3.1.31 2.3.1.46]	0.17	9.1E-45	1.8E-44	0.17	
K00817	histidinol-phosphate aminotransferase [EC:2.6.1.9]	0.17	9.3E-45	1.8E-44	0.21	
K15777	4,5-DOPA dioxygenase extradiol [EC:1.13.11.-]	0.17	9.3E-45	1.8E-44	0.19	
K01430	urease subunit gamma [EC:3.5.1.5]	0.17	9.5E-45	1.9E-44	0.18	
K10680	N-ethylmaleimide reductase [EC:1.-.-.-]	0.17	1.1E-44	2.2E-44	0.20	
K07644	two-component system, OmpR family, heavy metal sensor histidine kinase CusS [EC:2.7.13.3]	0.17	1.2E-44	2.4E-44	0.20	
K00989	ribonuclease PH [EC:2.7.7.56]	0.17	1.3E-44	2.5E-44	0.17	
K15761	toluene monooxygenase system protein B [EC:1.14.13.236 1.14.13.-]	0.17	1.4E-44	2.7E-44	0.23	yes
K03831	molybdopterin adenylyltransferase [EC:2.7.7.75]	0.17	2.0E-44	3.9E-44	0.19	
K01087	trehalose 6-phosphate phosphatase [EC:3.1.3.12]	0.17	2.1E-44	4.1E-44	0.19	
K01751	diaminopropionate ammonia-lyase [EC:4.3.1.15]	0.17	2.1E-44	4.1E-44	0.19	
K16559	endo-1,3-1,4-beta-glycanase ExoK [EC:3.2.1.-]	0.17	2.2E-44	4.3E-44	0.18	
K02291	15-cis-phytoene synthase [EC:2.5.1.32]	0.17	2.8E-44	5.5E-44	0.18	
K01684	galactonate dehydratase [EC:4.2.1.6]	0.17	3.0E-44	5.8E-44	0.19	
K15515	sulfoacetaldehyde dehydrogenase [EC:1.2.1.81]	0.17	3.2E-44	6.2E-44	0.28	
K01612	vanillate/4-hydroxybenzoate decarboxylase subunit C [EC:4.1.1.- 4.1.1.61]	0.16	3.8E-44	7.4E-44	0.19	

Predictor	Description	Pearson's r	P	FDR-adjusted P	Spearman's rho	Associated with fractures (P<0.05)
K07548	2-[hydroxy(phenyl)methyl]-succinyl-CoA dehydrogenase BbsD subunit [EC:1.1.1.35]	0.16	4.9E-44	9.5E-44	0.22	
K00826	branched-chain amino acid aminotransferase [EC:2.6.1.42]	0.16	5.1E-44	9.9E-44	0.21	
K01220	6-phospho-beta-galactosidase [EC:3.2.1.85]	0.16	5.3E-44	1.0E-43	0.17	
K15764	toluene monooxygenase system protein E [EC:1.14.13.236 1.14.13.-]	0.16	5.8E-44	1.1E-43	0.23	yes
K00835	valine--pyruvate aminotransferase [EC:2.6.1.66]	0.16	6.0E-44	1.2E-43	0.17	
K01466	allantoinase [EC:3.5.2.5]	0.16	6.8E-44	1.3E-43	0.20	
K00364	GMP reductase [EC:1.7.1.7]	0.16	8.3E-44	1.6E-43	0.17	
K01870	isoleucyl-tRNA synthetase [EC:6.1.1.5]	0.16	9.1E-44	1.8E-43	0.22	
K01829	disulfide reductase [EC:1.8.-.-]	0.16	9.3E-44	1.8E-43	0.24	yes
K01358	ATP-dependent Clp protease, protease subunit [EC:3.4.21.92]	0.16	9.5E-44	1.8E-43	0.22	
K02193	heme exporter protein A [EC:7.6.2.5]	0.16	1.1E-43	2.1E-43	0.19	
K14682	amino-acid N-acetyltransferase [EC:2.3.1.1]	0.16	1.5E-43	2.9E-43	0.19	
K00146	phenylacetaldehyde dehydrogenase [EC:1.2.1.39]	0.16	1.6E-43	3.1E-43	0.20	
K15514	3,4-dehydroadipyl-CoA semialdehyde dehydrogenase [EC:1.2.1.77]	0.16	1.9E-43	3.7E-43	0.23	yes
K07749	formyl-CoA transferase [EC:2.8.3.16]	0.16	1.9E-43	3.7E-43	0.18	
K12978	lipid A 4'-phosphatase [EC:3.1.3.-]	0.16	2.0E-43	3.9E-43	0.18	
K14368	3-alpha-mycarosylerythronolide B desosaminyl transferase [EC:2.4.1.278]	0.16	2.1E-43	4.0E-43	0.17	
K14366	6-deoxyerythronolide B hydroxylase [EC:1.14.15.35]	0.16	2.1E-43	4.0E-43	0.17	
K13311	dTDP-3-amino-3,4,6-trideoxy-alpha-D-glucopyranose N,N-dimethyltransferase [EC:2.1.1.234]	0.16	2.1E-43	4.0E-43	0.17	
K14370	erythromycin 12 hydroxylase [EC:1.14.13.154]	0.16	2.1E-43	4.0E-43	0.17	
K14369	erythromycin 3''-O-methyltransferase [EC:2.1.1.254]	0.16	2.1E-43	4.0E-43	0.17	
K03827	putative acetyltransferase [EC:2.3.1.-]	-0.16	2.5E-43	4.8E-43	-0.26	
K06281	hydrogenase large subunit [EC:1.12.99.6]	0.16	2.7E-43	5.2E-43	0.17	
K08305	membrane-bound lytic murein transglycosylase B [EC:4.2.2.-]	0.16	3.9E-43	7.5E-43	0.20	
K05816	sn-glycerol 3-phosphate transport system ATP-binding protein [EC:7.6.2.10]	0.16	4.6E-43	8.8E-43	0.18	
K01589	5-(carboxyamino)imidazole ribonucleotide synthase [EC:6.3.4.18]	0.16	5.2E-43	1.0E-42	0.17	
K00919	4-diphosphocytidyl-2-C-methyl-D-erythritol kinase [EC:2.7.1.148]	0.16	8.8E-43	1.7E-42	0.22	
K00101	L-lactate dehydrogenase (cytochrome) [EC:1.1.2.3]	0.16	9.8E-43	1.9E-42	0.20	
K03669	membrane glycosyltransferase [EC:2.4.1.-]	0.16	1.2E-42	2.3E-42	0.19	
K05522	endonuclease VIII [EC:3.2.2.- 4.2.99.18]	0.16	1.2E-42	2.3E-42	0.19	
K11610	beta-ketoacyl ACP reductase [EC:1.1.1.100]	0.16	1.6E-42	3.1E-42	0.23	
K02535	UDP-3-O-[3-hydroxymyristoyl] N-acetylglucosamine deacetylase [EC:3.5.1.108]	0.16	1.7E-42	3.2E-42	0.18	
K10778	AraC family transcriptional regulator, regulatory protein of adaptative response / methylated-DNA-[protein]-cysteine methyltransferase [EC:2.1.1.63]	0.16	1.7E-42	3.2E-42	0.20	
K13920	propanediol dehydratase small subunit [EC:4.2.1.28]	0.16	1.8E-42	3.4E-42	0.19	
K05363	serine/alanine adding enzyme [EC:2.3.2.10]	0.16	1.8E-42	3.4E-42	0.17	
K09887	dCTP deaminase (dUMP-forming) [EC:3.5.4.30]	0.16	2.0E-42	3.8E-42	0.15	
K14215	trans,polycis-decaprenyl diphosphate synthase [EC:2.5.1.86]	0.16	2.0E-42	3.8E-42	0.21	
K14261	alanine-synthesizing transaminase [EC:2.6.1.-]	0.16	2.0E-42	3.8E-42	0.19	
K08310	dihydroneopterin triphosphate diphosphatase [EC:3.6.1.67]	0.16	2.1E-42	4.0E-42	0.19	
K01782	3-hydroxyacyl-CoA dehydrogenase / enoyl-CoA hydratase / 3-hydroxybutyryl-CoA epimerase [EC:1.1.1.35 4.2.1.17 5.1.2.3]	0.16	2.3E-42	4.4E-42	0.19	
K02082	D-galactosamine 6-phosphate deaminase/isomerase [EC:3.5.99.-]	0.16	2.9E-42	5.5E-42	0.18	
K16043	scyllo-inositol 2-dehydrogenase (NAD+) [EC:1.1.1.370]	0.16	3.2E-42	6.1E-42	0.23	

Predictor	Description	Pearson's r	P	FDR-adjusted P	Spearman's rho	Associated with fractures (P<0.05)
K05823	N-acetyldiaminopimelate deacetylase [EC:3.5.1.47]	0.16	3.3E-42	6.2E-42	0.17	
K12555	penicillin-binding protein 2A [EC:2.4.1.129 3.4.16.4]	0.16	3.7E-42	7.0E-42	0.17	
K13497	anthranilate synthase/phosphoribosyltransferase [EC:4.1.3.27 2.4.2.18]	0.16	6.1E-42	1.2E-41	0.17	
K06282	hydrogenase small subunit [EC:1.12.99.6]	0.16	7.1E-42	1.3E-41	0.17	
K13786	cob(II)yrinic acid a,c-diamide reductase [EC:1.16.8.-]	0.16	8.1E-42	1.5E-41	0.19	
K03826	putative acetyltransferase [EC:2.3.1.-]	0.16	1.1E-41	2.1E-41	0.16	
K00853	L-ribulokinase [EC:2.7.1.16]	0.16	1.3E-41	2.5E-41	0.17	
K15054	(S)-mandelate dehydrogenase [EC:1.1.99.31]	0.16	1.4E-41	2.6E-41	0.23	
K01664	para-aminobenzoate synthetase component II [EC:2.6.1.85]	0.16	1.5E-41	2.8E-41	0.17	
K01674	carbonic anhydrase [EC:4.2.1.1]	0.16	1.7E-41	3.2E-41	0.18	
K02339	DNA polymerase III subunit chi [EC:2.7.7.7]	0.16	1.7E-41	3.2E-41	0.19	
K02302	uroporphyrin-III C-methyltransferase / precorrin-2 dehydrogenase / sirohydrochlorin ferrochelatase [EC:2.1.1.107 1.3.1.76 4.99.1.4]	0.16	2.2E-41	4.1E-41	0.19	
K01608	tartronate-semialdehyde synthase [EC:4.1.1.47]	0.16	2.6E-41	4.9E-41	0.19	
K07708	two-component system, NtrC family, nitrogen regulation sensor histidine kinase GlnL [EC:2.7.13.3]	0.16	2.7E-41	5.1E-41	0.19	
K08281	nicotinamidase/pyrazinamidase [EC:3.5.1.19 3.5.1.-]	0.16	3.6E-41	6.8E-41	0.17	
K10817	6-deoxyerythronolide-B synthase EryAI [EC:2.3.1.94]	0.16	3.9E-41	7.3E-41	0.17	
K03391	pentachlorophenol monooxygenase [EC:1.14.13.50]	0.16	3.9E-41	7.3E-41	0.17	
K01071	medium-chain acyl-[acyl-carrier-protein] hydrolase [EC:3.1.2.21]	0.16	3.9E-41	7.3E-41	0.16	
K16328	pseudouridine kinase [EC:2.7.1.83]	0.16	5.9E-41	1.1E-40	0.19	
K00960	DNA-directed RNA polymerase subunit 13 [EC:2.7.7.6]	0.16	6.4E-41	1.2E-40	0.17	
K03417	methylisocitrate lyase [EC:4.1.3.30]	0.16	7.2E-41	1.3E-40	0.19	
K07246	tartrate dehydrogenase/decarboxylase / D-malate dehydrogenase [EC:1.1.1.93 4.1.1.73 1.1.1.83]	0.16	9.3E-41	1.7E-40	0.19	
K00383	glutathione reductase (NADPH) [EC:1.8.1.7]	0.16	1.0E-40	1.9E-40	0.17	
K13609	delta1-piperideine-2-carboxylate reductase [EC:1.5.1.21]	0.16	1.1E-40	2.1E-40	0.23	
K00758	thymidine phosphorylase [EC:2.4.2.4]	0.16	1.4E-40	2.6E-40	0.19	
K00930	acetylglutamate kinase [EC:2.7.2.8]	0.16	1.5E-40	2.8E-40	0.22	
K03185	2-octaprenyl-6-methoxyphenol hydroxylase [EC:1.14.13.-]	0.16	1.5E-40	2.8E-40	0.19	
K01852	lanosterol synthase [EC:5.4.99.7]	0.16	1.9E-40	3.5E-40	0.23	
K03418	N,N-dimethylformamidase large subunit [EC:3.5.1.56]	0.16	2.0E-40	3.7E-40	0.22	yes
K02299	cytochrome o ubiquinol oxidase subunit III	0.16	2.0E-40	3.7E-40	0.19	
K05275	pyridoxine 4-dehydrogenase [EC:1.1.1.65]	0.16	2.1E-40	3.9E-40	0.18	
K01890	phenylalanyl-tRNA synthetase beta chain [EC:6.1.1.20]	0.16	2.1E-40	3.9E-40	0.21	
K07127	5-hydroxyisourate hydrolase [EC:3.5.2.17]	0.16	2.1E-40	3.9E-40	0.19	
K01638	malate synthase [EC:2.3.3.9]	0.16	2.2E-40	4.1E-40	0.19	
K12993	O-antigen biosynthesis alpha-1,2-mannosyltransferase [EC:2.4.1.371 2.4.1.-]	0.16	2.6E-40	4.8E-40	0.18	
K01699	propanediol dehydratase large subunit [EC:4.2.1.28]	0.16	2.7E-40	5.0E-40	0.19	
K01281	X-Pro dipeptidyl-peptidase [EC:3.4.14.11]	0.16	3.2E-40	5.9E-40	0.16	
K01720	2-methylcitrate dehydratase [EC:4.2.1.79]	0.16	3.2E-40	5.9E-40	0.19	
K00556	tRNA (guanosine-2'-O-)-methyltransferase [EC:2.1.1.34]	0.16	3.8E-40	7.0E-40	0.17	
K05928	tocopherol O-methyltransferase [EC:2.1.1.95]	0.16	4.0E-40	7.4E-40	0.20	
K12997	rhamnosyltransferase [EC:2.4.1.-]	0.16	4.1E-40	7.6E-40	0.16	
K02170	pimeloyl-[acyl-carrier protein] methyl ester esterase [EC:3.1.1.85]	0.16	4.4E-40	8.1E-40	0.19	

Predictor	Description	Pearson's r	P	FDR-adjusted P	Spearman's rho	Associated with fractures (P<0.05)
K05910	NADH peroxidase [EC:1.11.1.1]	0.16	4.6E-40	8.5E-40	0.18	
K00694	cellulose synthase (UDP-forming) [EC:2.4.1.12]	0.16	4.7E-40	8.7E-40	0.19	
K00765	ATP phosphoribosyltransferase [EC:2.4.2.17]	0.16	5.3E-40	9.8E-40	0.22	
K01426	amidase [EC:3.5.1.4]	0.16	5.8E-40	1.1E-39	0.17	
K01615	glutaconyl-CoA decarboxylase subunit alpha [EC:7.2.4.5]	0.16	7.0E-40	1.3E-39	0.21	
K01681	aconitate hydratase [EC:4.2.1.3]	0.16	8.2E-40	1.5E-39	0.15	
K03582	exodeoxyribonuclease V beta subunit [EC:3.1.11.5]	0.16	8.5E-40	1.6E-39	0.19	
K10837	O-phosphoseryl-tRNA(Sec) kinase [EC:2.7.1.164]	0.16	8.8E-40	1.6E-39	0.15	
K04479	DNA polymerase IV (archaeal DinB-like DNA polymerase) [EC:2.7.7.7]	0.16	9.3E-40	1.7E-39	0.19	
K00525	ribonucleoside-diphosphate reductase alpha chain [EC:1.17.4.1]	-0.16	9.9E-40	1.8E-39	-0.26	
K00543	acetylserotonin O-methyltransferase [EC:2.1.1.4]	0.16	1.1E-39	2.0E-39	0.22	
K14681	NA	0.16	1.1E-39	2.0E-39	0.21	
K01662	1-deoxy-D-xylulose-5-phosphate synthase [EC:2.2.1.7]	-0.16	1.4E-39	2.6E-39	-0.27	
K15038	succinyl-CoA reductase [EC:1.2.1.76]	0.16	1.5E-39	2.7E-39	0.15	
K00849	galactokinase [EC:2.7.1.6]	0.16	2.0E-39	3.7E-39	0.22	
K15527	cysteate synthase [EC:2.5.1.76]	0.16	2.2E-39	4.0E-39	0.17	
K03224	ATP synthase in type III secretion protein N [EC:7.4.2.8]	0.16	2.8E-39	5.1E-39	0.19	
K04035	magnesium-protoporphyrin IX monomethyl ester (oxidative) cyclase [EC:1.14.13.81]	0.16	2.8E-39	5.1E-39	0.23	
K01704	3-isopropylmalate/(R)-2-methylmalate dehydratase small subunit [EC:4.2.1.33 4.2.1.35]	0.16	3.3E-39	6.0E-39	0.20	
K03656	ATP-dependent DNA helicase Rep [EC:3.6.4.12]	0.16	3.4E-39	6.2E-39	0.19	
K01565	N-sulfoglucosamine sulfohydrolase [EC:3.10.1.1]	0.16	3.9E-39	7.1E-39	0.17	
K07317	adenine-specific DNA-methyltransferase [EC:2.1.1.72]	0.15	4.5E-39	8.2E-39	0.19	
K11440	choline dehydrogenase [EC:1.1.1.1]	0.15	4.9E-39	8.9E-39	0.23	
K06968	23S rRNA (cytidine2498-2'-O)-methyltransferase [EC:2.1.1.186]	0.15	5.0E-39	9.1E-39	0.18	
K01261	glutamyl aminopeptidase [EC:3.4.11.7]	0.15	5.2E-39	9.5E-39	0.16	
K11609	beta-ketoacyl ACP synthase [EC:2.3.1.293 2.3.1.294]	0.15	5.4E-39	9.8E-39	0.22	
K02781	glucitol/sorbitol PTS system EIIA component [EC:2.7.1.198]	0.15	5.6E-39	1.0E-38	0.17	
K00384	thioredoxin reductase (NADPH) [EC:1.8.1.9]	0.15	7.6E-39	1.4E-38	0.21	
K12661	L-rhamnonate dehydratase [EC:4.2.1.90]	0.15	9.5E-39	1.7E-38	0.18	
K00795	farnesyl diphosphate synthase [EC:2.5.1.1 2.5.1.10]	0.15	9.6E-39	1.7E-38	0.19	
K13797	DNA-directed RNA polymerase subunit beta-beta' [EC:2.7.7.6]	0.15	9.8E-39	1.8E-38	0.20	
K08304	membrane-bound lytic murein transglycosylase A [EC:4.2.2.-]	0.15	1.3E-38	2.4E-38	0.19	
K01073	acyl-CoA hydrolase [EC:3.1.2.20]	0.15	1.3E-38	2.4E-38	0.22	
K00712	poly(glycerol-phosphate) alpha-glucosyltransferase [EC:2.4.1.52]	0.15	1.4E-38	2.5E-38	0.17	
K04568	elongation factor P--(R)-beta-lysine ligase [EC:6.3.1.-]	0.15	1.4E-38	2.5E-38	0.18	
K01483	ureidoglycolate lyase [EC:4.3.2.3]	0.15	1.6E-38	2.9E-38	0.18	
K02549	o-succinylbenzoate synthase [EC:4.2.1.113]	0.15	1.6E-38	2.9E-38	0.16	
K15754	2'-aminobiphenyl-2,3-diol 1,2-dioxygenase, small subunit [EC:1.13.11.-]	0.15	1.7E-38	3.1E-38	0.21	yes
K02474	UDP-N-acetyl-D-glucosamine/UDP-N-acetyl-D-galactosamine dehydrogenase [EC:1.1.1.136 1.1.1.-]	0.15	1.7E-38	3.1E-38	0.16	
K01256	aminopeptidase N [EC:3.4.11.2]	0.15	1.7E-38	3.1E-38	0.15	
K15781	putative phosphoserine phosphatase / 1-acylglycerol-3-phosphate O-acyltransferase [EC:3.1.3.3 2.3.1.51]	0.15	2.0E-38	3.6E-38	0.22	yes
K07389	cytolysin-activating lysine-acyltransferase [EC:2.3.1.-]	0.15	2.1E-38	3.8E-38	0.17	

Predictor	Description	Pearson's r	P	FDR-adjusted P	Spearman's rho	Associated with fractures (P<0.05)
K08316	16S rRNA (guanine966-N2)-methyltransferase [EC:2.1.1.171]	0.15	2.1E-38	3.8E-38	0.16	
K03672	thioredoxin 2 [EC:1.8.1.8]	0.15	2.3E-38	4.1E-38	0.19	
K01591	orotidine-5'-phosphate decarboxylase [EC:4.1.1.23]	0.15	2.4E-38	4.3E-38	0.22	
K01525	bis(5'-nucleosyl)-tetraphosphatase (symmetrical) [EC:3.6.1.41]	0.15	2.8E-38	5.0E-38	0.18	
K01581	ornithine decarboxylase [EC:4.1.1.17]	0.15	4.2E-38	7.5E-38	0.16	
K11441	dehydrogluconokinase [EC:2.7.1.13]	0.15	4.2E-38	7.5E-38	0.21	
K04032	ethanolamine utilization cobalamin adenosyltransferase [EC:2.5.1.17]	0.15	4.2E-38	7.5E-38	0.18	
K07642	two-component system, OmpR family, sensor histidine kinase BaeS [EC:2.7.13.3]	0.15	4.7E-38	8.4E-38	0.18	
K01753	D-serine dehydratase [EC:4.3.1.18]	0.15	5.7E-38	1.0E-37	0.18	
K01716	3-hydroxyacyl-[acyl-carrier protein] dehydratase / trans-2-decenoyl-[acyl-carrier protein] isomerase [EC:4.2.1.59 5.3.3.14]	0.15	6.2E-38	1.1E-37	0.18	
K14661	nodulation protein F [EC:2.3.1.-]	0.15	7.5E-38	1.3E-37	0.16	
K11385	arabinosyltransferase A [EC:2.4.2.-]	0.15	8.2E-38	1.5E-37	0.21	
K01792	glucose-6-phosphate 1-epimerase [EC:5.1.3.15]	0.15	8.4E-38	1.5E-37	0.16	
K14469	acrylyl-CoA reductase (NADPH) / 3-hydroxypropionyl-CoA dehydratase / 3-hydroxypropionyl-CoA synthetase [EC:1.3.1.84 4.2.1.116 6.2.1.36]	0.15	1.1E-37	2.0E-37	0.18	
K00867	type I pantothenate kinase [EC:2.7.1.33]	0.15	1.2E-37	2.1E-37	0.15	
K03501	16S rRNA (guanine527-N7)-methyltransferase [EC:2.1.1.170]	-0.15	1.3E-37	2.3E-37	-0.24	
K01659	2-methylcitrate synthase [EC:2.3.3.5]	0.15	1.3E-37	2.3E-37	0.18	
K00692	levansucrase [EC:2.4.1.10]	0.15	2.3E-37	4.1E-37	0.21	
K03342	para-aminobenzoate synthetase / 4-amino-4-deoxychorismate lyase [EC:2.6.1.85 4.1.3.38]	0.15	3.0E-37	5.3E-37	0.17	
K12554	alanine adding enzyme [EC:2.3.2.-]	0.15	3.4E-37	6.0E-37	0.16	
K01147	exoribonuclease II [EC:3.1.13.1]	0.15	3.7E-37	6.6E-37	0.18	
K13919	propanediol dehydratase medium subunit [EC:4.2.1.28]	0.15	4.2E-37	7.5E-37	0.18	
K02745	N-acetylgalactosamine PTS system EIIB component [EC:2.7.1.-]	0.15	4.2E-37	7.5E-37	0.17	
K15755	2'-aminobiphenyl-2,3-diol 1,2-dioxygenase, large subunit [EC:1.13.11.-]	0.15	4.8E-37	8.5E-37	0.20	yes
K00700	1,4-alpha-glucan branching enzyme [EC:2.4.1.18]	0.15	5.1E-37	9.0E-37	0.22	yes
K09019	3-hydroxypropanoate dehydrogenase [EC:1.1.1.-]	0.15	5.5E-37	9.7E-37	0.18	
K01639	N-acetylneuraminate lyase [EC:4.1.3.3]	-0.15	5.8E-37	1.0E-36	-0.24	
K00630	glycerol-3-phosphate O-acyltransferase [EC:2.3.1.15]	0.15	5.9E-37	1.0E-36	0.14	
K01908	propionyl-CoA synthetase [EC:6.2.1.17]	0.15	6.1E-37	1.1E-36	0.18	
K03692	glucosylglycerol-phosphate synthase [EC:2.4.1.213]	0.15	7.7E-37	1.4E-36	0.17	
K13953	alcohol dehydrogenase, propanol-preferring [EC:1.1.1.1]	0.15	7.8E-37	1.4E-36	0.16	
K10916	two-component system, CAI-1 autoinducer sensor kinase/phosphatase CqsS [EC:2.7.13.3 3.1.3.-]	0.15	8.2E-37	1.4E-36	0.21	
K05964	holo-ACP synthase [EC:2.7.7.61]	0.15	9.2E-37	1.6E-36	0.17	
K01361	lactocepin [EC:3.4.21.96]	0.15	9.7E-37	1.7E-36	0.15	
K00052	3-isopropylmalate dehydrogenase [EC:1.1.1.85]	0.15	1.0E-36	1.8E-36	0.20	
K01846	methylaspartate mutase sigma subunit [EC:5.4.99.1]	0.15	1.1E-36	1.9E-36	0.18	
K14188	D-alanine--poly(phosphoribitol) ligase subunit 2 [EC:6.1.1.13]	0.15	1.1E-36	1.9E-36	0.15	
K04765	nucleoside triphosphate diphosphatase [EC:3.6.1.9]	0.15	1.3E-36	2.3E-36	0.18	
K03824	putative acetyltransferase [EC:2.3.1.-]	0.15	1.3E-36	2.3E-36	0.18	
K01690	phosphogluconate dehydratase [EC:4.2.1.12]	0.15	1.4E-36	2.5E-36	0.18	
K14977	(S)-ureidoglycine aminohydrolase [EC:3.5.3.26]	0.15	1.4E-36	2.5E-36	0.18	
K13498	indole-3-glycerol phosphate synthase / phosphoribosylanthranilate isomerase [EC:4.1.1.48 5.3.1.24]	0.15	1.5E-36	2.6E-36	0.16	

Predictor	Description	Pearson's r	P	FDR-adjusted P	Spearman's rho	Associated with fractures (P<0.05)
K01974	RNA 3'-terminal phosphate cyclase (ATP) [EC:6.5.1.4]	0.15	1.6E-36	2.8E-36	0.17	
K07336	PKHD-type hydroxylase [EC:1.14.11.-]	0.15	2.1E-36	3.7E-36	0.18	
K00493	xanthocillin biosynthesis cytochrome P450 monooxygenase [EC:1.14.-.-]	0.15	2.1E-36	3.7E-36	0.22	
K00982	[glutamine synthetase] adenylyltransferase / [glutamine synthetase]-adenylyl-L-tyrosine phosphorylase [EC:2.7.7.42 2.7.7.89]	0.15	2.1E-36	3.7E-36	0.16	
K13061	acyl homoserine lactone synthase [EC:2.3.1.184]	0.15	2.4E-36	4.2E-36	0.21	
K03476	L-ascorbate 6-phosphate lactonase [EC:3.1.1.-]	0.15	2.5E-36	4.4E-36	0.17	
K00918	ADP-dependent phosphofructokinase/glucokinase [EC:2.7.1.146 2.7.1.147]	0.15	3.0E-36	5.2E-36	0.22	
K02821	ascorbate PTS system EIIA or EIIAB component [EC:2.7.1.194]	0.15	3.1E-36	5.4E-36	0.15	
K02769	fructose PTS system EIIB component [EC:2.7.1.202]	0.15	3.5E-36	6.1E-36	0.16	
K05712	3-(3-hydroxy-phenyl)propionate hydroxylase [EC:1.14.13.127]	0.15	3.7E-36	6.5E-36	0.18	
K08300	ribonuclease E [EC:3.1.26.12]	0.15	4.0E-36	7.0E-36	0.16	
K08356	arsenite oxidase large subunit [EC:1.20.2.1 1.20.9.1]	0.15	4.1E-36	7.2E-36	0.22	
K16148	alpha-maltose-1-phosphate synthase [EC:2.4.1.342]	0.15	4.2E-36	7.3E-36	0.16	
K01120	3',5'-cyclic-nucleotide phosphodiesterase [EC:3.1.4.17]	0.15	6.0E-36	1.0E-35	0.22	
K07709	two-component system, NtrC family, sensor histidine kinase HydH [EC:2.7.13.3]	0.15	6.4E-36	1.1E-35	0.17	
K10806	acyl-CoA thioesterase YciA [EC:3.1.2.-]	0.15	8.2E-36	1.4E-35	0.18	
K00137	aminobutyraldehyde dehydrogenase [EC:1.2.1.19]	0.15	8.9E-36	1.5E-35	0.17	
K02083	allantoate deiminase [EC:3.5.3.9]	0.15	1.3E-35	2.3E-35	0.17	
K03732	ATP-dependent RNA helicase RhlB [EC:3.6.4.13]	0.15	1.4E-35	2.4E-35	0.18	
K10545	D-xylose transport system ATP-binding protein [EC:7.5.2.10]	0.15	1.5E-35	2.6E-35	0.18	
K01226	trehalose-6-phosphate hydrolase [EC:3.2.1.93]	0.15	2.2E-35	3.8E-35	0.15	
K03341	O-phospho-L-seryl-tRNASec:L-selenocysteinyl-tRNA synthase [EC:2.9.1.2]	0.15	2.3E-35	4.0E-35	0.14	
K02822	ascorbate PTS system EIIB component [EC:2.7.1.194]	0.15	2.5E-35	4.3E-35	0.15	
K03583	exodeoxyribonuclease V gamma subunit [EC:3.1.11.5]	0.15	2.6E-35	4.5E-35	0.18	
K02774	galactitol PTS system EIIB component [EC:2.7.1.200]	0.15	3.1E-35	5.4E-35	0.17	
K08884	serine/threonine protein kinase, bacterial [EC:2.7.11.1]	0.15	3.3E-35	5.7E-35	0.18	
K00123	formate dehydrogenase major subunit [EC:1.17.1.9]	0.15	3.8E-35	6.6E-35	0.17	
K10536	agmatine deiminase [EC:3.5.3.12]	0.15	4.0E-35	6.9E-35	0.16	
K04071	NA	0.15	4.5E-35	7.8E-35	0.16	
K01575	acetolactate decarboxylase [EC:4.1.1.5]	0.15	5.0E-35	8.6E-35	0.15	
K01437	aspartoacylase [EC:3.5.1.15]	0.15	5.7E-35	9.8E-35	0.22	
K11617	two-component system, NarL family, sensor histidine kinase LiaS [EC:2.7.13.3]	0.15	5.9E-35	1.0E-34	0.16	
K03801	lipoyl(octanoyl) transferase [EC:2.3.1.181]	0.15	6.3E-35	1.1E-34	0.15	
K00785	N-acetyllactosaminide alpha-2,3-sialyltransferase [EC:2.4.99.6]	0.15	6.8E-35	1.2E-34	0.20	
K00204	4Fe-4S ferredoxin	0.15	7.7E-35	1.3E-34	0.14	
K03471	ribonuclease HIII [EC:3.1.26.4]	0.15	9.6E-35	1.7E-34	0.16	
K01408	insulysin [EC:3.4.24.56]	0.15	1.1E-34	1.9E-34	0.14	
K01928	UDP-N-acetylmuramoyl-L-alanyl-D-glutamate--2,6-diaminopimelate ligase [EC:6.3.2.13]	0.15	1.2E-34	2.1E-34	0.20	
K04486	histidinol-phosphatase (PHP family) [EC:3.1.3.15]	0.15	1.3E-34	2.2E-34	0.15	
K02491	two-component system, sporulation sensor kinase A [EC:2.7.13.3]	0.15	1.4E-34	2.4E-34	0.22	
K00076	7-alpha-hydroxysteroid dehydrogenase [EC:1.1.1.159]	0.15	1.6E-34	2.7E-34	0.16	
K04835	methylaspartate ammonia-lyase [EC:4.3.1.2]	0.15	1.7E-34	2.9E-34	0.17	

Predictor	Description	Pearson's r	P	FDR-adjusted P	Spearman's rho	Associated with fractures (P<0.05)
K00322	NAD(P) transhydrogenase [EC:1.6.1.1]	0.15	1.7E-34	2.9E-34	0.18	
K00371	nitrate reductase / nitrite oxidoreductase, beta subunit [EC:1.7.5.1 1.7.99.-]	0.15	2.1E-34	3.6E-34	0.16	
K16301	deferrochelatase/peroxidase EfeB [EC:1.11.1.-]	0.14	2.1E-34	3.6E-34	0.16	
K13378	NADH-quinone oxidoreductase subunit C/D [EC:7.1.1.2]	-0.14	2.6E-34	4.4E-34	-0.26	
K05539	tRNA-dihydrouridine synthase A [EC:1.-.-.-]	0.14	2.9E-34	5.0E-34	0.17	
K13532	two-component system, sporulation sensor kinase D [EC:2.7.13.3]	0.14	3.2E-34	5.5E-34	0.22	
K01002	phosphoglycerol transferase [EC:2.7.8.20]	0.14	3.3E-34	5.6E-34	0.16	
K00526	ribonucleoside-diphosphate reductase beta chain [EC:1.17.4.1]	-0.14	3.8E-34	6.5E-34	-0.26	
K01867	tryptophanyl-tRNA synthetase [EC:6.1.1.2]	0.14	4.2E-34	7.2E-34	0.20	
K00769	xanthine phosphoribosyltransferase [EC:2.4.2.22]	0.14	4.4E-34	7.5E-34	0.17	
K02844	UDP-glucose:(heptosyl)LPS alpha-1,3-glucosyltransferase [EC:2.4.1.-]	0.14	4.5E-34	7.7E-34	0.17	
K02552	menaquinone-specific isochorismate synthase [EC:5.4.4.2]	0.14	4.9E-34	8.3E-34	0.15	
K03825	L-phenylalanine/L-methionine N-acetyltransferase [EC:2.3.1.53 2.3.1.-]	0.14	5.0E-34	8.5E-34	0.17	
K02773	galactitol PTS system EIIA component [EC:2.7.1.200]	0.14	5.5E-34	9.4E-34	0.16	
K11931	poly-beta-1,6-N-acetyl-D-glucosamine N-deacetylase [EC:3.5.1.-]	0.14	6.8E-34	1.2E-33	0.16	
K01141	exodeoxyribonuclease I [EC:3.1.11.1]	0.14	7.2E-34	1.2E-33	0.17	
K03919	DNA oxidative demethylase [EC:1.14.11.33]	0.14	1.3E-33	2.2E-33	0.17	
K00595	precorrin-6B C5,15-methyltransferase / cobalt-precorrin-6B C5,C15-methyltransferase [EC:2.1.1.132 2.1.1.289 2.1.1.196]	0.14	1.5E-33	2.5E-33	0.15	
K10563	formamidopyrimidine-DNA glycosylase [EC:3.2.2.23 4.2.99.18]	0.14	1.5E-33	2.5E-33	0.14	
K09994	(aminoalkyl)phosphonate N-acetyltransferase [EC:2.3.1.280]	0.14	2.1E-33	3.6E-33	0.16	
K15587	nickel transport system ATP-binding protein [EC:7.2.2.11]	0.14	2.1E-33	3.6E-33	0.17	
K07641	two-component system, OmpR family, sensor histidine kinase CreC [EC:2.7.13.3]	0.14	2.2E-33	3.7E-33	0.16	
K13829	shikimate kinase / 3-dehydroquinate synthase [EC:2.7.1.71 4.2.3.4]	0.14	2.3E-33	3.9E-33	0.15	
K10831	taurine transport system ATP-binding protein [EC:7.6.2.7]	0.14	2.8E-33	4.7E-33	0.16	
K15984	16S rRNA (guanine1516-N2)-methyltransferase [EC:2.1.1.242]	0.14	3.0E-33	5.1E-33	0.17	
K01584	arginine decarboxylase [EC:4.1.1.19]	0.14	4.2E-33	7.1E-33	0.17	
K02554	2-keto-4-pentenoate hydratase [EC:4.2.1.80]	0.14	4.4E-33	7.4E-33	0.17	
K00640	serine O-acetyltransferase [EC:2.3.1.30]	-0.14	5.3E-33	8.9E-33	-0.24	
K00656	formate C-acetyltransferase [EC:2.3.1.54]	-0.14	6.0E-33	1.0E-32	-0.24	
K00216	2,3-dihydro-2,3-dihydroxybenzoate dehydrogenase [EC:1.3.1.28]	0.14	6.1E-33	1.0E-32	0.16	
K07638	two-component system, OmpR family, osmolarity sensor histidine kinase EnvZ [EC:2.7.13.3]	0.14	6.4E-33	1.1E-32	0.17	
K00631	glycerol-3-phosphate O-acyltransferase [EC:2.3.1.15]	0.14	7.2E-33	1.2E-32	0.17	
K02371	enoyl-[acyl-carrier protein] reductase II [EC:1.3.1.9]	0.14	7.3E-33	1.2E-32	0.15	
K13713	fusion protein PurCD [EC:6.3.2.6 6.3.4.13]	0.14	1.0E-32	1.7E-32	0.17	yes
K00881	allose kinase [EC:2.7.1.55]	0.14	1.1E-32	1.8E-32	0.15	
K00374	nitrate reductase gamma subunit [EC:1.7.5.1 1.7.99.-]	0.14	1.2E-32	2.0E-32	0.16	
K13439	cysteine protease avirulence protein AvrRpt2 [EC:3.4.22.-]	0.14	1.3E-32	2.2E-32	0.14	
K05365	penicillin-binding protein 1B [EC:2.4.1.129 3.4.16.4]	0.14	1.4E-32	2.3E-32	0.17	
K00511	squalene monooxygenase [EC:1.14.14.17]	0.14	1.5E-32	2.5E-32	0.22	
K03046	DNA-directed RNA polymerase subunit beta' [EC:2.7.7.6]	0.14	2.0E-32	3.4E-32	0.19	
K12700	non-specific riboncleoside hydrolase [EC:3.2.-.-]	0.14	2.3E-32	3.9E-32	0.15	
K01304	pyroglutamyl-peptidase [EC:3.4.19.3]	0.14	2.5E-32	4.2E-32	0.14	

Predictor	Description	Pearson's r	P	FDR-adjusted P	Spearman's rho	Associated with fractures (P<0.05)
K05878	phosphoenolpyruvate---glycerone phosphotransferase subunit DhaK [EC:2.7.1.121]	0.14	2.6E-32	4.4E-32	0.15	
K12267	peptide methionine sulfoxide reductase msrA/msrB [EC:1.8.4.11 1.8.4.12]	0.14	3.0E-32	5.0E-32	0.14	
K00806	undecaprenyl diphosphate synthase [EC:2.5.1.31]	0.14	3.1E-32	5.2E-32	0.19	
K09471	gamma-glutamylputrescine oxidase [EC:1.4.3.-]	0.14	3.4E-32	5.7E-32	0.17	
K00288	methylenetetrahydrofolate dehydrogenase (NADP+) / methenyltetrahydrofolate cyclohydrolase / formyltetrahydrofolate synthetase [EC:1.5.1.5 3.5.4.9 6.3.4.3]	0.14	3.7E-32	6.2E-32	0.18	
K15461	tRNA 5-methylaminomethyl-2-thiouridine biosynthesis bifunctional protein [EC:2.1.1.61 1.5.-.-]	0.14	4.1E-32	6.8E-32	0.17	
K01708	galactarate dehydratase [EC:4.2.1.42]	0.14	4.2E-32	7.0E-32	0.17	
K05599	anthranilate 1,2-dioxygenase (deaminating, decarboxylating) large subunit [EC:1.14.12.1]	0.14	5.2E-32	8.7E-32	0.18	
K05600	anthranilate 1,2-dioxygenase (deaminating, decarboxylating) small subunit [EC:1.14.12.1]	0.14	5.2E-32	8.7E-32	0.18	
K00156	pyruvate dehydrogenase (quinone) [EC:1.2.5.1]	0.14	5.4E-32	9.0E-32	0.15	
K02478	two-component system, LytTR family, sensor kinase [EC:2.7.13.3]	0.14	6.0E-32	1.0E-31	0.17	
K06175	tRNA pseudouridine65 synthase [EC:5.4.99.26]	0.14	6.3E-32	1.0E-31	0.17	
K01739	cystathionine gamma-synthase [EC:2.5.1.48]	0.14	6.3E-32	1.0E-31	0.14	
K00370	nitrate reductase / nitrite oxidoreductase, alpha subunit [EC:1.7.5.1 1.7.99.-]	0.14	6.7E-32	1.1E-31	0.15	
K01315	plasminogen [EC:3.4.21.7]	0.14	7.1E-32	1.2E-31	0.12	
K10210	diapolycopene oxygenase [EC:1.14.99.44]	0.14	9.3E-32	1.5E-31	0.14	
K01646	citrate lyase subunit gamma (acyl carrier protein)	0.14	1.0E-31	1.7E-31	0.15	
K03101	signal peptidase II [EC:3.4.23.36]	0.14	1.0E-31	1.7E-31	0.22	
K12972	glyoxylate/hydroxypyruvate reductase [EC:1.1.1.79 1.1.1.81]	0.14	1.0E-31	1.7E-31	0.17	
K03922	acyl-[acyl-carrier-protein] desaturase [EC:1.14.19.2]	0.14	1.2E-31	2.0E-31	0.18	
K03828	putative acetyltransferase [EC:2.3.1.-]	0.14	1.3E-31	2.2E-31	0.16	
K11753	riboflavin kinase / FMN adenylyltransferase [EC:2.7.1.26 2.7.7.2]	0.14	1.4E-31	2.3E-31	0.19	
K06912	alpha-ketoglutarate-dependent 2,4-dichlorophenoxyacetate dioxygenase [EC:1.14.11.-]	0.14	1.7E-31	2.8E-31	0.18	
K05879	phosphoenolpyruvate---glycerone phosphotransferase subunit DhaL [EC:2.7.1.121]	0.14	2.0E-31	3.3E-31	0.16	
K14287	methionine transaminase [EC:2.6.1.88]	0.14	2.2E-31	3.6E-31	0.17	
K00557	tRNA (uracil-5-)-methyltransferase [EC:2.1.1.35]	0.14	2.4E-31	4.0E-31	0.16	
K08092	3-dehydro-L-gulonate 2-dehydrogenase [EC:1.1.1.130]	0.14	2.6E-31	4.3E-31	0.16	
K05308	gluconate/galactonate dehydratase [EC:4.2.1.140]	0.14	2.6E-31	4.3E-31	0.13	
K00675	N-hydroxyarylamine O-acetyltransferase [EC:2.3.1.118]	0.14	2.8E-31	4.6E-31	0.15	
K14658	nodulation protein A [EC:2.3.1.-]	0.14	3.0E-31	4.9E-31	0.21	yes
K08484	phosphotransferase system, enzyme I, PtsP [EC:2.7.3.9]	0.14	3.1E-31	5.1E-31	0.16	
K00855	phosphoribulokinase [EC:2.7.1.19]	0.14	3.4E-31	5.6E-31	0.16	
K01788	N-acylglucosamine-6-phosphate 2-epimerase [EC:5.1.3.9]	0.14	4.8E-31	7.9E-31	0.15	
K05886	serine 3-dehydrogenase (NADP+) [EC:1.1.1.276]	0.14	5.1E-31	8.4E-31	0.21	yes
K07678	two-component system, NarL family, sensor histidine kinase BarA [EC:2.7.13.3]	0.14	5.3E-31	8.7E-31	0.17	
K01902	succinyl-CoA synthetase alpha subunit [EC:6.2.1.5]	0.14	5.3E-31	8.7E-31	0.13	
K11216	autoinducer-2 kinase [EC:2.7.1.189]	0.14	5.6E-31	9.2E-31	0.17	
K08969	L-glutamine---4-(methylsulfanyl)-2-oxobutanoate aminotransferase [EC:2.6.1.117]	0.14	6.1E-31	1.0E-30	0.14	
K07645	two-component system, OmpR family, sensor histidine kinase QseC [EC:2.7.13.3]	0.14	6.2E-31	1.0E-30	0.16	
K00875	D-ribulokinase [EC:2.7.1.47]	0.14	6.4E-31	1.0E-30	0.15	
K16363	UDP-3-O-[3-hydroxymyristoyl] N-acetylglucosamine deacetylase / 3-hydroxyacyl-[acyl-carrier-protein] dehydratase [EC:3.5.1.108 4.2.1.59]	0.14	6.7E-31	1.1E-30	0.14	
K07683	two-component system, NarL family, sensor histidine kinase NreB [EC:2.7.13.3]	0.14	8.2E-31	1.3E-30	0.20	

Predictor	Description	Pearson's r	P	FDR-adjusted P	Spearman's rho	Associated with fractures (P<0.05)
K08100	bilirubin oxidase [EC:1.3.3.5]	0.14	1.0E-30	1.6E-30	0.17	
K04073	acetaldehyde dehydrogenase [EC:1.2.1.10]	0.14	1.1E-30	1.8E-30	0.16	
K02298	cytochrome o ubiquinol oxidase subunit I [EC:7.1.1.3]	0.14	1.2E-30	2.0E-30	0.16	
K02297	cytochrome o ubiquinol oxidase subunit II [EC:7.1.1.3]	0.14	1.3E-30	2.1E-30	0.16	
K01903	succinyl-CoA synthetase beta subunit [EC:6.2.1.5]	0.14	1.4E-30	2.3E-30	0.13	
K00673	arginine N-succinyltransferase [EC:2.3.1.109]	0.14	1.4E-30	2.3E-30	0.16	
K03181	chorismate lyase [EC:4.1.3.40]	0.14	1.5E-30	2.4E-30	0.16	
K15467	27-O-demethylrifamycin SV methyltransferase [EC:2.1.1.315]	0.14	1.7E-30	2.8E-30	0.14	
K12253	5-guanidino-2-oxopentanoate decarboxylase [EC:4.1.1.75]	0.14	2.0E-30	3.3E-30	0.14	
K15740	tetrahydromethanopterin:alpha-L-glutamate ligase [EC:6.3.2.33]	0.14	2.1E-30	3.4E-30	0.15	
K01577	oxalyl-CoA decarboxylase [EC:4.1.1.8]	0.14	2.1E-30	3.4E-30	0.15	
K05591	ATP-dependent RNA helicase DbpA [EC:3.6.4.13]	0.14	2.6E-30	4.2E-30	0.16	
K03794	sirohydrochlorin ferrochelatase [EC:4.99.1.4]	0.14	2.7E-30	4.4E-30	0.22	yes
K16305	fructose-bisphosphate aldolase / 6-deoxy-5-ketofructose 1-phosphate synthase [EC:4.1.2.13 2.2.1.11]	0.14	3.1E-30	5.0E-30	0.20	
K10824	nickel transport system ATP-binding protein [EC:7.2.2.11]	0.14	3.1E-30	5.0E-30	0.16	
K04128	hydroxymethyl cephem carbamoyltransferase [EC:2.1.3.7]	0.14	3.2E-30	5.2E-30	0.18	
K03777	D-lactate dehydrogenase (quinone) [EC:1.1.5.12]	0.14	3.6E-30	5.8E-30	0.16	
K12140	hydrogenase-4 component E [EC:1.-.-.-]	0.13	5.3E-30	8.6E-30	0.14	
K01643	citrate lyase subunit alpha / citrate CoA-transferase [EC:2.8.3.10]	0.13	7.5E-30	1.2E-29	0.15	
K10222	2,6-dioxo-6-phenylhexa-3-enoate hydrolase [EC:3.7.1.8]	0.13	7.8E-30	1.3E-29	0.15	
K08689	biphenyl 2,3-dioxygenase subunit alpha [EC:1.14.12.18]	0.13	7.8E-30	1.3E-29	0.15	
K00462	biphenyl-2,3-diol 1,2-dioxygenase [EC:1.13.11.39]	0.13	7.8E-30	1.3E-29	0.15	
K12141	hydrogenase-4 component F [EC:1.-.-.-]	0.13	8.7E-30	1.4E-29	0.14	
K03214	RNA methyltransferase, TrmH family [EC:2.1.1.-]	0.13	1.1E-29	1.8E-29	0.15	
K00942	guanylate kinase [EC:2.7.4.8]	0.13	1.2E-29	1.9E-29	0.18	
K01322	prolyl oligopeptidase [EC:3.4.21.26]	0.13	1.5E-29	2.4E-29	0.14	
K14728	phthiodiolone/phenolphthiodiolone dimycocerosates ketoreductase [EC:1.2.-.-]	0.13	1.7E-29	2.7E-29	0.17	
K00832	aromatic-amino-acid transaminase [EC:2.6.1.57]	0.13	2.1E-29	3.4E-29	0.15	
K16039	N-glycosyltransferase [EC:2.4.1.-]	0.13	2.4E-29	3.9E-29	0.18	yes
K01251	adenosylhomocysteinase [EC:3.3.1.1]	0.13	2.8E-29	4.5E-29	0.13	
K01139	GTP diphosphokinase / guanosine-3',5'-bis(diphosphate) 3'-diphosphatase [EC:2.7.6.5 3.1.7.2]	0.13	2.9E-29	4.7E-29	0.15	
K15736	(S)-2-hydroxyglutarate dehydrogenase [EC:1.1.5.13]	0.13	3.8E-29	6.1E-29	0.16	
K10111	multiple sugar transport system ATP-binding protein [EC:7.5.2.-]	0.13	4.5E-29	7.2E-29	0.16	
K01159	crossover junction endodeoxyribonuclease RuvC [EC:3.1.21.10]	0.13	5.0E-29	8.0E-29	0.15	
K00484	flavin reductase (NADH) [EC:1.5.1.36]	0.13	5.1E-29	8.2E-29	0.16	
K00255	long-chain-acyl-CoA dehydrogenase [EC:1.3.8.8]	0.13	5.1E-29	8.2E-29	0.17	
K05989	alpha-L-rhamnosidase [EC:3.2.1.40]	-0.13	5.7E-29	9.1E-29	-0.24	
K01347	IgA-specific serine endopeptidase [EC:3.4.21.72]	0.13	6.2E-29	9.9E-29	0.15	
K03774	FKBP-type peptidyl-prolyl cis-trans isomerase SlpA [EC:5.2.1.8]	0.13	6.3E-29	1.0E-28	0.16	
K15751	carbazole 1,9a-dioxygenase [EC:1.14.12.22]	0.13	6.5E-29	1.0E-28	0.17	
K13604	bacteriochlorophyllide d C-20 methyltransferase [EC:2.1.1.333]	0.13	7.8E-29	1.2E-28	0.19	
K01725	cyanate lyase [EC:4.2.1.104]	0.13	8.1E-29	1.3E-28	0.16	

Predictor	Description	Pearson's r	P	FDR-adjusted P	Spearman's rho	Associated with fractures (P<0.05)
K01274	beta-Ala-Xaa dipeptidase [EC:3.4.13.-]	0.13	9.7E-29	1.6E-28	0.14	
K01815	4-deoxy-L-threo-5-hexosulose-uronate ketol-isomerase [EC:5.3.1.17]	-0.13	1.0E-28	1.6E-28	-0.18	
K14187	chorismate mutase / prephenate dehydrogenase [EC:5.4.99.5 1.3.1.12]	0.13	1.1E-28	1.8E-28	0.14	
K15763	toluene monooxygenase system protein D [EC:1.14.13.236 1.14.13.-]	0.13	1.2E-28	1.9E-28	0.18	yes
K01470	creatinine amidohydrolase [EC:3.5.2.10]	-0.13	1.3E-28	2.1E-28	-0.24	
K00090	glyoxylate/hydroxypyruvate/2-ketogluconate reductase [EC:1.1.1.79 1.1.1.81 1.1.1.215]	0.13	1.4E-28	2.2E-28	0.16	
K10535	hydroxylamine dehydrogenase [EC:1.7.2.6]	0.13	1.5E-28	2.4E-28	0.18	
K00848	rhamnulokinase [EC:2.7.1.5]	-0.13	1.6E-28	2.5E-28	-0.23	
K03779	L(+)-tartrate dehydratase alpha subunit [EC:4.2.1.32]	0.13	1.9E-28	3.0E-28	0.14	
K13028	aldoxime dehydratase [EC:4.99.1.5]	0.13	1.9E-28	3.0E-28	0.19	
K08070	2-alkenal reductase [EC:1.3.1.74]	0.13	2.0E-28	3.2E-28	0.15	
K13524	4-aminobutyrate aminotransferase / (S)-3-amino-2-methylpropionate transaminase [EC:2.6.1.19 2.6.1.22]	0.13	2.1E-28	3.3E-28	0.15	
K03817	ribosomal-protein-serine acetyltransferase [EC:2.3.1.-]	0.13	2.2E-28	3.5E-28	0.13	
K06132	cardiolipin synthase C [EC:2.7.8.-]	0.13	2.4E-28	3.8E-28	0.15	
K02013	iron complex transport system ATP-binding protein [EC:7.2.2.-]	0.13	2.6E-28	4.1E-28	0.17	
K03800	lipoate---protein ligase [EC:6.3.1.20]	0.13	2.6E-28	4.1E-28	0.13	
K14260	alanine-synthesizing transaminase [EC:2.6.1.66 2.6.1.2]	0.13	2.7E-28	4.3E-28	0.13	
K15373	sulfoacetaldehyde reductase [EC:1.1.1.313]	0.13	2.7E-28	4.3E-28	0.20	
K06445	acyl-CoA dehydrogenase [EC:1.3.99.-]	0.13	3.1E-28	4.9E-28	0.16	
K01354	oligopeptidase B [EC:3.4.21.83]	0.13	3.2E-28	5.1E-28	0.14	
K00432	glutathione peroxidase [EC:1.11.1.9]	-0.13	3.4E-28	5.4E-28	-0.23	
K05541	tRNA-dihydrouridine synthase C [EC:1.-.-.-]	0.13	3.7E-28	5.9E-28	0.16	
K10681	two-component system, OmpR family, sensor histidine kinase SaeS [EC:2.7.13.3]	0.13	3.8E-28	6.0E-28	0.14	
K01424	L-asparaginase [EC:3.5.1.1]	-0.13	4.2E-28	6.6E-28	-0.21	
K08728	nucleoside deoxyribosyltransferase [EC:2.4.2.6]	0.13	5.0E-28	7.9E-28	0.14	
K15059	2-aminophenol/2-amino-5-chlorophenol 1,6-dioxygenase subunit beta [EC:1.13.11.74 1.13.11.76]	0.13	5.9E-28	9.3E-28	0.14	
K15253	chlorocatechol 1,2-dioxygenase [EC:1.13.11.-]	0.13	5.9E-28	9.3E-28	0.14	
K01860	chloromuconate cycloisomerase [EC:5.5.1.7]	0.13	5.9E-28	9.3E-28	0.14	
K15396	tRNA (cytidine32/uridine32-2'-O)-methyltransferase [EC:2.1.1.200]	0.13	8.3E-28	1.3E-27	0.15	
K00604	methionyl-tRNA formyltransferase [EC:2.1.2.9]	0.13	8.7E-28	1.4E-27	0.18	
K15460	tRNA1Val (adenine37-N6)-methyltransferase [EC:2.1.1.223]	-0.13	9.1E-28	1.4E-27	-0.21	
K08475	two-component system, NtrC family, phosphoglycerate transport system sensor histidine kinase PgtB [EC:2.7.13.3]	0.13	9.4E-28	1.5E-27	0.17	
K00455	3,4-dihydroxyphenylacetate 2,3-dioxygenase [EC:1.13.11.15]	0.13	1.0E-27	1.6E-27	0.16	
K00950	2-amino-4-hydroxy-6-hydroxymethyldihydropteridine diphosphokinase [EC:2.7.6.3]	-0.13	1.3E-27	2.0E-27	-0.25	
K08302	tagatose 1,6-diphosphate aldolase GatY/KbaY [EC:4.1.2.40]	0.13	1.3E-27	2.0E-27	0.14	
K16370	6-phosphofructokinase 2 [EC:2.7.1.11]	0.13	1.3E-27	2.0E-27	0.15	
K02614	acyl-CoA thioesterase [EC:3.1.2.-]	-0.13	1.4E-27	2.2E-27	-0.23	
K01784	UDP-glucose 4-epimerase [EC:5.1.3.2]	0.13	1.7E-27	2.7E-27	0.18	
K03186	flavin prenyltransferase [EC:2.5.1.129]	0.13	1.9E-27	3.0E-27	0.13	
K01515	ADP-ribose pyrophosphatase [EC:3.6.1.13]	0.13	2.3E-27	3.6E-27	0.13	
K03897	lysine N6-hydroxylase [EC:1.14.13.59]	0.13	2.4E-27	3.8E-27	0.16	
K00790	UDP-N-acetylglucosamine 1-carboxyvinyltransferase [EC:2.5.1.7]	0.13	2.5E-27	3.9E-27	0.20	

Predictor	Description	Pearson's r	P	FDR-adjusted P	Spearman's rho	Associated with fractures (P<0.05)
K00067	dTDP-4-dehydrorhamnose reductase [EC:1.1.1.133]	-0.13	3.0E-27	4.7E-27	-0.20	
K11065	thioredoxin-dependent peroxiredoxin [EC:1.11.1.24]	-0.13	3.3E-27	5.2E-27	-0.21	
K02337	DNA polymerase III subunit alpha [EC:2.7.7.7]	0.13	3.4E-27	5.3E-27	0.18	
K16303	p-cumate 2,3-dioxygenase subunit beta [EC:1.14.12.25]	0.13	4.5E-27	7.0E-27	0.17	
K01772	protoporphyrin/coproporphyrin ferrochelatase [EC:4.99.1.1 4.99.1.9]	0.13	5.2E-27	8.1E-27	0.14	
K04041	fructose-1,6-bisphosphatase III [EC:3.1.3.11]	-0.13	7.1E-27	1.1E-26	-0.25	
K00906	isocitrate dehydrogenase kinase/phosphatase [EC:2.7.11.5 3.1.3.-]	0.13	7.3E-27	1.1E-26	0.15	
K03430	2-aminoethylphosphonate-pyruvate transaminase [EC:2.6.1.37]	-0.13	7.4E-27	1.2E-26	-0.24	
K00024	malate dehydrogenase [EC:1.1.1.37]	-0.13	7.9E-27	1.2E-26	-0.23	
K00529	3-phenylpropionate/trans-cinnamate dioxygenase ferredoxin reductase component [EC:1.18.1.3]	0.13	8.1E-27	1.3E-26	0.15	
K01070	S-formylglutathione hydrolase [EC:3.1.2.12]	0.13	9.5E-27	1.5E-26	0.15	
K01878	glycyl-tRNA synthetase alpha chain [EC:6.1.1.14]	0.13	9.9E-27	1.5E-26	0.13	
K01929	UDP-N-acetylmuramoyl-tripeptide--D-alanyl-D-alanine ligase [EC:6.3.2.10]	0.13	1.0E-26	1.6E-26	0.18	
K06221	2,5-diketo-D-gluconate reductase A [EC:1.1.1.346]	0.13	1.2E-26	1.9E-26	0.15	
K01904	4-coumarate--CoA ligase [EC:6.2.1.12]	0.13	1.3E-26	2.0E-26	0.14	yes
K10774	tyrosine ammonia-lyase [EC:4.3.1.23]	0.13	1.3E-26	2.0E-26	0.14	yes
K16157	methane monooxygenase component A alpha chain [EC:1.14.13.25]	0.13	1.5E-26	2.3E-26	0.17	
K16158	methane monooxygenase component A beta chain [EC:1.14.13.25]	0.13	1.5E-26	2.3E-26	0.17	
K08312	ADP-ribose diphosphatase [EC:3.6.1.-]	0.13	1.7E-26	2.6E-26	0.15	
K13038	phosphopantothenoylcysteine decarboxylase / phosphopantothenate---cysteine ligase [EC:4.1.1.36 6.3.2.5]	0.13	1.8E-26	2.8E-26	0.13	
K00897	kanamycin kinase [EC:2.7.1.95]	0.13	1.8E-26	2.8E-26	0.14	
K00340	NADH-quinone oxidoreductase subunit K [EC:7.1.1.2]	-0.13	1.9E-26	2.9E-26	-0.23	
K10619	p-cumate 2,3-dioxygenase subunit alpha [EC:1.14.12.25]	0.13	2.1E-26	3.2E-26	0.17	
K08321	3-hydroxy-5-phosphonooxypentane-2,4-dione thiolase [EC:2.3.1.245]	0.13	2.7E-26	4.2E-26	0.15	
K07711	two-component system, NtrC family, sensor histidine kinase GlrK [EC:2.7.13.3]	0.13	3.7E-26	5.7E-26	0.15	
K01687	dihydroxy-acid dehydratase [EC:4.2.1.9]	0.13	3.8E-26	5.9E-26	0.19	
K03206	azobenzene reductase [EC:1.7.1.6]	0.13	3.9E-26	6.0E-26	0.19	
K01252	bifunctional isochorismate lyase / aryl carrier protein [EC:3.3.2.1 6.3.2.14]	0.13	4.0E-26	6.2E-26	0.15	
K03082	NA	0.13	4.1E-26	6.3E-26	0.15	
K04781	salicylate synthetase [EC:5.4.4.2 4.2.99.21]	0.13	5.3E-26	8.2E-26	0.14	
K05851	adenylate cyclase, class 1 [EC:4.6.1.1]	0.13	5.4E-26	8.3E-26	0.15	
K08566	plasminogen activator [EC:3.4.23.48]	0.13	6.1E-26	9.4E-26	0.17	
K01448	N-acetylmuramoyl-L-alanine amidase [EC:3.5.1.28]	-0.13	6.3E-26	9.7E-26	-0.23	
K07710	two-component system, NtrC family, sensor histidine kinase AtoS [EC:2.7.13.3]	0.12	6.4E-26	9.8E-26	0.14	
K01206	alpha-L-fucosidase [EC:3.2.1.51]	-0.12	6.9E-26	1.1E-25	-0.26	
K00971	mannose-1-phosphate guanylyltransferase [EC:2.7.7.13]	-0.12	8.1E-26	1.2E-25	-0.21	
K03781	catalase [EC:1.11.1.6]	0.12	9.2E-26	1.4E-25	0.12	
K09472	4-(gamma-glutamylamino)butanal dehydrogenase [EC:1.2.1.99]	0.12	1.0E-25	1.5E-25	0.14	
K03768	peptidyl-prolyl cis-trans isomerase B (cyclophilin B) [EC:5.2.1.8]	0.12	1.1E-25	1.7E-25	0.20	
K01879	glycyl-tRNA synthetase beta chain [EC:6.1.1.14]	0.12	1.1E-25	1.7E-25	0.13	
K01607	4-carboxymuconolactone decarboxylase [EC:4.1.1.44]	0.12	1.2E-25	1.8E-25	0.12	
K00073	ureidoglycolate dehydrogenase (NAD+) [EC:1.1.1.350]	0.12	1.2E-25	1.8E-25	0.14	

Predictor	Description	Pearson's r	P	FDR-adjusted P	Spearman's rho	Associated with fractures (P<0.05)
K01706	glucarate dehydratase [EC:4.2.1.40]	0.12	1.6E-25	2.4E-25	0.15	
K11072	spermidine/putrescine transport system ATP-binding protein [EC:7.6.2.11]	-0.12	1.8E-25	2.8E-25	-0.23	
K14587	protein sgcE [EC:5.1.3.-]	0.12	1.8E-25	2.8E-25	0.15	
K14159	ribonuclease HI / DNA polymerase III subunit epsilon [EC:3.1.26.4 2.7.7.7]	0.12	1.9E-25	2.9E-25	0.12	
K01778	diaminopimelate epimerase [EC:5.1.1.7]	0.12	2.2E-25	3.4E-25	0.17	
K00098	L-idonate 5-dehydrogenase [EC:1.1.1.264]	0.12	2.2E-25	3.4E-25	0.14	
K16066	3-hydroxy acid dehydrogenase / malonic semialdehyde reductase [EC:1.1.1.381 1.1.1.-]	0.12	2.2E-25	3.4E-25	0.14	
K00813	aspartate aminotransferase [EC:2.6.1.1]	0.12	2.3E-25	3.5E-25	0.14	
K01791	UDP-N-acetylglucosamine 2-epimerase (non-hydrolysing) [EC:5.1.3.14]	-0.12	2.3E-25	3.5E-25	-0.24	
K13074	biflaviolin synthase [EC:1.14.19.69]	0.12	2.7E-25	4.1E-25	0.17	
K08319	L-threonate 2-dehydrogenase [EC:1.1.1.411]	0.12	2.8E-25	4.3E-25	0.15	
K01910	[citrate (pro-3S)-lyase] ligase [EC:6.2.1.22]	0.12	2.9E-25	4.4E-25	0.14	
K01923	phosphoribosylaminoimidazole-succinocarboxamide synthase [EC:6.3.2.6]	0.12	3.2E-25	4.9E-25	0.18	
K01807	ribose 5-phosphate isomerase A [EC:5.3.1.6]	0.12	3.4E-25	5.2E-25	0.12	
K13689	beta-1,4-N-acetylgalactosaminyltransferase [EC:2.4.1.-]	0.12	4.3E-25	6.5E-25	0.13	
K09828	Delta24-sterol reductase [EC:1.3.1.72 1.3.1.-]	0.12	4.4E-25	6.7E-25	0.13	
K05774	ribose 1,5-bisphosphokinase [EC:2.7.4.23]	0.12	4.6E-25	7.0E-25	0.14	
K12111	evolved beta-galactosidase subunit alpha [EC:3.2.1.23]	0.12	4.9E-25	7.4E-25	0.14	
K10915	CAI-1 autoinducer synthase [EC:2.3.-.-]	0.12	5.6E-25	8.5E-25	0.15	
K12248	beta-galactoside alpha-2,6-sialyltransferase (sialyltransferase 0160) [EC:2.4.99.1]	0.12	6.1E-25	9.2E-25	0.11	
K07673	two-component system, NarL family, nitrate/nitrite sensor histidine kinase NarX [EC:2.7.13.3]	0.12	7.4E-25	1.1E-24	0.14	
K16159	methane monooxygenase component A gamma chain [EC:1.14.13.25]	0.12	7.9E-25	1.2E-24	0.16	
K16161	methane monooxygenase component C [EC:1.14.13.25]	0.12	7.9E-25	1.2E-24	0.16	
K01582	lysine decarboxylase [EC:4.1.1.18]	0.12	8.3E-25	1.3E-24	0.13	
K08306	membrane-bound lytic murein transglycosylase C [EC:4.2.2.-]	0.12	8.7E-25	1.3E-24	0.14	
K01919	glutamate--cysteine ligase [EC:6.3.2.2]	0.12	8.8E-25	1.3E-24	0.12	
K02000	glycine betaine/proline transport system ATP-binding protein [EC:7.6.2.9]	-0.12	9.8E-25	1.5E-24	-0.26	
K15020	acryloyl-coenzyme A reductase [EC:1.3.1.84]	0.12	1.1E-24	1.7E-24	0.11	
K00343	NADH-quinone oxidoreductase subunit N [EC:7.1.1.2]	0.12	1.2E-24	1.8E-24	0.12	
K07675	two-component system, NarL family, sensor histidine kinase UhpB [EC:2.7.13.3]	0.12	1.2E-24	1.8E-24	0.14	
K01912	phenylacetate-CoA ligase [EC:6.2.1.30]	-0.12	1.4E-24	2.1E-24	-0.21	
K05972	acetylxylan esterase [EC:3.1.1.72]	0.12	1.4E-24	2.1E-24	0.12	
K15972	tetracenomycin A2 monooxygenase-dioxygenase [EC:1.14.13.200]	0.12	1.4E-24	2.1E-24	0.12	
K01218	mannan endo-1,4-beta-mannosidase [EC:3.2.1.78]	0.12	1.5E-24	2.3E-24	0.12	
K01484	succinylarginine dihydrolase [EC:3.5.3.23]	0.12	1.5E-24	2.3E-24	0.14	
K00068	sorbitol-6-phosphate 2-dehydrogenase [EC:1.1.1.140]	0.12	1.5E-24	2.3E-24	0.13	
K01190	beta-galactosidase [EC:3.2.1.23]	-0.12	1.5E-24	2.3E-24	-0.24	
K10539	L-arabinose transport system ATP-binding protein [EC:7.5.2.12]	0.12	1.8E-24	2.7E-24	0.14	
K07812	trimethylamine-N-oxide reductase (cytochrome c) [EC:1.7.2.3]	0.12	1.9E-24	2.9E-24	0.13	
K00471	gamma-butyrobetaine dioxygenase [EC:1.14.11.1]	0.12	1.9E-24	2.9E-24	0.19	
K13308	dTDP-4-amino-4,6-dideoxy-D-glucose transaminase [EC:2.6.1.33]	0.12	2.1E-24	3.1E-24	0.15	
K03654	ATP-dependent DNA helicase RecQ [EC:3.6.4.12]	-0.12	2.1E-24	3.1E-24	-0.23	

Predictor	Description	Pearson's r	P	FDR-adjusted P	Spearman's rho	Associated with fractures (P<0.05)
K06021	NA	0.12	2.7E-24	4.0E-24	0.13	
K11066	N-acetylmuramoyl-L-alanine amidase [EC:3.5.1.28]	0.12	2.8E-24	4.2E-24	0.14	
K00622	arylamine N-acetyltransferase [EC:2.3.1.5]	0.12	3.1E-24	4.6E-24	0.15	
K01625	2-dehydro-3-deoxyphosphogluconate aldolase / (4S)-4-hydroxy-2-oxoglutarate aldolase [EC:4.1.2.14 4.1.3.42]	-0.12	6.9E-24	1.0E-23	-0.23	
K12988	alpha-1,3-rhamnosyltransferase [EC:2.4.1.-]	0.12	7.8E-24	1.2E-23	0.11	
K15765	toluene monooxygenase electron transfer component [EC:1.18.1.3]	0.12	8.1E-24	1.2E-23	0.19	
K12447	UDP-sugar pyrophosphorylase [EC:2.7.7.64]	0.12	9.9E-24	1.5E-23	0.11	
K02336	DNA polymerase II [EC:2.7.7.7]	0.12	1.2E-23	1.8E-23	0.14	
K02439	thiosulfate sulfurtransferase [EC:2.8.1.1]	0.12	1.2E-23	1.8E-23	0.14	
K01766	cysteine sulfinate desulfinase [EC:4.4.1.-]	0.12	1.5E-23	2.2E-23	0.13	
K03602	exodeoxyribonuclease VII small subunit [EC:3.1.11.6]	0.12	1.8E-23	2.7E-23	0.19	
K00802	spermine synthase [EC:2.5.1.22]	0.12	1.8E-23	2.7E-23	0.17	
K16178	dimethylamine---corrinoid protein Co-methyltransferase [EC:2.1.1.249]	0.12	1.8E-23	2.7E-23	0.13	
K00463	indoleamine 2,3-dioxygenase [EC:1.13.11.52]	0.12	2.0E-23	3.0E-23	0.15	
K01673	carbonic anhydrase [EC:4.2.1.1]	0.12	2.3E-23	3.4E-23	0.11	
K01631	2-dehydro-3-deoxyphosphogalactonate aldolase [EC:4.1.2.21]	0.12	2.8E-23	4.2E-23	0.14	
K00796	dihydropteroate synthase [EC:2.5.1.15]	-0.12	2.9E-23	4.3E-23	-0.20	
K06447	succinylglutamic semialdehyde dehydrogenase [EC:1.2.1.71]	0.12	3.6E-23	5.3E-23	0.14	
K09011	(R)-citramalate synthase [EC:2.3.1.182]	-0.12	3.6E-23	5.3E-23	-0.25	
K05362	UDP-N-acetylmuramoyl-L-alanyl-D-glutamate-L-lysine ligase [EC:6.3.2.7]	0.12	4.3E-23	6.4E-23	0.12	
K06607	myo-inositol catabolism protein IolS [EC:1.1.1.-]	0.12	4.9E-23	7.3E-23	0.15	yes
K06222	2,5-diketo-D-gluconate reductase B [EC:1.1.1.346]	0.12	5.8E-23	8.6E-23	0.14	
K01572	NA	-0.12	5.8E-23	8.6E-23	-0.23	
K01961	acetyl-CoA carboxylase, biotin carboxylase subunit [EC:6.4.1.2 6.3.4.14]	-0.12	6.5E-23	9.6E-23	-0.23	
K06177	tRNA pseudouridine32 synthase / 23S rRNA pseudouridine746 synthase [EC:5.4.99.28 5.4.99.29]	-0.12	6.5E-23	9.6E-23	-0.25	
K13500	chondroitin synthase [EC:2.4.1.175 2.4.1.226]	0.12	6.9E-23	1.0E-22	0.15	yes
K10215	monooxygenase [EC:1.14.13.-]	0.12	7.7E-23	1.1E-22	0.19	yes
K07637	two-component system, OmpR family, sensor histidine kinase PhoQ [EC:2.7.13.3]	0.12	8.4E-23	1.2E-22	0.14	
K00880	L-xylulokinase [EC:2.7.1.53]	0.12	8.6E-23	1.3E-22	0.12	
K03079	L-ribulose-5-phosphate 3-epimerase [EC:5.1.3.22]	0.12	8.8E-23	1.3E-22	0.12	
K08687	N-carbamoylsarcosine amidase [EC:3.5.1.59]	0.12	9.1E-23	1.3E-22	0.14	yes
K01082	3'(2'), 5'-bisphosphate nucleotidase [EC:3.1.3.7]	0.12	9.6E-23	1.4E-22	0.12	
K13501	anthranilate synthase / indole-3-glycerol phosphate synthase / phosphoribosylanthranilate isomerase [EC:4.1.3.27 4.1.1.48 5.3.1.24]	0.12	1.1E-22	1.6E-22	0.12	
K03829	putative acetyltransferase [EC:2.3.1.-]	0.12	1.3E-22	1.9E-22	0.14	
K10984	galactosamine PTS system EIIB component [EC:2.7.1.-]	0.12	1.4E-22	2.1E-22	0.13	
K03787	5'-nucleotidase [EC:3.1.3.5]	-0.12	1.4E-22	2.1E-22	-0.23	
K01399	pseudolysin [EC:3.4.24.26]	0.12	1.4E-22	2.1E-22	0.18	
K09761	16S rRNA (uracil1498-N3)-methyltransferase [EC:2.1.1.193]	0.12	1.4E-22	2.1E-22	0.16	
K05304	sialic acid synthase [EC:2.5.1.56 2.5.1.57 2.5.1.132]	0.12	1.5E-22	2.2E-22	0.15	
K03081	NA	0.12	1.6E-22	2.4E-22	0.13	
K11392	16S rRNA (cytosine1407-C5)-methyltransferase [EC:2.1.1.178]	0.12	1.7E-22	2.5E-22	0.13	
K07106	N-acetylmuramic acid 6-phosphate etherase [EC:4.2.1.126]	-0.12	1.8E-22	2.6E-22	-0.21	

Predictor	Description	Pearson's r	P	FDR-adjusted P	Spearman's rho	Associated with fractures (P<0.05)
K00030	isocitrate dehydrogenase (NAD+) [EC:1.1.1.41]	0.12	1.9E-22	2.8E-22	0.14	
K13062	acyl homoserine lactone synthase [EC:2.3.1.184]	0.12	1.9E-22	2.8E-22	0.12	
K11717	cysteine desulfurase / selenocysteine lyase [EC:2.8.1.7 4.4.1.16]	0.12	2.0E-22	2.9E-22	0.17	
K07677	two-component system, NarL family, capsular synthesis sensor histidine kinase RcsC [EC:2.7.13.3]	0.12	2.2E-22	3.2E-22	0.13	
K01825	3-hydroxyacyl-CoA dehydrogenase / enoyl-CoA hydratase / 3-hydroxybutyryl-CoA epimerase / enoyl-CoA isomerase [EC:1.1.1.35 4.2.1.17 5.1.2.3 5.3.3.8]	0.12	2.4E-22	3.5E-22	0.13	
K14596	zeaxanthin glucosyltransferase [EC:2.4.1.276]	0.12	2.5E-22	3.7E-22	0.20	
K03814	monofunctional glycosyltransferase [EC:2.4.1.129]	-0.12	2.6E-22	3.8E-22	-0.25	
K00788	thiamine-phosphate pyrophosphorylase [EC:2.5.1.3]	-0.12	2.6E-22	3.8E-22	-0.23	
K00011	aldehyde reductase [EC:1.1.1.21]	0.12	2.7E-22	3.9E-22	0.15	yes
K00256	NA	0.12	2.7E-22	3.9E-22	0.15	yes
K00895	diphosphate-dependent phosphofructokinase [EC:2.7.1.90]	-0.12	2.9E-22	4.2E-22	-0.24	
K00883	2-dehydro-3-deoxygalactonokinase [EC:2.7.1.58]	0.12	3.5E-22	5.1E-22	0.13	
K14333	2,3-dihydroxybenzoate decarboxylase [EC:4.1.1.46]	0.11	3.7E-22	5.4E-22	0.15	
K03657	DNA helicase II / ATP-dependent DNA helicase PcrA [EC:3.6.4.12]	0.11	4.2E-22	6.1E-22	0.17	
K03278	UDP-D-galactose:(glucosyl)LPS alpha-1,3-D-galactosyltransferase [EC:2.4.1.44]	0.11	4.3E-22	6.3E-22	0.14	
K01709	CDP-glucose 4,6-dehydratase [EC:4.2.1.45]	-0.11	4.7E-22	6.8E-22	-0.23	
K07639	two-component system, OmpR family, sensor histidine kinase RstB [EC:2.7.13.3]	0.11	4.7E-22	6.8E-22	0.13	
K06957	tRNA(Met) cytidine acetyltransferase [EC:2.3.1.193]	0.11	5.8E-22	8.4E-22	0.13	
K00978	glucose-1-phosphate cytidylyltransferase [EC:2.7.7.33]	-0.11	6.3E-22	9.2E-22	-0.23	
K03822	putative long chain acyl-CoA synthase [EC:6.2.1.-]	0.11	6.4E-22	9.3E-22	0.14	
K03772	FKBP-type peptidyl-prolyl cis-trans isomerase FkpA [EC:5.2.1.8]	0.11	7.6E-22	1.1E-21	0.12	
K00657	diamine N-acetyltransferase [EC:2.3.1.57]	-0.11	8.2E-22	1.2E-21	-0.20	
K12524	bifunctional aspartokinase / homoserine dehydrogenase 1 [EC:2.7.2.4 1.1.1.3]	-0.11	8.3E-22	1.2E-21	-0.25	
K00981	phosphatidate cytidylyltransferase [EC:2.7.7.41]	-0.11	9.7E-22	1.4E-21	-0.16	
K03526	(E)-4-hydroxy-3-methylbut-2-enyl-diphosphate synthase [EC:1.17.7.1 1.17.7.3]	0.11	1.2E-21	1.7E-21	0.18	
K03472	D-erythrose 4-phosphate dehydrogenase [EC:1.2.1.72]	0.11	1.3E-21	1.9E-21	0.13	
K00425	cytochrome bd ubiquinol oxidase subunit I [EC:7.1.1.7]	-0.11	1.5E-21	2.2E-21	-0.19	yes
K16212	4-O-beta-D-mannosyl-D-glucose phosphorylase [EC:2.4.1.281]	-0.11	1.6E-21	2.3E-21	-0.23	
K15019	3-hydroxypropionyl-coenzyme A dehydratase [EC:4.2.1.116]	0.11	1.8E-21	2.6E-21	0.11	
K06441	ferredoxin hydrogenase gamma subunit [EC:1.12.7.2]	0.11	1.9E-21	2.8E-21	0.17	
K01150	deoxyribonuclease I [EC:3.1.21.1]	0.11	2.1E-21	3.0E-21	0.13	
K01599	uroporphyrinogen decarboxylase [EC:4.1.1.37]	-0.11	2.2E-21	3.2E-21	-0.23	
K03078	3-dehydro-L-gulonate-6-phosphate decarboxylase [EC:4.1.1.85]	0.11	2.4E-21	3.5E-21	0.12	
K10027	phytoene desaturase [EC:1.3.99.26 1.3.99.28 1.3.99.29 1.3.99.31]	0.11	2.7E-21	3.9E-21	0.15	
K05526	succinylglutamate desuccinylase [EC:3.5.1.96]	0.11	2.9E-21	4.2E-21	0.13	
K03438	16S rRNA (cytosine1402-N4)-methyltransferase [EC:2.1.1.199]	0.11	2.9E-21	4.2E-21	0.18	yes
K00598	trans-aconitate 2-methyltransferase [EC:2.1.1.144]	0.11	3.1E-21	4.5E-21	0.12	
K03184	3-demethoxyubiquinol 3-hydroxylase [EC:1.14.99.60]	0.11	3.2E-21	4.6E-21	0.13	
K11258	acetolactate synthase II small subunit [EC:2.2.1.6]	0.11	3.3E-21	4.8E-21	0.13	
K07640	two-component system, OmpR family, sensor histidine kinase CpxA [EC:2.7.13.3]	0.11	3.6E-21	5.2E-21	0.13	
K02560	lauroyl-Kdo2-lipid IVA myristoyltransferase [EC:2.3.1.243]	0.11	3.7E-21	5.3E-21	0.13	
K01467	beta-lactamase class C [EC:3.5.2.6]	0.11	3.7E-21	5.3E-21	0.11	

Predictor	Description	Pearson's r	P	FDR-adjusted P	Spearman's rho	Associated with fractures (P<0.05)
K01893	asparaginyl-tRNA synthetase [EC:6.1.1.22]	-0.11	4.1E-21	5.9E-21	-0.16	
K00058	D-3-phosphoglycerate dehydrogenase / 2-oxoglutarate reductase [EC:1.1.1.95 1.1.1.399]	0.11	4.6E-21	6.6E-21	0.15	
K01271	Xaa-Pro dipeptidase [EC:3.4.13.9]	0.11	4.8E-21	6.9E-21	0.12	
K08317	hydroxycarboxylate dehydrogenase A [EC:1.1.1.-]	0.11	4.9E-21	7.0E-21	0.12	
K01194	alpha,alpha-trehalase [EC:3.2.1.28]	0.11	5.3E-21	7.6E-21	0.13	
K00940	nucleoside-diphosphate kinase [EC:2.7.4.6]	-0.11	8.1E-21	1.2E-20	-0.20	
K03212	23S rRNA (uracil747-C5)-methyltransferase [EC:2.1.1.189]	0.11	8.4E-21	1.2E-20	0.13	
K08261	D-sorbitol dehydrogenase (acceptor) [EC:1.1.99.21]	0.11	9.1E-21	1.3E-20	0.15	
K07444	putative N6-adenine-specific DNA methylase [EC:2.1.1.-]	-0.11	9.4E-21	1.3E-20	-0.22	
K03473	erythronate-4-phosphate dehydrogenase [EC:1.1.1.290]	-0.11	9.8E-21	1.4E-20	-0.21	
K00748	lipid-A-disaccharide synthase [EC:2.4.1.182]	-0.11	1.1E-20	1.6E-20	-0.23	
K00426	cytochrome bd ubiquinol oxidase subunit II [EC:7.1.1.7]	-0.11	1.1E-20	1.6E-20	-0.19	yes
K01277	dipeptidyl-peptidase III [EC:3.4.14.4]	0.11	1.2E-20	1.7E-20	0.12	
K08264	heterodisulfide reductase subunit D [EC:1.8.98.1]	0.11	1.2E-20	1.7E-20	0.12	
K01880	glycyl-tRNA synthetase [EC:6.1.1.14]	0.11	1.3E-20	1.9E-20	0.22	yes
K02036	phosphate transport system ATP-binding protein [EC:7.3.2.1]	0.11	1.3E-20	1.9E-20	0.18	
K02438	glycogen debranching enzyme [EC:3.2.1.196]	0.11	1.6E-20	2.3E-20	0.13	
K10830	manganese/zinc transport system ATP-binding protein [EC:7.2.2.5]	0.11	1.7E-20	2.4E-20	0.12	
K00998	CDP-diacylglycerol---serine O-phosphatidyltransferase [EC:2.7.8.8]	0.11	1.8E-20	2.6E-20	0.12	
K00467	lactate 2-monooxygenase [EC:1.13.12.4]	0.11	2.5E-20	3.6E-20	0.14	
K07589	D-erythro-7,8-dihydroneopterin triphosphate epimerase [EC:5.1.99.7]	0.11	2.7E-20	3.8E-20	0.12	
K04084	thioredoxin:protein disulfide reductase [EC:1.8.4.16]	-0.11	2.8E-20	4.0E-20	-0.23	
K03395	aminoglycoside 3-N-acetyltransferase I [EC:2.3.1.60]	0.11	2.8E-20	4.0E-20	0.17	
K01284	peptidyl-dipeptidase Dcp [EC:3.4.15.5]	-0.11	2.9E-20	4.1E-20	-0.21	
K12500	thioesterase III [EC:3.1.2.-]	0.11	3.0E-20	4.3E-20	0.12	
K10530	L-lactate oxidase [EC:1.1.3.2]	0.11	3.3E-20	4.7E-20	0.15	
K09457	7-cyano-7-deazaguanine reductase [EC:1.7.1.13]	-0.11	3.5E-20	5.0E-20	-0.25	
K01546	potassium-transporting ATPase potassium-binding subunit	-0.11	3.8E-20	5.4E-20	-0.17	
K15376	gephyrin [EC:2.10.1.1 2.7.7.75]	0.11	4.0E-20	5.7E-20	0.12	
K00306	sarcosine oxidase / L-pipecolate oxidase [EC:1.5.3.1 1.5.3.7]	0.11	4.0E-20	5.7E-20	0.12	
K01438	acetylornithine deacetylase [EC:3.5.1.16]	-0.11	5.8E-20	8.2E-20	-0.22	
K01385	thermopsin [EC:3.4.23.42]	0.11	6.3E-20	8.9E-20	0.10	
K08641	zinc D-Ala-D-Ala dipeptidase [EC:3.4.13.22]	-0.11	6.4E-20	9.1E-20	-0.22	
K00789	S-adenosylmethionine synthetase [EC:2.5.1.6]	0.11	6.9E-20	9.8E-20	0.15	
K05714	2-hydroxy-6-oxonona-2,4-dienedioate hydrolase [EC:3.7.1.14]	0.11	7.6E-20	1.1E-19	0.12	
K02517	Kdo2-lipid IVA lauroyltransferase/acyltransferase [EC:2.3.1.241 2.3.1.-]	-0.11	8.1E-20	1.1E-19	-0.20	
K04783	yersiniabactin salicyl-AMP ligase [EC:6.3.2.-]	0.11	9.0E-20	1.3E-19	0.12	
K07679	two-component system, NarL family, sensor histidine kinase EvgS [EC:2.7.13.3]	0.11	9.6E-20	1.4E-19	0.12	
K02615	3-oxo-5,6-didehydrosuberyl-CoA/3-oxoadipyl-CoA thiolase [EC:2.3.1.223 2.3.1.174]	0.11	1.0E-19	1.4E-19	0.13	
K07751	PepB aminopeptidase [EC:3.4.11.23]	0.11	1.1E-19	1.6E-19	0.12	
K03788	acid phosphatase (class B) [EC:3.1.3.2]	0.11	1.1E-19	1.6E-19	0.11	
K01521	CDP-diacylglycerol pyrophosphatase [EC:3.6.1.26]	0.11	1.1E-19	1.6E-19	0.12	

Predictor	Description	Pearson's r	P	FDR-adjusted P	Spearman's rho	Associated with fractures (P<0.05)
K16045	3beta-hydroxy-Delta5-steroid dehydrogenase / steroid Delta-isomerase [EC:1.1.1.145 5.3.3.1]	0.11	1.3E-19	1.8E-19	0.15	
K05590	ATP-dependent RNA helicase SrmB [EC:3.6.4.13]	0.11	1.4E-19	2.0E-19	0.12	
K08351	biotin/methionine sulfoxide reductase [EC:1.-.-.-]	0.11	1.8E-19	2.5E-19	0.12	
K01630	2-dehydro-3-deoxyglucarate aldolase [EC:4.1.2.20]	0.11	1.8E-19	2.5E-19	0.12	
K00603	glutamate formiminotransferase / 5-formyltetrahydrofolate cyclo-ligase [EC:2.1.2.5 6.3.3.2]	-0.11	2.2E-19	3.1E-19	-0.25	
K07026	mannosyl-3-phosphoglycerate phosphatase [EC:3.1.3.70]	0.11	2.2E-19	3.1E-19	0.12	
K10017	histidine transport system ATP-binding protein [EC:7.4.2.1]	0.11	2.2E-19	3.1E-19	0.12	
K02848	heptose I phosphotransferase [EC:2.7.1.-]	0.11	2.3E-19	3.2E-19	0.12	
K01117	sphingomyelin phosphodiesterase [EC:3.1.4.12]	0.11	2.3E-19	3.2E-19	0.11	
K09024	flavin reductase [EC:1.5.1.-]	0.11	2.3E-19	3.2E-19	0.12	
K01812	glucuronate isomerase [EC:5.3.1.12]	-0.11	2.3E-19	3.2E-19	-0.18	
K02799	NA	0.11	2.4E-19	3.4E-19	0.16	
K07107	acyl-CoA thioester hydrolase [EC:3.1.2.-]	-0.11	2.7E-19	3.8E-19	-0.18	
K07271	lipopolysaccharide cholinephosphotransferase [EC:2.7.8.-]	-0.11	2.8E-19	3.9E-19	-0.20	
K00226	dihydroorotate dehydrogenase (fumarate) [EC:1.3.98.1]	-0.11	3.0E-19	4.2E-19	-0.22	
K02548	1,4-dihydroxy-2-naphthoate polyprenyltransferase [EC:2.5.1.74]	-0.11	3.0E-19	4.2E-19	-0.18	
K01192	beta-mannosidase [EC:3.2.1.25]	-0.11	3.3E-19	4.6E-19	-0.24	
K10551	D-allose transport system ATP-binding protein [EC:7.5.2.8]	0.11	3.9E-19	5.5E-19	0.11	
K14659	chitooligosaccharide deacetylase [EC:3.5.1.-]	0.11	4.0E-19	5.6E-19	0.17	
K14666	N-acetylglucosaminyltransferase [EC:2.4.1.-]	0.11	4.0E-19	5.6E-19	0.17	
K01960	pyruvate carboxylase subunit B [EC:6.4.1.1]	-0.11	4.3E-19	6.0E-19	-0.24	
K08265	heterodisulfide reductase subunit E [EC:1.8.98.1]	0.11	4.4E-19	6.1E-19	0.11	
K08313	fructose-6-phosphate aldolase 1 [EC:4.1.2.-]	0.11	4.5E-19	6.3E-19	0.12	
K07568	S-adenosylmethionine:tRNA ribosyltransferase-isomerase [EC:2.4.99.17]	-0.11	4.5E-19	6.3E-19	-0.18	
K12556	penicillin-binding protein 2X	0.11	4.5E-19	6.3E-19	0.11	
K05713	2,3-dihydroxyphenylpropionate 1,2-dioxygenase [EC:1.13.11.16]	0.11	4.5E-19	6.3E-19	0.12	
K07636	two-component system, OmpR family, phosphate regulon sensor histidine kinase PhoR [EC:2.7.13.3]	0.11	4.7E-19	6.5E-19	0.19	
K03775	FKBP-type peptidyl-prolyl cis-trans isomerase SlyD [EC:5.2.1.8]	-0.11	4.8E-19	6.7E-19	-0.22	
K00606	3-methyl-2-oxobutanoate hydroxymethyltransferase [EC:2.1.2.11]	-0.11	5.4E-19	7.5E-19	-0.16	
K07255	taurine dehydrogenase small subunit [EC:1.4.2.-]	0.11	5.7E-19	7.9E-19	0.10	
K00842	NA	0.11	6.6E-19	9.2E-19	0.09	
K12957	alcohol/geraniol dehydrogenase (NADP+) [EC:1.1.1.2 1.1.1.183]	0.11	7.3E-19	1.0E-18	0.12	
K01085	glucose-1-phosphatase [EC:3.1.3.10]	0.11	7.5E-19	1.0E-18	0.12	
K08723	5'-nucleotidase [EC:3.1.3.5]	0.11	8.1E-19	1.1E-18	0.12	
K00901	diacylglycerol kinase (ATP) [EC:2.7.1.107]	-0.11	9.0E-19	1.2E-18	-0.22	
K01186	sialidase-1 [EC:3.2.1.18]	-0.10	1.0E-18	1.4E-18	-0.22	
K08322	L-gulonate 5-dehydrogenase [EC:1.1.1.380]	0.10	1.1E-18	1.5E-18	0.11	
K02344	DNA polymerase III subunit psi [EC:2.7.7.7]	0.10	1.2E-18	1.7E-18	0.11	
K02523	octaprenyl-diphosphate synthase [EC:2.5.1.90]	-0.10	1.9E-18	2.6E-18	-0.20	
K03780	L(+)-tartrate dehydratase beta subunit [EC:4.2.1.32]	0.10	1.9E-18	2.6E-18	0.11	
K07648	two-component system, OmpR family, aerobic respiration control sensor histidine kinase ArcB [EC:2.7.13.3]	0.10	2.7E-18	3.7E-18	0.11	
K13938	dihydromonapterin reductase / dihydrofolate reductase [EC:1.5.1.50 1.5.1.3]	0.10	2.9E-18	4.0E-18	0.12	

Predictor	Description	Pearson's r	P	FDR-adjusted P	Spearman's rho	Associated with fractures (P<0.05)
K00884	N-acetylglucosamine kinase [EC:2.7.1.59]	0.10	2.9E-18	4.0E-18	0.11	
K05926	23S rRNA (adenosine1067-2'-O)-methyltransferase [EC:2.1.1.230]	0.10	3.1E-18	4.3E-18	0.13	
K13086	mannosylfructose-6-phosphate phosphatase [EC:3.1.3.79]	0.10	3.2E-18	4.4E-18	0.10	
K15242	2,6-dichloro-p-hydroquinone 1,2-dioxygenase [EC:1.13.11.-]	0.10	3.9E-18	5.4E-18	0.12	
K00791	tRNA dimethylallyltransferase [EC:2.5.1.75]	-0.10	4.3E-18	5.9E-18	-0.24	
K00222	Delta14-sterol reductase [EC:1.3.1.70]	0.10	4.8E-18	6.6E-18	0.09	
K01115	phospholipase D1/2 [EC:3.1.4.4]	0.10	5.0E-18	6.9E-18	0.16	
K15760	toluene monooxygenase system protein A [EC:1.14.13.236 1.14.13.-]	0.10	5.2E-18	7.2E-18	0.14	
K00053	ketol-acid reductoisomerase [EC:1.1.1.86]	0.10	5.4E-18	7.4E-18	0.13	
K15863	NADH-quinone oxidoreductase subunit L/M [EC:7.1.1.2]	0.10	5.6E-18	7.7E-18	0.10	
K05946	N-acetylglucosaminyldiphosphoundecaprenol N-acetyl-beta-D-mannosaminyltransferase [EC:2.4.1.187]	-0.10	5.8E-18	8.0E-18	-0.20	
K08314	fructose-6-phosphate aldolase 2 [EC:4.1.2.-]	0.10	5.9E-18	8.1E-18	0.11	
K01921	D-alanine-D-alanine ligase [EC:6.3.2.4]	0.10	6.2E-18	8.5E-18	0.15	
K00687	penicillin-binding protein 2B	0.10	6.5E-18	8.9E-18	0.10	
K12152	phosphatase NudJ [EC:3.6.1.-]	0.10	8.6E-18	1.2E-17	0.11	
K03771	peptidyl-prolyl cis-trans isomerase SurA [EC:5.2.1.8]	-0.10	9.4E-18	1.3E-17	-0.22	
K02472	UDP-N-acetyl-D-mannosaminuronic acid dehydrogenase [EC:1.1.1.336]	-0.10	9.5E-18	1.3E-17	-0.22	
K05601	hydroxylamine reductase [EC:1.7.99.1]	0.10	9.8E-18	1.3E-17	0.13	
K09758	aspartate 4-decarboxylase [EC:4.1.1.12]	-0.10	1.4E-17	1.9E-17	-0.24	
K00001	alcohol dehydrogenase [EC:1.1.1.1]	-0.10	1.5E-17	2.1E-17	-0.21	
K01924	UDP-N-acetylmuramate--alanine ligase [EC:6.3.2.8]	0.10	1.5E-17	2.1E-17	0.15	yes
K01573	oxaloacetate decarboxylase (Na+ extruding) subunit gamma	-0.10	1.6E-17	2.2E-17	-0.24	
K15907	pentalenene oxygenase [EC:1.14.15.32]	0.10	1.6E-17	2.2E-17	0.09	
K01649	2-isopropylmalate synthase [EC:2.3.3.13]	0.10	1.8E-17	2.5E-17	0.14	
K01686	mannonate dehydratase [EC:4.2.1.8]	-0.10	1.9E-17	2.6E-17	-0.17	
K11264	methylmalonyl-CoA decarboxylase [EC:4.1.1.-]	0.10	1.9E-17	2.6E-17	0.11	
K08678	UDP-glucuronate decarboxylase [EC:4.1.1.35]	-0.10	2.2E-17	3.0E-17	-0.20	
K13059	N-acetylhexosamine 1-kinase [EC:2.7.1.162]	0.10	2.3E-17	3.1E-17	0.12	
K01491	methylenetetrahydrofolate dehydrogenase (NADP+) / methenyltetrahydrofolate cyclohydrolase [EC:1.5.1.5 3.5.4.9]	0.10	2.5E-17	3.4E-17	0.12	
K03183	demethylmenaquinone methyltransferase / 2-methoxy-6-polyprenyl-1,4-benzoquinol methylase [EC:2.1.1.163 2.1.1.201]	-0.10	2.8E-17	3.8E-17	-0.14	
K01492	phosphoribosylglycinamide/phosphoribosylaminoimidazolecarboxamide formyltransferase [EC:2.1.2.2 2.1.2.3]	-0.10	2.9E-17	4.0E-17	-0.24	
K15018	3-hydroxypropionyl-coenzyme A synthetase [EC:6.2.1.36]	0.10	3.1E-17	4.2E-17	0.10	
K01144	NA	-0.10	3.2E-17	4.4E-17	-0.24	
K01744	aspartate ammonia-lyase [EC:4.3.1.1]	-0.10	3.2E-17	4.4E-17	-0.20	
K13656	undecaprenyl-phosphate glucose phosphotransferase [EC:2.7.8.31]	0.10	3.4E-17	4.6E-17	0.12	
K01818	L-fucose/D-arabinose isomerase [EC:5.3.1.25 5.3.1.3]	-0.10	4.4E-17	6.0E-17	-0.23	
K05301	sulfite dehydrogenase (cytochrome) subunit A [EC:1.8.2.1]	0.10	4.8E-17	6.5E-17	0.15	
K05368	NAD(P)H-flavin reductase [EC:1.5.1.41]	0.10	5.1E-17	6.9E-17	0.11	
K01964	acetyl-CoA/propionyl-CoA carboxylase [EC:6.4.1.2 6.4.1.3]	0.10	5.7E-17	7.7E-17	0.10	
K05575	NAD(P)H-quinone oxidoreductase subunit 4 [EC:7.1.1.2]	0.10	5.8E-17	7.9E-17	0.11	
K15976	putative NAD(P)H nitroreductase [EC:1.-.-.-]	0.10	6.6E-17	9.0E-17	0.15	
K00892	inosine kinase [EC:2.7.1.73]	0.10	7.3E-17	9.9E-17	0.11	

Predictor	Description	Pearson's r	P	FDR-adjusted P	Spearman's rho	Associated with fractures (P<0.05)
K15538	glycoprotein endo-alpha-1,2-mannosidase [EC:3.2.1.130]	0.10	8.5E-17	1.2E-16	0.10	
K13065	shikimate O-hydroxycinnamoyltransferase [EC:2.3.1.133]	0.10	8.5E-17	1.2E-16	0.10	
K14581	naphthalene 1,2-dioxygenase ferredoxin reductase component [EC:1.18.1.7]	0.10	8.6E-17	1.2E-16	0.14	
K01895	acetyl-CoA synthetase [EC:6.2.1.1]	-0.10	9.3E-17	1.3E-16	-0.17	
K12525	bifunctional aspartokinase / homoserine dehydrogenase 2 [EC:2.7.2.4 1.1.1.3]	0.10	1.2E-16	1.6E-16	0.11	
K07806	UDP-4-amino-4-deoxy-L-arabinose-oxoglutarate aminotransferase [EC:2.6.1.87]	0.10	1.4E-16	1.9E-16	0.10	
K01191	alpha-mannosidase [EC:3.2.1.24]	0.10	1.5E-16	2.0E-16	0.09	
K01914	aspartate--ammonia ligase [EC:6.3.1.1]	-0.10	1.5E-16	2.0E-16	-0.19	
K00993	ethanolaminephosphotransferase [EC:2.7.8.1]	0.10	1.5E-16	2.0E-16	0.09	yes
K01407	protease III [EC:3.4.24.55]	0.10	1.7E-16	2.3E-16	0.11	
K03177	tRNA pseudouridine55 synthase [EC:5.4.99.25]	0.10	1.8E-16	2.4E-16	0.12	
K06949	ribosome biogenesis GTPase / thiamine phosphate phosphatase [EC:3.6.1.- 3.1.3.100]	-0.10	1.9E-16	2.6E-16	-0.13	
K10678	nitroreductase [EC:1.-.-.-]	0.10	2.0E-16	2.7E-16	0.11	
K00077	2-dehydropantoate 2-reductase [EC:1.1.1.169]	-0.10	2.0E-16	2.7E-16	-0.18	
K07811	trimethylamine-N-oxide reductase (cytochrome c) [EC:1.7.2.3]	0.10	2.1E-16	2.8E-16	0.10	
K07704	two-component system, LytTR family, sensor histidine kinase LytS [EC:2.7.13.3]	0.10	2.2E-16	3.0E-16	0.12	
K07674	two-component system, NarL family, nitrate/nitrite sensor histidine kinase NarQ [EC:2.7.13.3]	0.10	2.4E-16	3.2E-16	0.10	
K02846	N-methyl-L-tryptophan oxidase [EC:1.5.3.-]	0.10	2.5E-16	3.4E-16	0.11	
K11933	NADH oxidoreductase Hcr [EC:1.-.-.-]	0.10	2.5E-16	3.4E-16	0.10	
K07264	4-amino-4-deoxy-L-arabinose transferase [EC:2.4.2.43]	0.10	2.8E-16	3.8E-16	0.11	
K02364	L-serine---[L-seryl-carrier protein] ligase [EC:6.3.2.14 6.2.1.72]	0.10	2.8E-16	3.8E-16	0.11	
K00018	glycerate dehydrogenase [EC:1.1.1.29]	-0.10	2.9E-16	3.9E-16	-0.18	
K11608	mycobacterial beta-ketoacyl-[acyl-carrier-protein] synthase III [EC:2.3.1.301]	0.10	3.0E-16	4.0E-16	0.15	
K01755	argininosuccinate lyase [EC:4.3.2.1]	-0.10	3.3E-16	4.4E-16	-0.17	
K00350	Na+-transporting NADH:ubiquinone oxidoreductase subunit E [EC:7.2.1.1]	-0.10	3.7E-16	5.0E-16	-0.22	
K01096	phosphatidylglycerophosphatase B [EC:3.1.3.27 3.1.3.81 3.1.3.4 3.6.1.27]	0.10	3.8E-16	5.1E-16	0.10	
K00351	Na+-transporting NADH:ubiquinone oxidoreductase subunit F [EC:7.2.1.1]	-0.10	3.8E-16	5.1E-16	-0.22	
K05306	phosphonoacetaldehyde hydrolase [EC:3.11.1.1]	-0.10	4.1E-16	5.5E-16	-0.21	
K13574	hydroxycarboxylate dehydrogenase B [EC:1.1.1.237 1.1.1.-]	0.10	4.2E-16	5.6E-16	0.11	
K00874	2-dehydro-3-deoxygluconokinase [EC:2.7.1.45]	-0.10	4.5E-16	6.0E-16	-0.17	
K00951	GTP pyrophosphokinase [EC:2.7.6.5]	-0.10	4.7E-16	6.3E-16	-0.15	
K00174	2-oxoglutarate/2-oxoacid ferredoxin oxidoreductase subunit alpha [EC:1.2.7.3 1.2.7.11]	-0.10	5.2E-16	7.0E-16	-0.20	
K04564	superoxide dismutase, Fe-Mn family [EC:1.15.1.1]	-0.10	5.5E-16	7.4E-16	-0.14	
K11202	fructose-like PTS system EIIB component [EC:2.7.1.-]	0.10	6.0E-16	8.0E-16	0.10	
K10011	UDP-4-amino-4-deoxy-L-arabinose formyltransferase / UDP-glucuronic acid dehydrogenase (UDP-4-keto-hexauronic acid decarboxylating) [EC:2.1.2.13 1.1.1.305]	0.10	6.2E-16	8.3E-16	0.10	
K01119	2',3'-cyclic-nucleotide 2'-phosphodiesterase / 3'-nucleotidase [EC:3.1.4.16 3.1.3.6]	-0.10	6.5E-16	8.7E-16	-0.19	
K00983	N-acylneuraminate cytidylyltransferase [EC:2.7.7.43]	-0.10	7.1E-16	9.5E-16	-0.19	
K02346	DNA polymerase IV [EC:2.7.7.7]	-0.10	7.1E-16	9.5E-16	-0.10	
K03054	DNA-directed RNA polymerase subunit I [EC:2.7.7.6]	0.10	8.4E-16	1.1E-15	0.10	
K02362	enterobactin synthetase component D [EC:6.3.2.14 2.7.8.-]	0.10	9.3E-16	1.2E-15	0.11	
K01610	phosphoenolpyruvate carboxykinase (ATP) [EC:4.1.1.49]	-0.10	9.5E-16	1.3E-15	-0.18	
K03207	colanic acid biosynthesis protein WcaH [EC:3.6.1.-]	0.10	9.8E-16	1.3E-15	0.10	

Predictor	Description	Pearson's r	P	FDR-adjusted P	Spearman's rho	Associated with fractures (P<0.05)
K08324	succinate-semialdehyde dehydrogenase [EC:1.2.1.16 1.2.1.24]	0.10	1.2E-15	1.6E-15	0.10	
K10834	heme transport system ATP-binding protein [EC:7.6.2.5]	0.09	1.4E-15	1.9E-15	0.09	
K11527	two-component system, sensor histidine kinase and response regulator [EC:2.7.13.3]	-0.09	1.5E-15	2.0E-15	-0.22	
K00554	tRNA (guanine37-N1)-methyltransferase [EC:2.1.1.228]	0.09	1.5E-15	2.0E-15	0.15	yes
K08289	phosphoribosylglycinamide formyltransferase 2 [EC:6.3.1.21]	-0.09	1.5E-15	2.0E-15	-0.18	
K12343	3-oxo-5-alpha-steroid 4-dehydrogenase 1 [EC:1.3.1.22]	-0.09	1.5E-15	2.0E-15	-0.20	
K10909	two-component system, autoinducer 2 sensor kinase/phosphatase LuxQ [EC:2.7.13.3 3.1.3.-]	0.09	1.6E-15	2.1E-15	0.10	
K15929	glycosyltransferase [EC:2.4.1.-]	0.09	1.6E-15	2.1E-15	0.09	
K03169	DNA topoisomerase III [EC:5.6.2.1]	-0.09	1.7E-15	2.3E-15	-0.16	
K10206	LL-diaminopimelate aminotransferase [EC:2.6.1.83]	-0.09	2.1E-15	2.8E-15	-0.21	
K13043	N-succinyl-L-ornithine transcarbamylase [EC:2.1.3.11]	-0.09	2.1E-15	2.8E-15	-0.20	
K02853	enterobacterial common antigen polymerase [EC:2.4.1.-]	0.09	2.3E-15	3.0E-15	0.10	
K01297	muramoyltetrapeptide carboxypeptidase [EC:3.4.17.13]	0.09	2.8E-15	3.7E-15	0.10	
K12973	lipid IVA palmitoyltransferase [EC:2.3.1.251]	0.09	3.1E-15	4.1E-15	0.10	
K01205	alpha-N-acetylglucosaminidase [EC:3.2.1.50]	-0.09	3.4E-15	4.5E-15	-0.20	
K00179	indolepyruvate ferredoxin oxidoreductase, alpha subunit [EC:1.2.7.8]	-0.09	3.6E-15	4.8E-15	-0.19	
K01885	glutamyl-tRNA synthetase [EC:6.1.1.17]	0.09	4.0E-15	5.3E-15	0.13	
K12373	hexosaminidase [EC:3.2.1.52]	-0.09	4.7E-15	6.2E-15	-0.21	
K05970	sialate O-acetylesterase [EC:3.1.1.53]	-0.09	5.0E-15	6.6E-15	-0.19	
K01547	potassium-transporting ATPase ATP-binding subunit [EC:7.2.2.6]	-0.09	5.1E-15	6.7E-15	-0.17	
K13069	diguanylate cyclase [EC:2.7.7.65]	0.09	5.2E-15	6.9E-15	0.10	
K02469	DNA gyrase subunit A [EC:5.6.2.2]	0.09	5.3E-15	7.0E-15	0.12	
K15922	sulfoquinovosidase [EC:3.2.1.199]	0.09	5.8E-15	7.7E-15	0.10	
K16176	methylamine---corrinoid protein Co-methyltransferase [EC:2.1.1.248]	0.09	5.9E-15	7.8E-15	0.08	
K02536	UDP-3-O-[3-hydroxymyristoyl] glucosamine N-acyltransferase [EC:2.3.1.191]	-0.09	6.0E-15	7.9E-15	-0.19	
K13014	undecaprenyl phosphate-alpha-L-ara4FN deformylase [EC:3.5.1.-]	0.09	7.0E-15	9.2E-15	0.10	
K07701	two-component system, CitB family, sensor histidine kinase DcuS [EC:2.7.13.3]	0.09	7.1E-15	9.3E-15	0.10	
K12251	N-carbamoylputrescine amidase [EC:3.5.1.53]	-0.09	8.3E-15	1.1E-14	-0.20	
K00820	glutamine---fructose-6-phosphate transaminase (isomerizing) [EC:2.6.1.16]	0.09	9.0E-15	1.2E-14	0.13	yes
K12139	hydrogenase-4 component D [EC:1.-.-.-]	0.09	1.1E-14	1.4E-14	0.10	
K12582	dTDP-N-acetylfucosamine:lipid II N-acetylfucosaminyltransferase [EC:2.4.1.325]	0.09	1.1E-14	1.4E-14	0.10	
K00046	gluconate 5-dehydrogenase [EC:1.1.1.69]	-0.09	1.2E-14	1.6E-14	-0.14	
K00847	fructokinase [EC:2.7.1.4]	-0.09	1.3E-14	1.7E-14	-0.22	
K05593	aminoglycoside 6-adenylyltransferase [EC:2.7.7.-]	0.09	1.5E-14	2.0E-14	0.11	
K00281	glycine dehydrogenase [EC:1.4.4.2]	-0.09	1.7E-14	2.2E-14	-0.19	
K03100	signal peptidase I [EC:3.4.21.89]	-0.09	2.0E-14	2.6E-14	-0.16	
K13450	phosphothreonine lyase [EC:4.2.3.-]	0.09	2.1E-14	2.8E-14	0.11	
K00097	4-hydroxythreonine-4-phosphate dehydrogenase [EC:1.1.1.262]	-0.09	2.3E-14	3.0E-14	-0.18	
K07588	GTPase [EC:3.6.5.-]	-0.09	2.3E-14	3.0E-14	-0.15	
K13245	c-di-GMP-specific phosphodiesterase [EC:3.1.4.52]	0.09	2.5E-14	3.3E-14	0.10	
K13876	2-keto-3-deoxy-L-arabinonate dehydratase [EC:4.2.1.43]	0.09	2.6E-14	3.4E-14	0.10	
K03182	4-hydroxy-3-polyprenylbenzoate decarboxylase [EC:4.1.1.98]	0.09	2.7E-14	3.5E-14	0.09	

Predictor	Description	Pearson's r	P	FDR-adjusted P	Spearman's rho	Associated with fractures (P<0.05)
K02564	glucosamine-6-phosphate deaminase [EC:3.5.99.6]	-0.09	2.8E-14	3.7E-14	-0.12	
K07251	thiamine kinase [EC:2.7.1.89]	0.09	2.8E-14	3.7E-14	0.10	
K08253	non-specific protein-tyrosine kinase [EC:2.7.10.2]	0.09	3.1E-14	4.1E-14	0.12	
K01160	crossover junction endodeoxyribonuclease RusA [EC:3.1.21.10]	0.09	4.0E-14	5.2E-14	0.09	
K03820	apolipoprotein N-acyltransferase [EC:2.3.1.269]	0.09	5.3E-14	6.9E-14	0.09	
K06074	vitamin B12 transport system ATP-binding protein [EC:7.6.2.8]	0.09	5.6E-14	7.3E-14	0.10	
K00652	8-amino-7-oxononanoate synthase [EC:2.3.1.47]	-0.09	5.9E-14	7.7E-14	-0.20	
K02345	DNA polymerase III subunit theta [EC:2.7.7.7]	0.09	6.4E-14	8.3E-14	0.09	
K00885	N-acylmannosamine kinase [EC:2.7.1.60]	0.09	6.5E-14	8.5E-14	0.09	
K07757	sugar-phosphatase [EC:3.1.3.23]	0.09	7.1E-14	9.2E-14	0.09	
K11391	23S rRNA (guanine1835-N2)-methyltransferase [EC:2.1.1.174]	0.09	7.3E-14	9.5E-14	0.09	
K08682	acyl carrier protein phosphodiesterase [EC:3.1.4.14]	0.09	7.6E-14	9.9E-14	0.10	
K02527	3-deoxy-D-manno-octulosonic-acid transferase [EC:2.4.99.12 2.4.99.13 2.4.99.14 2.4.99.15]	-0.09	7.7E-14	1.0E-13	-0.19	
K15635	2,3-bisphosphoglycerate-independent phosphoglycerate mutase [EC:5.4.2.12]	-0.09	8.2E-14	1.1E-13	-0.20	
K02377	GDP-L-fucose synthase [EC:1.1.1.271]	-0.09	8.2E-14	1.1E-13	-0.20	
K09020	ureidoacrylate peracid hydrolase [EC:3.5.1.110]	0.09	8.4E-14	1.1E-13	0.10	
K01689	enolase [EC:4.2.1.11]	0.09	9.5E-14	1.2E-13	0.15	yes
K03474	pyridoxine 5-phosphate synthase [EC:2.6.99.2]	-0.09	9.7E-14	1.3E-13	-0.15	
K01156	type III restriction enzyme [EC:3.1.21.5]	0.09	9.7E-14	1.3E-13	0.09	
K04708	3-dehydrosphinganine reductase [EC:1.1.1.102]	0.09	9.9E-14	1.3E-13	0.08	
K11627	pyrrolysyl-tRNA synthetase [EC:6.1.1.26]	0.09	1.0E-13	1.3E-13	0.10	
K00029	malate dehydrogenase (oxaloacetate-decarboxylating)(NADP+) [EC:1.1.1.40]	-0.09	1.1E-13	1.4E-13	-0.15	
K07676	two-component system, NarL family, sensor histidine kinase RcsD [EC:2.7.13.3]	0.09	1.1E-13	1.4E-13	0.09	
K01785	aldose 1-epimerase [EC:5.1.3.3]	-0.09	1.1E-13	1.4E-13	-0.17	
K00859	dephospho-CoA kinase [EC:2.7.1.24]	-0.09	1.2E-13	1.6E-13	-0.11	
K00338	NADH-quinone oxidoreductase subunit I [EC:7.1.1.2]	-0.09	1.2E-13	1.6E-13	-0.18	
K12944	nucleoside triphosphatase [EC:3.6.1.-]	0.09	1.3E-13	1.7E-13	0.09	
K01093	4-phytase / acid phosphatase [EC:3.1.3.26 3.1.3.2]	0.09	1.4E-13	1.8E-13	0.10	
K00211	prephenate dehydrogenase (NADP+) [EC:1.3.1.13]	0.09	1.5E-13	1.9E-13	0.13	
K08320	(d)CTP diphosphatase [EC:3.6.1.65]	0.09	1.5E-13	1.9E-13	0.09	
K05535	alpha 1,2-mannosyltransferase [EC:2.4.1.-]	0.09	1.6E-13	2.1E-13	0.08	
K08685	quinohemoprotein amine dehydrogenase [EC:1.4.9.1]	0.09	1.8E-13	2.3E-13	0.15	
K12136	hydrogenase-4 component A [EC:1.-.-.-]	0.09	1.8E-13	2.3E-13	0.08	
K01169	ribonuclease I (enterobacter ribonuclease) [EC:4.6.1.21]	0.09	1.9E-13	2.4E-13	0.09	
K00342	NADH-quinone oxidoreductase subunit M [EC:7.1.1.2]	-0.09	1.9E-13	2.4E-13	-0.18	
K09023	aminoacrylate hydrolase [EC:3.5.1.-]	0.09	2.1E-13	2.7E-13	0.09	
K00691	maltose phosphorylase [EC:2.4.1.8]	0.09	2.1E-13	2.7E-13	0.09	
K06941	23S rRNA (adenine2503-C2)-methyltransferase [EC:2.1.1.192]	0.09	2.2E-13	2.8E-13	0.16	
K00208	enoyl-[acyl-carrier protein] reductase I [EC:1.3.1.9 1.3.1.10]	-0.09	2.3E-13	3.0E-13	-0.18	
K04118	pimeloyl-CoA dehydrogenase [EC:1.3.1.62]	0.09	2.3E-13	3.0E-13	0.09	
K01446	peptidoglycan recognition protein	0.09	2.6E-13	3.3E-13	0.10	
K12570	streptomycin 6-kinase [EC:2.7.1.72]	0.09	2.6E-13	3.3E-13	0.10	

Predictor	Description	Pearson's r	P	FDR-adjusted P	Spearman's rho	Associated with fractures (P<0.05)
K01661	naphthoate synthase [EC:4.1.3.36]	-0.09	2.9E-13	3.7E-13	-0.19	
K07643	two-component system, OmpR family, sensor histidine kinase BasS [EC:2.7.13.3]	0.09	2.9E-13	3.7E-13	0.09	
K01711	GDPmannose 4,6-dehydratase [EC:4.2.1.47]	-0.09	3.3E-13	4.2E-13	-0.18	
K00979	3-deoxy-manno-octulosonate cytidylyltransferase (CMP-KDO synthetase) [EC:2.7.7.38]	-0.09	3.3E-13	4.2E-13	-0.18	
K14051	c-di-GMP phosphodiesterase Gmr [EC:3.1.4.52]	0.09	3.5E-13	4.5E-13	0.09	
K05592	ATP-dependent RNA helicase DeaD [EC:3.6.4.13]	-0.09	3.7E-13	4.7E-13	-0.18	
K03534	L-rhamnose mutarotase [EC:5.1.3.32]	-0.09	4.1E-13	5.3E-13	-0.19	
K01241	AMP nucleosidase [EC:3.2.2.4]	-0.09	4.1E-13	5.3E-13	-0.21	
K00860	adenylylsulfate kinase [EC:2.7.1.25]	-0.09	4.5E-13	5.8E-13	-0.21	
K02361	isochorismate synthase [EC:5.4.4.2]	-0.09	4.6E-13	5.9E-13	-0.21	
K00036	glucose-6-phosphate 1-dehydrogenase [EC:1.1.1.49 1.1.1.363]	-0.09	4.8E-13	6.1E-13	-0.21	
K07316	adenine-specific DNA-methyltransferase [EC:2.1.1.72]	0.09	4.8E-13	6.1E-13	0.09	
K02551	2-succinyl-5-enolpyruvyl-6-hydroxy-3-cyclohexene-1-carboxylate synthase [EC:2.2.1.9]	-0.09	4.9E-13	6.3E-13	-0.19	
K14082	[methyl-Co(III) methylamine-specific corrinoid protein]:coenzyme M methyltransferase [EC:2.1.1.247]	0.09	5.0E-13	6.4E-13	0.09	
K00956	sulfate adenylyltransferase subunit 1 [EC:2.7.7.4]	-0.09	5.3E-13	6.8E-13	-0.21	
K02840	UDP-D-galactose:(glucosyl)LPS alpha-1,6-D-galactosyltransferase [EC:2.4.1.-]	0.09	6.2E-13	7.9E-13	0.11	
K01450	NA	0.09	6.5E-13	8.3E-13	0.08	
K07259	serine-type D-Ala-D-Ala carboxypeptidase/endopeptidase (penicillin-binding protein 4) [EC:3.4.16.4 3.4.21.-]	-0.09	6.6E-13	8.4E-13	-0.21	
K14466	4-hydroxybutyrate---CoA ligase (AMP-forming) [EC:6.2.1.40]	0.09	6.6E-13	8.4E-13	0.10	
K08348	formate dehydrogenase-N, alpha subunit [EC:1.17.5.3]	0.09	6.9E-13	8.8E-13	0.09	
K01202	galactosylceramidase [EC:3.2.1.46]	0.09	8.4E-13	1.1E-12	0.08	
K01104	protein-tyrosine phosphatase [EC:3.1.3.48]	0.09	8.9E-13	1.1E-12	0.10	
K01278	dipeptidyl-peptidase 4 [EC:3.4.14.5]	-0.08	1.0E-12	1.3E-12	-0.21	
K03819	putative colanic acid biosynthesis acetyltransferase WcaB [EC:2.3.1.-]	0.08	1.2E-12	1.5E-12	0.09	
K11645	fructose-bisphosphate aldolase, class I [EC:4.1.2.13]	-0.08	1.3E-12	1.7E-12	-0.15	
K12945	GDP-mannose pyrophosphatase NudK [EC:3.6.1.-]	0.08	1.4E-12	1.8E-12	0.09	
K01461	N-acyl-D-glutamate deacylase [EC:3.5.1.82]	0.08	1.5E-12	1.9E-12	0.08	
K01081	5'-nucleotidase [EC:3.1.3.5]	-0.08	1.6E-12	2.0E-12	-0.23	
K08308	membrane-bound lytic murein transglycosylase E [EC:4.2.2.-]	0.08	1.6E-12	2.0E-12	0.09	
K13281	UV DNA damage endonuclease [EC:3.-.-.-]	0.08	1.7E-12	2.2E-12	0.12	
K03367	D-alanine--poly(phosphoribitol) ligase subunit 1 [EC:6.1.1.13]	0.08	1.9E-12	2.4E-12	0.08	
K02230	cobaltochelatase CobN [EC:6.6.1.2]	-0.08	2.2E-12	2.8E-12	-0.20	
K05286	GPI mannosyltransferase 3 [EC:2.4.1.-]	0.08	2.4E-12	3.0E-12	0.09	
K00152	salicylaldehyde dehydrogenase [EC:1.2.1.65]	0.08	2.5E-12	3.2E-12	0.09	
K01460	glutathionylspermidine amidase/synthetase [EC:3.5.1.78 6.3.1.8]	0.08	2.8E-12	3.5E-12	0.09	
K12145	hydrogenase-4 component J [EC:1.-.-.-]	0.08	2.8E-12	3.5E-12	0.09	
K00969	nicotinate-nucleotide adenylyltransferase [EC:2.7.7.18]	0.08	3.0E-12	3.8E-12	0.15	
K00840	succinylornithine aminotransferase [EC:2.6.1.81]	0.08	3.1E-12	3.9E-12	0.09	
K12974	KDO2-lipid IV(A) palmitoleoyltransferase [EC:2.3.1.242]	0.08	3.3E-12	4.2E-12	0.09	
K01341	kexin [EC:3.4.21.61]	0.08	3.4E-12	4.3E-12	0.12	
K03052	DNA-directed RNA polymerase subunit G [EC:2.7.7.6]	0.08	3.4E-12	4.3E-12	0.07	
K05841	sterol 3beta-glucosyltransferase [EC:2.4.1.173]	0.08	3.4E-12	4.3E-12	0.11	

Predictor	Description	Pearson's r	P	FDR-adjusted P	Spearman's rho	Associated with fractures (P<0.05)
K11938	HMP-PP phosphatase [EC:3.6.1.-]	0.08	3.6E-12	4.5E-12	0.09	
K01524	exopolyphosphatase / guanosine-5'-triphosphate,3'-diphosphate pyrophosphatase [EC:3.6.1.11 3.6.1.40]	0.08	4.6E-12	5.8E-12	0.11	
K02506	leader peptidase HopD [EC:3.4.23.43]	0.08	4.6E-12	5.8E-12	0.09	
K05606	methylmalonyl-CoA/ethylmalonyl-CoA epimerase [EC:5.1.99.1]	0.08	5.6E-12	7.1E-12	0.08	
K11754	dihydrofolate synthase / folylpolyglutamate synthase [EC:6.3.2.12 6.3.2.17]	0.08	5.9E-12	7.4E-12	0.13	yes
K11201	fructose-like PTS system EIIA component [EC:2.7.1.-]	0.08	6.1E-12	7.7E-12	0.08	
K02850	heptose II phosphotransferase [EC:2.7.1.-]	0.08	6.1E-12	7.7E-12	0.09	
K08280	lipopolysaccharide O-acetyltransferase [EC:2.3.1.-]	0.08	6.9E-12	8.7E-12	0.10	
K03815	xanthosine phosphorylase [EC:2.4.2.-]	0.08	8.2E-12	1.0E-11	0.09	
K00057	glycerol-3-phosphate dehydrogenase (NAD(P)+) [EC:1.1.1.94]	0.08	8.3E-12	1.0E-11	0.11	
K01246	DNA-3-methyladenine glycosylase I [EC:3.2.2.20]	-0.08	8.8E-12	1.1E-11	-0.13	
K09018	pyrimidine oxygenase [EC:1.14.99.46]	0.08	8.9E-12	1.1E-11	0.09	
K01886	glutaminyl-tRNA synthetase [EC:6.1.1.18]	-0.08	9.1E-12	1.1E-11	-0.11	
K12660	2-dehydro-3-deoxy-L-rhamnonate aldolase [EC:4.1.2.53]	0.08	9.6E-12	1.2E-11	0.09	
K07647	two-component system, OmpR family, sensor histidine kinase TorS [EC:2.7.13.3]	0.08	9.9E-12	1.2E-11	0.09	
K12250	pentalenene synthase [EC:4.2.3.7]	0.08	1.1E-11	1.4E-11	0.07	
K04340	scyllo-inosamine-4-phosphate amidinotransferase 1 [EC:2.1.4.2]	0.08	1.1E-11	1.4E-11	0.07	
K14742	tRNA threonylcarbamoyladenosine biosynthesis protein TsaB	-0.08	1.2E-11	1.5E-11	-0.14	
K13985	N-acyl-phosphatidylethanolamine-hydrolysing phospholipase D [EC:3.1.4.54]	0.08	1.2E-11	1.5E-11	0.08	
K00743	N-acetyllactosaminide 3-alpha-galactosyltransferase [EC:2.4.1.87]	0.08	1.3E-11	1.6E-11	0.09	
K14585	trans-o-hydroxybenzylidenepyruvate hydratase-aldolase [EC:4.1.2.45]	0.08	1.3E-11	1.6E-11	0.09	
K00757	uridine phosphorylase [EC:2.4.2.3]	-0.08	1.5E-11	1.9E-11	-0.14	
K00094	galactitol-1-phosphate 5-dehydrogenase [EC:1.1.1.251]	0.08	1.8E-11	2.2E-11	0.09	
K03340	diaminopimelate dehydrogenase [EC:1.4.1.16]	-0.08	1.8E-11	2.2E-11	-0.19	
K12138	hydrogenase-4 component C [EC:1.-.-.-]	0.08	1.9E-11	2.4E-11	0.07	
K01155	type II restriction enzyme [EC:3.1.21.4]	-0.08	1.9E-11	2.4E-11	-0.22	
K03040	DNA-directed RNA polymerase subunit alpha [EC:2.7.7.6]	0.08	1.9E-11	2.4E-11	0.11	
K03773	FKBP-type peptidyl-prolyl cis-trans isomerase FklB [EC:5.2.1.8]	-0.08	2.4E-11	3.0E-11	-0.21	
K07229	ferric-chelate reductase (NADPH) [EC:1.16.1.9]	0.08	2.6E-11	3.2E-11	0.08	
K02464	general secretion pathway protein O [EC:3.4.23.43 2.1.1.-]	0.08	2.8E-11	3.5E-11	0.10	
K11755	phosphoribosyl-AMP cyclohydrolase / phosphoribosyl-ATP pyrophosphohydrolase [EC:3.5.4.19 3.6.1.31]	-0.08	2.8E-11	3.5E-11	-0.13	
K00175	2-oxoglutarate/2-oxoacid ferredoxin oxidoreductase subunit beta [EC:1.2.7.3 1.2.7.11]	-0.08	3.0E-11	3.7E-11	-0.18	
K06113	arabinan endo-1,5-alpha-L-arabinosidase [EC:3.2.1.99]	-0.08	3.2E-11	4.0E-11	-0.18	
K01057	6-phosphogluconolactonase [EC:3.1.1.31]	-0.08	3.5E-11	4.4E-11	-0.21	
K02190	sirohydrochlorin cobaltochelatase [EC:4.99.1.3]	-0.08	3.6E-11	4.5E-11	-0.18	
K11927	ATP-dependent RNA helicase RhlE [EC:3.6.4.13]	-0.08	3.7E-11	4.6E-11	-0.10	
K01270	dipeptidase D [EC:3.4.13.-]	-0.08	4.2E-11	5.2E-11	-0.12	
K07700	two-component system, CitB family, cit operon sensor histidine kinase CitA [EC:2.7.13.3]	0.08	4.4E-11	5.5E-11	0.08	
K01712	urocanate hydratase [EC:4.2.1.49]	-0.08	4.5E-11	5.6E-11	-0.14	
K00600	glycine hydroxymethyltransferase [EC:2.1.2.1]	0.08	5.2E-11	6.5E-11	0.12	yes
K00639	glycine C-acetyltransferase [EC:2.3.1.29]	-0.08	5.2E-11	6.5E-11	-0.12	
K00609	aspartate carbamoyltransferase catalytic subunit [EC:2.1.3.2]	0.08	5.4E-11	6.7E-11	0.10	

Predictor	Description	Pearson's r	P	FDR-adjusted P	Spearman's rho	Associated with fractures (P<0.05)
K05367	penicillin-binding protein 1C [EC:2.4.1.129]	0.08	5.9E-11	7.3E-11	0.08	
K00962	polyribonucleotide nucleotidyltransferase [EC:2.7.7.8]	0.08	6.5E-11	8.1E-11	0.10	
K01054	acylglycerol lipase [EC:3.1.1.23]	0.08	7.3E-11	9.0E-11	0.06	
K00634	phosphate butyryltransferase [EC:2.3.1.19]	-0.08	7.3E-11	9.0E-11	-0.21	
K00833	adenosylmethionine---8-amino-7-oxononanoate aminotransferase [EC:2.6.1.62]	0.08	7.6E-11	9.4E-11	0.09	
K07310	Tat-targeted selenate reductase subunit YnfF [EC:1.97.1.9]	0.08	7.9E-11	9.8E-11	0.07	
K02079	N-acetylgalactosamine-6-phosphate deacetylase [EC:3.5.1.25]	0.08	7.9E-11	9.8E-11	0.09	
K15923	alpha-L-fucosidase 2 [EC:3.2.1.51]	-0.08	8.5E-11	1.1E-10	-0.20	
K09470	gamma-glutamylputrescine synthase [EC:6.3.1.11]	0.08	8.6E-11	1.1E-10	0.08	
K07319	adenine-specific DNA-methyltransferase [EC:2.1.1.72]	0.08	9.1E-11	1.1E-10	0.09	
K01468	imidazolonepropionase [EC:3.5.2.7]	-0.08	9.4E-11	1.2E-10	-0.14	
K08640	zinc D-Ala-D-Ala carboxypeptidase [EC:3.4.17.14]	0.08	1.0E-10	1.2E-10	0.08	
K13419	serine/threonine-protein kinase PknK [EC:2.7.11.1]	0.08	1.1E-10	1.4E-10	0.12	
K08325	NADP-dependent alcohol dehydrogenase [EC:1.1.-.-]	-0.08	1.1E-10	1.4E-10	-0.14	
K01810	glucose-6-phosphate isomerase [EC:5.3.1.9]	0.08	1.2E-10	1.5E-10	0.11	
K09568	FK506-binding protein 1 [EC:5.2.1.8]	0.08	1.3E-10	1.6E-10	0.09	
K01197	hyaluronoglucosaminidase [EC:3.2.1.35]	-0.08	1.4E-10	1.7E-10	-0.23	
K01429	urease subunit beta [EC:3.5.1.5]	0.08	1.4E-10	1.7E-10	0.07	
K12144	hydrogenase-4 component I [EC:1.-.-.-]	0.08	2.5E-10	3.1E-10	0.08	
K01089	imidazoleglycerol-phosphate dehydratase / histidinol-phosphatase [EC:4.2.1.19 3.1.3.15]	-0.08	2.7E-10	3.3E-10	-0.14	
K01262	Xaa-Pro aminopeptidase [EC:3.4.11.9]	-0.08	2.7E-10	3.3E-10	-0.11	
K00099	1-deoxy-D-xylulose-5-phosphate reductoisomerase [EC:1.1.1.267]	0.08	2.7E-10	3.3E-10	0.12	
K08307	membrane-bound lytic murein transglycosylase D [EC:4.2.2.-]	-0.07	3.1E-10	3.8E-10	-0.14	
K03269	UDP-2,3-diacylglucosamine hydrolase [EC:3.6.1.54]	-0.07	3.3E-10	4.1E-10	-0.14	
K00287	dihydrofolate reductase [EC:1.5.1.3]	0.07	3.4E-10	4.2E-10	0.10	
K12142	hydrogenase-4 component G [EC:1.-.-.-]	0.07	3.4E-10	4.2E-10	0.08	
K04720	threonine-phosphate decarboxylase [EC:4.1.1.81]	-0.07	3.8E-10	4.7E-10	-0.18	
K10775	phenylalanine ammonia-lyase [EC:4.3.1.24]	0.07	4.3E-10	5.3E-10	0.12	
K03080	NA	0.07	4.6E-10	5.6E-10	0.08	
K03655	ATP-dependent DNA helicase RecG [EC:3.6.4.12]	-0.07	5.6E-10	6.9E-10	-0.17	
K03270	3-deoxy-D-manno-octulosonate 8-phosphate phosphatase (KDO 8-P phosphatase) [EC:3.1.3.45]	-0.07	5.8E-10	7.1E-10	-0.14	
K15987	K(+)-stimulated pyrophosphate-energized sodium pump [EC:7.1.3.2]	-0.07	5.9E-10	7.2E-10	-0.13	
K01489	cytidine deaminase [EC:3.5.4.5]	-0.07	5.9E-10	7.2E-10	-0.13	
K01737	6-pyruvoyltetrahydropterin/6-carboxytetrahydropterin synthase [EC:4.2.3.12 4.1.2.50]	-0.07	6.3E-10	7.7E-10	-0.16	
K01840	phosphomannomutase [EC:5.4.2.8]	-0.07	6.7E-10	8.2E-10	-0.13	
K07964	heparanase [EC:3.2.1.166]	0.07	6.7E-10	8.2E-10	0.05	
K00991	2-C-methyl-D-erythritol 4-phosphate cytidylyltransferase [EC:2.7.7.60]	-0.07	6.9E-10	8.4E-10	-0.15	
K00655	1-acyl-sn-glycerol-3-phosphate acyltransferase [EC:2.3.1.51]	0.07	7.0E-10	8.6E-10	0.09	
K01633	7,8-dihydroneopterin aldolase/epimerase/oxygenase [EC:4.1.2.25 5.1.99.8 1.13.11.81]	-0.07	7.3E-10	8.9E-10	-0.12	
K14631	flavin reductase ActVB [EC:1.5.1.-]	0.07	8.1E-10	9.9E-10	0.11	
K00812	aspartate aminotransferase [EC:2.6.1.1]	-0.07	8.5E-10	1.0E-09	-0.19	
K09458	3-oxoacyl-[acyl-carrier-protein] synthase II [EC:2.3.1.179]	0.07	8.9E-10	1.1E-09	0.14	

Predictor	Description	Pearson's r	P	FDR-adjusted P	Spearman's rho	Associated with fractures (P<0.05)
K15064	syringate O-demethylase [EC:2.1.1.-]	0.07	1.0E-09	1.2E-09	0.07	
K08599	YopT peptidase [EC:3.4.22.-]	0.07	1.0E-09	1.2E-09	0.11	
K01745	histidine ammonia-lyase [EC:4.3.1.3]	-0.07	1.1E-09	1.3E-09	-0.12	
K09473	gamma-glutamyl-gamma-aminobutyrate hydrolase [EC:3.5.1.94]	0.07	1.1E-09	1.3E-09	0.08	
K13664	acyltransferase [EC:2.3.1.-]	0.07	1.2E-09	1.5E-09	0.07	
K13658	beta-1,4-mannosyltransferase [EC:2.4.1.251]	0.07	1.2E-09	1.5E-09	0.07	
K05986	nuclease S1 [EC:3.1.30.1]	0.07	1.2E-09	1.5E-09	0.07	
K01425	glutaminase [EC:3.5.1.2]	-0.07	1.2E-09	1.5E-09	-0.16	
K01719	uroporphyrinogen-III synthase [EC:4.2.1.75]	-0.07	1.3E-09	1.6E-09	-0.19	
K08838	serine/threonine-protein kinase 24/25/MST4 [EC:2.7.11.1]	0.07	1.4E-09	1.7E-09	0.06	
K00537	arsenate reductase (glutaredoxin) [EC:1.20.4.1]	0.07	1.5E-09	1.8E-09	0.07	
K03778	D-lactate dehydrogenase [EC:1.1.1.28]	-0.07	1.5E-09	1.8E-09	-0.09	
K02493	release factor glutamine methyltransferase [EC:2.1.1.297]	0.07	1.6E-09	1.9E-09	0.12	yes
K12137	hydrogenase-4 component B [EC:1.-.-.-]	0.07	1.6E-09	1.9E-09	0.07	
K02118	V/A-type H+/Na+-transporting ATPase subunit B	-0.07	1.7E-09	2.1E-09	-0.18	
K13243	c-di-GMP-specific phosphodiesterase [EC:3.1.4.52]	0.07	1.8E-09	2.2E-09	0.08	
K01947	biotin---[acetyl-CoA-carboxylase] ligase / type III pantothenate kinase [EC:6.3.4.15 2.7.1.33]	0.07	1.9E-09	2.3E-09	0.05	
K02124	V/A-type H+/Na+-transporting ATPase subunit K	-0.07	2.0E-09	2.4E-09	-0.18	
K01200	pullulanase [EC:3.2.1.41]	-0.07	2.0E-09	2.4E-09	-0.19	
K00857	thymidine kinase [EC:2.7.1.21]	-0.07	2.1E-09	2.5E-09	-0.08	
K07656	two-component system, OmpR family, sensor histidine kinase TrcS [EC:2.7.13.3]	0.07	2.1E-09	2.5E-09	0.10	
K01079	phosphoserine phosphatase [EC:3.1.3.3]	-0.07	2.2E-09	2.7E-09	-0.16	
K08318	4-hydroxybutyrate dehydrogenase / sulfolactaldehyde 3-reductase [EC:1.1.1.61 1.1.1.373]	0.07	2.4E-09	2.9E-09	0.08	
K06046	long-chain-fatty-acid---luciferin-component ligase [EC:6.2.1.19]	0.07	2.4E-09	2.9E-09	0.10	
K02226	alpha-ribazole phosphatase [EC:3.1.3.73]	-0.07	2.5E-09	3.0E-09	-0.16	
K01586	diaminopimelate decarboxylase [EC:4.1.1.20]	0.07	2.6E-09	3.1E-09	0.06	yes
K02316	DNA primase [EC:2.7.7.101]	0.07	3.0E-09	3.6E-09	0.12	yes
K03895	aerobactin synthase [EC:6.3.2.39]	0.07	3.2E-09	3.9E-09	0.08	
K02115	F-type H+-transporting ATPase subunit gamma	0.07	3.2E-09	3.9E-09	0.12	
K15756	2-hydroxy-6-oxo-6-(2'-aminophenyl)hexa-2,4-dienoate hydrolase [EC:3.7.1.13]	0.07	3.4E-09	4.1E-09	0.07	
K00009	mannitol-1-phosphate 5-dehydrogenase [EC:1.1.1.17]	0.07	3.6E-09	4.3E-09	0.07	
K03737	pyruvate-ferredoxin/flavodoxin oxidoreductase [EC:1.2.7.1 1.2.7.-]	-0.07	3.9E-09	4.7E-09	-0.10	
K14465	succinate semialdehyde reductase (NADPH) [EC:1.1.1.-]	0.07	4.0E-09	4.8E-09	0.06	
K01176	alpha-amylase [EC:3.2.1.1]	0.07	4.5E-09	5.4E-09	0.07	
K07306	anaerobic dimethyl sulfoxide reductase subunit A [EC:1.8.5.3]	0.07	4.6E-09	5.5E-09	0.07	
K07407	alpha-galactosidase [EC:3.2.1.22]	0.07	4.8E-09	5.8E-09	0.11	
K03797	carboxyl-terminal processing protease [EC:3.4.21.102]	-0.07	5.7E-09	6.8E-09	-0.11	
K07256	taurine dehydrogenase large subunit [EC:1.4.2.-]	0.07	6.2E-09	7.4E-09	0.06	
K01590	histidine decarboxylase [EC:4.1.1.22]	-0.07	6.6E-09	7.9E-09	-0.08	
K05711	2,3-dihydroxy-2,3-dihydrophenylpropionate dehydrogenase [EC:1.3.1.87]	0.07	6.6E-09	7.9E-09	0.08	
K01754	threonine dehydratase [EC:4.3.1.19]	0.07	7.2E-09	8.6E-09	0.07	
K00929	butyrate kinase [EC:2.7.2.7]	-0.07	7.6E-09	9.1E-09	-0.14	

Predictor	Description	Pearson's r	P	FDR-adjusted P	Spearman's rho	Associated with fractures (P<0.05)
K09483	3-dehydroshikimate dehydratase [EC:4.2.1.118]	0.07	8.6E-09	1.0E-08	0.05	
K03783	purine-nucleoside phosphorylase [EC:2.4.2.1]	-0.07	9.4E-09	1.1E-08	-0.09	
K14744	prophage endopeptidase [EC:3.4.-.-]	0.07	9.5E-09	1.1E-08	0.08	
K00259	alanine dehydrogenase [EC:1.4.1.1]	-0.07	1.0E-08	1.2E-08	-0.13	
K11785	1,4-dihydroxy-6-naphthoate synthase [EC:1.14.-.-]	0.07	1.0E-08	1.2E-08	0.06	
K11784	cyclic dehypoxanthinyl futalosine synthase [EC:1.21.98.1]	0.07	1.3E-08	1.6E-08	0.06	
K08589	gingipain R [EC:3.4.22.37]	0.07	1.4E-08	1.7E-08	0.14	
K12981	KDO transferase III [EC:2.4.99.-]	0.07	1.4E-08	1.7E-08	0.09	
K02111	F-type H+/Na+-transporting ATPase subunit alpha [EC:7.1.2.2 7.2.2.1]	0.07	1.4E-08	1.7E-08	0.12	
K01935	dethiobiotin synthetase [EC:6.3.3.3]	-0.07	1.5E-08	1.8E-08	-0.18	
K01790	dTDP-4-dehydrorhamnose 3,5-epimerase [EC:5.1.3.13]	-0.07	2.1E-08	2.5E-08	-0.13	
K00798	cob(I)alamin adenosyltransferase [EC:2.5.1.17]	-0.07	2.3E-08	2.7E-08	-0.13	
K04516	chorismate mutase [EC:5.4.99.5]	-0.07	2.4E-08	2.9E-08	-0.13	
K04518	prephenate dehydratase [EC:4.2.1.51]	-0.07	2.6E-08	3.1E-08	-0.11	
K00567	methylated-DNA-[protein]-cysteine S-methyltransferase [EC:2.1.1.63]	-0.07	2.6E-08	3.1E-08	-0.09	
K03894	N2-citryl-N6-acetyl-N6-hydroxylysine synthase [EC:6.3.2.38]	0.07	3.2E-08	3.8E-08	0.08	
K01580	glutamate decarboxylase [EC:4.1.1.15]	-0.07	3.3E-08	3.9E-08	-0.07	
K12292	ATP-binding cassette, subfamily C, bacterial, competence factor transporting protein [EC:3.4.22.-]	0.07	3.5E-08	4.2E-08	0.11	
K00348	Na+-transporting NADH:ubiquinone oxidoreductase subunit C [EC:7.2.1.1]	-0.07	3.6E-08	4.3E-08	-0.13	
K00937	polyphosphate kinase [EC:2.7.4.1]	-0.07	3.9E-08	4.6E-08	-0.21	
K03394	precorrin-2/cobalt-factor-2 C20-methyltransferase [EC:2.1.1.130 2.1.1.151]	-0.07	4.0E-08	4.8E-08	-0.19	
K01911	o-succinylbenzoate---CoA ligase [EC:6.2.1.26]	-0.07	4.0E-08	4.8E-08	-0.18	
K01250	pyrimidine-specific ribonucleoside hydrolase [EC:3.2.-.-]	0.07	4.2E-08	5.0E-08	0.06	
K00831	phosphoserine aminotransferase [EC:2.6.1.52]	0.07	4.2E-08	5.0E-08	0.11	yes
K08255	CoA-disulfide reductase [EC:1.8.1.14]	0.07	4.3E-08	5.1E-08	0.05	
K12551	monofunctional glycosyltransferase [EC:2.4.1.129]	0.07	4.3E-08	5.1E-08	0.05	
K13967	N-acetylmannosamine-6-phosphate 2-epimerase / N-acetylmannosamine kinase [EC:5.1.3.9 2.7.1.60]	0.07	4.3E-08	5.1E-08	0.05	
K02231	adenosylcobinamide kinase / adenosylcobinamide-phosphate guanylyltransferase [EC:2.7.1.156 2.7.7.62]	-0.07	4.4E-08	5.2E-08	-0.18	
K00346	Na+-transporting NADH:ubiquinone oxidoreductase subunit A [EC:7.2.1.1]	-0.07	4.6E-08	5.5E-08	-0.13	
K12999	glucosyltransferase [EC:2.4.1.-]	0.06	4.8E-08	5.7E-08	0.07	
K01355	omptin [EC:3.4.23.49]	0.06	4.9E-08	5.8E-08	0.07	
K01843	lysine 2,3-aminomutase [EC:5.4.3.2]	-0.06	5.0E-08	5.9E-08	-0.13	
K00347	Na+-transporting NADH:ubiquinone oxidoreductase subunit B [EC:7.2.1.1]	-0.06	5.1E-08	6.0E-08	-0.13	
K01548	potassium-transporting ATPase KdpC subunit	-0.06	5.1E-08	6.0E-08	-0.09	
K14583	1,2-dihydroxynaphthalene dioxygenase [EC:1.13.11.56]	0.06	5.1E-08	6.0E-08	0.05	
K14584	2-hydroxychromene-2-carboxylate isomerase [EC:5.99.1.4]	0.06	5.1E-08	6.0E-08	0.05	
K14582	cis-1,2-dihydro-1,2-dihydroxynaphthalene/dibenzothiophene dihydrodiol dehydrogenase [EC:1.3.1.29 1.3.1.60]	0.06	5.1E-08	6.0E-08	0.05	
K14580	naphthalene 1,2-dioxygenase subunit beta [EC:1.14.12.12 1.14.12.23 1.14.12.24]	0.06	5.1E-08	6.0E-08	0.05	
K02224	cobyrinic acid a,c-diamide synthase [EC:6.3.5.9 6.3.5.11]	-0.06	5.4E-08	6.4E-08	-0.16	
K02113	F-type H+-transporting ATPase subunit delta	0.06	5.5E-08	6.5E-08	0.11	
K00349	Na+-transporting NADH:ubiquinone oxidoreductase subunit D [EC:7.2.1.1]	-0.06	6.0E-08	7.1E-08	-0.13	
K00658	2-oxoglutarate dehydrogenase E2 component (dihydrolipoamide succinyltransferase) [EC:2.3.1.61]	0.06	6.2E-08	7.3E-08	0.06	

Predictor	Description	Pearson's r	P	FDR-adjusted P	Spearman's rho	Associated with fractures (P<0.05)
K03564	thioredoxin-dependent peroxiredoxin [EC:1.11.1.24]	-0.06	7.0E-08	8.3E-08	-0.08	
K02114	F-type H+-transporting ATPase subunit epsilon	0.06	7.5E-08	8.8E-08	0.11	
K01665	para-aminobenzoate synthetase component I [EC:2.6.1.85]	-0.06	8.1E-08	9.5E-08	-0.18	
K14982	two-component system, OmpR family, sensor histidine kinase CiaH [EC:2.7.13.3]	0.06	8.4E-08	9.9E-08	0.06	
K03276	UDP-glucose/galactose:(glucosyl)LPS alpha-1,2-glucosyl/galactosyltransferase [EC:2.4.1.-]	0.06	9.5E-08	1.1E-07	0.07	
K00590	site-specific DNA-methyltransferase (cytosine-N4-specific) [EC:2.1.1.113]	-0.06	9.7E-08	1.1E-07	-0.07	
K06180	23S rRNA pseudouridine1911/1915/1917 synthase [EC:5.4.99.23]	0.06	9.8E-08	1.2E-07	0.12	
K00768	nicotinate-nucleotide--dimethylbenzimidazole phosphoribosyltransferase [EC:2.4.2.21]	-0.06	1.0E-07	1.2E-07	-0.14	
K00932	propionate kinase [EC:2.7.2.15]	0.06	1.0E-07	1.2E-07	0.07	
K05708	3-phenylpropionate/trans-cinnamate dioxygenase subunit alpha [EC:1.14.12.19]	0.06	1.0E-07	1.2E-07	0.07	
K02188	cobalt-precorrin-5B (C1)-methyltransferase [EC:2.1.1.195]	-0.06	1.0E-07	1.2E-07	-0.16	
K01805	xylose isomerase [EC:5.3.1.5]	-0.06	1.1E-07	1.3E-07	-0.17	
K00571	site-specific DNA-methyltransferase (adenine-specific) [EC:2.1.1.72]	0.06	1.1E-07	1.3E-07	0.07	
K07646	two-component system, OmpR family, sensor histidine kinase KdpD [EC:2.7.13.3]	-0.06	1.1E-07	1.3E-07	-0.08	
K00683	glutaminyl-peptide cyclotransferase [EC:2.3.2.5]	0.06	1.1E-07	1.3E-07	0.10	
K00970	poly(A) polymerase [EC:2.7.7.19]	0.06	1.3E-07	1.5E-07	0.06	
K01216	licheninase [EC:3.2.1.73]	-0.06	1.4E-07	1.6E-07	-0.07	
K05709	3-phenylpropionate/trans-cinnamate dioxygenase subunit beta [EC:1.14.12.19]	0.06	1.4E-07	1.6E-07	0.07	
K00177	2-oxoglutarate ferredoxin oxidoreductase subunit gamma [EC:1.2.7.3]	-0.06	1.4E-07	1.6E-07	-0.11	
K00254	dihydroorotate dehydrogenase [EC:1.3.5.2]	0.06	1.9E-07	2.2E-07	0.06	
K00545	catechol O-methyltransferase [EC:2.1.1.6]	0.06	2.0E-07	2.3E-07	0.08	
K14652	3,4-dihydroxy 2-butanone 4-phosphate synthase / GTP cyclohydrolase II [EC:4.1.99.12 3.5.4.25]	-0.06	2.2E-07	2.6E-07	-0.12	
K14441	ribosomal protein S12 methylthiotransferase [EC:2.8.4.4]	-0.06	2.2E-07	2.6E-07	-0.08	
K13085	phosphatidylinositol-4,5-bisphosphate 4-phosphatase [EC:3.1.3.78]	0.06	2.2E-07	2.6E-07	0.08	
K15036	acetyl-CoA/propionyl-CoA carboxylase [EC:6.4.1.2 6.4.1.3 2.1.3.15]	0.06	2.3E-07	2.7E-07	0.06	
K01534	Zn2+/Cd2+-exporting ATPase [EC:7.2.2.12 7.2.2.21]	-0.06	2.4E-07	2.8E-07	-0.16	
K00625	phosphate acetyltransferase [EC:2.3.1.8]	-0.06	2.4E-07	2.8E-07	-0.14	
K00721	dolichol-phosphate mannosyltransferase [EC:2.4.1.83]	-0.06	2.6E-07	3.0E-07	-0.12	
K07698	two-component system, sporulation sensor kinase C [EC:2.7.13.3]	0.06	2.8E-07	3.3E-07	0.13	
K15017	malonyl-CoA/succinyl-CoA reductase (NADPH) [EC:1.2.1.75 1.2.1.76]	0.06	2.8E-07	3.3E-07	0.05	
K00227	Delta7-sterol 5-desaturase [EC:1.14.19.20]	0.06	3.1E-07	3.6E-07	0.06	
K11640	two-component system, LytTR family, sensor histidine kinase NatK [EC:2.7.13.3]	0.06	3.5E-07	4.1E-07	0.04	
K00266	glutamate synthase (NADPH) small chain [EC:1.4.1.13]	-0.06	4.0E-07	4.7E-07	-0.16	yes
K03896	acetyl CoA:N6-hydroxylysine acetyl transferase [EC:2.3.1.102]	0.06	4.3E-07	5.0E-07	0.07	
K02232	adenosylcobyric acid synthase [EC:6.3.5.10]	-0.06	4.3E-07	5.0E-07	-0.16	
K13037	L-alanine-L-anticapsin ligase [EC:6.3.2.49]	0.06	4.4E-07	5.1E-07	0.05	
K02756	NA	0.06	4.6E-07	5.3E-07	0.06	
K01185	lysozyme [EC:3.2.1.17]	0.06	4.7E-07	5.5E-07	0.06	
K01447	N-acetylmuramoyl-L-alanine amidase [EC:3.5.1.28]	-0.06	4.8E-07	5.6E-07	-0.15	
K01918	pantoate--beta-alanine ligase [EC:6.3.2.1]	-0.06	4.9E-07	5.7E-07	-0.04	
K00647	3-oxoacyl-[acyl-carrier-protein] synthase I [EC:2.3.1.41]	-0.06	5.3E-07	6.2E-07	-0.18	
K01821	4-oxalocrotonate tautomerase [EC:5.3.2.6]	0.06	5.6E-07	6.5E-07	0.06	

Predictor	Description	Pearson's r	P	FDR-adjusted P	Spearman's rho	Associated with fractures (P<0.05)
K00560	thymidylate synthase [EC:2.1.1.45]	-0.06	5.6E-07	6.5E-07	-0.08	
K03275	UDP-glucose:(glucosyl)LPS alpha-1,3-glucosyltransferase [EC:2.4.1.-]	0.06	5.8E-07	6.7E-07	0.07	
K00931	glutamate 5-kinase [EC:2.7.2.11]	0.06	5.9E-07	6.8E-07	0.08	
K01579	aspartate 1-decarboxylase [EC:4.1.1.11]	-0.06	6.0E-07	7.0E-07	-0.04	
K00050	NA	0.06	6.4E-07	7.4E-07	0.08	
K01740	O-acetylhomoserine (thiol)-lyase [EC:2.5.1.49]	-0.06	6.4E-07	7.4E-07	-0.14	
K12977	lipid A 1-phosphatase [EC:3.1.3.-]	0.06	6.7E-07	7.8E-07	0.04	
K00275	pyridoxamine 5'-phosphate oxidase [EC:1.4.3.5]	0.06	7.1E-07	8.2E-07	0.06	
K13244	c-di-GMP-specific phosphodiesterase [EC:3.1.4.52]	0.06	7.3E-07	8.4E-07	0.07	
K05887	quinate/shikimate dehydrogenase [EC:1.1.1.282]	0.06	7.3E-07	8.4E-07	0.07	
K01337	lysyl endopeptidase [EC:3.4.21.50]	0.06	8.2E-07	9.5E-07	0.07	
K01938	formate--tetrahydrofolate ligase [EC:6.3.4.3]	-0.06	9.5E-07	1.1E-06	-0.14	
K07154	serine/threonine-protein kinase HipA [EC:2.7.11.1]	-0.06	1.2E-06	1.4E-06	-0.07	
K15853	acyl transferase [EC:2.3.1.-]	0.06	1.3E-06	1.5E-06	0.06	
K00210	NA	-0.06	1.5E-06	1.7E-06	-0.06	
K04844	hypothetical glycosyl hydrolase [EC:3.2.1.-]	0.06	1.5E-06	1.7E-06	0.07	
K07314	serine/threonine protein phosphatase 2 [EC:3.1.3.16]	0.06	1.5E-06	1.7E-06	0.07	
K15634	2,3-bisphosphoglycerate-dependent phosphoglycerate mutase [EC:5.4.2.11]	0.06	1.5E-06	1.7E-06	0.07	
K01481	protein-arginine deiminase [EC:3.5.3.15]	0.06	1.6E-06	1.8E-06	0.05	
K02428	XTP/dITP diphosphohydrolase [EC:3.6.1.66]	-0.06	1.8E-06	2.1E-06	-0.08	
K03813	molybdenum transport protein [EC:2.4.2.-]	0.06	1.9E-06	2.2E-06	0.07	
K03648	uracil-DNA glycosylase [EC:3.2.2.27]	0.06	2.0E-06	2.3E-06	0.11	yes
K01714	4-hydroxy-tetrahydrodipicolinate synthase [EC:4.3.3.7]	-0.06	2.2E-06	2.5E-06	-0.14	
K00034	glucose 1-dehydrogenase [EC:1.1.1.47]	0.06	2.2E-06	2.5E-06	0.08	
K05369	15,16-dihydrobiliverdin:ferredoxin oxidoreductase [EC:1.3.7.2]	0.06	2.4E-06	2.7E-06	0.04	
K05356	all-trans-nonaprenyl-diphosphate synthase [EC:2.5.1.84 2.5.1.85]	0.06	2.4E-06	2.7E-06	0.04	
K15226	arogenate dehydrogenase (NADP+) [EC:1.3.1.78]	0.06	2.4E-06	2.7E-06	0.04	
K14331	fatty aldehyde decarbonylase [EC:4.1.99.5]	0.06	2.4E-06	2.7E-06	0.04	
K14330	fatty aldehyde-generating acyl-ACP reductase [EC:1.2.1.80]	0.06	2.4E-06	2.7E-06	0.04	
K05572	NAD(P)H-quinone oxidoreductase subunit 1 [EC:7.1.1.2]	0.06	2.4E-06	2.7E-06	0.04	
K05585	NAD(P)H-quinone oxidoreductase subunit N [EC:7.1.1.2]	0.06	2.4E-06	2.7E-06	0.04	
K02288	phycocyanobilin lyase subunit alpha [EC:4.4.1.32]	0.06	2.4E-06	2.7E-06	0.04	
K05370	phycoerythrobilin:ferredoxin oxidoreductase [EC:1.3.7.3]	0.06	2.4E-06	2.7E-06	0.04	
K11520	two-component system, OmpR family, manganese sensing sensor histidine kinase [EC:2.7.13.3]	0.06	2.4E-06	2.7E-06	0.04	
K03867	UDP-glucose:tetrahydrobiopterin glucosyltransferase [EC:2.4.1.-]	0.06	2.4E-06	2.7E-06	0.04	
K13381	bifunctional chitinase/lysozyme [EC:3.2.1.14 3.2.1.17]	0.06	2.6E-06	3.0E-06	0.07	
K01771	1-phosphatidylinositol phosphodiesterase [EC:4.6.1.13]	-0.06	2.9E-06	3.3E-06	-0.06	
K11194	fructose PTS system EIIA component [EC:2.7.1.202]	0.06	3.0E-06	3.4E-06	0.05	
K11195	fructose PTS system EIIB component [EC:2.7.1.202]	0.06	3.0E-06	3.4E-06	0.05	
K13789	geranylgeranyl diphosphate synthase, type II [EC:2.5.1.1 2.5.1.10 2.5.1.29]	-0.06	3.0E-06	3.4E-06	-0.12	
K07405	alpha-amylase [EC:3.2.1.1]	-0.06	3.2E-06	3.7E-06	-0.18	
K00973	glucose-1-phosphate thymidylyltransferase [EC:2.7.7.24]	-0.06	3.3E-06	3.8E-06	-0.04	

Predictor	Description	Pearson's r	P	FDR-adjusted P	Spearman's rho	Associated with fractures (P<0.05)
K00240	succinate dehydrogenase / fumarate reductase, iron-sulfur subunit [EC:1.3.5.1 1.3.5.4]	0.06	3.4E-06	3.9E-06	0.11	
K01520	dUTP pyrophosphatase [EC:3.6.1.23]	-0.06	3.5E-06	4.0E-06	-0.12	
K03550	holliday junction DNA helicase RuvA [EC:3.6.4.12]	0.06	3.5E-06	4.0E-06	0.10	yes
K00764	amidophosphoribosyltransferase [EC:2.4.2.14]	-0.06	3.6E-06	4.1E-06	-0.14	yes
K13001	mannosyltransferase [EC:2.4.1.-]	0.06	3.8E-06	4.3E-06	0.10	
K00215	4-hydroxy-tetrahydrodipicolinate reductase [EC:1.17.1.8]	0.06	3.8E-06	4.3E-06	0.13	
K01966	propionyl-CoA carboxylase beta chain [EC:6.4.1.3 2.1.3.15]	0.06	3.8E-06	4.3E-06	0.05	
K03427	type I restriction enzyme M protein [EC:2.1.1.72]	-0.05	3.9E-06	4.4E-06	-0.12	
K01802	peptidylprolyl isomerase [EC:5.2.1.8]	-0.05	4.9E-06	5.6E-06	-0.17	
K05349	beta-glucosidase [EC:3.2.1.21]	-0.05	5.0E-06	5.7E-06	-0.09	yes
K05977	2'-hydroxybiphenyl-2-sulfinate desulfinase [EC:3.13.1.3]	0.05	5.2E-06	5.9E-06	0.04	
K02117	V/A-type H+/Na+-transporting ATPase subunit A [EC:7.1.2.2 7.2.2.1]	-0.05	5.4E-06	6.1E-06	-0.12	
K06041	arabinose-5-phosphate isomerase [EC:5.3.1.13]	-0.05	5.4E-06	6.1E-06	-0.07	
K00185	dimethyl sulfoxide reductase membrane subunit	0.05	5.9E-06	6.7E-06	0.07	
K01443	N-acetylglucosamine-6-phosphate deacetylase [EC:3.5.1.25]	-0.05	6.1E-06	6.9E-06	-0.10	
K00176	2-oxoglutarate ferredoxin oxidoreductase subunit delta [EC:1.2.7.3]	-0.05	6.2E-06	7.0E-06	-0.10	
K02567	nitrate reductase (cytochrome) [EC:1.9.6.1]	0.05	6.3E-06	7.1E-06	0.06	
K02080	putative deaminase/isomerase [EC:3.5.99.-]	0.05	6.4E-06	7.3E-06	0.07	
K00219	2,4-dienoyl-CoA reductase (NADPH2) [EC:1.3.1.34]	0.05	6.5E-06	7.4E-06	0.06	
K16153	glycogen phosphorylase/synthase [EC:2.4.1.1 2.4.1.11]	0.05	6.5E-06	7.4E-06	0.05	
K01814	phosphoribosylformimino-5-aminoimidazole carboxamide ribotide isomerase [EC:5.3.1.16]	0.05	6.5E-06	7.4E-06	0.12	
K00761	uracil phosphoribosyltransferase [EC:2.4.2.9]	-0.05	7.4E-06	8.4E-06	-0.16	yes
K11023	pertussis toxin subunit 1 [EC:2.4.2.-]	0.05	8.3E-06	9.4E-06	0.08	
K00876	uridine kinase [EC:2.7.1.48]	-0.05	8.5E-06	9.6E-06	-0.06	
K02121	V/A-type H+/Na+-transporting ATPase subunit E	-0.05	9.1E-06	1.0E-05	-0.12	
K02484	two-component system, OmpR family, sensor kinase [EC:2.7.13.3]	-0.05	9.5E-06	1.1E-05	-0.17	
K00048	lactaldehyde reductase [EC:1.1.1.77]	-0.05	9.6E-06	1.1E-05	-0.14	
K00928	aspartate kinase [EC:2.7.2.4]	0.05	1.1E-05	1.2E-05	0.12	
K03525	type III pantothenate kinase [EC:2.7.1.33]	0.05	1.1E-05	1.2E-05	0.09	
K02217	ferritin [EC:1.16.3.2]	-0.05	1.2E-05	1.4E-05	-0.13	
K01482	dimethylargininase [EC:3.5.3.18]	0.05	1.2E-05	1.4E-05	0.06	
K00059	3-oxoacyl-[acyl-carrier protein] reductase [EC:1.1.1.100]	0.05	1.2E-05	1.4E-05	0.04	yes
K01568	pyruvate decarboxylase [EC:4.1.1.1]	0.05	1.2E-05	1.4E-05	0.03	
K00654	serine palmitoyltransferase [EC:2.3.1.50]	0.05	1.2E-05	1.4E-05	0.03	
K06033	arylmalonate decarboxylase [EC:4.1.1.76]	0.05	1.2E-05	1.4E-05	0.04	
K00925	acetate kinase [EC:2.7.2.1]	0.05	1.3E-05	1.5E-05	0.10	yes
K00244	fumarate reductase flavoprotein subunit [EC:1.3.5.4]	0.05	1.3E-05	1.5E-05	0.05	
K11752	diaminohydroxyphosphoribosylaminopyrimidine deaminase / 5-amino-6-(5-phosphoribosylamino)uracil reductase [EC:3.5.4.26 1.1.1.193]	-0.05	1.4E-05	1.6E-05	-0.14	
K12297	23S rRNA (guanine2069-N7)-methyltransferase / 23S rRNA (guanine2445-N2)-methyltransferase [EC:2.1.1.264 2.1.1.173]	0.05	1.4E-05	1.6E-05	0.06	
K00818	acetylornithine aminotransferase [EC:2.6.1.11]	-0.05	1.4E-05	1.6E-05	-0.06	
K10814	hydrogen cyanide synthase HcnA [EC:1.4.99.5]	0.05	1.5E-05	1.7E-05	0.10	
K05579	NAD(P)H-quinone oxidoreductase subunit H [EC:7.1.1.2]	0.05	1.5E-05	1.7E-05	0.04	

Predictor	Description	Pearson's r	P	FDR-adjusted P	Spearman's rho	Associated with fractures (P<0.05)
K05581	NAD(P)H-quinone oxidoreductase subunit J [EC:7.1.1.2]	0.05	1.5E-05	1.7E-05	0.04	
K05582	NAD(P)H-quinone oxidoreductase subunit K [EC:7.1.1.2]	0.05	1.5E-05	1.7E-05	0.04	
K01703	3-isopropylmalate/(R)-2-methylmalate dehydratase large subunit [EC:4.2.1.33 4.2.1.35]	0.05	1.6E-05	1.8E-05	0.08	
K00865	glycerate 2-kinase [EC:2.7.1.165]	0.05	1.6E-05	1.8E-05	0.00	
K00286	pyrroline-5-carboxylate reductase [EC:1.5.1.2]	-0.05	1.7E-05	1.9E-05	-0.10	
K06001	tryptophan synthase beta chain [EC:4.2.1.20]	-0.05	1.7E-05	1.9E-05	-0.02	
K01187	alpha-glucosidase [EC:3.2.1.20]	-0.05	1.8E-05	2.0E-05	-0.11	
K01627	2-dehydro-3-deoxyphosphooctonate aldolase (KDO 8-P synthase) [EC:2.5.1.55]	-0.05	2.2E-05	2.5E-05	-0.07	
K10353	deoxyadenosine kinase [EC:2.7.1.76]	0.05	2.5E-05	2.8E-05	0.08	
K12986	1,5-rhamnosyltransferase [EC:2.4.1.-]	0.05	2.5E-05	2.8E-05	0.07	
K14468	malonyl-CoA reductase / 3-hydroxypropionate dehydrogenase (NADP+) [EC:1.2.1.75 1.1.1.298]	0.05	2.6E-05	2.9E-05	0.04	
K15052	propionyl-CoA carboxylase [EC:6.4.1.3 2.1.3.15]	0.05	2.6E-05	2.9E-05	0.04	
K14472	succinyl-CoA:(S)-malate CoA-transferase subunit B [EC:2.8.3.22]	0.05	2.6E-05	2.9E-05	0.04	
K01710	dTDP-glucose 4,6-dehydratase [EC:4.2.1.46]	0.05	2.7E-05	3.0E-05	0.06	
K03426	NAD+ diphosphatase [EC:3.6.1.22]	-0.05	2.8E-05	3.1E-05	-0.13	
K01585	arginine decarboxylase [EC:4.1.1.19]	-0.05	2.8E-05	3.1E-05	-0.07	
K00382	dihydrolipoamide dehydrogenase [EC:1.8.1.4]	0.05	2.9E-05	3.2E-05	0.10	
K01130	arylsulfatase [EC:3.1.6.1]	0.05	3.3E-05	3.7E-05	0.04	
K01847	methylmalonyl-CoA mutase [EC:5.4.99.2]	-0.05	3.5E-05	3.9E-05	-0.07	
K03644	lipoyl synthase [EC:2.8.1.8]	-0.05	3.5E-05	3.9E-05	-0.06	
K00794	6,7-dimethyl-8-ribityllumazine synthase [EC:2.5.1.78]	-0.05	3.7E-05	4.1E-05	0.02	
K01759	lactoylglutathione lyase [EC:4.4.1.5]	-0.05	3.9E-05	4.3E-05	-0.04	
K03386	peroxiredoxin 2/4 [EC:1.11.1.24]	-0.05	3.9E-05	4.3E-05	-0.01	
K12466	(+)-trans-carveol dehydrogenase [EC:1.1.1.275]	0.05	4.0E-05	4.5E-05	0.08	
K06178	23S rRNA pseudouridine2605 synthase [EC:5.4.99.22]	0.05	4.1E-05	4.6E-05	0.08	yes
K00651	homoserine O-succinyltransferase/O-acetyltransferase [EC:2.3.1.46 2.3.1.31]	-0.05	4.3E-05	4.8E-05	-0.11	
K01676	fumarate hydratase, class I [EC:4.2.1.2]	-0.05	4.4E-05	4.9E-05	-0.06	
K00330	NADH-quinone oxidoreductase subunit A [EC:7.1.1.2]	-0.05	4.6E-05	5.1E-05	-0.06	
K03770	peptidyl-prolyl cis-trans isomerase D [EC:5.2.1.8]	-0.05	4.8E-05	5.3E-05	-0.06	
K01537	P-type Ca2+ transporter type 2C [EC:7.2.2.10]	0.05	5.3E-05	5.9E-05	0.13	
K00245	fumarate reductase iron-sulfur subunit [EC:1.3.5.4]	0.05	5.6E-05	6.2E-05	0.05	
K00183	NA	-0.05	6.3E-05	7.0E-05	-0.04	
K02123	V/A-type H+/Na+-transporting ATPase subunit I	-0.05	6.6E-05	7.3E-05	-0.12	
K00891	shikimate kinase [EC:2.7.1.71]	-0.05	6.6E-05	7.3E-05	-0.06	
K00605	aminomethyltransferase [EC:2.1.2.10]	-0.05	6.8E-05	7.5E-05	-0.05	
K02203	phosphoserine / homoserine phosphotransferase [EC:3.1.3.3 2.7.1.39]	0.05	6.9E-05	7.7E-05	0.05	
K00957	sulfate adenylyltransferase subunit 2 [EC:2.7.7.4]	-0.05	7.0E-05	7.8E-05	-0.15	
K00767	nicotinate-nucleotide pyrophosphorylase (carboxylating) [EC:2.4.2.19]	0.05	7.1E-05	7.9E-05	0.11	
K01809	mannose-6-phosphate isomerase [EC:5.3.1.8]	-0.05	7.1E-05	7.9E-05	-0.03	
K00854	xylulokinase [EC:2.7.1.17]	-0.05	7.1E-05	7.9E-05	-0.10	
K02495	oxygen-independent coproporphyrinogen III oxidase [EC:1.3.98.3]	0.05	7.4E-05	8.2E-05	0.11	
K00648	3-oxoacyl-[acyl-carrier-protein] synthase III [EC:2.3.1.180]	-0.05	7.6E-05	8.4E-05	0.00	

Predictor	Description	Pearson's r	P	FDR-adjusted P	Spearman's rho	Associated with fractures (P<0.05)
K07173	S-ribosylhomocysteine lyase [EC:4.4.1.21]	0.05	8.3E-05	9.2E-05	-0.01	
K00331	NADH-quinone oxidoreductase subunit B [EC:7.1.1.2]	-0.05	8.7E-05	9.6E-05	-0.06	
K10150	cysteine synthase / O-phosphoserine sulfhydrylase / cystathionine beta-synthase [EC:2.5.1.47 2.5.1.65 4.2.1.22]	0.05	9.5E-05	1.1E-04	0.03	
K11991	tRNA(adenine34) deaminase [EC:3.5.4.33]	-0.05	9.9E-05	1.1E-04	-0.05	
K06019	pyrophosphatase PpaX [EC:3.6.1.1]	0.05	1.0E-04	1.1E-04	0.06	
K02233	adenosylcobinamide-GDP ribazoletransferase [EC:2.7.8.26]	-0.05	1.0E-04	1.1E-04	-0.11	
K13246	c-di-GMP phosphodiesterase [EC:3.1.4.52]	0.05	1.2E-04	1.3E-04	0.04	
K02812	sorbose PTS system EIIA component [EC:2.7.1.206]	0.05	1.2E-04	1.3E-04	0.06	
K02813	sorbose PTS system EIIB component [EC:2.7.1.206]	0.05	1.2E-04	1.3E-04	0.06	
K01868	threonyl-tRNA synthetase [EC:6.1.1.3]	-0.05	1.4E-04	1.5E-04	-0.08	
K01972	DNA ligase (NAD+) [EC:6.5.1.2]	-0.05	1.5E-04	1.7E-04	-0.04	
K00845	glucokinase [EC:2.7.1.2]	0.05	1.6E-04	1.8E-04	0.09	
K00341	NADH-quinone oxidoreductase subunit L [EC:7.1.1.2]	-0.04	1.7E-04	1.9E-04	-0.05	
K02687	ribosomal protein L11 methyltransferase [EC:2.1.1.-]	-0.04	1.7E-04	1.9E-04	-0.07	
K01804	L-arabinose isomerase [EC:5.3.1.4]	0.04	1.8E-04	2.0E-04	-0.02	
K01731	pectate disaccharide-lyase [EC:4.2.2.9]	0.04	2.0E-04	2.2E-04	0.05	
K00339	NADH-quinone oxidoreductase subunit J [EC:7.1.1.2]	-0.04	2.1E-04	2.3E-04	-0.05	
K01808	ribose 5-phosphate isomerase B [EC:5.3.1.6]	-0.04	2.1E-04	2.3E-04	-0.04	
K02109	F-type H+-transporting ATPase subunit b	0.04	2.2E-04	2.4E-04	0.07	
K01619	deoxyribose-phosphate aldolase [EC:4.1.2.4]	-0.04	2.2E-04	2.4E-04	-0.04	
K01077	alkaline phosphatase [EC:3.1.3.1]	-0.04	2.2E-04	2.4E-04	-0.08	
K00337	NADH-quinone oxidoreductase subunit H [EC:7.1.1.2]	-0.04	2.3E-04	2.5E-04	-0.05	
K00912	tetraacyldisaccharide 4'-kinase [EC:2.7.1.130]	-0.04	2.4E-04	2.6E-04	-0.06	
K01592	tyrosine decarboxylase [EC:4.1.1.25]	0.04	2.4E-04	2.6E-04	0.03	
K06153	undecaprenyl-diphosphatase [EC:3.6.1.27]	0.04	3.1E-04	3.4E-04	0.07	
K05366	penicillin-binding protein 1A [EC:2.4.1.129 3.4.16.4]	0.04	3.5E-04	3.8E-04	0.12	
K00941	hydroxymethylpyrimidine/phosphomethylpyrimidine kinase [EC:2.7.1.49 2.7.4.7]	-0.04	3.8E-04	4.2E-04	-0.11	
K03279	UDP-glucose:(galactosyl)LPS alpha-1,2-glucosyltransferase [EC:2.4.1.58]	0.04	4.0E-04	4.4E-04	0.06	
K01389	neprilysin [EC:3.4.24.11]	-0.04	4.0E-04	4.4E-04	-0.05	
K00759	adenine phosphoribosyltransferase [EC:2.4.2.7]	-0.04	4.3E-04	4.7E-04	-0.12	
K03941	NADH dehydrogenase (ubiquinone) Fe-S protein 8 [EC:7.1.1.2]	0.04	4.4E-04	4.8E-04	0.03	
K02108	F-type H+-transporting ATPase subunit a	0.04	5.0E-04	5.5E-04	0.07	
K01129	dGTPase [EC:3.1.5.1]	-0.04	5.1E-04	5.6E-04	-0.09	
K01817	phosphoribosylanthranilate isomerase [EC:5.3.1.24]	0.04	5.2E-04	5.7E-04	0.06	
K14415	tRNA-splicing ligase RtcB (3'-phosphate/5'-hydroxy nucleic acid ligase) [EC:6.5.1.8]	0.04	5.4E-04	5.9E-04	0.05	
K06970	23S rRNA (adenine1618-N6)-methyltransferase [EC:2.1.1.181]	-0.04	5.4E-04	5.9E-04	-0.04	yes
K00784	ribonuclease Z [EC:3.1.26.11]	-0.04	5.5E-04	6.0E-04	-0.06	
K01493	dCMP deaminase [EC:3.5.4.12]	-0.04	5.8E-04	6.3E-04	-0.05	
K03439	tRNA (guanine-N7-)-methyltransferase [EC:2.1.1.33]	0.04	5.8E-04	6.3E-04	0.16	
K00494	alkanal monooxygenase alpha chain [EC:1.14.14.3]	0.04	6.3E-04	6.9E-04	0.03	
K15854	alkanal monooxygenase beta chain [EC:1.14.14.3]	0.04	6.3E-04	6.9E-04	0.03	
K03400	long-chain-fatty-acyl-CoA reductase [EC:1.2.1.50]	0.04	6.3E-04	6.9E-04	0.03	

Predictor	Description	Pearson's r	P	FDR-adjusted P	Spearman's rho	Associated with fractures (P<0.05)
K00075	UDP-N-acetylmuramate dehydrogenase [EC:1.3.1.98]	-0.04	6.5E-04	7.1E-04	-0.04	
K00677	UDP-N-acetylglucosamine acyltransferase [EC:2.3.1.129]	-0.04	6.7E-04	7.3E-04	-0.02	
K01092	myo-inositol-1(or 4)-monophosphatase [EC:3.1.3.25]	-0.04	6.8E-04	7.4E-04	-0.16	
K01696	tryptophan synthase beta chain [EC:4.2.1.20]	0.04	7.0E-04	7.6E-04	0.07	
K00013	histidinol dehydrogenase [EC:1.1.1.23]	0.04	7.2E-04	7.8E-04	0.11	
K01629	rhamnulose-1-phosphate aldolase [EC:4.1.2.19]	-0.04	7.5E-04	8.2E-04	-0.17	
K00602	phosphoribosylaminoimidazolecarboxamide formyltransferase / IMP cyclohydrolase [EC:2.1.2.3 3.5.4.10]	-0.04	8.2E-04	8.9E-04	-0.04	
K00850	6-phosphofructokinase 1 [EC:2.7.1.11]	-0.04	8.5E-04	9.2E-04	-0.09	
K11173	hydroxyacid-oxoacid transhydrogenase [EC:1.1.99.24]	0.04	8.9E-04	9.7E-04	0.07	
K00223	Delta24(24(1))-sterol reductase [EC:1.3.1.71]	0.04	9.2E-04	1.0E-03	0.04	
K00766	anthranilate phosphoribosyltransferase [EC:2.4.2.18]	-0.04	9.3E-04	1.0E-03	-0.12	
K02227	adenosylcobinamide-phosphate synthase [EC:6.3.1.10]	-0.04	9.9E-04	1.1E-03	-0.12	
K03517	quinolinate synthase [EC:2.5.1.72]	0.04	1.2E-03	1.3E-03	0.11	
K03685	ribonuclease III [EC:3.1.26.3]	-0.04	1.2E-03	1.3E-03	-0.05	
K01787	N-acylglucosamine 2-epimerase [EC:5.1.3.8]	-0.04	1.2E-03	1.3E-03	-0.06	
K02755	beta-glucoside PTS system EIIA component [EC:2.7.1.-]	0.04	1.3E-03	1.4E-03	0.03	
K04342	streptomycin-6-phosphatase [EC:3.1.3.39]	0.04	1.4E-03	1.5E-03	0.05	
K10620	2,3-dihydroxy-2,3-dihydro-p-cumate dehydrogenase [EC:1.3.1.58]	0.04	1.5E-03	1.6E-03	0.01	
K00689	dextransucrase [EC:2.4.1.5]	0.04	1.5E-03	1.6E-03	0.07	
K07309	Tat-targeted selenate reductase subunit YnfE [EC:1.97.1.9]	0.04	1.6E-03	1.7E-03	0.04	
K03332	fructan beta-fructosidase [EC:3.2.1.80]	-0.04	1.7E-03	1.8E-03	-0.04	
K00615	transketolase [EC:2.2.1.1]	-0.04	1.9E-03	2.1E-03	-0.13	
K11949	4-(2-carboxyphenyl)-2-oxobut-3-enoate aldolase [EC:4.1.2.34]	-0.04	2.2E-03	2.4E-03	-0.05	
K16055	trehalose 6-phosphate synthase/phosphatase [EC:2.4.1.15 3.1.3.12]	-0.04	2.2E-03	2.4E-03	-0.18	
K00387	sulfite oxidase [EC:1.8.3.1]	0.04	2.2E-03	2.4E-03	0.02	
K00633	galactoside O-acetyltransferase [EC:2.3.1.18]	-0.04	2.2E-03	2.4E-03	-0.04	yes
K00527	ribonucleoside-triphosphate reductase (thioredoxin) [EC:1.17.4.2]	0.04	2.3E-03	2.5E-03	-0.03	yes
K00713	UDP-glucose:(glucosyl)LPS alpha-1,2-glucosyltransferase [EC:2.4.1.-]	0.04	2.3E-03	2.5E-03	0.06	
K12985	(galactosyl)LPS 1,2-glucosyltransferase [EC:2.4.1.-]	0.04	2.4E-03	2.6E-03	0.06	
K12983	UDP-glucose:(glucosyl)LPS beta-1,3-glucosyltransferase [EC:2.4.1.-]	0.04	2.4E-03	2.6E-03	0.06	
K06269	serine/threonine-protein phosphatase PP1 catalytic subunit [EC:3.1.3.16]	0.04	2.5E-03	2.7E-03	0.03	
K01280	tripeptidyl-peptidase II [EC:3.4.14.10]	-0.04	2.6E-03	2.8E-03	-0.05	
K03601	exodeoxyribonuclease VII large subunit [EC:3.1.11.6]	0.04	2.6E-03	2.8E-03	0.03	
K04069	pyruvate formate lyase activating enzyme [EC:1.97.1.4]	-0.04	2.6E-03	2.8E-03	-0.13	
K02619	4-amino-4-deoxychorismate lyase [EC:4.1.3.38]	-0.04	2.8E-03	3.0E-03	-0.14	
K10908	DNA-directed RNA polymerase, mitochondrial [EC:2.7.7.6]	0.04	3.1E-03	3.3E-03	0.03	
K01652	acetolactate synthase I/II/III large subunit [EC:2.2.1.6]	0.04	3.1E-03	3.3E-03	0.09	
K02342	DNA polymerase III subunit epsilon [EC:2.7.7.7]	-0.04	3.1E-03	3.3E-03	-0.03	
K02528	16S rRNA (adenine1518-N6/adenine1519-N6)-dimethyltransferase [EC:2.1.1.182]	0.03	3.3E-03	3.5E-03	0.03	
K02852	UDP-N-acetyl-D-mannosaminouronate:lipid I N-acetyl-D-mannosaminouronosyltransferase [EC:2.4.1.180]	-0.03	3.5E-03	3.8E-03	-0.18	
K01858	myo-inositol-1-phosphate synthase [EC:5.5.1.4]	0.03	3.5E-03	3.8E-03	-0.10	
K00703	starch synthase [EC:2.4.1.21]	-0.03	4.0E-03	4.3E-03	-0.02	

Predictor	Description	Pearson's r	P	FDR-adjusted P	Spearman's rho	Associated with fractures (P<0.05)
K00145	N-acetyl-gamma-glutamyl-phosphate reductase [EC:1.2.1.38]	0.03	4.2E-03	4.5E-03	0.10	
K00180	indolepyruvate ferredoxin oxidoreductase, beta subunit [EC:1.2.7.8]	-0.03	4.2E-03	4.5E-03	-0.05	
K08298	L-carnitine CoA-transferase [EC:2.8.3.21]	0.03	4.3E-03	4.6E-03	0.04	
K03365	cytosine/creatinine deaminase [EC:3.5.4.1 3.5.4.21]	0.03	4.4E-03	4.7E-03	0.01	
K03816	xanthine phosphoribosyltransferase [EC:2.4.2.22]	-0.03	4.5E-03	4.8E-03	-0.11	
K03215	23S rRNA (uracil1939-C5)-methyltransferase [EC:2.1.1.190]	0.03	4.5E-03	4.8E-03	0.10	
K01209	alpha-L-arabinofuranosidase [EC:3.2.1.55]	-0.03	4.7E-03	5.0E-03	-0.13	yes
K09565	peptidyl-prolyl isomerase F (cyclophilin D) [EC:5.2.1.8]	0.03	4.9E-03	5.2E-03	0.03	
K00954	pantetheine-phosphate adenylyltransferase [EC:2.7.7.3]	0.03	4.9E-03	5.2E-03	0.04	yes
K01201	glucosylceramidase [EC:3.2.1.45]	-0.03	5.2E-03	5.6E-03	-0.14	
K03385	nitrite reductase (cytochrome c-552) [EC:1.7.2.2]	-0.03	5.7E-03	6.1E-03	-0.04	
K08679	UDP-glucuronate 4-epimerase [EC:5.1.3.6]	0.03	5.9E-03	6.3E-03	0.05	
K01658	anthranilate synthase component II [EC:4.1.3.27]	-0.03	6.9E-03	7.4E-03	-0.10	
K01613	phosphatidylserine decarboxylase [EC:4.1.1.65]	-0.03	7.0E-03	7.5E-03	-0.02	
K00688	glycogen phosphorylase [EC:2.4.1.1]	-0.03	7.7E-03	8.2E-03	-0.13	
K01442	choloylglycine hydrolase [EC:3.5.1.24]	0.03	8.4E-03	9.0E-03	0.04	yes
K08693	2',3'-cyclic-nucleotide 2'-phosphodiesterase / 3'-nucleotidase / 5'-nucleotidase [EC:3.1.4.16 3.1.3.6 3.1.3.5]	0.03	8.6E-03	9.2E-03	0.07	
K01318	glutamyl endopeptidase [EC:3.4.21.19]	0.03	8.7E-03	9.3E-03	0.02	yes
K06969	23S rRNA (cytosine1962-C5)-methyltransferase [EC:2.1.1.191]	0.03	9.3E-03	9.9E-03	0.06	
K02120	V/A-type H+/Na+-transporting ATPase subunit D	-0.03	9.4E-03	1.0E-02	-0.07	
K01738	cysteine synthase [EC:2.5.1.47]	-0.03	9.9E-03	1.1E-02	-0.05	
K13873	L-arabinose 1-dehydrogenase [EC:1.1.1.376]	0.03	1.1E-02	1.2E-02	0.01	
K01854	UDP-galactopyranose mutase [EC:5.4.99.9]	0.03	1.2E-02	1.3E-02	-0.08	
K01813	L-rhamnose isomerase [EC:5.3.1.14]	-0.03	1.2E-02	1.3E-02	-0.16	
K00945	CMP/dCMP kinase [EC:2.7.4.25]	-0.03	1.5E-02	1.6E-02	-0.02	
K00129	aldehyde dehydrogenase (NAD(P)+) [EC:1.2.1.5]	0.03	1.6E-02	1.7E-02	0.01	
K09650	rhomboid-like protein [EC:3.4.21.105]	-0.03	1.6E-02	1.7E-02	-0.04	
K07408	cytochrome P450 family 1 subfamily A1 [EC:1.14.14.1]	0.03	1.8E-02	1.9E-02	0.02	
K00485	dimethylaniline monooxygenase (N-oxide forming) [EC:1.14.13.8]	0.03	1.8E-02	1.9E-02	0.02	
K14155	cysteine-S-conjugate beta-lyase [EC:4.4.1.13]	-0.03	1.9E-02	2.0E-02	-0.06	
K00844	hexokinase [EC:2.7.1.1]	-0.03	2.0E-02	2.1E-02	-0.03	
K11175	phosphoribosylglycinamide formyltransferase 1 [EC:2.1.2.2]	0.03	2.3E-02	2.4E-02	0.09	
K03786	3-dehydroquinate dehydratase II [EC:4.2.1.10]	0.03	2.3E-02	2.4E-02	0.05	yes
K00986	RNA-directed DNA polymerase [EC:2.7.7.49]	-0.03	2.6E-02	2.8E-02	-0.16	
K02778	glucose PTS system EIIB component [EC:2.7.1.199]	0.03	2.8E-02	3.0E-02	0.04	
K01666	4-hydroxy 2-oxovalerate aldolase [EC:4.1.3.39]	-0.03	2.8E-02	3.0E-02	-0.18	
K01433	formyltetrahydrofolate deformylase [EC:3.5.1.10]	-0.03	2.8E-02	3.0E-02	-0.18	
K11381	2-oxoisovalerate dehydrogenase E1 component [EC:1.2.4.4]	-0.03	2.9E-02	3.1E-02	-0.02	
K15532	unsaturated rhamnogalacturonyl hydrolase [EC:3.2.1.172]	-0.03	3.1E-02	3.3E-02	-0.16	
K07248	lactaldehyde dehydrogenase / glycolaldehyde dehydrogenase [EC:1.2.1.22 1.2.1.21]	-0.03	3.1E-02	3.3E-02	-0.18	
K01023	arylsulfate sulfotransferase [EC:2.8.2.22]	-0.03	3.5E-02	3.7E-02	-0.18	
K02372	3-hydroxyacyl-[acyl-carrier-protein] dehydratase [EC:4.2.1.59]	-0.02	3.8E-02	4.0E-02	-0.14	

Predictor	Description	Pearson's r	P	FDR-adjusted P	Spearman's rho	Associated with fractures (P<0.05)
K05525	linalool 8-monooxygenase [EC:1.14.14.84]	0.02	3.8E-02	4.0E-02	0.07	
K01873	valyl-tRNA synthetase [EC:6.1.1.9]	0.02	3.8E-02	4.0E-02	0.04	
K11323	histone arginine demethylase JMJD6 [EC:1.14.11.-]	0.02	3.9E-02	4.1E-02	0.00	
K12410	NAD-dependent deacetylase [EC:2.3.1.286]	-0.02	3.9E-02	4.1E-02	-0.01	
K02110	F-type H+-transporting ATPase subunit c	0.02	4.0E-02	4.2E-02	0.03	
K09474	acid phosphatase (class A) [EC:3.1.3.2]	-0.02	4.0E-02	4.2E-02	-0.18	
K00184	dimethyl sulfoxide reductase iron-sulfur subunit	0.02	4.0E-02	4.2E-02	0.03	
K01654	N-acetylneuraminate synthase [EC:2.5.1.56]	-0.02	4.1E-02	4.3E-02	-0.13	
K00661	maltose O-acetyltransferase [EC:2.3.1.79]	-0.02	4.2E-02	4.4E-02	-0.12	yes
K05573	NAD(P)H-quinone oxidoreductase subunit 2 [EC:7.1.1.2]	-0.02	4.3E-02	4.5E-02	-0.02	
K10677	inulin fructotransferase (DFA-I-forming) [EC:4.2.2.17]	0.02	4.5E-02	4.7E-02	0.01	
K01776	glutamate racemase [EC:5.1.1.3]	-0.02	4.5E-02	4.7E-02	-0.04	
K11945	extradiol dioxygenase [EC:1.13.11.-]	-0.02	4.6E-02	4.8E-02	-0.03	
K01695	tryptophan synthase alpha chain [EC:4.2.1.20]	0.02	4.9E-02	5.2E-02	0.02	
K08080	CMP-N-acetylneuraminate monooxygenase [EC:1.14.18.2]	0.02	5.0E-02	5.3E-02	0.01	yes
K13307	dTDP-3-amino-3,6-dideoxy-alpha-D-glucopyranose N,N-dimethyltransferase [EC:2.1.1.235]	0.02	5.1E-02	5.4E-02	0.01	
K15944	nogalaviketone/aklaviketone reductase [EC:1.1.1.- 1.1.1.362]	0.02	5.1E-02	5.4E-02	0.01	
K01609	indole-3-glycerol phosphate synthase [EC:4.1.1.48]	-0.02	5.5E-02	5.8E-02	-0.07	
K00262	glutamate dehydrogenase (NADP+) [EC:1.4.1.4]	0.02	5.7E-02	6.0E-02	0.04	
K01153	type I restriction enzyme, R subunit [EC:3.1.21.3]	-0.02	5.7E-02	6.0E-02	-0.12	
K01012	biotin synthase [EC:2.8.1.6]	0.02	5.8E-02	6.1E-02	0.04	
K05574	NAD(P)H-quinone oxidoreductase subunit 3 [EC:7.1.1.2]	0.02	5.9E-02	6.2E-02	0.05	
K11944	PAH dioxygenase small subunit [EC:1.13.11.-]	-0.02	6.0E-02	6.3E-02	-0.03	
K07304	peptide-methionine (S)-S-oxide reductase [EC:1.8.4.11]	-0.02	7.5E-02	7.9E-02	-0.17	
K01051	pectinesterase [EC:3.1.1.11]	-0.02	7.6E-02	8.0E-02	-0.14	
K01834	2,3-bisphosphoglycerate-dependent phosphoglycerate mutase [EC:5.4.2.11]	0.02	7.7E-02	8.1E-02	-0.02	yes
K13002	glycosyltransferase [EC:2.4.1.-]	-0.02	7.7E-02	8.1E-02	-0.18	
K01183	chitinase [EC:3.2.1.14]	-0.02	7.7E-02	8.1E-02	-0.17	
K01950	NAD+ synthase (glutamine-hydrolysing) [EC:6.3.5.1]	0.02	7.8E-02	8.2E-02	0.05	
K13000	mannosyltransferase [EC:2.4.1.-]	-0.02	7.9E-02	8.3E-02	-0.03	
K01657	anthranilate synthase component I [EC:4.1.3.27]	0.02	8.1E-02	8.5E-02	0.01	
K13051	L-asparaginase / beta-aspartyl-peptidase [EC:3.5.1.1 3.4.19.5]	0.02	8.2E-02	8.6E-02	-0.11	
K16214	UDP-N-acetylglucosamine kinase [EC:2.7.1.176]	0.02	8.2E-02	8.6E-02	0.02	
K01730	oligogalacturonide lyase [EC:4.2.2.6]	-0.02	8.3E-02	8.7E-02	-0.18	
K00033	6-phosphogluconate dehydrogenase [EC:1.1.1.44 1.1.1.343]	-0.02	8.6E-02	9.0E-02	-0.10	
K09680	type II pantothenate kinase [EC:2.7.1.33]	-0.02	8.8E-02	9.2E-02	-0.17	
K01925	UDP-N-acetylmuramoylalanine--D-glutamate ligase [EC:6.3.2.9]	-0.02	9.0E-02	9.4E-02	-0.01	
K04068	anaerobic ribonucleoside-triphosphate reductase activating protein [EC:1.97.1.4]	0.02	9.4E-02	9.8E-02	0.05	
K03469	ribonuclease HI [EC:3.1.26.4]	0.02	9.5E-02	9.9E-02	0.02	
K00645	[acyl-carrier-protein] S-malonyltransferase [EC:2.3.1.39]	0.02	9.6E-02	1.0E-01	0.08	
K12904	phosphonoacetaldehyde reductase [EC:1.1.1.309]	-0.02	9.7E-02	1.0E-01	-0.03	
K12507	acyl-CoA synthetase [EC:6.2.1.-]	0.02	1.0E-01	1.0E-01	0.02	

Predictor	Description	Pearson's r	P	FDR-adjusted P	Spearman's rho	Associated with fractures (P<0.05)
K01235	alpha-glucuronidase [EC:3.2.1.139]	-0.02	1.0E-01	1.0E-01	-0.17	
K03823	phosphinothricin acetyltransferase [EC:2.3.1.183]	-0.02	1.0E-01	1.0E-01	-0.05	
K01955	carbamoyl-phosphate synthase large subunit [EC:6.3.5.5]	-0.02	1.1E-01	1.1E-01	-0.10	
K01126	glycerophosphoryl diester phosphodiesterase [EC:3.1.4.46]	0.02	1.1E-01	1.1E-01	0.03	
K03168	DNA topoisomerase I [EC:5.6.2.1]	-0.02	1.1E-01	1.1E-01	0.01	
K15241	tetrachlorohydroquinone reductive dehalogenase [EC:1.21.4.5]	0.02	1.1E-01	1.1E-01	0.00	
K11948	1-hydroxy-2-naphthoate dioxygenase [EC:1.13.11.38]	-0.02	1.1E-01	1.1E-01	-0.03	
K11947	aldehyde dehydrogenase [EC:1.2.1.-]	-0.02	1.1E-01	1.1E-01	-0.03	
K11946	hydratase-aldolase [EC:4.1.2.-]	-0.02	1.1E-01	1.1E-01	-0.03	
K11943	PAH dioxygenase large subunit [EC:1.13.11.-]	-0.02	1.1E-01	1.1E-01	-0.03	
K12745	deacetoxycephalosporin-C hydroxylase [EC:1.14.11.26]	-0.02	1.2E-01	1.2E-01	-0.03	
K12744	deacetoxycephalosporin-C synthase [EC:1.14.20.1]	-0.02	1.2E-01	1.2E-01	-0.03	
K04126	isopenicillin-N synthase [EC:1.21.3.1]	-0.02	1.2E-01	1.2E-01	-0.03	
K12743	N-(5-amino-5-carboxypentanoyl)-L-cysteinyl-D-valine synthase [EC:6.3.2.26]	-0.02	1.2E-01	1.2E-01	-0.03	
K03741	arsenate reductase (thioredoxin) [EC:1.20.4.4]	-0.02	1.2E-01	1.2E-01	-0.17	
K12567	titin [EC:2.7.11.1]	0.02	1.3E-01	1.3E-01	0.00	
K04567	lysyl-tRNA synthetase, class II [EC:6.1.1.6]	-0.02	1.4E-01	1.5E-01	0.03	
K00705	4-alpha-glucanotransferase [EC:2.4.1.25]	0.02	1.4E-01	1.5E-01	0.03	
K00616	transaldolase [EC:2.2.1.2]	-0.02	1.5E-01	1.6E-01	-0.03	
K01811	alpha-D-xyloside xylohydrolase [EC:3.2.1.177]	-0.02	1.6E-01	1.7E-01	-0.17	
K01775	alanine racemase [EC:5.1.1.1]	-0.02	1.6E-01	1.7E-01	0.06	
K01533	P-type Cu2+ transporter [EC:7.2.2.9]	-0.02	1.6E-01	1.7E-01	-0.01	yes
K00946	thiamine-monophosphate kinase [EC:2.7.4.16]	0.02	1.7E-01	1.8E-01	0.04	
K13821	RHH-type transcriptional regulator, proline utilization regulon repressor / proline dehydrogenase / delta 1-pyrroline-5-carboxylate dehydrogenase [EC:1.5.5.2 1.2.1.88]	-0.02	1.9E-01	2.0E-01	-0.16	
K03818	putative colanic acid biosynthesis acetyltransferase WcaF [EC:2.3.1.-]	-0.02	1.9E-01	2.0E-01	-0.16	
K03218	23S rRNA (guanosine2251-2'-O)-methyltransferase [EC:2.1.1.185]	-0.02	2.0E-01	2.1E-01	0.01	
K00231	protoporphyrinogen/coproporphyrinogen III oxidase [EC:1.3.3.4 1.3.3.15]	-0.02	2.0E-01	2.1E-01	-0.01	
K00684	leucyl/phenylalanyl-tRNA---protein transferase [EC:2.3.2.6]	-0.02	2.0E-01	2.1E-01	-0.16	
K03581	exodeoxyribonuclease V alpha subunit [EC:3.1.11.5]	0.02	2.1E-01	2.2E-01	-0.05	
K03885	NADH:ubiquinone reductase (H+-translocating) [EC:7.1.1.2]	0.02	2.1E-01	2.2E-01	0.02	
K01915	glutamine synthetase [EC:6.3.1.2]	-0.01	2.2E-01	2.3E-01	-0.09	yes
K03536	ribonuclease P protein component [EC:3.1.26.5]	0.01	2.2E-01	2.3E-01	0.01	yes
K01265	methionyl aminopeptidase [EC:3.4.11.18]	-0.01	2.2E-01	2.3E-01	-0.08	yes
K00326	cytochrome-b5 reductase [EC:1.6.2.2]	0.01	2.3E-01	2.4E-01	0.01	yes
K05929	phosphoethanolamine N-methyltransferase [EC:2.1.1.103]	0.01	2.3E-01	2.4E-01	0.01	yes
K01518	bis(5'-nucleosidyl)-tetraphosphatase [EC:3.6.1.17]	0.01	2.4E-01	2.5E-01	0.01	yes
K03527	4-hydroxy-3-methylbut-2-en-1-yl diphosphate reductase [EC:1.17.7.4]	0.01	2.4E-01	2.5E-01	0.07	
K01897	long-chain acyl-CoA synthetase [EC:6.2.1.3]	-0.01	2.4E-01	2.5E-01	-0.09	yes
K01151	deoxyribonuclease IV [EC:3.1.21.2]	0.01	2.4E-01	2.5E-01	0.07	
K02782	glucitol/sorbitol PTS system EIIB component [EC:2.7.1.198]	-0.01	2.6E-01	2.7E-01	-0.03	
K11987	prostaglandin-endoperoxide synthase 2 [EC:1.14.99.1]	-0.01	2.6E-01	2.7E-01	0.00	
K00428	cytochrome c peroxidase [EC:1.11.1.5]	-0.01	2.7E-01	2.8E-01	-0.01	yes

Predictor	Description	Pearson's r	P	FDR-adjusted P	Spearman's rho	Associated with fractures (P<0.05)
K08303	U32 family peptidase [EC:3.4.-.-]	0.01	2.7E-01	2.8E-01	0.08	
K01127	glycosylphosphatidylinositol phospholipase D [EC:3.1.4.50]	0.01	2.8E-01	2.9E-01	0.01	yes
K02112	F-type H+/Na+-transporting ATPase subunit beta [EC:7.1.2.2 7.2.2.1]	-0.01	2.9E-01	3.0E-01	-0.04	
K01372	bleomycin hydrolase [EC:3.4.22.40]	-0.01	2.9E-01	3.0E-01	-0.10	
K03470	ribonuclease HII [EC:3.1.26.4]	-0.01	2.9E-01	3.0E-01	0.01	
K00012	UDPglucose 6-dehydrogenase [EC:1.1.1.22]	0.01	3.0E-01	3.1E-01	0.08	
K03551	holliday junction DNA helicase RuvB [EC:3.6.4.12]	-0.01	3.0E-01	3.1E-01	0.04	
K02169	malonyl-CoA O-methyltransferase [EC:2.1.1.197]	-0.01	3.0E-01	3.1E-01	-0.15	
K16593	pimeloyl-[acyl-carrier protein] synthase [EC:1.14.14.46]	-0.01	3.2E-01	3.3E-01	0.03	
K03043	DNA-directed RNA polymerase subunit beta [EC:2.7.7.6]	-0.01	3.4E-01	3.5E-01	-0.06	
K01154	type I restriction enzyme, S subunit [EC:3.1.21.3]	-0.01	3.4E-01	3.5E-01	-0.13	
K01685	altronate hydrolase [EC:4.2.1.7]	-0.01	3.5E-01	3.6E-01	-0.12	
K06989	aspartate dehydrogenase [EC:1.4.1.21]	-0.01	3.5E-01	3.6E-01	-0.15	
K00041	tagaturonate reductase [EC:1.1.1.58]	-0.01	3.5E-01	3.6E-01	-0.12	
K01338	ATP-dependent Lon protease [EC:3.4.21.53]	-0.01	3.5E-01	3.6E-01	0.01	
K11633	two-component system, OmpR family, sensor histidine kinase YxdK [EC:2.7.13.3]	0.01	3.7E-01	3.8E-01	0.06	
K01620	threonine aldolase [EC:4.1.2.48]	0.01	3.8E-01	3.9E-01	0.00	
K00868	pyridoxine kinase [EC:2.7.1.35]	-0.01	3.8E-01	3.9E-01	-0.05	
K01735	3-dehydroquinate synthase [EC:4.2.3.4]	-0.01	3.9E-01	4.0E-01	-0.01	
K01488	adenosine deaminase [EC:3.5.4.4]	0.01	3.9E-01	4.0E-01	0.01	
K03940	NADH dehydrogenase (ubiquinone) Fe-S protein 7 [EC:7.1.1.2]	-0.01	4.1E-01	4.2E-01	-0.01	
K01734	methylglyoxal synthase [EC:4.2.3.3]	-0.01	4.2E-01	4.3E-01	0.00	
K01783	ribulose-phosphate 3-epimerase [EC:5.1.3.1]	-0.01	4.3E-01	4.4E-01	0.03	
K01390	IgA-specific metalloendopeptidase [EC:3.4.24.13]	0.01	4.4E-01	4.5E-01	0.04	
K01006	pyruvate, orthophosphate dikinase [EC:2.7.9.1]	-0.01	4.4E-01	4.5E-01	0.03	
K02343	DNA polymerase III subunit gamma/tau [EC:2.7.7.7]	0.01	4.5E-01	4.6E-01	0.00	
K00031	isocitrate dehydrogenase [EC:1.1.1.42]	-0.01	4.6E-01	4.7E-01	-0.03	
K11214	sedoheptulokinase [EC:2.7.1.14]	0.01	4.6E-01	4.7E-01	0.06	
K01091	phosphoglycolate phosphatase [EC:3.1.3.18]	0.01	4.6E-01	4.7E-01	0.04	
K00773	queuine tRNA-ribosyltransferase [EC:2.4.2.29]	0.01	4.7E-01	4.8E-01	0.06	
K05995	dipeptidase E [EC:3.4.13.21]	-0.01	4.7E-01	4.8E-01	-0.08	
K12308	beta-galactosidase [EC:3.2.1.23]	0.01	4.9E-01	5.0E-01	0.02	
K00290	saccharopine dehydrogenase (NAD+, L-lysine forming) [EC:1.5.1.7]	-0.01	4.9E-01	5.0E-01	0.03	
K13747	carboxynorspermidine decarboxylase [EC:4.1.1.96]	-0.01	5.2E-01	5.3E-01	0.03	
K08352	thiosulfate reductase / polysulfide reductase chain A [EC:1.8.5.5]	0.01	5.3E-01	5.4E-01	0.02	
K13252	putrescine carbamoyltransferase [EC:2.1.3.6]	0.01	5.3E-01	5.4E-01	0.01	
K01951	GMP synthase (glutamine-hydrolysing) [EC:6.3.5.2]	0.01	5.4E-01	5.5E-01	-0.06	
K01647	citrate synthase [EC:2.3.3.1]	-0.01	5.5E-01	5.6E-01	0.04	
K00701	cyclomaltodextrin glucanotransferase [EC:2.4.1.19]	0.01	5.6E-01	5.7E-01	0.00	
K00819	ornithine--oxo-acid transaminase [EC:2.6.1.13]	-0.01	5.7E-01	5.8E-01	-0.14	
K16038	N-methyltransferase [EC:2.1.1.-]	0.01	5.8E-01	5.9E-01	-0.01	yes
K00793	riboflavin synthase [EC:2.5.1.9]	-0.01	6.0E-01	6.1E-01	0.04	

Predictor	Description	Pearson's r	P	FDR-adjusted P	Spearman's rho	Associated with fractures (P<0.05)
K00278	L-aspartate oxidase [EC:1.4.3.16]	0.01	6.0E-01	6.1E-01	0.06	
K01181	endo-1,4-beta-xylanase [EC:3.2.1.8]	-0.01	6.1E-01	6.2E-01	-0.16	
K00265	glutamate synthase (NADPH) large chain [EC:1.4.1.13]	-0.01	6.1E-01	6.2E-01	-0.15	
K11628	mycocerosic acid synthase [EC:2.3.1.111]	-0.01	6.3E-01	6.4E-01	-0.03	
K12445	trans enoyl reductase [EC:1.3.1.-]	-0.01	6.3E-01	6.4E-01	-0.03	
K07682	two-component system, NarL family, sensor histidine kinase DevS [EC:2.7.13.3]	-0.01	6.3E-01	6.4E-01	-0.03	
K00864	glycerol kinase [EC:2.7.1.30]	0.01	6.4E-01	6.5E-01	-0.10	
K00028	malate dehydrogenase (decarboxylating) [EC:1.1.1.39]	0.01	6.5E-01	6.6E-01	-0.01	
K01551	arsenite/tail-anchored protein-transporting ATPase [EC:7.3.2.7 7.3.-.-]	-0.01	6.6E-01	6.7E-01	-0.16	
K01770	2-C-methyl-D-erythritol 2,4-cyclodiphosphate synthase [EC:4.6.1.12]	0.01	6.7E-01	6.8E-01	0.02	yes
K00297	methylenetetrahydrofolate reductase (NADH) [EC:1.5.1.54]	-0.01	6.7E-01	6.8E-01	-0.11	
K15633	2,3-bisphosphoglycerate-independent phosphoglycerate mutase [EC:5.4.2.12]	0.00	6.8E-01	6.9E-01	0.05	
K12674	(carboxyethyl)arginine beta-lactam-synthase [EC:6.3.3.4]	0.00	7.0E-01	7.1E-01	-0.01	
K12675	clavaminate synthase [EC:1.14.11.21]	0.00	7.0E-01	7.1E-01	-0.01	
K12673	N2-(2-carboxyethyl)arginine synthase [EC:2.5.1.66]	0.00	7.0E-01	7.1E-01	-0.01	
K00093	methanol dehydrogenase [EC:1.1.1.244]	0.00	7.2E-01	7.3E-01	-0.03	
K00189	2-oxoisovalerate/pyruvate ferredoxin oxidoreductase gamma subunit [EC:1.2.7.7 1.2.7.1]	0.00	7.2E-01	7.3E-01	-0.02	
K01143	exodeoxyribonuclease (lambda-induced) [EC:3.1.11.3]	0.00	7.5E-01	7.5E-01	-0.01	
K03280	UDP-N-acetylglucosamine:(glucosyl)LPS alpha-1,2-N-acetylglucosaminyltransferase [EC:2.4.1.56]	0.00	7.5E-01	7.5E-01	-0.01	yes
K03587	cell division protein FtsI (penicillin-binding protein 3) [EC:3.4.16.4]	0.00	7.5E-01	7.5E-01	-0.01	
K09459	phosphonopyruvate decarboxylase [EC:4.1.1.82]	0.00	7.6E-01	7.6E-01	0.01	
K00999	CDP-diacylglycerol--inositol 3-phosphatidyltransferase [EC:2.7.8.11]	0.00	7.9E-01	7.9E-01	-0.02	
K14467	4-hydroxybutyrate---CoA ligase (AMP-forming) [EC:6.2.1.40]	0.00	8.1E-01	8.1E-01	-0.01	
K14471	succinyl-CoA:(S)-malate CoA-transferase subunit A [EC:2.8.3.22]	0.00	8.2E-01	8.2E-01	-0.02	
K14459	hexosaminidase [EC:3.2.1.52]	0.00	8.3E-01	8.3E-01	-0.03	
K16035	7-O-carbamoyltransferase [EC:2.1.3.-]	0.00	8.3E-01	8.3E-01	-0.01	yes
K00147	glutamate-5-semialdehyde dehydrogenase [EC:1.2.1.41]	0.00	8.3E-01	8.3E-01	0.01	
K00613	glycine amidinotransferase [EC:2.1.4.1]	0.00	8.6E-01	8.6E-01	0.00	
K06131	cardiolipin synthase A/B [EC:2.7.8.-]	0.00	8.7E-01	8.7E-01	0.04	
K01953	asparagine synthase (glutamine-hydrolysing) [EC:6.3.5.4]	0.00	8.9E-01	8.9E-01	-0.02	
K01243	adenosylhomocysteine nucleosidase [EC:3.2.2.9]	0.00	8.9E-01	8.9E-01	-0.02	
K01841	phosphoenolpyruvate phosphomutase [EC:5.4.2.9]	0.00	8.9E-01	8.9E-01	0.01	
K11410	short-chain 2-methylacyl-CoA dehydrogenase [EC:1.3.8.5]	0.00	8.9E-01	8.9E-01	-0.02	
K01531	P-type Mg2+ transporter [EC:7.2.2.14]	0.00	8.9E-01	8.9E-01	0.00	
K01733	threonine synthase [EC:4.2.3.1]	0.00	9.1E-01	9.1E-01	-0.01	
K00280	lysyl oxidase-like protein 2/3/4 [EC:1.4.3.-]	0.00	9.3E-01	9.3E-01	-0.01	
K14157	alpha-aminoadipic semialdehyde synthase [EC:1.5.1.8 1.5.1.9]	0.00	9.7E-01	9.7E-01	-0.02	
K01835	phosphoglucomutase [EC:5.4.2.2]	0.00	9.8E-01	9.8E-01	0.04	
K07056	16S rRNA (cytidine1402-2'-O)-methyltransferase [EC:2.1.1.198]	0.00	1.0E+00	1.0E+00	0.03	

Supplementary Table 22. Top 25 functional groups correlated with the relative abundance of Tenericutes and associated with fractures

Predictor	Description	Pearson's r	P	FDR-adjusted P	Spearman's rho	Associated with fractures (P< 0.05)	Implicated in butyrate metabolism
K08651	thermitase [EC:3.4.21.66]	0.45	<5.9E-323	8.9E-320	0.52	yes	
K15898	pseudaminic acid synthase [EC:2.5.1.97]	0.44	<5.9E-323	8.9E-320	0.48	yes	
K00100	butanol dehydrogenase [EC:1.1.1.-]	0.43	4.4E-318	4.4E-315	0.47	yes	yes
K00354	NADPH2 dehydrogenase [EC:1.6.99.1]	0.42	4.7E-304	3.5E-301	0.45	yes	
K14128	F420-non-reducing hydrogenase small subunit [EC:1.12.99.- 1.8.98.5]	0.42	6.2E-294	3.7E-291	0.46	yes	
K14080	[methyl-Co(III) methanol/glycine betaine-specific corrinoid protein]:coenzyme M methyltransferase [EC:2.1.1.246 2.1.1.377]	0.42	2.2E-292	1.1E-289	0.45	yes	
K14126	F420-non-reducing hydrogenase large subunit [EC:1.12.99.- 1.8.98.5]	0.42	3.5E-292	1.5E-289	0.46	yes	
K00532	ferredoxin hydrogenase [EC:1.12.7.2]	0.41	1.3E-288	4.9E-286	0.44	yes	
K01959	pyruvate carboxylase subunit A [EC:6.4.1.1]	0.41	3.3E-286	1.1E-283	0.45	yes	
K03462	nicotinamide phosphoribosyltransferase [EC:2.4.2.12]	0.41	1.3E-283	3.9E-281	0.44	yes	
K00021	hydroxymethylglutaryl-CoA reductase (NADPH) [EC:1.1.1.34]	0.41	6.9E-281	1.7E-278	0.45	yes	
K00150	glyceraldehyde-3-phosphate dehydrogenase (NAD(P)) [EC:1.2.1.59]	0.41	1.4E-280	3.2E-278	0.44	yes	
K00263	leucine dehydrogenase [EC:1.4.1.9]	0.41	3.7E-278	8.0E-276	0.43	yes	
K12454	CDP-paratose 2-epimerase [EC:5.1.3.10]	0.40	1.6E-274	3.0E-272	0.43	yes	
K00404	cytochrome c oxidase cbb3-type subunit I [EC:7.1.1.9]	0.40	2.6E-274	4.6E-272	0.42	yes	
K07651	two-component system, OmpR family, sensor histidine kinase ResE [EC:2.7.13.3]	0.40	5.7E-274	9.6E-272	0.43	yes	
K04566	lysyl-tRNA synthetase, class I [EC:6.1.1.6]	0.40	1.3E-273	2.1E-271	0.44	yes	
K00809	deoxyhypusine synthase [EC:2.5.1.46]	0.40	2.4E-273	3.6E-271	0.44	yes	
K10747	DNA ligase 1 [EC:6.5.1.1 6.5.1.6 6.5.1.7]	0.40	4.5E-273	6.5E-271	0.44	yes	
K13282	cyanophycinase [EC:3.4.15.6]	0.40	1.6E-272	2.2E-270	0.43	yes	
K01622	fructose 1,6-bisphosphate aldolase/phosphatase [EC:4.1.2.13 3.1.3.11]	0.40	2.7E-272	3.5E-270	0.44	yes	
K00202	formylmethanofuran dehydrogenase subunit C [EC:1.2.7.12]	0.40	3.7E-271	4.7E-269	0.44	yes	
K02277	cytochrome c oxidase subunit IV [EC:7.1.1.9]	0.40	1.7E-269	2.1E-267	0.42	yes	
K00372	assimilatory nitrate reductase catalytic subunit [EC:1.7.99.-]	0.40	5.7E-269	6.6E-267	0.42	yes	
K00952	nicotinamide-nucleotide adenylyltransferase [EC:2.7.7.1]	0.40	3.1E-268	3.5E-266	0.43	yes	

Pearson's correlation between functional groups and the relative abundance of Tenericutes. n = 7043 (1092 cases). FDR, false discovery rate

Supplementary Table 23. Incident fracture case definitions

Fracture type	ICD-10
Hip fractures	S72.0, S72.1, S72.2
Major osteoporotic fractures	S22.0, S22.1,<br>S32.0,<br>S42.2,<br>S52.5, S52.6,<br>S72.0, S72.1, S72.2
Any fracture	S02, S12, S22, S32, S42, S52, S62, S72, S82, S92
